# Supplementary material for: qTAG: an adaptable plasmid scaffold for CRISPR-based endogenous tagging
Source: EMBO J. 2024 Dec 12;44(3):947–74. doi: 10.1038/s44318-024-00337-5 (PMC11790981; doi:10.1038/s44318-024-00337-5)
Supplement: Supplementary file 1 — Table EV1 [file 44318_2024_337_MOESM1_ESM.docx]

**Table EV1. Sources of Tag Sequences**

| **Tag** | **Source Plasmid** | **Source Study** | **Modifications** |
| --- | --- | --- | --- |
| Cterm mStayGold | <https://www.addgene.org/212019/> | (Ando et al.)(Ando *et al.*, 2023) | Removal of internal restriction sites, codon optimization |
| Nterm mStayGold | <https://www.addgene.org/212018/> | (Ando et al.)(Ando *et al.*, 2023) | Removal of internal restriction sites, codon optimization |
| mNeon | <https://www.addgene.org/58179/> | (Shaner et al.)(Shaner *et al.*, 2013) | Removal of internal restriction sites, codon optimization |
| moxGFP | <https://www.addgene.org/68070/> | (Costantini et al.)(Costantini *et al.*, 2015) | Removal of internal restriction sites, codon optimization |
| mScarlet | <https://www.addgene.org/85042/> | (Bindels et al.)(Bindels *et al.*, 2017) | Removal of internal restriction sites, codon optimization |
| super-TagRFP | <https://www.addgene.org/173014/> | (Mo et al.)(Mo *et al.*, 2020) | Removal of internal restriction sites, codon optimization |
| miRFP670nano3 | <https://www.addgene.org/184664/> | (Oliinyk et al.)(Oliinyk *et al.*, 2022) | Removal of internal restriction sites, codon optimization |
| miniTurbo | <https://www.addgene.org/107174/> | (Branon et al.)(Branon *et al.*, 2018) | Removal of internal restriction sites, codon optimization |
| ultraID | <https://www.addgene.org/172878/> | (Kubitz et al.)(Kubitz *et al.*, 2022) | Removal of internal restriction sites, codon optimization |
| dTAG | <https://www.addgene.org/91797/> | (Nabet et al.)(Nabet *et al.*, 2018) | Removal of internal restriction sites, codon optimization |
| 3xFLAG | Synthesized | - | - |
| 3xHA | Synthesized | - | - |
| V5 | Synthesized | - | - |
