# Supplementary material for: qTAG: an adaptable plasmid scaffold for CRISPR-based endogenous tagging
Source: EMBO J. 2024 Dec 12;44(3):947–74. doi: 10.1038/s44318-024-00337-5 (PMC11790981; doi:10.1038/s44318-024-00337-5)
Supplement: Supplementary file 2 — Table EV2 [file 44318_2024_337_MOESM2_ESM.docx]

**Table EV2. qTAG relevant sequences and primers**

| **Sequences (5’ - 3')** | **Purpose** | **Description** |
| --- | --- | --- |
| TTGTAAAACGACGGCCAGTGAATTC | HDR | Nterm **LHA** Left Gibson Cloning Sequence (Top Strand) |
| GCTACCGGTCTTGCTCTTGCC | HDR | Nterm **LHA** Right Gibson Cloning Sequence (Bottom Strand) |
| AGGAAGCGGAGGATCTGGCGGTACC | HDR | Nterm **RHA** Left Gibson Cloning Sequence (Top Strand) |
| CTATGACCATGATTACGCCAAGCTT | HDR | Nterm **RHA** Right Gibson Cloning Sequence (Bottom Strand) |
| TTGTAAAACGACGGCCAGTGAATTC | HDR | Cterm and KO **LHA** Left Gibson Cloning Sequence (Top Strand) |
| TACCGCCAGATCCTCCGCTTCCTCC | HDR | Cterm and KO **LHA** Right Gibson Cloning Sequence (Bottom Strand) |
| CTCGAGACCGGTAAGAGCAAGTGA | HDR | Cterm and KO **RHA** Left Gibson Cloning Sequence (Top Strand) |
| CTATGACCATGATTACGCCAAGCTT | HDR | Cterm and KO **RHA** Right Gibson Cloning Sequence (Bottom Strand) |
| acagctGAATTCGCATCGTACGCGTACGTG  TTTGGNNNNNNNNNNNNNNNNNNNNGGC  AAGAGCAAGACCGGTAGC | MMEJ | Nterm MMEJ Fw Cassette Primer (Replace 'N') (Top Strand) |
| agctgtAAGCTTGCATCGTACGCGTACGTG  TTTGGNNNNNNNNNNNNNNNNNNNNGGTA  CCGCCAGATCCTCCG | MMEJ | Nterm MMEJ Rev Cassette Primer (Replace 'N') (Bottom Strand) |
| acagctGAATTCGCATCGTACGCGTACGTG  TTTGGNNNNNNNNNNNNNNNNNNNNGGA  GGAAGCGGAGGATCTGGC | MMEJ | Cterm MMEJ Fw Cassette Primer (Replace 'N') (Top Strand) |
| agctgtAAGCTTGCATCGTACGCGTACGTG  TTTGGNNNNNNNNNNNNNNNNNNNNTCAC  TTGCTCTTACCGGTCTCG | MMEJ | Cterm MMEJ Rev Cassette Primer (Replace 'N') (Bottom Strand) |
| GGCAAGAGCAAGACCGGTAGC | Amplification + Sequencing | Nterm Cassette Fw Amplification & Sequencing Primer (Top Strand) |
| GGTACCGCCAGATCCTCCG | Amplification + Sequencing | Nterm Cassette Rev Amplification & Sequencing Primer (Bottom) |
| GGAGGAAGCGGAGGATCTGGC | Amplification + Sequencing | Cterm Cassette Fw Amplification & Sequencing Primer (Top Strand) |
| TCACTTGCTCTTACCGGTCTCG | Amplification + Sequencing | Cterm Cassette Rev Amplification & Sequencing Primer (Bottom Strand) |
| GCTACTAACTTCAGCCTGCTGAAGC | Sequencing | P2A Fw Sequencing Primer (Top Strand) |
| AGGTCCAGGGTTCTCCTCCAC | Sequencing | P2A Rev Sequencing Primer (Bottom Strand) |
| CTGCAAGGCGATTAAGTTGGGTAAC | Sequencing | LHA Fw Sequencing Primer (Top Strand) |
| GGCTCGTATGTTGTGTGGAATTGT | Sequencing | RHA Rev Sequencing Primer (Bottom Strand) |
| GCCTTTTTACGGTTCCTGGC | Sequencing | pX-line sgRNA Sequencing Primer (Top Strand) |
