# Supplementary material for: qTAG: an adaptable plasmid scaffold for CRISPR-based endogenous tagging
Source: EMBO J. 2024 Dec 12;44(3):947–74. doi: 10.1038/s44318-024-00337-5 (PMC11790981; doi:10.1038/s44318-024-00337-5)
Supplement: Supplementary file 3 — Table EV3 [file 44318_2024_337_MOESM3_ESM.docx]

**Table EV3. Gene Design Information**

| **Target Gene** | **Localization** | **Target Terminus** | **crRNA (5’ – 3’)** | **PAM** | **Adapted design from** |
| --- | --- | --- | --- | --- | --- |
| ARL13B | Cilia | C | CTCATAAACAAGACGTATGG | AGG | Designed in this study |
| CANX | Endoplasmic Reticulum | C | AGATTGTTTCACTCTCTTCG | TGG | OpenCell(Cho *et al.*, 2022) |
| CLTC | Coated Pits, Vesicles | C | CACATGCTGTACCCAAAGCC | AGG | OpenCell(Cho *et al.*, 2022) |
| PXN | Focal Adhesion | C | CTTCCTCAAGCTCTTCTGCT | AGG | OpenCell(Cho *et al.*, 2022) |
| PEX3 | Peroxisomes | C | TTTTAGTACCCCTCAGCAAC | TGG | OpenCell(Cho *et al.*, 2022) |
| COX8A | Mitochondria | C | CAGAACGGACCCCTTCACTC | TGG | Designed in this study |
| CEP192 | Centrosome | C | CGACTAATTGGTGAAGCTCT | TGG | OpenCell(Cho *et al.*, 2022) |
| EZR | Cell Membrane | C | TTCGAGGCCCTGTAACAGCC | AGG | OpenCell(Cho *et al.*, 2022) |
| H2BC11 | Nuclei/Chromosomes | C | ACTCACTGTTTACTTAGCGC | TGG | Allen Institute(Roberts *et al.*, 2017) |
| H3C2 | Nuclei/Chromosomes | C | ACGCTCTTTCTCCGCGAATG | CGG | Designed in this study |
| LAMP1 | Lysosomes | C | CTATCTAGCCTGGTGCACGC | AGG | Designed in this study |
| MAPRE1 | Microtubule +TIPs | C | CTTTGTGATACCTGATGAAG | GGG | Designed in this study |
| TOMM20 | Mitochondria | C | GAGCTTGGCTGAAGATGATG | TGG | OpenCell(Cho *et al.*, 2022) |
| PLK4 | Centrosome | C | AGTTTTAATCAATGAAAATT | AGG | Designed in this study |
| TUBB4B | Microtubules | C | CCTAGAGCCTTCAGTCACTG | GGG | Designed in this study |
| VIM | Intermediate Filaments | C | GCGCAAGATAGATTTGGAAT | AGG | Designed in this study |
| ACTB | Actin | N | GCCGTTGTCGACGACGAGCG | CGG | Allen Institute(Roberts *et al.*, 2017) |
| CENPA | Centromeres | N | GTGTCATGGGCCCGCGCCGC | CGG | OpenCell(Cho *et al.*, 2022) |
| CEP135 | Centrosomes | N | ATACCGCCAGACTCTGACAG | TGG | Designed in this study |
| CETN2 | Centrosomes | N | AAAGGCCTCCAACTTTAAGA | AGG | OpenCell(Cho *et al.*, 2022) |
| GOLGA2 | Golgi | N | TCGGGTTTCTTCCGACATCG | CGG | OpenCell(Cho *et al.*, 2022) |
| LMNB1 | Nuclear Envelope | N | GGGGTCGCAGTCGCCATGGC | GGG | Allen Institute(Roberts *et al.*, 2017) |
| MAP4 | Microtubules | N | TTAATGCATCTGCAAGACTG | AGG | OpenCell(Cho *et al.*, 2022) |
| MYO1C | Membrane | N | GGCGGTGAGCGCACTCTCCA | TGG | OpenCell(Cho *et al.*, 2022) |
| PCNT | Centrosome | N | TGGAAGTTGAGCAAGAGCAG | CGG | Designed in this study |
| RAB7A | Endosomes | N | TAGTTTGAAGGATGACCTCT | AGG | OpenCell(Cho *et al.*, 2022) |
| TJP1 | Tight Junctions | N | GGCCGCGGAGGCGCTCACCT | TGG | OpenCell(Cho *et al.*, 2022) |
| TUBA1B | Microtubules | N | GATGCACTCACGCTGCGGGA | AGG | Allen Institute(Roberts *et al.*, 2017) |
| TP53 | - | N | TCGACGCTAGGATCTGACTG | CGG | Designed in this study |
| AAVS1 | - | - | GGGGCCACTAGGGACAGGAT | TGG | Allen Institute(Roberts *et al.*, 2017) |
