# Supplementary material for: qTAG: an adaptable plasmid scaffold for CRISPR-based endogenous tagging
Source: EMBO J. 2024 Dec 12;44(3):947–74. doi: 10.1038/s44318-024-00337-5 (PMC11790981; doi:10.1038/s44318-024-00337-5)
Supplement: Supplementary file 4 — Table EV4 [file 44318_2024_337_MOESM4_ESM.docx]

**Table EV4. Plasmids for the qTAG system**

| **Plasmid Name** | **Terminus** | **Tag** | **Marker** | **Plasmid Information and Map** |
| --- | --- | --- | --- | --- |
| qTAG-N-Blast-mNeon | N | mNeonGreen | Blasticidin | <https://www.addgene.org/207676/> |
| qTAG-N-Blast-moxGFP | N | moxGFP | Blasticidin | <https://www.addgene.org/207677/> |
| qTAG-N-Blast-mScarlet | N | mScarlet | Blasticidin | <https://www.addgene.org/207678/> |
| qTAG-N-Blast-sTagRFP | N | super-TagRFP | Blasticidin | <https://www.addgene.org/207679/> |
| qTAG-N-Blast-miRFP670nano3 | N | miRFP670nano3 | Blasticidin | <https://www.addgene.org/207680/> |
| qTAG-N-Blast-miniTurbo | N | miniTurbo | Blasticidin | <https://www.addgene.org/207681/> |
| qTAG-N-Blast-ultraID | N | ultraID | Blasticidin | <https://www.addgene.org/207682/> |
| qTAG-N-Blast-dTAG | N | dTAG | Blasticidin | <https://www.addgene.org/207683/> |
| qTAG-N-Blast-3xFLAG | N | 3xFLAG | Blasticidin | <https://www.addgene.org/207684/> |
| qTAG-N-Blast-3xHA | N | 3xHA | Blasticidin | <https://www.addgene.org/207685/> |
| qTAG-N-Blast-V5 | N | V5 | Blasticidin | <https://www.addgene.org/207686/> |
| qTAG-N-Puro-mNeon | N | mNeonGreen | Puromycin | <https://www.addgene.org/207687/> |
| qTAG-N-Puro-moxGFP | N | moxGFP | Puromycin | <https://www.addgene.org/207688/> |
| qTAG-N-Puro-mScarlet | N | mScarlet | Puromycin | <https://www.addgene.org/207689/> |
| qTAG-N-Puro-sTagRFP | N | super-TagRFP | Puromycin | <https://www.addgene.org/207690/> |
| qTAG-N-Puro-miRFP670nano3 | N | miRFP670nano3 | Puromycin | <https://www.addgene.org/207691/> |
| qTAG-N-Puro-miniTurbo | N | miniTurbo | Puromycin | <https://www.addgene.org/207692/> |
| qTAG-N-Puro-ultraID | N | ultraID | Puromycin | <https://www.addgene.org/207693/> |
| qTAG-N-Puro-dTAG | N | dTAG | Puromycin | <https://www.addgene.org/207694/> |
| qTAG-N-Puro-3xFLAG | N | 3xFLAG | Puromycin | <https://www.addgene.org/207695/> |
| qTAG-N-Puro-3xHA | N | 3xHA | Puromycin | <https://www.addgene.org/207696/> |
| qTAG-N-Puro-V5 | N | V5 | Puromycin | <https://www.addgene.org/207697/> |
| qTAG-N-Zeo-mNeon | N | mNeonGreen | Zeocin | <https://www.addgene.org/207698/> |
| qTAG-N-Zeo-moxGFP | N | moxGFP | Zeocin | <https://www.addgene.org/207699/> |
| qTAG-N-Zeo-mScarlet | N | mScarlet | Zeocin | <https://www.addgene.org/207700/> |
| qTAG-N-Zeo-sTagRFP | N | super-TagRFP | Zeocin | <https://www.addgene.org/207701/> |
| qTAG-N-Zeo-miRFP670nano3 | N | miRFP670nano3 | Zeocin | <https://www.addgene.org/207702/> |
| qTAG-N-Zeo-miniTurbo | N | miniTurbo | Zeocin | <https://www.addgene.org/207703/> |
| qTAG-N-Zeo-ultraID | N | ultraID | Zeocin | <https://www.addgene.org/207704/> |
| qTAG-N-Zeo-dTAG | N | dTAG | Zeocin | <https://www.addgene.org/207705/> |
| qTAG-N-Zeo-3xFLAG | N | 3xFLAG | Zeocin | <https://www.addgene.org/207706/> |
| qTAG-N-Zeo-3xHA | N | 3xHA | Zeocin | <https://www.addgene.org/207707/> |
| qTAG-N-Zeo-V5 | N | V5 | Zeocin | <https://www.addgene.org/207708/> |
| qTAG-C-mNeon-Blast | C | mNeonGreen | Blasticidin | <https://www.addgene.org/207709/> |
| qTAG-C-moxGFP-Blast | C | moxGFP | Blasticidin | <https://www.addgene.org/207710/> |
| qTAG-C-mScarlet-Blast | C | mScarlet | Blasticidin | <https://www.addgene.org/207711/> |
| qTAG-C-sTagRFP-Blast | C | super-TagRFP | Blasticidin | <https://www.addgene.org/207712/> |
| qTAG-C-miRFP670nano3-Blast | C | miRFP670nano3 | Blasticidin | <https://www.addgene.org/207713/> |
| qTAG-C-miniTurbo-Blast | C | miniTurbo | Blasticidin | <https://www.addgene.org/207714/> |
| qTAG-C-ultraID-Blast | C | ultraID | Blasticidin | <https://www.addgene.org/207715/> |
| qTAG-C-dTAG-Blast | C | dTAG | Blasticidin | <https://www.addgene.org/207716/> |
| qTAG-C-3xFLAG-Blast | C | 3xFLAG | Blasticidin | <https://www.addgene.org/207717/> |
| qTAG-C-3xHA-Blast | C | 3xHA | Blasticidin | <https://www.addgene.org/207718/> |
| qTAG-C-V5-Blast | C | V5 | Blasticidin | <https://www.addgene.org/207719/> |
| qTAG-C-mNeon-Puro | C | mNeonGreen | Puromycin | <https://www.addgene.org/207720/> |
| qTAG-C-moxGFP-Puro | C | moxGFP | Puromycin | <https://www.addgene.org/207721/> |
| qTAG-C-mScarlet-Puro | C | mScarlet | Puromycin | <https://www.addgene.org/207722/> |
| qTAG-C-sTagRFP-Puro | C | super-TagRFP | Puromycin | <https://www.addgene.org/207723/> |
| qTAG-C-miRFP670nano3-Puro | C | miRFP670nano3 | Puromycin | <https://www.addgene.org/207724/> |
| qTAG-C-miniTurbo-Puro | C | miniTurbo | Puromycin | <https://www.addgene.org/207725/> |
| qTAG-C-ultraID-Puro | C | ultraID | Puromycin | <https://www.addgene.org/207726/> |
| qTAG-C-dTAG-Puro | C | dTAG | Puromycin | <https://www.addgene.org/207727/> |
| qTAG-C-3xFLAG-Puro | C | 3xFLAG | Puromycin | <https://www.addgene.org/207728/> |
| qTAG-C-3xHA-Puro | C | 3xHA | Puromycin | <https://www.addgene.org/207729/> |
| qTAG-C-V5-Puro | C | V5 | Puromycin | <https://www.addgene.org/207730/> |
| qTAG-C-mNeon-Zeo | C | mNeonGreen | Zeocin | <https://www.addgene.org/207731/> |
| qTAG-C-moxGFP-Zeo | C | moxGFP | Zeocin | <https://www.addgene.org/207732/> |
| qTAG-C-mScarlet-Zeo | C | mScarlet | Zeocin | <https://www.addgene.org/207733/> |
| qTAG-C-sTagRFP-Zeo | C | super-TagRFP | Zeocin | <https://www.addgene.org/207734/> |
| qTAG-C-miRFP670nano3-Zeo | C | miRFP670nano3 | Zeocin | <https://www.addgene.org/207735/> |
| qTAG-C-miniTurbo-Zeo | C | miniTurbo | Zeocin | <https://www.addgene.org/207736/> |
| qTAG-C-ultraID-Zeo | C | ultraID | Zeocin | <https://www.addgene.org/207737/> |
| qTAG-C-dTAG-Zeo | C | dTAG | Zeocin | <https://www.addgene.org/207738/> |
| qTAG-C-3xFLAG-Zeo | C | 3xFLAG | Zeocin | <https://www.addgene.org/207739/> |
| qTAG-C-3xHA-Zeo | C | 3xHA | Zeocin | <https://www.addgene.org/207740/> |
| qTAG-C-V5-Zeo | C | V5 | Zeocin | <https://www.addgene.org/207741/> |
| pEF1α-Cre-PGK-Puro | - | Cre Recombinase | Puromycin | <https://www.addgene.org/207742/> |
| pEF1α-Cre-2A-Puro | - | Cre Recombinase | Puromycin | <https://www.addgene.org/207743/> |
| pEF1α-Cre-2A-moxGFP | - | Cre Recombinase | moxGFP | <https://www.addgene.org/207744/> |
| pEF1α-Cre-2A-mScarlet | - | Cre Recombinase | mScarlet | <https://www.addgene.org/207745/> |
| pEF1α-Cre-2A-mTagBFP2 | - | Cre Recombinase | mTagBFP2 | <https://www.addgene.org/207746/> |
| pEF1α-Cre-2A-miRFP670nano3 | - | Cre Recombinase | miRFP670nano3 | <https://www.addgene.org/207747/> |
| pX330 | - | Cas9 + sgRNA | - | <https://www.addgene.org/42230/> |
| pX330-PITCh | - | Cas9 + sgRNA + PITCh sgRNA | - | <https://www.addgene.org/127875/> |
| pX330-PITCh-ACTB | - | Cas9 + sgRNA + PITCh sgRNA | - | <https://www.addgene.org/207748/> |
| qTAG-N-Solo-mNeon-ACTB | N | mNeonGreen | - | <https://www.addgene.org/207749/> |
| qTAG-N-Solo-mScarlet-ACTB | N | mScarlet | - | <https://www.addgene.org/207750/> |
| qTAG-N-Blast-mNeon-ACTB | N | mNeonGreen | Blasticidin | <https://www.addgene.org/207751/> |
| qTAG-N-Puro-mNeon-ACTB | N | mNeonGreen | Puromycin | <https://www.addgene.org/207752/> |
| qTAG-N-Blast-mScarlet-ACTB | N | mScarlet | Blasticidin | <https://www.addgene.org/207753/> |
| qTAG-N-Puro-mScarlet-ACTB | N | mScarlet | Puromycin | <https://www.addgene.org/207754/> |
| pX330-PITCh-H2BC11 | - | Cas9 + sgRNA + PITCh sgRNA | - | <https://www.addgene.org/207755/> |
| qTAG-C-Solo-mScarlet-H2BC11 | C | mScarlet | - | <https://www.addgene.org/207756/> |
| qTAG-C-moxGFP-Blast-H2BC11 | C | moxGFP | Blasticidin | <https://www.addgene.org/207757/> |
| qTAG-C-moxGFP-Puro-H2BC11 | C | moxGFP | Puromycin | <https://www.addgene.org/207758/> |
| qTAG-C-moxGFP-Zeo-H2BC11 | C | moxGFP | Zeocin | <https://www.addgene.org/207759/> |
| qTAG-C-moxGFP-Puro-H2BC11-MMEJ | C | moxGFP | Puromycin | <https://www.addgene.org/207760/> |
| qTAG-C-mScarlet-Puro-H2BC11 | C | mScarlet | Puromycin | <https://www.addgene.org/207761/> |
| qTAG-C-miRFP670nano3-Blast-H2BC11 | C | miRFP670nano3 | Blasticidin | <https://www.addgene.org/207762/> |
| pX330-PITCh-TUBA1B | - | Cas9 + sgRNA + PITCh sgRNA | - | <https://www.addgene.org/207763/> |
| qTAG-N-Solo-mScarlet-TUBA1B | N | mScarlet | - | <https://www.addgene.org/207764/> |
| qTAG-N-Solo-sTagRFP-TUBA1B | N | super-TagRFP | - | <https://www.addgene.org/207765/> |
| qTAG-N-Blast-moxGFP-TUBA1B | N | moxGFP | Blasticidin | <https://www.addgene.org/207766/> |
| qTAG-N-Puro-moxGFP-TUBA1B | N | moxGFP | Puromycin | <https://www.addgene.org/207767/> |
| qTAG-N-Blast-sTagRFP-TUBA1B | N | super-TagRFP | Blasticidin | <https://www.addgene.org/207768/> |
| qTAG-N-Puro-sTagRFP-TUBA1B | N | super-TagRFP | Puromycin | <https://www.addgene.org/207769/> |
| pX330-PITCh-LMNB1 | - | Cas9 + sgRNA + PITCh sgRNA | - | <https://www.addgene.org/207770/> |
| qTAG-N-Solo-mScarlet-LMNB1 | N | mScarlet | - | <https://www.addgene.org/207771/> |
| qTAG-N-Blast-moxGFP-LMNB1 | N | moxGFP | Blasticidin | <https://www.addgene.org/207772/> |
| qTAG-N-Puro-moxGFP-LMNB1 | N | moxGFP | Puromycin | <https://www.addgene.org/207773/> |
| qTAG-N-Blast-miniTurbo-LMNB1 | N | miniTurbo | Blasticidin | <https://www.addgene.org/207774/> |
| qTAG-N-Blast-ultraID-LMNB1 | N | ultraID | Blasticidin | <https://www.addgene.org/207775/> |
| qTAG-N-Blast-dTAG-LMNB1 | N | dTAG | Blasticidin | <https://www.addgene.org/207776/> |
| qTAG-N-Blast-3xFLAG-LMNB1 | N | 3xFLAG | Blasticidin | <https://www.addgene.org/207777/> |
| qTAG-N-Blast-3xHA-LMNB1 | N | 3xHA | Blasticidin | <https://www.addgene.org/207778/> |
| qTAG-N-Blast-V5-LMNB1 | N | V5 | Blasticidin | <https://www.addgene.org/207779/> |
| pX330-PITCh-H3C2 | - | Cas9 + sgRNA + PITCh sgRNA | - | <https://www.addgene.org/207780/> |
| qTAG-C-Solo-mScarlet-H3C2 | C | mScarlet | - | <https://www.addgene.org/207781/> |
| qTAG-C-mNeon-Blast-H3C2 | C | mNeonGreen | Blasticidin | <https://www.addgene.org/207782/> |
| qTAG-C-moxGFP-Puro-H3C2 | C | moxGFP | Puromycin | <https://www.addgene.org/207783/> |
| qTAG-C-miRFP670nano3-Blast-H3C2 | C | miRFP670nano3 | Blasticidin | <https://www.addgene.org/207784/> |
| pX330-PITCh-TUBB4B | - | Cas9 + sgRNA + PITCh sgRNA | - | <https://www.addgene.org/207785/> |
| qTAG-C-mNeon-Blast-TUBB4B | C | mNeonGreen | Blasticidin | <https://www.addgene.org/207786/> |
| pX330-LAMP1 | - | Cas9 + sgRNA | - | <https://www.addgene.org/207787/> |
| qTAG-C-moxGFP-Puro-LAMP1 | C | moxGFP | Puromycin | <https://www.addgene.org/207788/> |
| pX330-PITCh-TOMM20 | - | Cas9 + sgRNA + PITCh sgRNA | - | <https://www.addgene.org/207789/> |
| qTAG-C-mNeon-Puro-TOMM20 | C | mNeonGreen | Puromycin | <https://www.addgene.org/207790/> |
| pX330-PITCh-GOLGA2 | - | Cas9 + sgRNA + PITCh sgRNA | - | <https://www.addgene.org/207791/> |
| qTAG-N-Puro-moxGFP-GOLGA2 | N | moxGFP | Puromycin | <https://www.addgene.org/207792/> |
| pX330-PITCh-MAPRE1 | - | Cas9 + sgRNA + PITCh sgRNA | - | <https://www.addgene.org/207793/> |
| qTAG-C-moxGFP-Puro-MAPRE1 | C | moxGFP | Puromycin | <https://www.addgene.org/207794/> |
| qTAG-N-Puro-mStayGold | N | mStayGold | Puromycin | <https://www.addgene.org/227250/> |
| qTAG-N-Blast-mStayGold | N | mStayGold | Blasticidin | <https://www.addgene.org/227251/> |
| qTAG-N-Zeo-mStayGold | N | mStayGold | Zeocin | <https://www.addgene.org/227252/> |
| qTAG-C-mStayGold-Puro | C | mStayGold | Puromycin | <https://www.addgene.org/227253/> |
| qTAG-C-mStayGold-Blast | C | mStayGold | Blasticidin | <https://www.addgene.org/227254/> |
| qTAG-C-mStayGold-Zeo | C | mStayGold | Zeocin | <https://www.addgene.org/227255/> |
| qTAG-C-mStayGold-EFS-Puro | C | mStayGold | Puromycin | <https://www.addgene.org/227256/> |
| qTAG-C-mStayGold-EFS-Blast | C | mStayGold | Blasticidin | <https://www.addgene.org/227257/> |
| qTAG-C-mStayGold-EFS-Zeo | C | mStayGold | Zeocin | <https://www.addgene.org/227258/> |
| qTAG-C-mScarlet-EFS-Puro | C | mScarlet | Puromycin | <https://www.addgene.org/227259/> |
| qTAG-C-mScarlet-EFS-Blast | C | mScarlet | Blasticidin | <https://www.addgene.org/227260/> |
| qTAG-C-mScarlet-EFS-Zeo | C | mScarlet | Zeocin | <https://www.addgene.org/227261/> |
| qTAG-KO-Puro | N | - | Puromycin | <https://www.addgene.org/227262/> |
| qTAG-KO-Blast | N | - | Blasticidin | <https://www.addgene.org/227263/> |
| qTAG-KO-PGK-Puro | N | - | Puromycin | <https://www.addgene.org/227264/> |
| qTAG-KO-PGK-Blast | N | - | Blasticidin | <https://www.addgene.org/227265/> |
| qTAG-AAVS1-Ef1a-Puro | - | - | Puromycin | <https://www.addgene.org/227266/> |
| qTAG-AAVS1-Ef1a-Blast | - | - | Blasticidin | <https://www.addgene.org/227267/> |
| qTAG-AAVS1-Ef1a-Zeo | - | - | Zeocin | <https://www.addgene.org/227268/> |
| qTAG-AAVS1-PGK-Puro | - | - | Puromycin | <https://www.addgene.org/227269/> |
| qTAG-AAVS1-PGK-Blast | - | - | Blasticidin | <https://www.addgene.org/227270/> |
| qTAG-AAVS1-PGK-Zeo | - | - | Zeocin | <https://www.addgene.org/227271/> |
| pX330-AAVS1 | - | - | - | <https://www.addgene.org/227272/> |
| qTAG-AAVS1-Ef1a-Puro-moxGFP | - | moxGFP | Puromycin | <https://www.addgene.org/227273/> |
| qTAG-AAVS1-Ef1a-Puro-mScarlet | - | mScarlet | Puromycin | <https://www.addgene.org/227274/> |
| qTAG-AAVS1-PGK-Puro-moxGFP | - | moxGFP | Puromycin | <https://www.addgene.org/227275/> |
| pX330-PITCh-ARL13B | - | Cas9 + sgRNA + PITCh sgRNA | - | <https://www.addgene.org/227276/> |
| qTAG-C-Solo-mStayGold-ARL13B | C | mStayGold | - | <https://www.addgene.org/227277/> |
| qTAG-C-mStayGold-EFS-Blast-ARL13B | C | mStayGold | Blasticidin | <https://www.addgene.org/227278/> |
| pX330-CANX | - | Cas9 + sgRNA | - | <https://www.addgene.org/227279/> |
| qTAG-C-Solo-mStayGold-CANX | C | mStayGold | - | <https://www.addgene.org/227280/> |
| qTAG-C-mStayGold-Puro-CANX | C | mStayGold | Puromycin | <https://www.addgene.org/227281/> |
| pX330-CENPA | - | Cas9 + sgRNA | - | <https://www.addgene.org/227282/> |
| qTAG-N-Solo-mStayGold-CENPA | N | mStayGold | - | <https://www.addgene.org/227283/> |
| pX330-PCNT | - | Cas9 + sgRNA | - | <https://www.addgene.org/227284/> |
| qTAG-N-Solo-mStayGold-PCNT | N | mStayGold | - | <https://www.addgene.org/227285/> |
| pX330-CEP135 | - | Cas9 + sgRNA | - | <https://www.addgene.org/227286/> |
| qTAG-N-Solo-mStayGold-CEP135 | N | mStayGold | - | <https://www.addgene.org/227287/> |
| pX330-PITCh-CEP192 | - | Cas9 + sgRNA + PITCh sgRNA | - | <https://www.addgene.org/227288/> |
| qTAG-C-Solo-mStayGold-CEP192 | C | mStayGold | - | <https://www.addgene.org/227289/> |
| qTAG-C-mStayGold-EFS-Puro-CEP192 | C | mStayGold | Puromycin | <https://www.addgene.org/227290/> |
| pX330-CETN2 | - | Cas9 + sgRNA | - | <https://www.addgene.org/227291/> |
| qTAG-N-Solo-mStayGold-CETN2 | N | mStayGold | - | <https://www.addgene.org/227292/> |
| pX330-EZR | - | Cas9 + sgRNA | - | <https://www.addgene.org/227293/> |
| qTAG-C-Solo-mStayGold-EZR | C | mStayGold | Puromycin | <https://www.addgene.org/227294/> |
| pX330-PITCh-MAP4 | - | Cas9 + sgRNA + PITCh sgRNA | - | <https://www.addgene.org/227295/> |
| qTAG-N-Solo-mStayGold-MAP4 | N | mStayGold | - | <https://www.addgene.org/227296/> |
| pX330-RAB7A | - | Cas9 + sgRNA | - | <https://www.addgene.org/227297/> |
| qTAG-N-Solo-mStayGold-RAB7A | N | mStayGold | - | <https://www.addgene.org/227298/> |
| pX330-TJP1 | - | Cas9 + sgRNA | - | <https://www.addgene.org/227299/> |
| qTAG-N-Solo-mStayGold-TJP1 | N | mStayGold | - | <https://www.addgene.org/227300/> |
| pX330-PITCh-VIM | - | Cas9 + sgRNA + PITCh sgRNA | - | <https://www.addgene.org/227301/> |
| qTAG-C-Solo-mStayGold-VIM | C | mStayGold | - | <https://www.addgene.org/227302/> |
| pX330-PITCh-PEX3 | - | Cas9 + sgRNA + PITCh sgRNA | - | <https://www.addgene.org/227303/> |
| qTAG-C-Solo-mStayGold-PEX3 | C | mStayGold | - | <https://www.addgene.org/227304/> |
| pX330-PITCh-MYO1C | - | Cas9 + sgRNA + PITCh sgRNA | - | <https://www.addgene.org/227305/> |
| qTAG-N-Solo-mStayGold-MYO1C | N | mScarlet | - | <https://www.addgene.org/227306/> |
| qTAG-C-Solo-mStayGold-TOMM20 | C | mStayGold | - | <https://www.addgene.org/227307/> |
| pX330-PITCh-COX8A | - | Cas9 + sgRNA + PITCh sgRNA | - | <https://www.addgene.org/227308/> |
| qTAG-C-Solo-mStayGold-COX8A | C | mStayGold | - | <https://www.addgene.org/227309/> |
| pX458-PLK4 | - | Cas9 + sgRNA | - | <https://www.addgene.org/227310/> |
| qTAG-C-mStayGold-EFS-Puro-PLK4 | C | mStayGold | Puromycin | <https://www.addgene.org/227311/> |
| pX330-PITCh-CLTC | - | Cas9 + sgRNA + PITCh sgRNA | - | <https://www.addgene.org/227312/> |
| qTAG-C-Solo-mStayGold-CLTC | C | mStayGold | - | <https://www.addgene.org/227313/> |
| qTAG-C-mStayGold-Puro-CLTC | C | mStayGold | Puromycin | <https://www.addgene.org/227314/> |
| pX330-PITCh-PXN | - | Cas9 + sgRNA + PITCh sgRNA | - | <https://www.addgene.org/227315/> |
| qTAG-C-Solo-mStayGold-PXN | C | mStayGold | - | <https://www.addgene.org/227316/> |
| qTAG-C-mStayGold-Puro-PXN | C | mStayGold | Puromycin | <https://www.addgene.org/227317/> |
| pX330-TP53 | - | Cas9 + sgRNA | - | <https://www.addgene.org/227318/> |
| qTAG-KO-Puro-TP53 | N | - | Puromycin | <https://www.addgene.org/227319/> |
| qTAG-N-Puro-mStayGold-ACTB | N | mStayGold | Puromycin | <https://www.addgene.org/227320/> |
| qTAG-N-Puro-mStayGold-TUBA1B | N | mStayGold | Puromycin | <https://www.addgene.org/227321/> |
| qTAG-C-Solo-mStayGold-LAMP1 | C | mStayGold | - | <https://www.addgene.org/227322/> |
| qTAG-C-Solo-mScarlet-MAPRE1 | C | mScarlet | - | <https://www.addgene.org/227323/> |
| qTAG-C-Solo-mStayGold-MAPRE1 | C | mStayGold | - | <https://www.addgene.org/227324/> |
| qTAG-N-Solo-mStayGold-GOLGA2 | N | mStayGold | - | <https://www.addgene.org/227325/> |
| qTAG-N-Solo-mStayGold-ACTB | N | mStayGold | - | <https://www.addgene.org/227326/> |
| qTAG-N-Solo-mStayGold-TUBA1B | N | mStayGold | - | <https://www.addgene.org/227327/> |
| qTAG-N-Solo-mStayGold-LMNB1 | N | mStayGold | - | <https://www.addgene.org/227328/> |
| qTAG-N-Puro-mStayGold-LMNB1 | N | mStayGold | Puromycin | <https://www.addgene.org/227329/> |
| qTAG-C-Solo-mStayGold-H2BC11 | C | mStayGold | - | <https://www.addgene.org/227330/> |
| qTAG-C-Solo-miRFP670nano3-H2BC11 | C | miRFP670nano3 | - | <https://www.addgene.org/227331/> |
| qTAG-C-mStayGold-Puro-H2BC11 | C | mStayGold | Puromycin | <https://www.addgene.org/227332/> |
| qTAG-C-mStayGold-Puro-H2BC11-MMEJ | C | mStayGold | Puromycin | <https://www.addgene.org/227333/> |
| qTAG-C-Solo-mStayGold-H3C2 | C | mStayGold | - | <https://www.addgene.org/227334/> |
| qTAG-C-Solo-miRFP670nano3-H3C2 | C | miRFP670nano3 | - | <https://www.addgene.org/227335/> |
