# Supplementary material for: qTAG: an adaptable plasmid scaffold for CRISPR-based endogenous tagging
Source: EMBO J. 2024 Dec 12;44(3):947–74. doi: 10.1038/s44318-024-00337-5 (PMC11790981; doi:10.1038/s44318-024-00337-5)
Supplement: Supplementary file 6 — Dataset EV1 [file 44318_2024_337_MOESM6_ESM.zip › Dataset EV1/README.docx]

**Dataset EV1. A collection of files highlighting the design, cloning sequences, primer locations, and final plasmid products using qTAG cassettes targeting N-terminus or C-terminus insertions. (a)** The provided sequence files are fully annotated and highlight the design of a C-terminal insertion of a qTAG-moxGFP-Puro cassette into the H2BC11 gene. They include a genomic loci design file, HDR and MMEJ homology arm designs, along with the final cloned CRISPR and repair plasmids. **(b)** The provided sequence files are fully annotated and highlight the design of a N-terminal insertion of a qTAG-Puro-Neon cassette into the ACTB gene. All of the corresponding design and final plasmid files are also included for this example.
