# Supplementary material for: qTAG: an adaptable plasmid scaffold for CRISPR-based endogenous tagging
Source: EMBO J. 2024 Dec 12;44(3):947–74. doi: 10.1038/s44318-024-00337-5 (PMC11790981; doi:10.1038/s44318-024-00337-5)
Supplement: Supplementary file 8 — Source data Fig. 2 [file 44318_2024_337_MOESM8_ESM.zip › 02_Figure_02/2E/README.docx]

Included for each image set are the following:

- TIFF images labeled as "_FULL_RANGE" are MaxIP images that retain the full 16-bit dynamic range from acquisition. To align with the appearance of the RGB TIFF images, contrast levels may require adjustment.
- RGB 8-bit processed TIFF images corresponding to the figure, with contrast thresholds applied.
