# Supplementary figures and images for: qTAG: an adaptable plasmid scaffold for CRISPR-based endogenous tagging (part 1 of 5)
Source: EMBO J. 2024 Dec 12;44(3):947–74. doi: 10.1038/s44318-024-00337-5 (PMC11790981; doi:10.1038/s44318-024-00337-5)

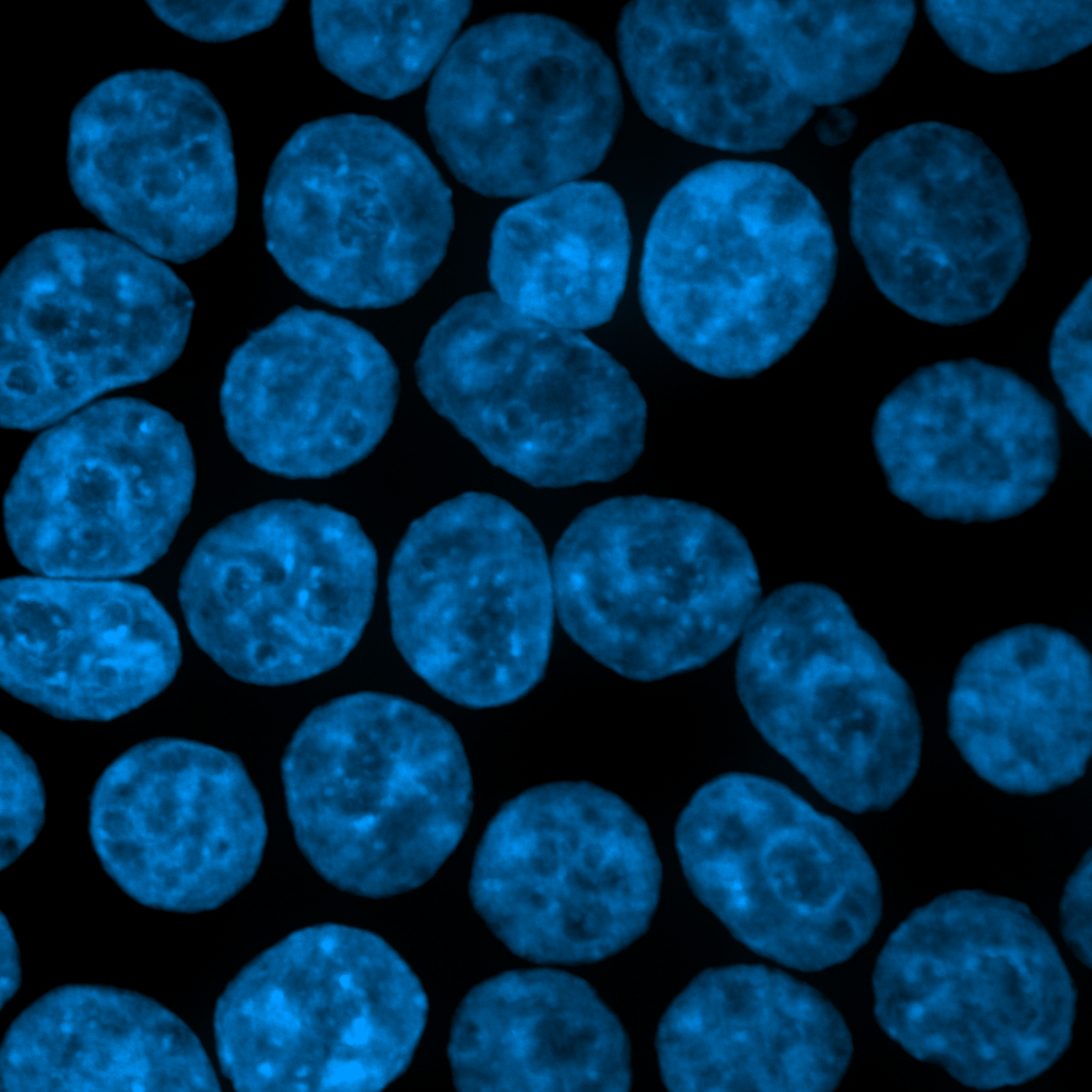

Supplement: Supplementary file 8 — Source data Fig. 2 [file 44318_2024_337_MOESM8_ESM.zip › 02_Figure_02/2E/01_WT/HEK-CTRL-DAPI.tif]

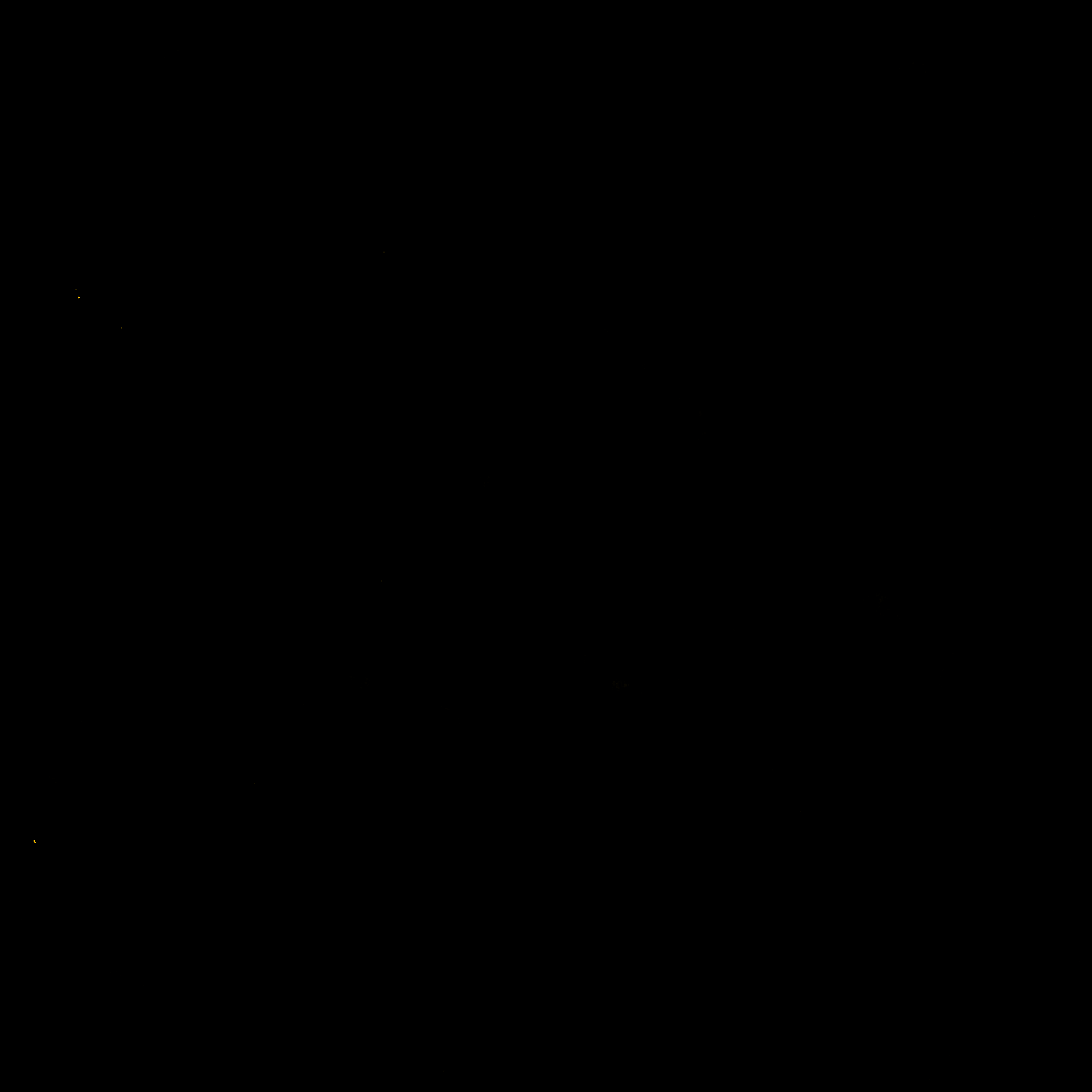

Supplement: Supplementary file 8 — Source data Fig. 2 [file 44318_2024_337_MOESM8_ESM.zip › 02_Figure_02/2E/01_WT/HEK-CTRL-GFP.tif]

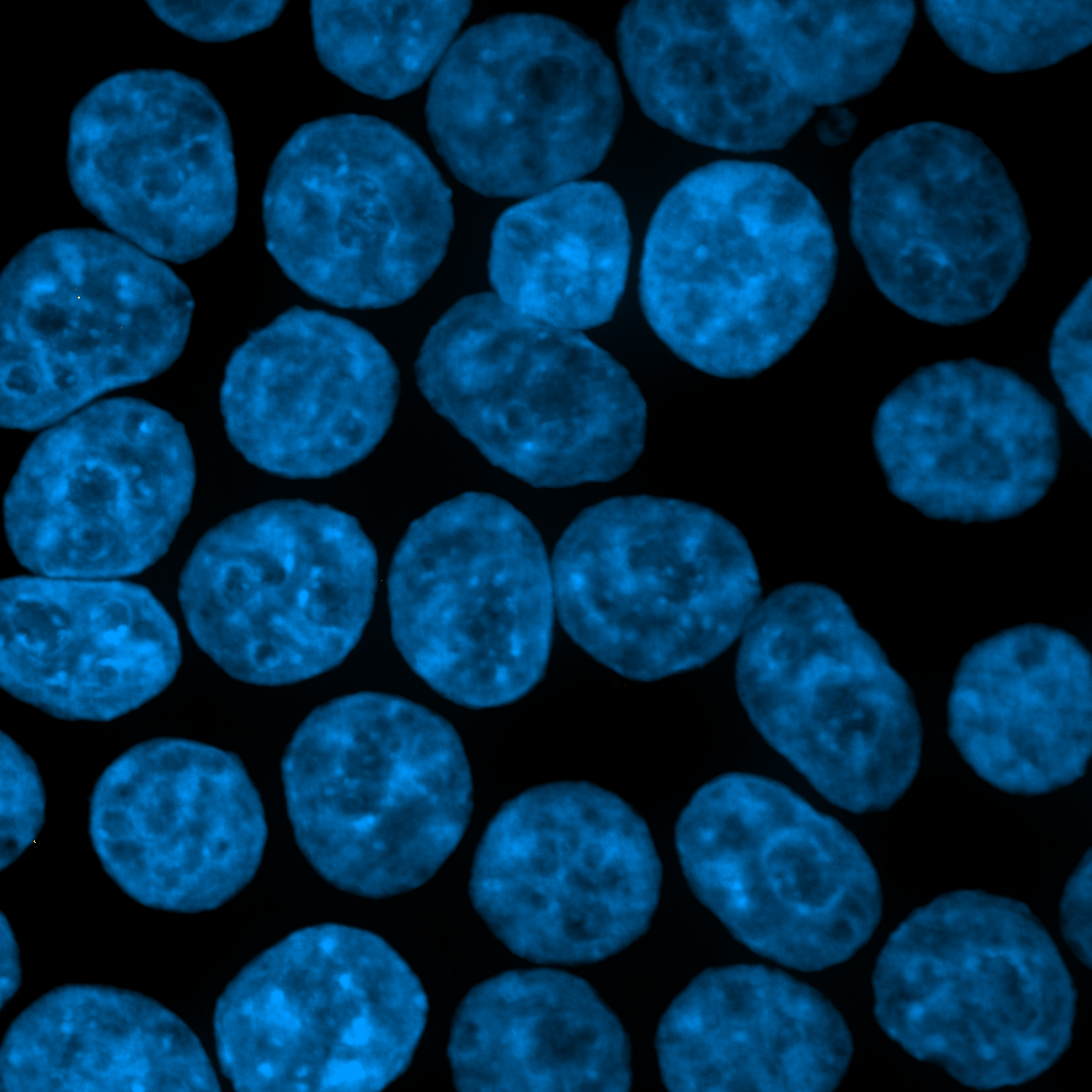

Supplement: Supplementary file 8 — Source data Fig. 2 [file 44318_2024_337_MOESM8_ESM.zip › 02_Figure_02/2E/01_WT/HEK-CTRL-Merge.tif]

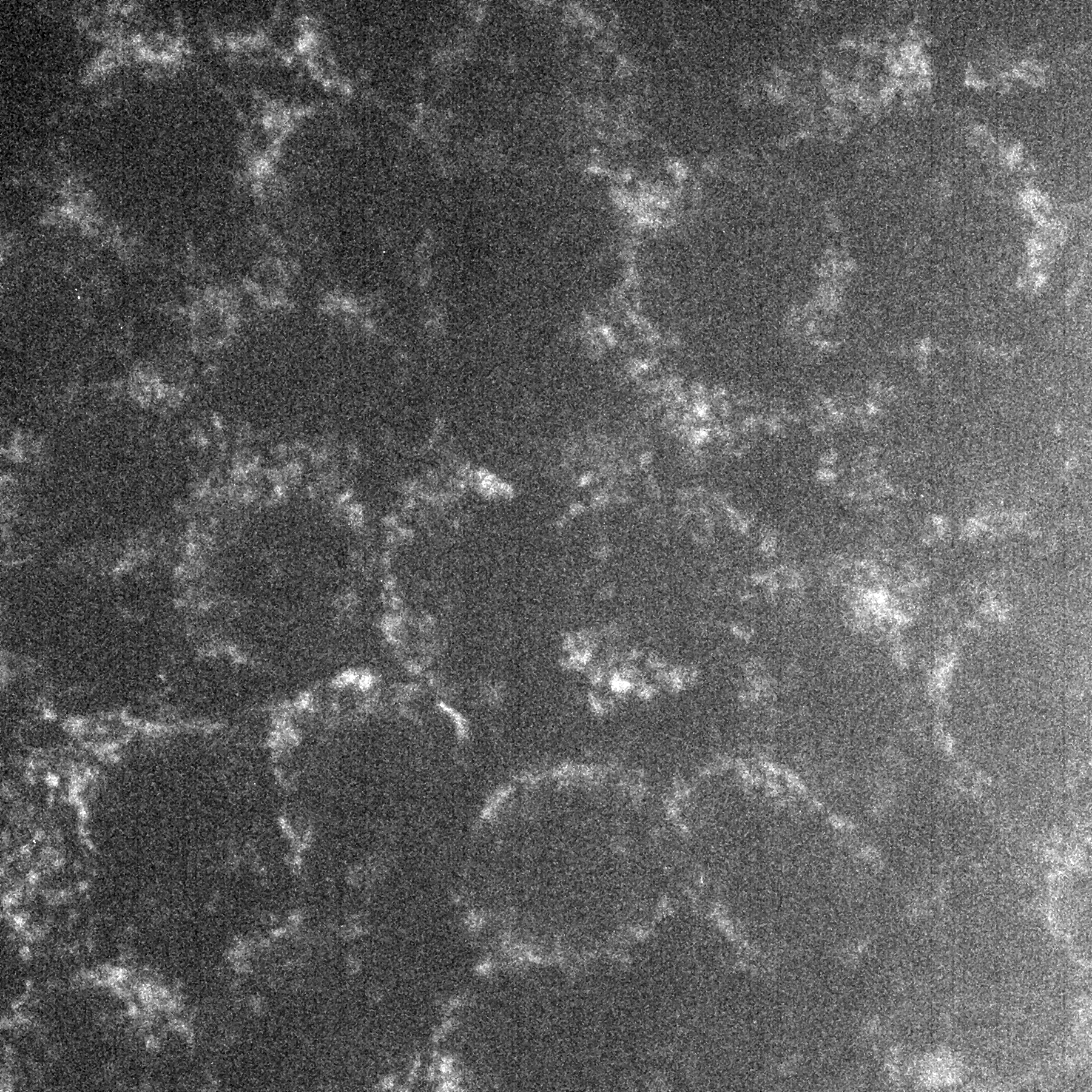

Supplement: Supplementary file 8 — Source data Fig. 2 [file 44318_2024_337_MOESM8_ESM.zip › 02_Figure_02/2E/01_WT/_FULL-RANGE-HEK-WT.tif]

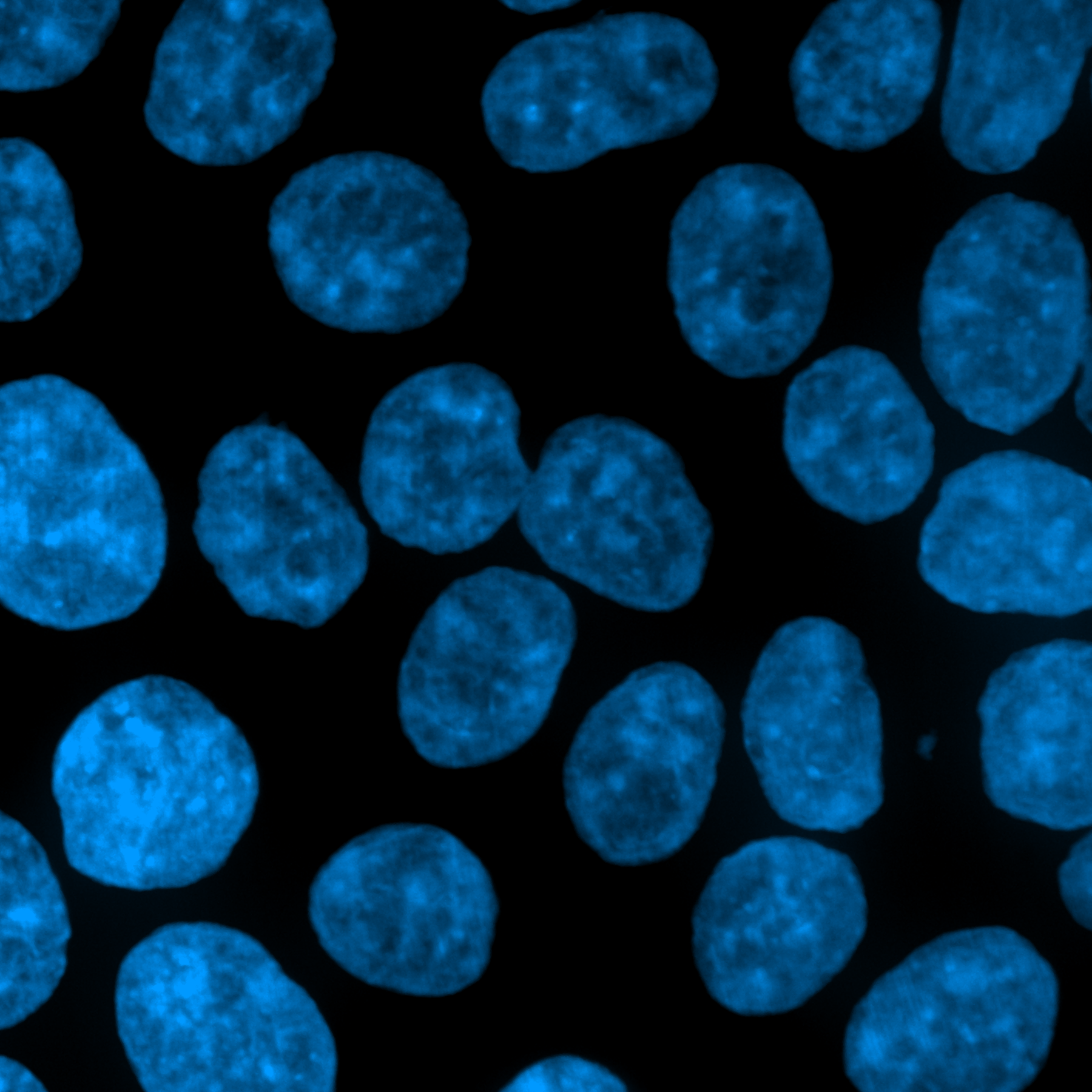

Supplement: Supplementary file 8 — Source data Fig. 2 [file 44318_2024_337_MOESM8_ESM.zip › 02_Figure_02/2E/02_HDR/HEK-HDR-DAPI.tif]

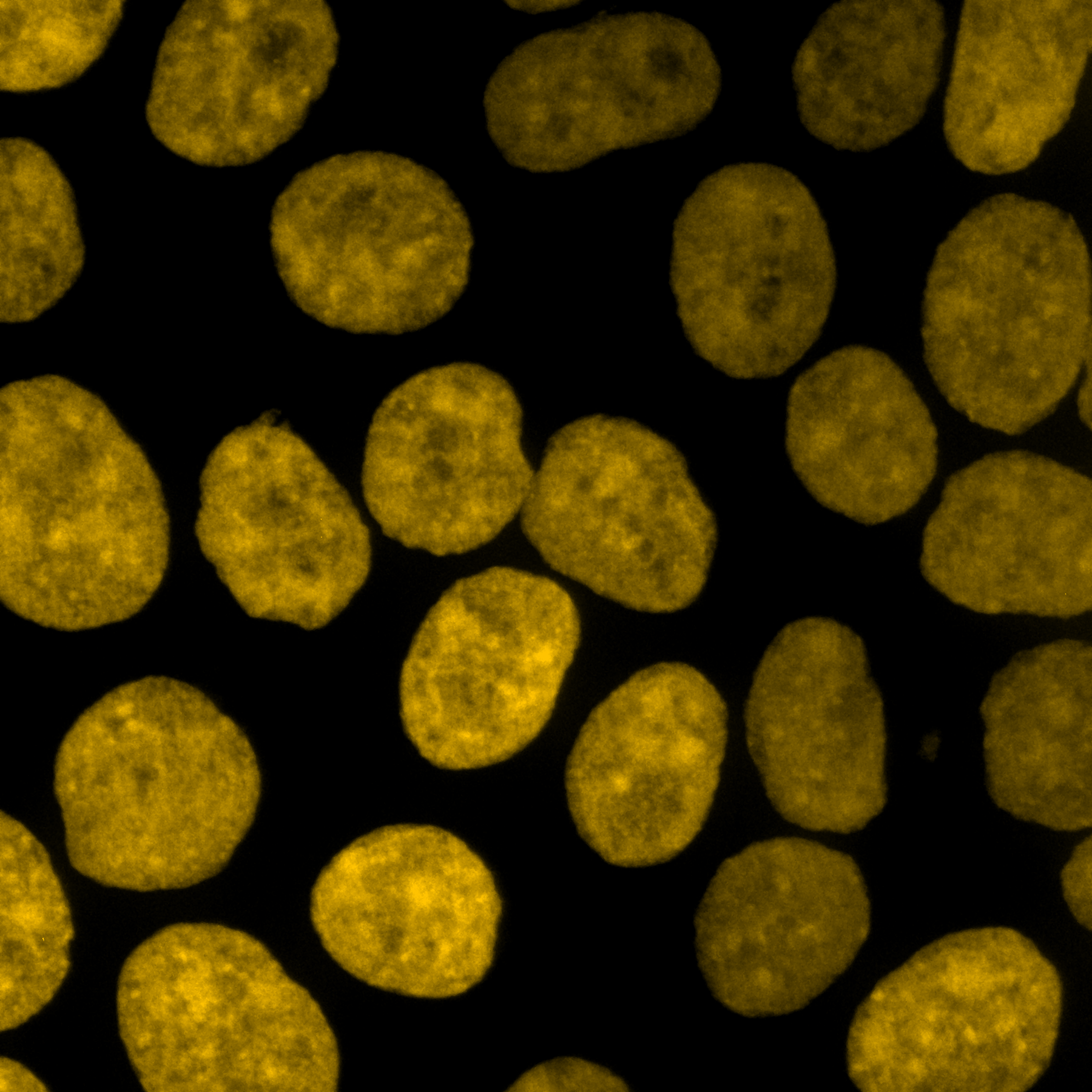

Supplement: Supplementary file 8 — Source data Fig. 2 [file 44318_2024_337_MOESM8_ESM.zip › 02_Figure_02/2E/02_HDR/HEK-HDR-GFP.tif]

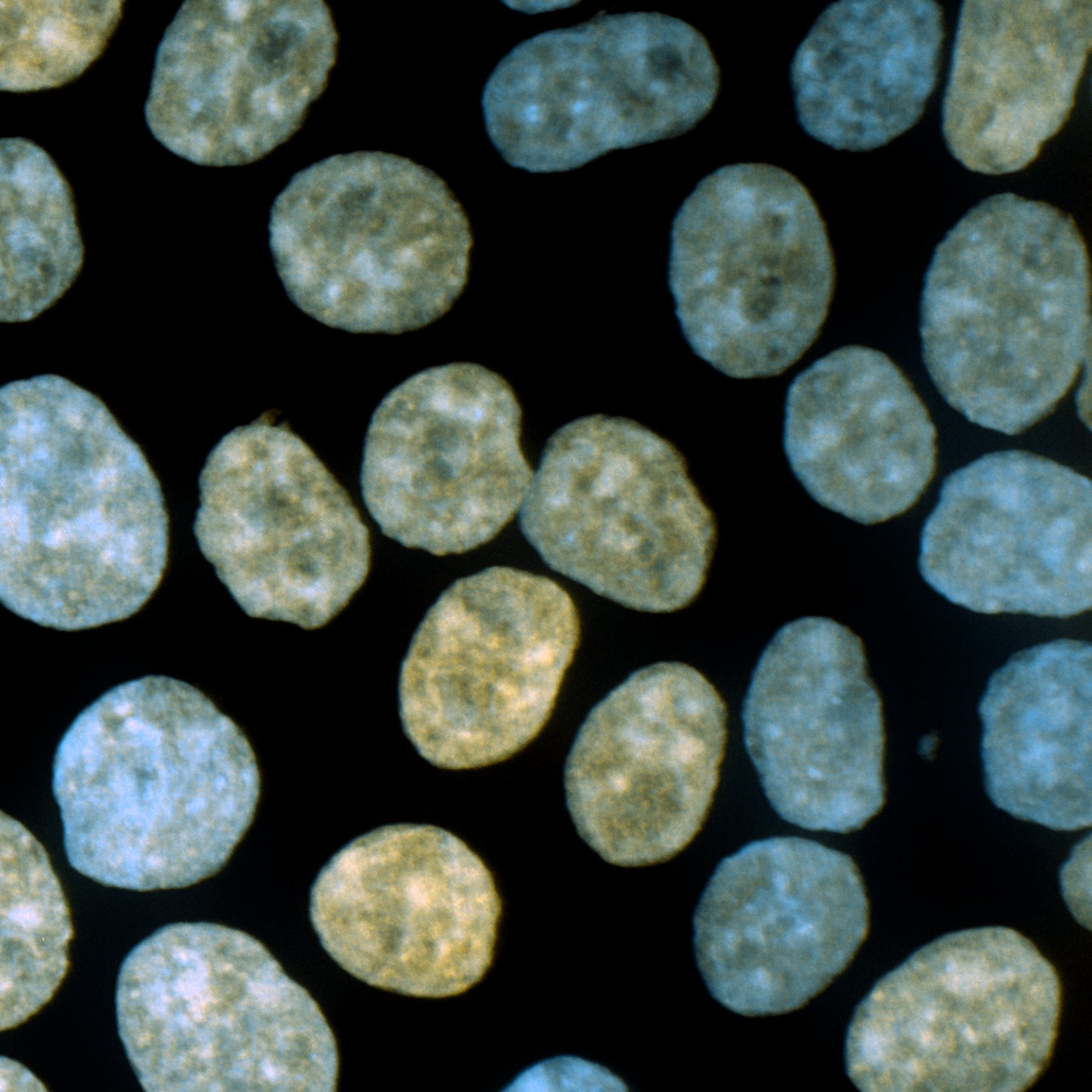

Supplement: Supplementary file 8 — Source data Fig. 2 [file 44318_2024_337_MOESM8_ESM.zip › 02_Figure_02/2E/02_HDR/HEK-HDR-Merge.tif]

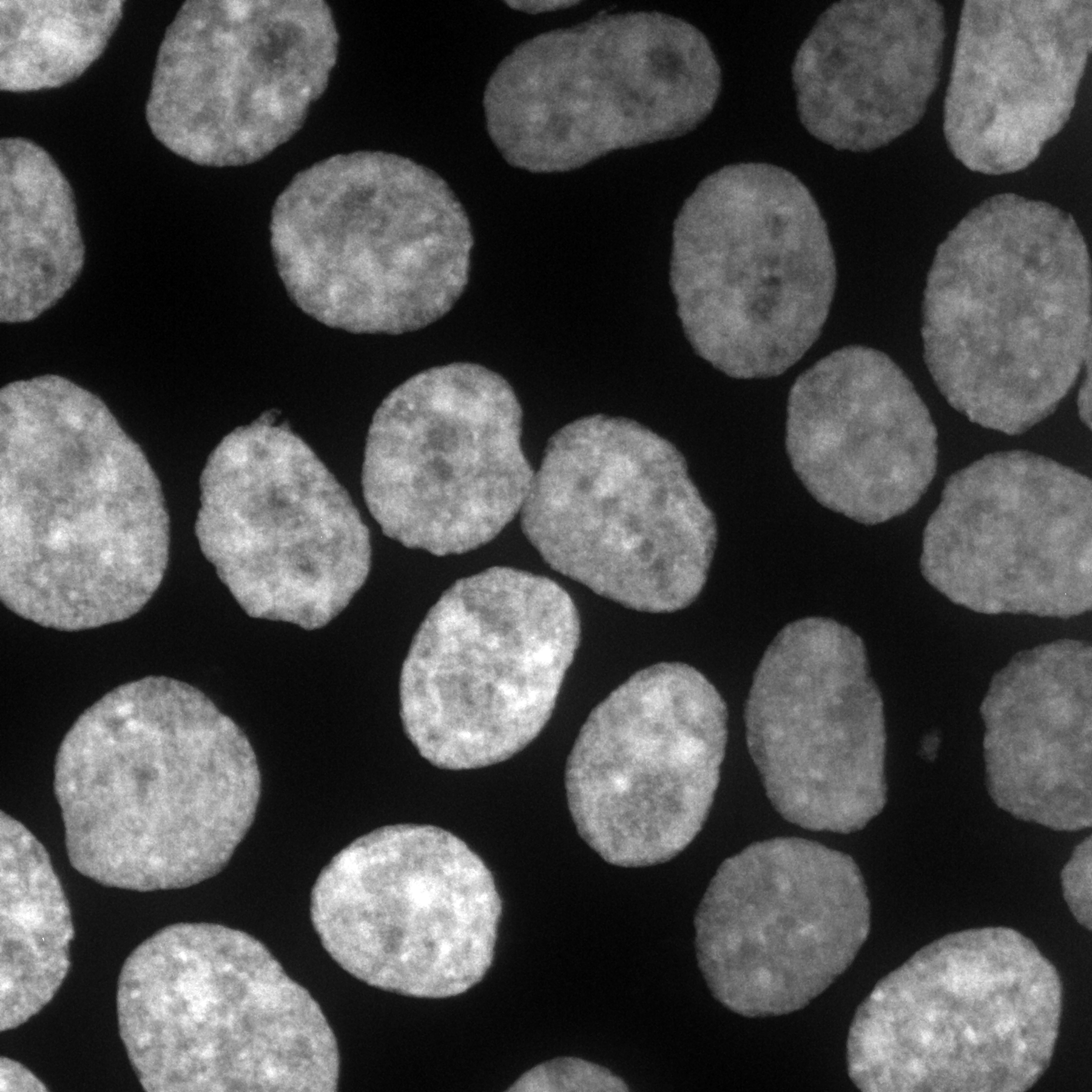

Supplement: Supplementary file 8 — Source data Fig. 2 [file 44318_2024_337_MOESM8_ESM.zip › 02_Figure_02/2E/02_HDR/_FULL-RANGE-HEK-HDR.tif]

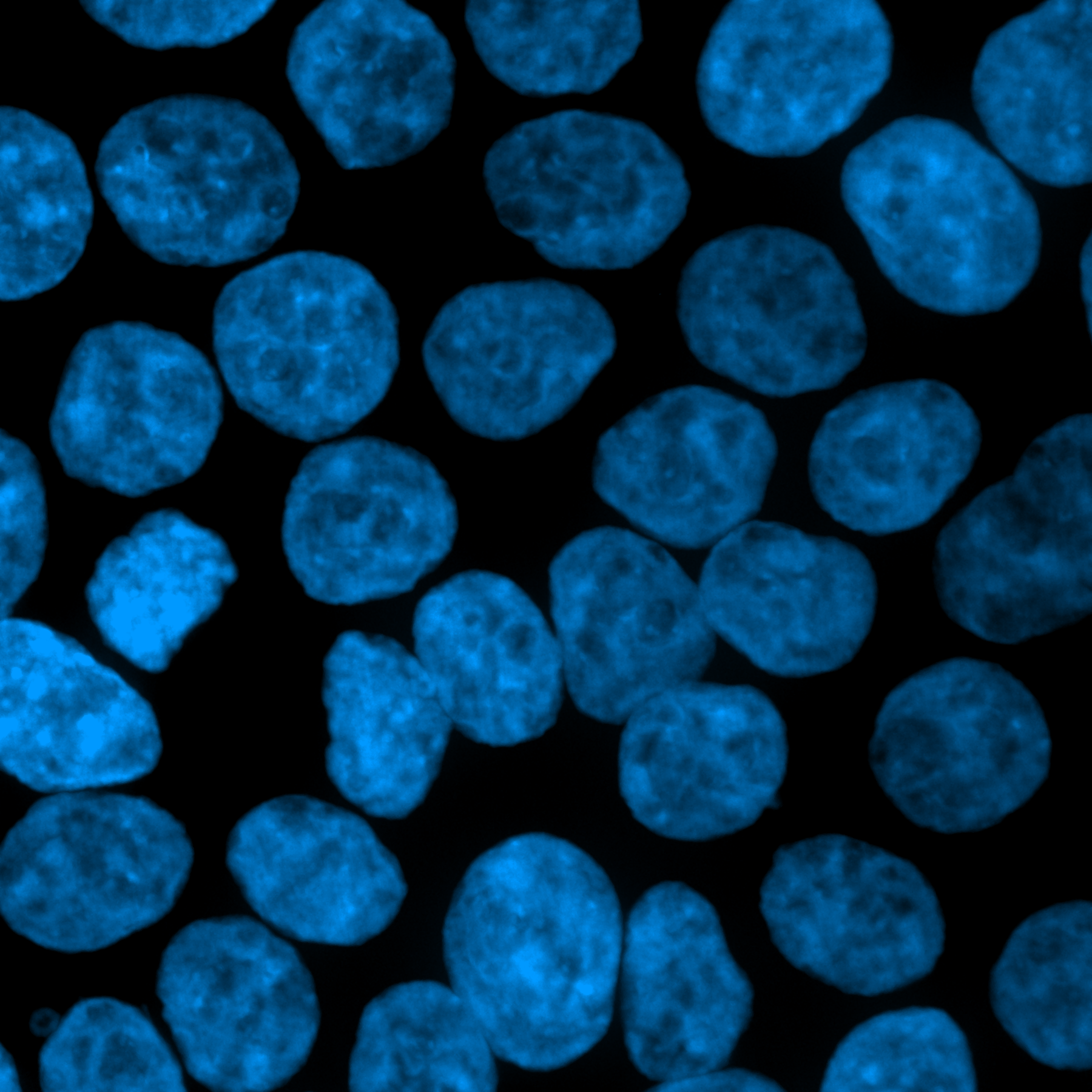

Supplement: Supplementary file 8 — Source data Fig. 2 [file 44318_2024_337_MOESM8_ESM.zip › 02_Figure_02/2E/03_MMEJ/HEK-MMEJ-DAPI.tif]

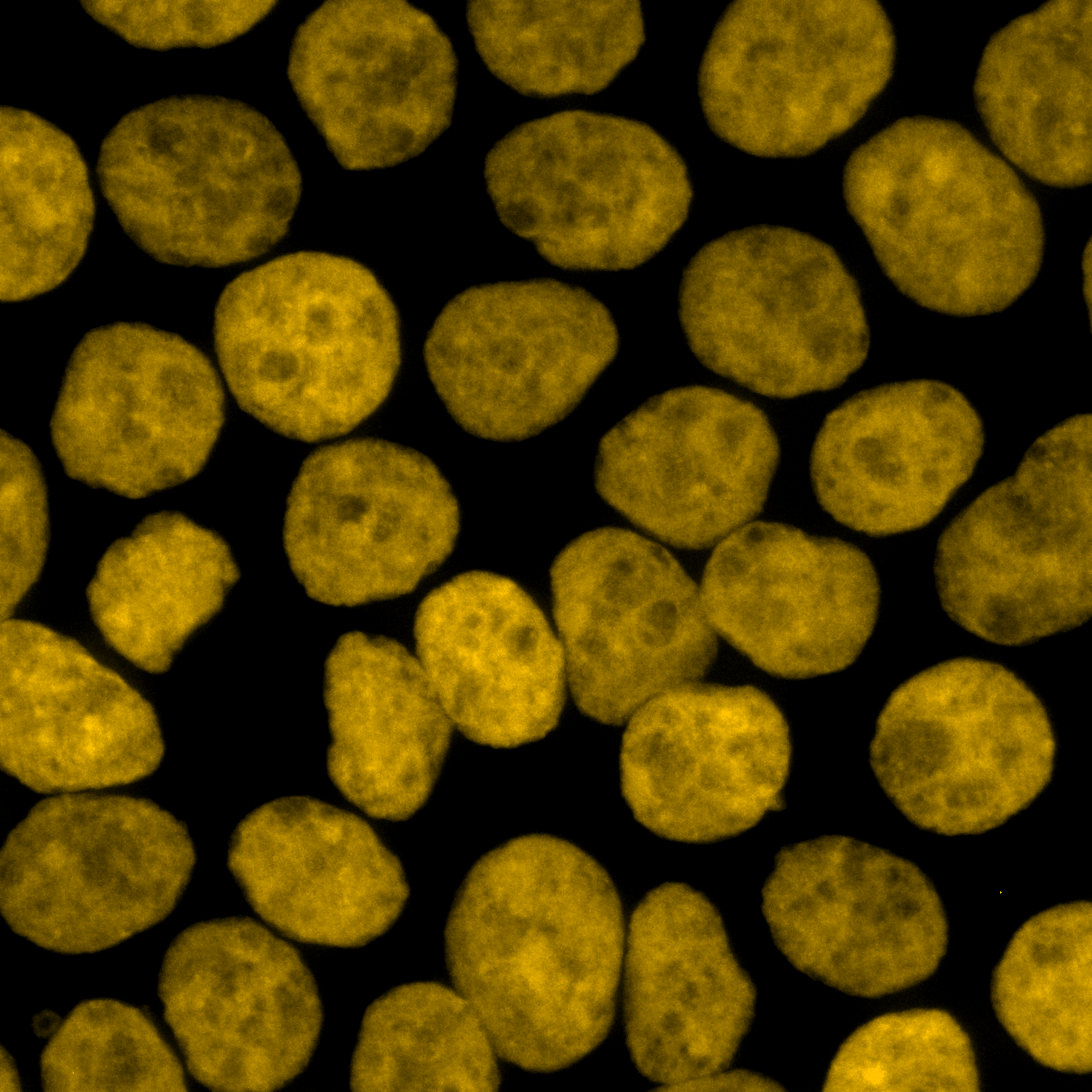

Supplement: Supplementary file 8 — Source data Fig. 2 [file 44318_2024_337_MOESM8_ESM.zip › 02_Figure_02/2E/03_MMEJ/HEK-MMEJ-GFP.tif]

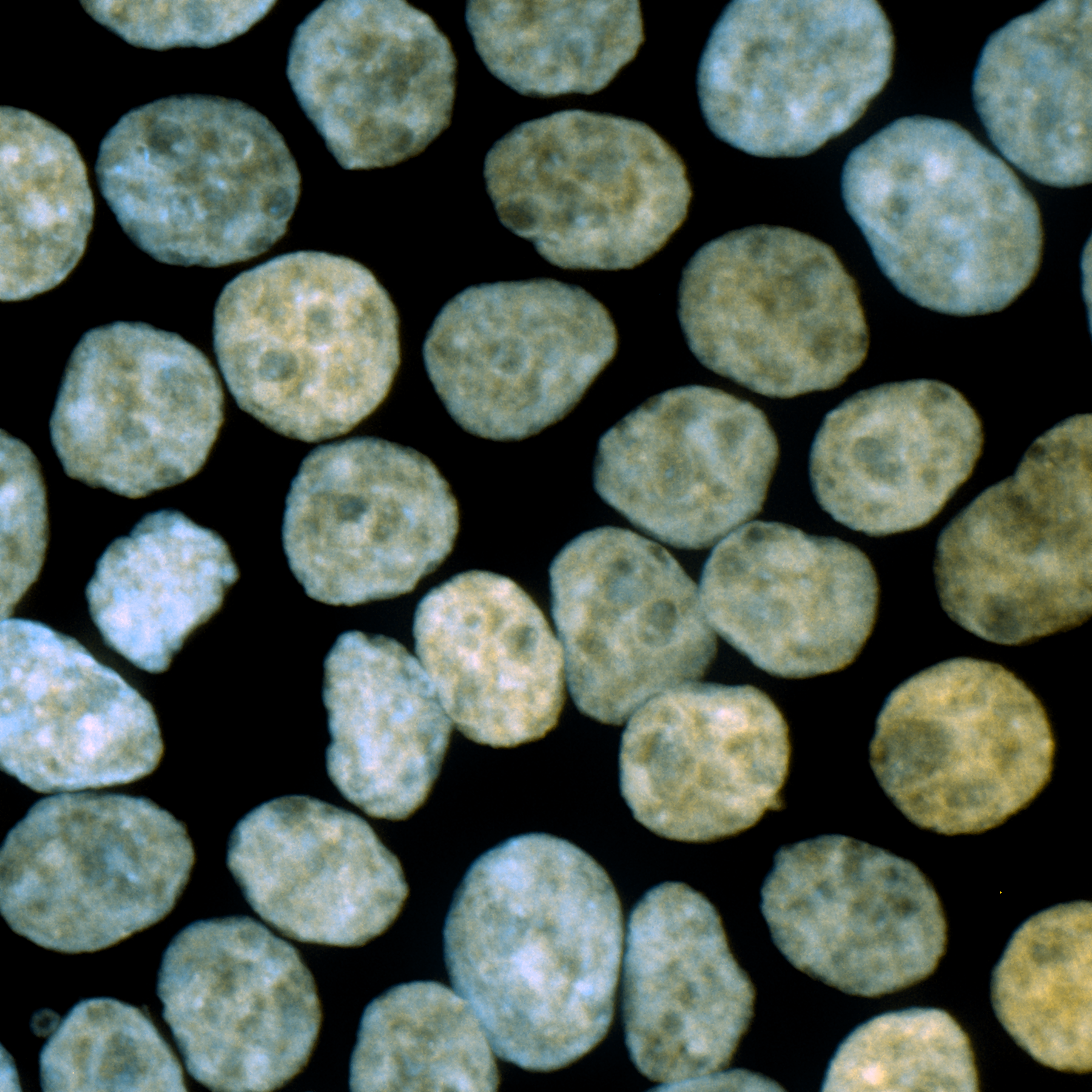

Supplement: Supplementary file 8 — Source data Fig. 2 [file 44318_2024_337_MOESM8_ESM.zip › 02_Figure_02/2E/03_MMEJ/HEK-MMEJ-Merge.tif]

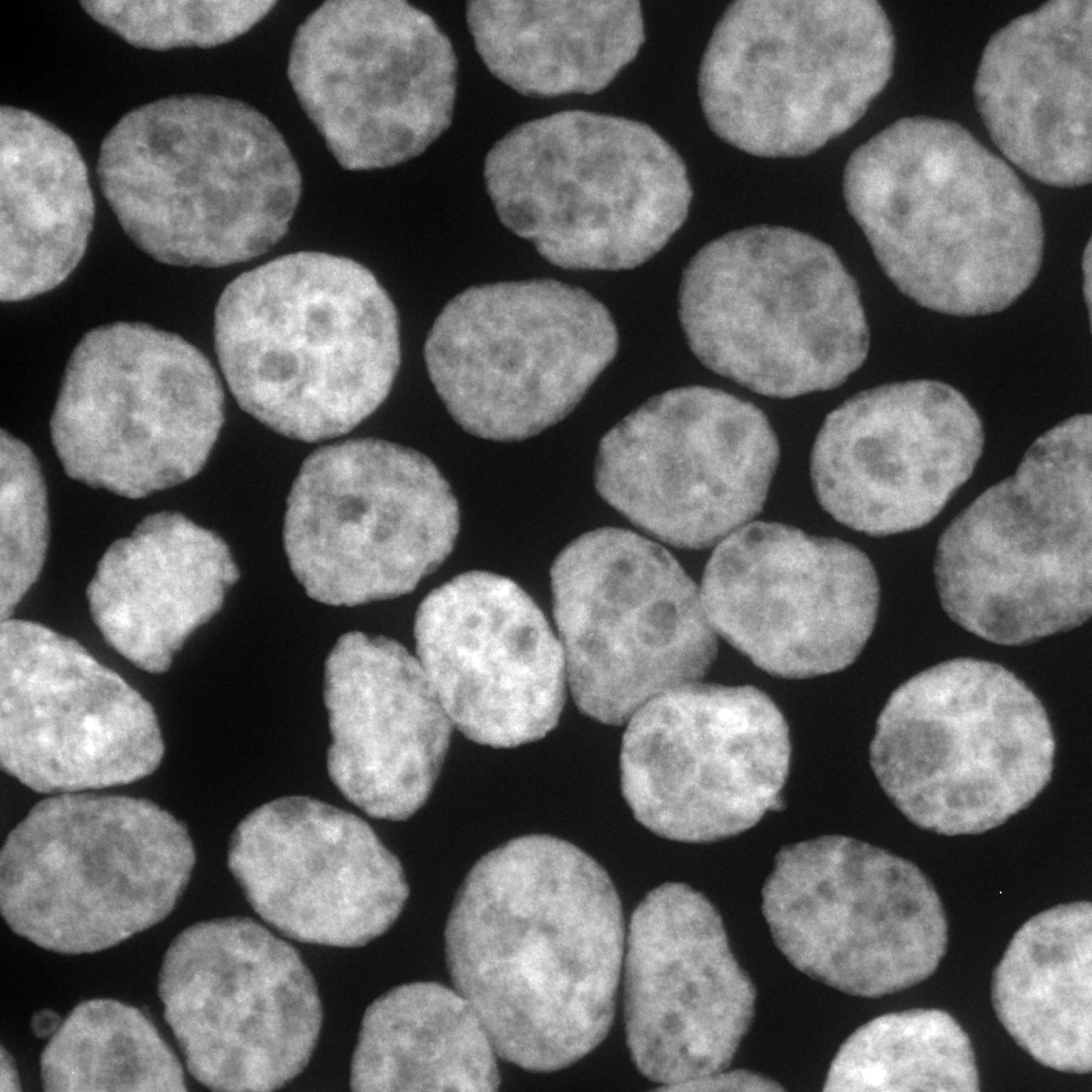

Supplement: Supplementary file 8 — Source data Fig. 2 [file 44318_2024_337_MOESM8_ESM.zip › 02_Figure_02/2E/03_MMEJ/_FULL-RANGE-HEK-MMEJ.tif]

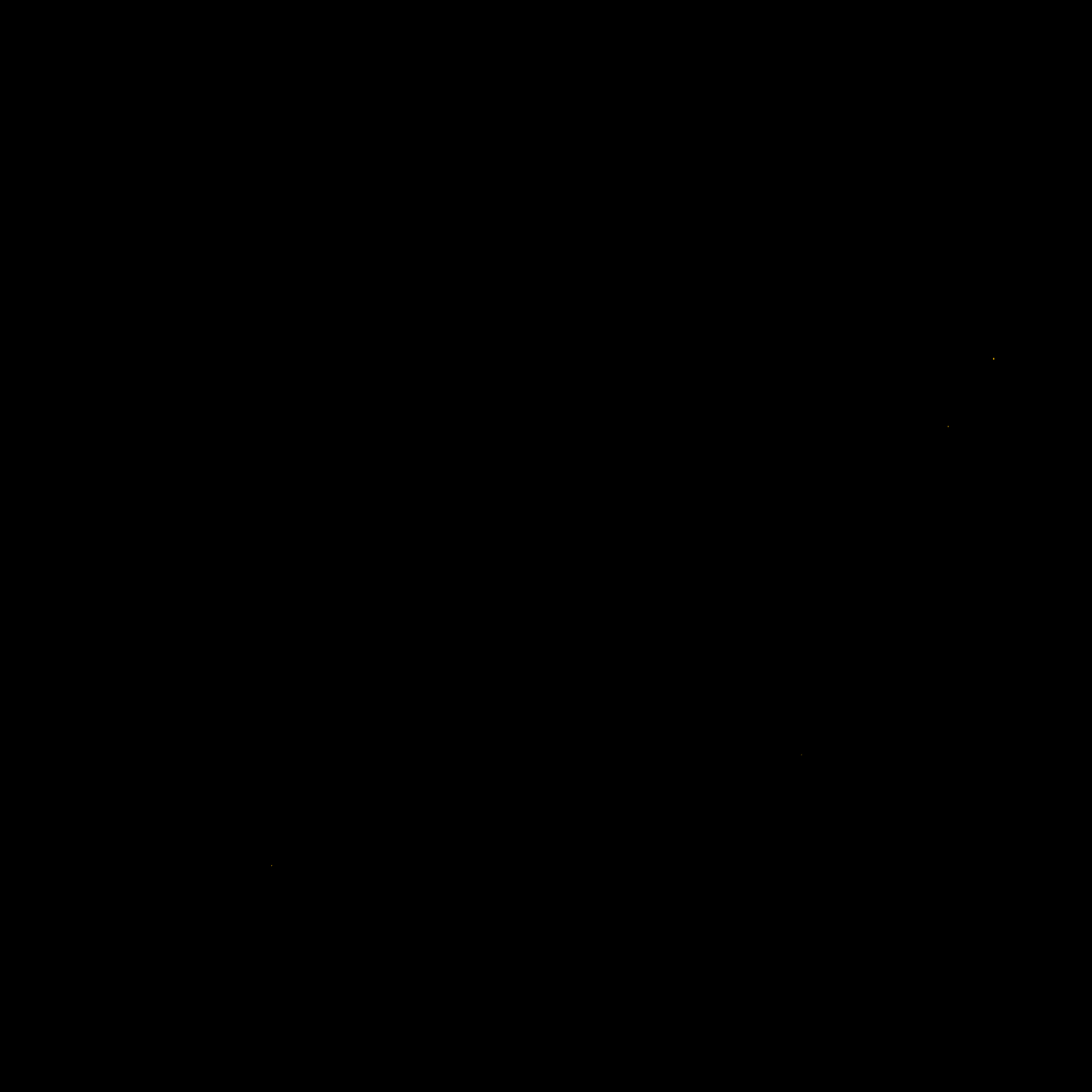

Supplement: Supplementary file 9 — Source data Fig. 3 [file 44318_2024_337_MOESM9_ESM.zip › 03_Figure_03/3E/01_WT/WT-GFP.tif]

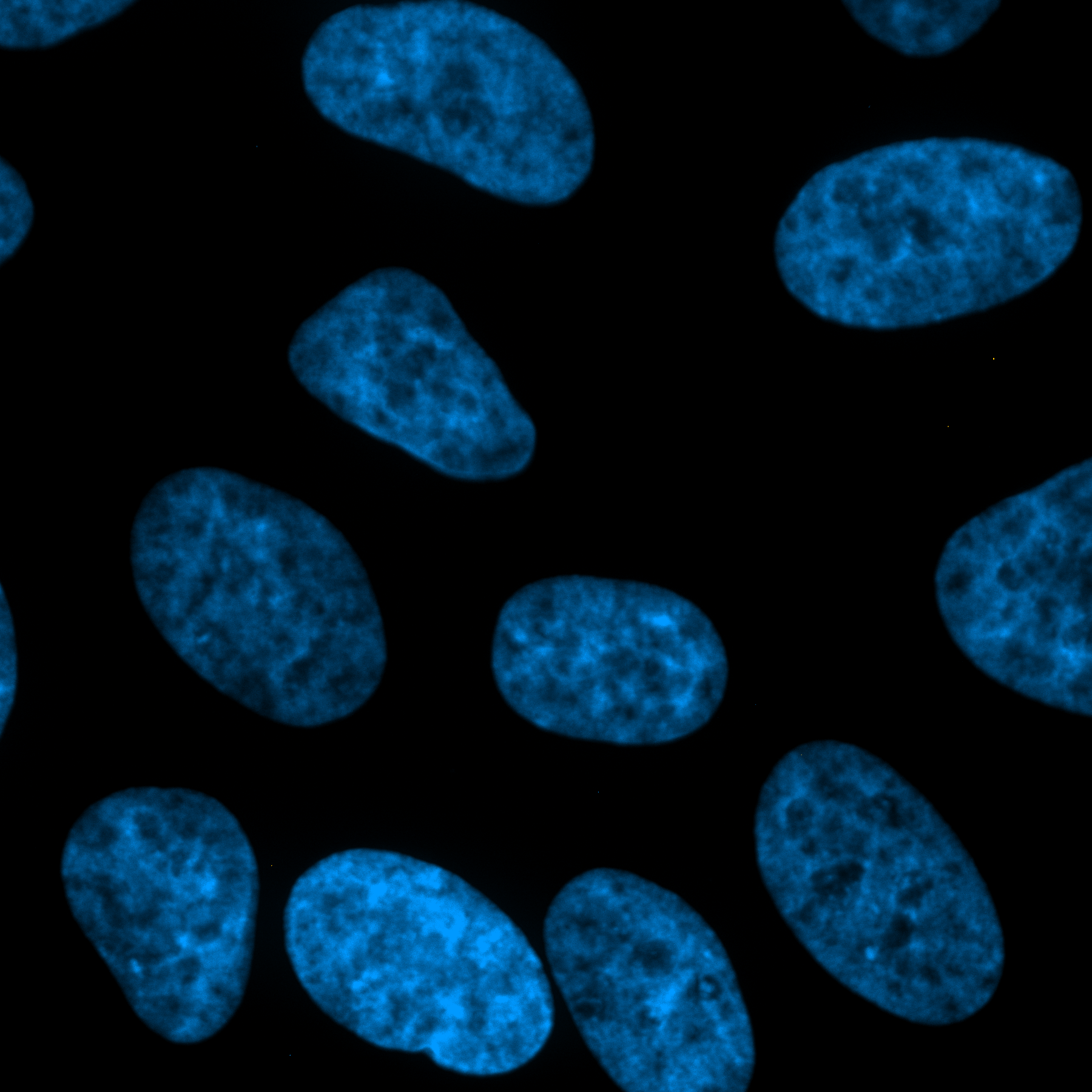

Supplement: Supplementary file 9 — Source data Fig. 3 [file 44318_2024_337_MOESM9_ESM.zip › 03_Figure_03/3E/01_WT/WT-Merge.tif]

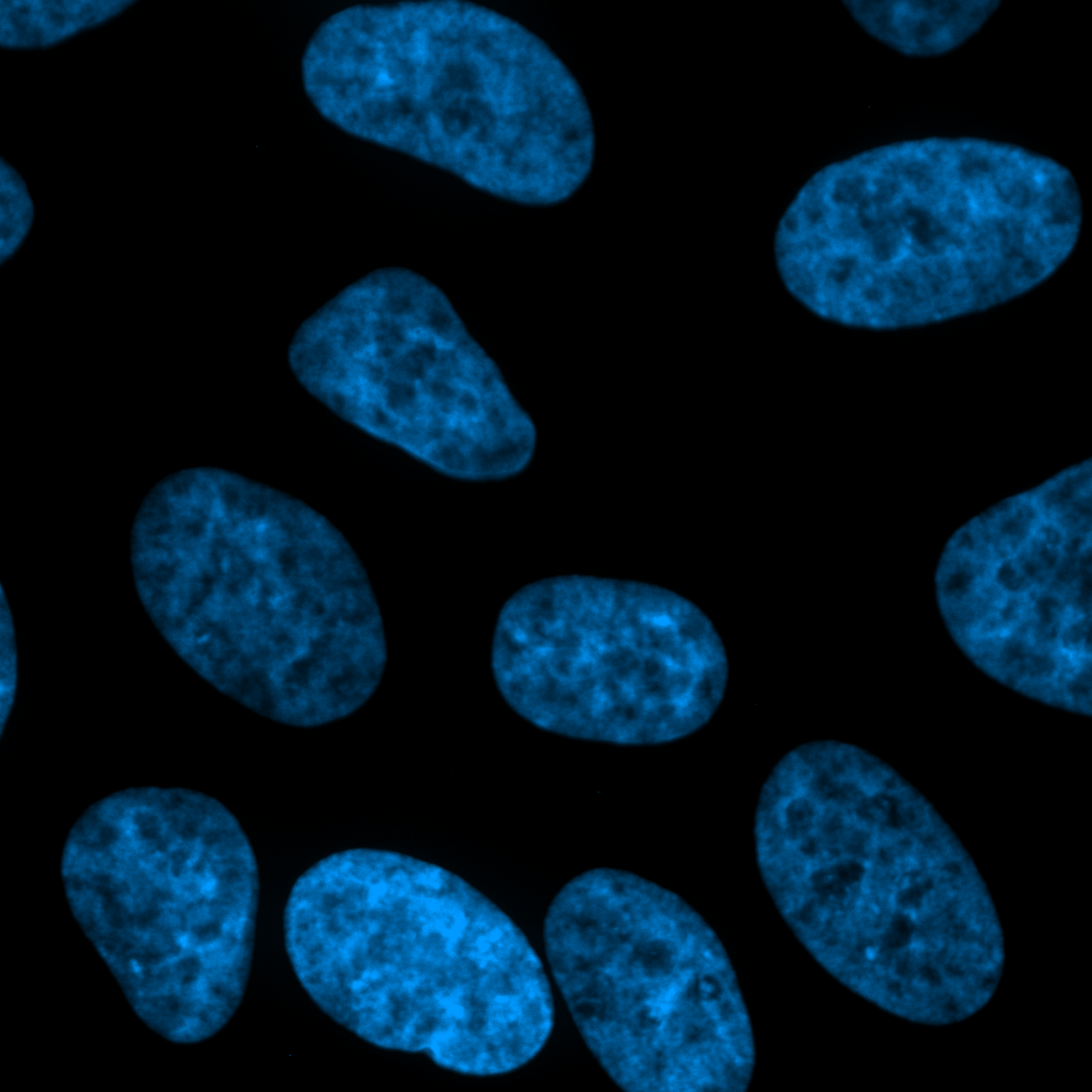

Supplement: Supplementary file 9 — Source data Fig. 3 [file 44318_2024_337_MOESM9_ESM.zip › 03_Figure_03/3E/01_WT/WT-mNeon.tif]

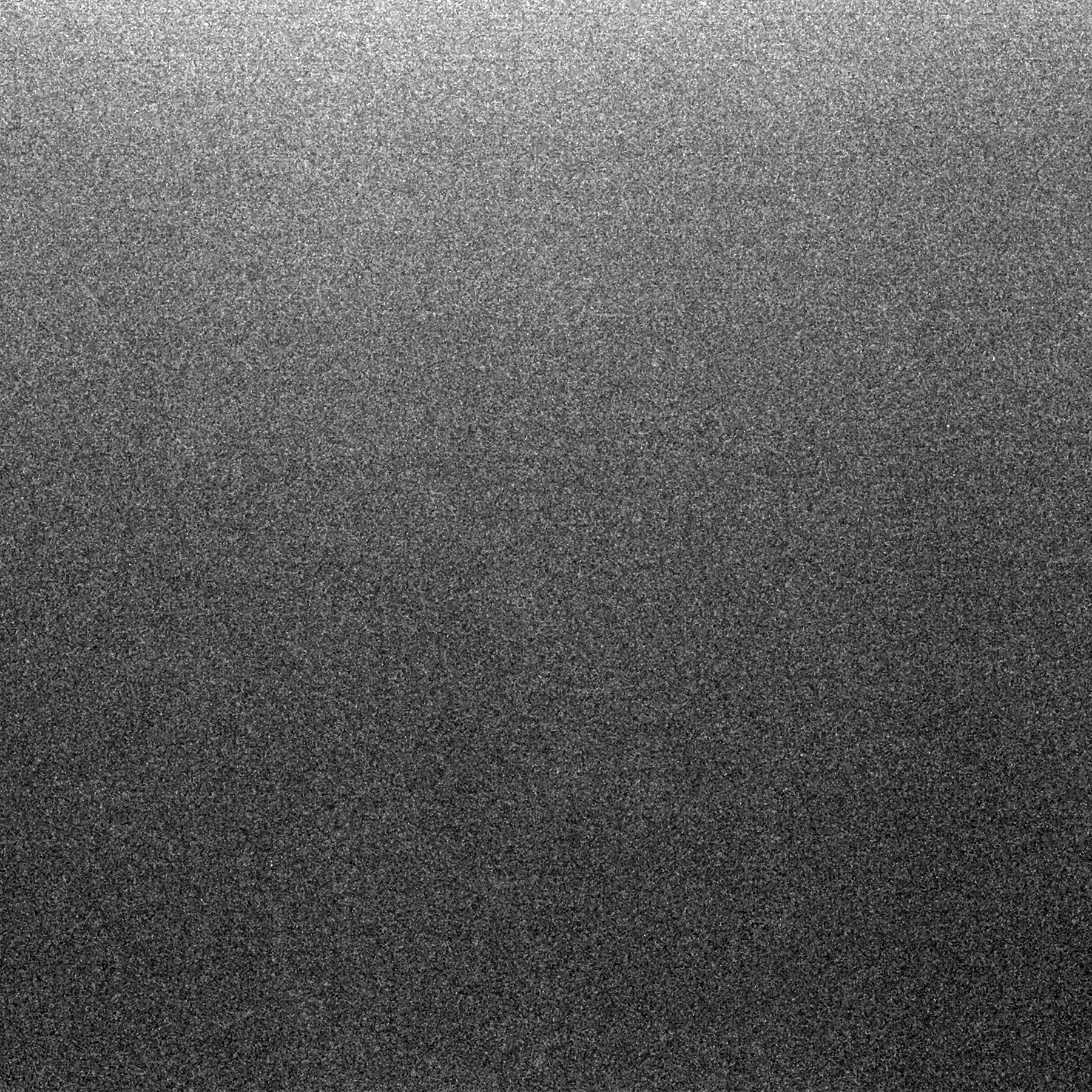

Supplement: Supplementary file 9 — Source data Fig. 3 [file 44318_2024_337_MOESM9_ESM.zip › 03_Figure_03/3E/01_WT/_FULL-RANGE-WT.tif]

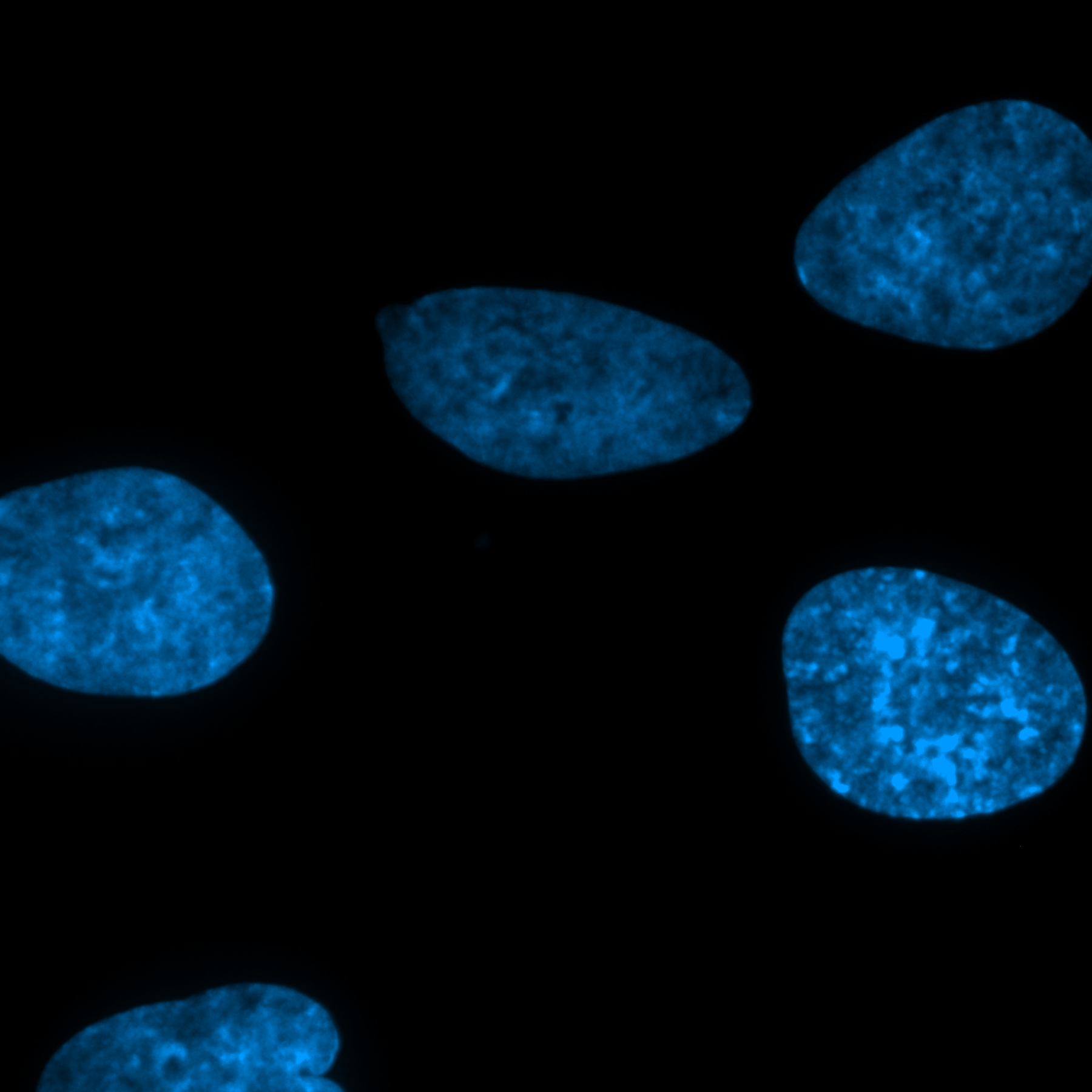

Supplement: Supplementary file 9 — Source data Fig. 3 [file 44318_2024_337_MOESM9_ESM.zip › 03_Figure_03/3E/02_Selected Pool/Selected-Pool-DAPI.tif]

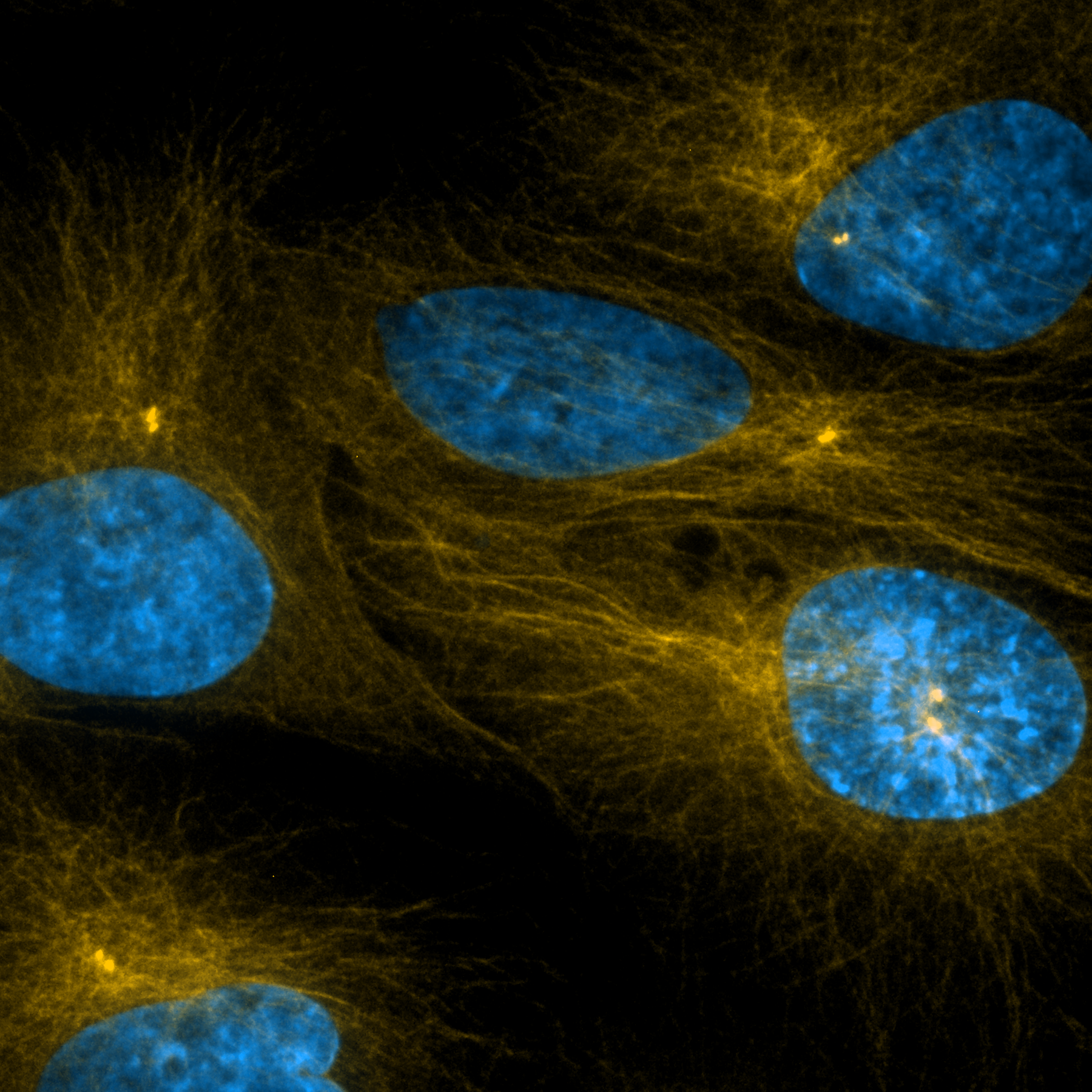

Supplement: Supplementary file 9 — Source data Fig. 3 [file 44318_2024_337_MOESM9_ESM.zip › 03_Figure_03/3E/02_Selected Pool/Selected-Pool-Merge.tif]

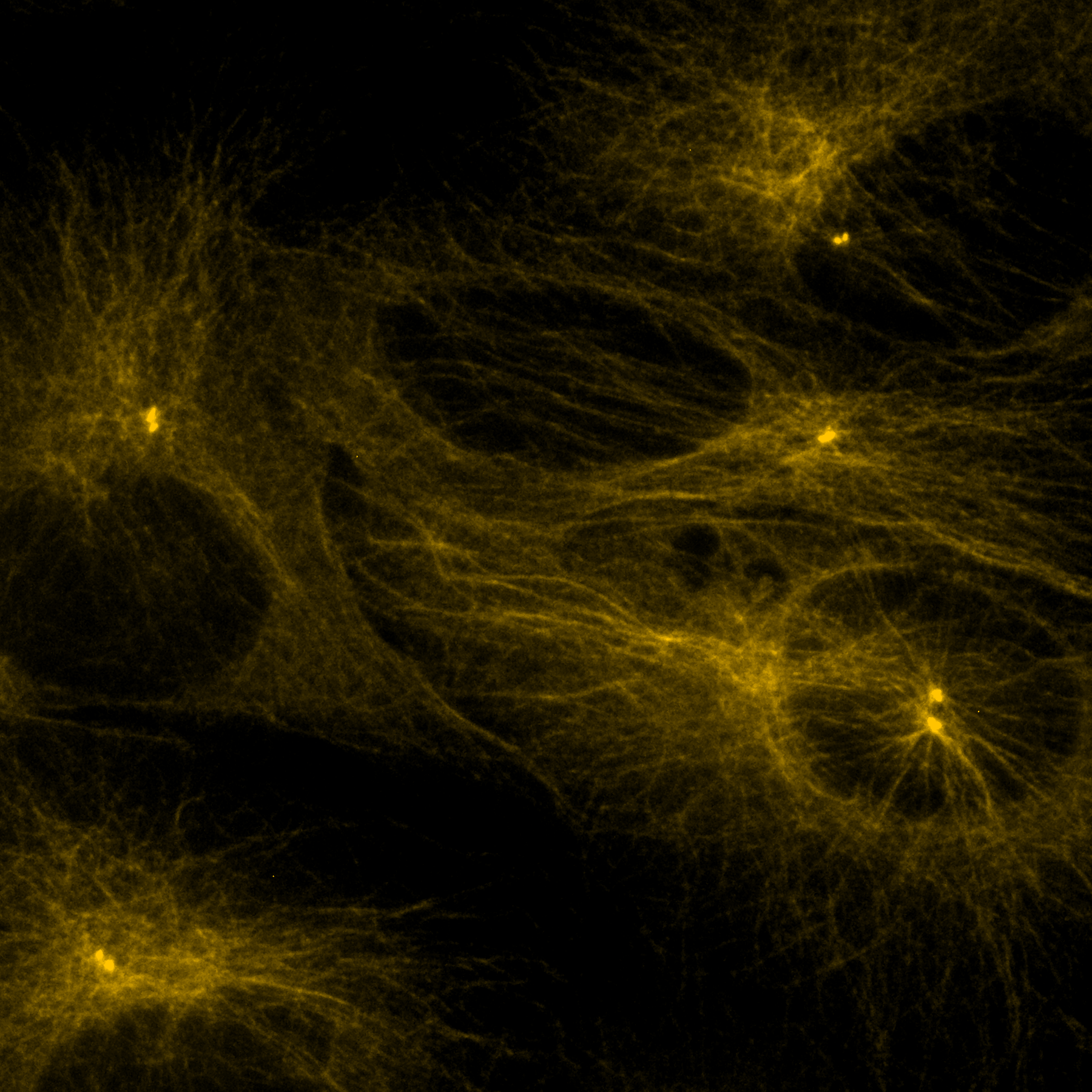

Supplement: Supplementary file 9 — Source data Fig. 3 [file 44318_2024_337_MOESM9_ESM.zip › 03_Figure_03/3E/02_Selected Pool/Selected-Pool-mNeon.tif]

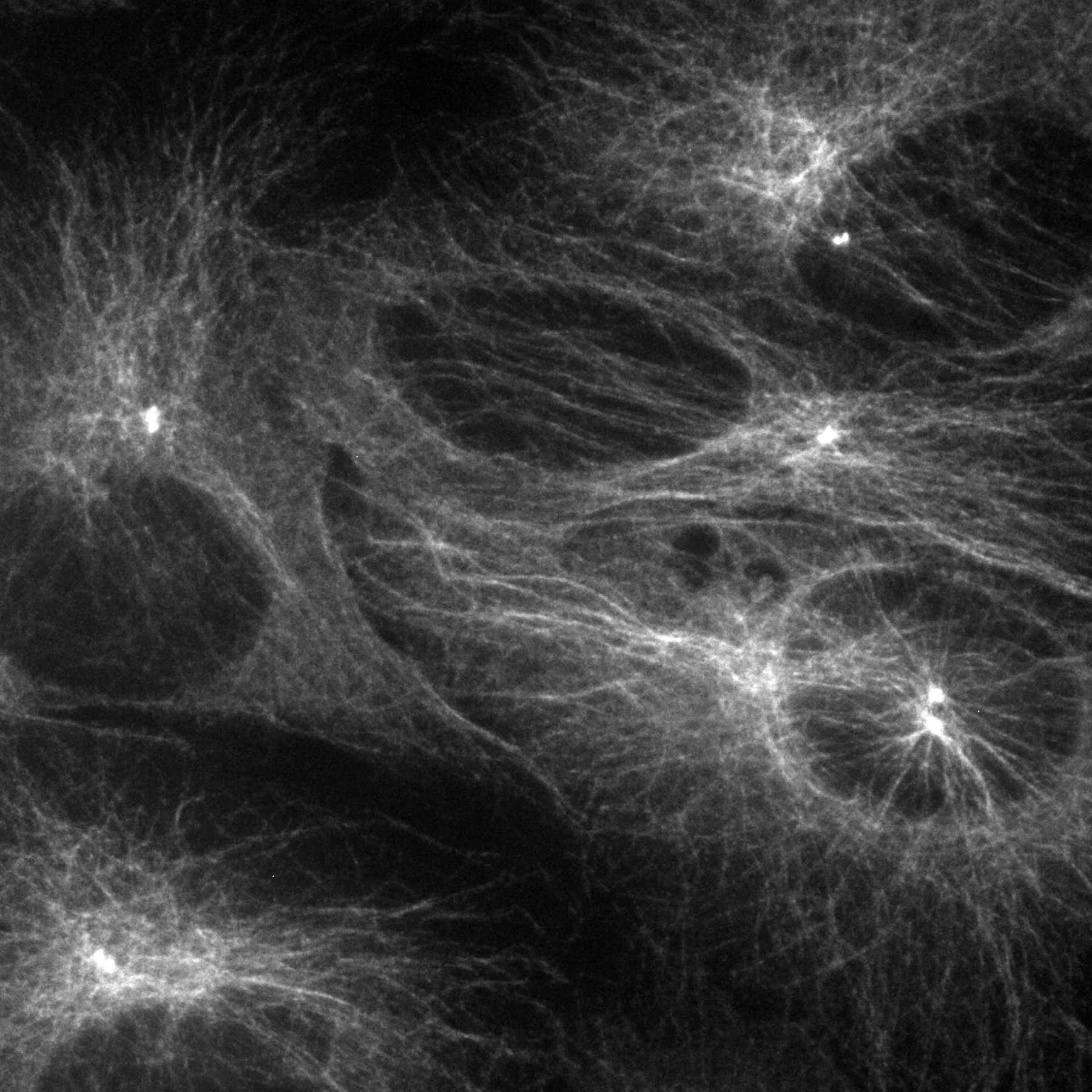

Supplement: Supplementary file 9 — Source data Fig. 3 [file 44318_2024_337_MOESM9_ESM.zip › 03_Figure_03/3E/02_Selected Pool/_FULL-RANGE-Selected-Pool.tif]

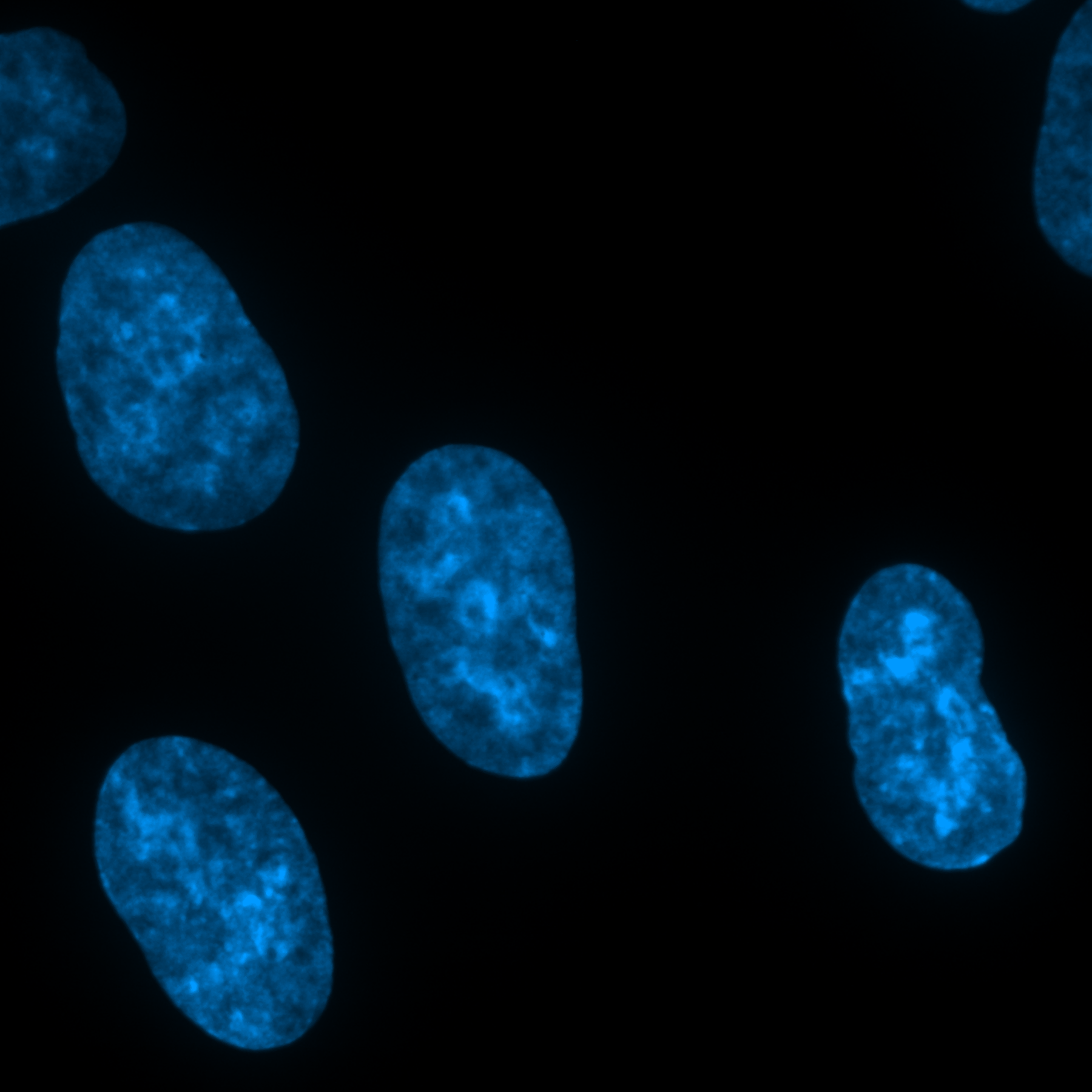

Supplement: Supplementary file 9 — Source data Fig. 3 [file 44318_2024_337_MOESM9_ESM.zip › 03_Figure_03/3E/03_Cre Pool/Cre-Pool-DAPI.tif]

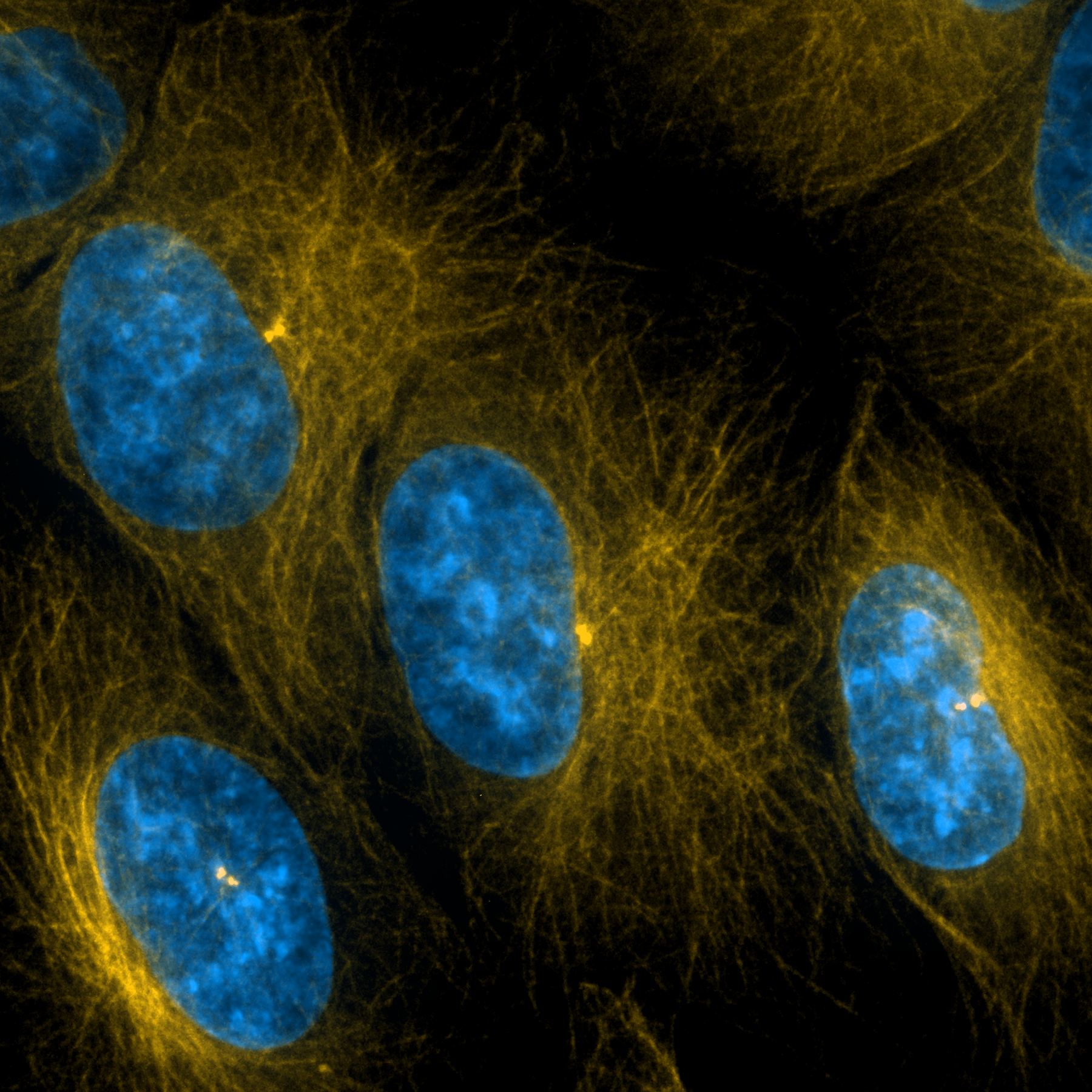

Supplement: Supplementary file 9 — Source data Fig. 3 [file 44318_2024_337_MOESM9_ESM.zip › 03_Figure_03/3E/03_Cre Pool/Cre-Pool-Merge.tif]

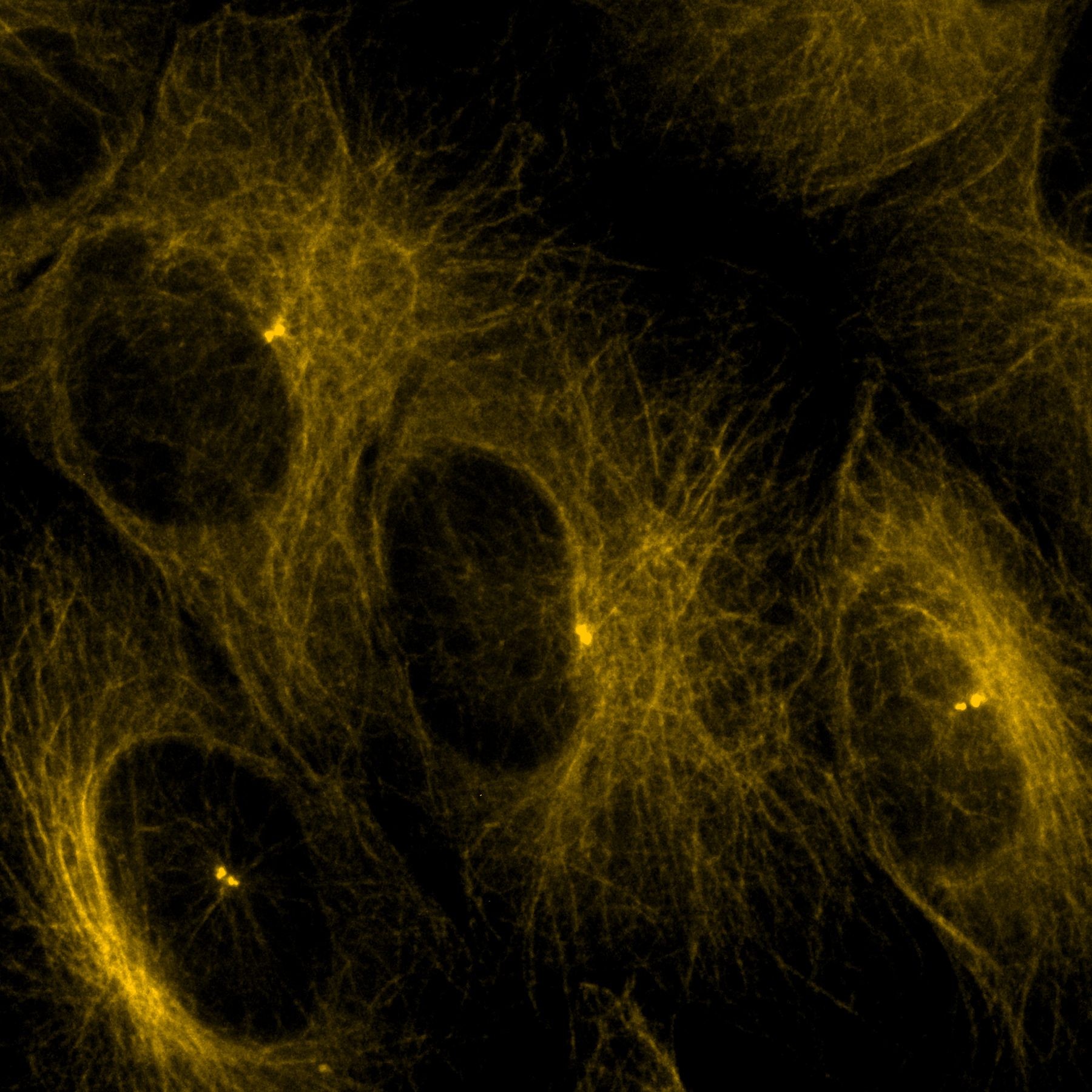

Supplement: Supplementary file 9 — Source data Fig. 3 [file 44318_2024_337_MOESM9_ESM.zip › 03_Figure_03/3E/03_Cre Pool/Cre-Pool-mNeon.tif]

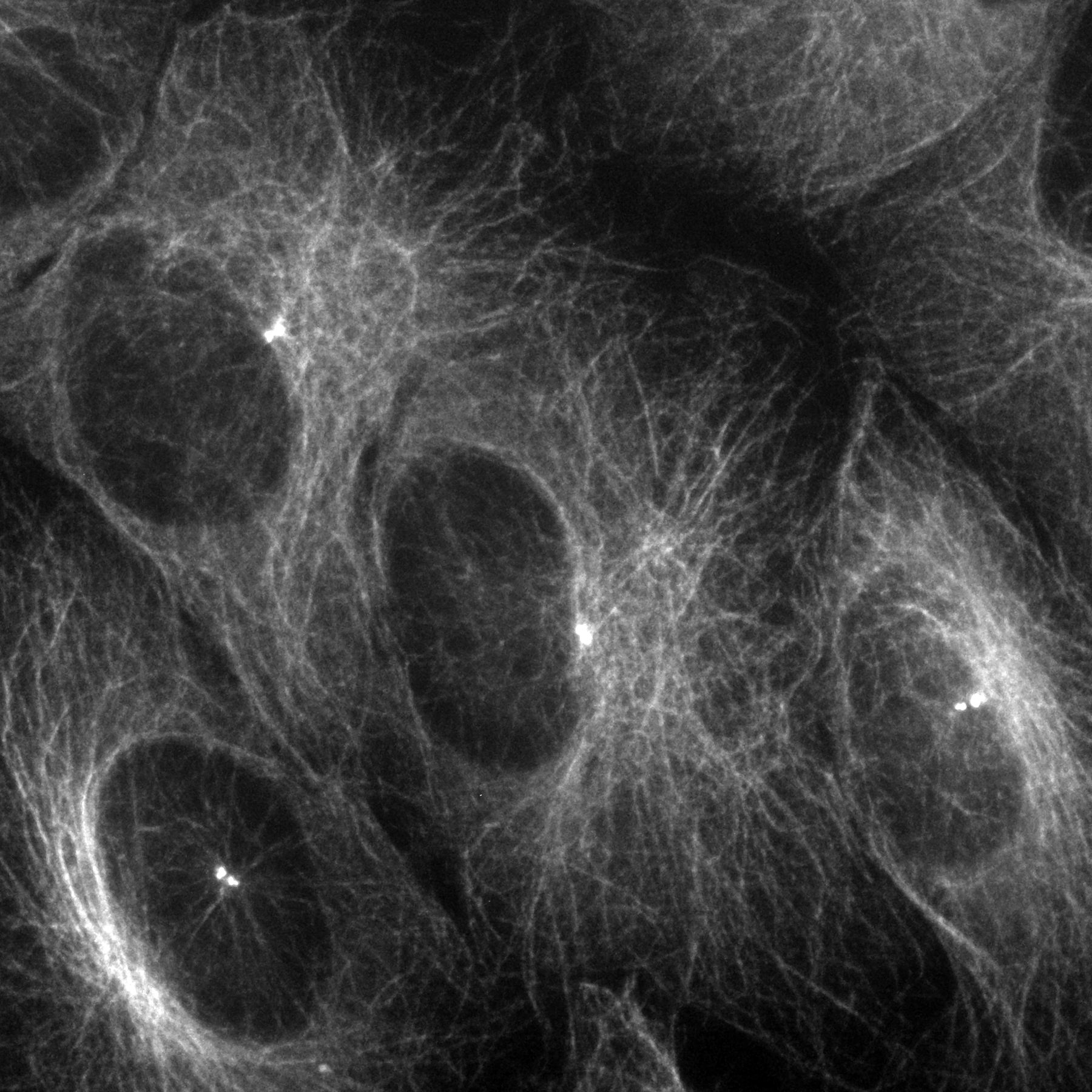

Supplement: Supplementary file 9 — Source data Fig. 3 [file 44318_2024_337_MOESM9_ESM.zip › 03_Figure_03/3E/03_Cre Pool/_FULL-RANGE-Cre-Pool.tif]

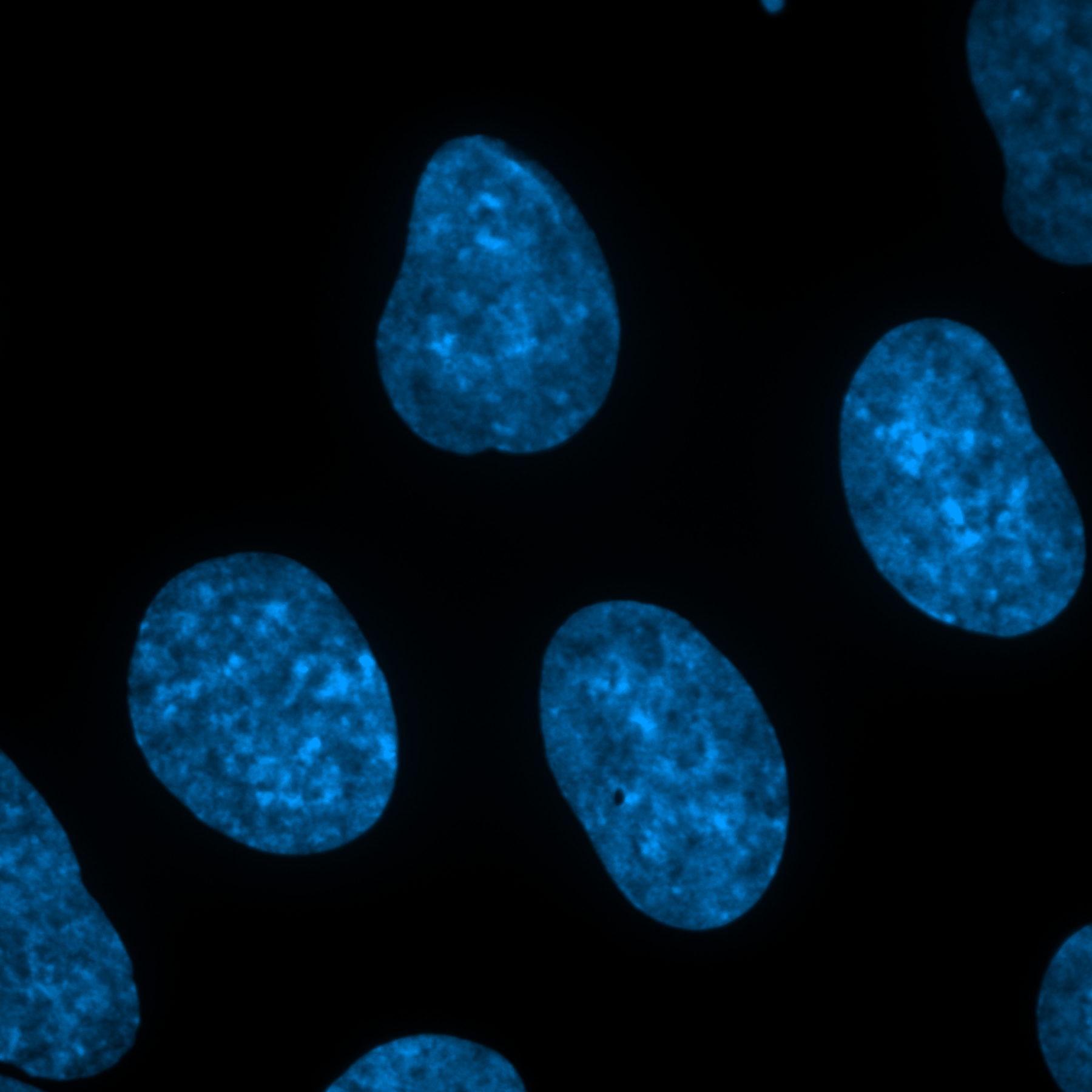

Supplement: Supplementary file 9 — Source data Fig. 3 [file 44318_2024_337_MOESM9_ESM.zip › 03_Figure_03/3E/04_Cre Clone/Cre-Clone-DAPI.tif]

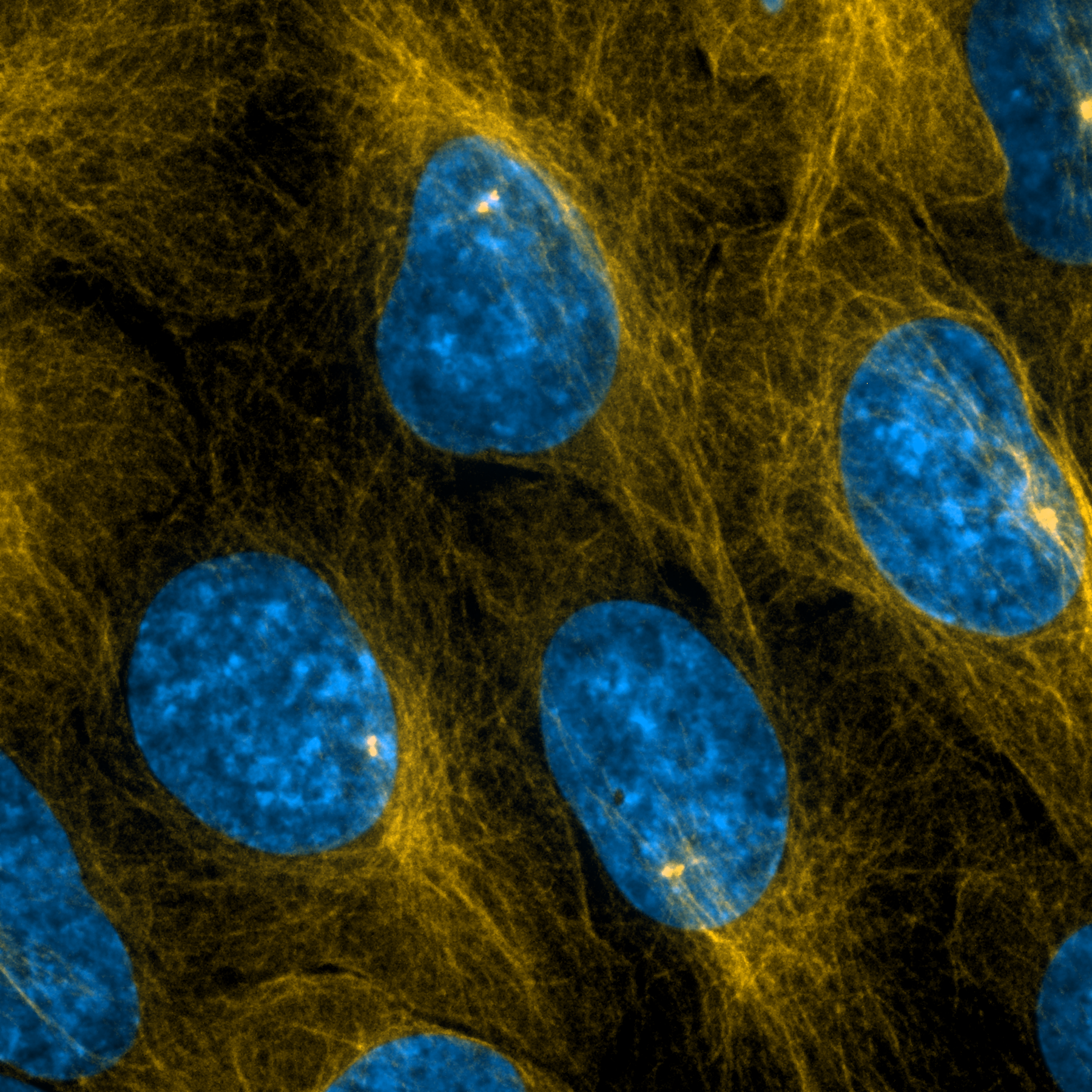

Supplement: Supplementary file 9 — Source data Fig. 3 [file 44318_2024_337_MOESM9_ESM.zip › 03_Figure_03/3E/04_Cre Clone/Cre-Clone-Merge.tif]

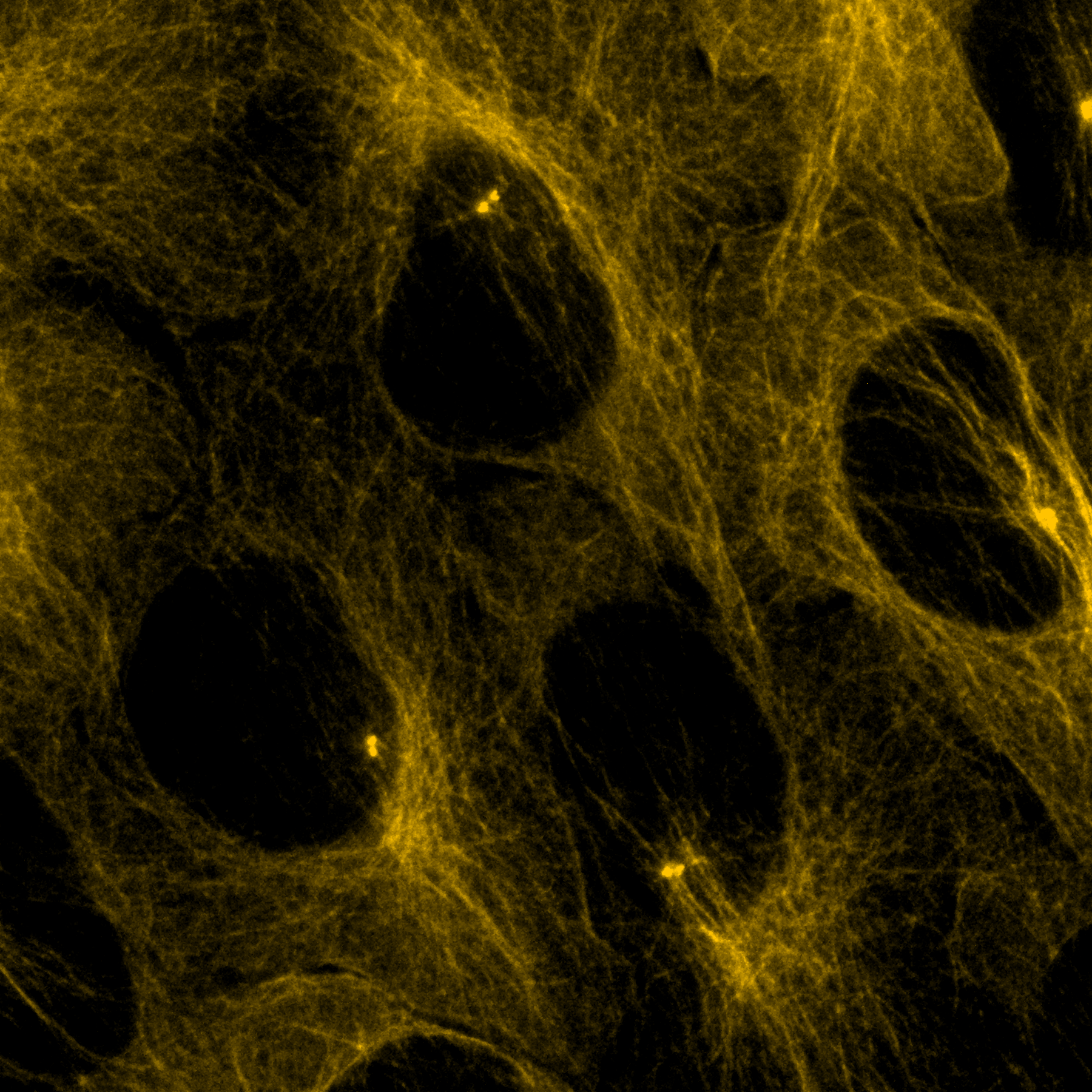

Supplement: Supplementary file 9 — Source data Fig. 3 [file 44318_2024_337_MOESM9_ESM.zip › 03_Figure_03/3E/04_Cre Clone/Cre-Clone-mNeon.tif]

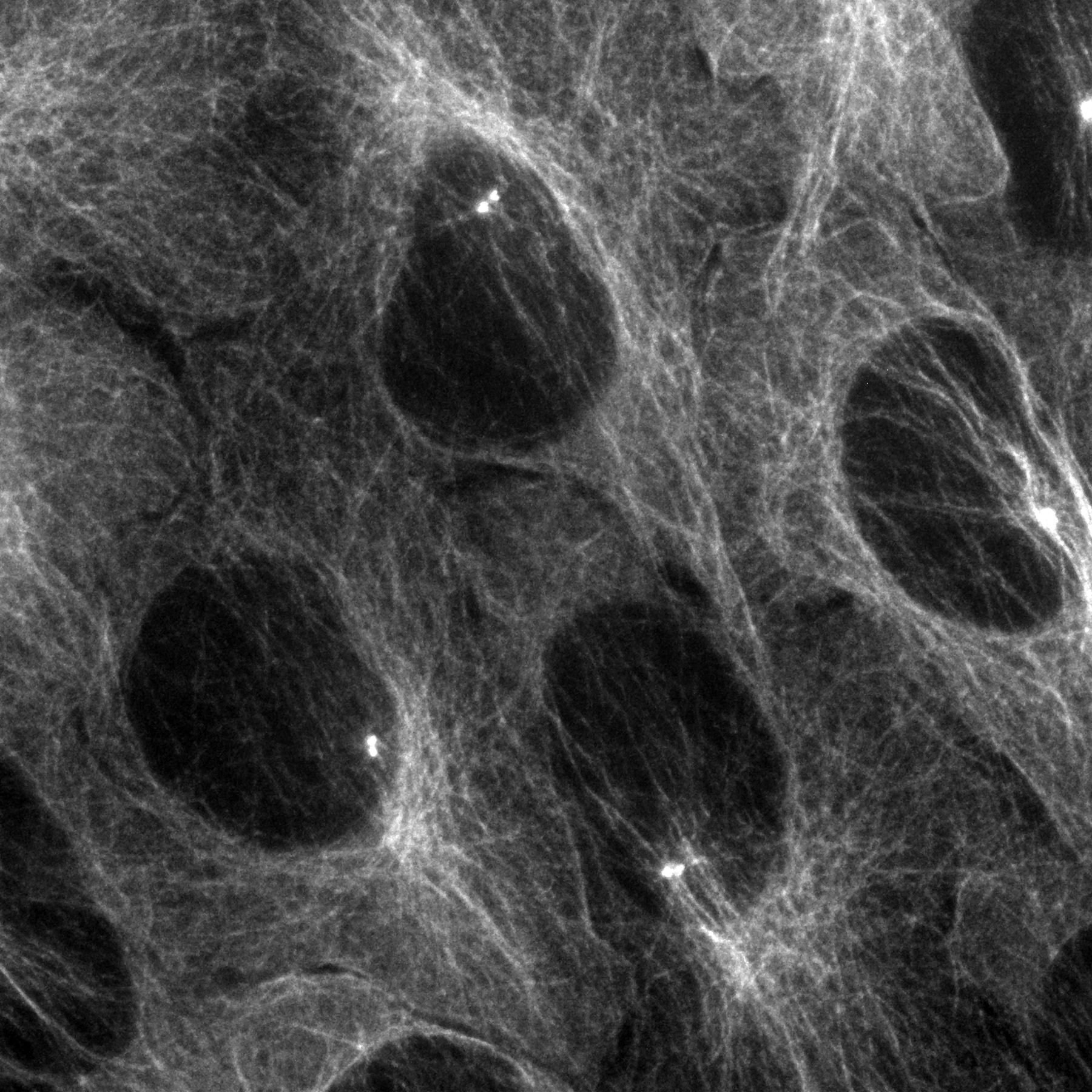

Supplement: Supplementary file 9 — Source data Fig. 3 [file 44318_2024_337_MOESM9_ESM.zip › 03_Figure_03/3E/04_Cre Clone/_FULL-RANGE-Cre-Clone.tif]

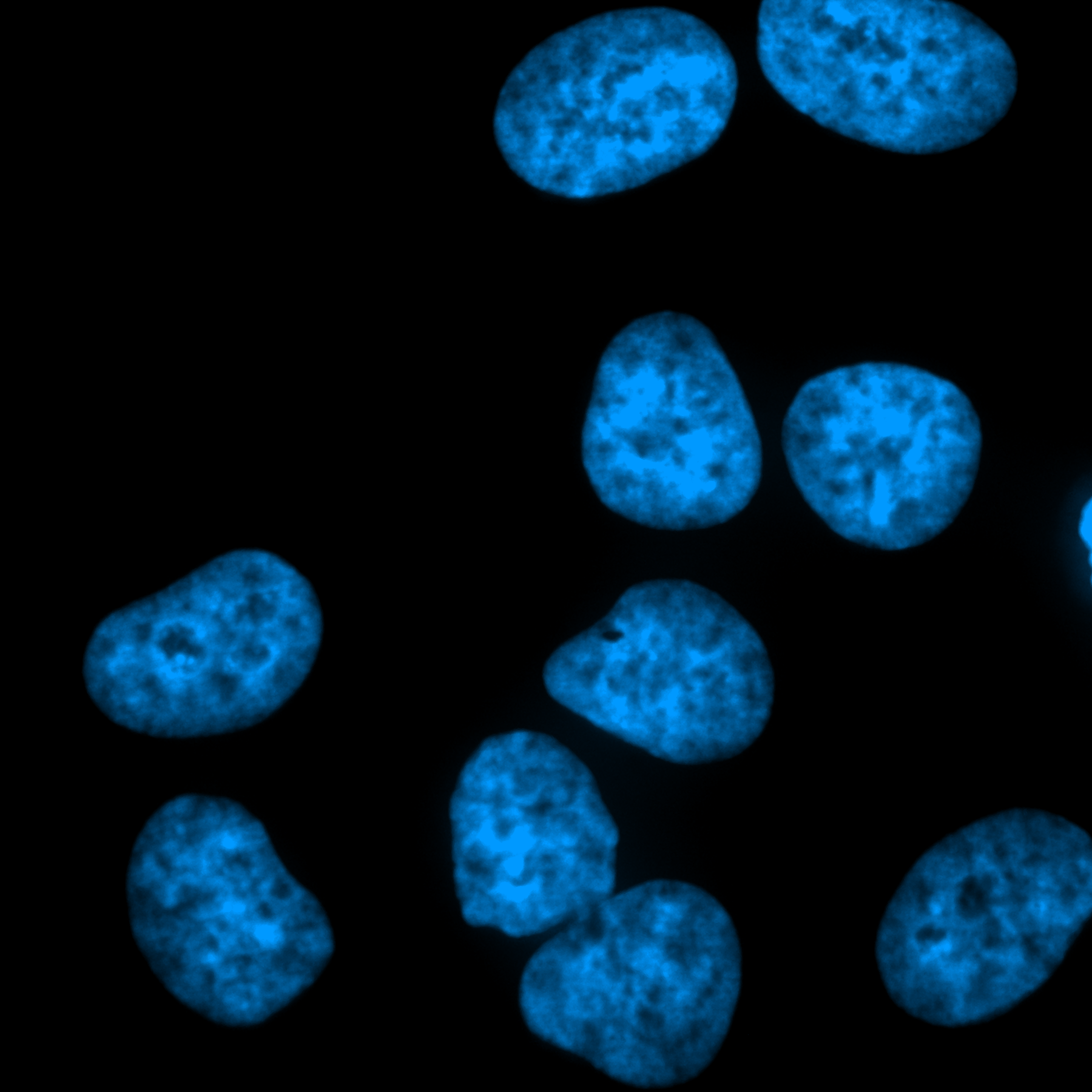

Supplement: Supplementary file 9 — Source data Fig. 3 [file 44318_2024_337_MOESM9_ESM.zip › 03_Figure_03/3G/01-WT/WT-DAPI.tif]

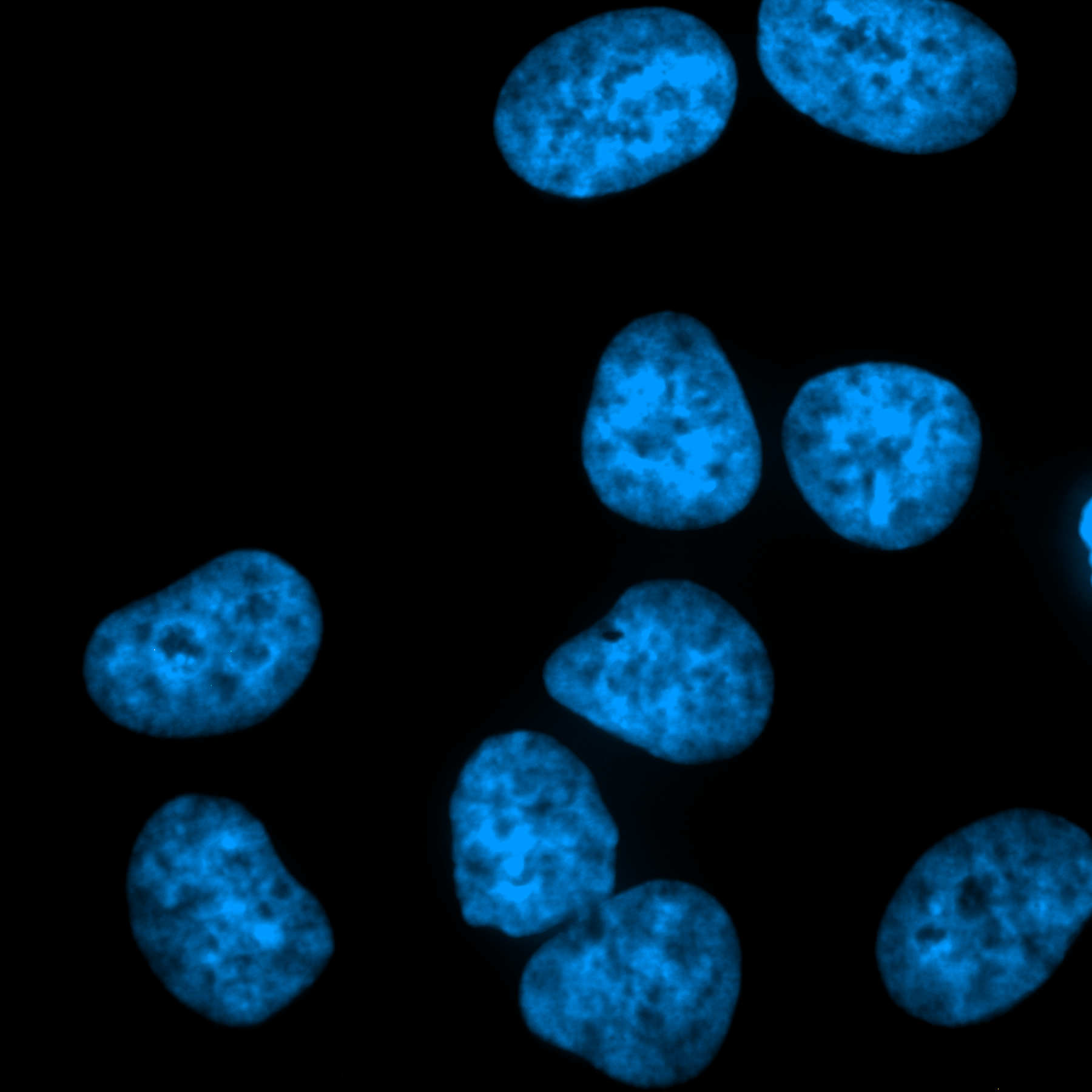

Supplement: Supplementary file 9 — Source data Fig. 3 [file 44318_2024_337_MOESM9_ESM.zip › 03_Figure_03/3G/01-WT/WT-Merge.tif]

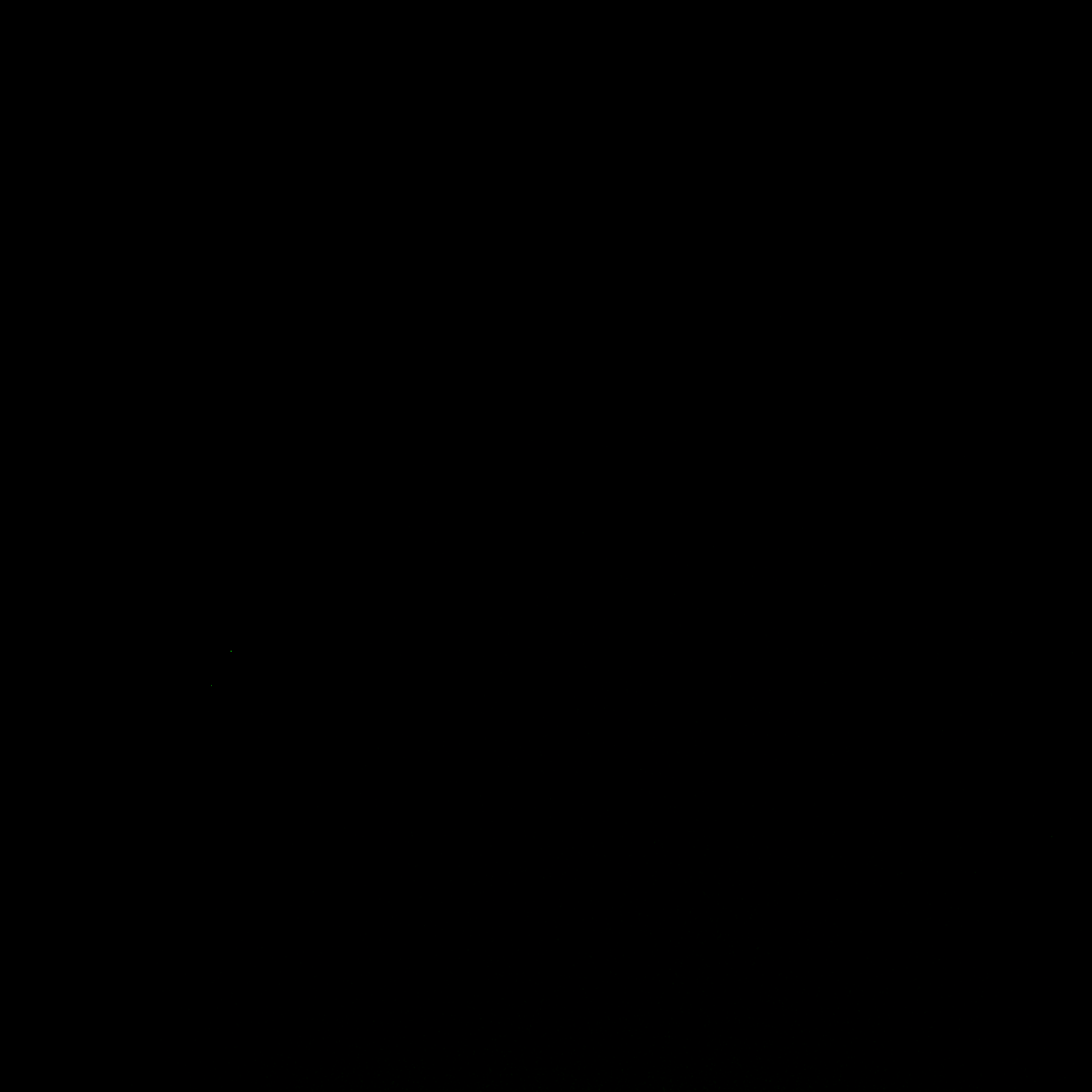

Supplement: Supplementary file 9 — Source data Fig. 3 [file 44318_2024_337_MOESM9_ESM.zip › 03_Figure_03/3G/01-WT/WT-miRFPnano3.tif]

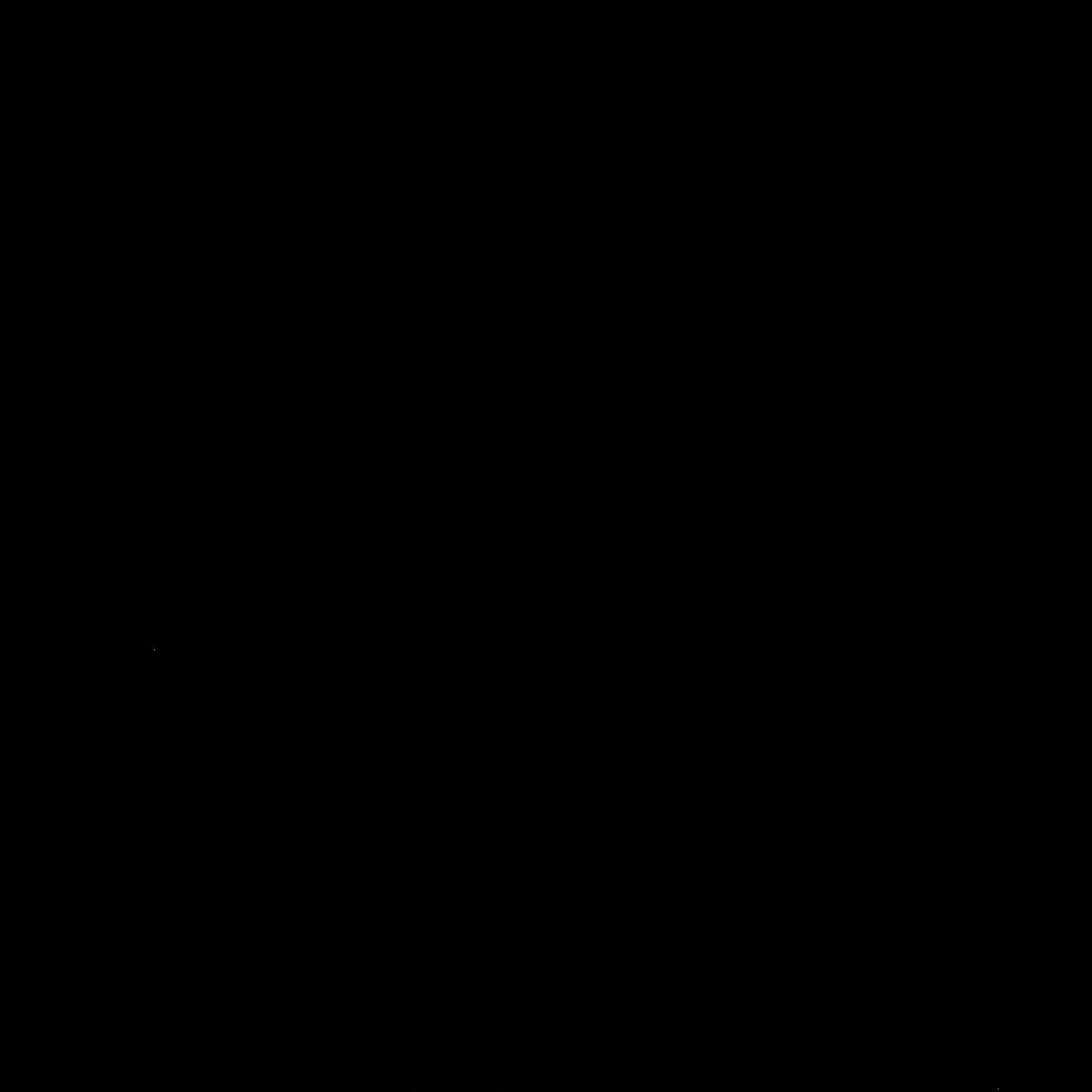

Supplement: Supplementary file 9 — Source data Fig. 3 [file 44318_2024_337_MOESM9_ESM.zip › 03_Figure_03/3G/01-WT/WT-mNeon.tif]

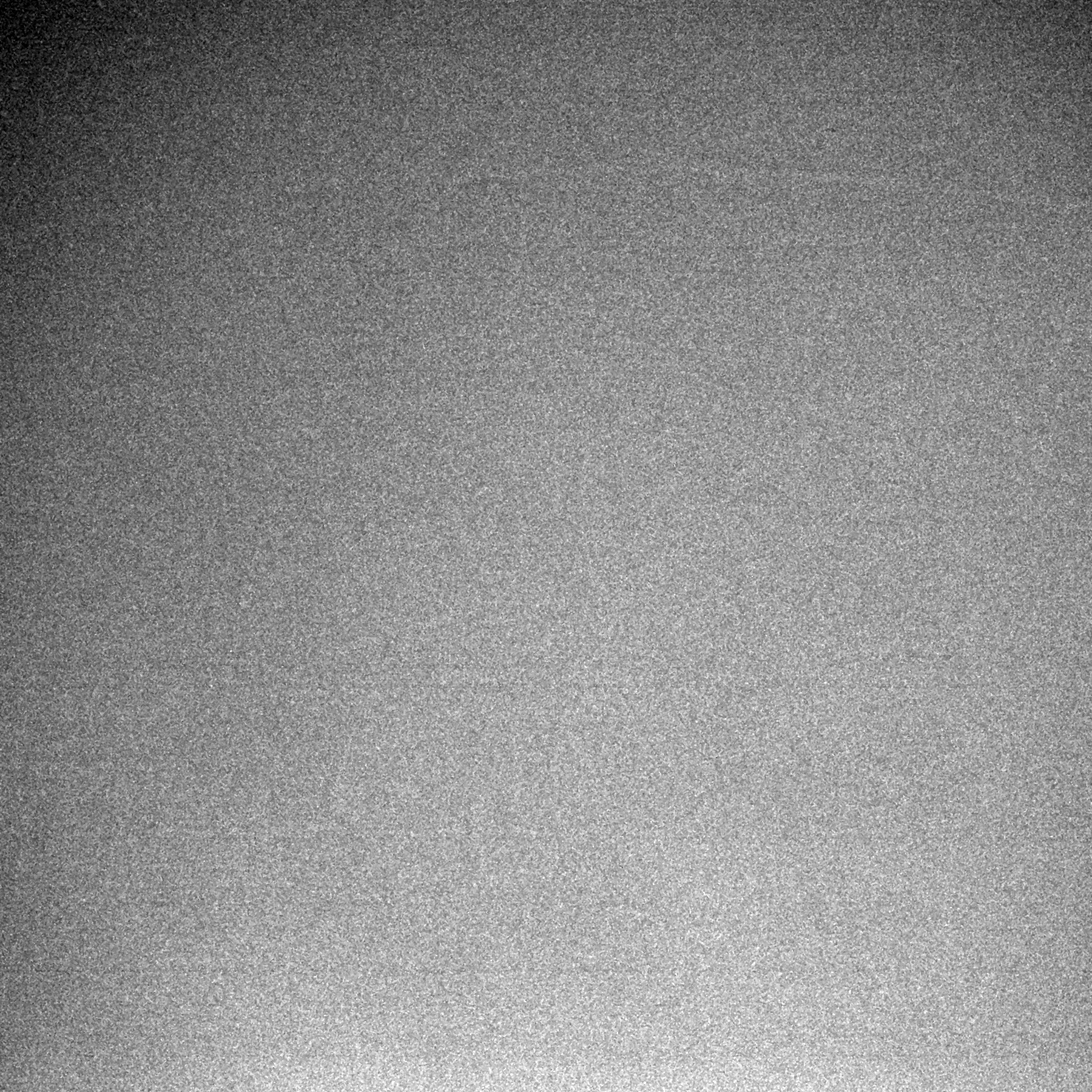

Supplement: Supplementary file 9 — Source data Fig. 3 [file 44318_2024_337_MOESM9_ESM.zip › 03_Figure_03/3G/01-WT/_FULL-RANGE-WT.tif]

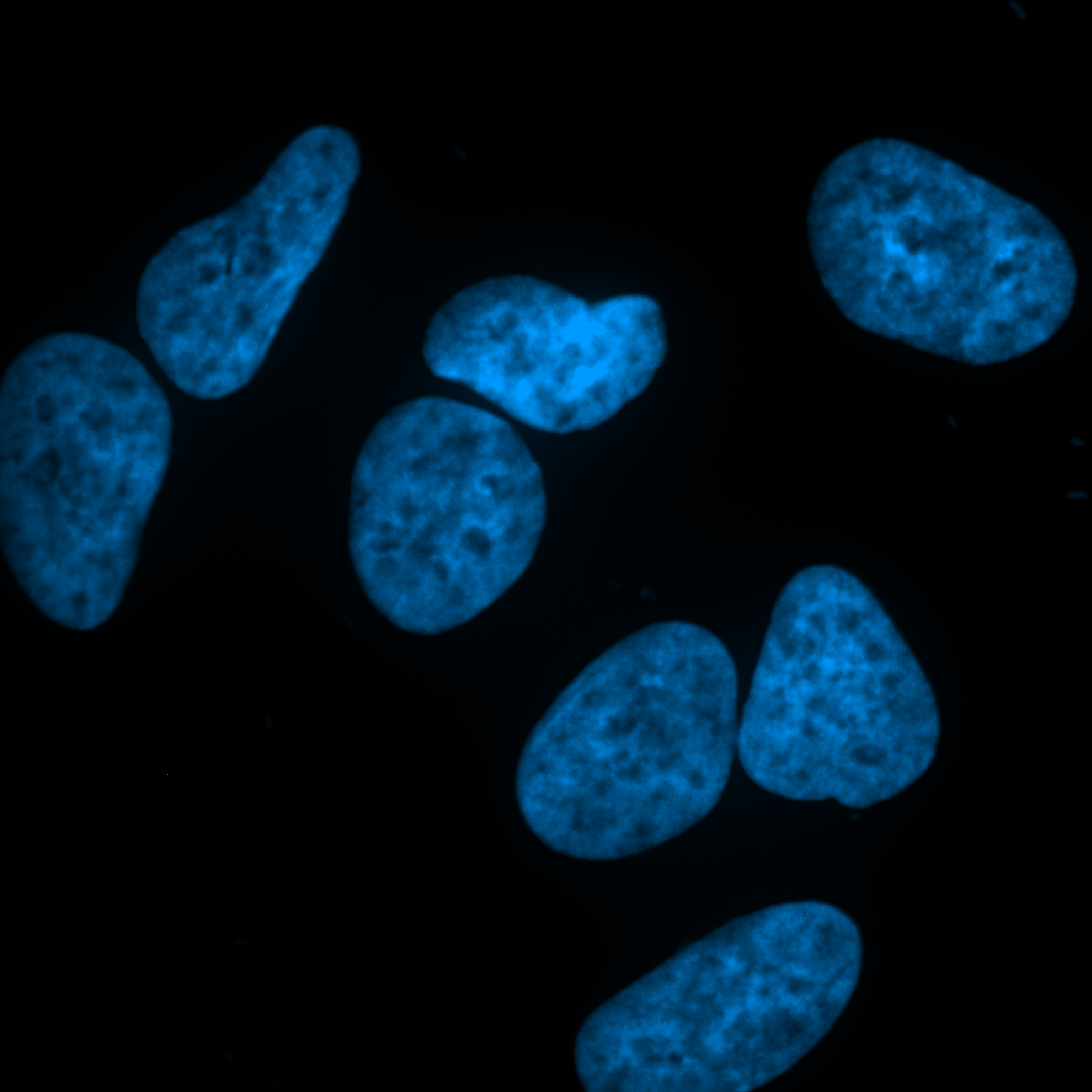

Supplement: Supplementary file 9 — Source data Fig. 3 [file 44318_2024_337_MOESM9_ESM.zip › 03_Figure_03/3G/02-TUBB4B-mNeon/Cre-Clone-DAPI.tif]

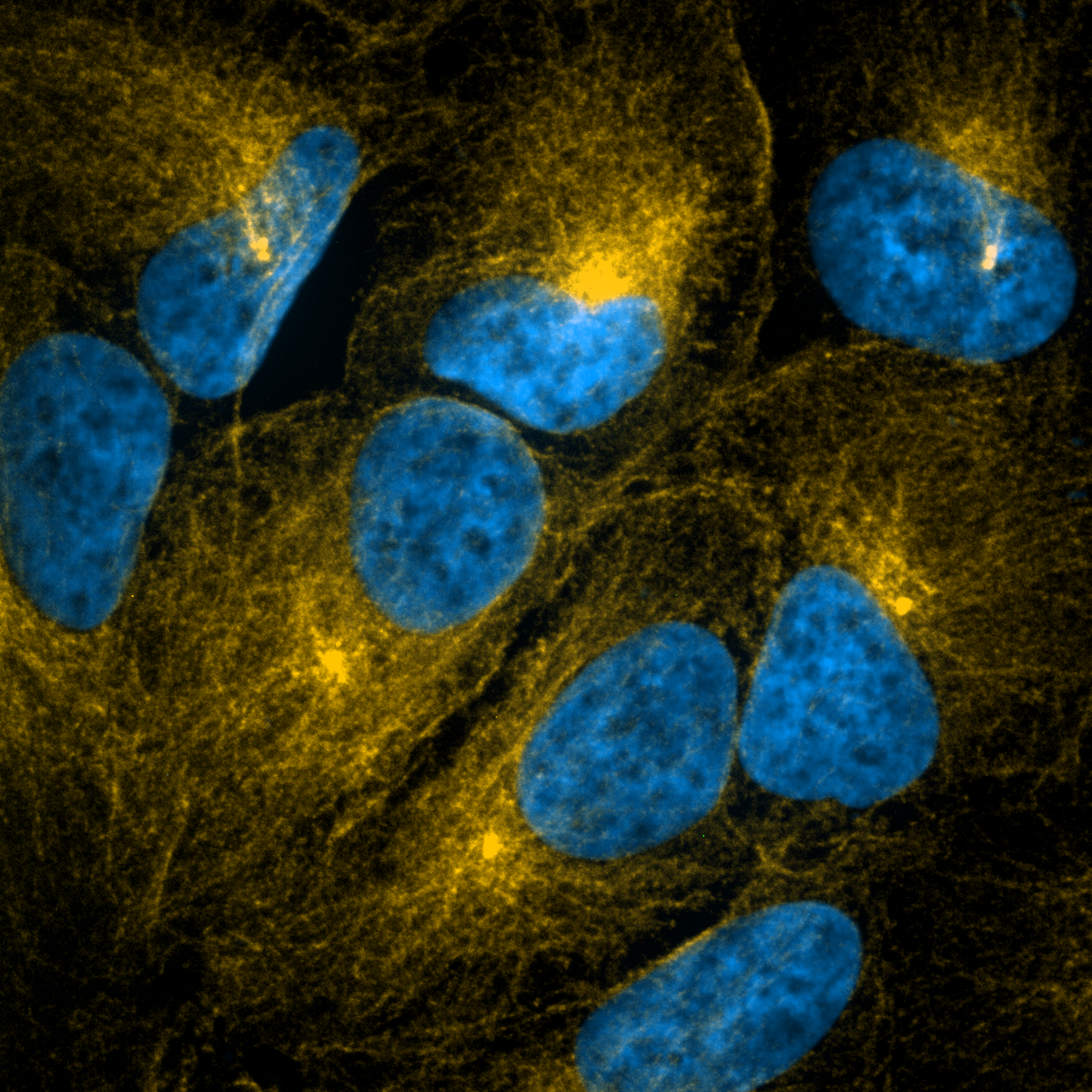

Supplement: Supplementary file 9 — Source data Fig. 3 [file 44318_2024_337_MOESM9_ESM.zip › 03_Figure_03/3G/02-TUBB4B-mNeon/Cre-Clone-Merge.tif]

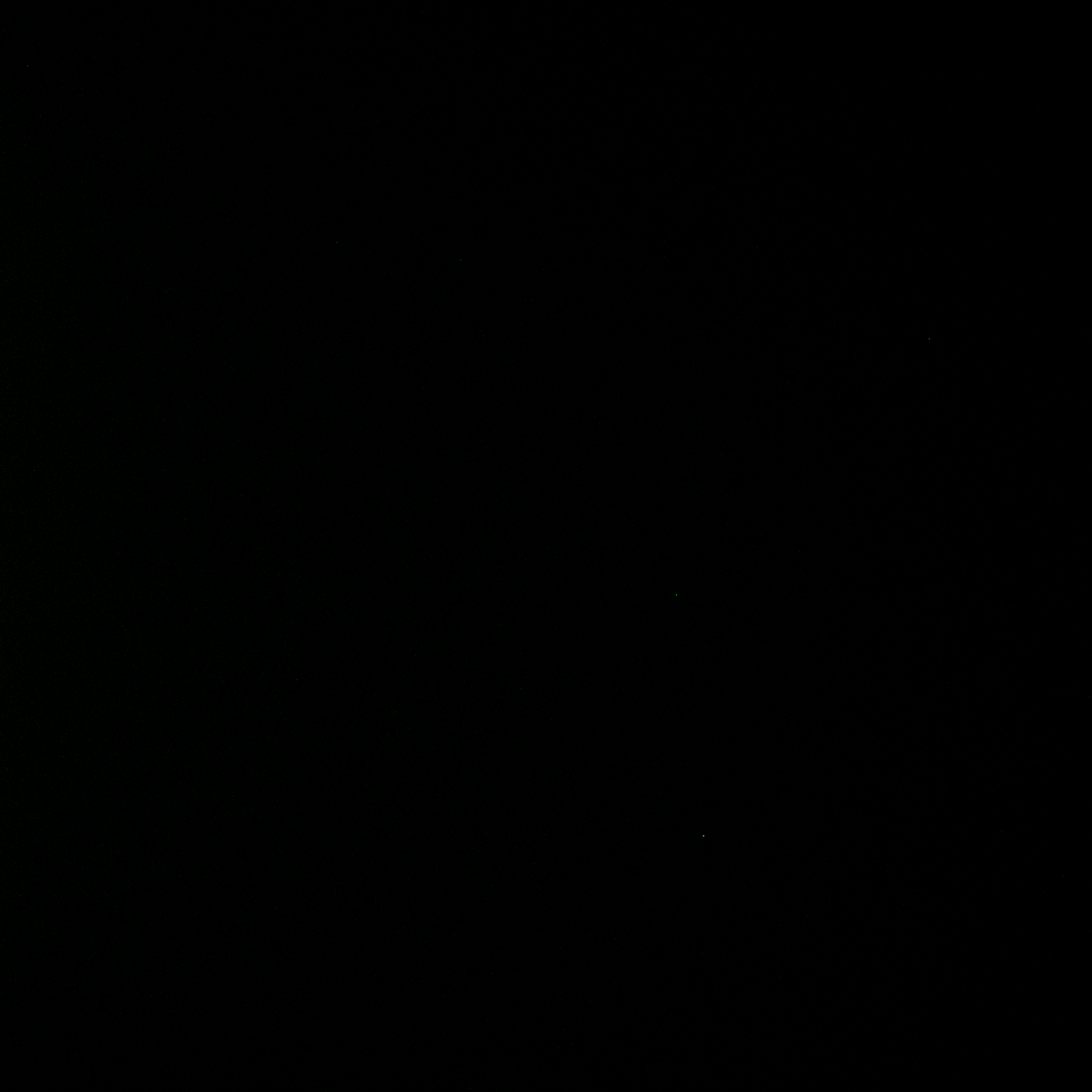

Supplement: Supplementary file 9 — Source data Fig. 3 [file 44318_2024_337_MOESM9_ESM.zip › 03_Figure_03/3G/02-TUBB4B-mNeon/Cre-Clone-miRFPnano3.tif]

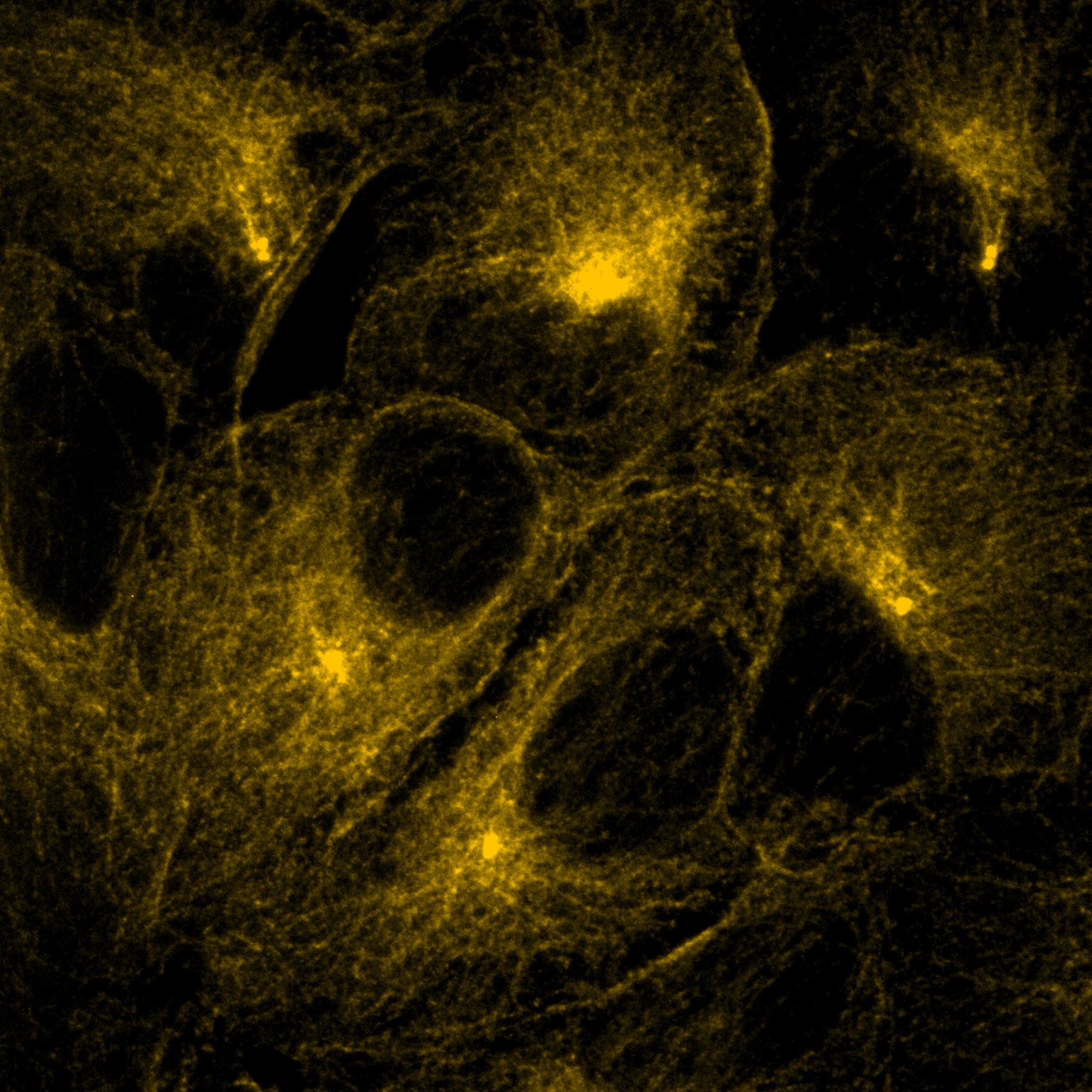

Supplement: Supplementary file 9 — Source data Fig. 3 [file 44318_2024_337_MOESM9_ESM.zip › 03_Figure_03/3G/02-TUBB4B-mNeon/Cre-Clone-mNeon.tif]

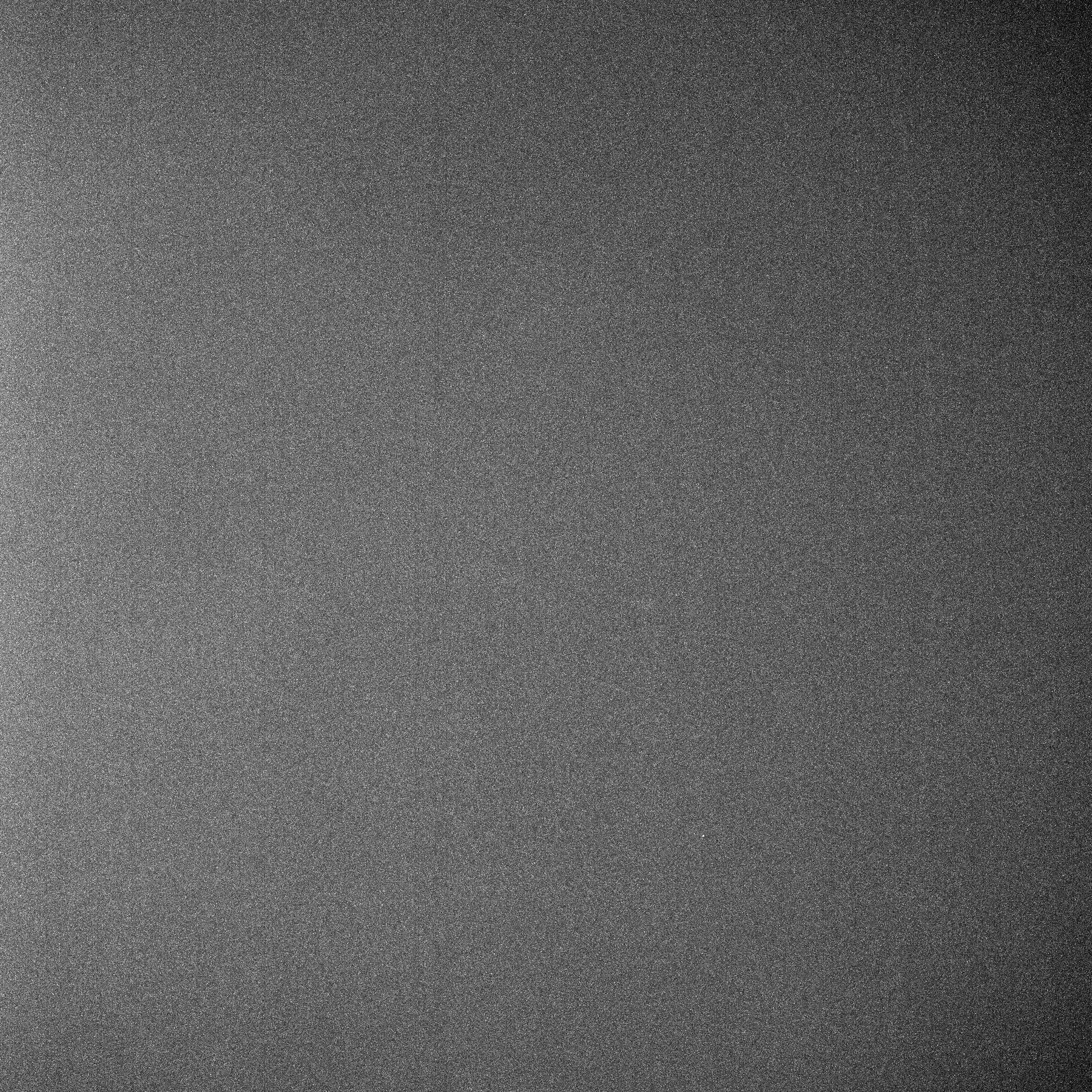

Supplement: Supplementary file 9 — Source data Fig. 3 [file 44318_2024_337_MOESM9_ESM.zip › 03_Figure_03/3G/02-TUBB4B-mNeon/_FULL-RANGE-Cre-Clone.tif]

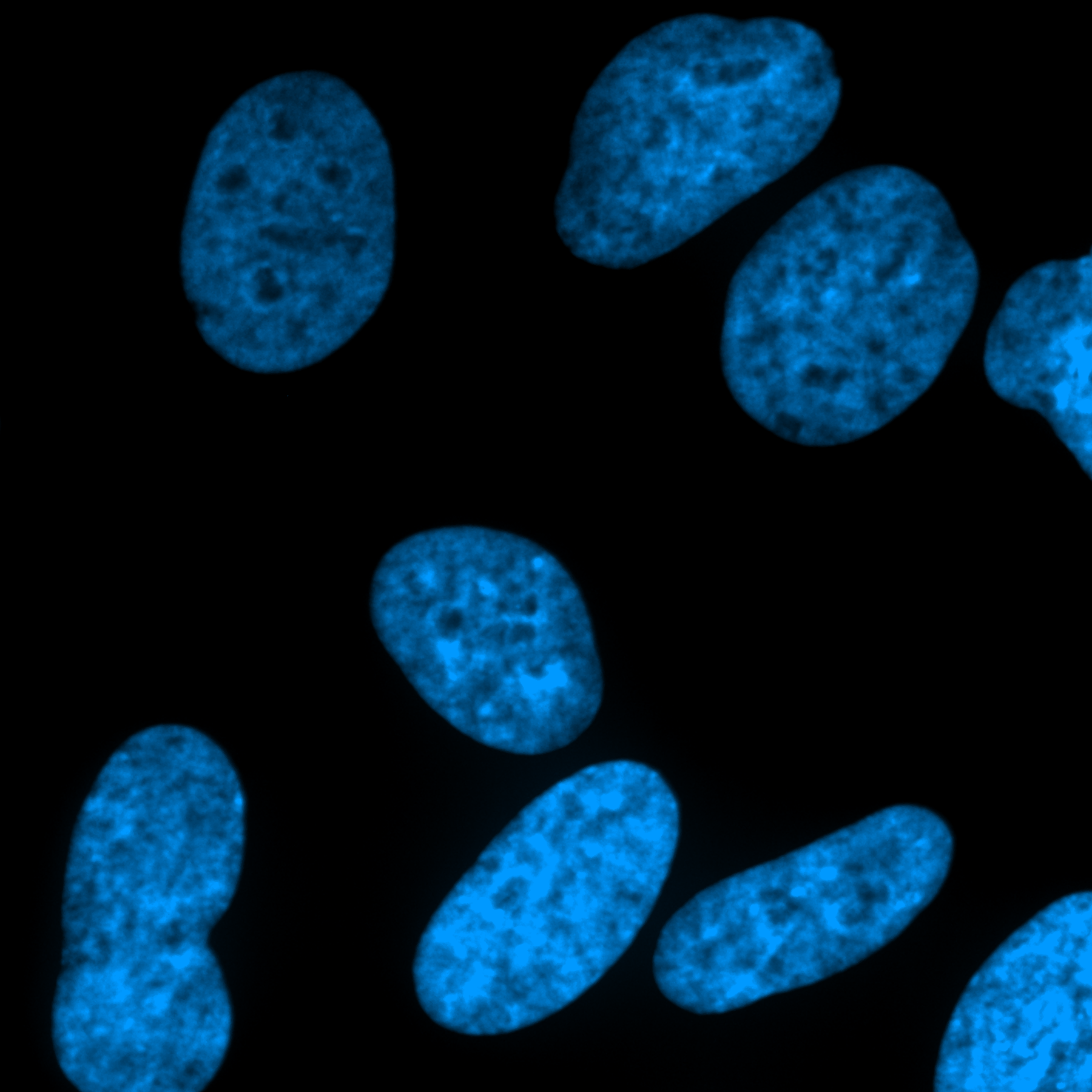

Supplement: Supplementary file 9 — Source data Fig. 3 [file 44318_2024_337_MOESM9_ESM.zip › 03_Figure_03/3G/03-TUBB4B-mNeon-H3C2-miRFPnano3/Cre-Double-DAPI.tif]

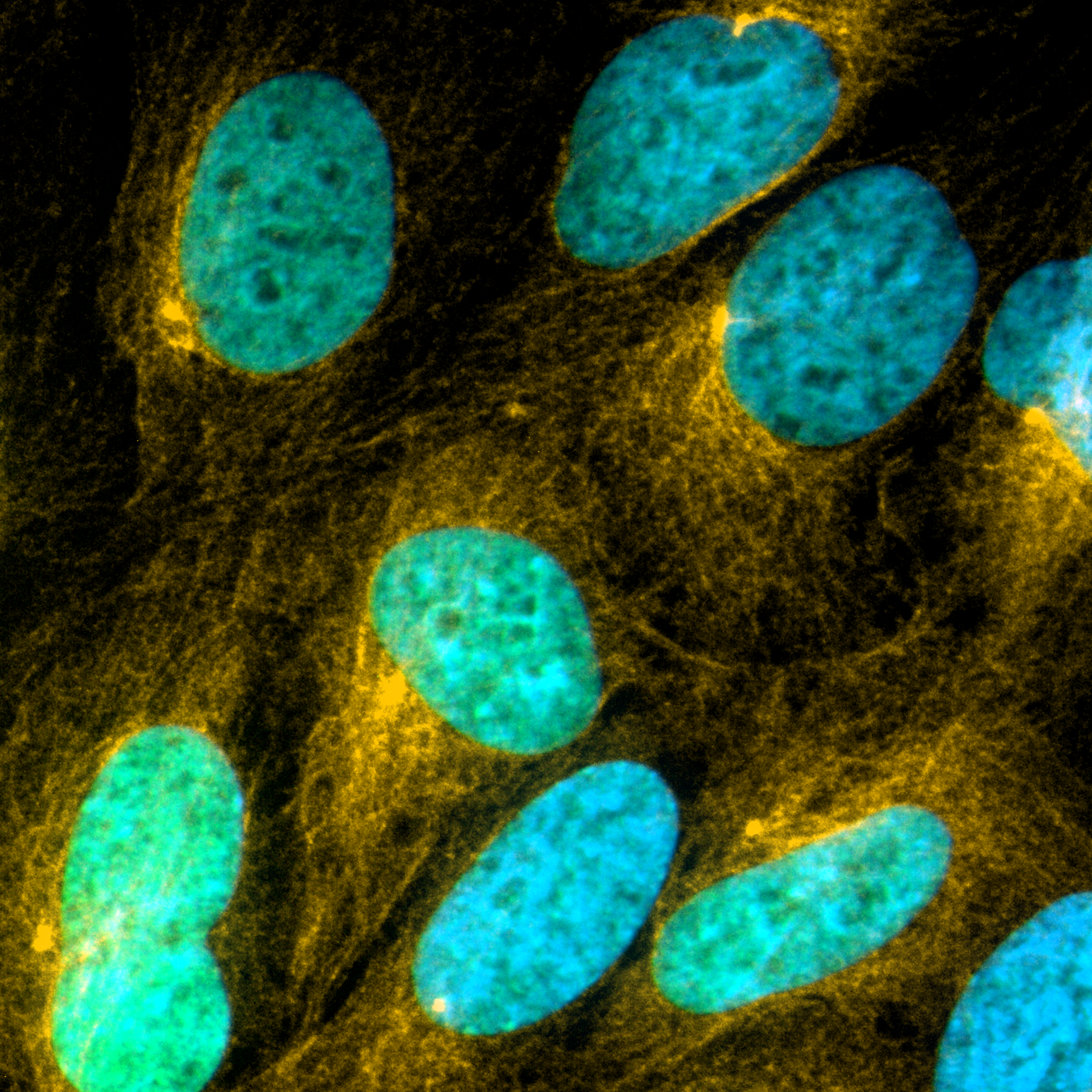

Supplement: Supplementary file 9 — Source data Fig. 3 [file 44318_2024_337_MOESM9_ESM.zip › 03_Figure_03/3G/03-TUBB4B-mNeon-H3C2-miRFPnano3/Cre-Double-Merge.tif]

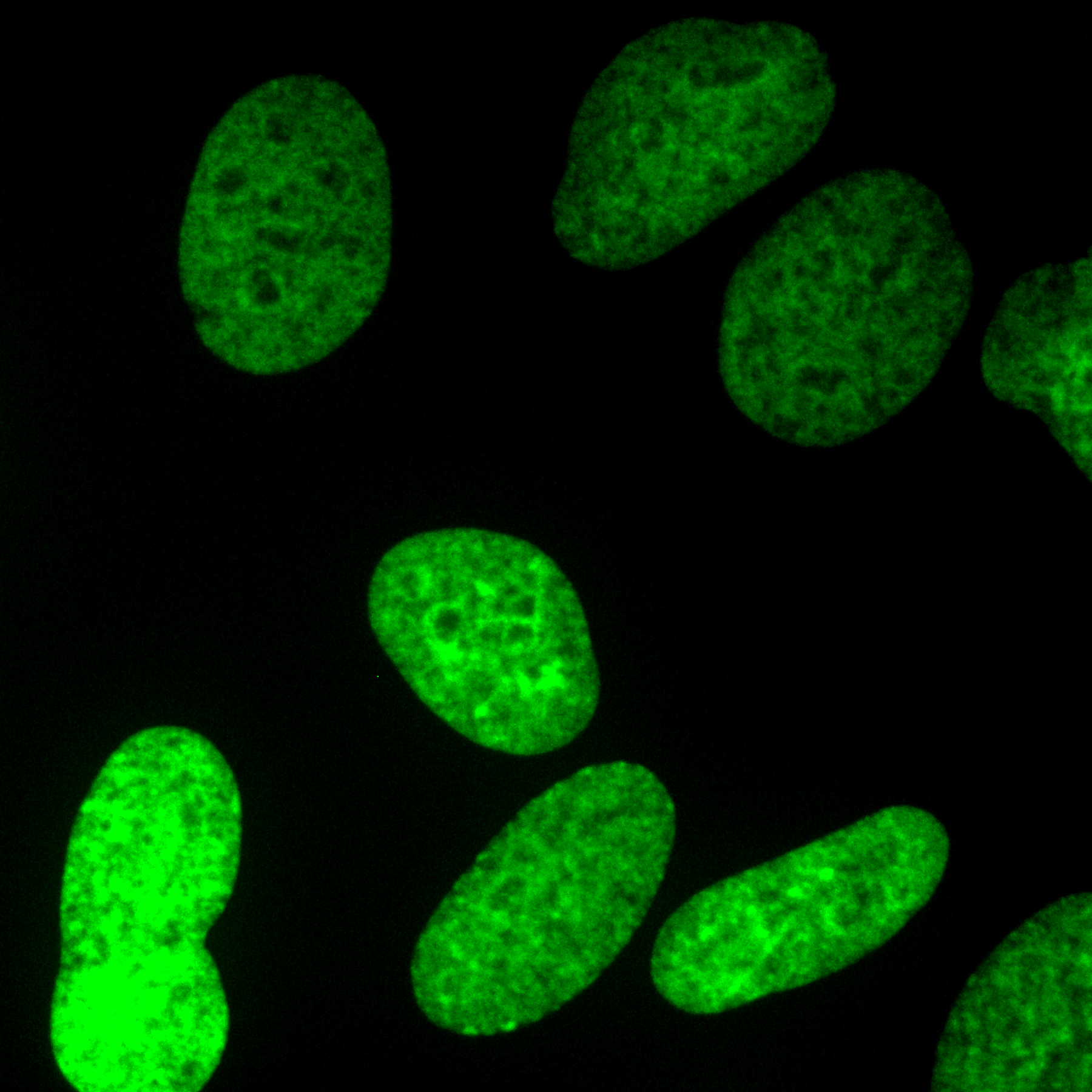

Supplement: Supplementary file 9 — Source data Fig. 3 [file 44318_2024_337_MOESM9_ESM.zip › 03_Figure_03/3G/03-TUBB4B-mNeon-H3C2-miRFPnano3/Cre-Double-miRFPnano3.tif]

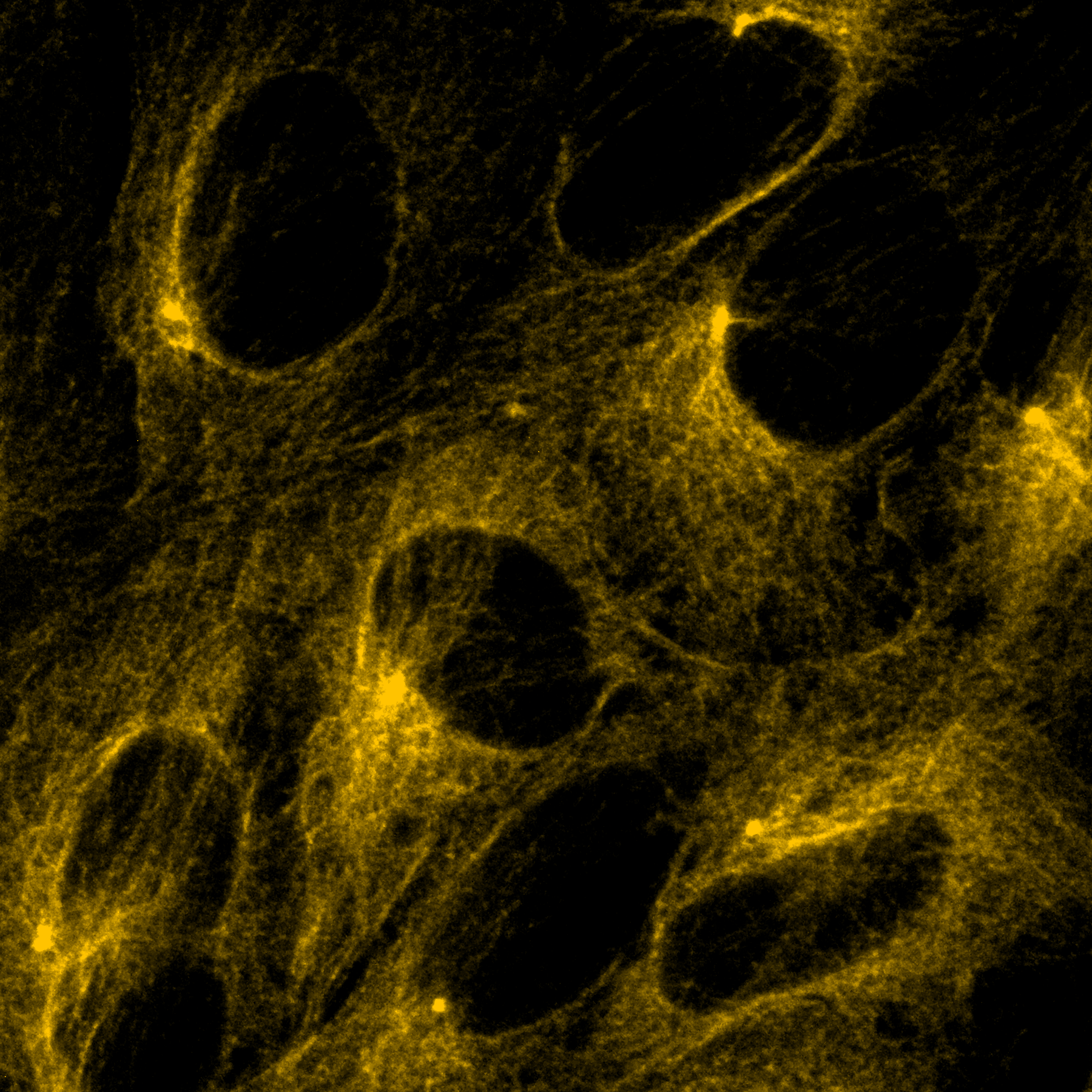

Supplement: Supplementary file 9 — Source data Fig. 3 [file 44318_2024_337_MOESM9_ESM.zip › 03_Figure_03/3G/03-TUBB4B-mNeon-H3C2-miRFPnano3/Cre-Double-mNeon.tif]

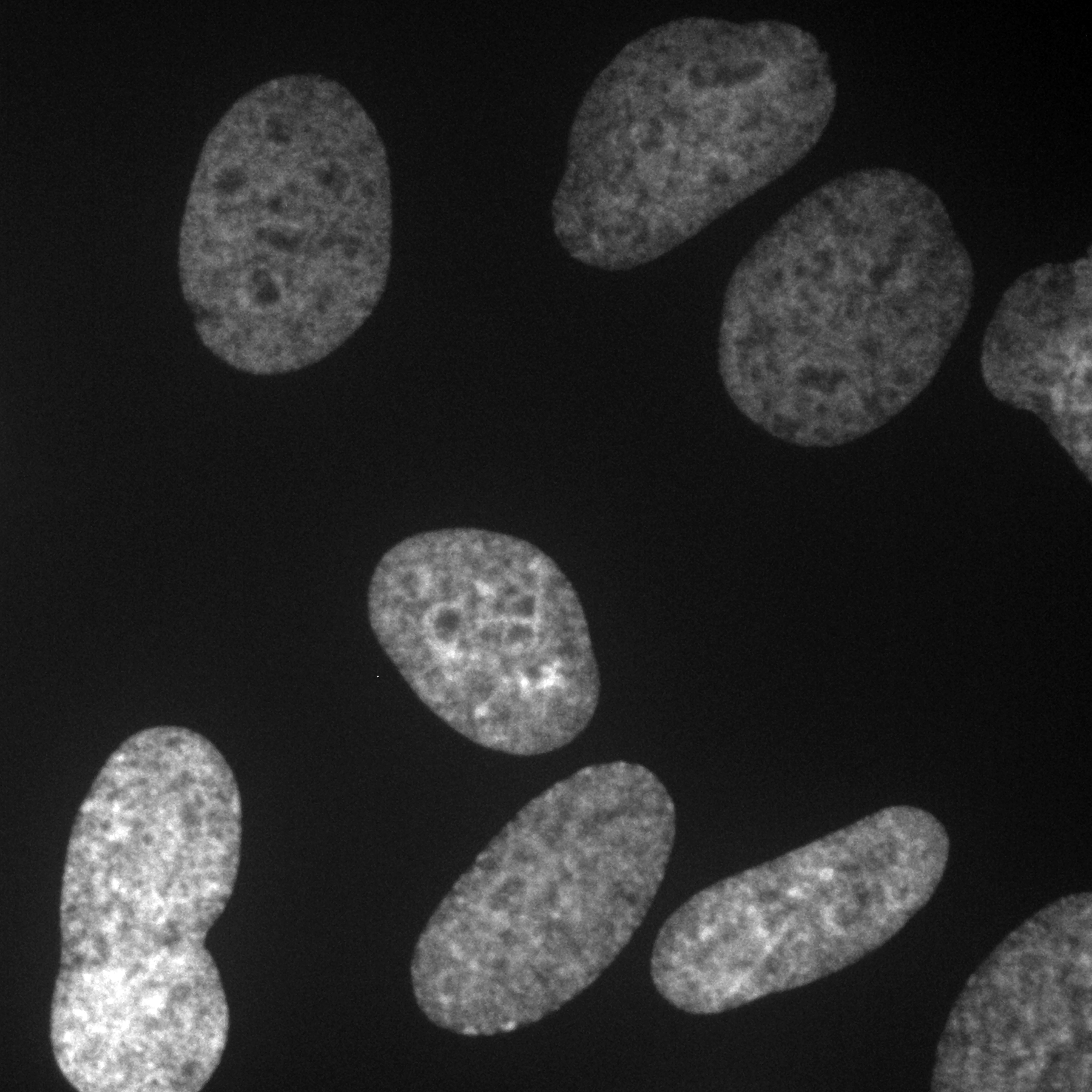

Supplement: Supplementary file 9 — Source data Fig. 3 [file 44318_2024_337_MOESM9_ESM.zip › 03_Figure_03/3G/03-TUBB4B-mNeon-H3C2-miRFPnano3/_FULL-RANGE-Double.tif]

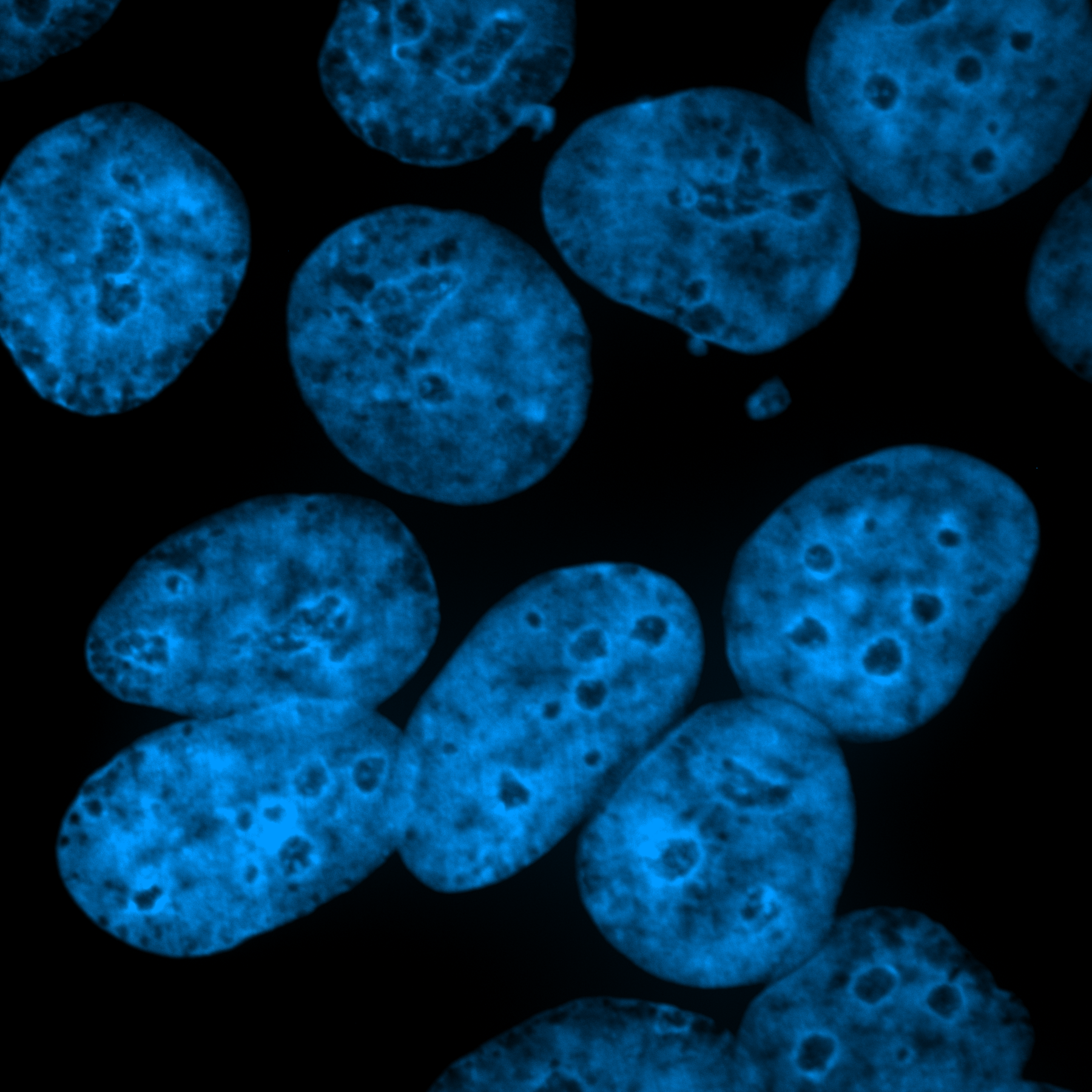

Supplement: Supplementary file 10 — Source data Fig. 4 [file 44318_2024_337_MOESM10_ESM.zip › 04_Figure_04/4C/60min-miniTurbo-STREP(+)/60min-miniTurbo-STREP(+)-DAPI.tif]

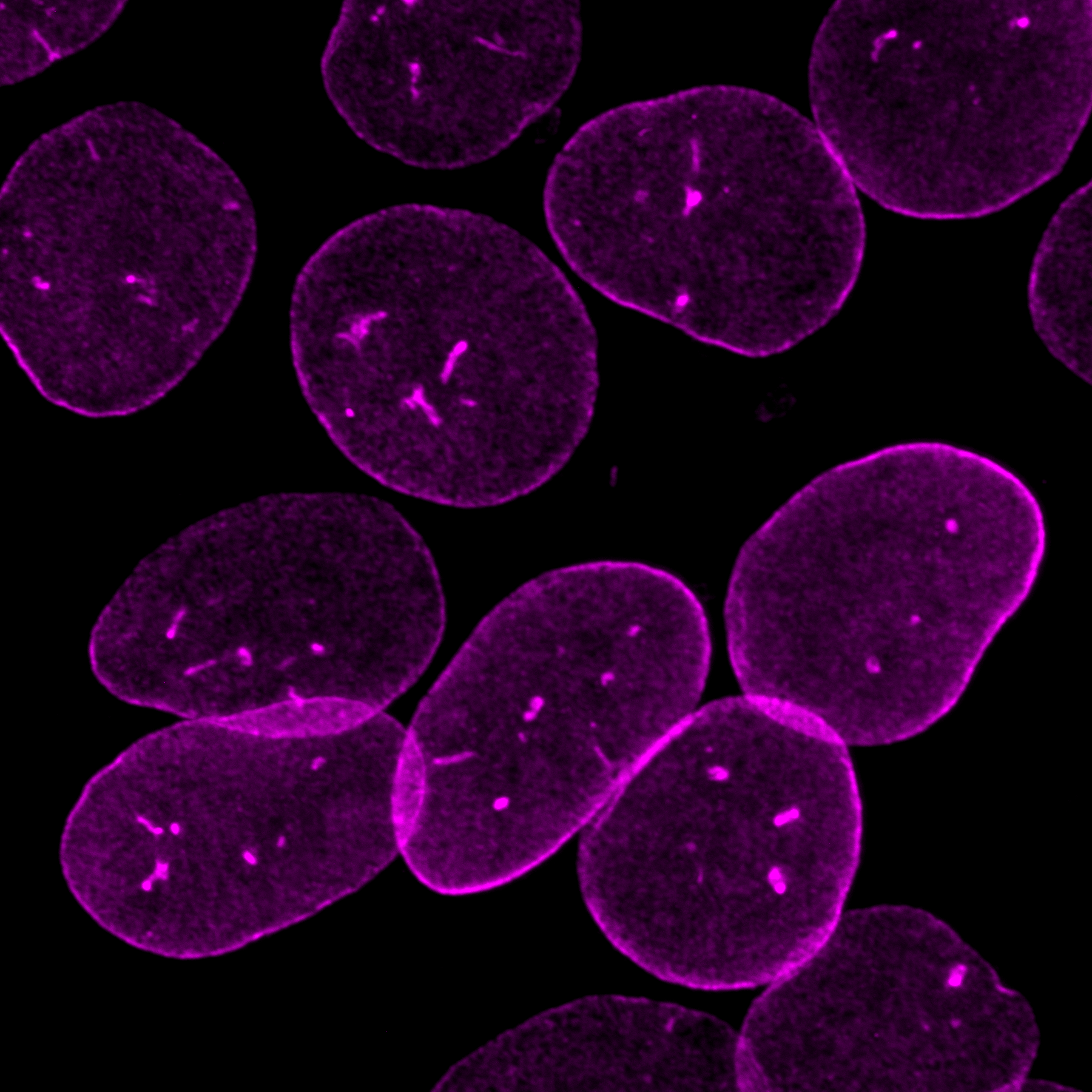

Supplement: Supplementary file 10 — Source data Fig. 4 [file 44318_2024_337_MOESM10_ESM.zip › 04_Figure_04/4C/60min-miniTurbo-STREP(+)/60min-miniTurbo-STREP(+)-LMNB1.tif]

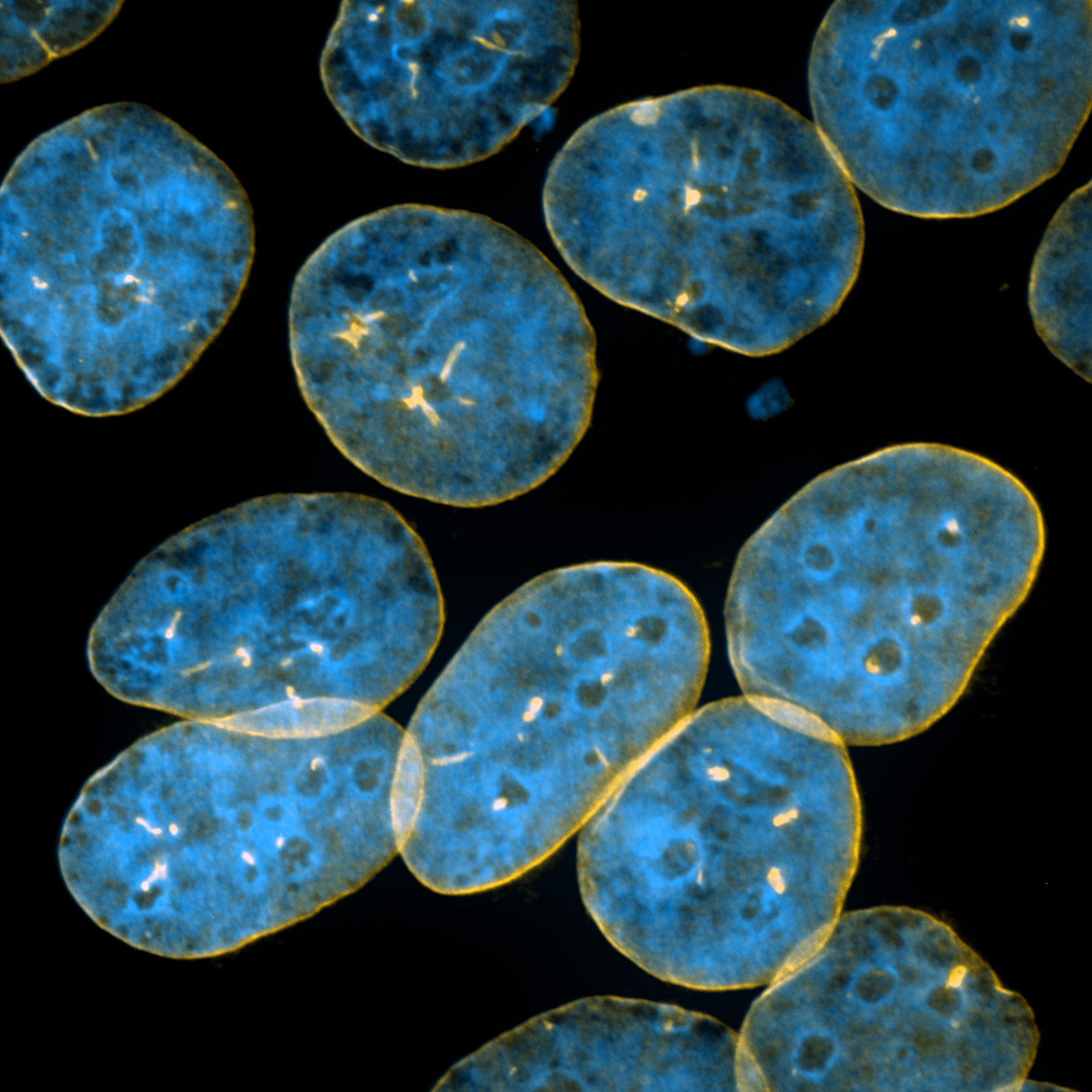

Supplement: Supplementary file 10 — Source data Fig. 4 [file 44318_2024_337_MOESM10_ESM.zip › 04_Figure_04/4C/60min-miniTurbo-STREP(+)/60min-miniTurbo-STREP(+)-Merge.tif]

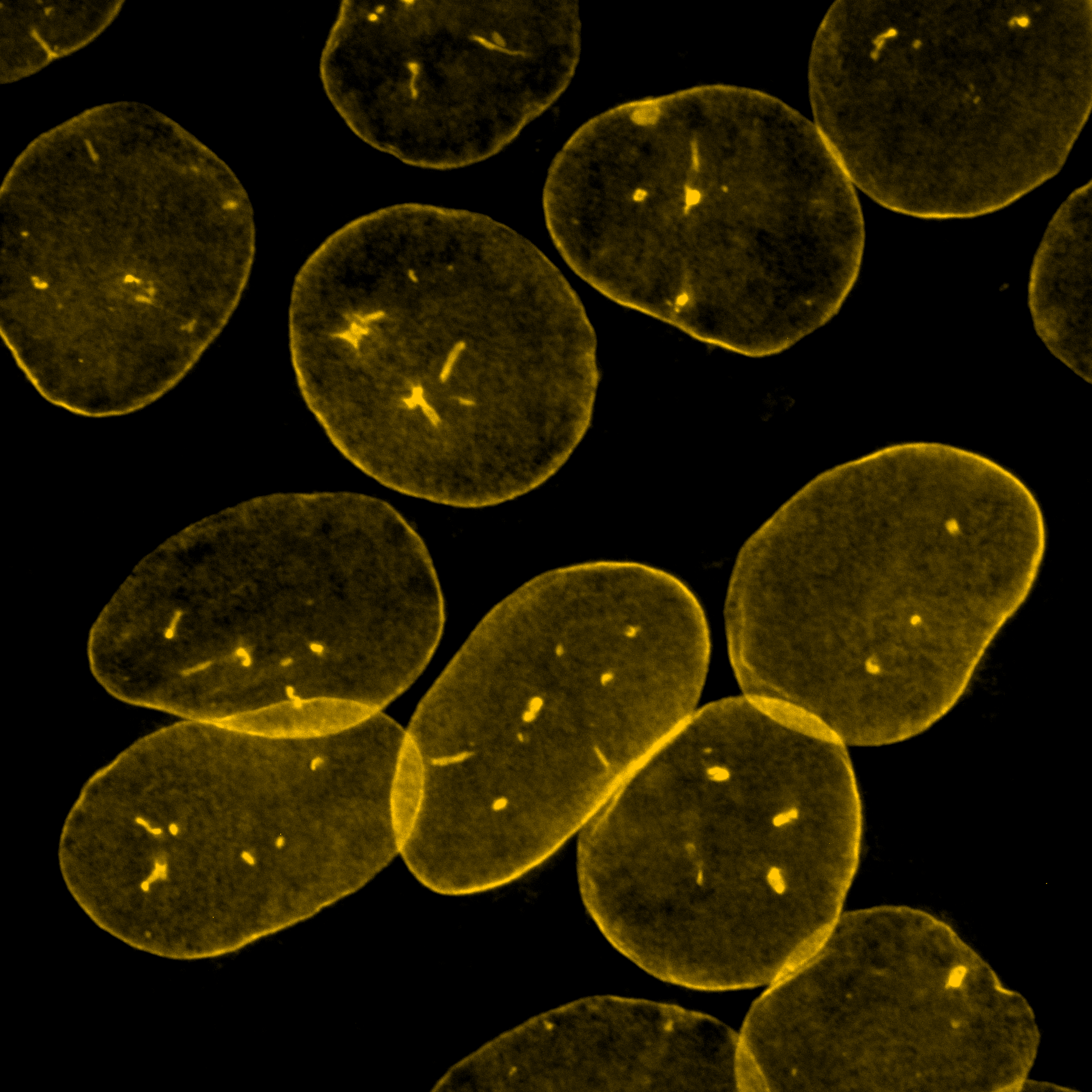

Supplement: Supplementary file 10 — Source data Fig. 4 [file 44318_2024_337_MOESM10_ESM.zip › 04_Figure_04/4C/60min-miniTurbo-STREP(+)/60min-miniTurbo-STREP(+)-Streptavidin.tif]

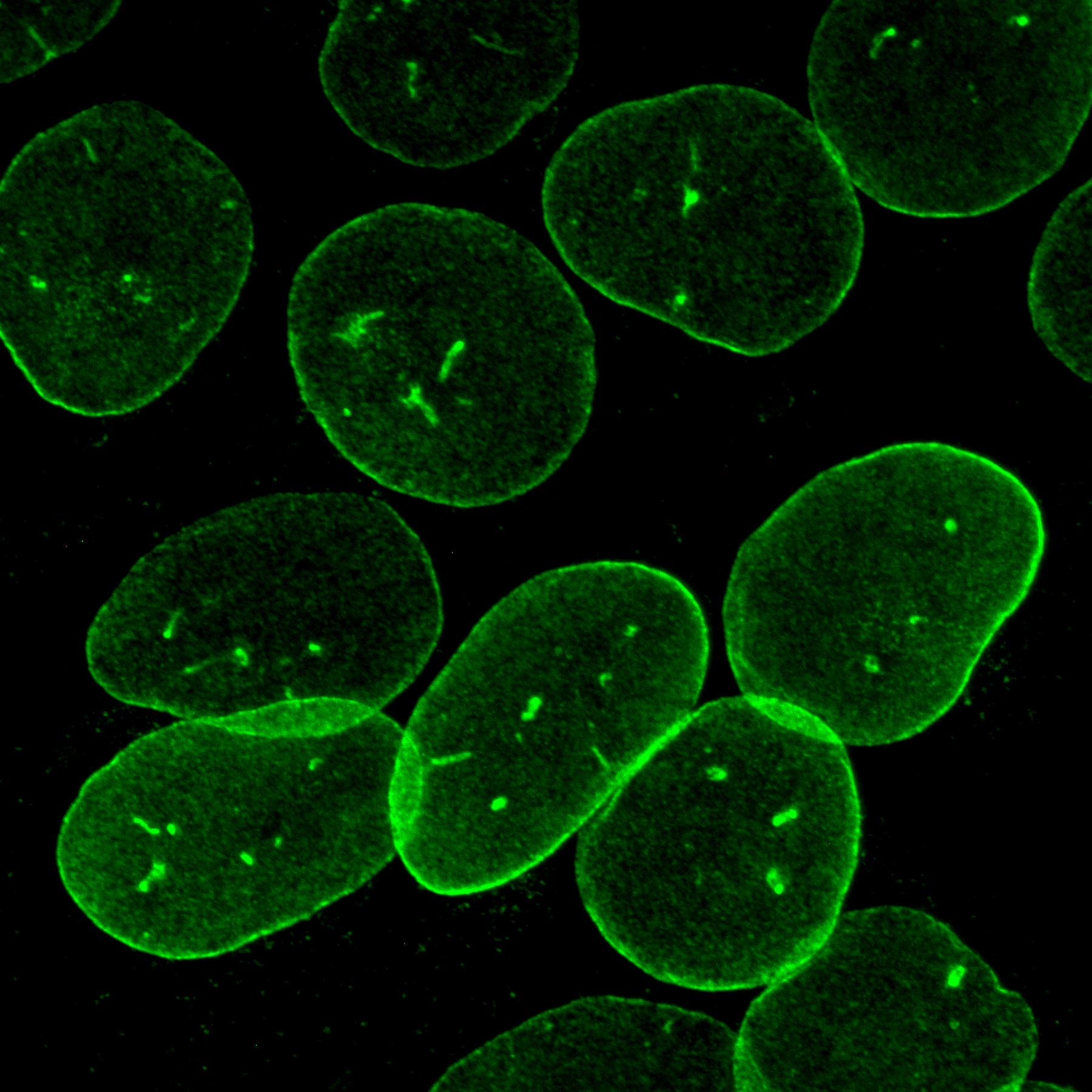

Supplement: Supplementary file 10 — Source data Fig. 4 [file 44318_2024_337_MOESM10_ESM.zip › 04_Figure_04/4C/60min-miniTurbo-STREP(+)/60min-miniTurbo-STREP(+)-V5.tif]

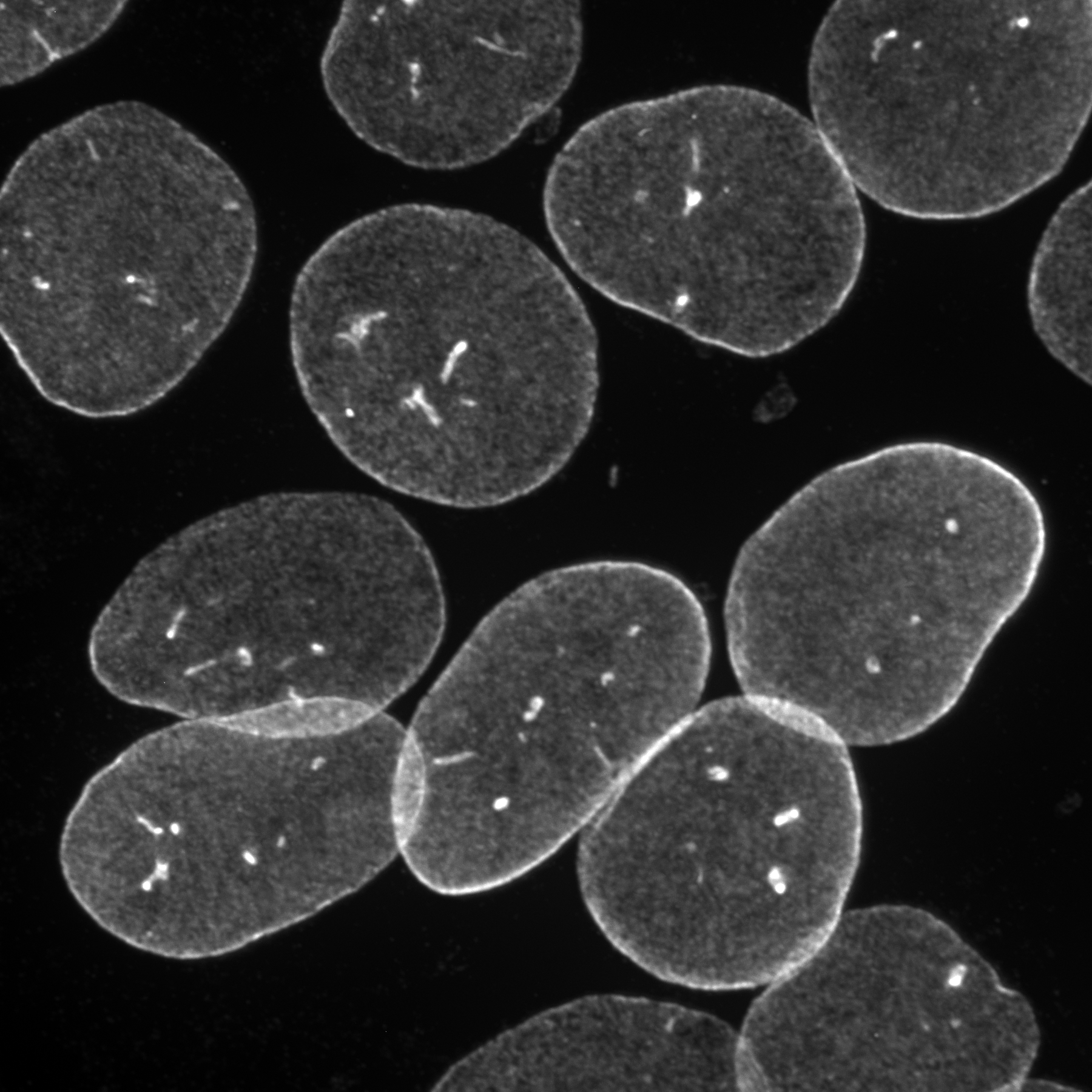

Supplement: Supplementary file 10 — Source data Fig. 4 [file 44318_2024_337_MOESM10_ESM.zip › 04_Figure_04/4C/60min-miniTurbo-STREP(+)/_FULL-RANGE-60min-miniTurbo-STREP(+).tif]

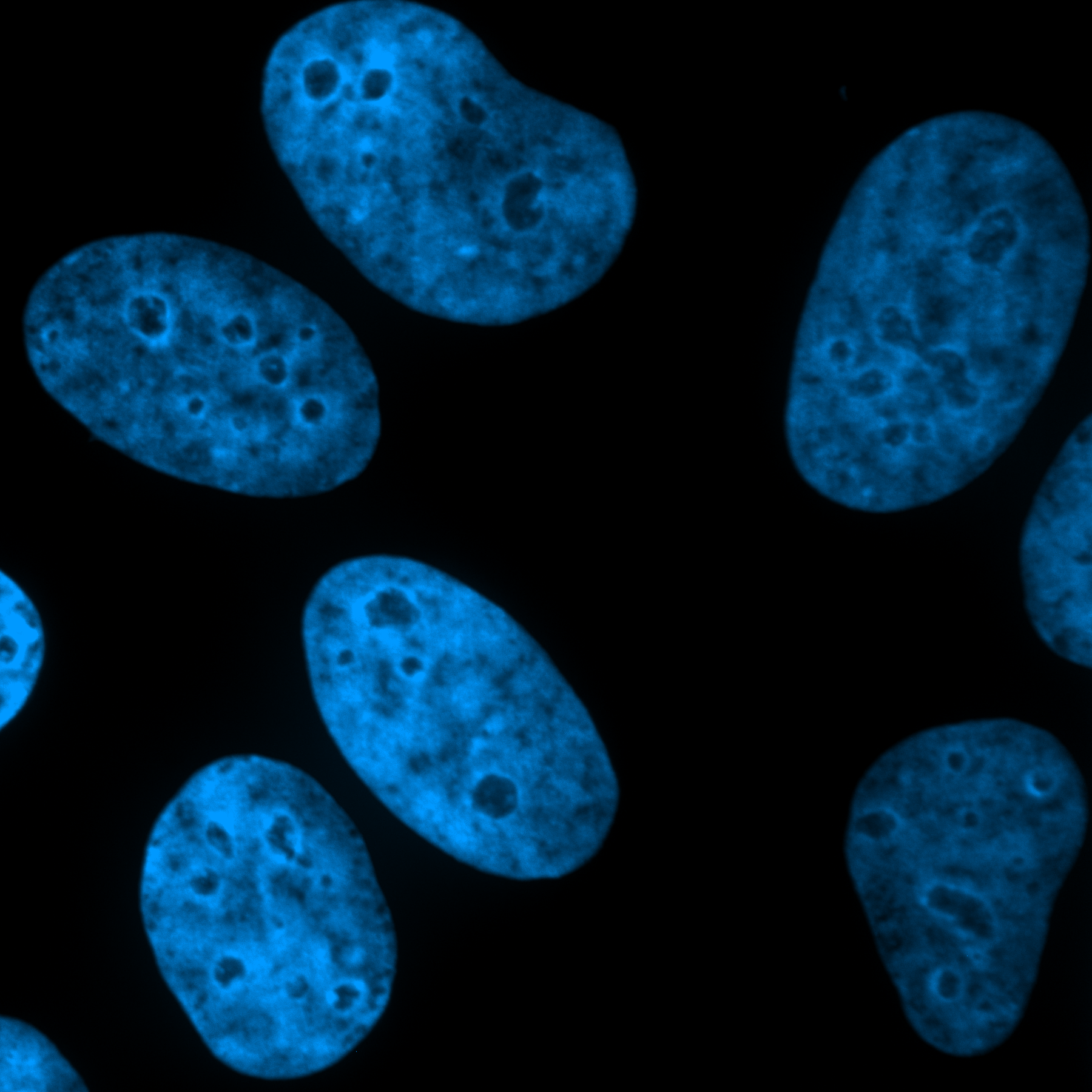

Supplement: Supplementary file 10 — Source data Fig. 4 [file 44318_2024_337_MOESM10_ESM.zip › 04_Figure_04/4C/60min-miniTurbo-STREP(-)/60min-miniTurbo-STREP(-)-DAPI.tif]

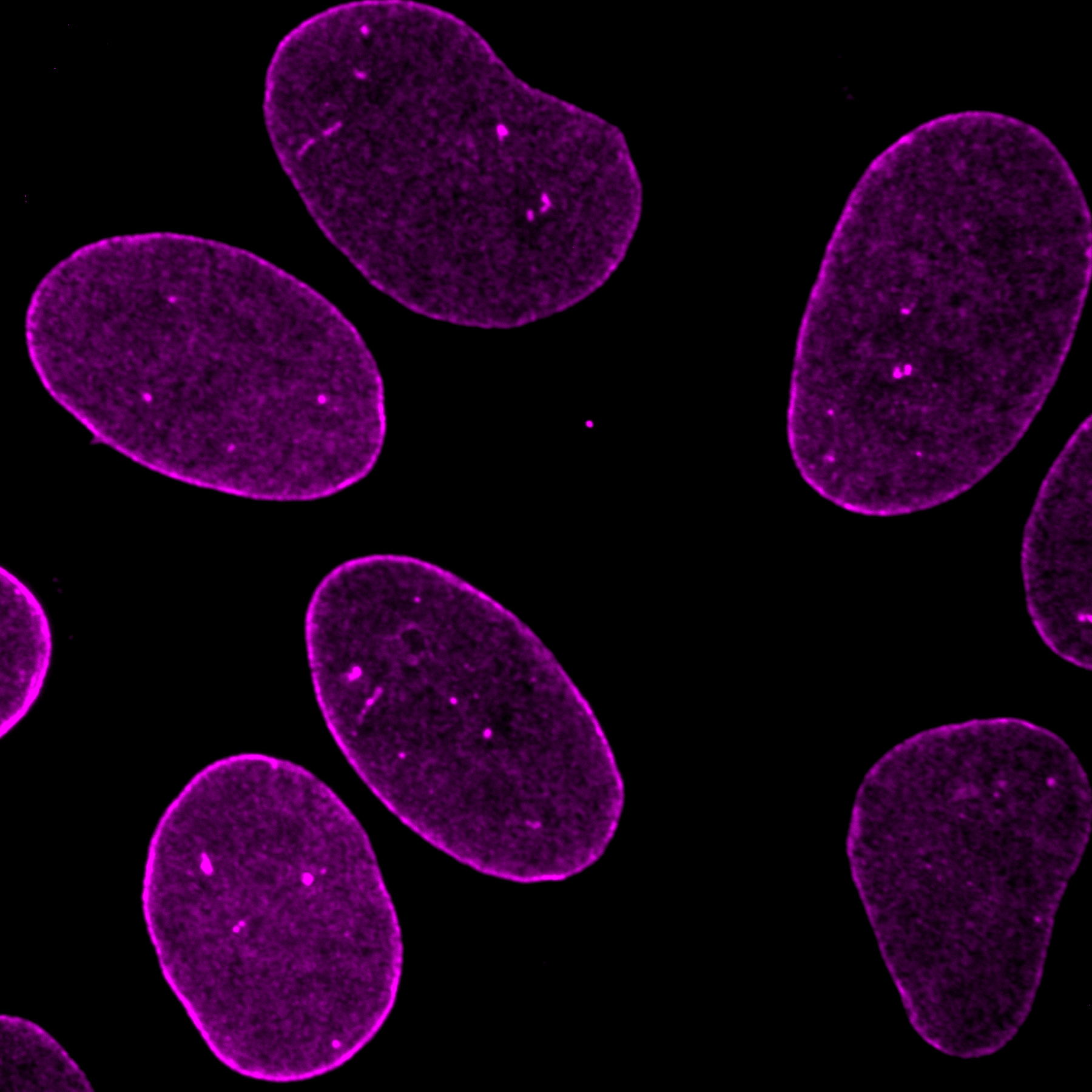

Supplement: Supplementary file 10 — Source data Fig. 4 [file 44318_2024_337_MOESM10_ESM.zip › 04_Figure_04/4C/60min-miniTurbo-STREP(-)/60min-miniTurbo-STREP(-)-LMNB1.tif]

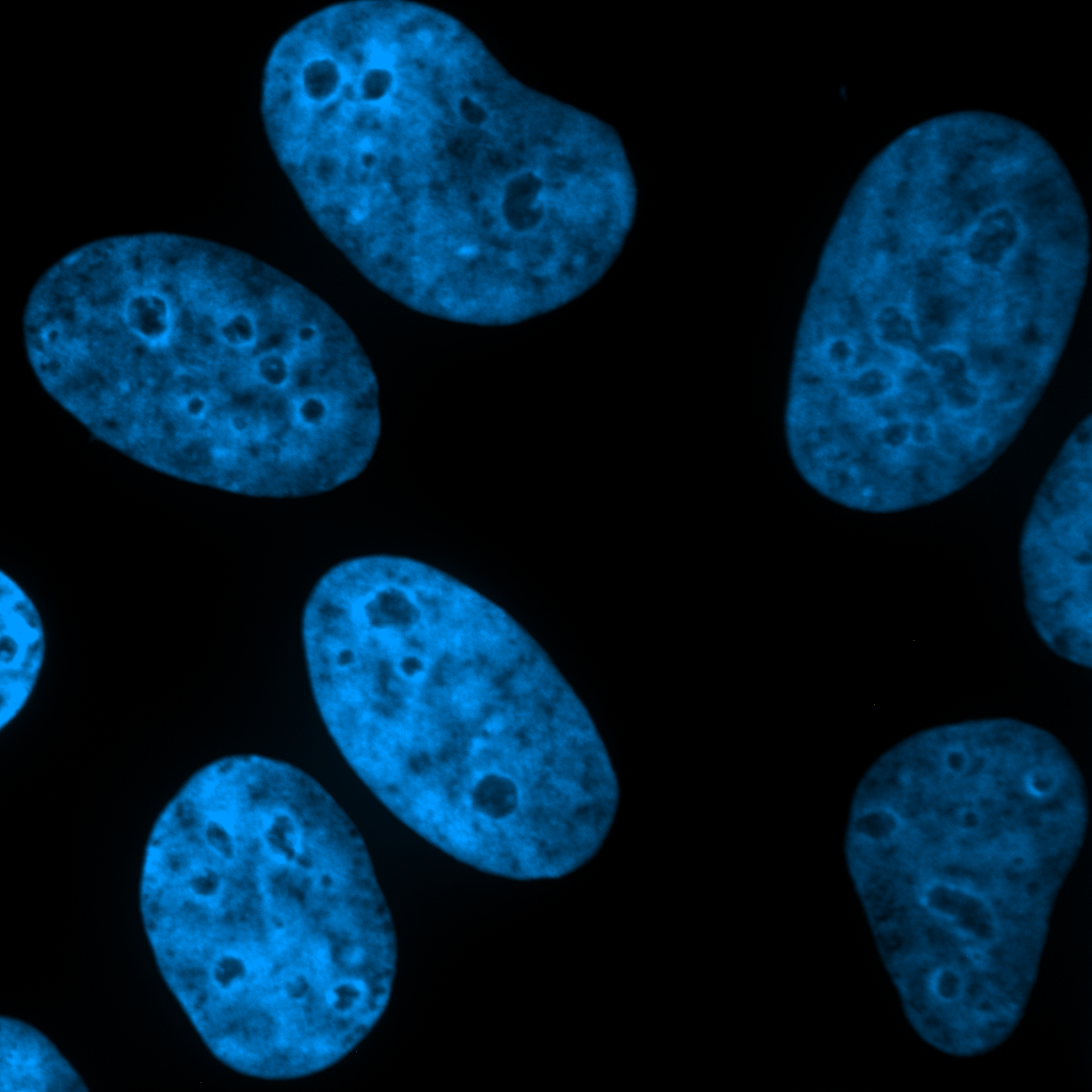

Supplement: Supplementary file 10 — Source data Fig. 4 [file 44318_2024_337_MOESM10_ESM.zip › 04_Figure_04/4C/60min-miniTurbo-STREP(-)/60min-miniTurbo-STREP(-)-MergeI.tif]

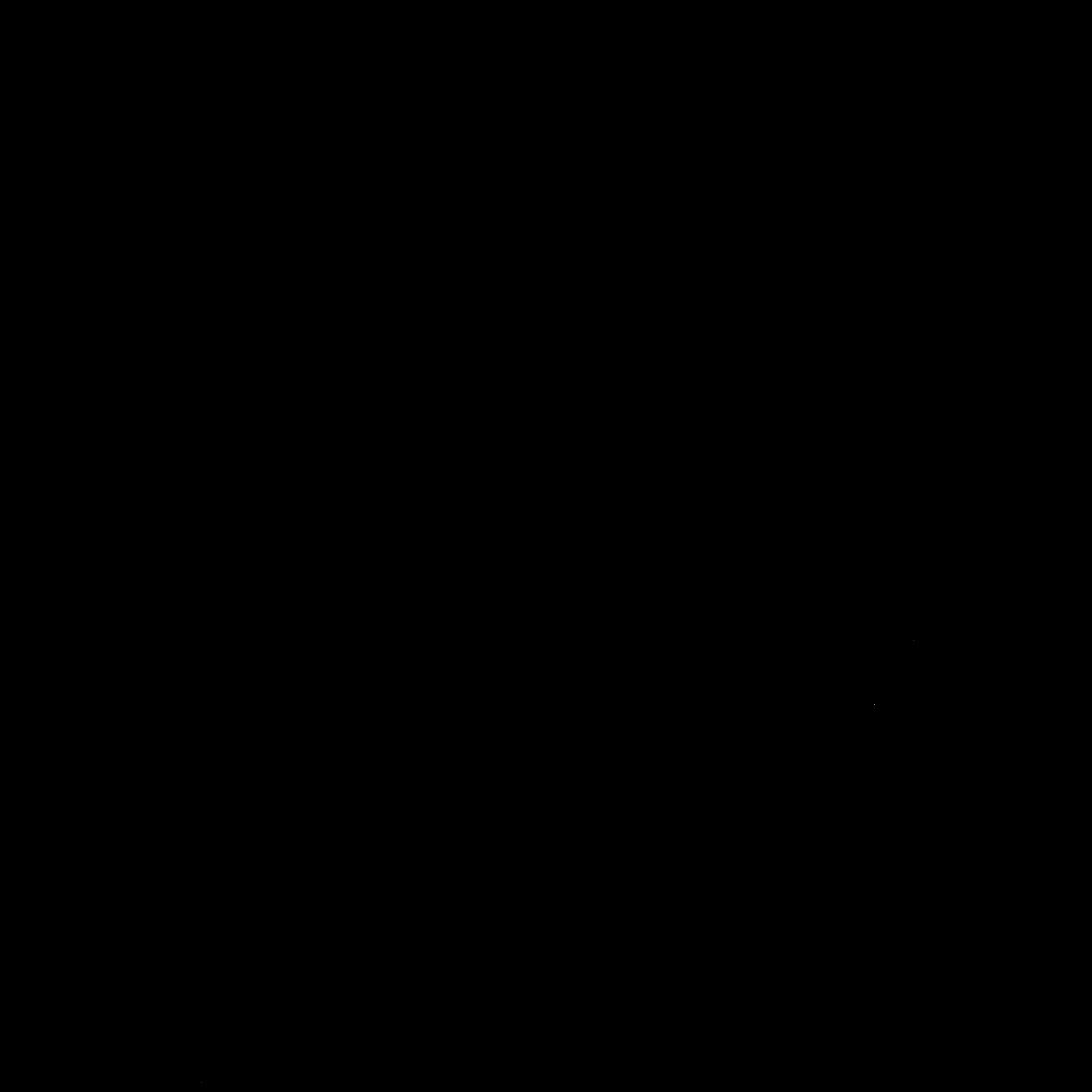

Supplement: Supplementary file 10 — Source data Fig. 4 [file 44318_2024_337_MOESM10_ESM.zip › 04_Figure_04/4C/60min-miniTurbo-STREP(-)/60min-miniTurbo-STREP(-)-Streptavidin.tif]

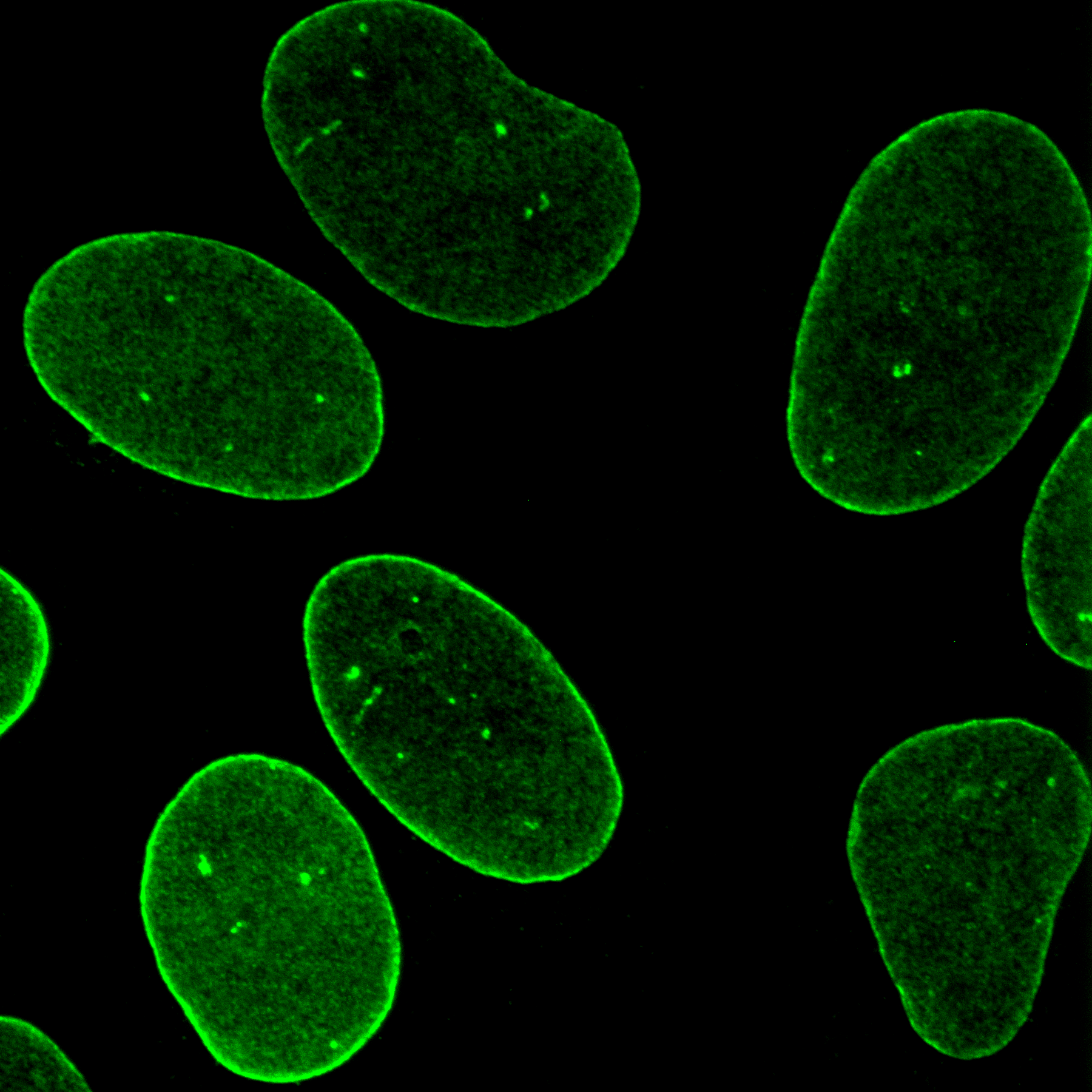

Supplement: Supplementary file 10 — Source data Fig. 4 [file 44318_2024_337_MOESM10_ESM.zip › 04_Figure_04/4C/60min-miniTurbo-STREP(-)/60min-miniTurbo-STREP(-)-V5.tif]

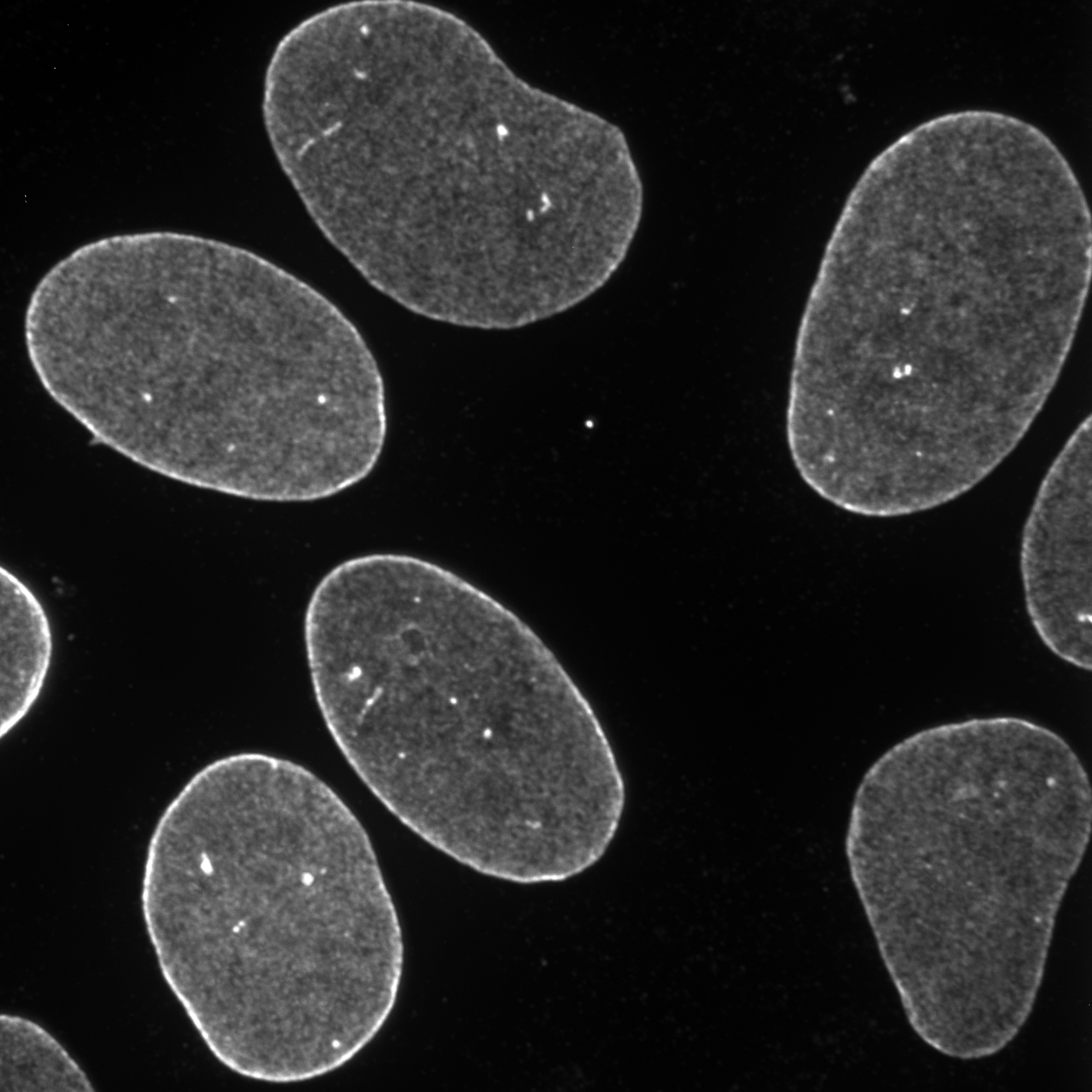

Supplement: Supplementary file 10 — Source data Fig. 4 [file 44318_2024_337_MOESM10_ESM.zip › 04_Figure_04/4C/60min-miniTurbo-STREP(-)/_FULL-RANGE-60min-miniTurbo-STREP(-).tif]

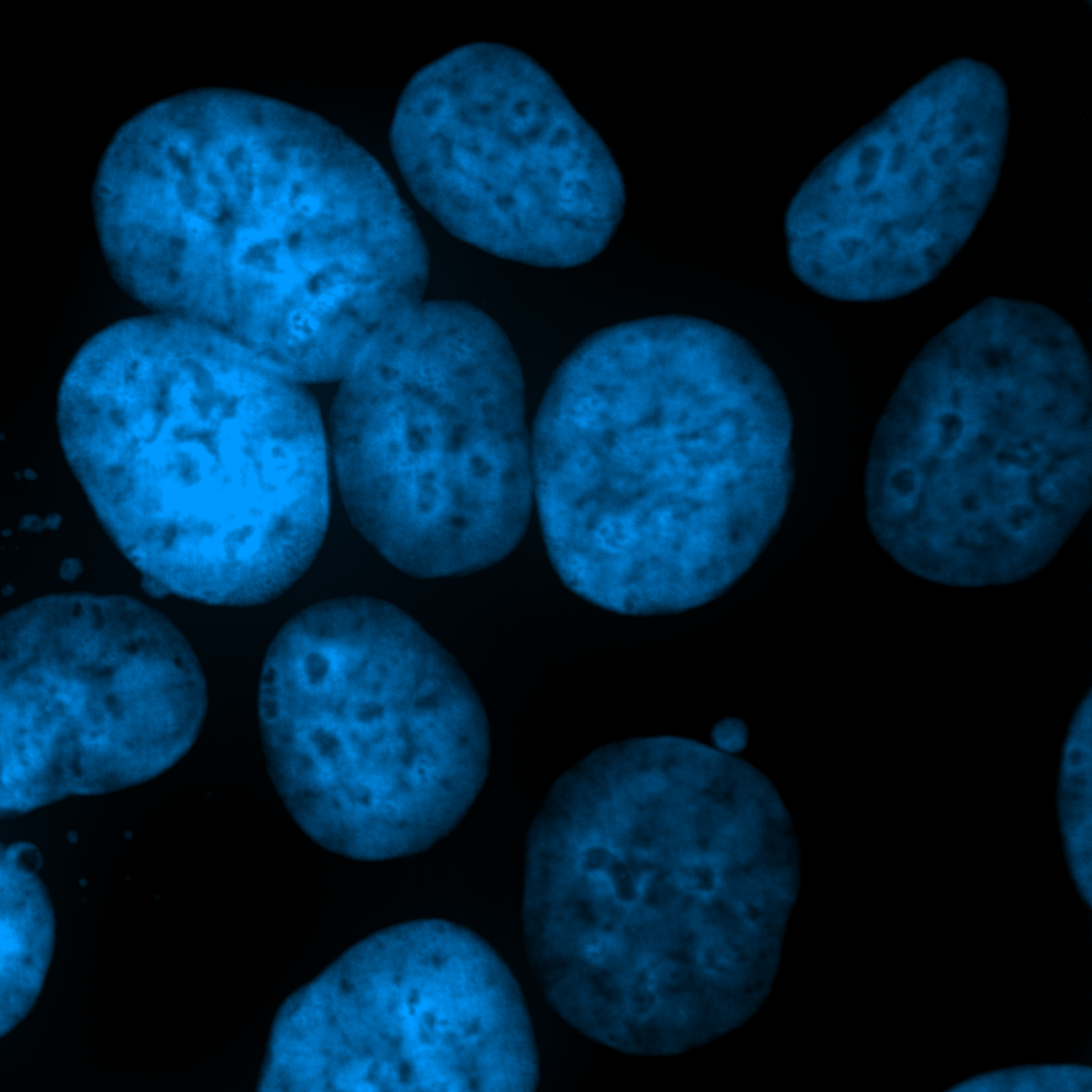

Supplement: Supplementary file 10 — Source data Fig. 4 [file 44318_2024_337_MOESM10_ESM.zip › 04_Figure_04/4C/60min-ultraID-STREP(+)/60min-ultraID-STREP(+)-DAPI.tif]

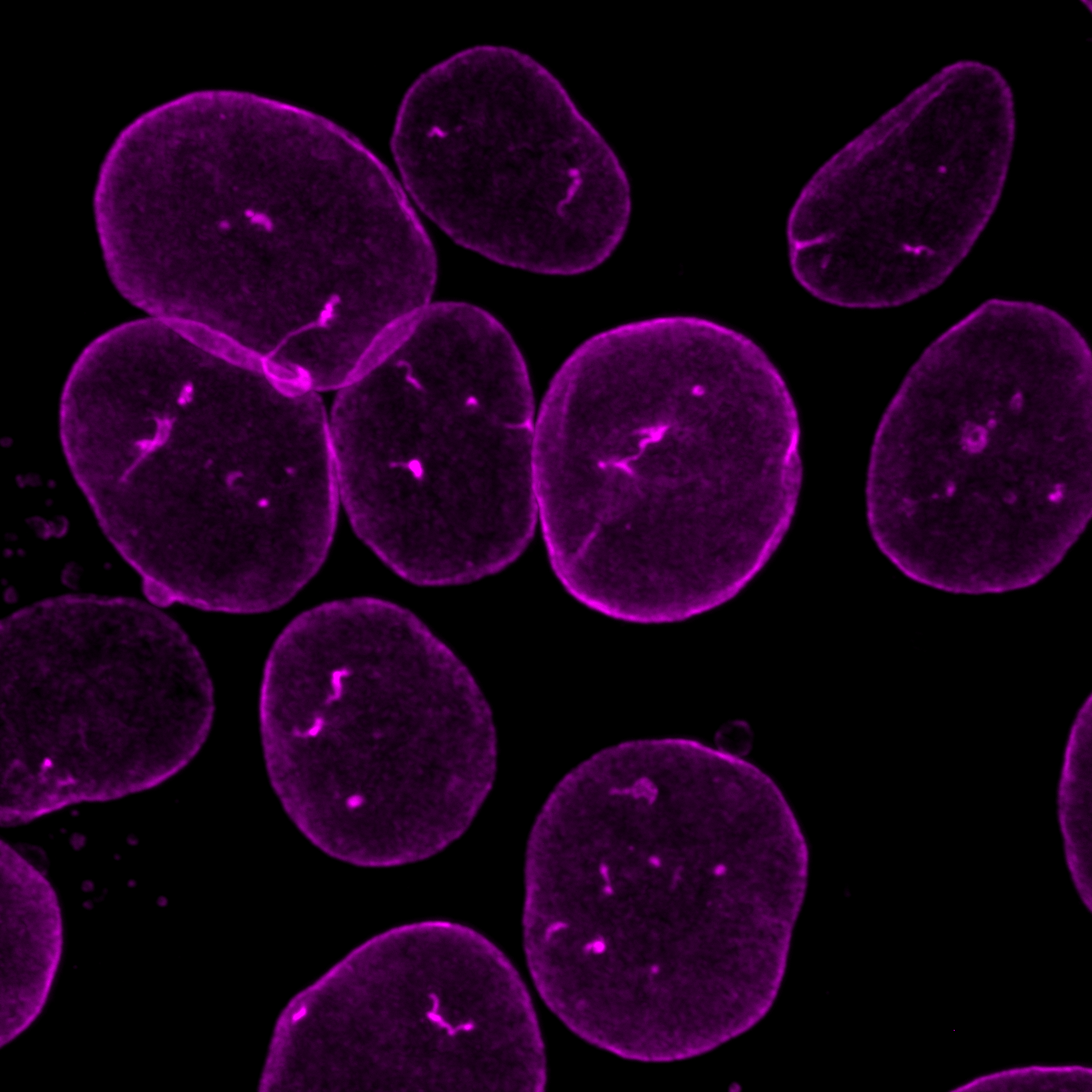

Supplement: Supplementary file 10 — Source data Fig. 4 [file 44318_2024_337_MOESM10_ESM.zip › 04_Figure_04/4C/60min-ultraID-STREP(+)/60min-ultraID-STREP(+)-LMNB1.tif]

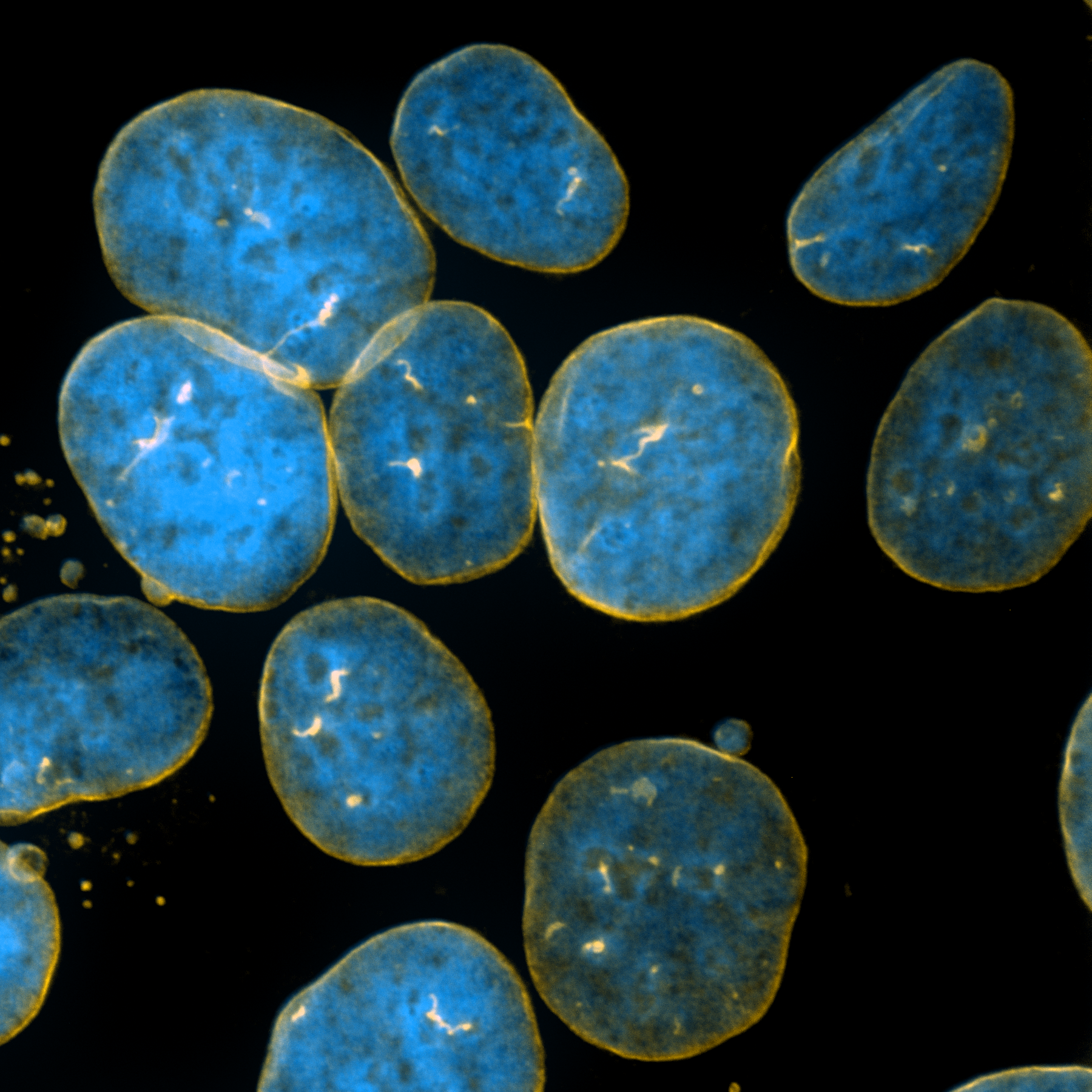

Supplement: Supplementary file 10 — Source data Fig. 4 [file 44318_2024_337_MOESM10_ESM.zip › 04_Figure_04/4C/60min-ultraID-STREP(+)/60min-ultraID-STREP(+)-Merge.tif]

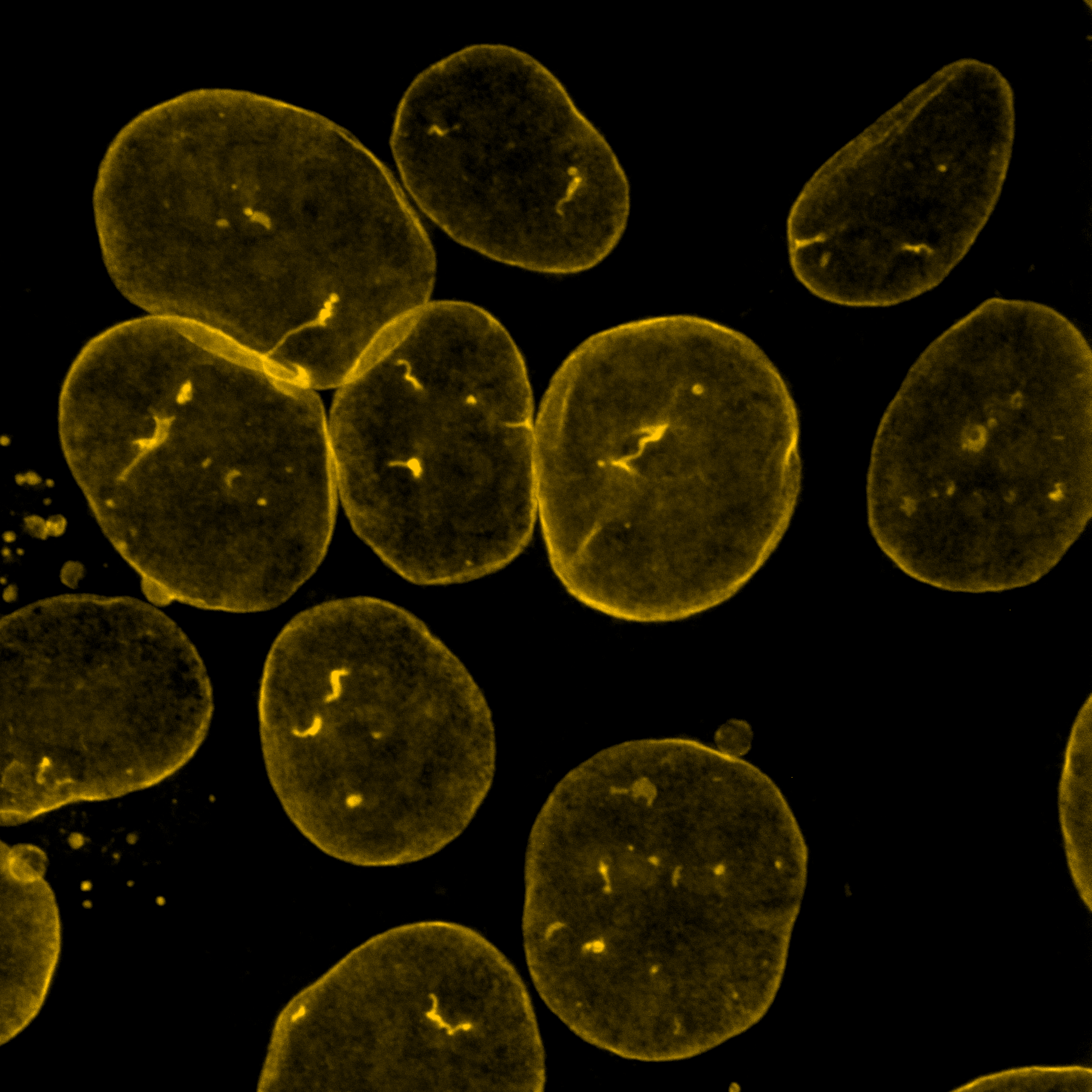

Supplement: Supplementary file 10 — Source data Fig. 4 [file 44318_2024_337_MOESM10_ESM.zip › 04_Figure_04/4C/60min-ultraID-STREP(+)/60min-ultraID-STREP(+)-Streptavidin.tif]

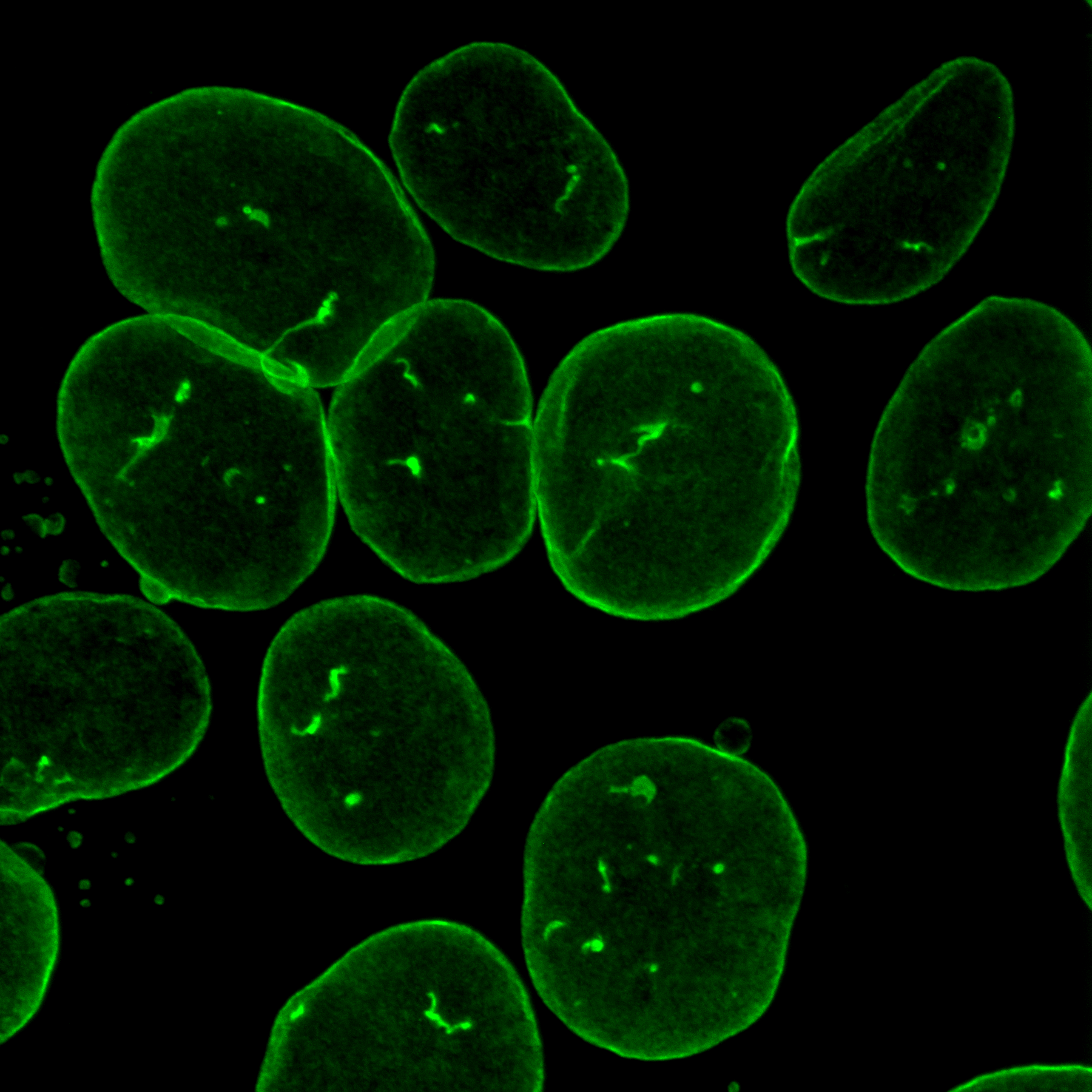

Supplement: Supplementary file 10 — Source data Fig. 4 [file 44318_2024_337_MOESM10_ESM.zip › 04_Figure_04/4C/60min-ultraID-STREP(+)/60min-ultraID-STREP(+)-V5.tif]

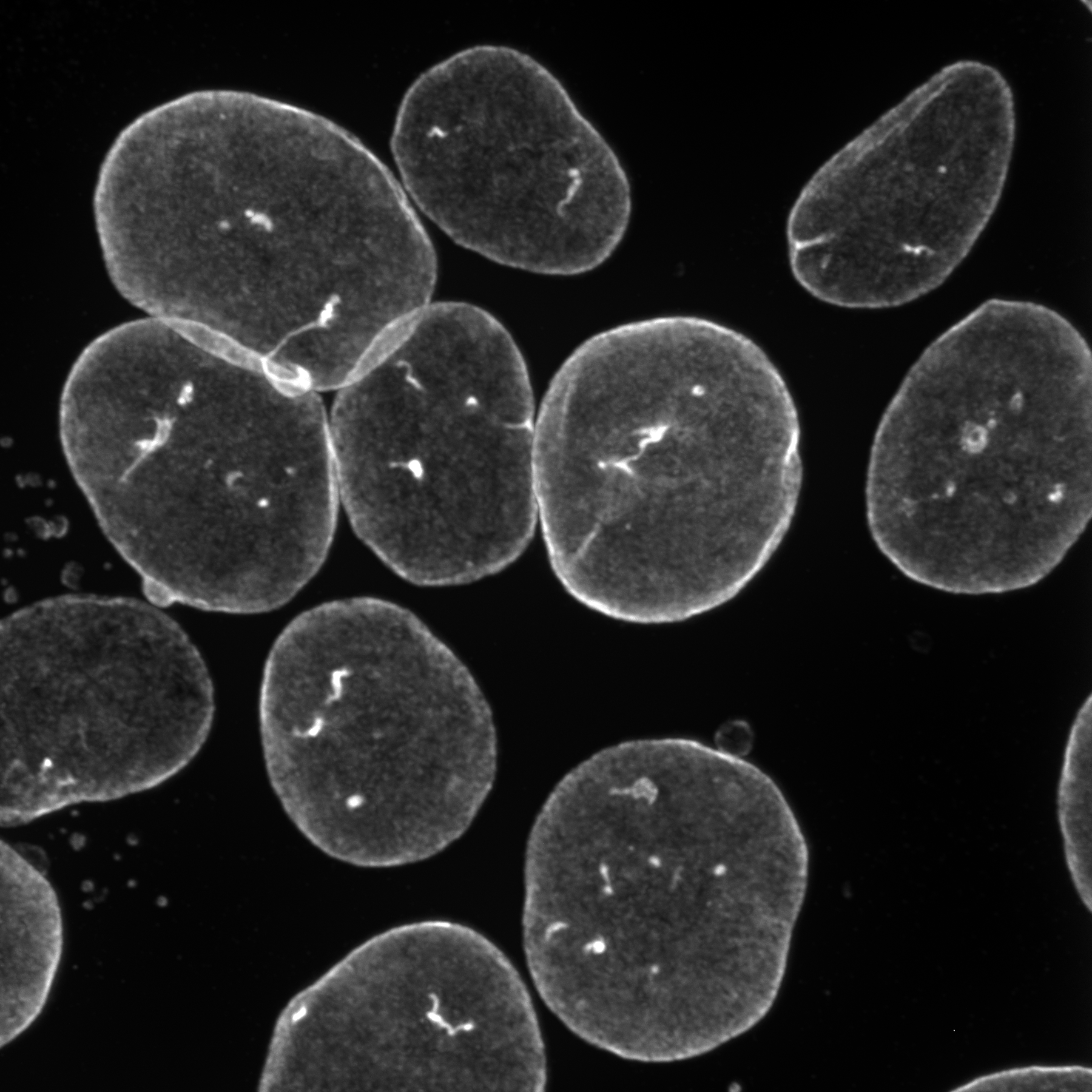

Supplement: Supplementary file 10 — Source data Fig. 4 [file 44318_2024_337_MOESM10_ESM.zip › 04_Figure_04/4C/60min-ultraID-STREP(+)/_FULL-RANGE-60min-ultraID-STREP(+).tif]

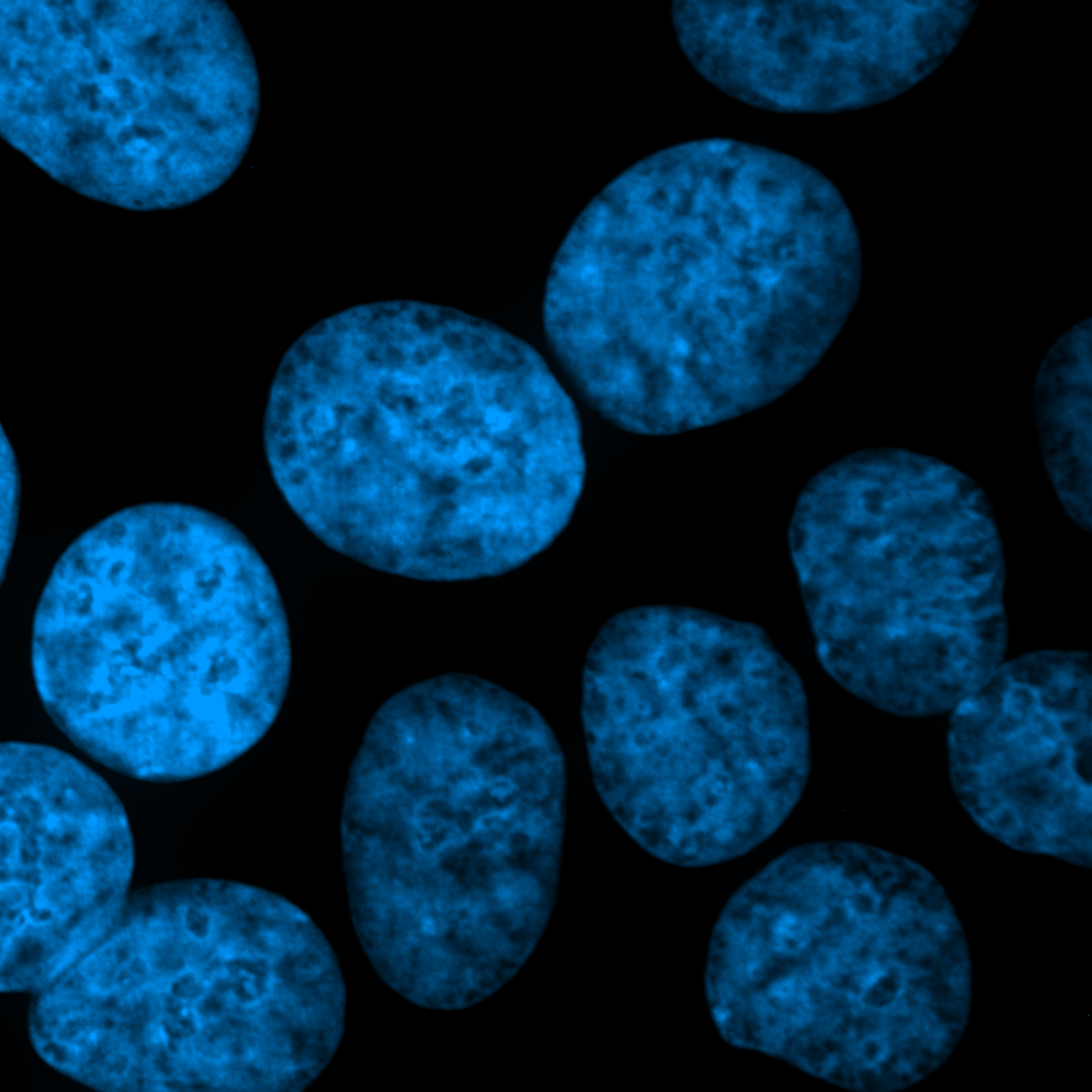

Supplement: Supplementary file 10 — Source data Fig. 4 [file 44318_2024_337_MOESM10_ESM.zip › 04_Figure_04/4C/60min-ultraID-STREP(-)/60min-ultraID-STREP(-)-DAPI.tif]

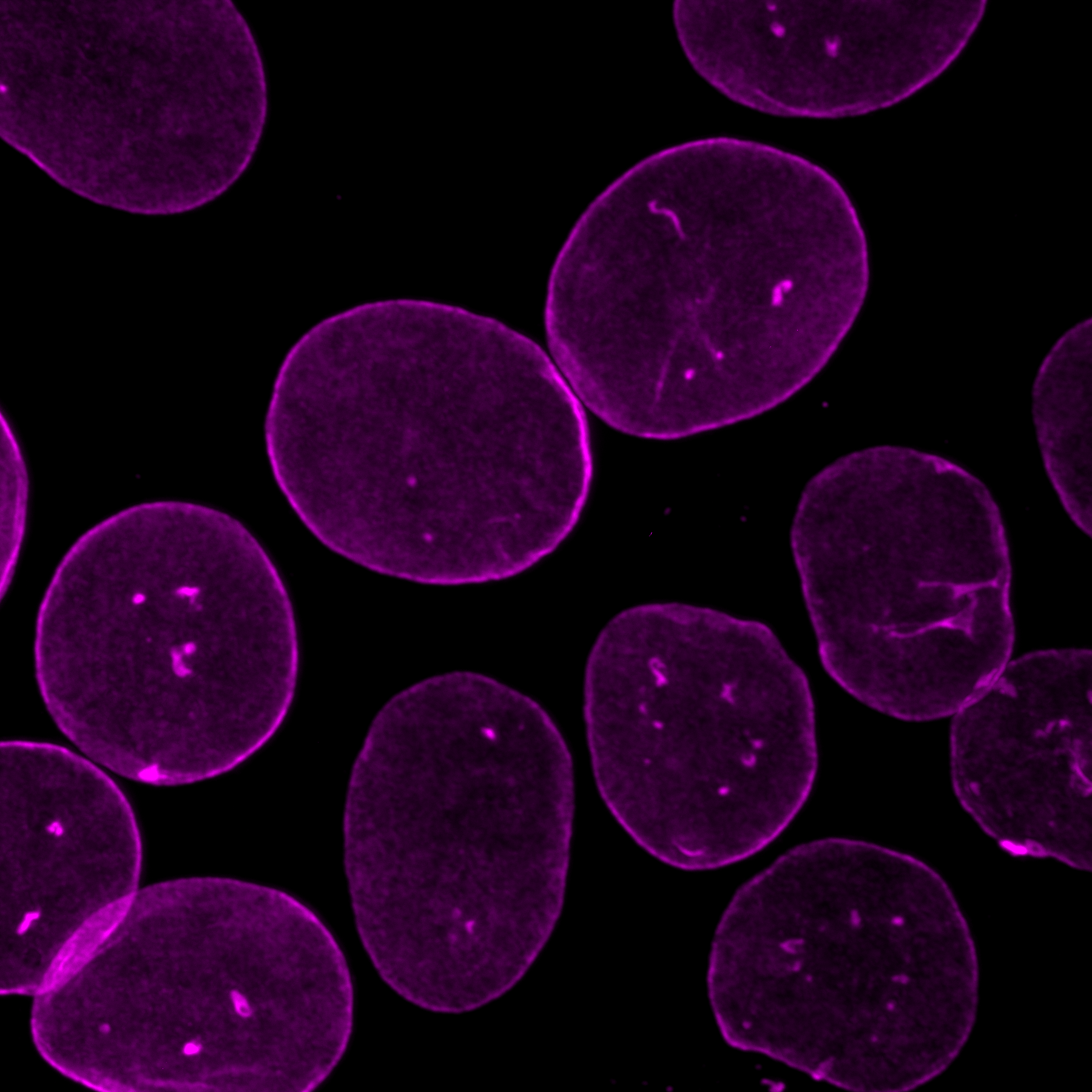

Supplement: Supplementary file 10 — Source data Fig. 4 [file 44318_2024_337_MOESM10_ESM.zip › 04_Figure_04/4C/60min-ultraID-STREP(-)/60min-ultraID-STREP(-)-LMNB1.tif]

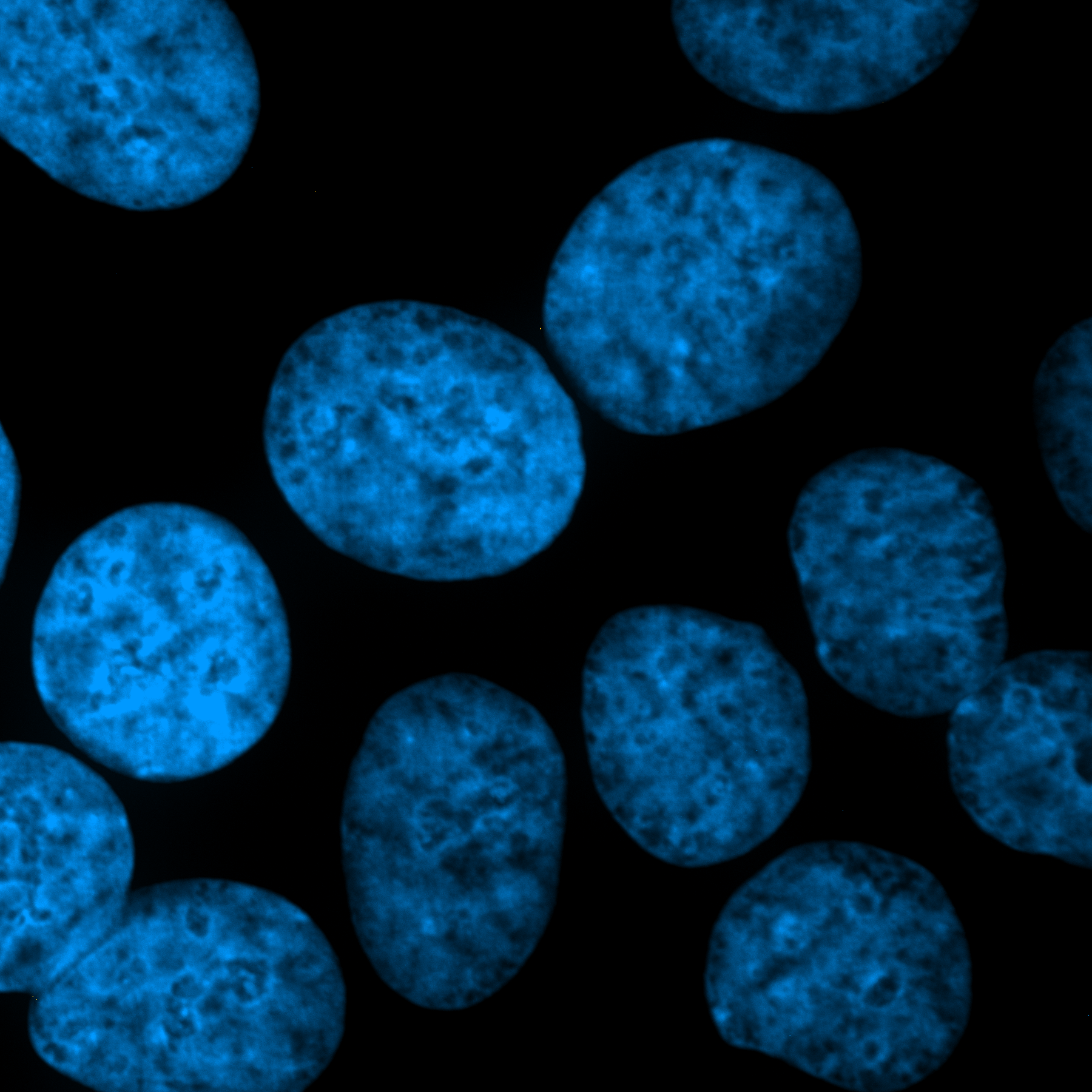

Supplement: Supplementary file 10 — Source data Fig. 4 [file 44318_2024_337_MOESM10_ESM.zip › 04_Figure_04/4C/60min-ultraID-STREP(-)/60min-ultraID-STREP(-)-Merge.tif]

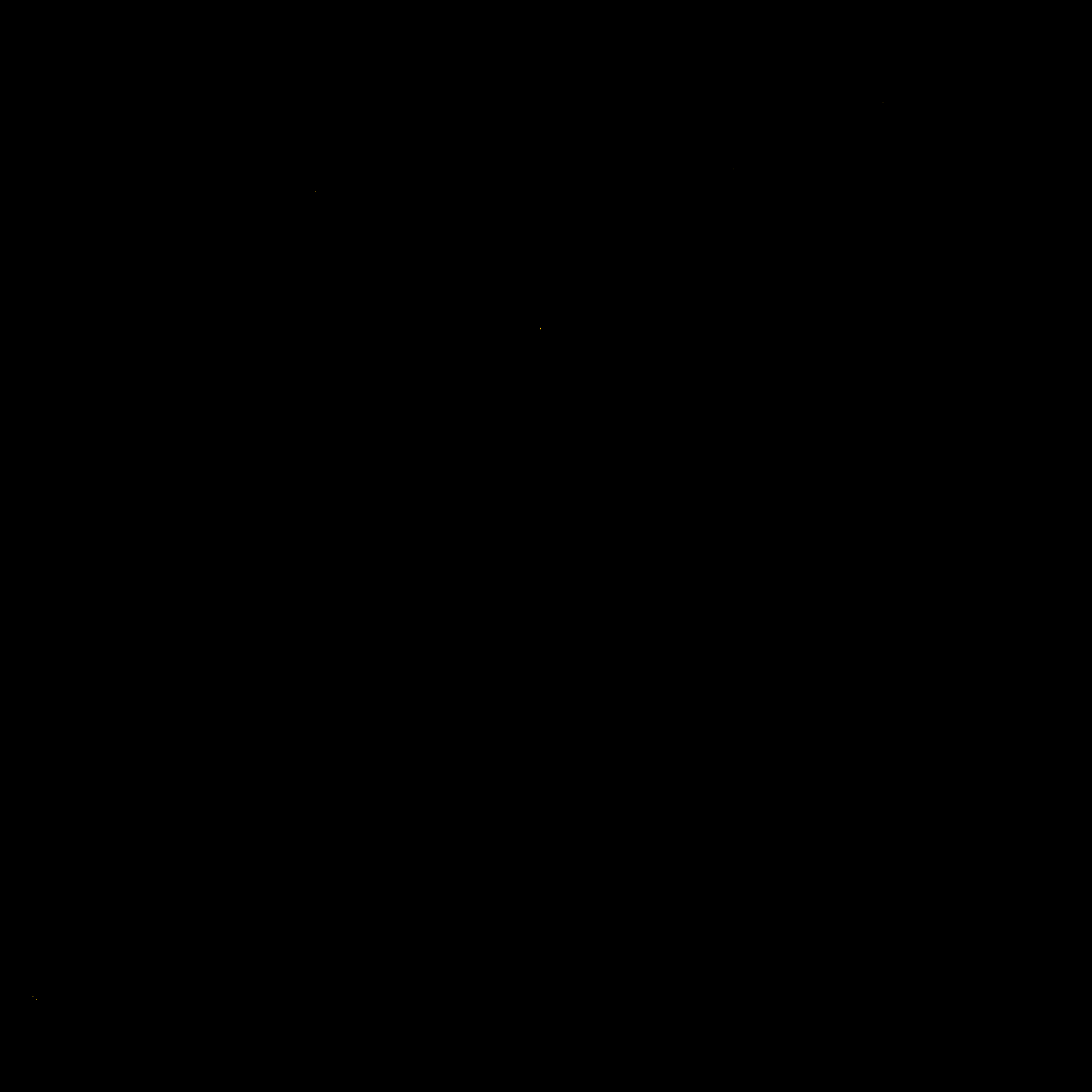

Supplement: Supplementary file 10 — Source data Fig. 4 [file 44318_2024_337_MOESM10_ESM.zip › 04_Figure_04/4C/60min-ultraID-STREP(-)/60min-ultraID-STREP(-)-Streptavidin.tif]

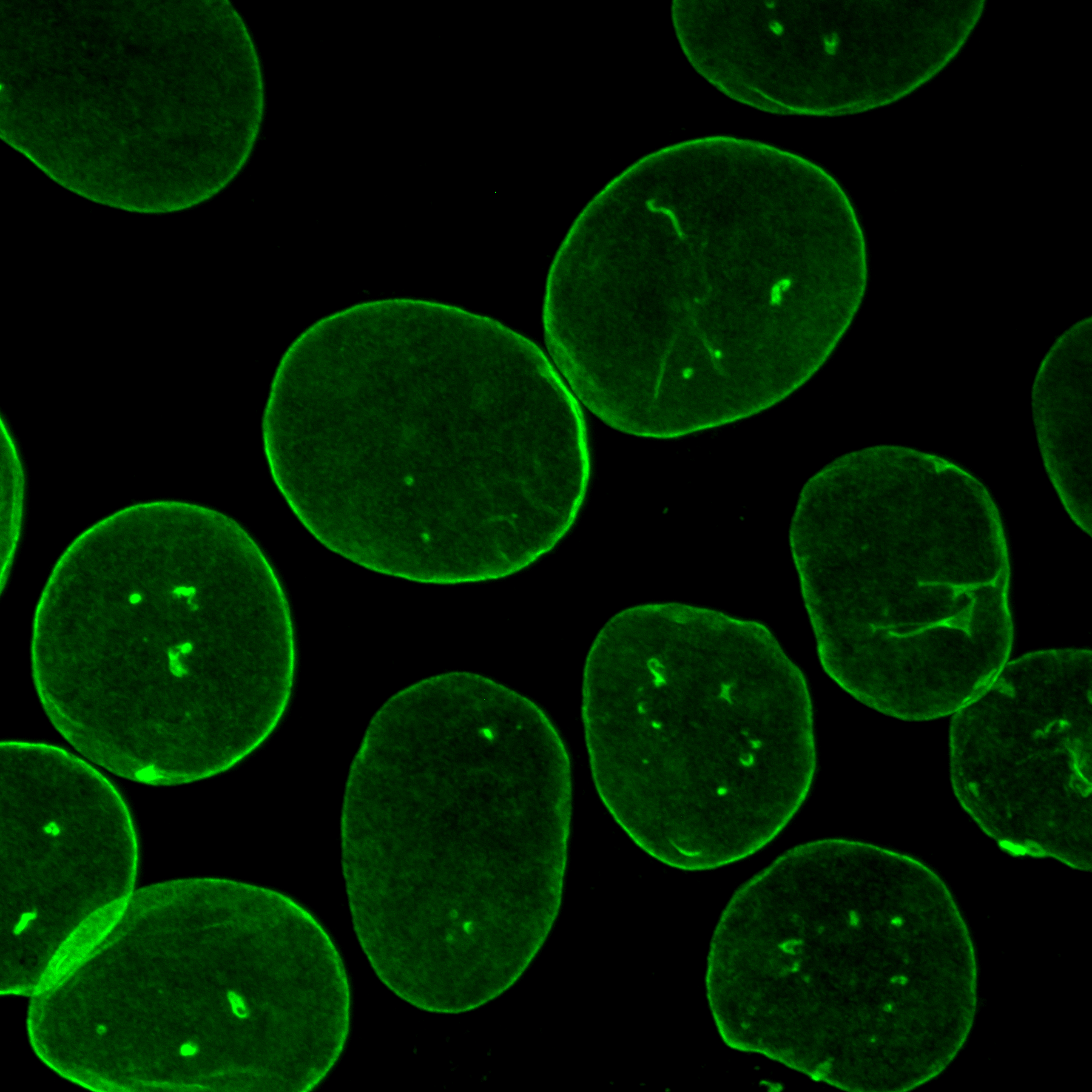

Supplement: Supplementary file 10 — Source data Fig. 4 [file 44318_2024_337_MOESM10_ESM.zip › 04_Figure_04/4C/60min-ultraID-STREP(-)/60min-ultraID-STREP(-)-V5.tif]

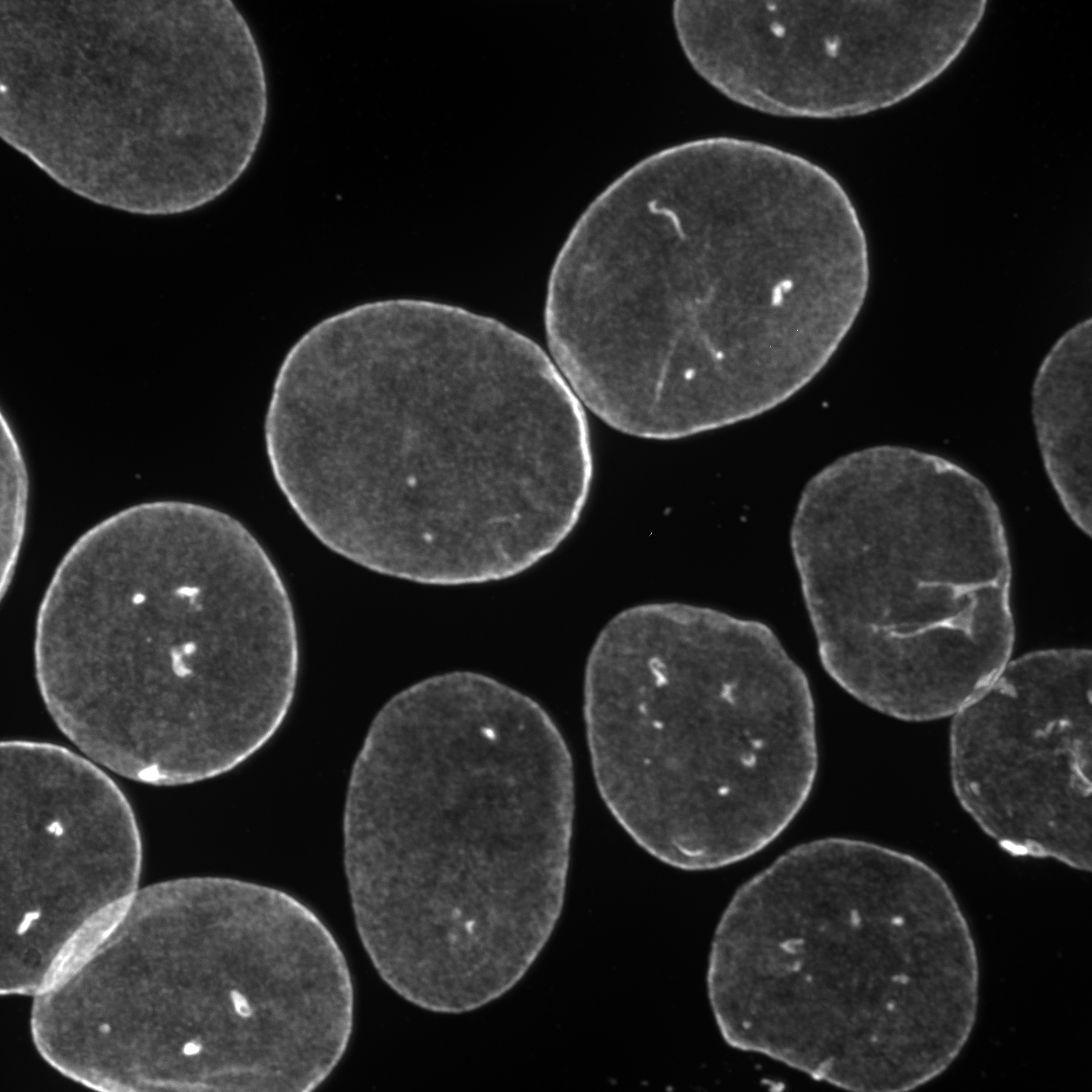

Supplement: Supplementary file 10 — Source data Fig. 4 [file 44318_2024_337_MOESM10_ESM.zip › 04_Figure_04/4C/60min-ultraID-STREP(-)/_FULL-RANGE-60min-ultraID-STREP(-).tif]

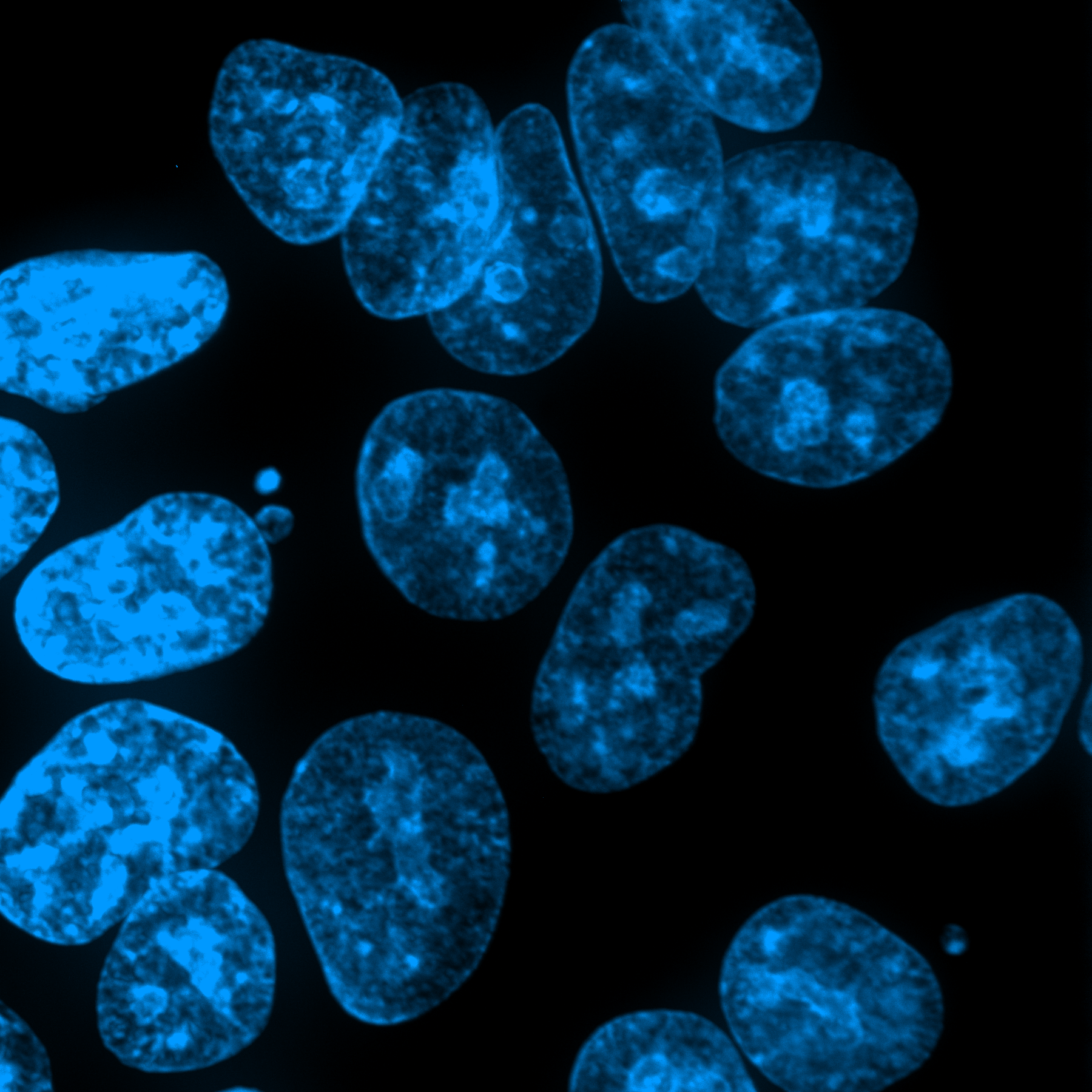

Supplement: Supplementary file 10 — Source data Fig. 4 [file 44318_2024_337_MOESM10_ESM.zip › 04_Figure_04/4C/60min-WT-STREP(+)/60min-WT-STREP(+)-DAPI.tif]

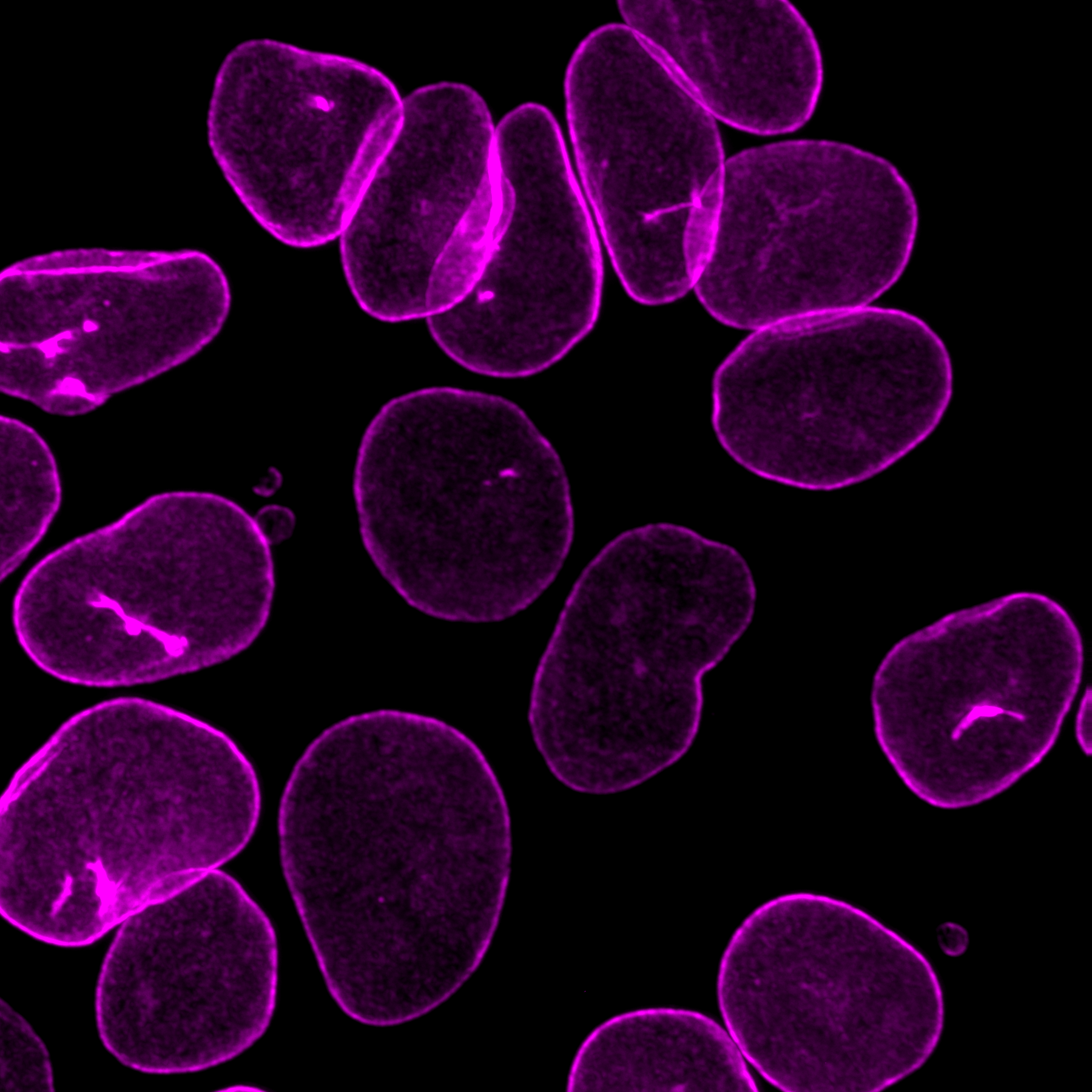

Supplement: Supplementary file 10 — Source data Fig. 4 [file 44318_2024_337_MOESM10_ESM.zip › 04_Figure_04/4C/60min-WT-STREP(+)/60min-WT-STREP(+)-LMNB1.tif]

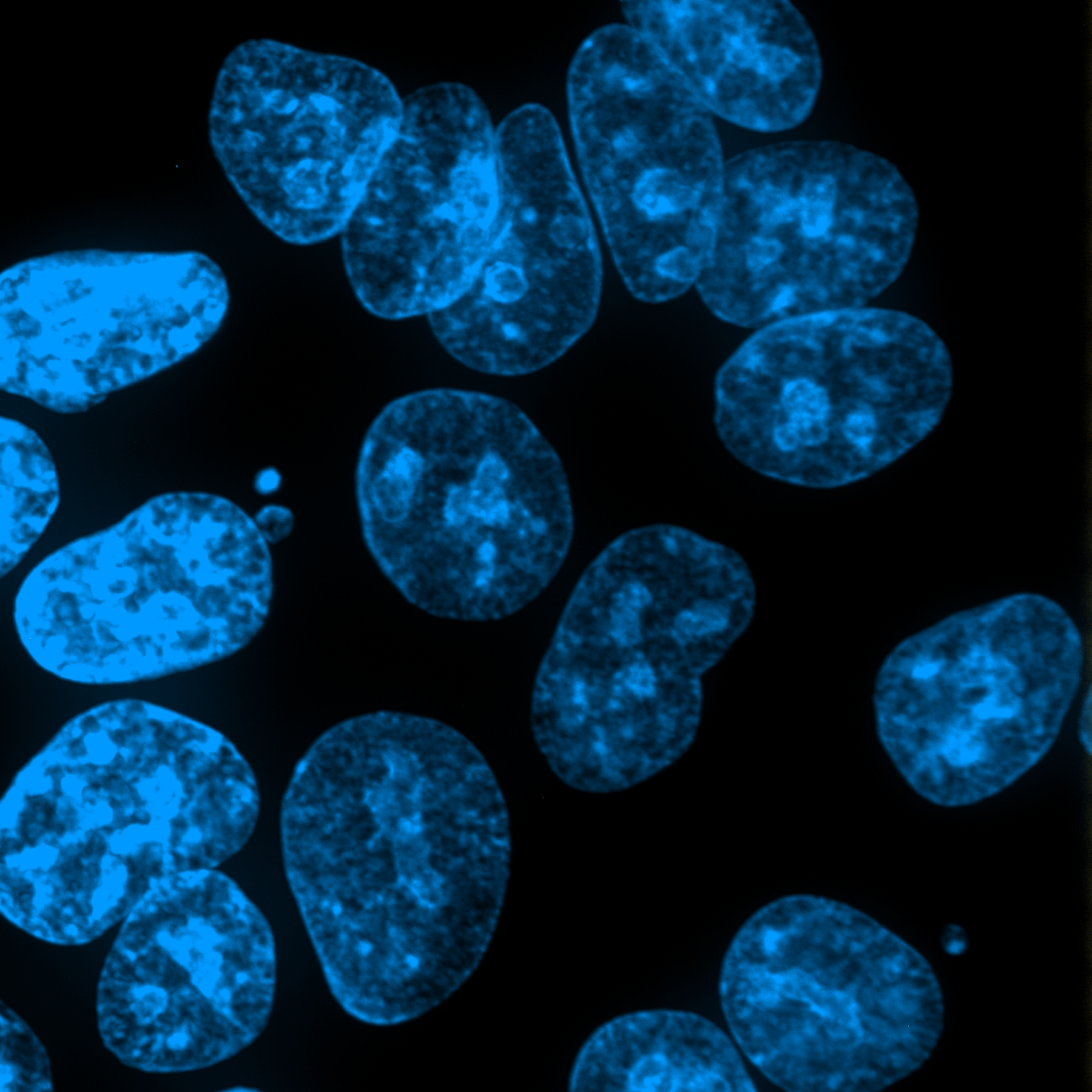

Supplement: Supplementary file 10 — Source data Fig. 4 [file 44318_2024_337_MOESM10_ESM.zip › 04_Figure_04/4C/60min-WT-STREP(+)/60min-WT-STREP(+)-Merge.tif]

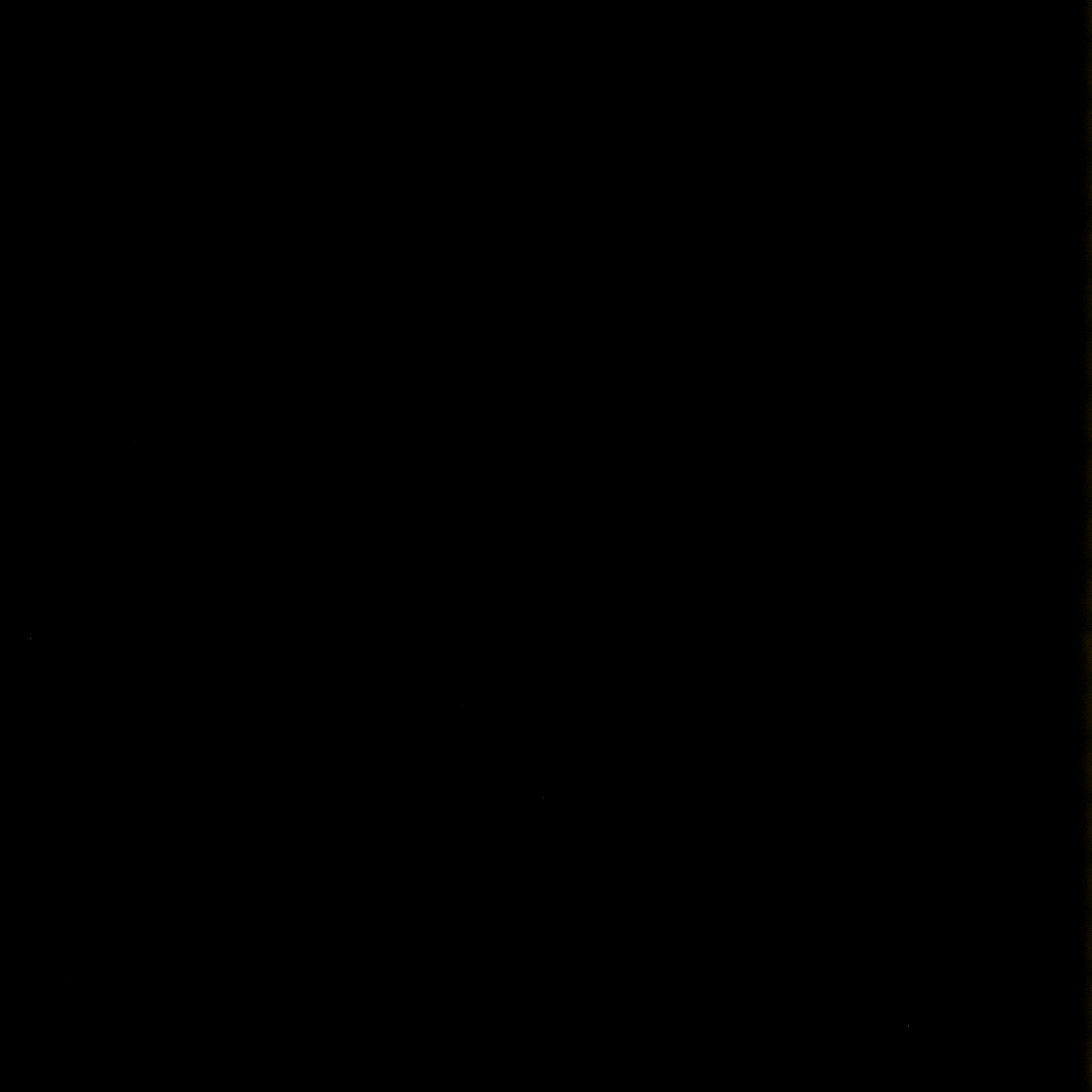

Supplement: Supplementary file 10 — Source data Fig. 4 [file 44318_2024_337_MOESM10_ESM.zip › 04_Figure_04/4C/60min-WT-STREP(+)/60min-WT-STREP(+)-Streptavidin.tif]

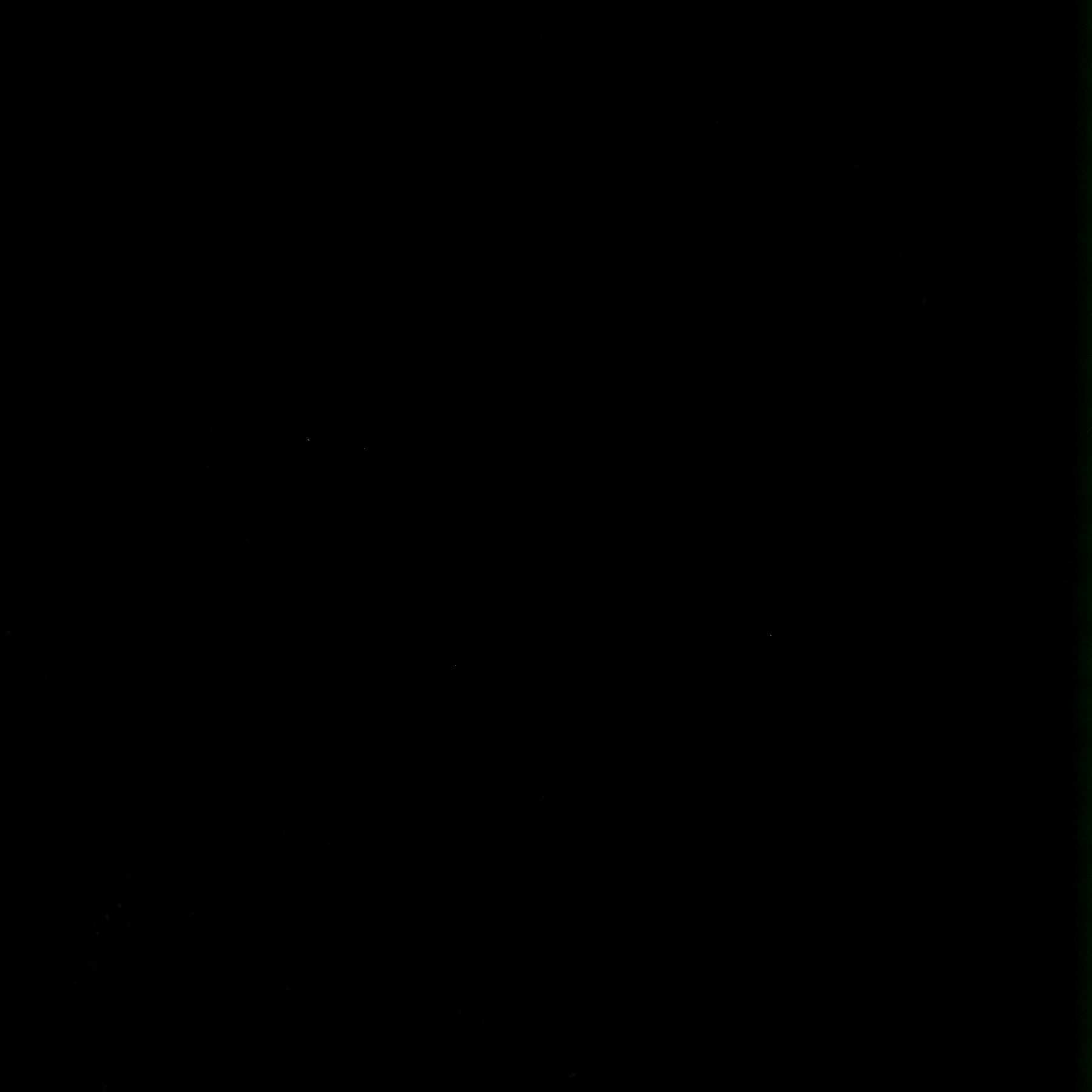

Supplement: Supplementary file 10 — Source data Fig. 4 [file 44318_2024_337_MOESM10_ESM.zip › 04_Figure_04/4C/60min-WT-STREP(+)/60min-WT-STREP(+)-V5.tif]

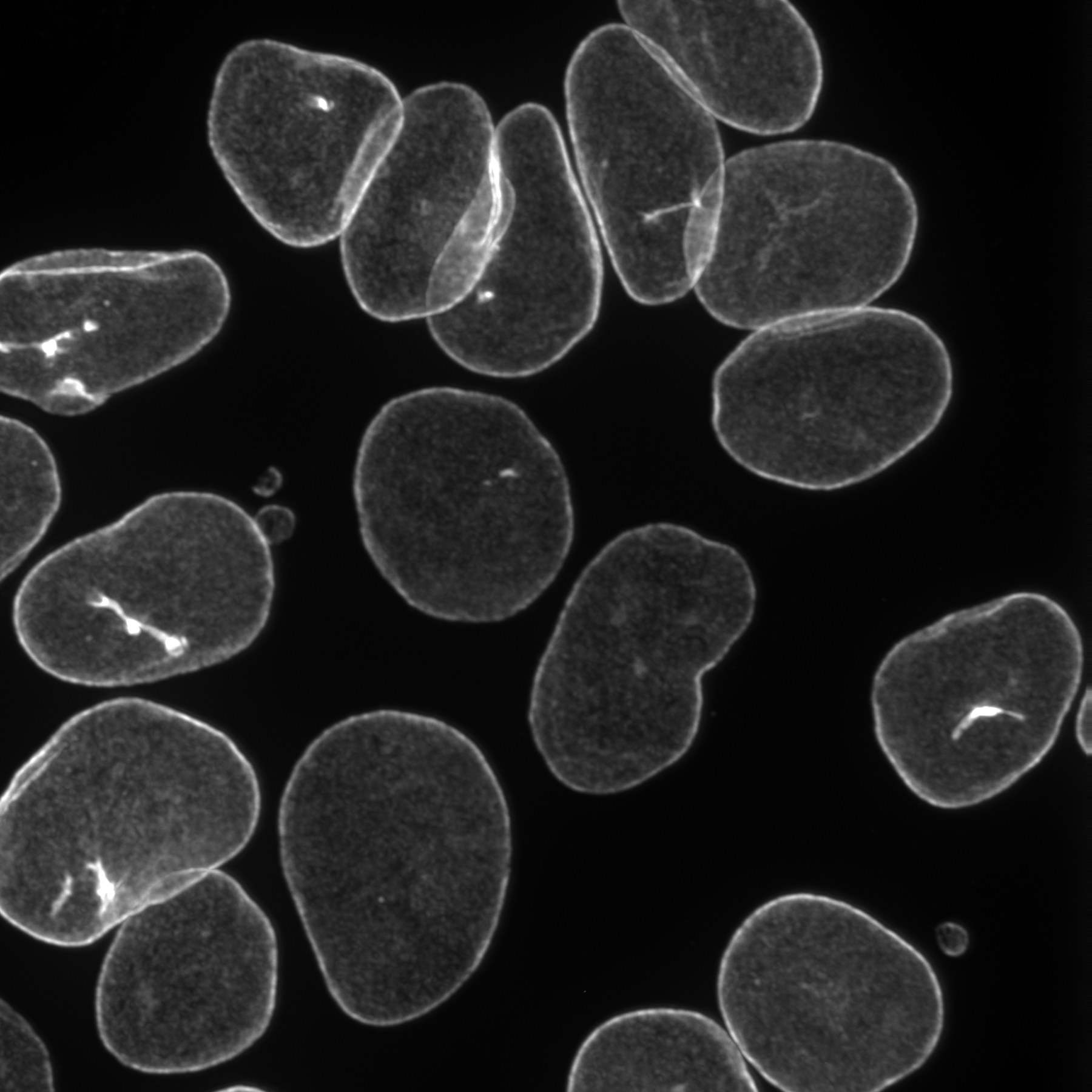

Supplement: Supplementary file 10 — Source data Fig. 4 [file 44318_2024_337_MOESM10_ESM.zip › 04_Figure_04/4C/60min-WT-STREP(+)/_FULL-RANGE-60min-WT-STREP(+).tif]

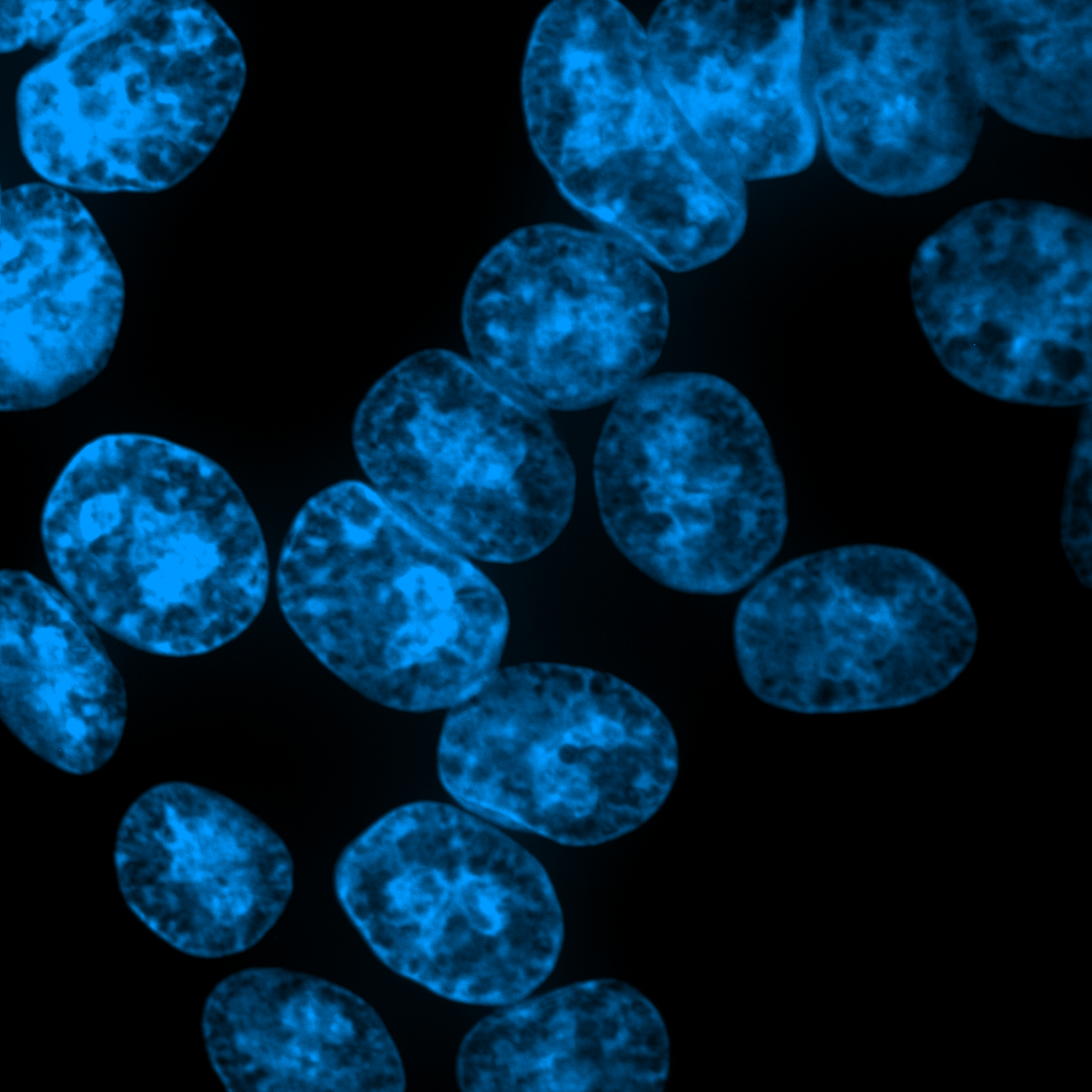

Supplement: Supplementary file 10 — Source data Fig. 4 [file 44318_2024_337_MOESM10_ESM.zip › 04_Figure_04/4C/60min-WT-STREP(-)/60min-WT-STREP(-)-DAPI.tif]

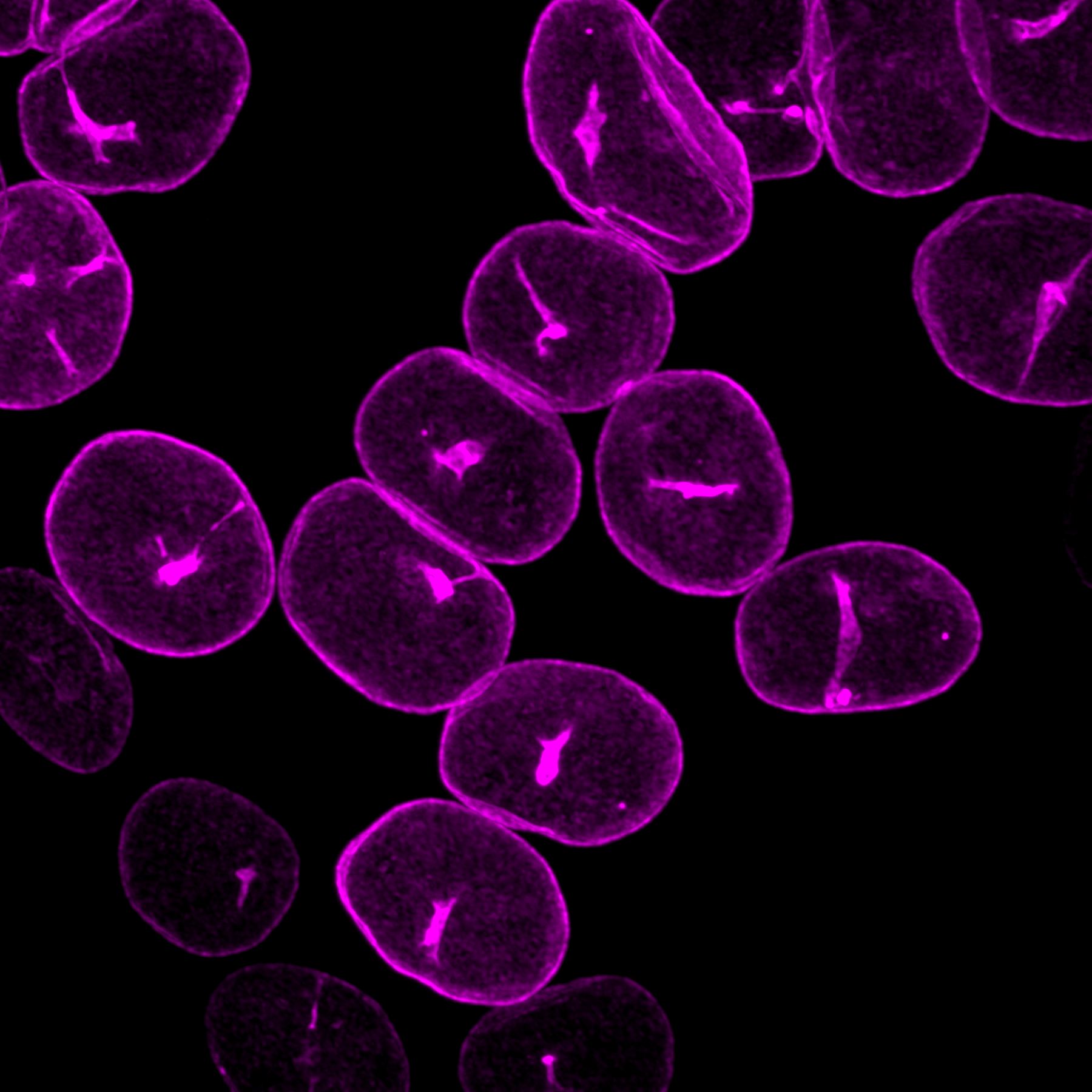

Supplement: Supplementary file 10 — Source data Fig. 4 [file 44318_2024_337_MOESM10_ESM.zip › 04_Figure_04/4C/60min-WT-STREP(-)/60min-WT-STREP(-)-LMNB1.tif]

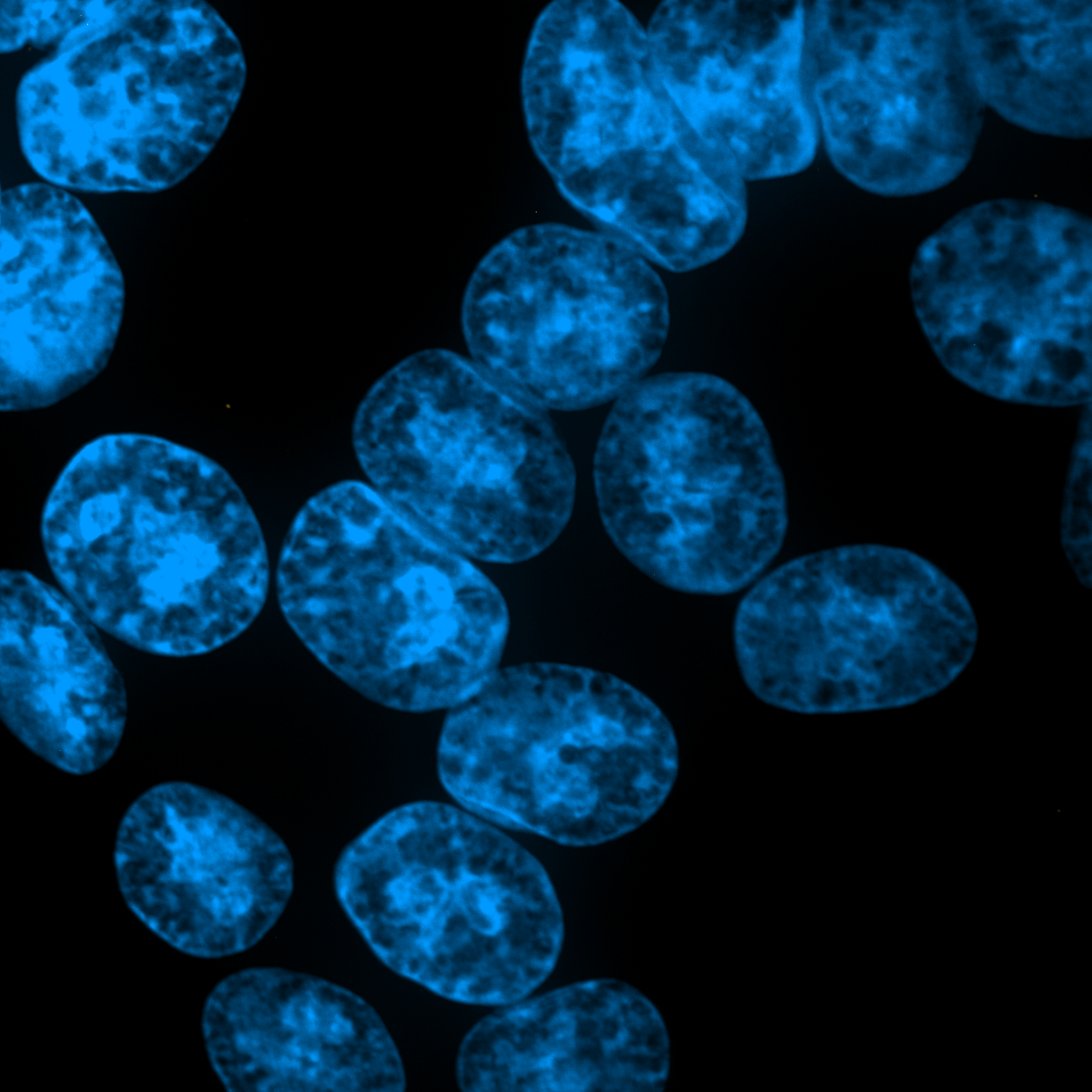

Supplement: Supplementary file 10 — Source data Fig. 4 [file 44318_2024_337_MOESM10_ESM.zip › 04_Figure_04/4C/60min-WT-STREP(-)/60min-WT-STREP(-)-Merge.tif]

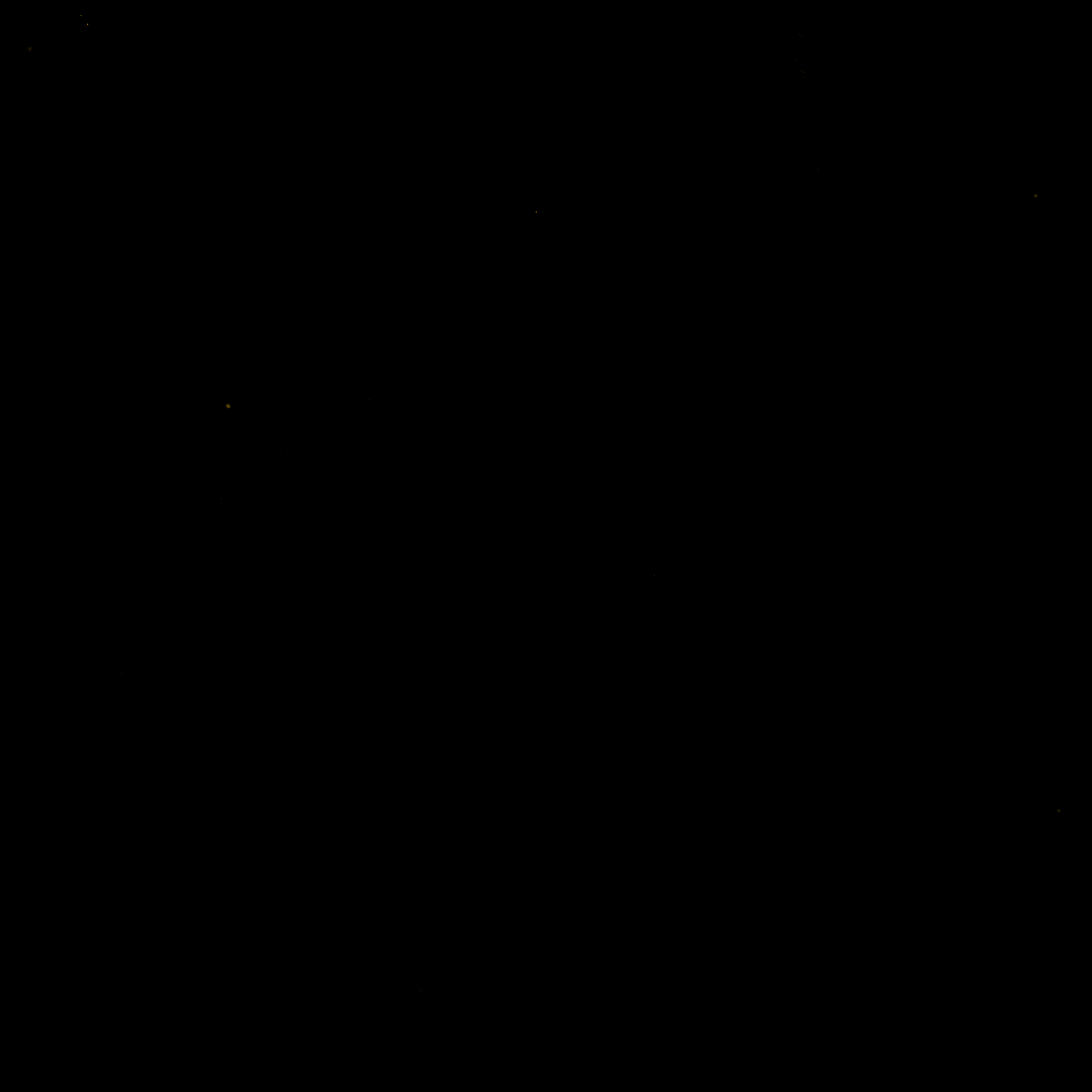

Supplement: Supplementary file 10 — Source data Fig. 4 [file 44318_2024_337_MOESM10_ESM.zip › 04_Figure_04/4C/60min-WT-STREP(-)/60min-WT-STREP(-)-Streptavidin.tif]

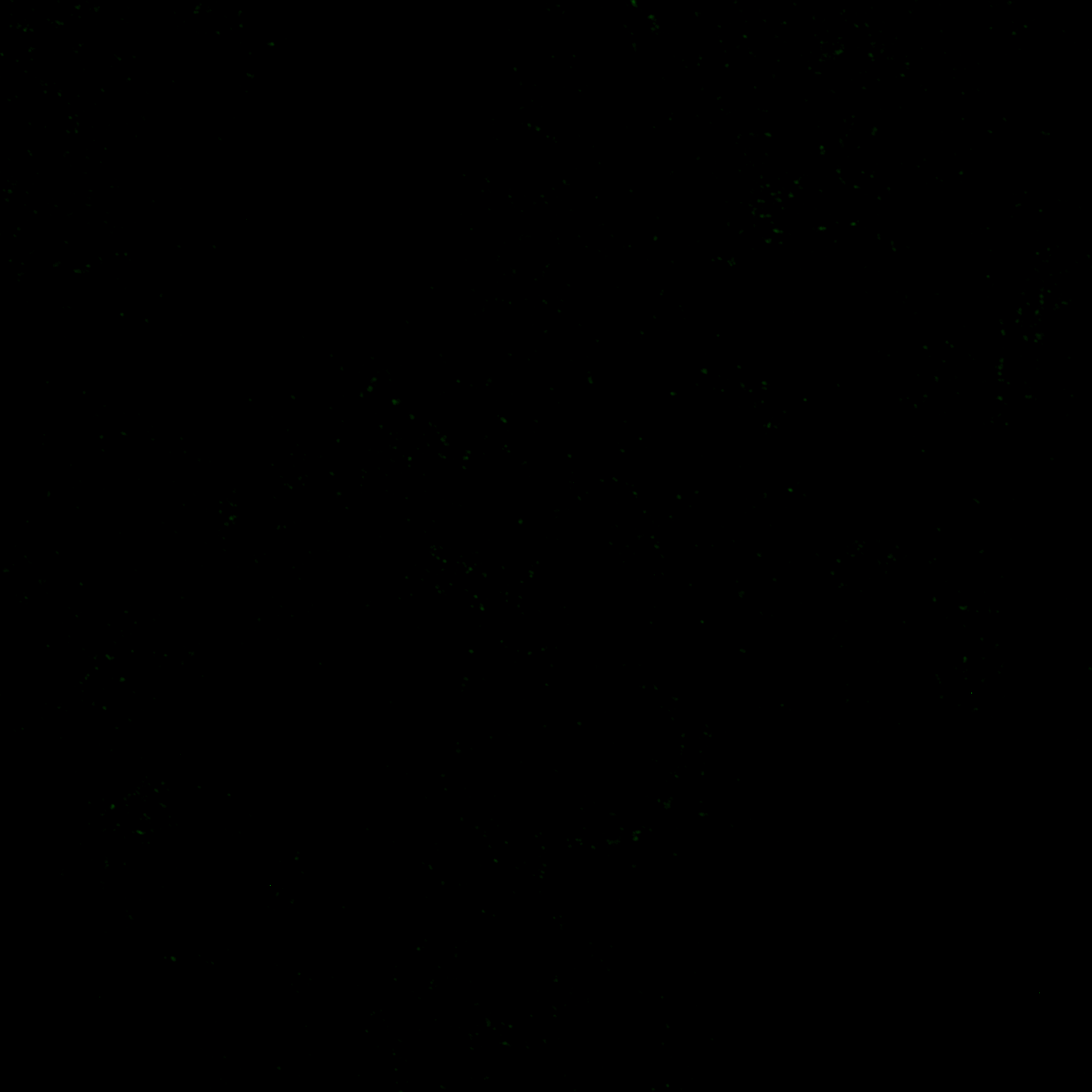

Supplement: Supplementary file 10 — Source data Fig. 4 [file 44318_2024_337_MOESM10_ESM.zip › 04_Figure_04/4C/60min-WT-STREP(-)/60min-WT-STREP(-)-V5.tif]

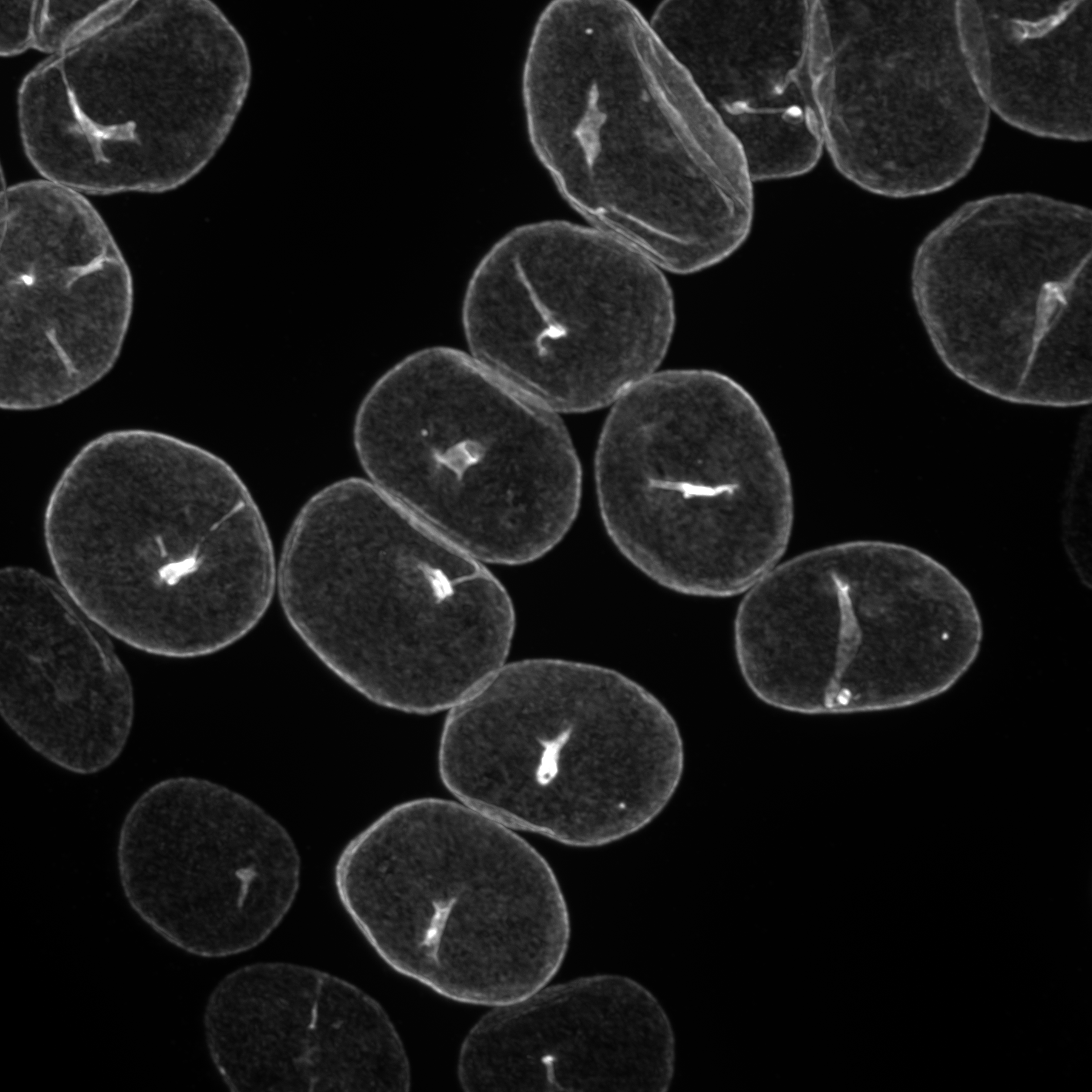

Supplement: Supplementary file 10 — Source data Fig. 4 [file 44318_2024_337_MOESM10_ESM.zip › 04_Figure_04/4C/60min-WT-STREP(-)/_FULL-RANGE-60min-WT-STREP(-).tif]

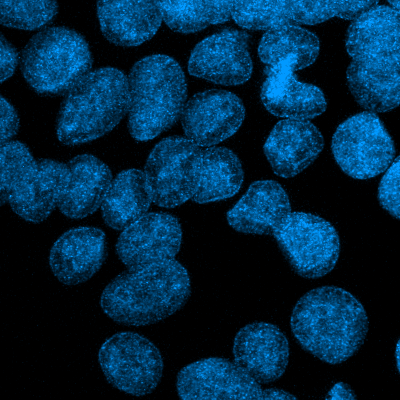

Supplement: Supplementary file 10 — Source data Fig. 4 [file 44318_2024_337_MOESM10_ESM.zip › 04_Figure_04/4E/dTAG-0h/dTAG-0h-DAPI.tif]

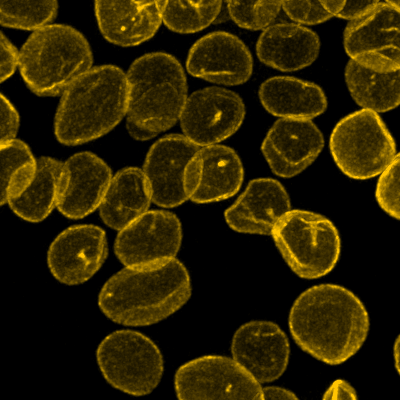

Supplement: Supplementary file 10 — Source data Fig. 4 [file 44318_2024_337_MOESM10_ESM.zip › 04_Figure_04/4E/dTAG-0h/dTAG-0h-LMNB1.tif]

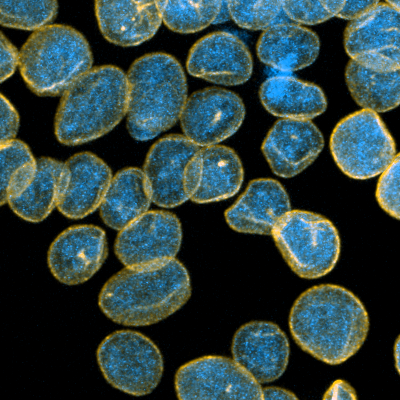

Supplement: Supplementary file 10 — Source data Fig. 4 [file 44318_2024_337_MOESM10_ESM.zip › 04_Figure_04/4E/dTAG-0h/dTAG-0h-Merge.tif]

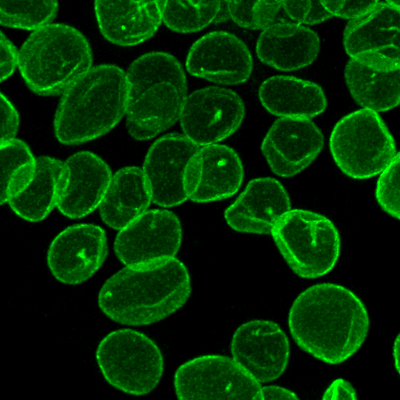

Supplement: Supplementary file 10 — Source data Fig. 4 [file 44318_2024_337_MOESM10_ESM.zip › 04_Figure_04/4E/dTAG-0h/dTAG-0h-V5.tif]

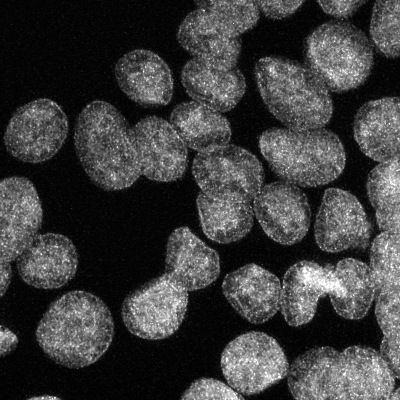

Supplement: Supplementary file 10 — Source data Fig. 4 [file 44318_2024_337_MOESM10_ESM.zip › 04_Figure_04/4E/dTAG-0h/_FULL-RANGE-dTAG-0h.tif]

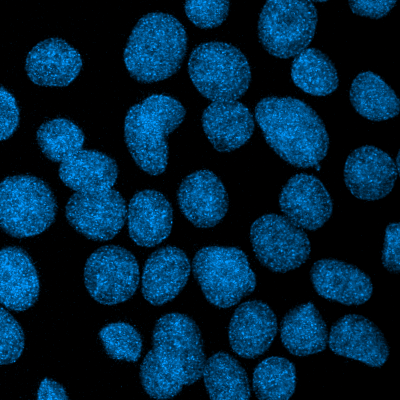

Supplement: Supplementary file 10 — Source data Fig. 4 [file 44318_2024_337_MOESM10_ESM.zip › 04_Figure_04/4E/dTAG-24h/dTAG-24h-DAPI.tif]

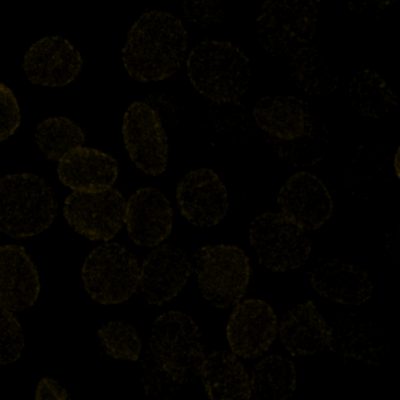

Supplement: Supplementary file 10 — Source data Fig. 4 [file 44318_2024_337_MOESM10_ESM.zip › 04_Figure_04/4E/dTAG-24h/dTAG-24h-LMNB1.tif]

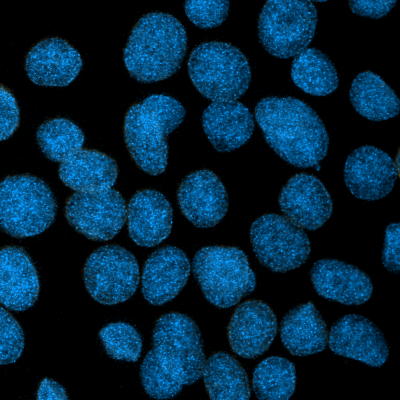

Supplement: Supplementary file 10 — Source data Fig. 4 [file 44318_2024_337_MOESM10_ESM.zip › 04_Figure_04/4E/dTAG-24h/dTAG-24h-Merge.tif]

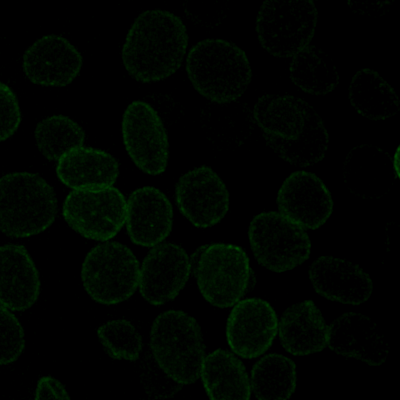

Supplement: Supplementary file 10 — Source data Fig. 4 [file 44318_2024_337_MOESM10_ESM.zip › 04_Figure_04/4E/dTAG-24h/dTAG-24h-V5.tif]

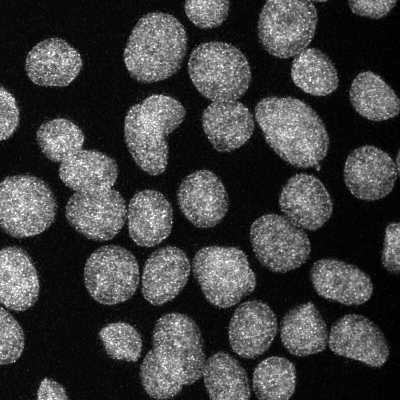

Supplement: Supplementary file 10 — Source data Fig. 4 [file 44318_2024_337_MOESM10_ESM.zip › 04_Figure_04/4E/dTAG-24h/_FULL-RANGE-dTAG-24h.tif]

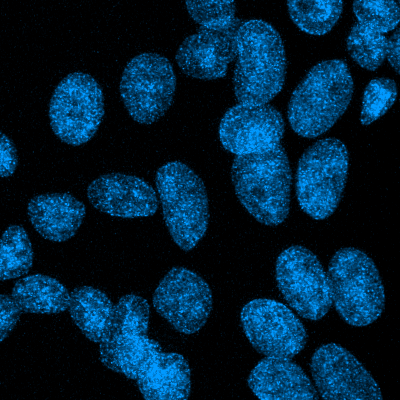

Supplement: Supplementary file 10 — Source data Fig. 4 [file 44318_2024_337_MOESM10_ESM.zip › 04_Figure_04/4E/WT-0h/WT-0h-DAPI.tif]

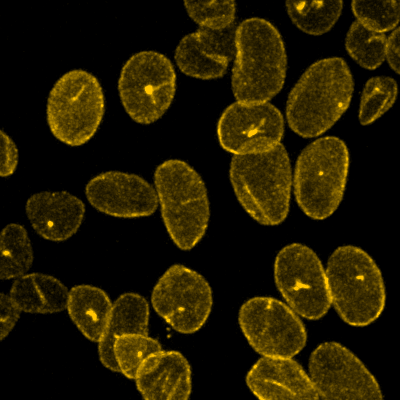

Supplement: Supplementary file 10 — Source data Fig. 4 [file 44318_2024_337_MOESM10_ESM.zip › 04_Figure_04/4E/WT-0h/WT-0h-LMNB1.tif]

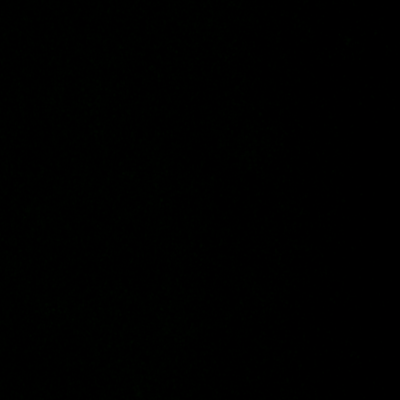

Supplement: Supplementary file 10 — Source data Fig. 4 [file 44318_2024_337_MOESM10_ESM.zip › 04_Figure_04/4E/WT-0h/WT-0h-V5.tif]

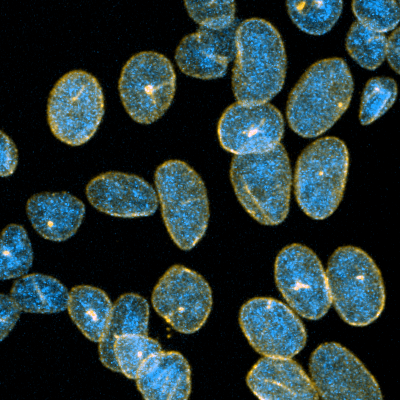

Supplement: Supplementary file 10 — Source data Fig. 4 [file 44318_2024_337_MOESM10_ESM.zip › 04_Figure_04/4E/WT-0h/WT-0h_Merge.tif]

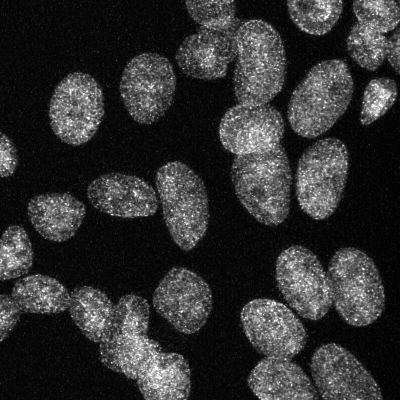

Supplement: Supplementary file 10 — Source data Fig. 4 [file 44318_2024_337_MOESM10_ESM.zip › 04_Figure_04/4E/WT-0h/_FULL-RANGE-WT-0h.tif]

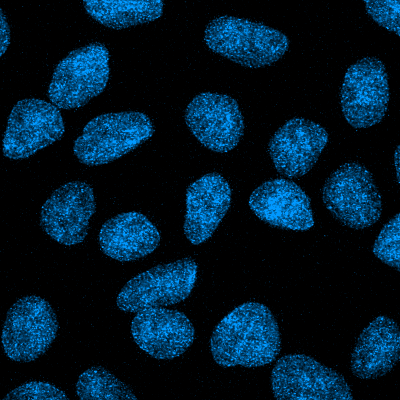

Supplement: Supplementary file 10 — Source data Fig. 4 [file 44318_2024_337_MOESM10_ESM.zip › 04_Figure_04/4E/WT-24h/WT-24h-DAPI.tif]

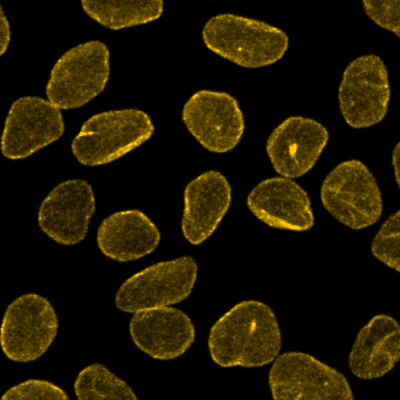

Supplement: Supplementary file 10 — Source data Fig. 4 [file 44318_2024_337_MOESM10_ESM.zip › 04_Figure_04/4E/WT-24h/WT-24h-LMNB1.tif]

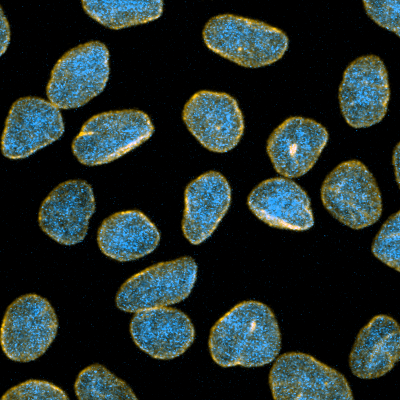

Supplement: Supplementary file 10 — Source data Fig. 4 [file 44318_2024_337_MOESM10_ESM.zip › 04_Figure_04/4E/WT-24h/WT-24h-Merge.tif]

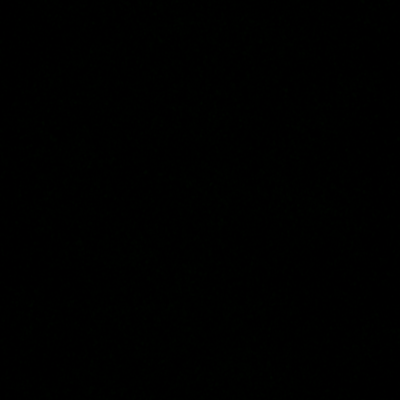

Supplement: Supplementary file 10 — Source data Fig. 4 [file 44318_2024_337_MOESM10_ESM.zip › 04_Figure_04/4E/WT-24h/WT-24h-V5.tif]

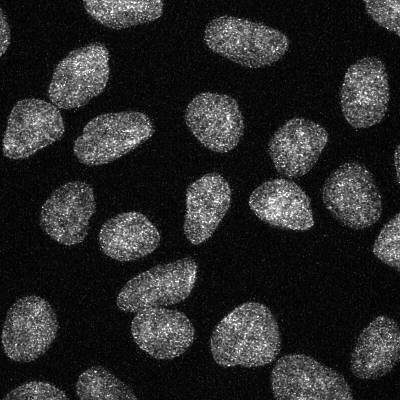

Supplement: Supplementary file 10 — Source data Fig. 4 [file 44318_2024_337_MOESM10_ESM.zip › 04_Figure_04/4E/WT-24h/_FULL-RANGE-WT-24h.tif]

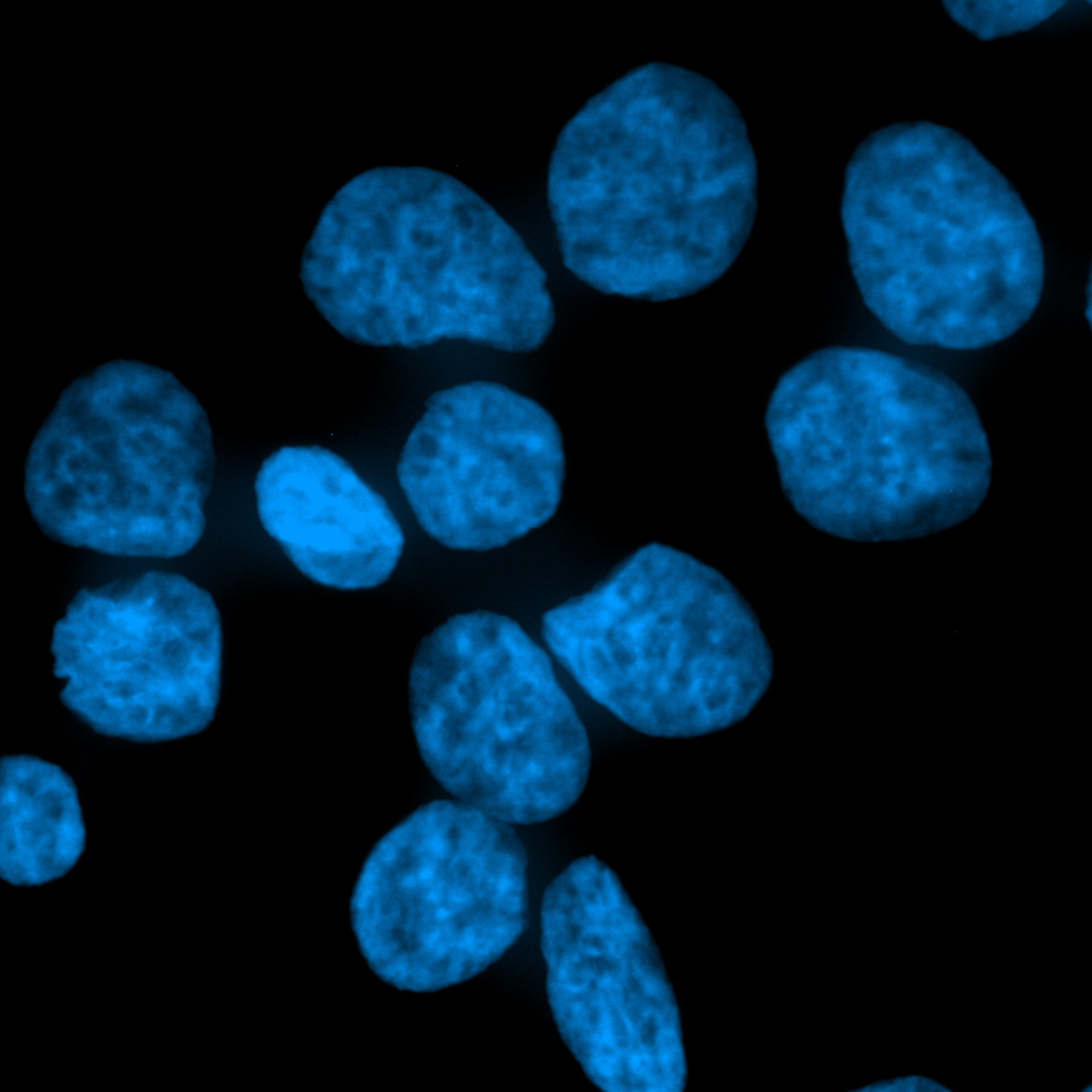

Supplement: Supplementary file 10 — Source data Fig. 4 [file 44318_2024_337_MOESM10_ESM.zip › 04_Figure_04/4G/FLAG/FLAG-DAPI.tif]
