# Supplementary figures and images for: qTAG: an adaptable plasmid scaffold for CRISPR-based endogenous tagging (part 2 of 5)
Source: EMBO J. 2024 Dec 12;44(3):947–74. doi: 10.1038/s44318-024-00337-5 (PMC11790981; doi:10.1038/s44318-024-00337-5)

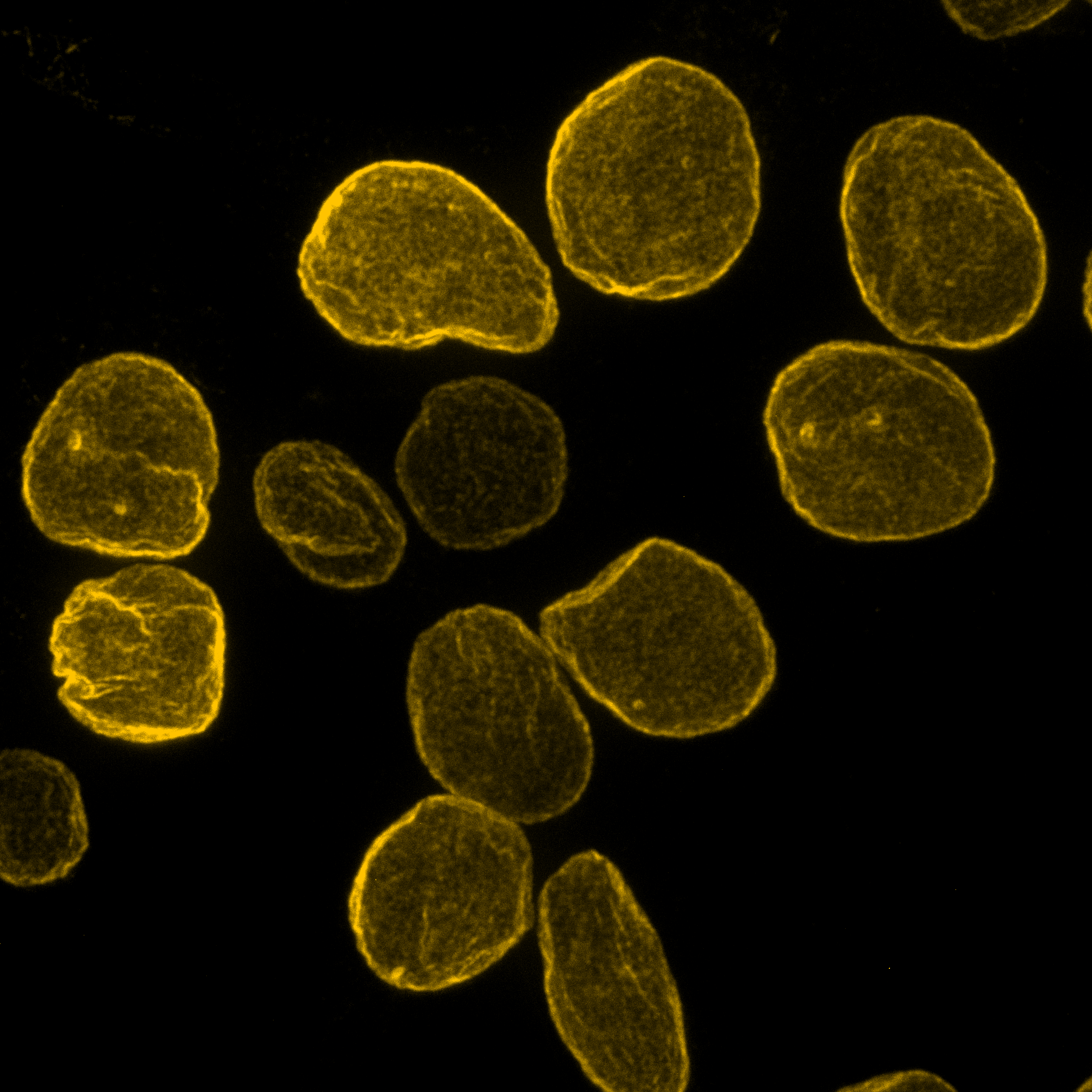

Supplement: Supplementary file 10 — Source data Fig. 4 [file 44318_2024_337_MOESM10_ESM.zip › 04_Figure_04/4G/FLAG/FLAG-FLAG.tif]

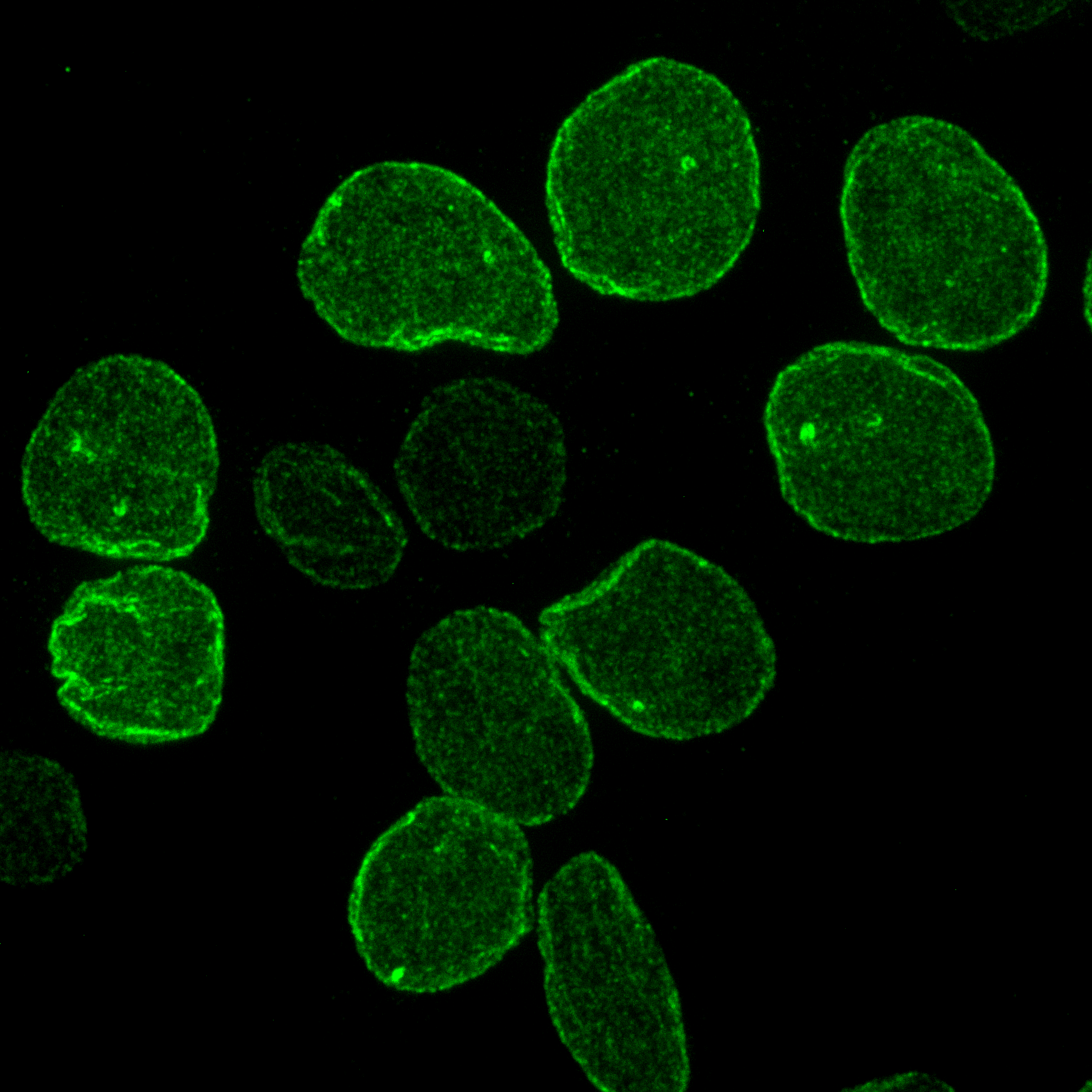

Supplement: Supplementary file 10 — Source data Fig. 4 [file 44318_2024_337_MOESM10_ESM.zip › 04_Figure_04/4G/FLAG/FLAG-LMNB1.tif]

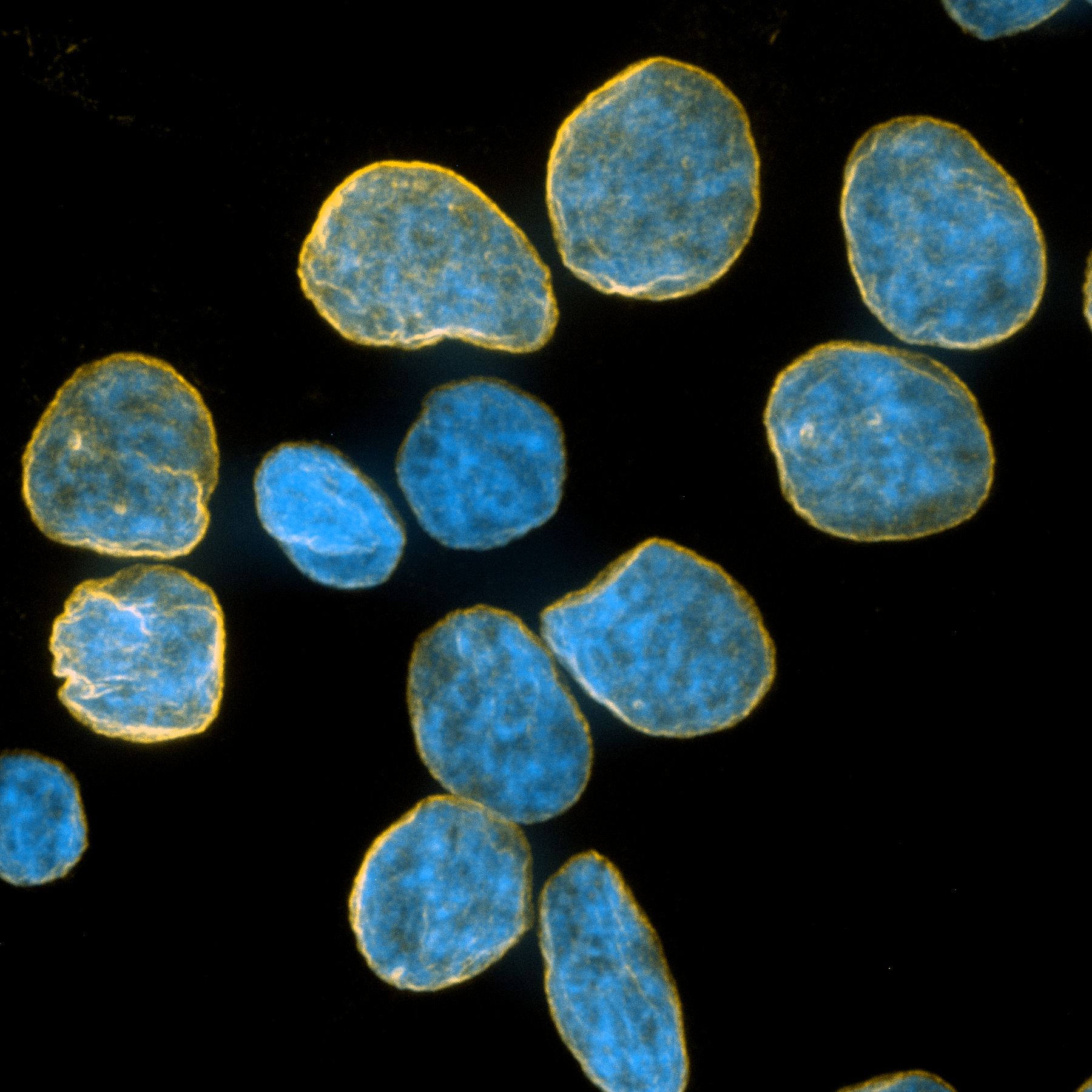

Supplement: Supplementary file 10 — Source data Fig. 4 [file 44318_2024_337_MOESM10_ESM.zip › 04_Figure_04/4G/FLAG/FLAG-Merge.tif]

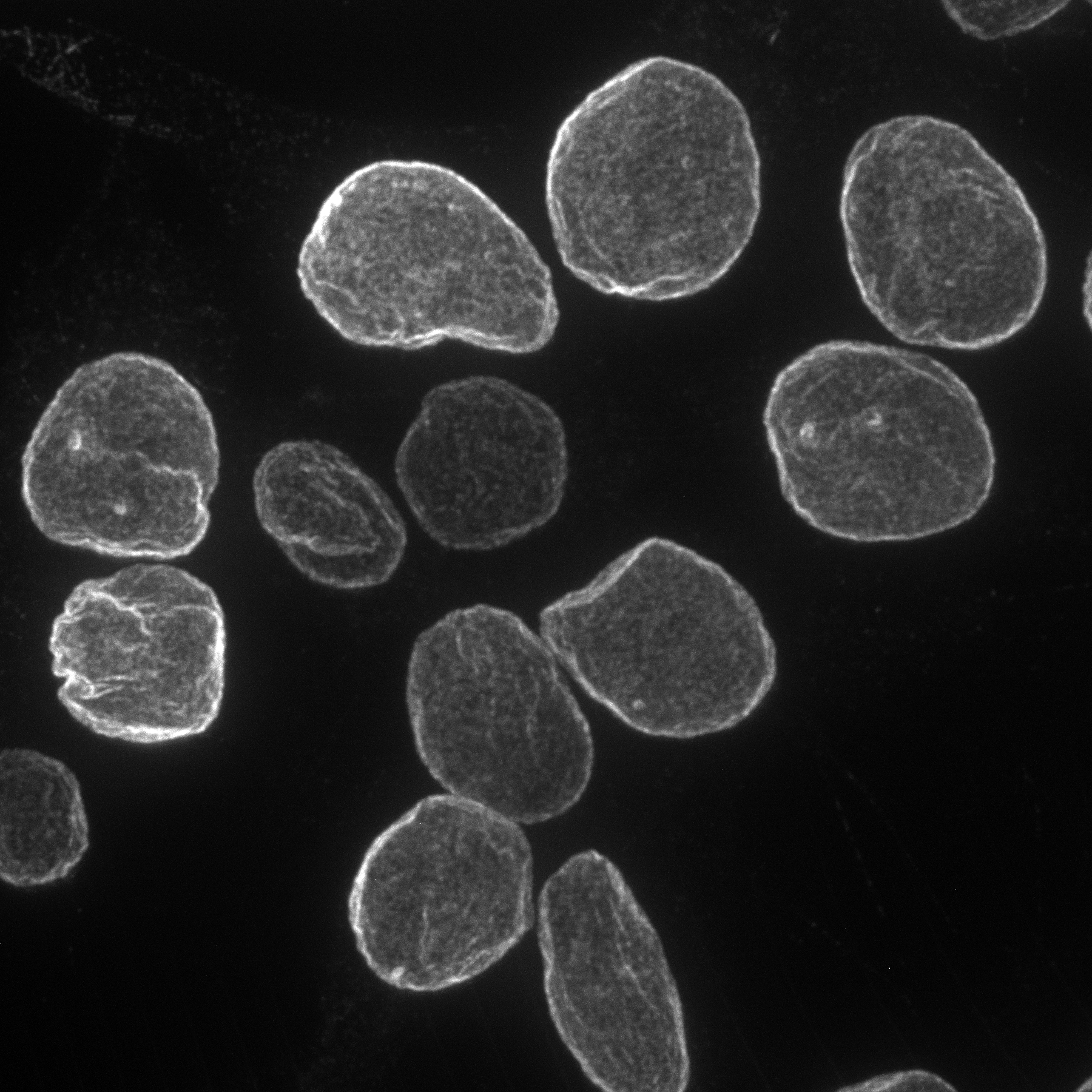

Supplement: Supplementary file 10 — Source data Fig. 4 [file 44318_2024_337_MOESM10_ESM.zip › 04_Figure_04/4G/FLAG/_FULL-RANGE-FLAG.tif]

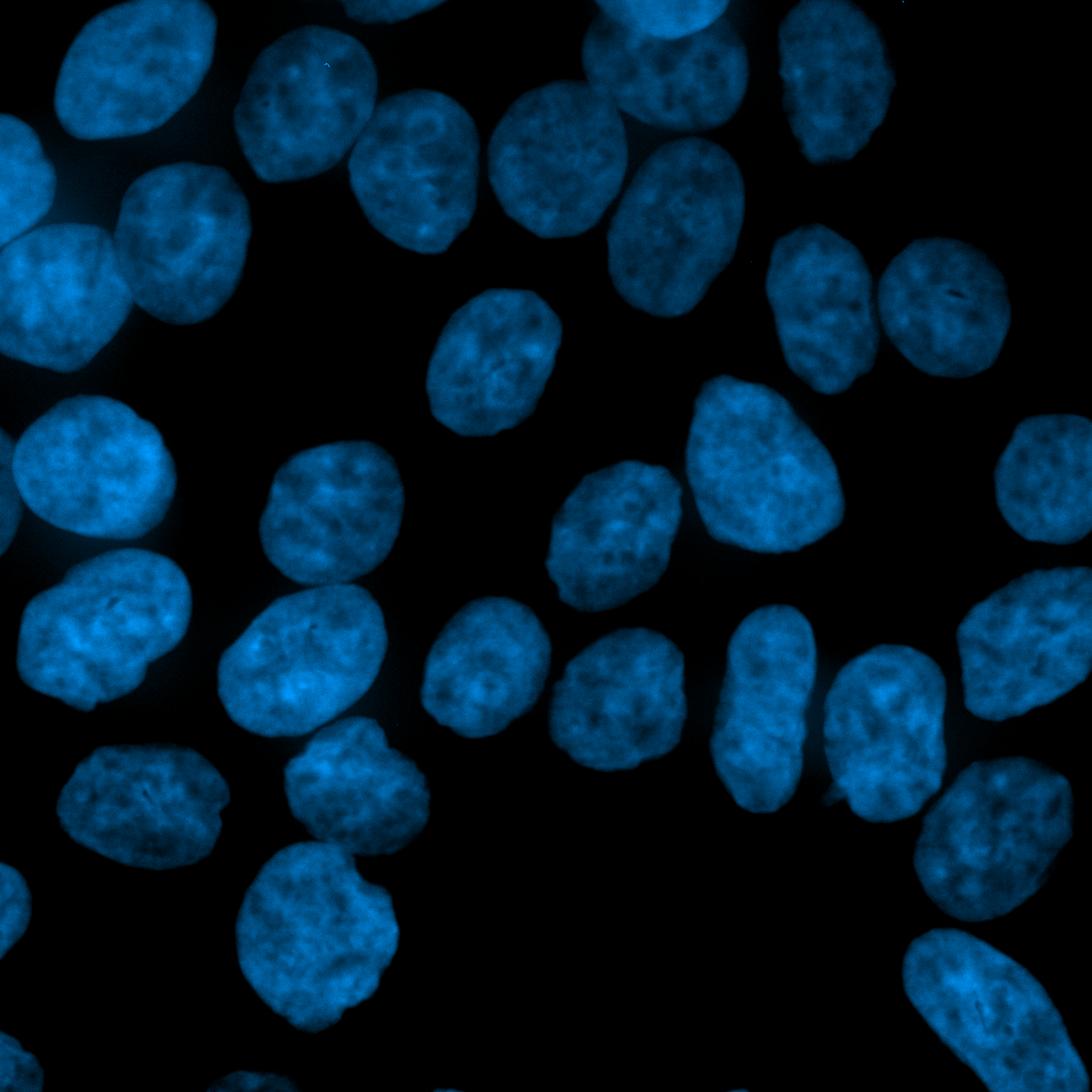

Supplement: Supplementary file 10 — Source data Fig. 4 [file 44318_2024_337_MOESM10_ESM.zip › 04_Figure_04/4G/FLAG-CTRL/FLAG-CTRL-DAPI.tif]

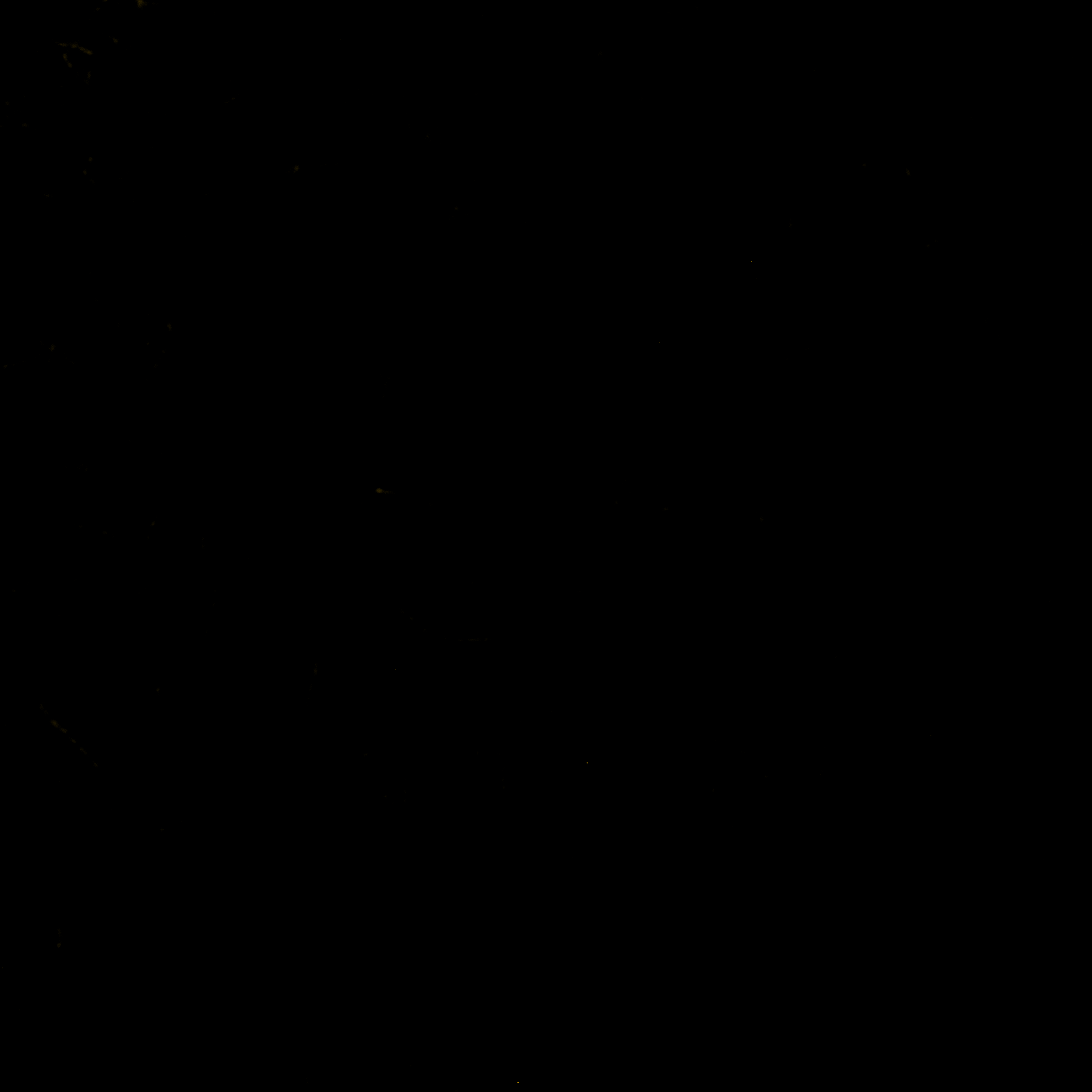

Supplement: Supplementary file 10 — Source data Fig. 4 [file 44318_2024_337_MOESM10_ESM.zip › 04_Figure_04/4G/FLAG-CTRL/FLAG-CTRL-FLAG.tif]

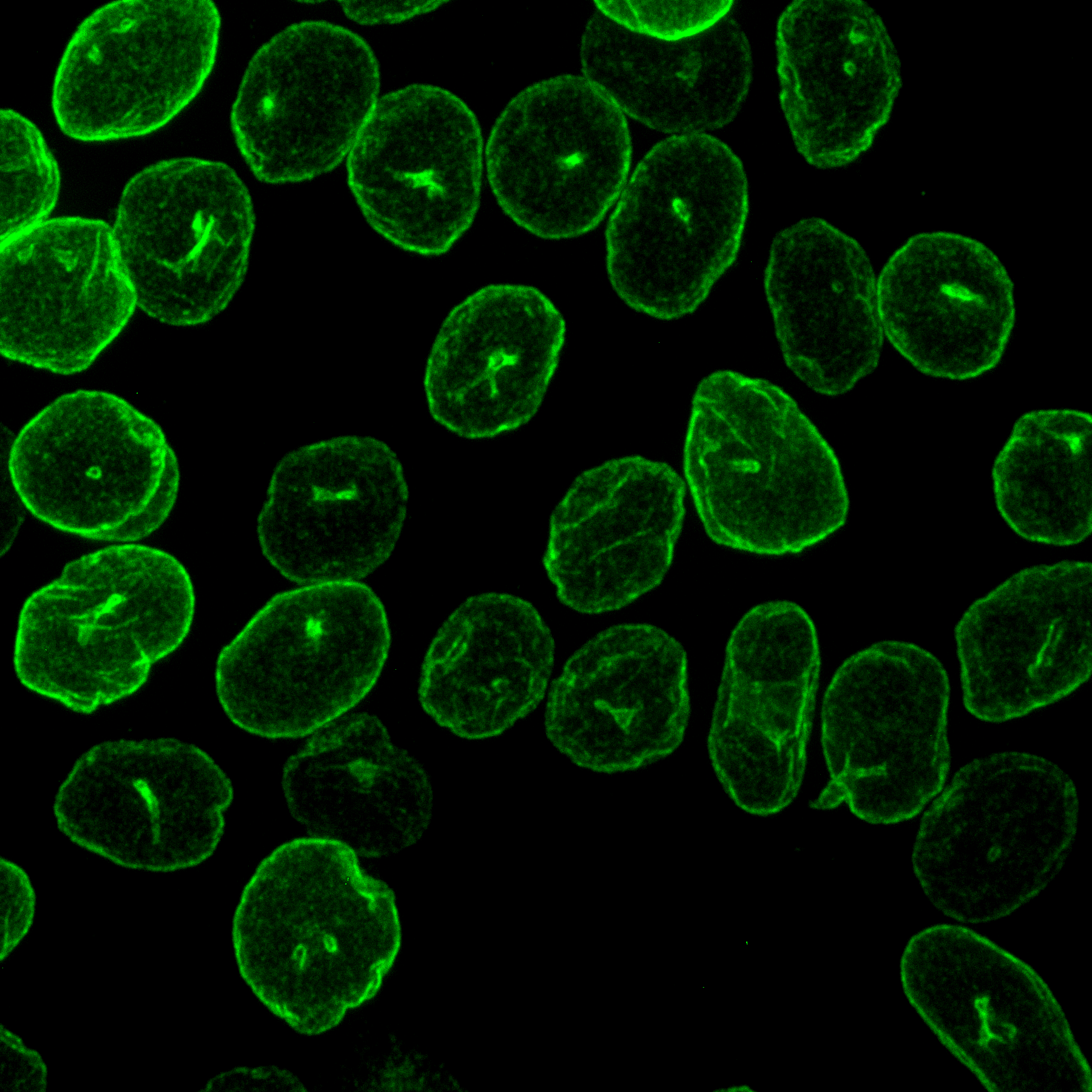

Supplement: Supplementary file 10 — Source data Fig. 4 [file 44318_2024_337_MOESM10_ESM.zip › 04_Figure_04/4G/FLAG-CTRL/FLAG-CTRL-LMNB1.tif]

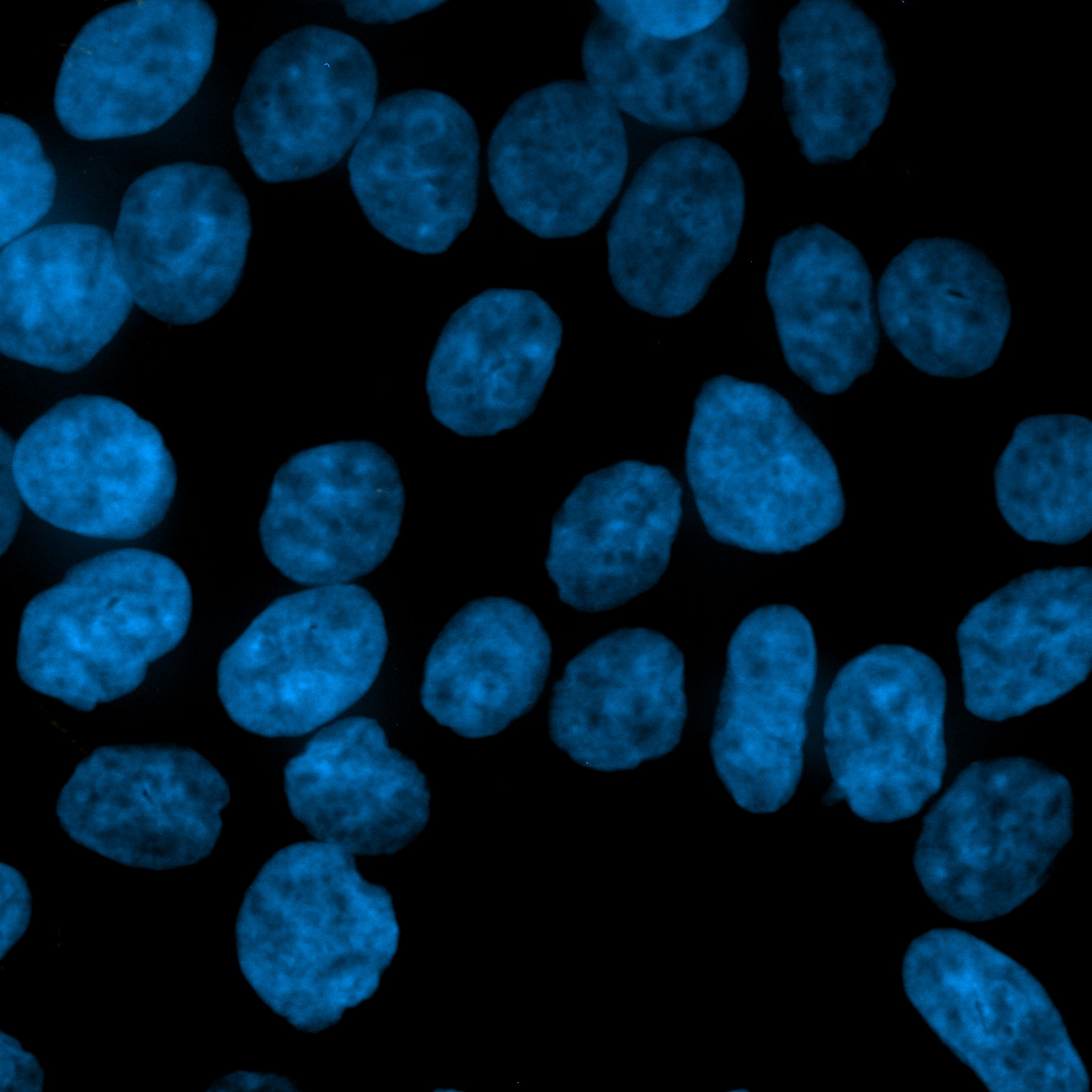

Supplement: Supplementary file 10 — Source data Fig. 4 [file 44318_2024_337_MOESM10_ESM.zip › 04_Figure_04/4G/FLAG-CTRL/FLAG-CTRL-Merge.tif]

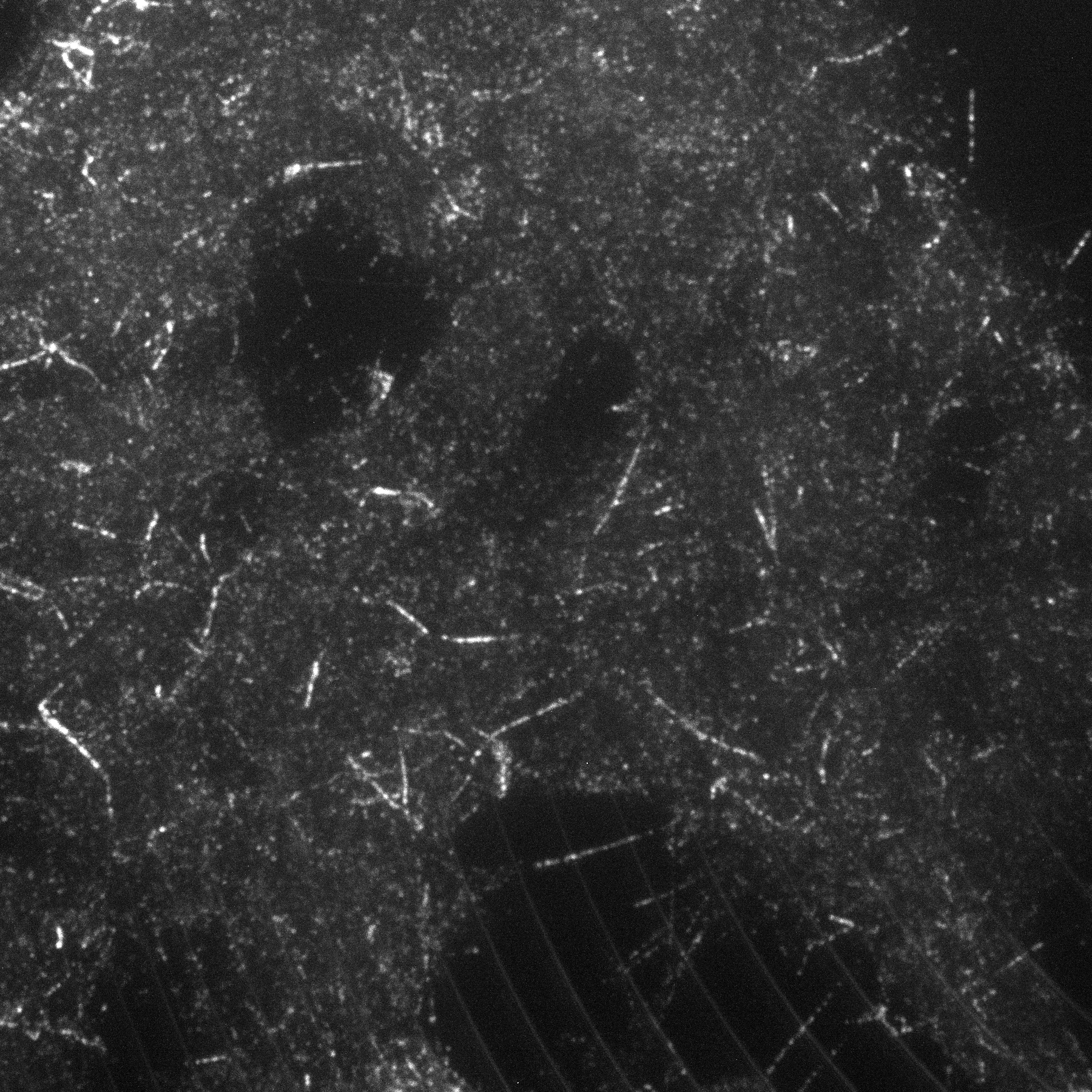

Supplement: Supplementary file 10 — Source data Fig. 4 [file 44318_2024_337_MOESM10_ESM.zip › 04_Figure_04/4G/FLAG-CTRL/_FULL-RANGE-FLAG-CTRL.tif]

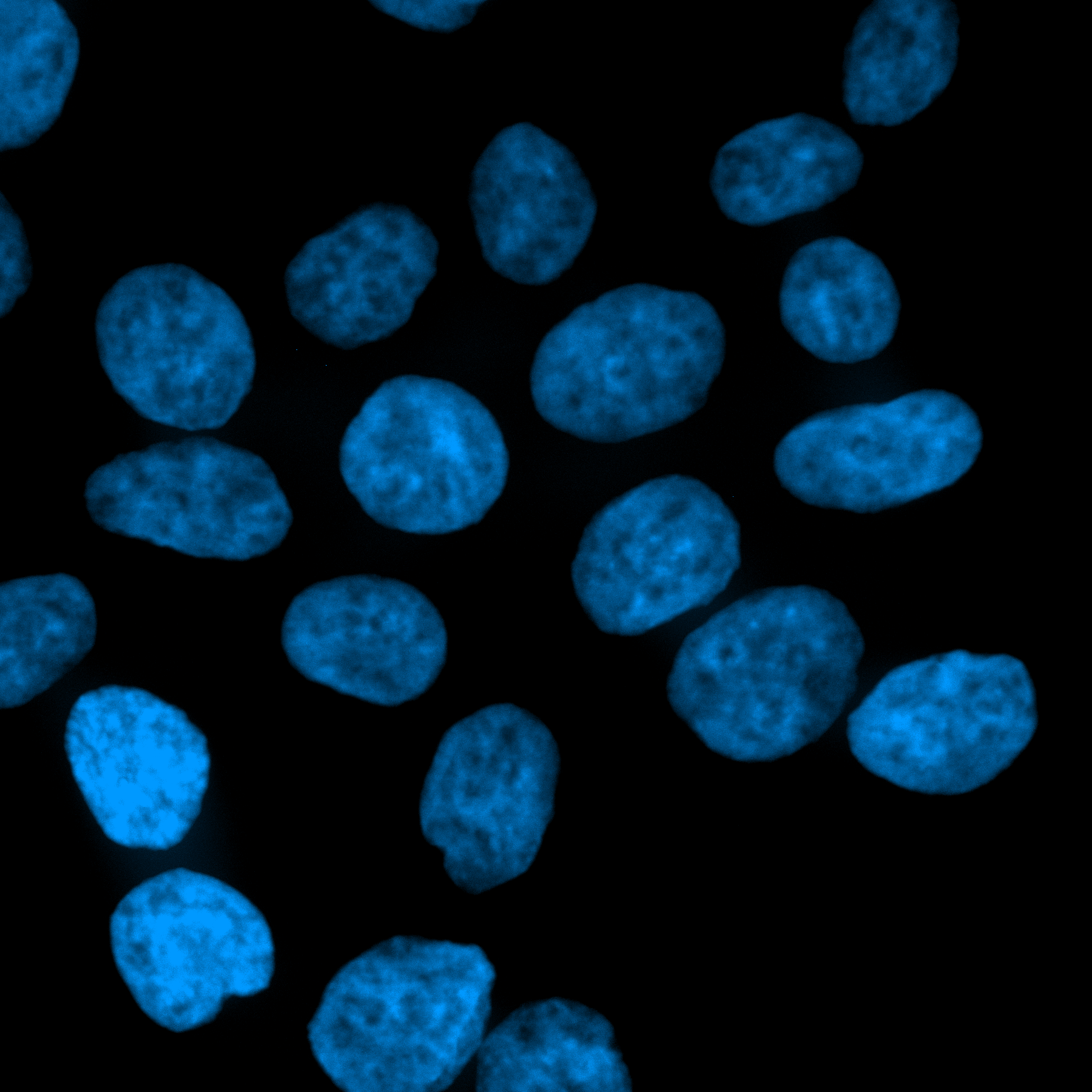

Supplement: Supplementary file 10 — Source data Fig. 4 [file 44318_2024_337_MOESM10_ESM.zip › 04_Figure_04/4G/HA/HA-DAPI.tif]

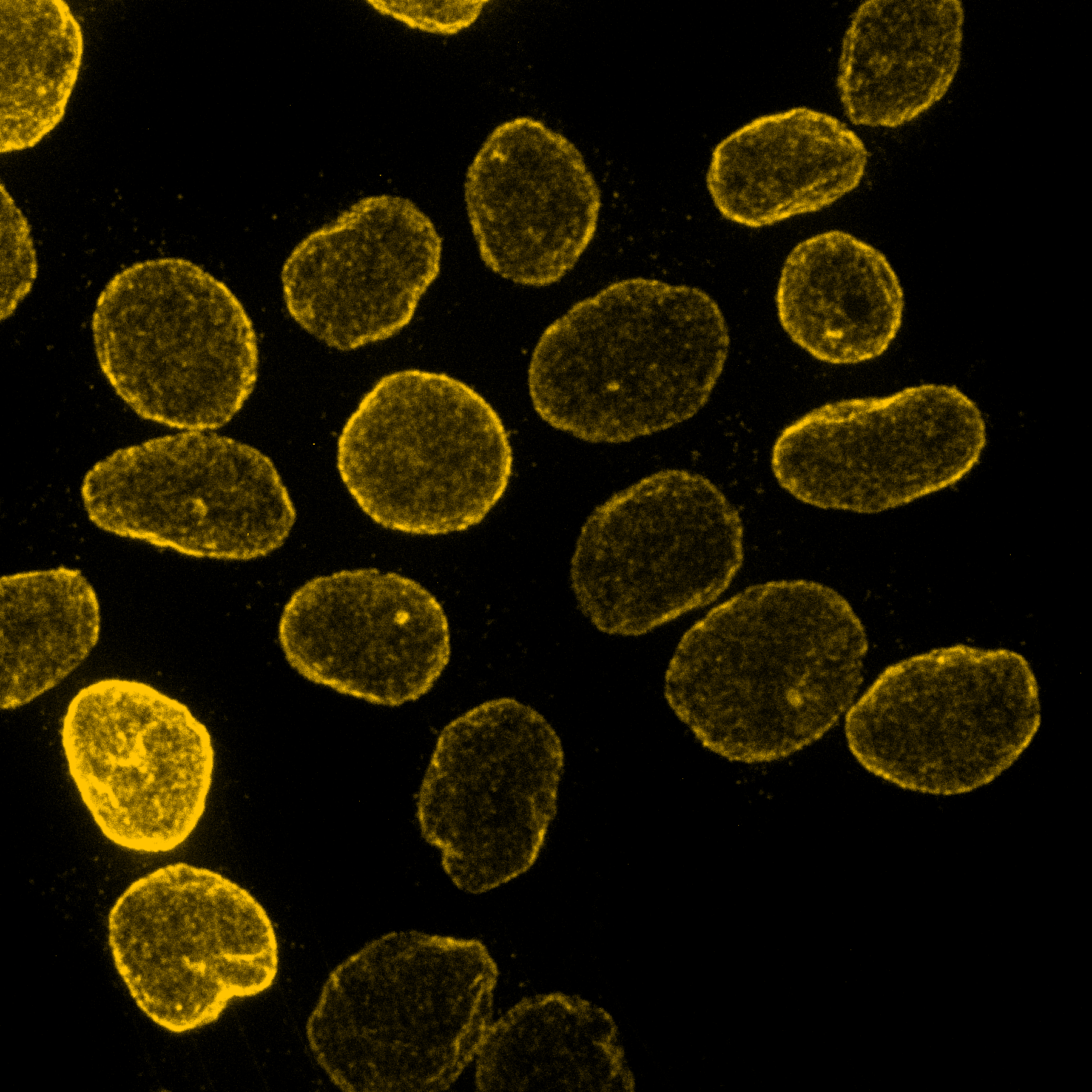

Supplement: Supplementary file 10 — Source data Fig. 4 [file 44318_2024_337_MOESM10_ESM.zip › 04_Figure_04/4G/HA/HA-HA.tif]

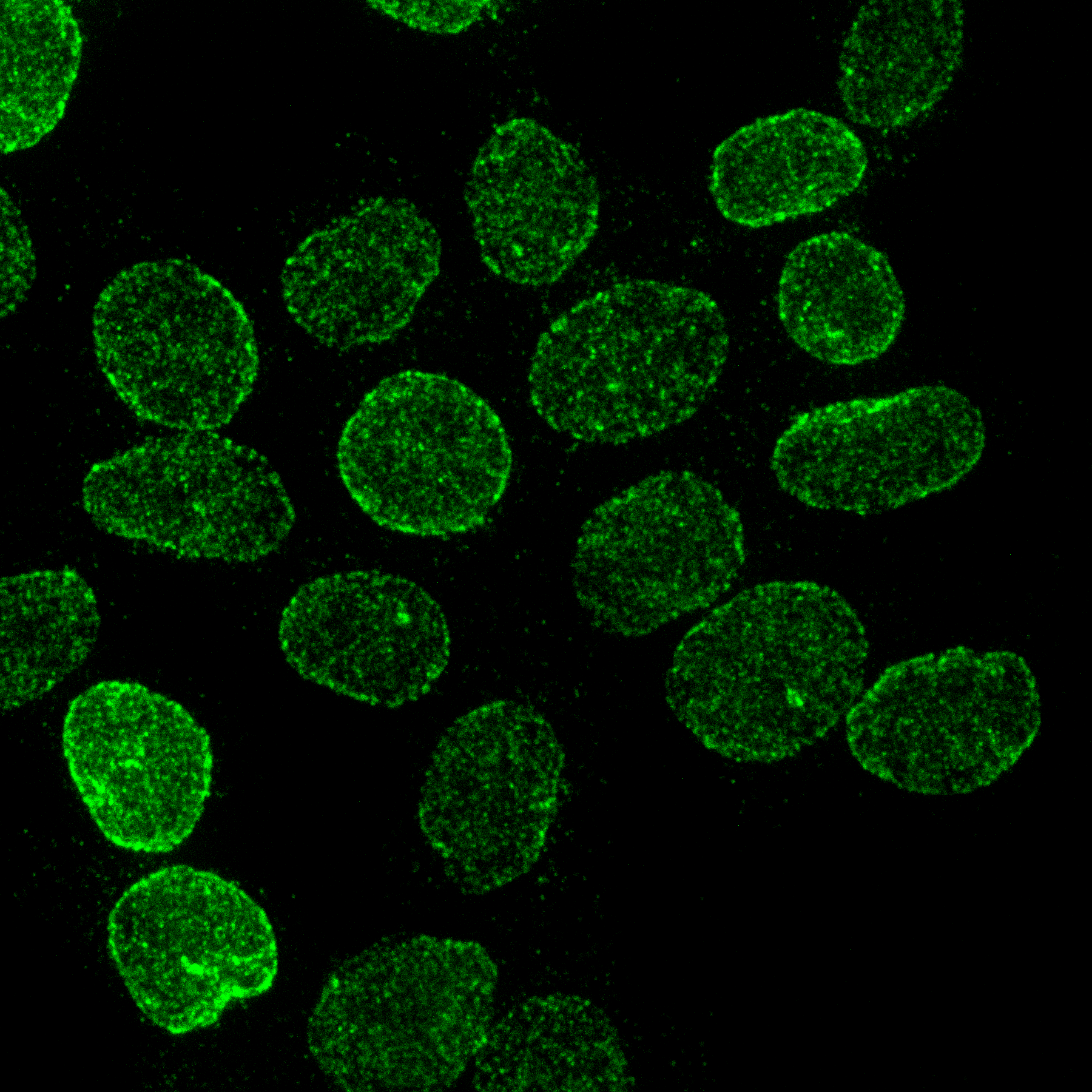

Supplement: Supplementary file 10 — Source data Fig. 4 [file 44318_2024_337_MOESM10_ESM.zip › 04_Figure_04/4G/HA/HA-LMNB1.tif]

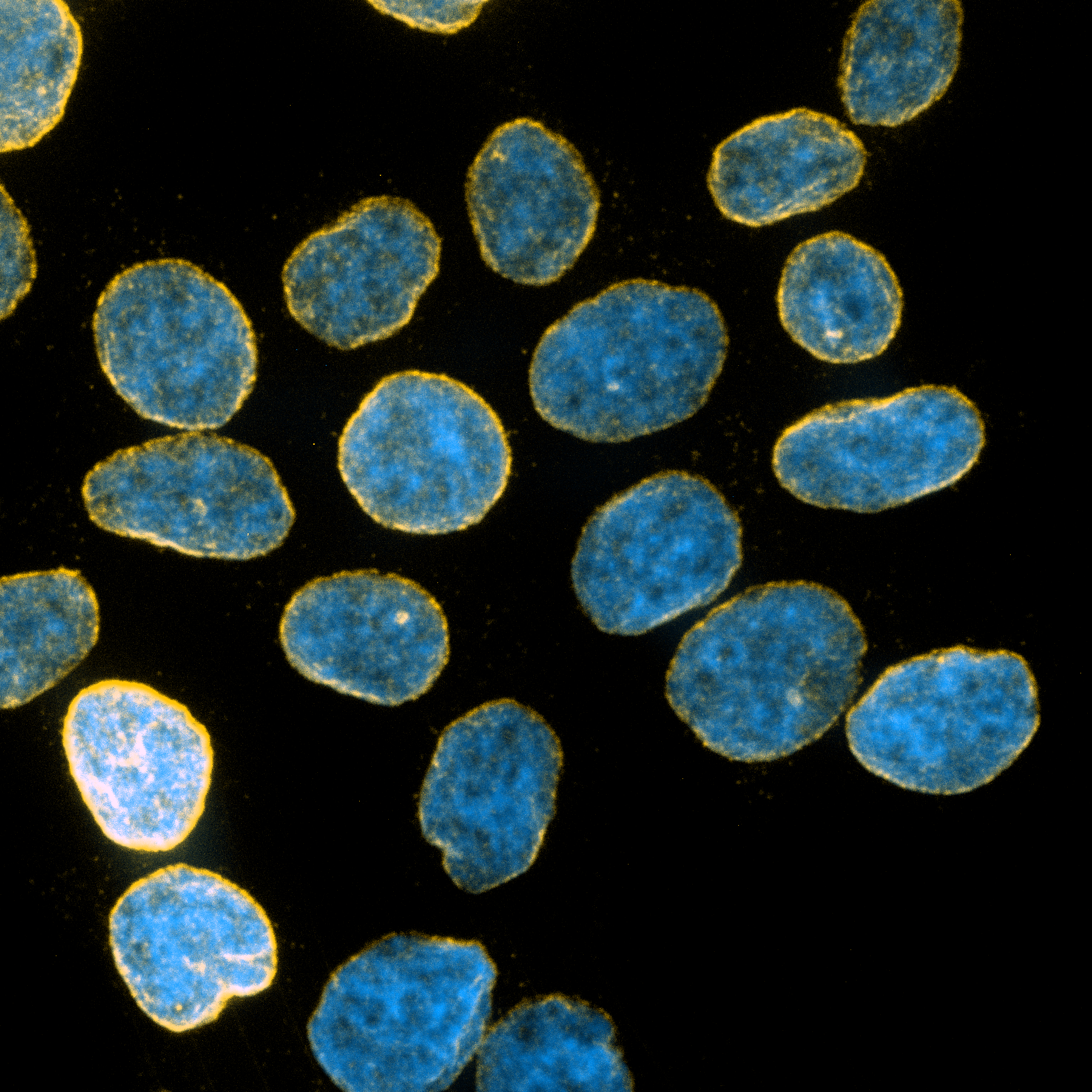

Supplement: Supplementary file 10 — Source data Fig. 4 [file 44318_2024_337_MOESM10_ESM.zip › 04_Figure_04/4G/HA/HA-Merge.tif]

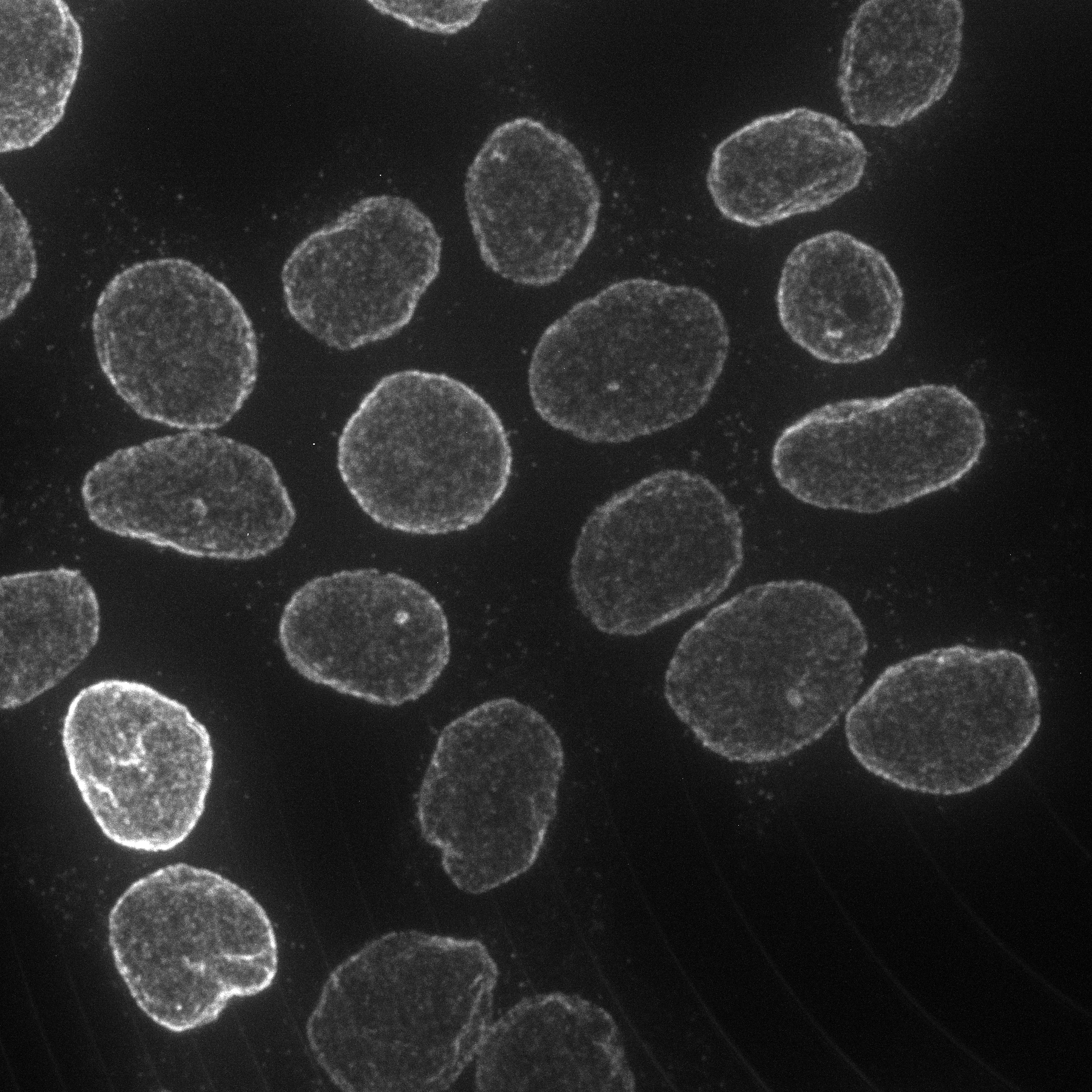

Supplement: Supplementary file 10 — Source data Fig. 4 [file 44318_2024_337_MOESM10_ESM.zip › 04_Figure_04/4G/HA/_FULL-RANGE-HA.tif]

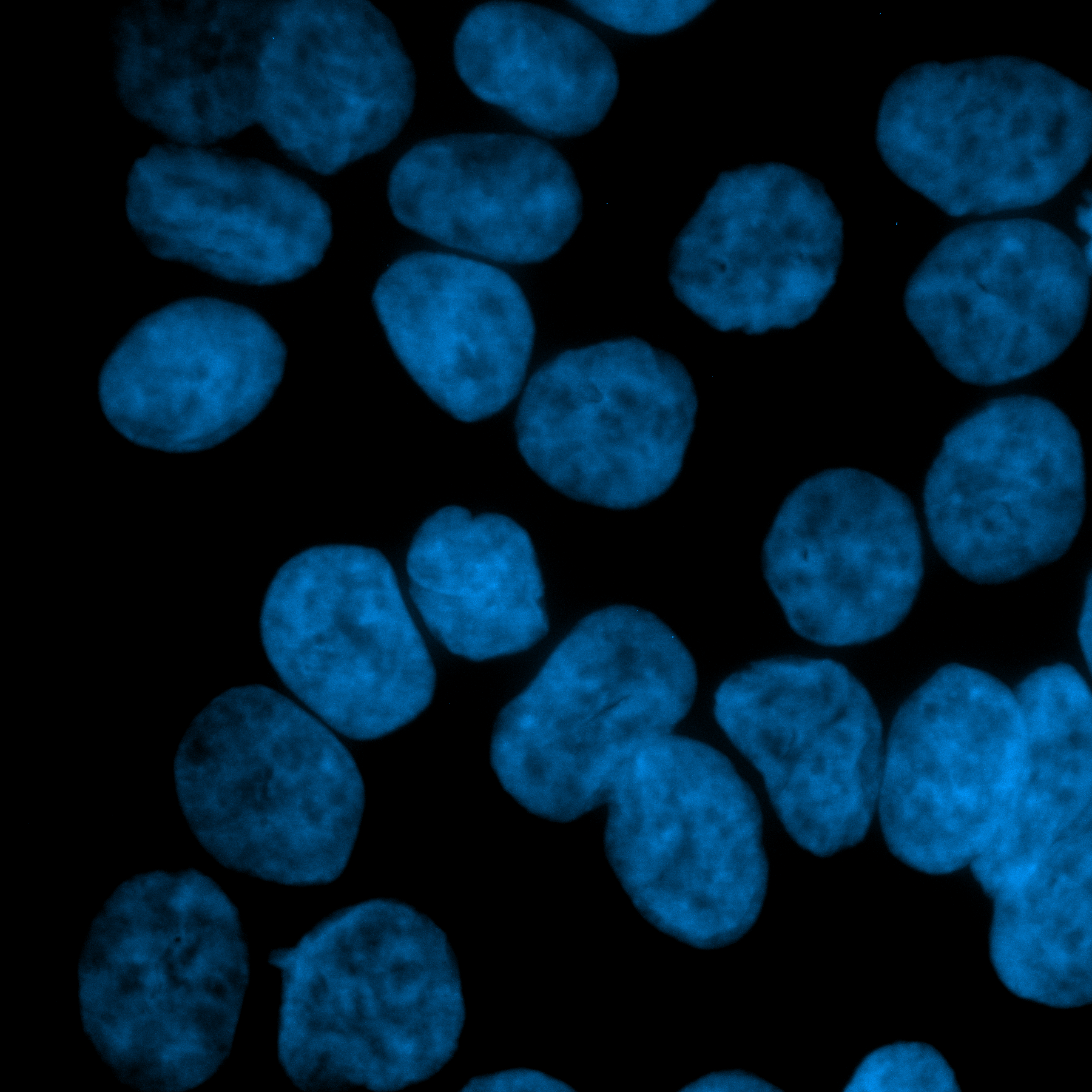

Supplement: Supplementary file 10 — Source data Fig. 4 [file 44318_2024_337_MOESM10_ESM.zip › 04_Figure_04/4G/HA-CTRL/HA-CTRL-DAPI.tif]

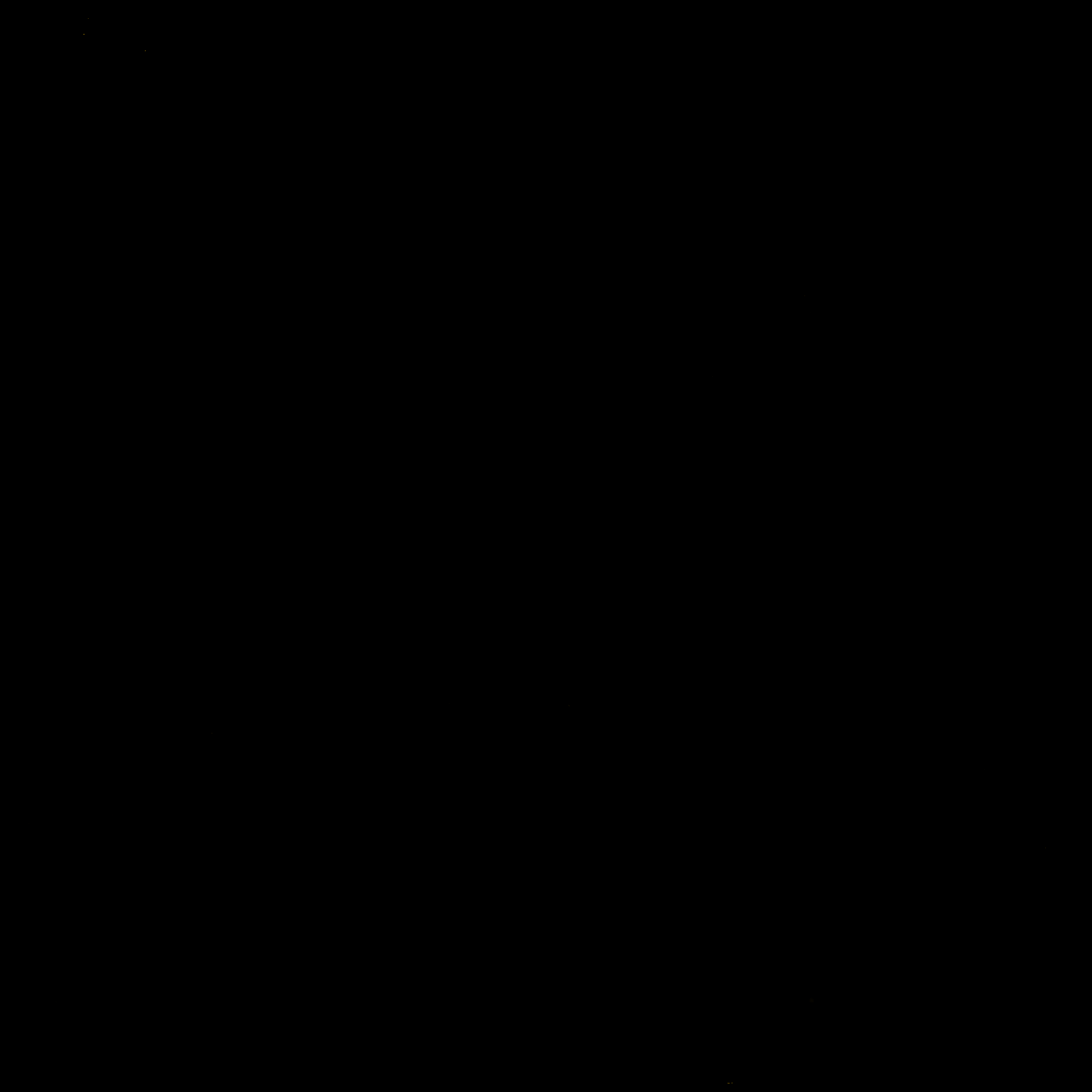

Supplement: Supplementary file 10 — Source data Fig. 4 [file 44318_2024_337_MOESM10_ESM.zip › 04_Figure_04/4G/HA-CTRL/HA-CTRL-HA.tif]

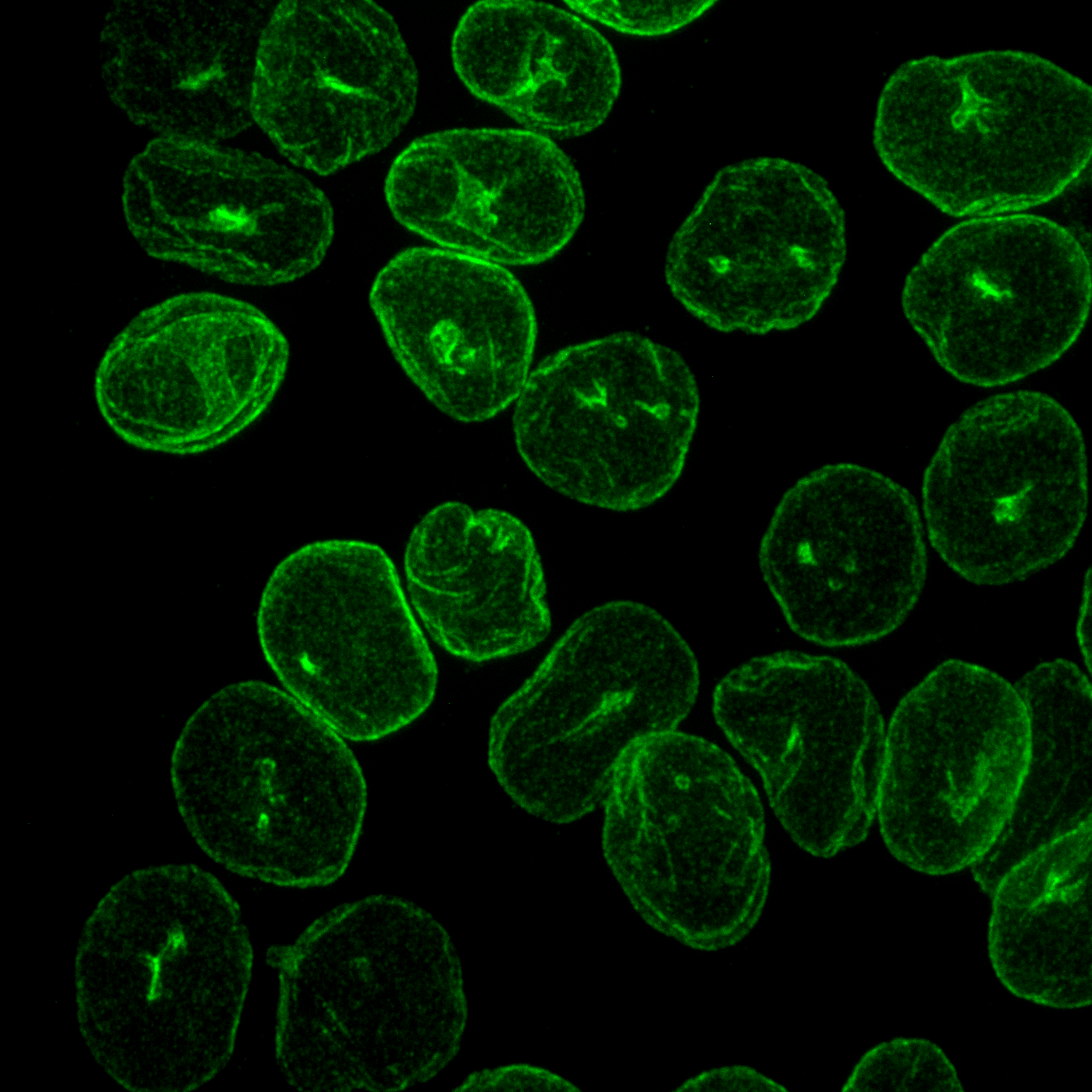

Supplement: Supplementary file 10 — Source data Fig. 4 [file 44318_2024_337_MOESM10_ESM.zip › 04_Figure_04/4G/HA-CTRL/HA-CTRL-LMNB1.tif]

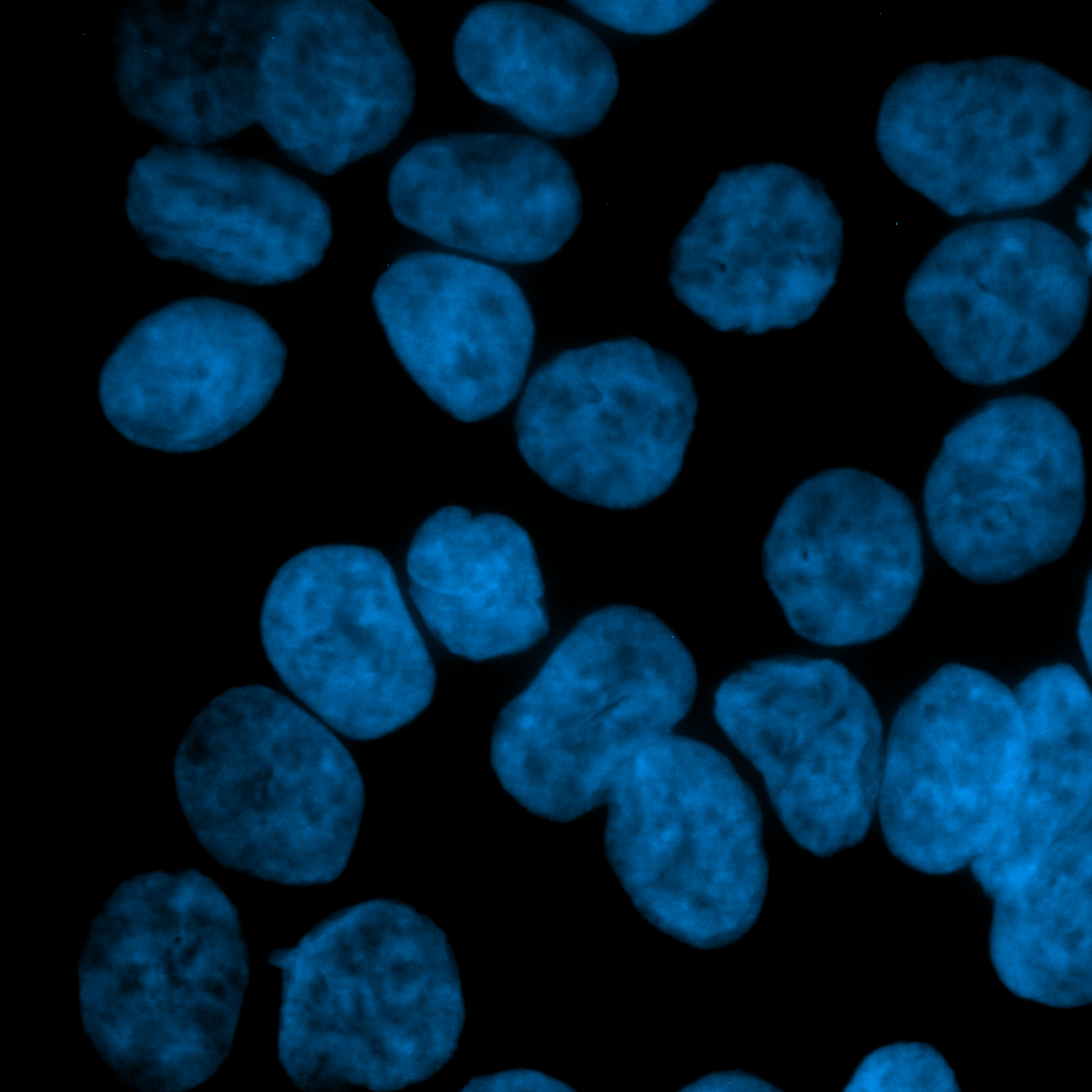

Supplement: Supplementary file 10 — Source data Fig. 4 [file 44318_2024_337_MOESM10_ESM.zip › 04_Figure_04/4G/HA-CTRL/HA-CTRL-Merge.tif]

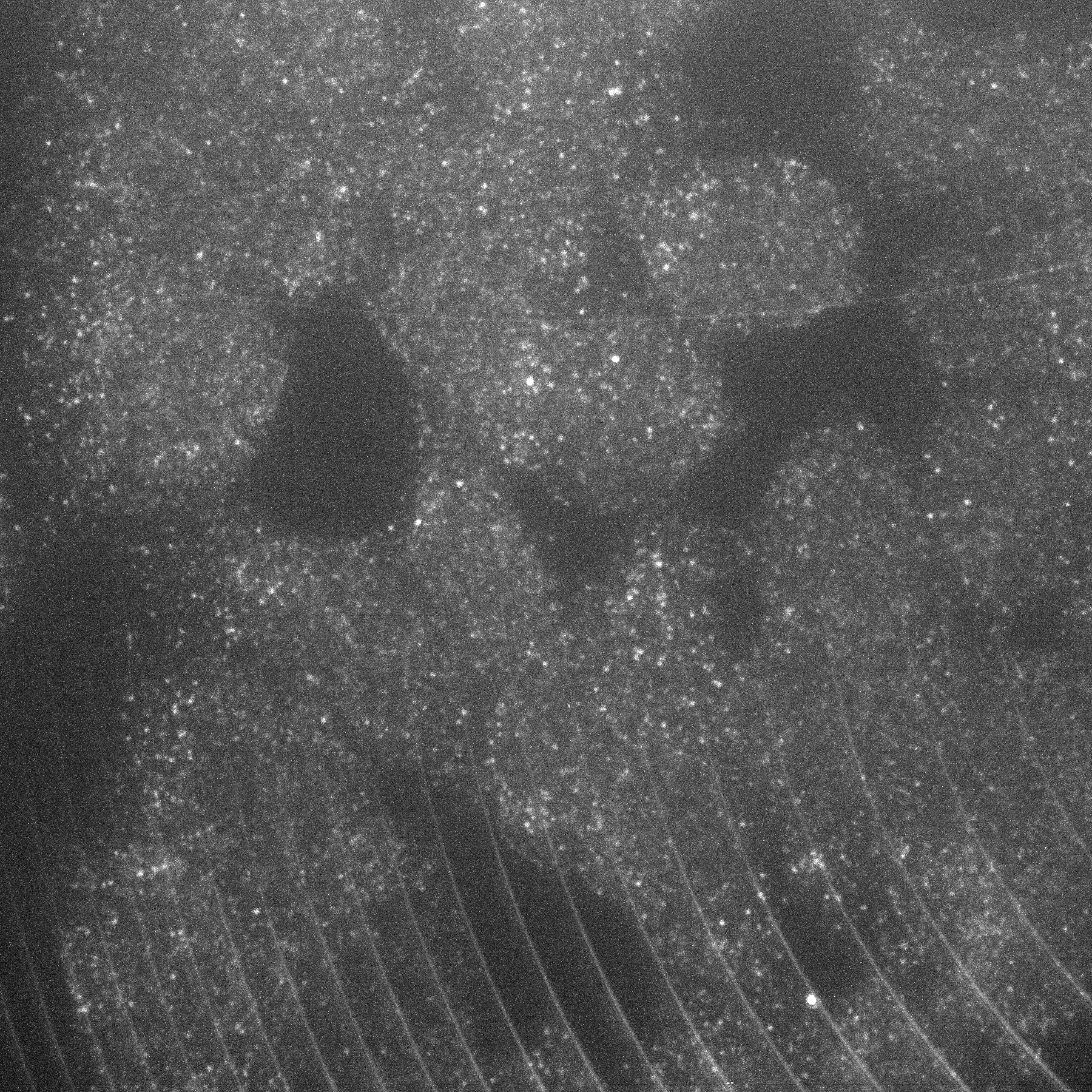

Supplement: Supplementary file 10 — Source data Fig. 4 [file 44318_2024_337_MOESM10_ESM.zip › 04_Figure_04/4G/HA-CTRL/_FULL-RANGE-HA-CTRL.tif]

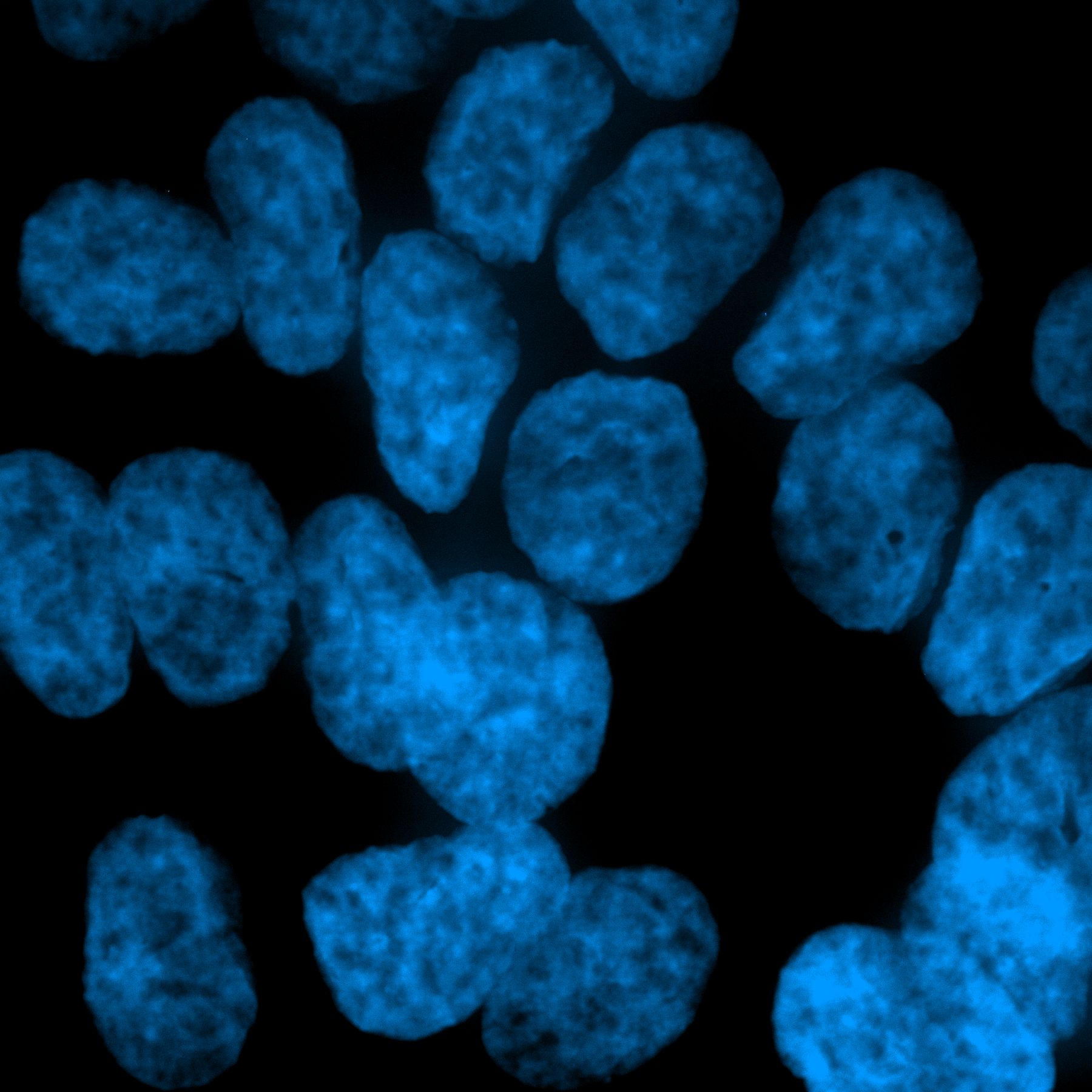

Supplement: Supplementary file 10 — Source data Fig. 4 [file 44318_2024_337_MOESM10_ESM.zip › 04_Figure_04/4G/V5/V5-DAPI.tif]

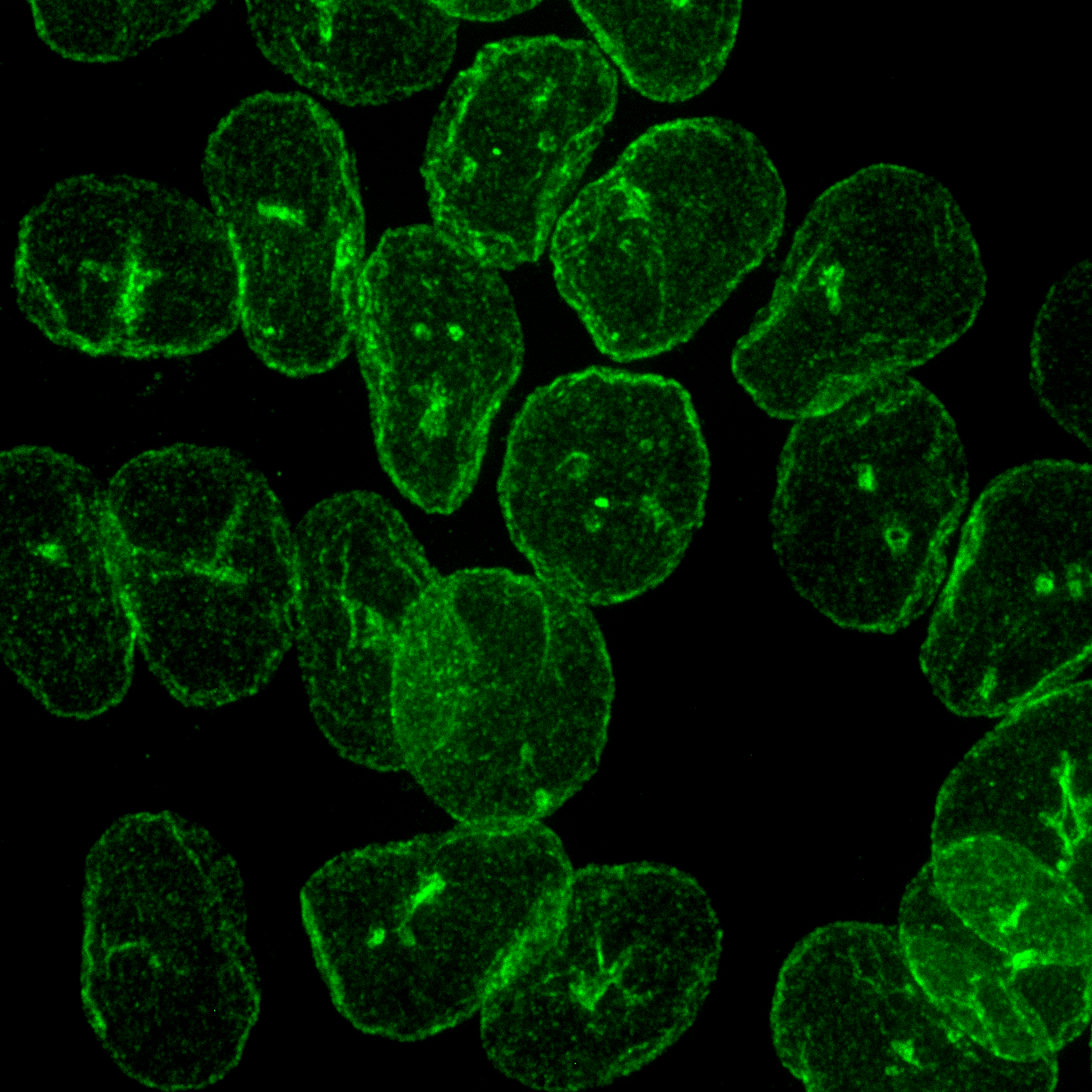

Supplement: Supplementary file 10 — Source data Fig. 4 [file 44318_2024_337_MOESM10_ESM.zip › 04_Figure_04/4G/V5/V5-LMNB1.tif]

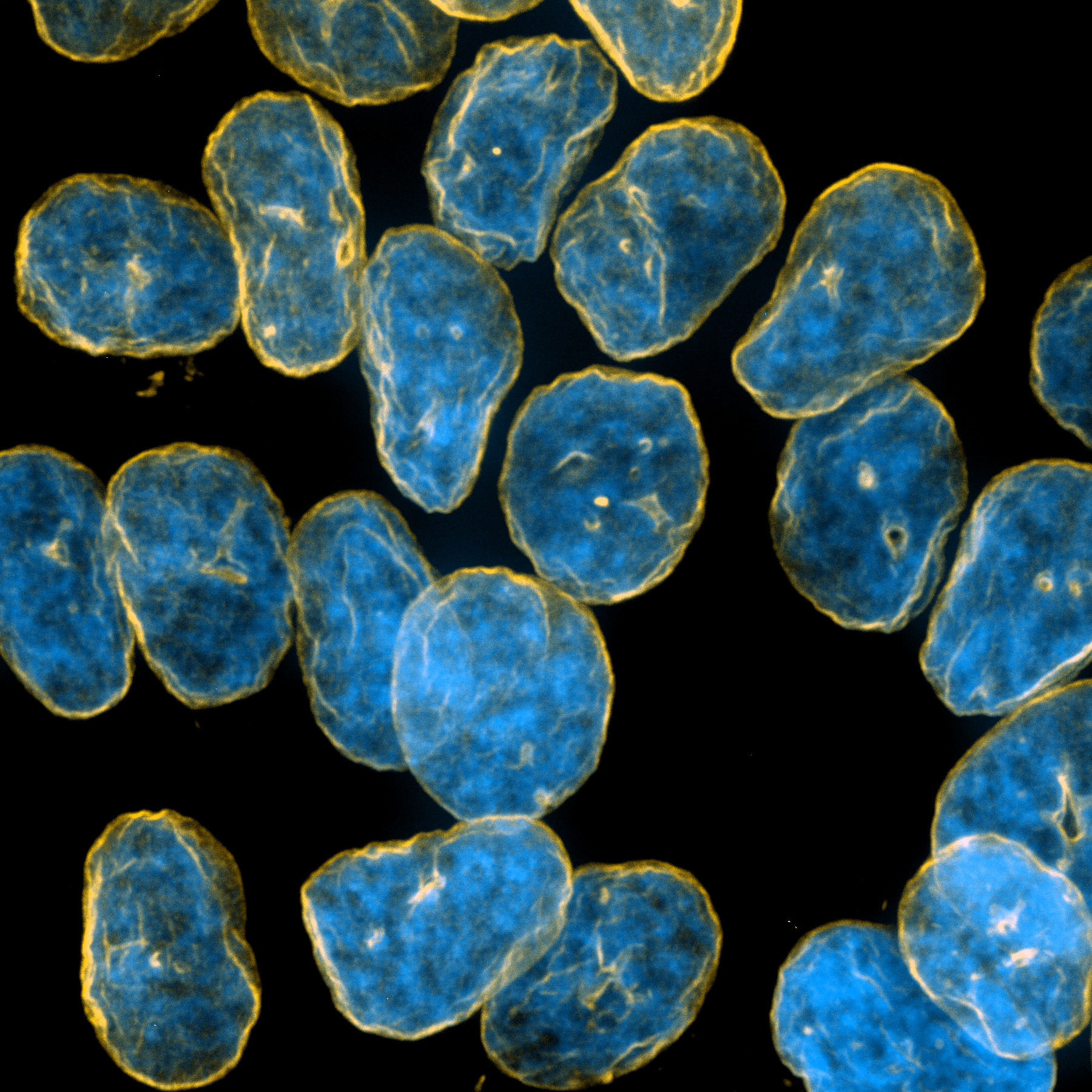

Supplement: Supplementary file 10 — Source data Fig. 4 [file 44318_2024_337_MOESM10_ESM.zip › 04_Figure_04/4G/V5/V5-Merge.tif]

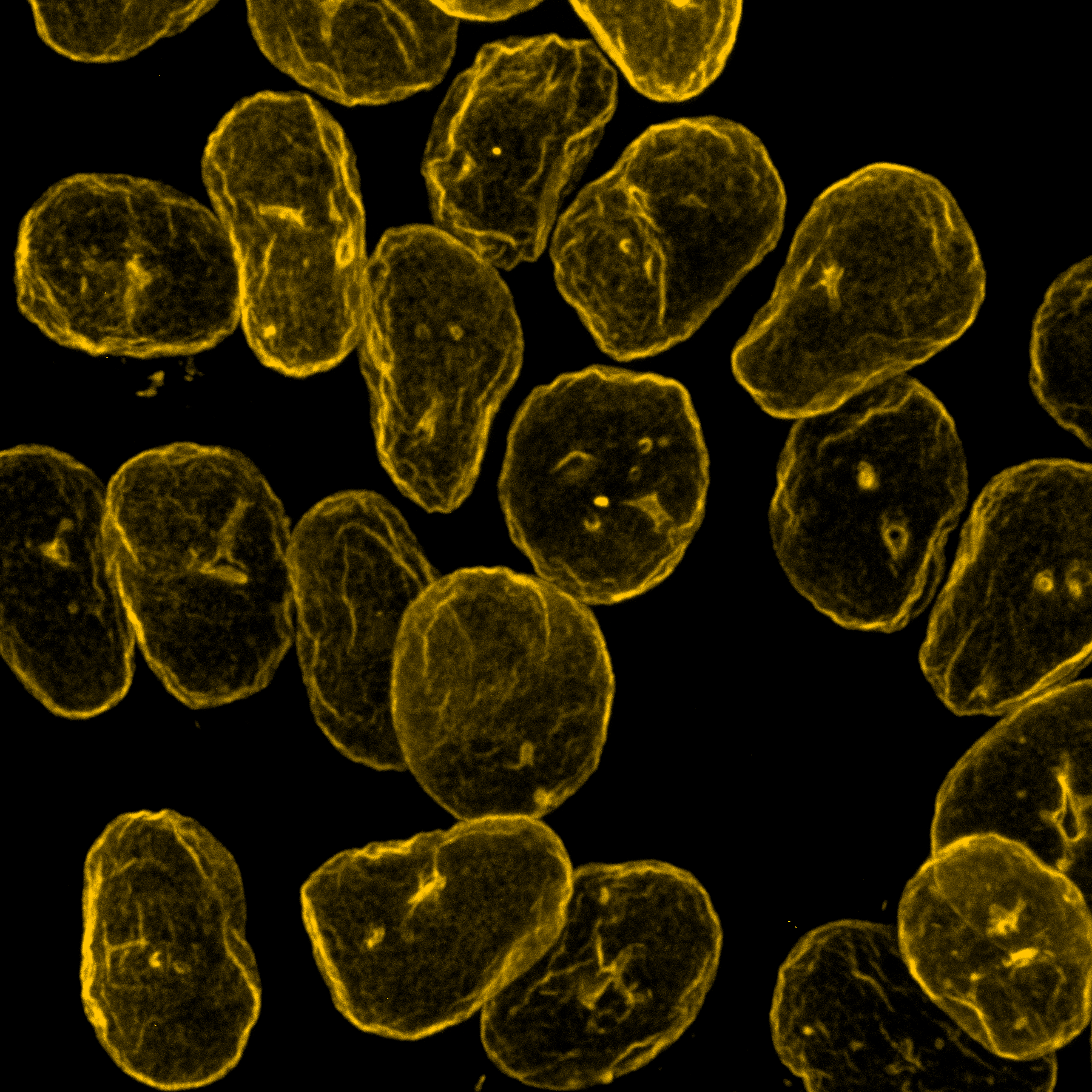

Supplement: Supplementary file 10 — Source data Fig. 4 [file 44318_2024_337_MOESM10_ESM.zip › 04_Figure_04/4G/V5/V5-V5.tif]

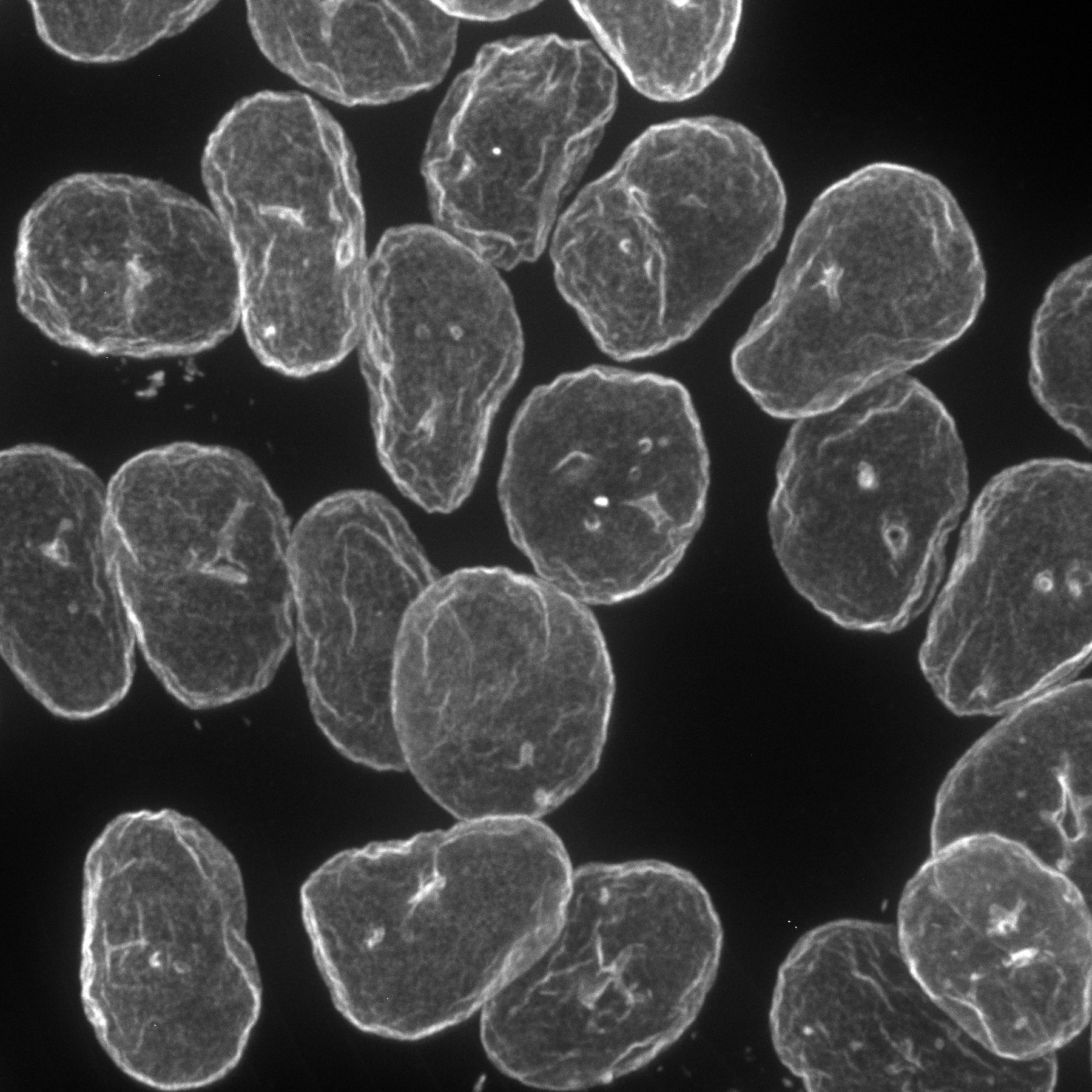

Supplement: Supplementary file 10 — Source data Fig. 4 [file 44318_2024_337_MOESM10_ESM.zip › 04_Figure_04/4G/V5/_FULL-RANGE-V5.tif]

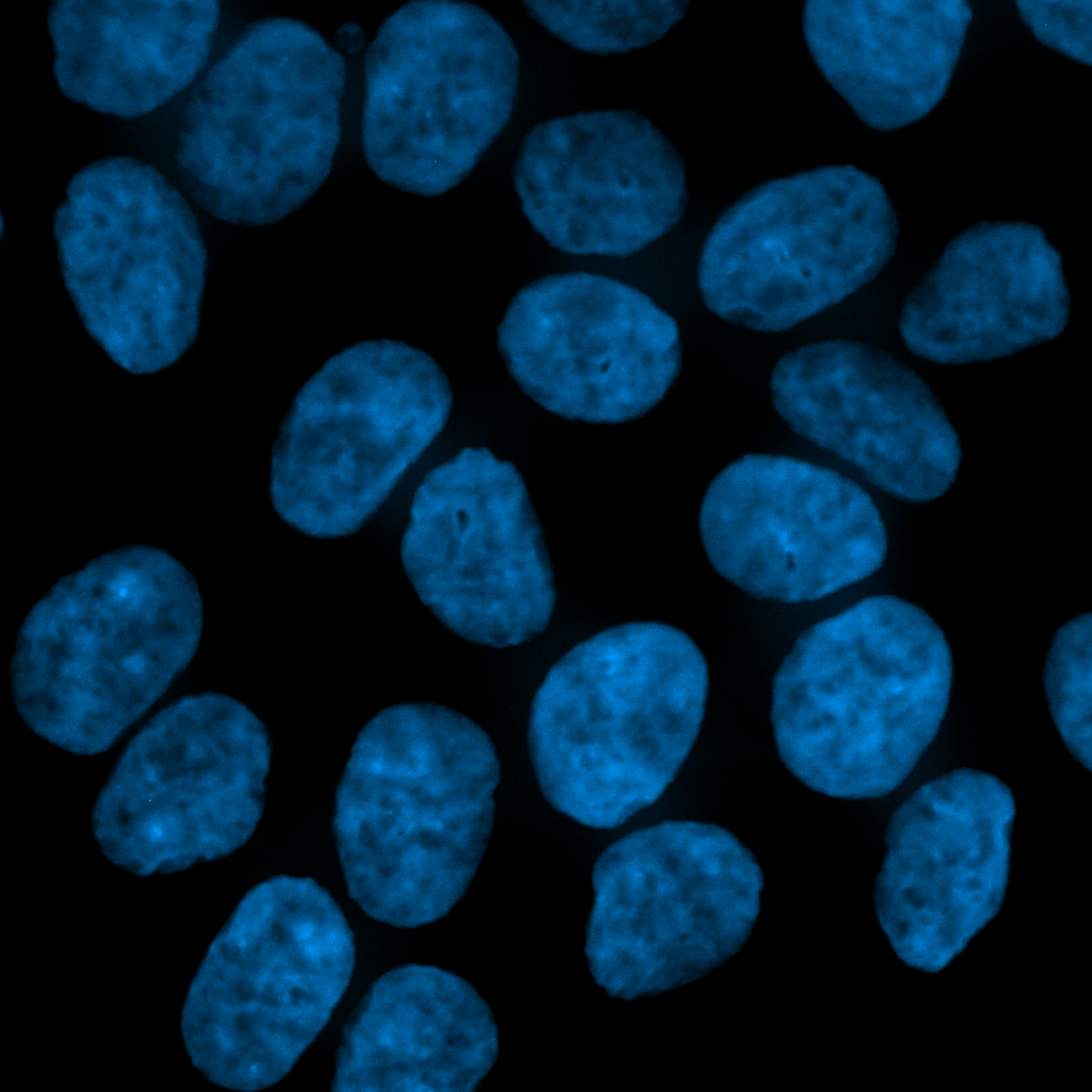

Supplement: Supplementary file 10 — Source data Fig. 4 [file 44318_2024_337_MOESM10_ESM.zip › 04_Figure_04/4G/V5-CTRL/V5-CTRL-DAPI.tif]

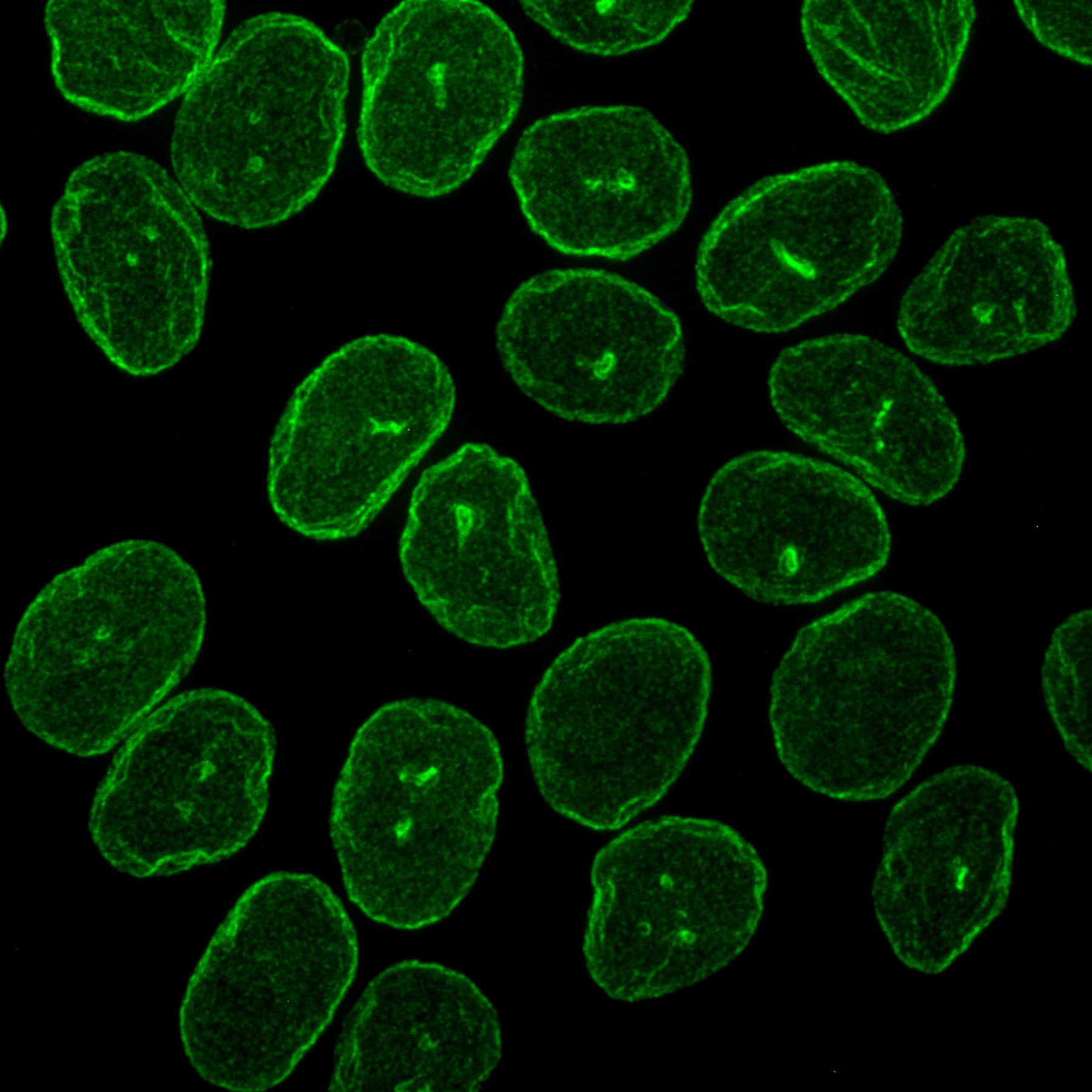

Supplement: Supplementary file 10 — Source data Fig. 4 [file 44318_2024_337_MOESM10_ESM.zip › 04_Figure_04/4G/V5-CTRL/V5-CTRL-LMNB1.tif]

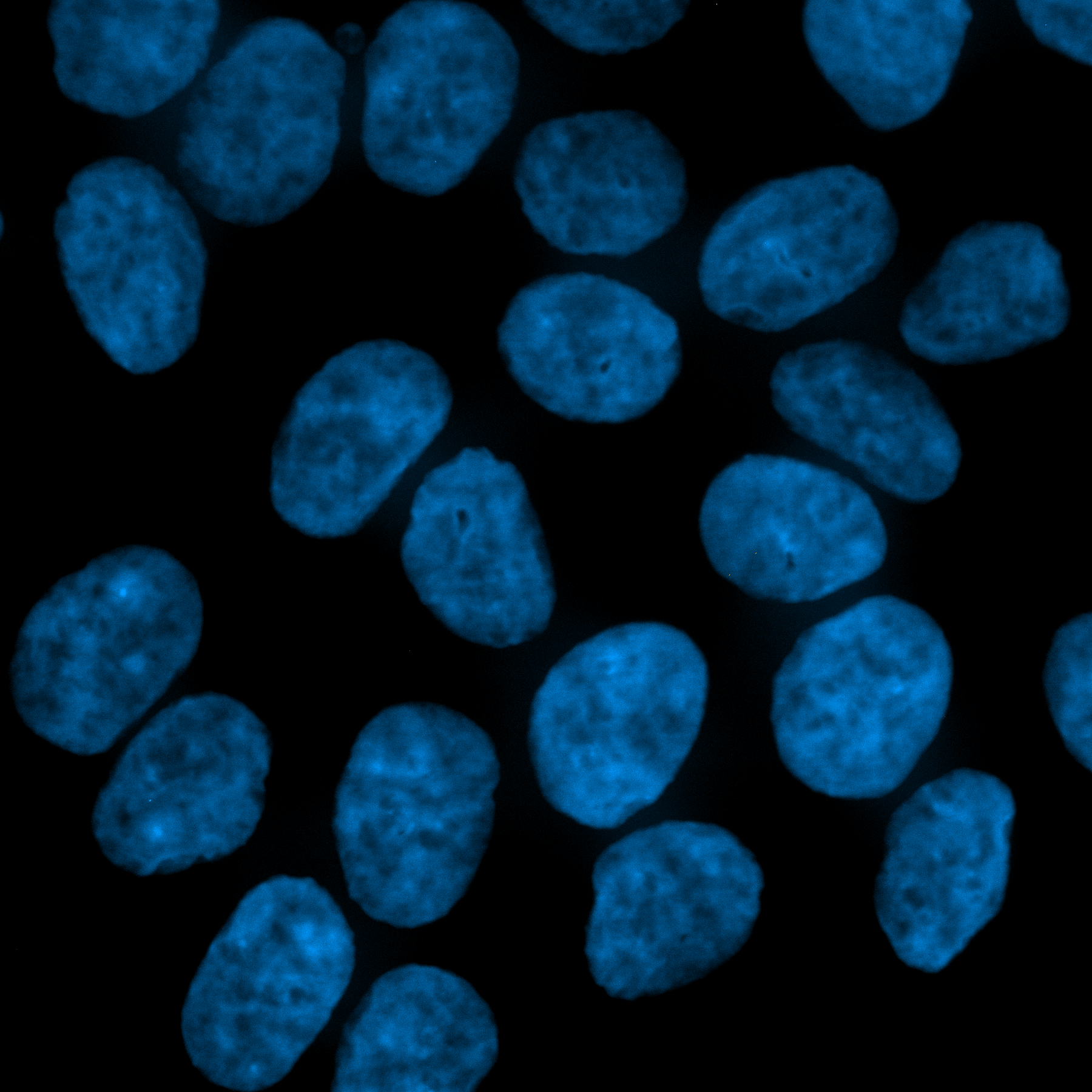

Supplement: Supplementary file 10 — Source data Fig. 4 [file 44318_2024_337_MOESM10_ESM.zip › 04_Figure_04/4G/V5-CTRL/V5-CTRL-Merge.tif]

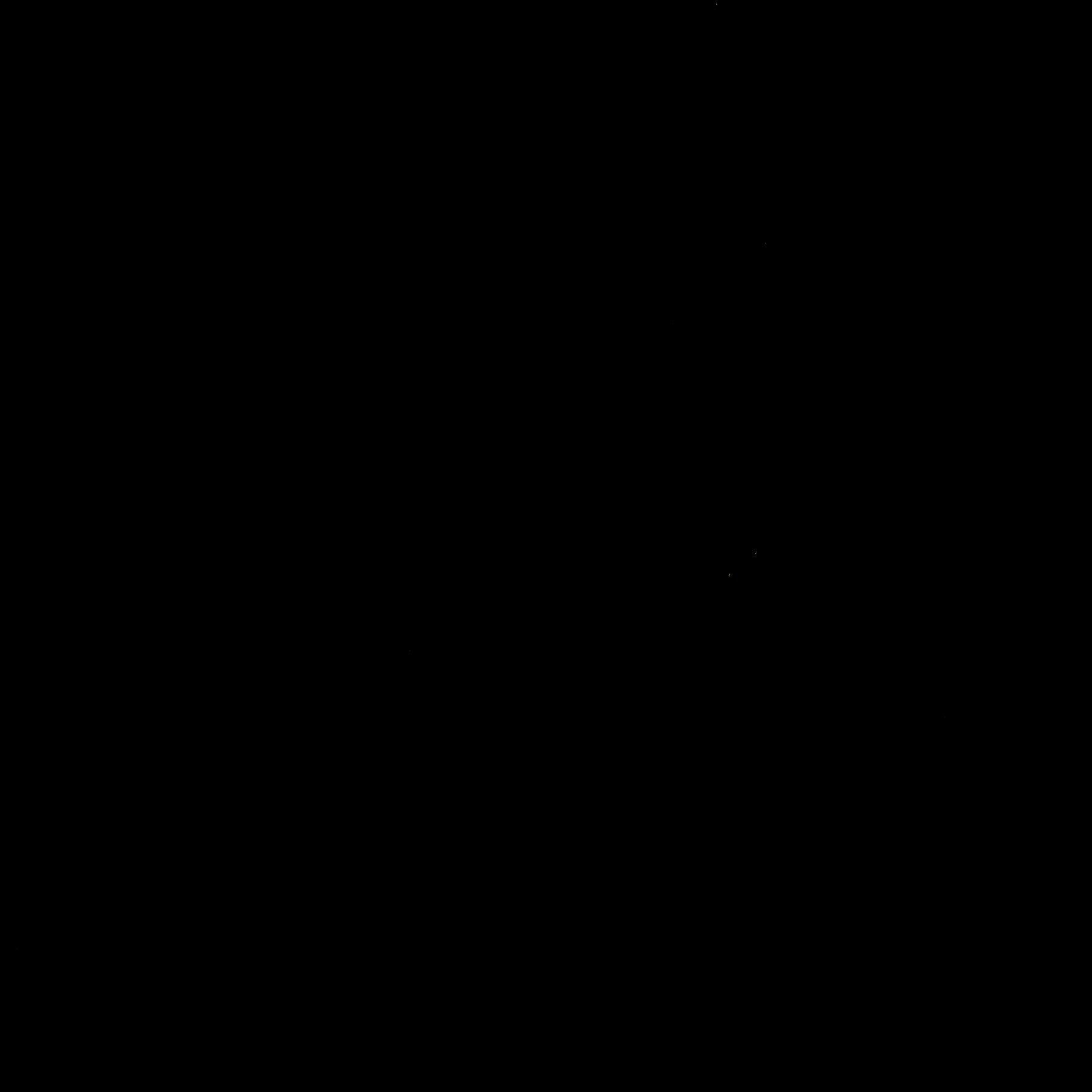

Supplement: Supplementary file 10 — Source data Fig. 4 [file 44318_2024_337_MOESM10_ESM.zip › 04_Figure_04/4G/V5-CTRL/V5-CTRL-V5.tif]

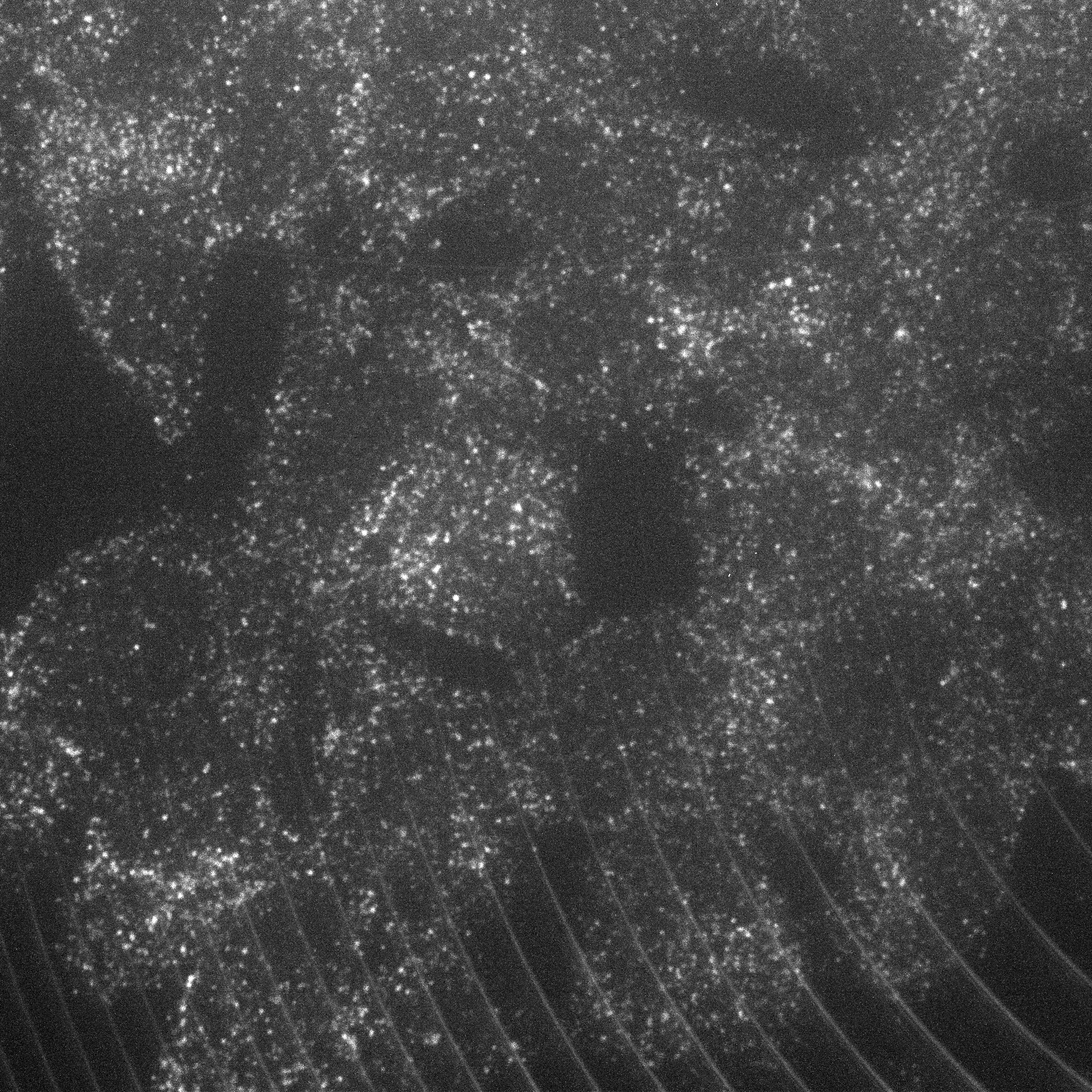

Supplement: Supplementary file 10 — Source data Fig. 4 [file 44318_2024_337_MOESM10_ESM.zip › 04_Figure_04/4G/V5-CTRL/_FULL-RANGE-V5-CTRL.tif]

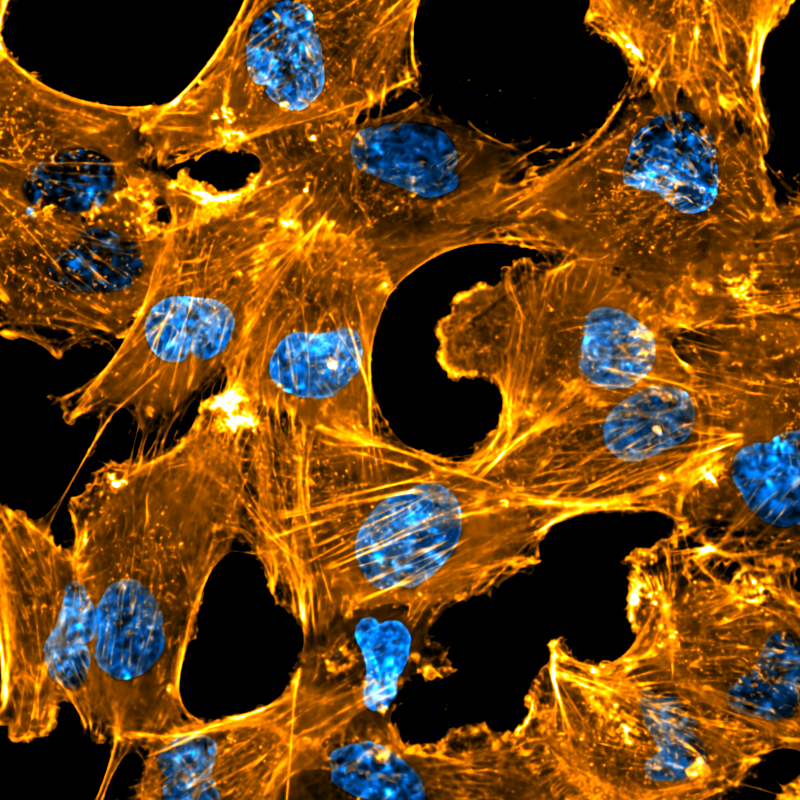

Supplement: Supplementary file 11 — Source data Fig. 5 [file 44318_2024_337_MOESM11_ESM.zip › 05_Figure_05/5B/Images/mNeon-0min.tif]

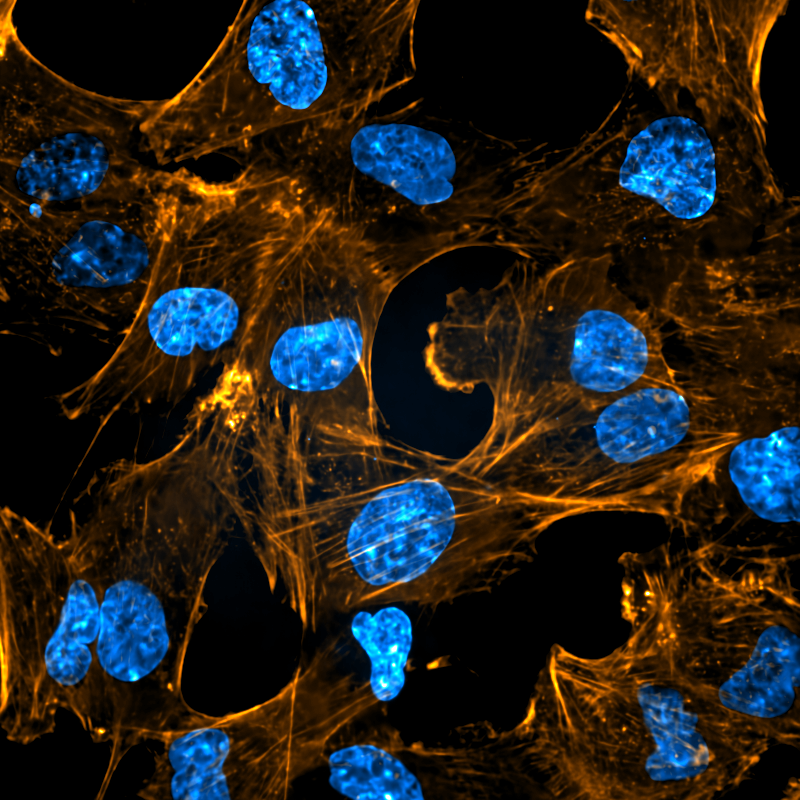

Supplement: Supplementary file 11 — Source data Fig. 5 [file 44318_2024_337_MOESM11_ESM.zip › 05_Figure_05/5B/Images/mNeon-10min.tif]

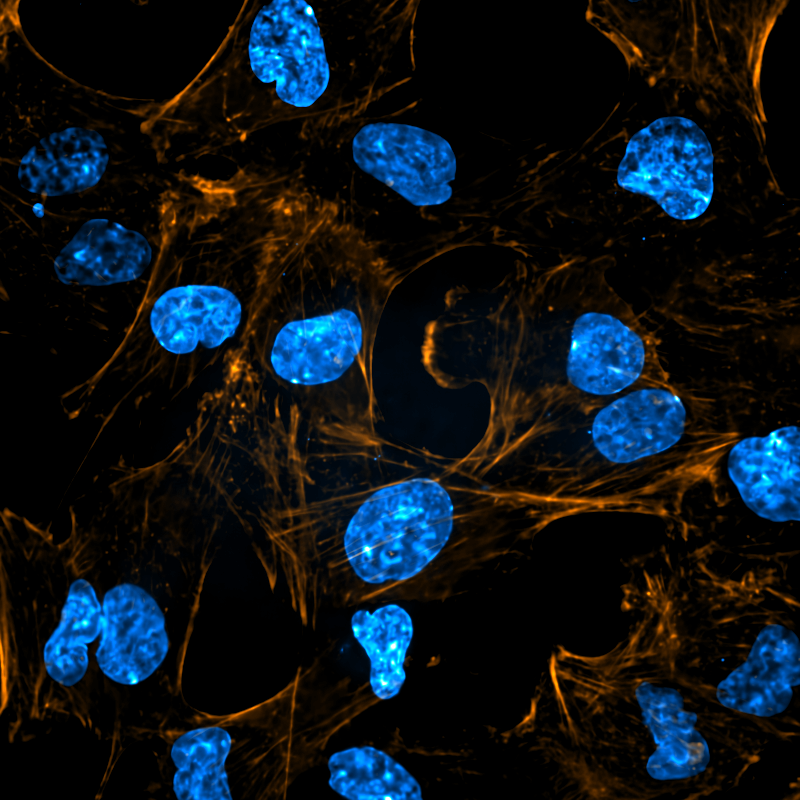

Supplement: Supplementary file 11 — Source data Fig. 5 [file 44318_2024_337_MOESM11_ESM.zip › 05_Figure_05/5B/Images/mNeon-15min.tif]

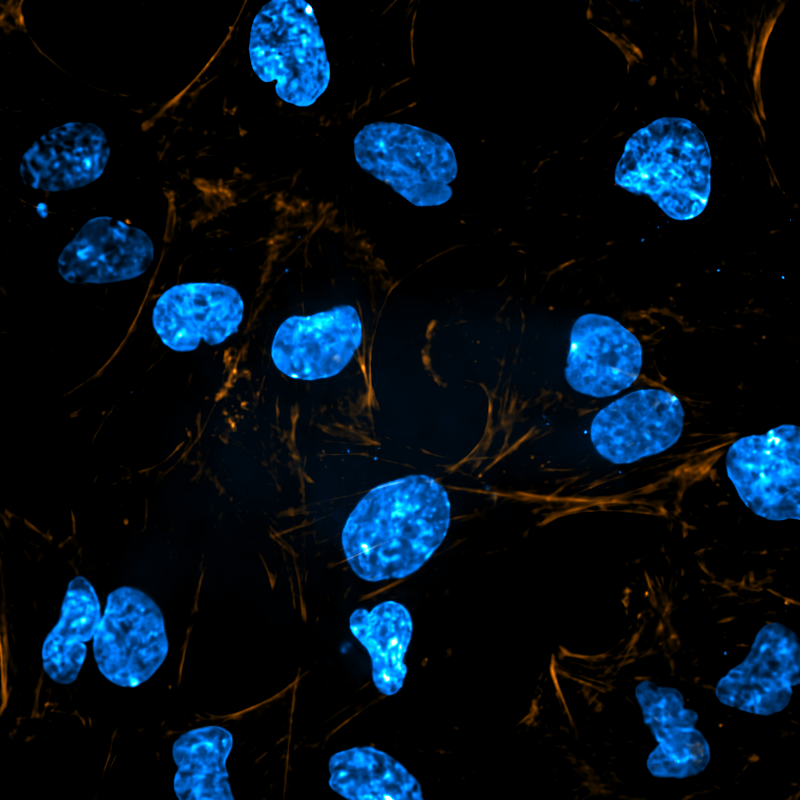

Supplement: Supplementary file 11 — Source data Fig. 5 [file 44318_2024_337_MOESM11_ESM.zip › 05_Figure_05/5B/Images/mNeon-20min.tif]

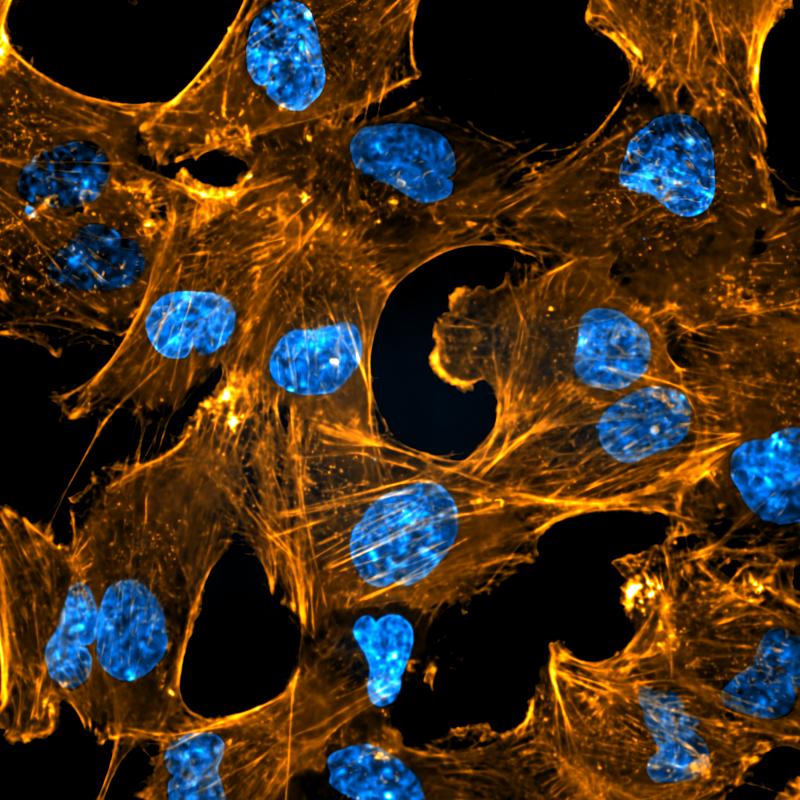

Supplement: Supplementary file 11 — Source data Fig. 5 [file 44318_2024_337_MOESM11_ESM.zip › 05_Figure_05/5B/Images/mNeon-5min.tif]

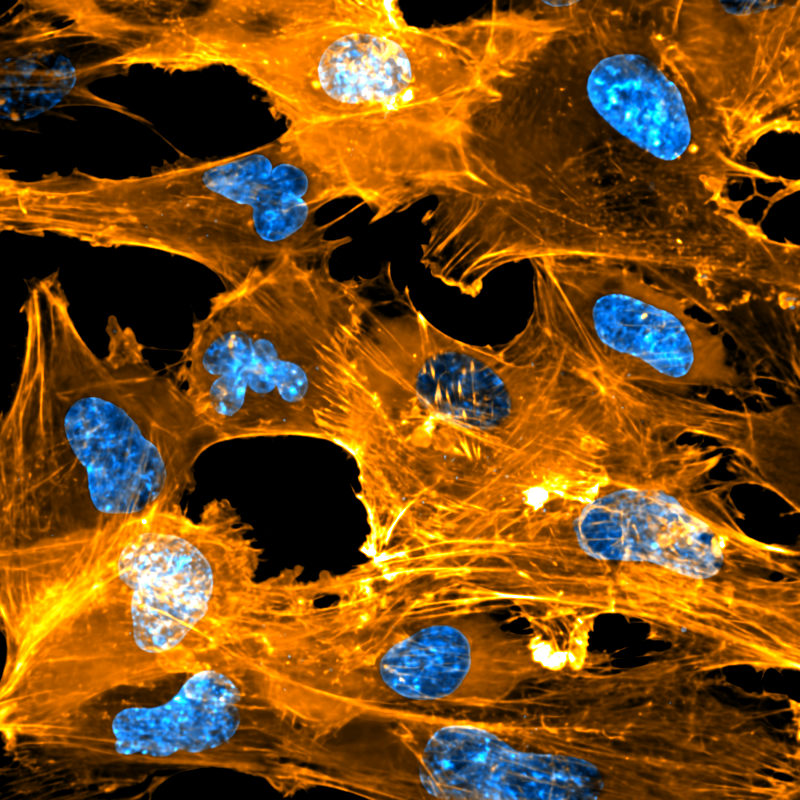

Supplement: Supplementary file 11 — Source data Fig. 5 [file 44318_2024_337_MOESM11_ESM.zip › 05_Figure_05/5B/Images/mStayGold-0min.tif]

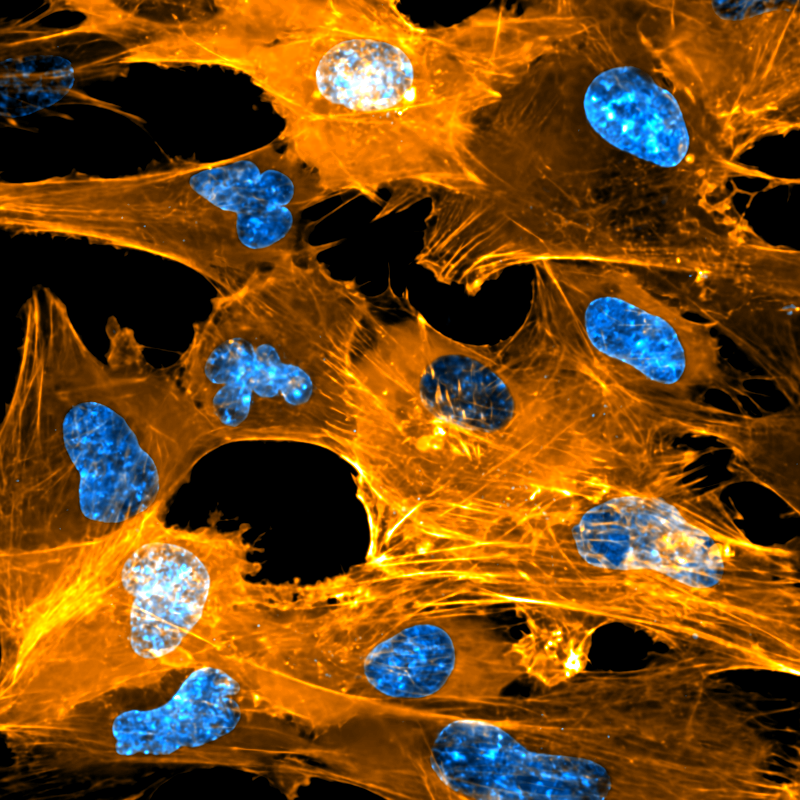

Supplement: Supplementary file 11 — Source data Fig. 5 [file 44318_2024_337_MOESM11_ESM.zip › 05_Figure_05/5B/Images/mStayGold-10min.tif]

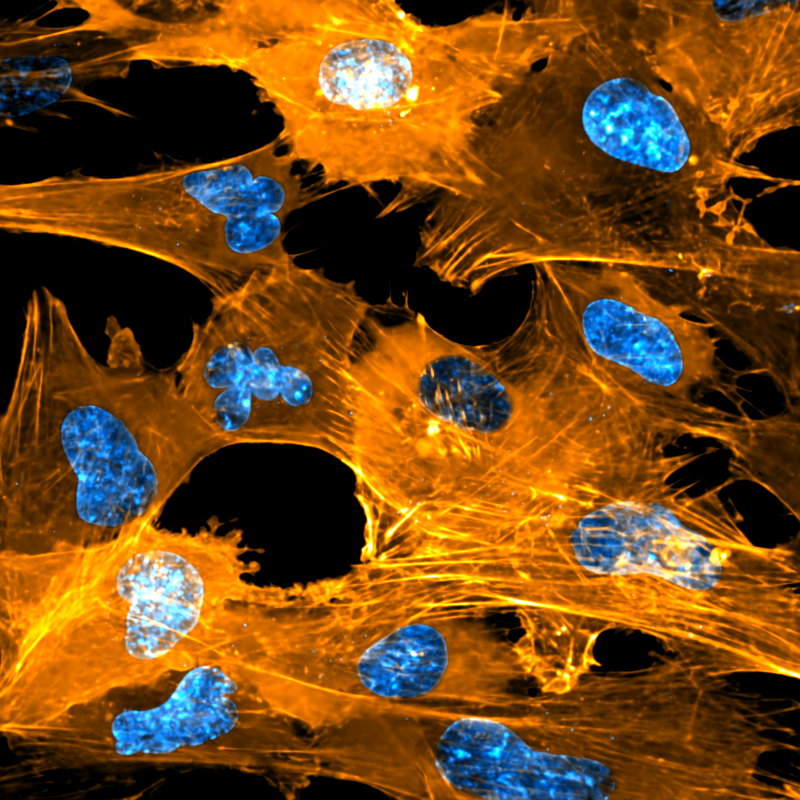

Supplement: Supplementary file 11 — Source data Fig. 5 [file 44318_2024_337_MOESM11_ESM.zip › 05_Figure_05/5B/Images/mStayGold-15min.tif]

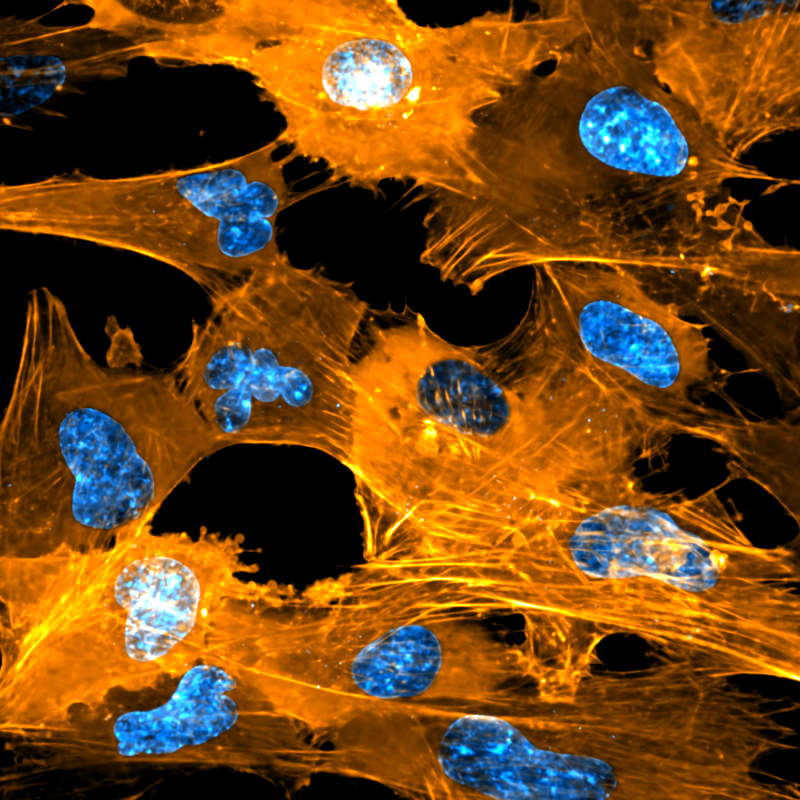

Supplement: Supplementary file 11 — Source data Fig. 5 [file 44318_2024_337_MOESM11_ESM.zip › 05_Figure_05/5B/Images/mStayGold-20min.tif]

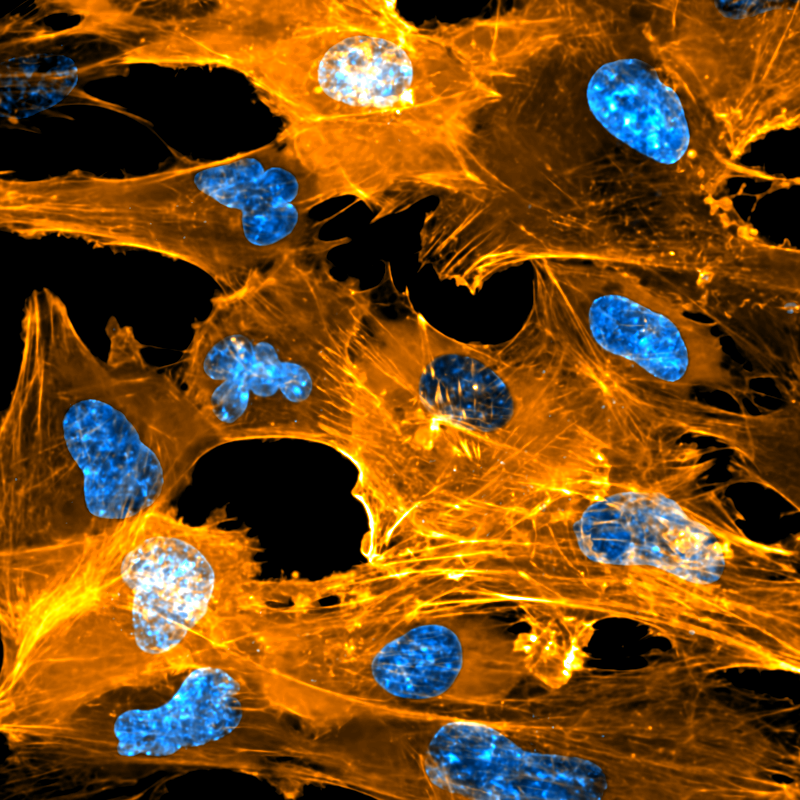

Supplement: Supplementary file 11 — Source data Fig. 5 [file 44318_2024_337_MOESM11_ESM.zip › 05_Figure_05/5B/Images/mStayGold-5min.tif]

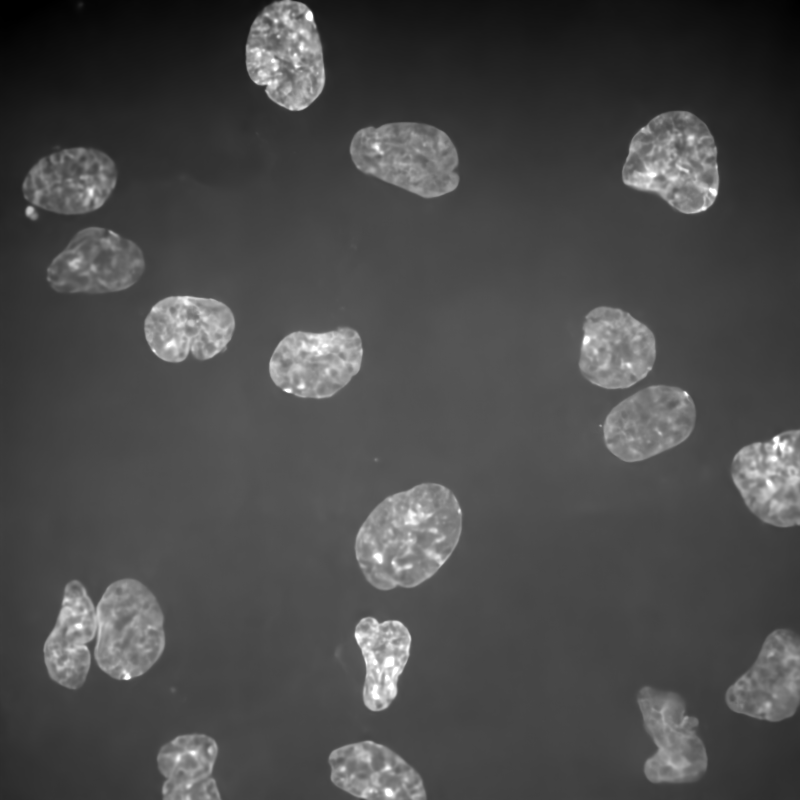

Supplement: Supplementary file 11 — Source data Fig. 5 [file 44318_2024_337_MOESM11_ESM.zip › 05_Figure_05/5B/Images/_FULL-RANGE-mNeon.tif]

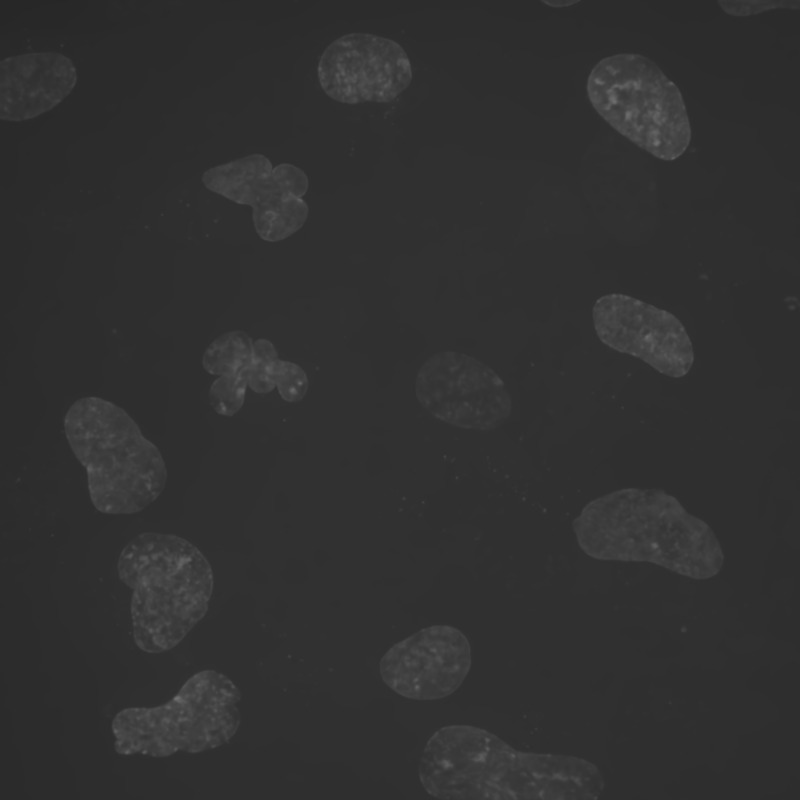

Supplement: Supplementary file 11 — Source data Fig. 5 [file 44318_2024_337_MOESM11_ESM.zip › 05_Figure_05/5B/Images/_FULL-RANGE-mStayGold.tif]

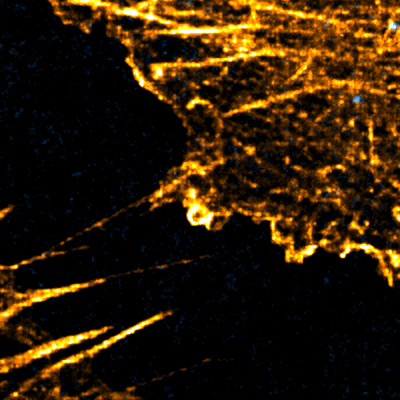

Supplement: Supplementary file 11 — Source data Fig. 5 [file 44318_2024_337_MOESM11_ESM.zip › 05_Figure_05/5C/SIM Time Course/SIM-ACTB-mStayGold-0min.tif]

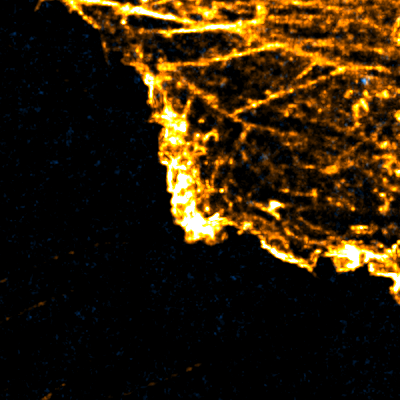

Supplement: Supplementary file 11 — Source data Fig. 5 [file 44318_2024_337_MOESM11_ESM.zip › 05_Figure_05/5C/SIM Time Course/SIM-ACTB-mStayGold-10min.tif]

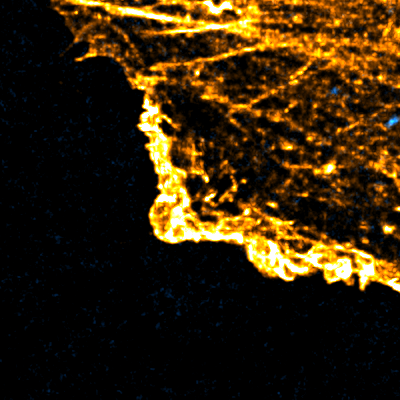

Supplement: Supplementary file 11 — Source data Fig. 5 [file 44318_2024_337_MOESM11_ESM.zip › 05_Figure_05/5C/SIM Time Course/SIM-ACTB-mStayGold-15min.tif]

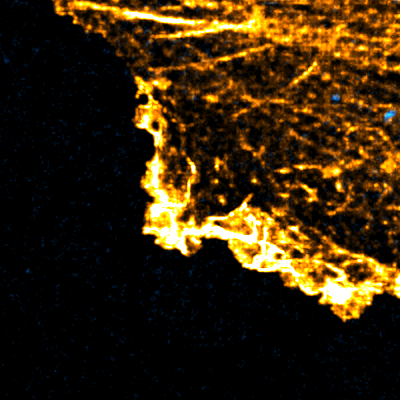

Supplement: Supplementary file 11 — Source data Fig. 5 [file 44318_2024_337_MOESM11_ESM.zip › 05_Figure_05/5C/SIM Time Course/SIM-ACTB-mStayGold-20min.tif]

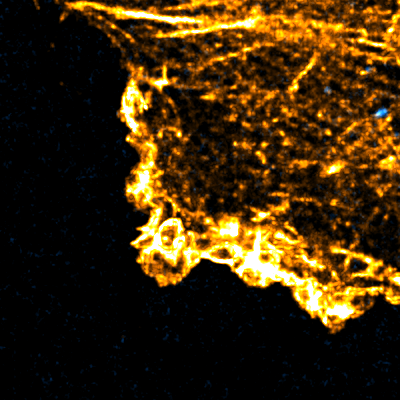

Supplement: Supplementary file 11 — Source data Fig. 5 [file 44318_2024_337_MOESM11_ESM.zip › 05_Figure_05/5C/SIM Time Course/SIM-ACTB-mStayGold-25min.tif]

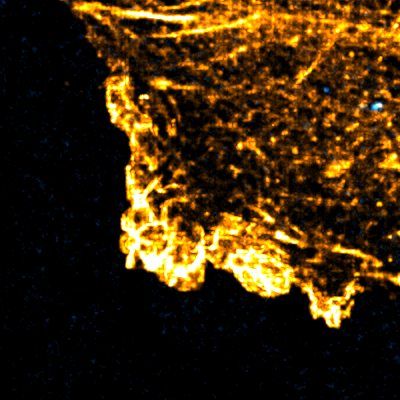

Supplement: Supplementary file 11 — Source data Fig. 5 [file 44318_2024_337_MOESM11_ESM.zip › 05_Figure_05/5C/SIM Time Course/SIM-ACTB-mStayGold-30min.tif]

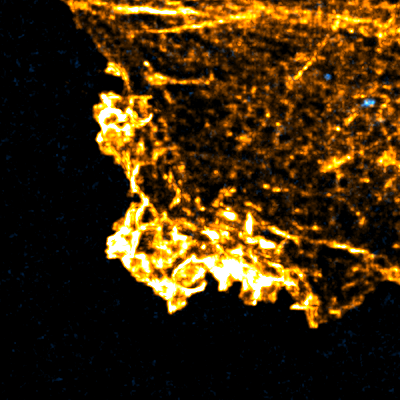

Supplement: Supplementary file 11 — Source data Fig. 5 [file 44318_2024_337_MOESM11_ESM.zip › 05_Figure_05/5C/SIM Time Course/SIM-ACTB-mStayGold-35min.tif]

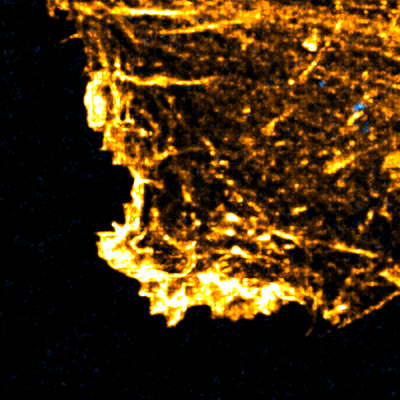

Supplement: Supplementary file 11 — Source data Fig. 5 [file 44318_2024_337_MOESM11_ESM.zip › 05_Figure_05/5C/SIM Time Course/SIM-ACTB-mStayGold-40min.tif]

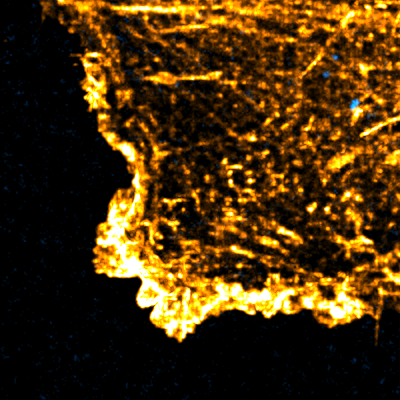

Supplement: Supplementary file 11 — Source data Fig. 5 [file 44318_2024_337_MOESM11_ESM.zip › 05_Figure_05/5C/SIM Time Course/SIM-ACTB-mStayGold-45min.tif]

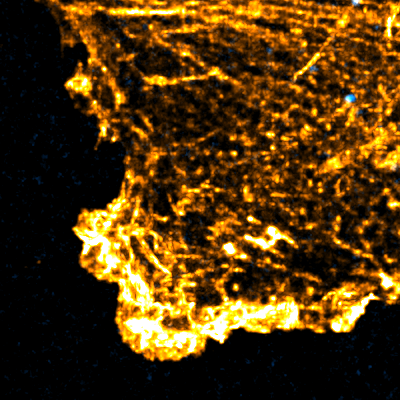

Supplement: Supplementary file 11 — Source data Fig. 5 [file 44318_2024_337_MOESM11_ESM.zip › 05_Figure_05/5C/SIM Time Course/SIM-ACTB-mStayGold-50min.tif]

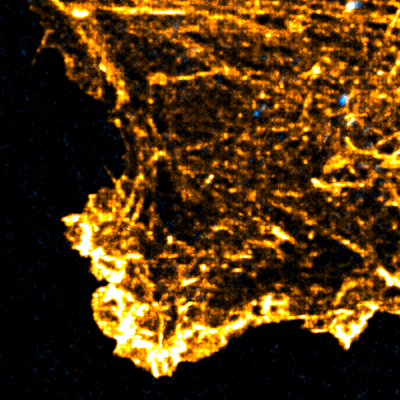

Supplement: Supplementary file 11 — Source data Fig. 5 [file 44318_2024_337_MOESM11_ESM.zip › 05_Figure_05/5C/SIM Time Course/SIM-ACTB-mStayGold-55min.tif]

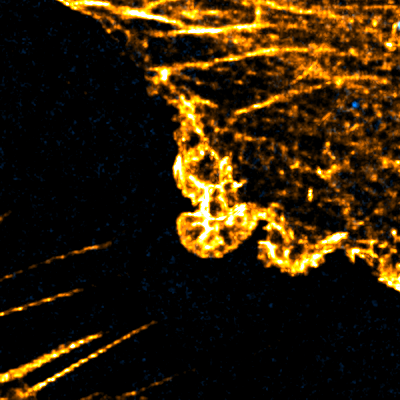

Supplement: Supplementary file 11 — Source data Fig. 5 [file 44318_2024_337_MOESM11_ESM.zip › 05_Figure_05/5C/SIM Time Course/SIM-ACTB-mStayGold-5min.tif]

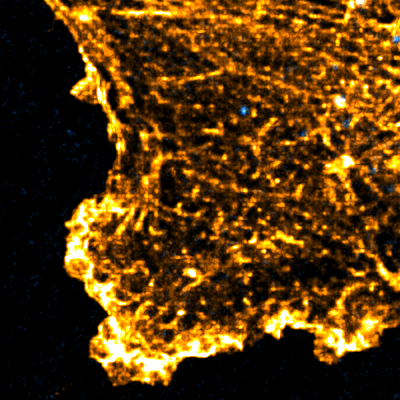

Supplement: Supplementary file 11 — Source data Fig. 5 [file 44318_2024_337_MOESM11_ESM.zip › 05_Figure_05/5C/SIM Time Course/SIM-ACTB-mStayGold-60min.tif]

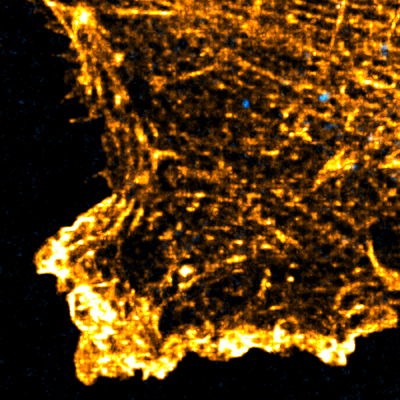

Supplement: Supplementary file 11 — Source data Fig. 5 [file 44318_2024_337_MOESM11_ESM.zip › 05_Figure_05/5C/SIM Time Course/SIM-ACTB-mStayGold-65min.tif]

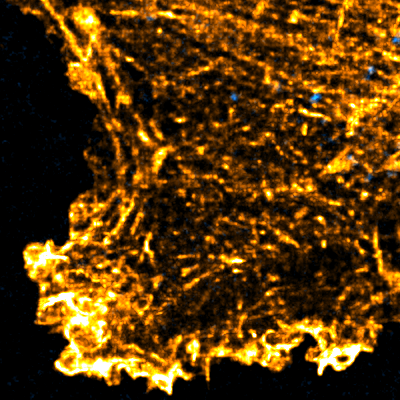

Supplement: Supplementary file 11 — Source data Fig. 5 [file 44318_2024_337_MOESM11_ESM.zip › 05_Figure_05/5C/SIM Time Course/SIM-ACTB-mStayGold-70min.tif]

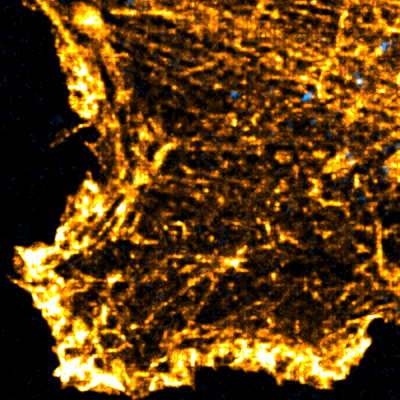

Supplement: Supplementary file 11 — Source data Fig. 5 [file 44318_2024_337_MOESM11_ESM.zip › 05_Figure_05/5C/SIM Time Course/SIM-ACTB-mStayGold-75min.tif]

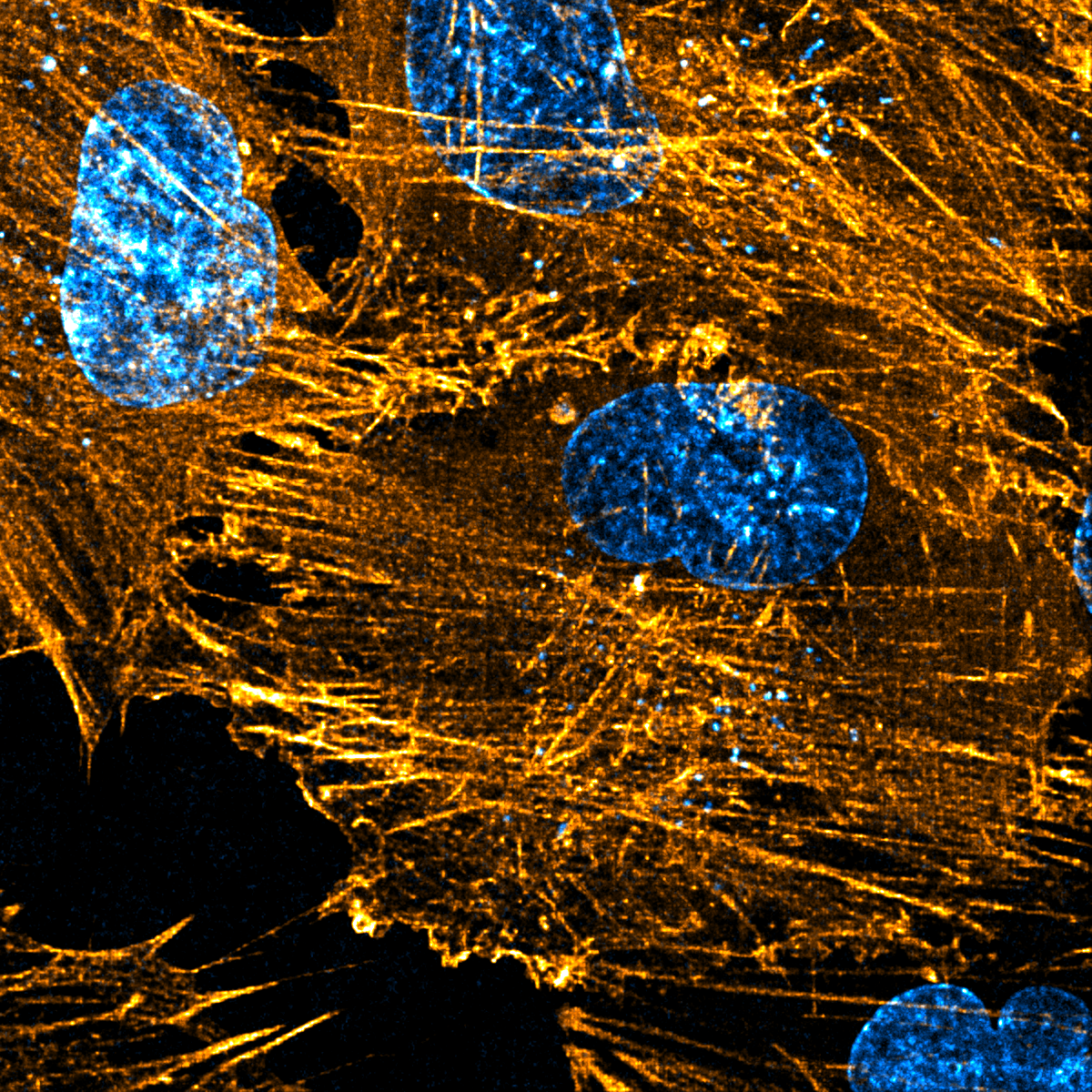

Supplement: Supplementary file 11 — Source data Fig. 5 [file 44318_2024_337_MOESM11_ESM.zip › 05_Figure_05/5C/SIM-ACTB-mStayGold.tif]

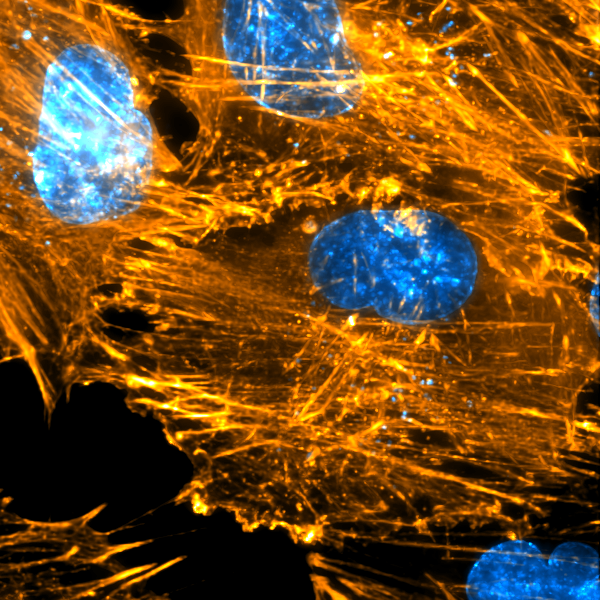

Supplement: Supplementary file 11 — Source data Fig. 5 [file 44318_2024_337_MOESM11_ESM.zip › 05_Figure_05/5C/Widefield-ACTB-mStayGold.tif]

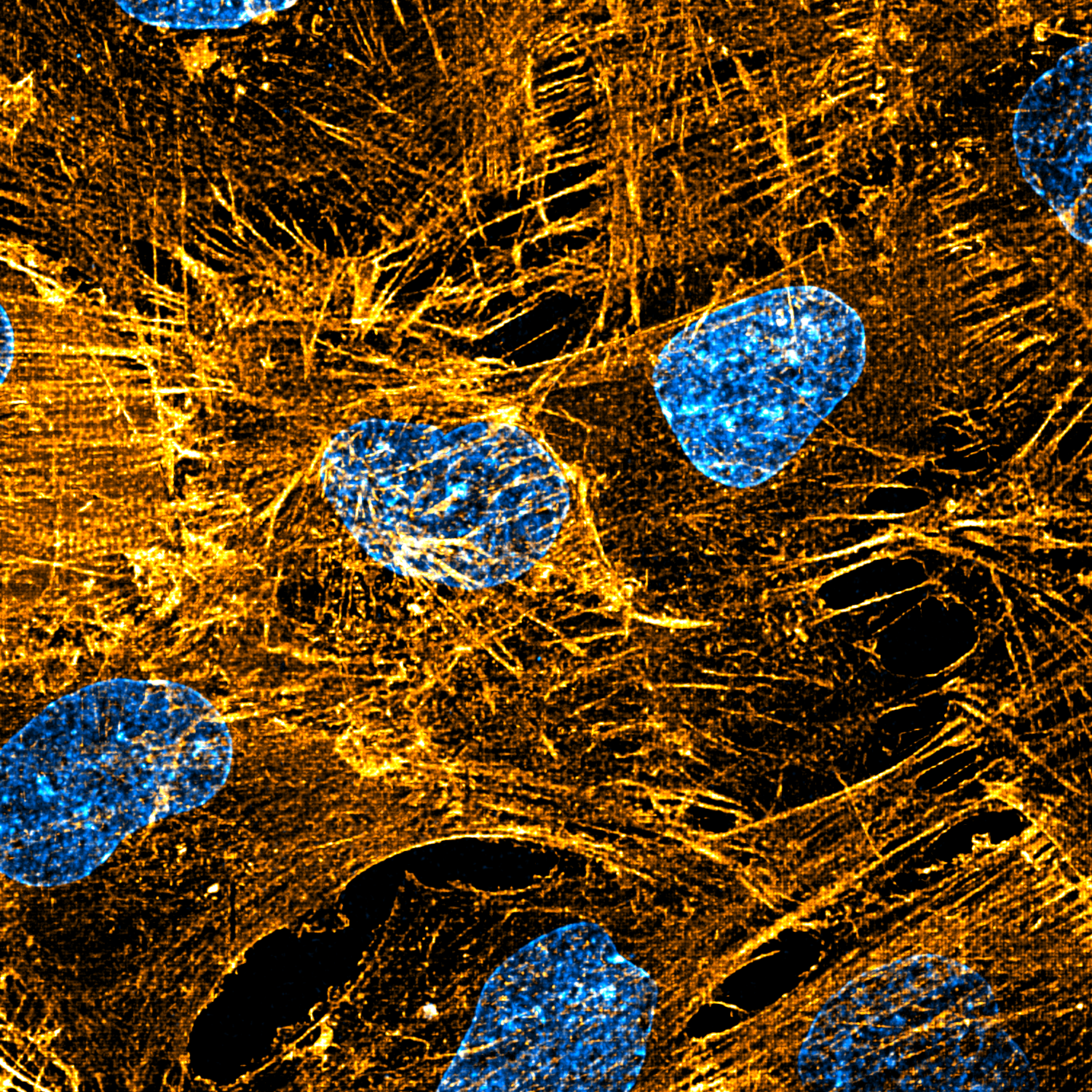

Supplement: Supplementary file 11 — Source data Fig. 5 [file 44318_2024_337_MOESM11_ESM.zip › 05_Figure_05/5D/ACTB.tif]

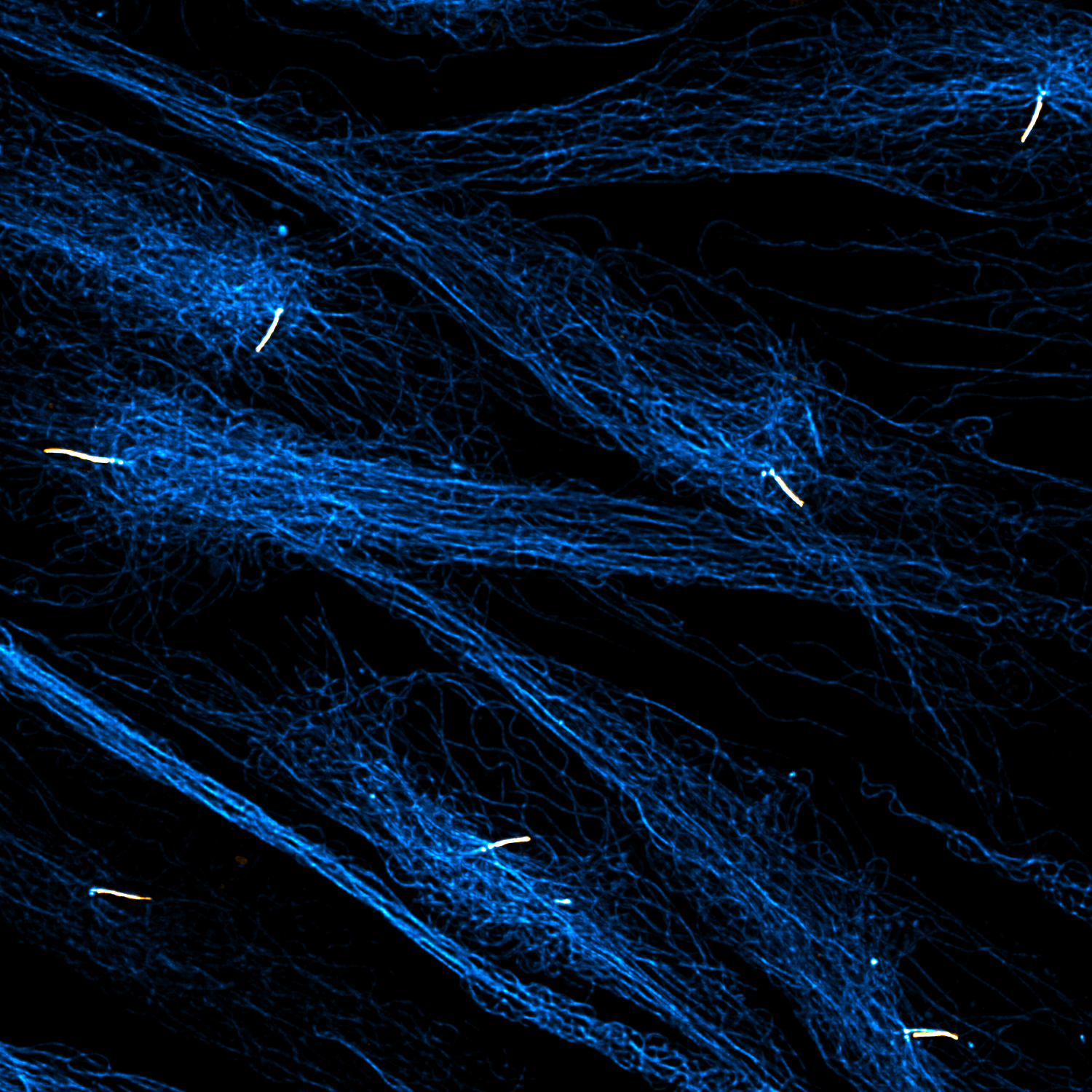

Supplement: Supplementary file 11 — Source data Fig. 5 [file 44318_2024_337_MOESM11_ESM.zip › 05_Figure_05/5D/ARL13B.tif]

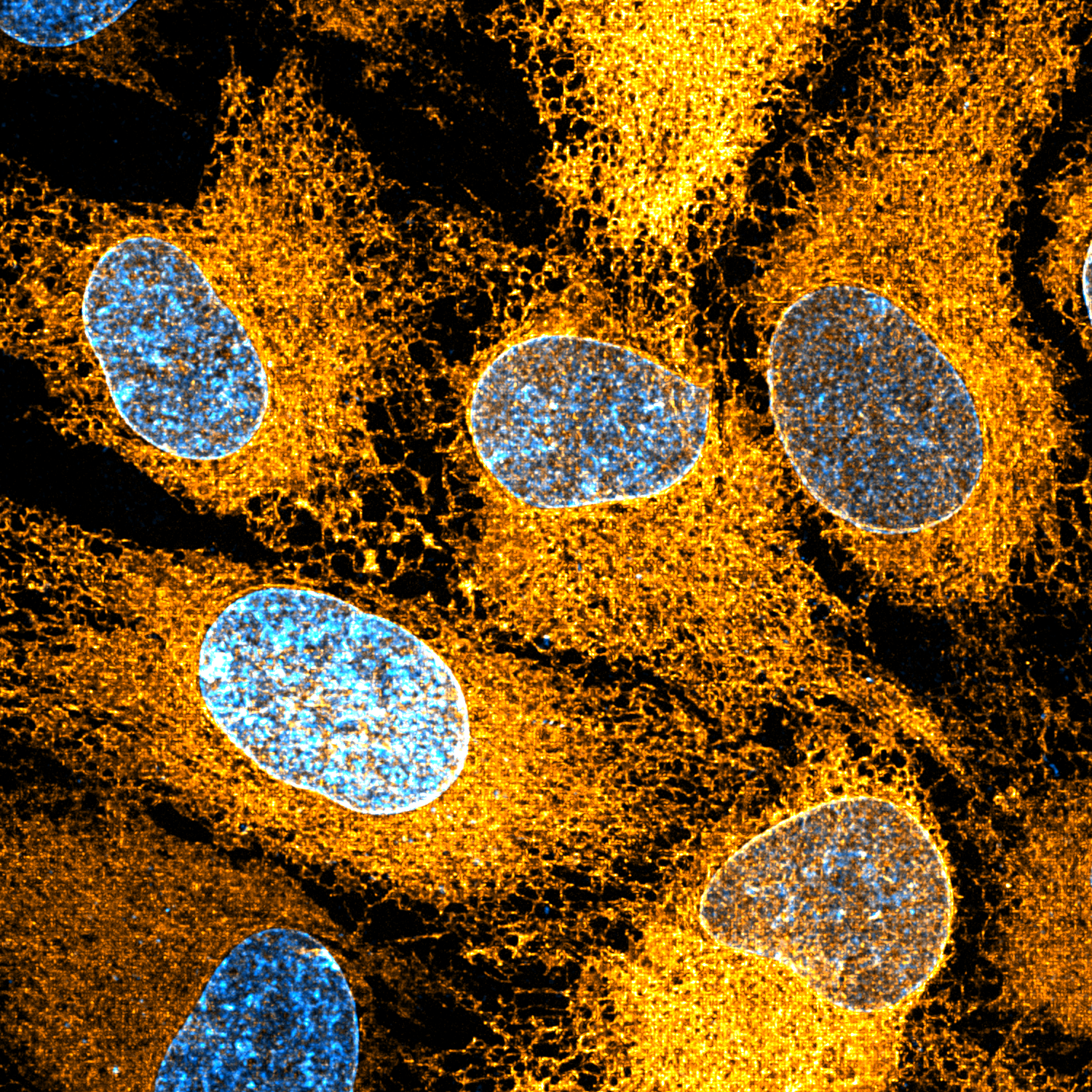

Supplement: Supplementary file 11 — Source data Fig. 5 [file 44318_2024_337_MOESM11_ESM.zip › 05_Figure_05/5D/CANX.tif]

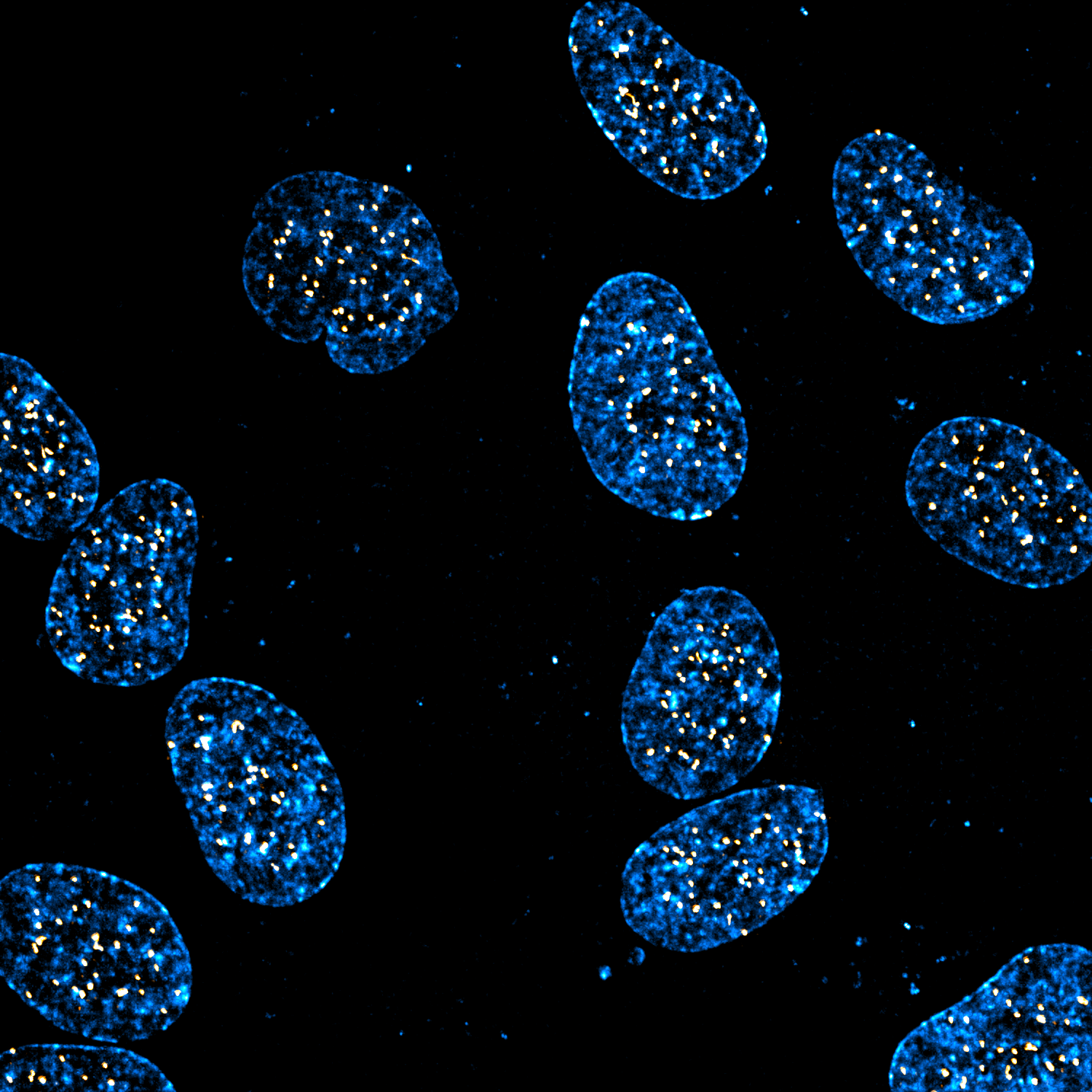

Supplement: Supplementary file 11 — Source data Fig. 5 [file 44318_2024_337_MOESM11_ESM.zip › 05_Figure_05/5D/CENPA.tif]

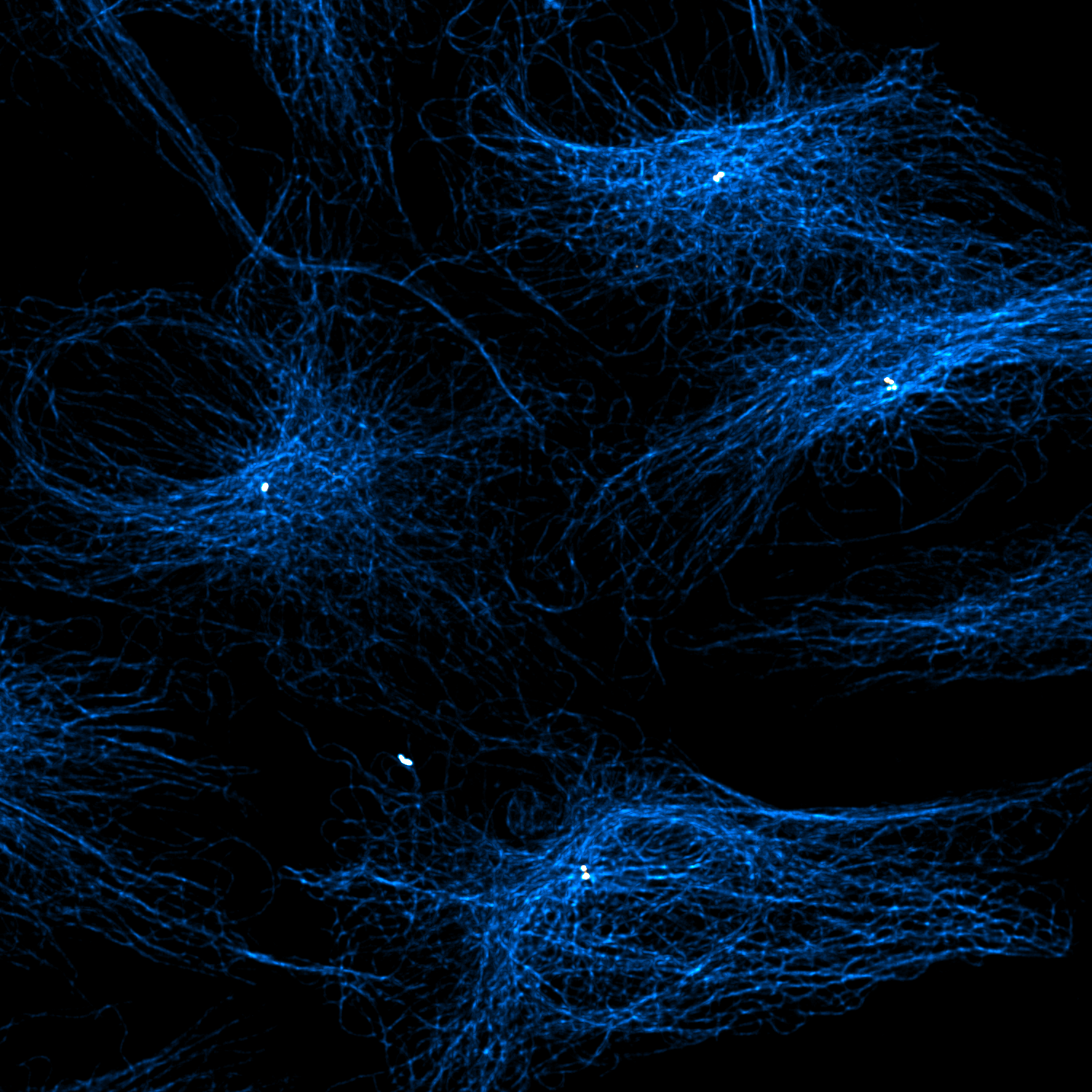

Supplement: Supplementary file 11 — Source data Fig. 5 [file 44318_2024_337_MOESM11_ESM.zip › 05_Figure_05/5D/CEP135.tif]

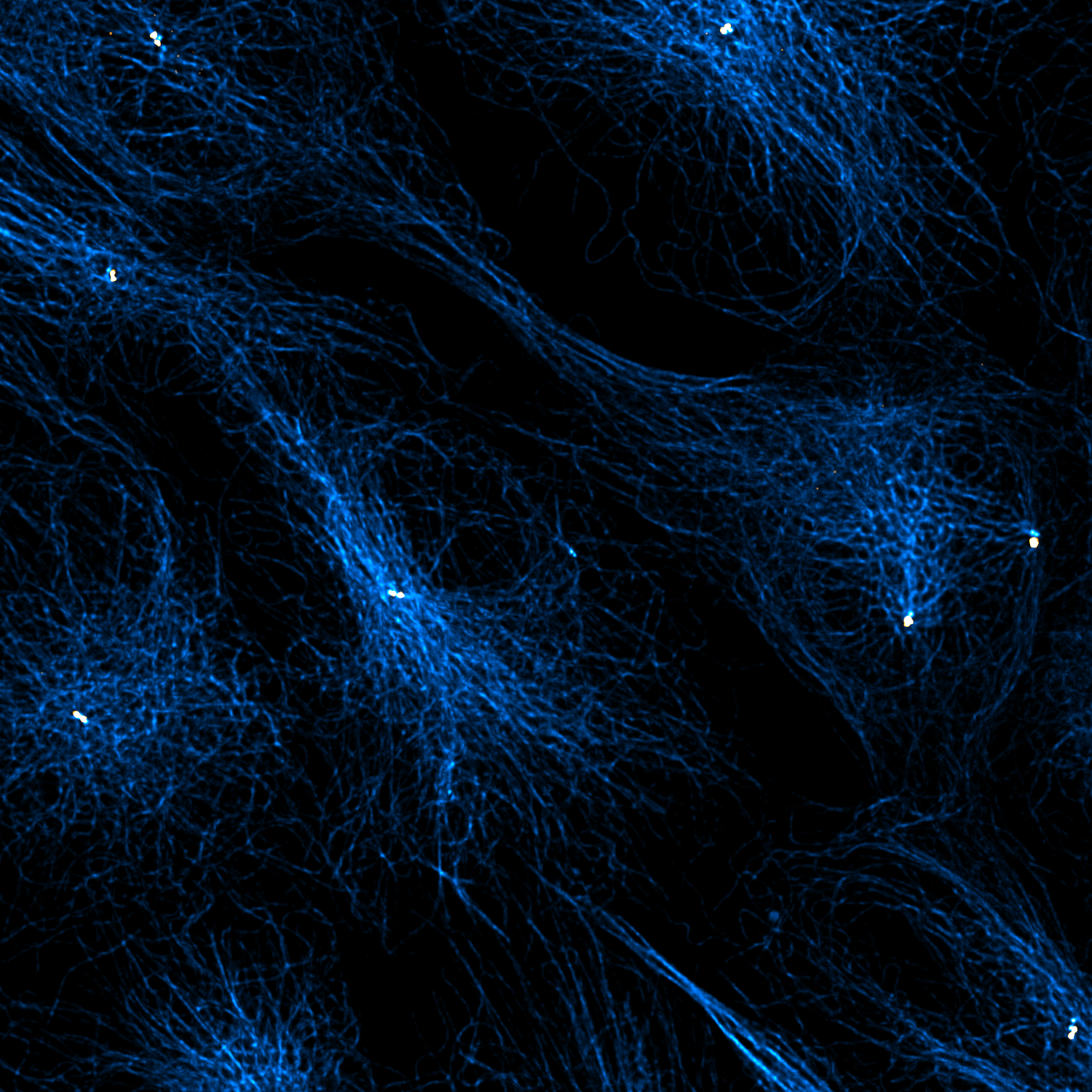

Supplement: Supplementary file 11 — Source data Fig. 5 [file 44318_2024_337_MOESM11_ESM.zip › 05_Figure_05/5D/CEP192.tif]

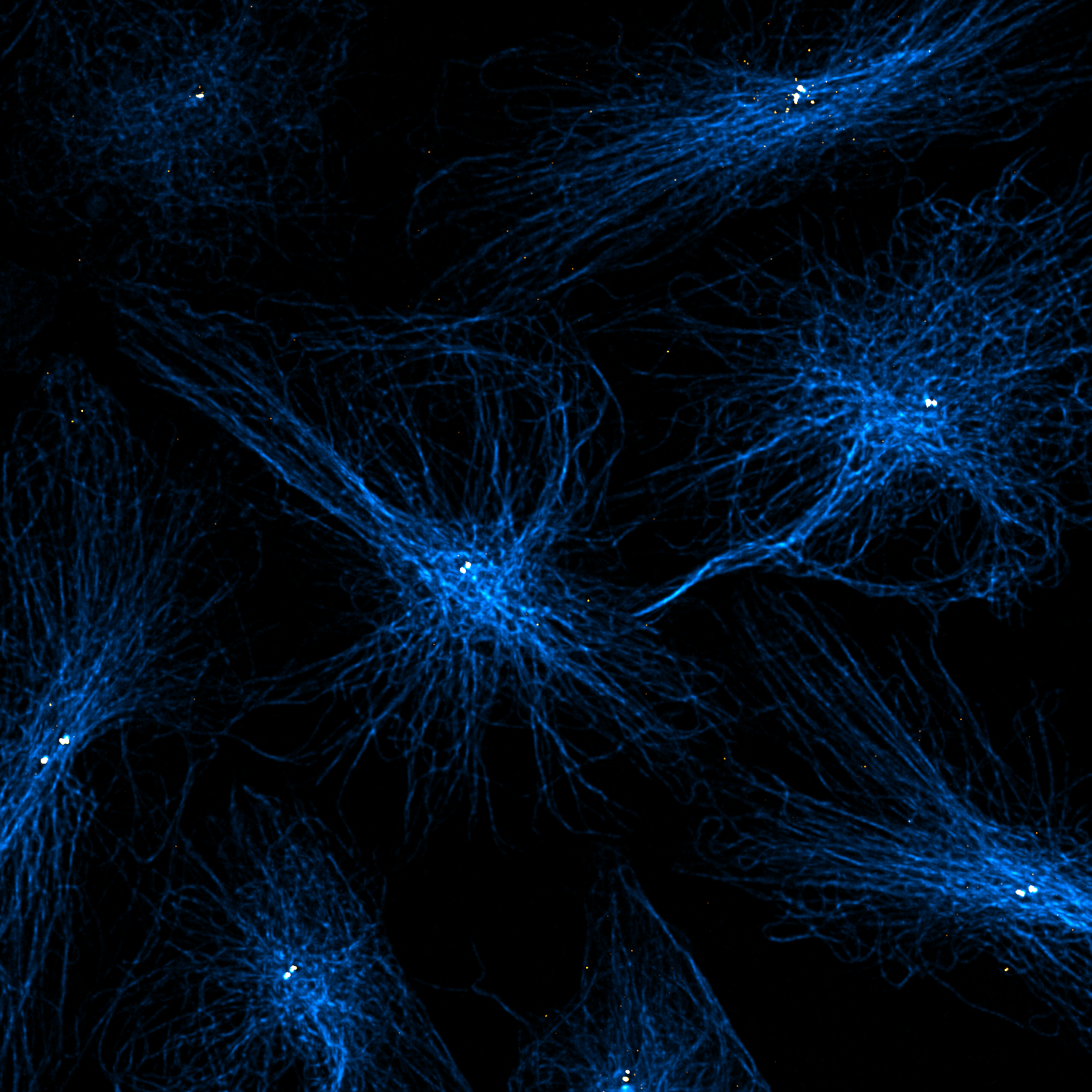

Supplement: Supplementary file 11 — Source data Fig. 5 [file 44318_2024_337_MOESM11_ESM.zip › 05_Figure_05/5D/CETN2.tif]

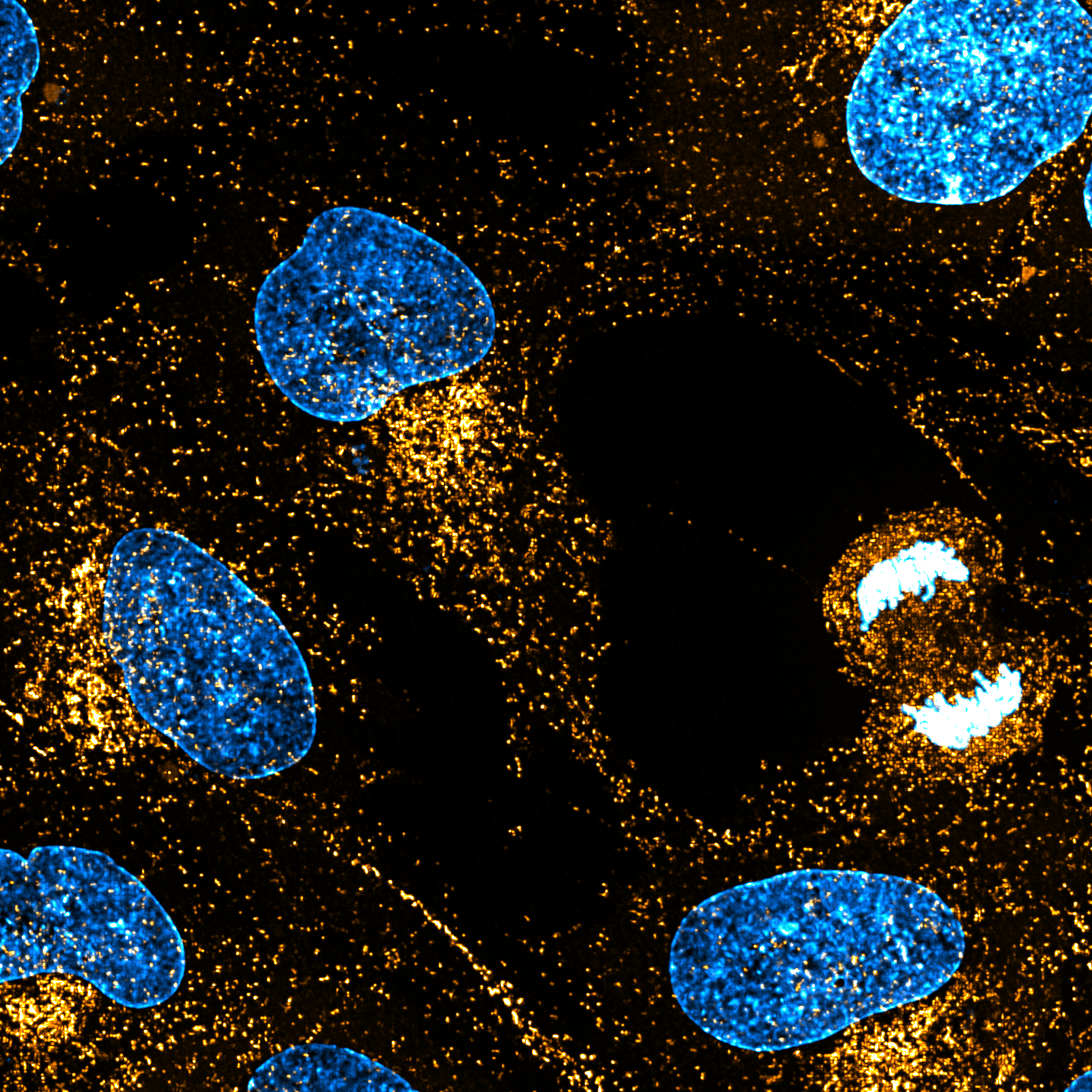

Supplement: Supplementary file 11 — Source data Fig. 5 [file 44318_2024_337_MOESM11_ESM.zip › 05_Figure_05/5D/CLTC.tif]

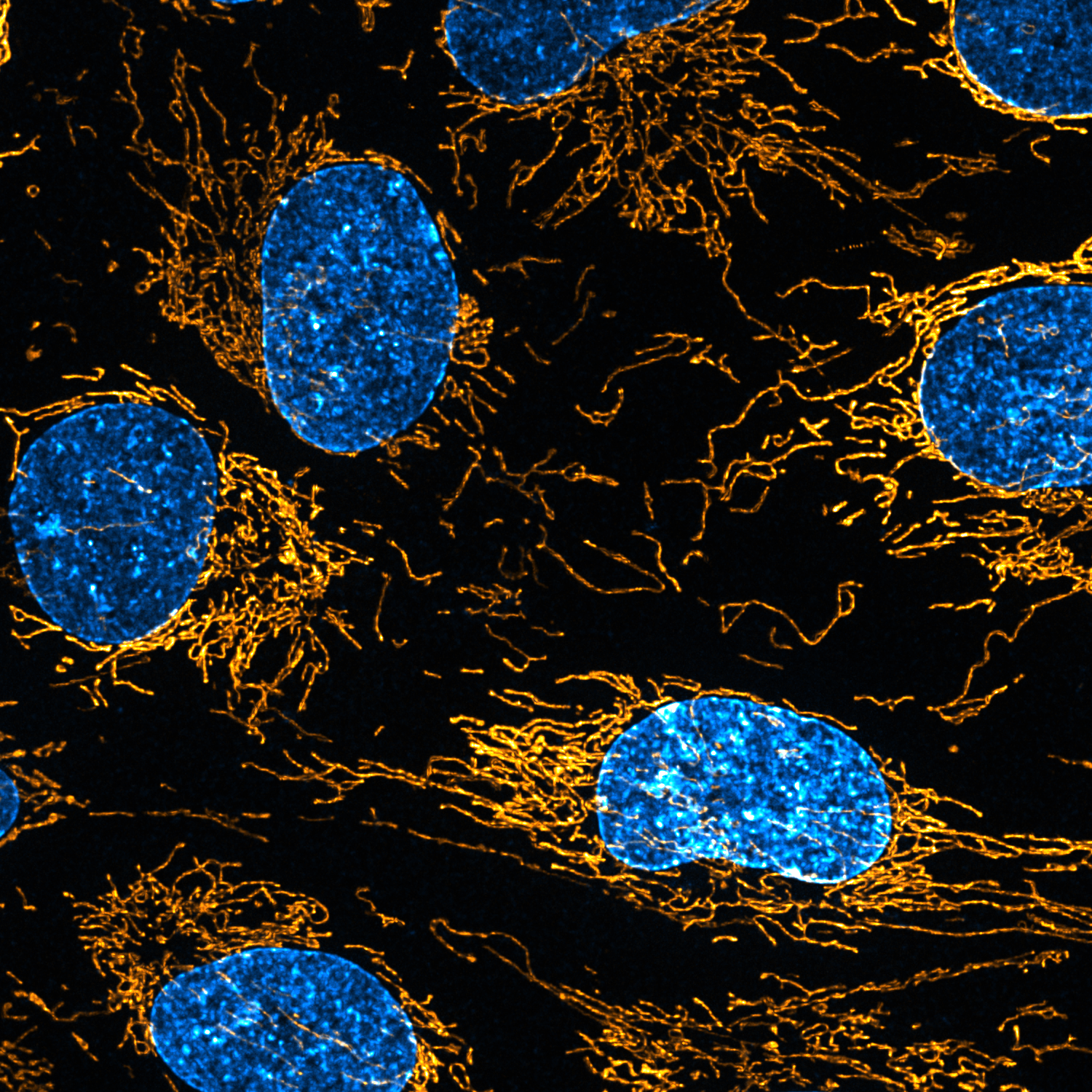

Supplement: Supplementary file 11 — Source data Fig. 5 [file 44318_2024_337_MOESM11_ESM.zip › 05_Figure_05/5D/COX8A.tif]

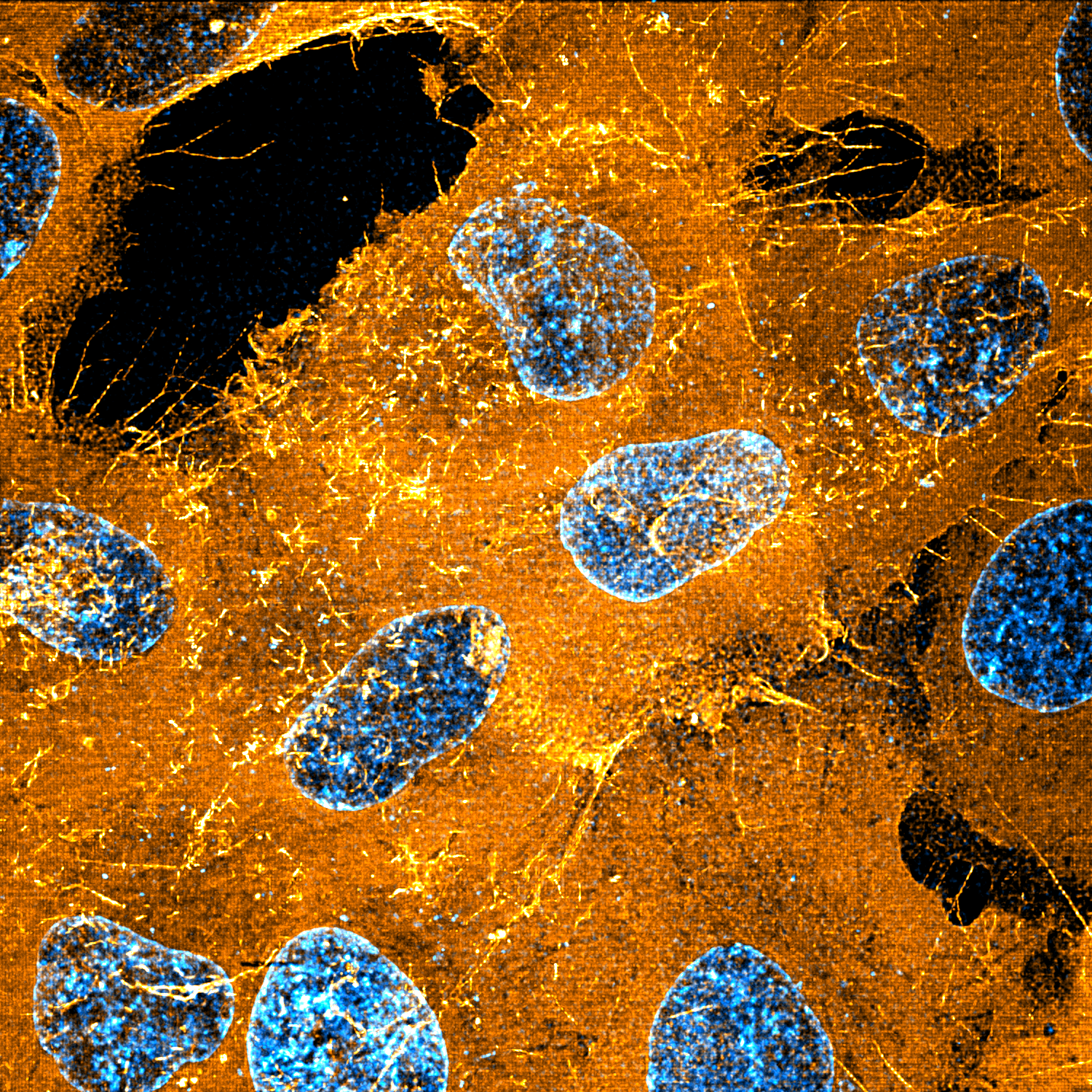

Supplement: Supplementary file 11 — Source data Fig. 5 [file 44318_2024_337_MOESM11_ESM.zip › 05_Figure_05/5D/EZR.tif]

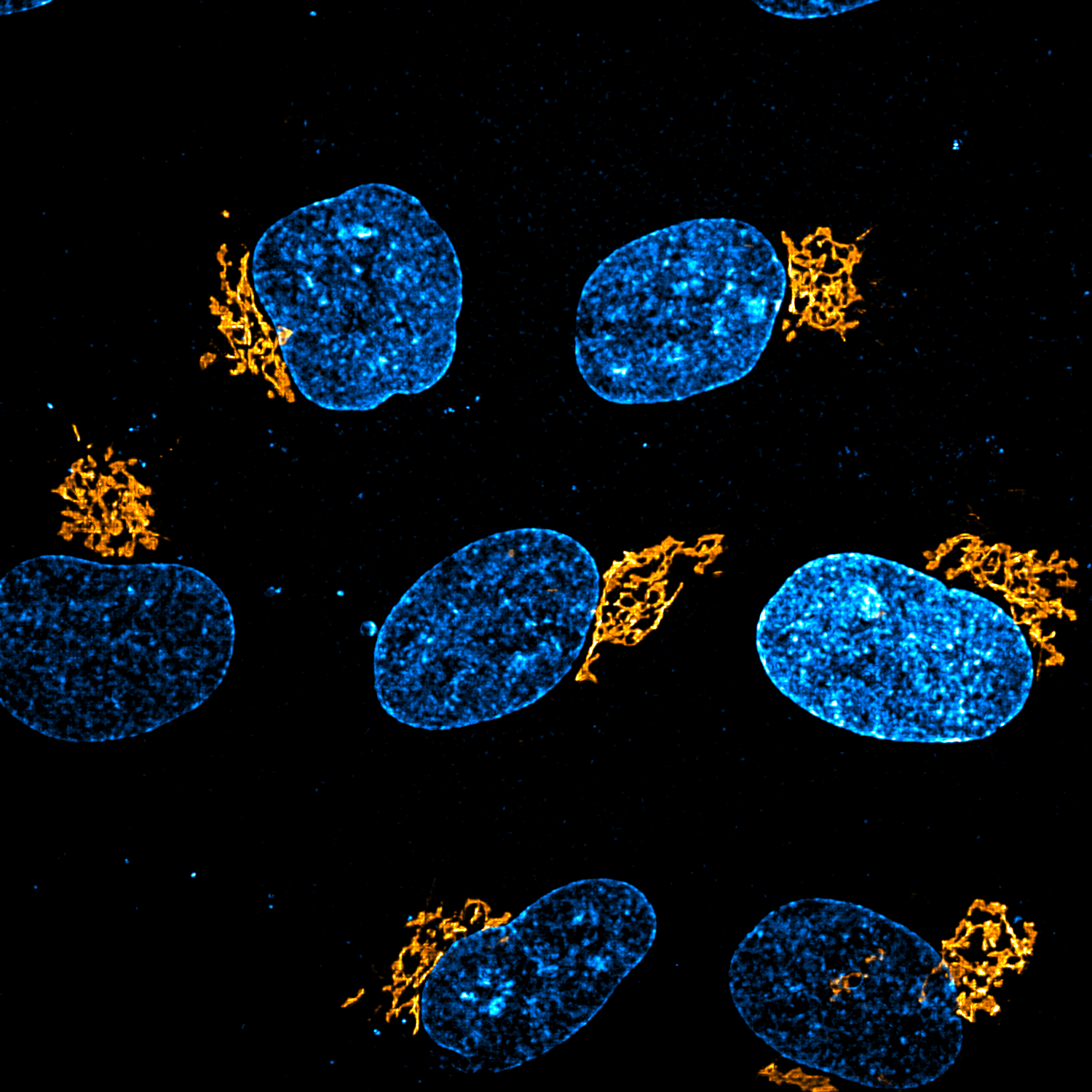

Supplement: Supplementary file 11 — Source data Fig. 5 [file 44318_2024_337_MOESM11_ESM.zip › 05_Figure_05/5D/GOLGA2.tif]

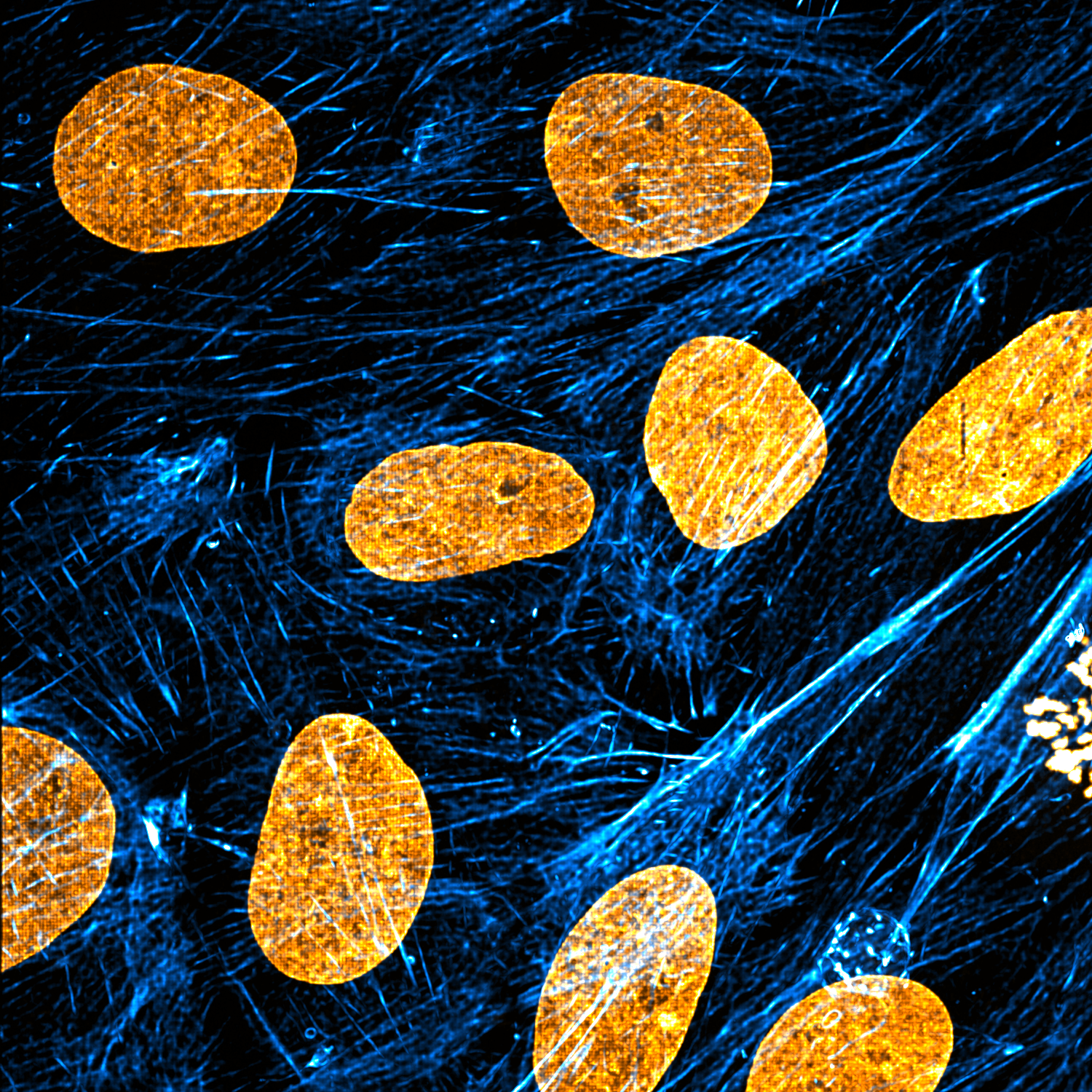

Supplement: Supplementary file 11 — Source data Fig. 5 [file 44318_2024_337_MOESM11_ESM.zip › 05_Figure_05/5D/H2BC11.tif]

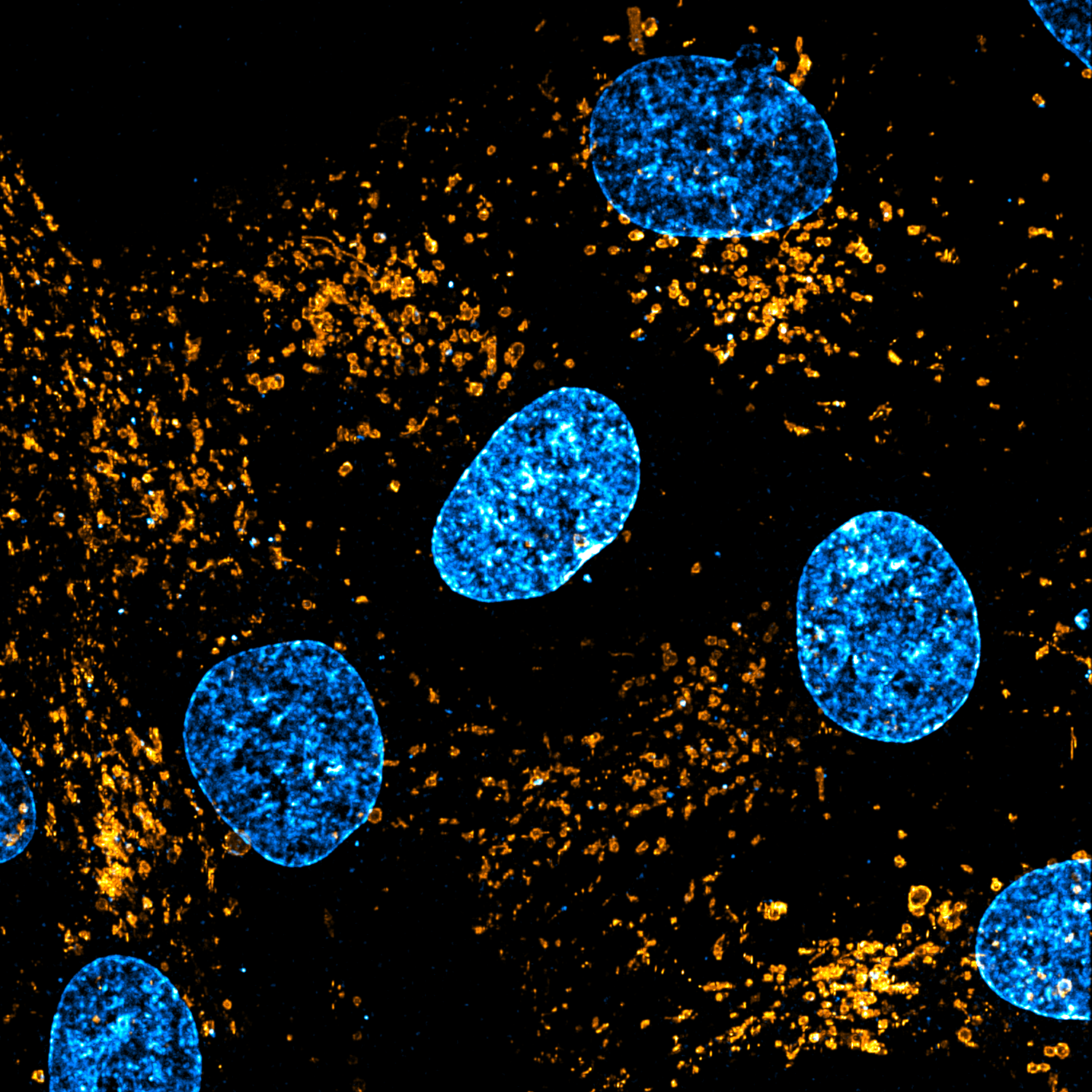

Supplement: Supplementary file 11 — Source data Fig. 5 [file 44318_2024_337_MOESM11_ESM.zip › 05_Figure_05/5D/LAMP1.tif]

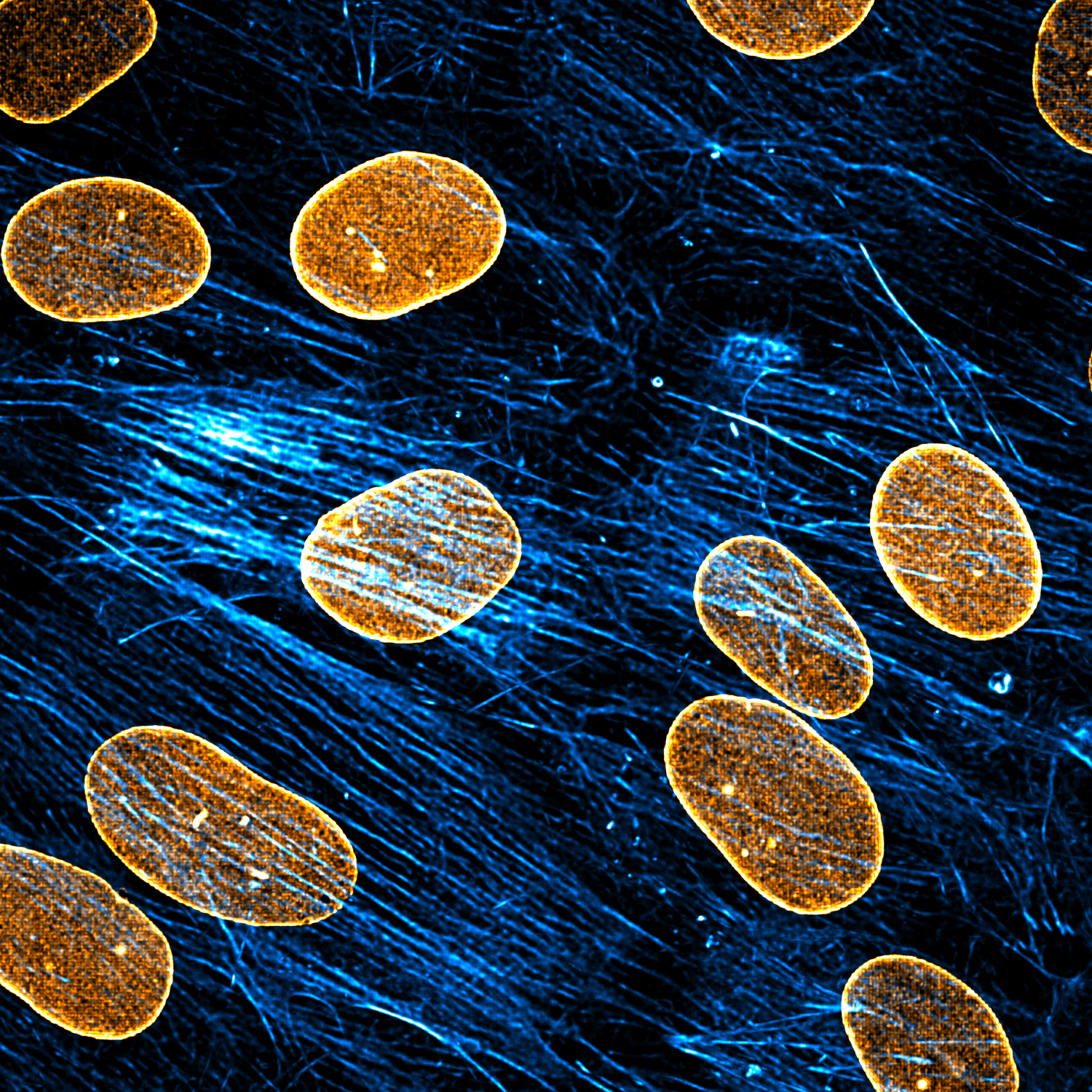

Supplement: Supplementary file 11 — Source data Fig. 5 [file 44318_2024_337_MOESM11_ESM.zip › 05_Figure_05/5D/LMNB1.tif]

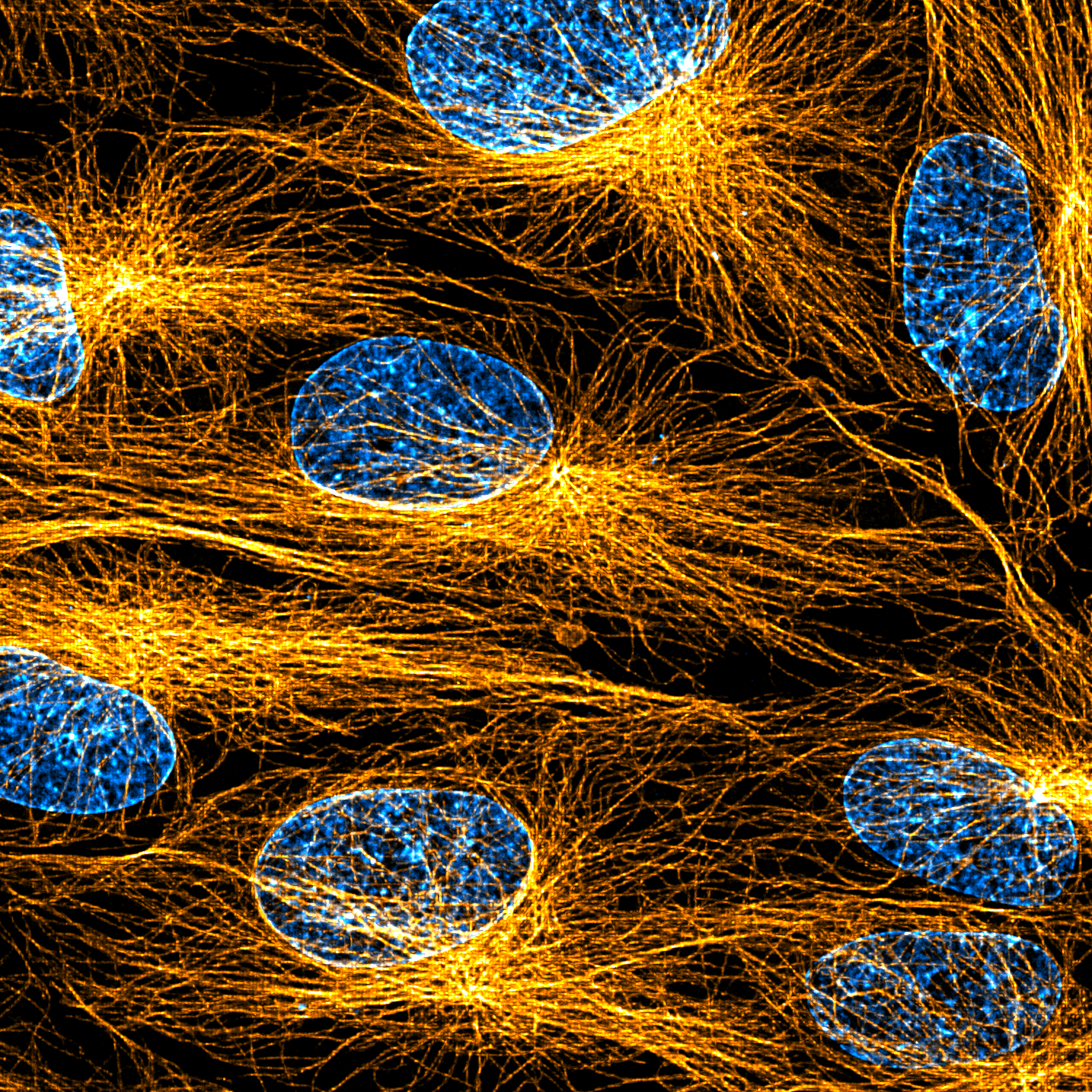

Supplement: Supplementary file 11 — Source data Fig. 5 [file 44318_2024_337_MOESM11_ESM.zip › 05_Figure_05/5D/MAP4.tif]

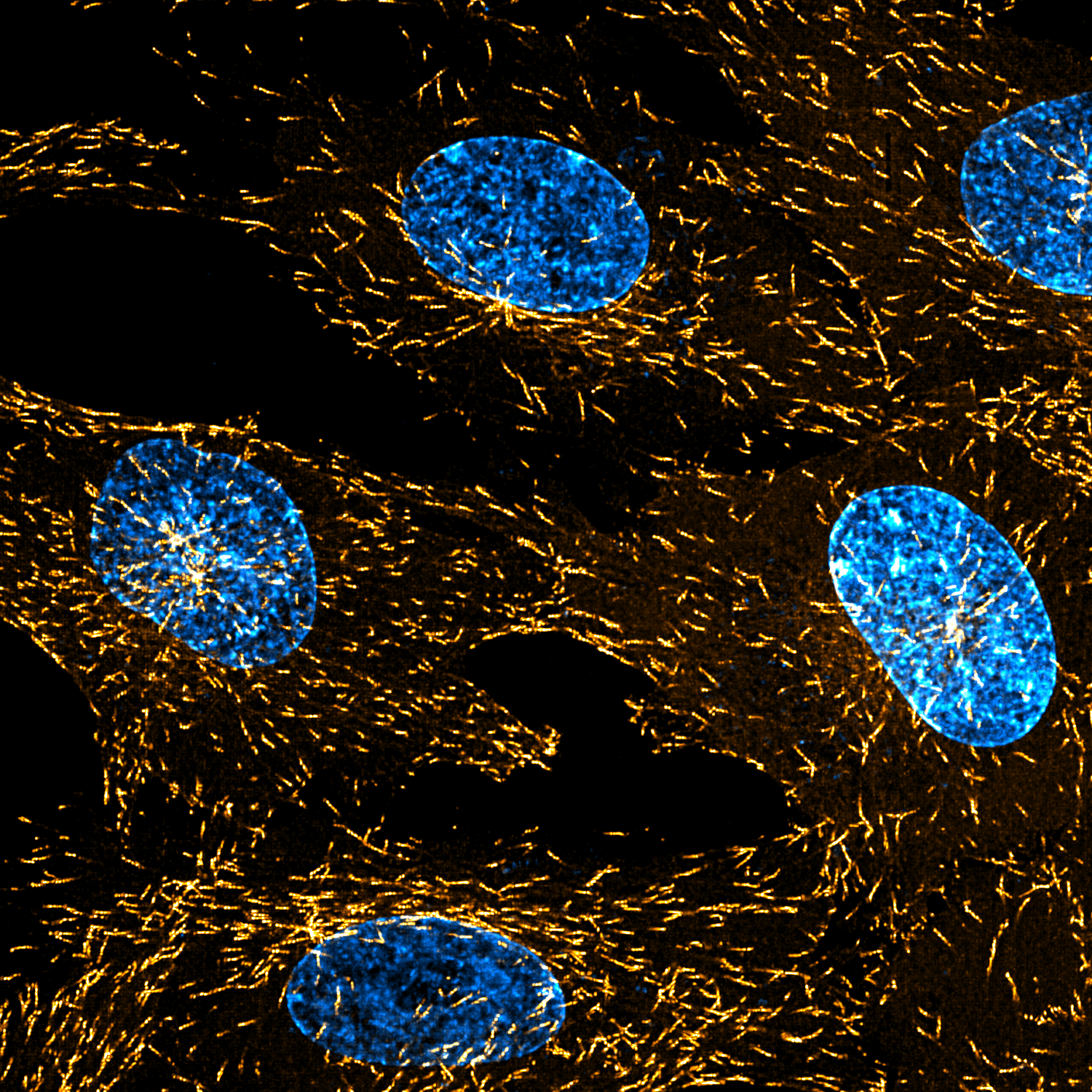

Supplement: Supplementary file 11 — Source data Fig. 5 [file 44318_2024_337_MOESM11_ESM.zip › 05_Figure_05/5D/MAPRE1.tif]

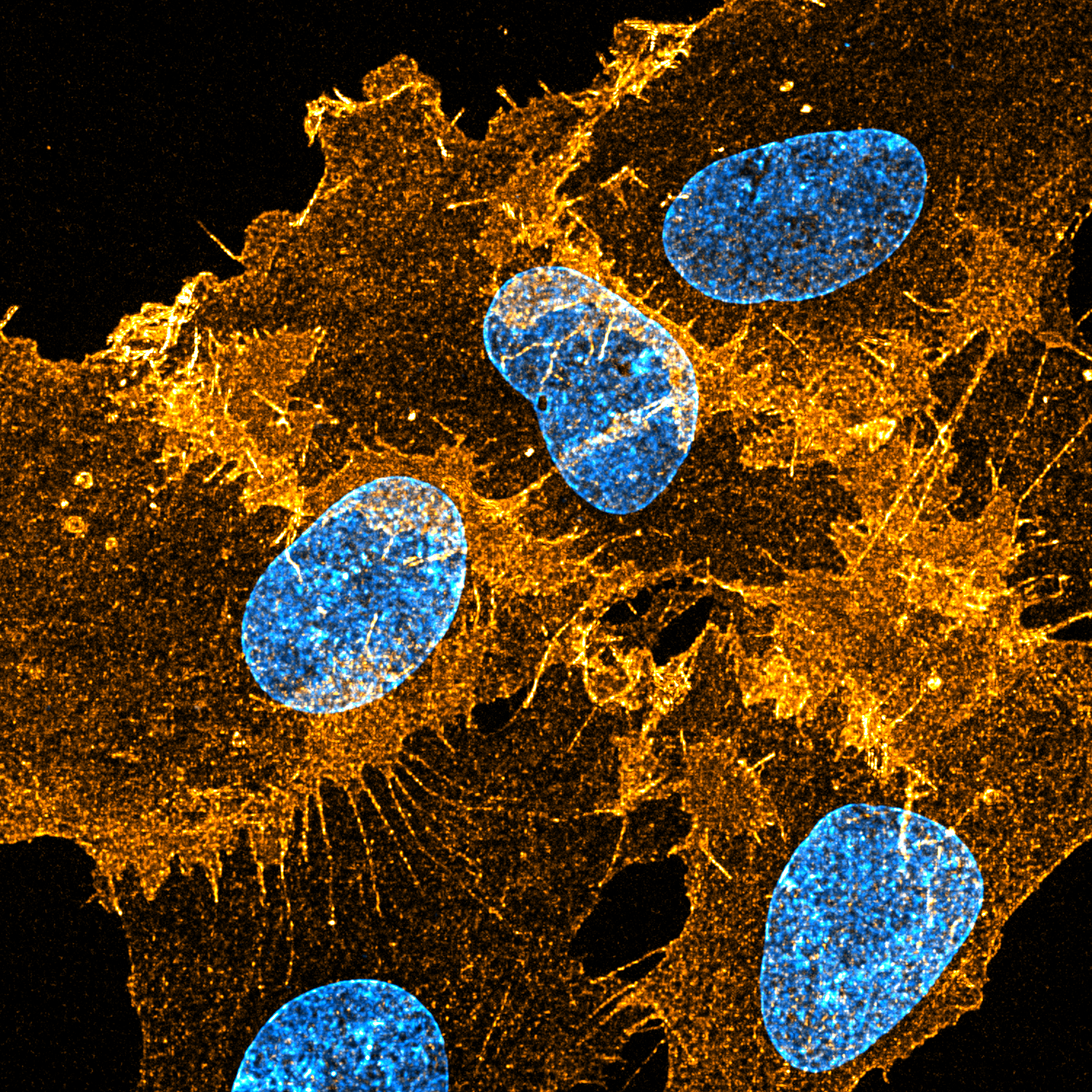

Supplement: Supplementary file 11 — Source data Fig. 5 [file 44318_2024_337_MOESM11_ESM.zip › 05_Figure_05/5D/MYO1C.tif]

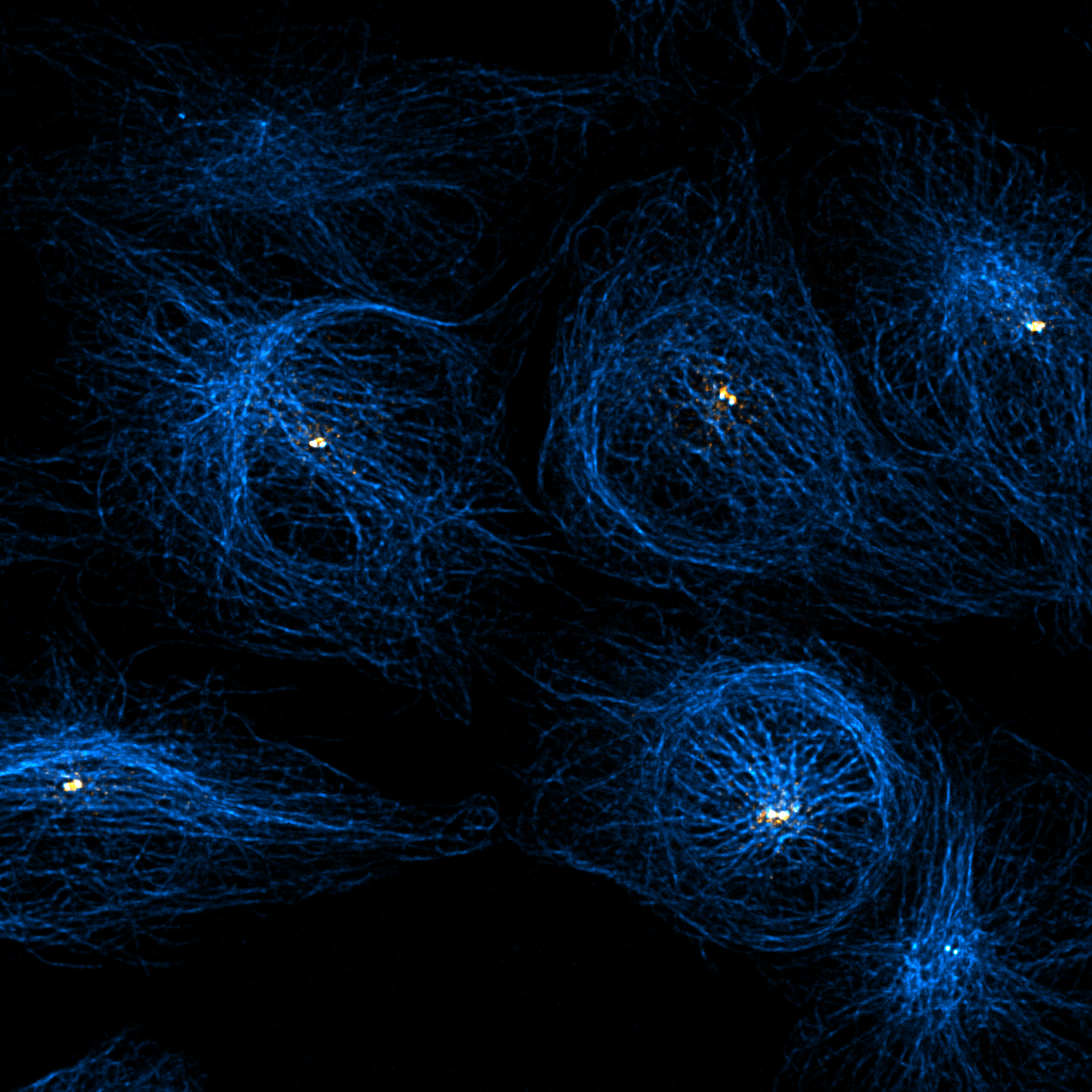

Supplement: Supplementary file 11 — Source data Fig. 5 [file 44318_2024_337_MOESM11_ESM.zip › 05_Figure_05/5D/PCNT.tif]

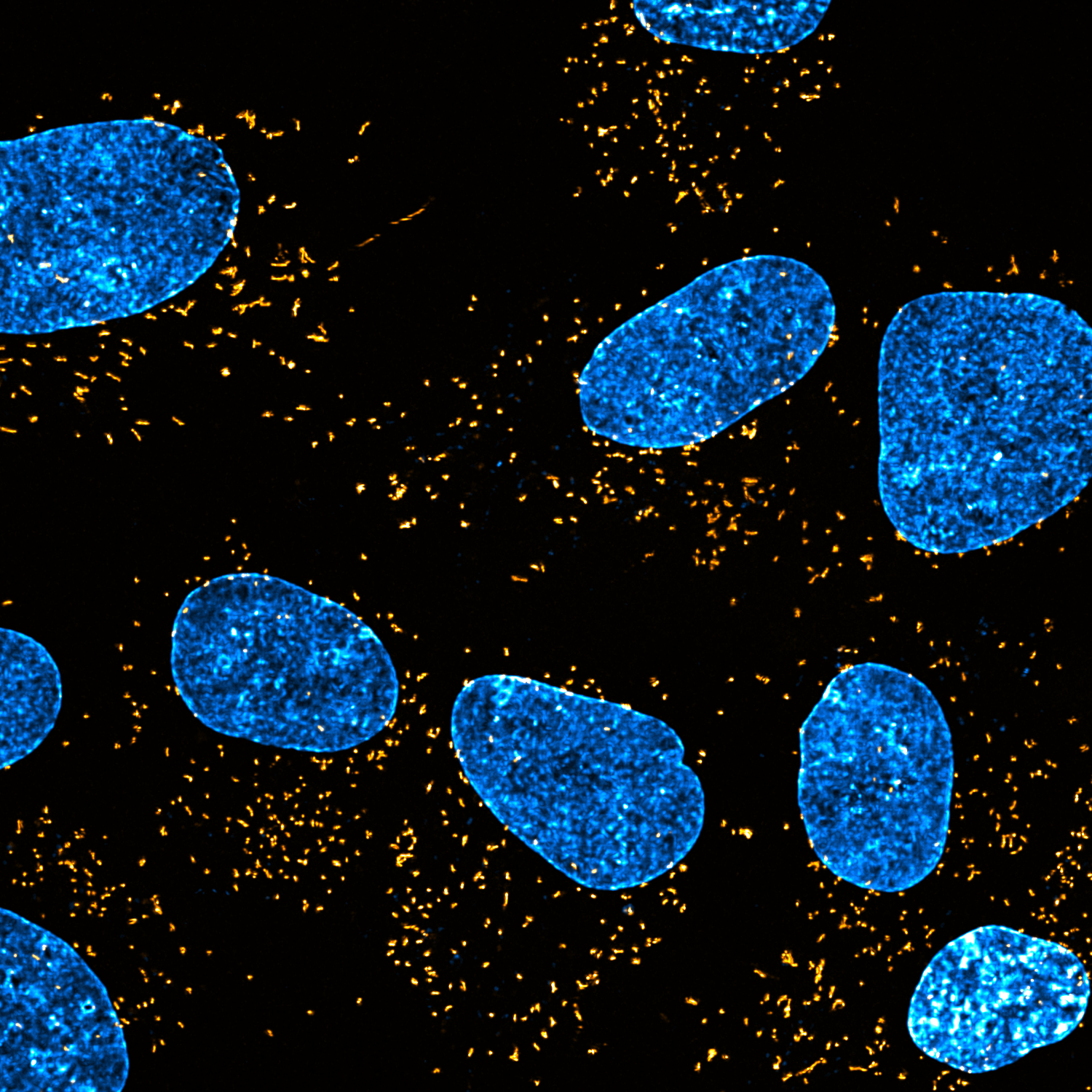

Supplement: Supplementary file 11 — Source data Fig. 5 [file 44318_2024_337_MOESM11_ESM.zip › 05_Figure_05/5D/PEX3.tif]

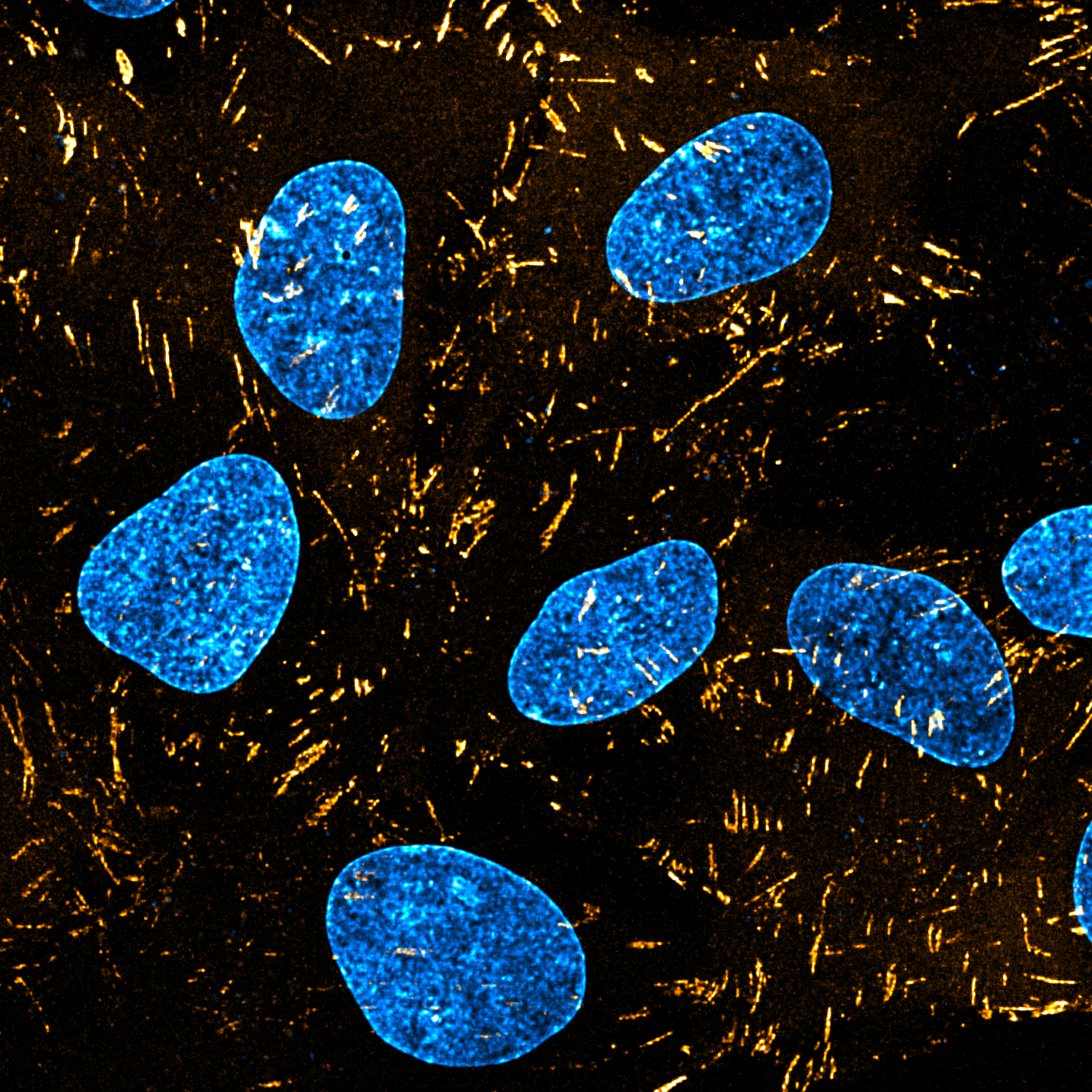

Supplement: Supplementary file 11 — Source data Fig. 5 [file 44318_2024_337_MOESM11_ESM.zip › 05_Figure_05/5D/PXN.tif]

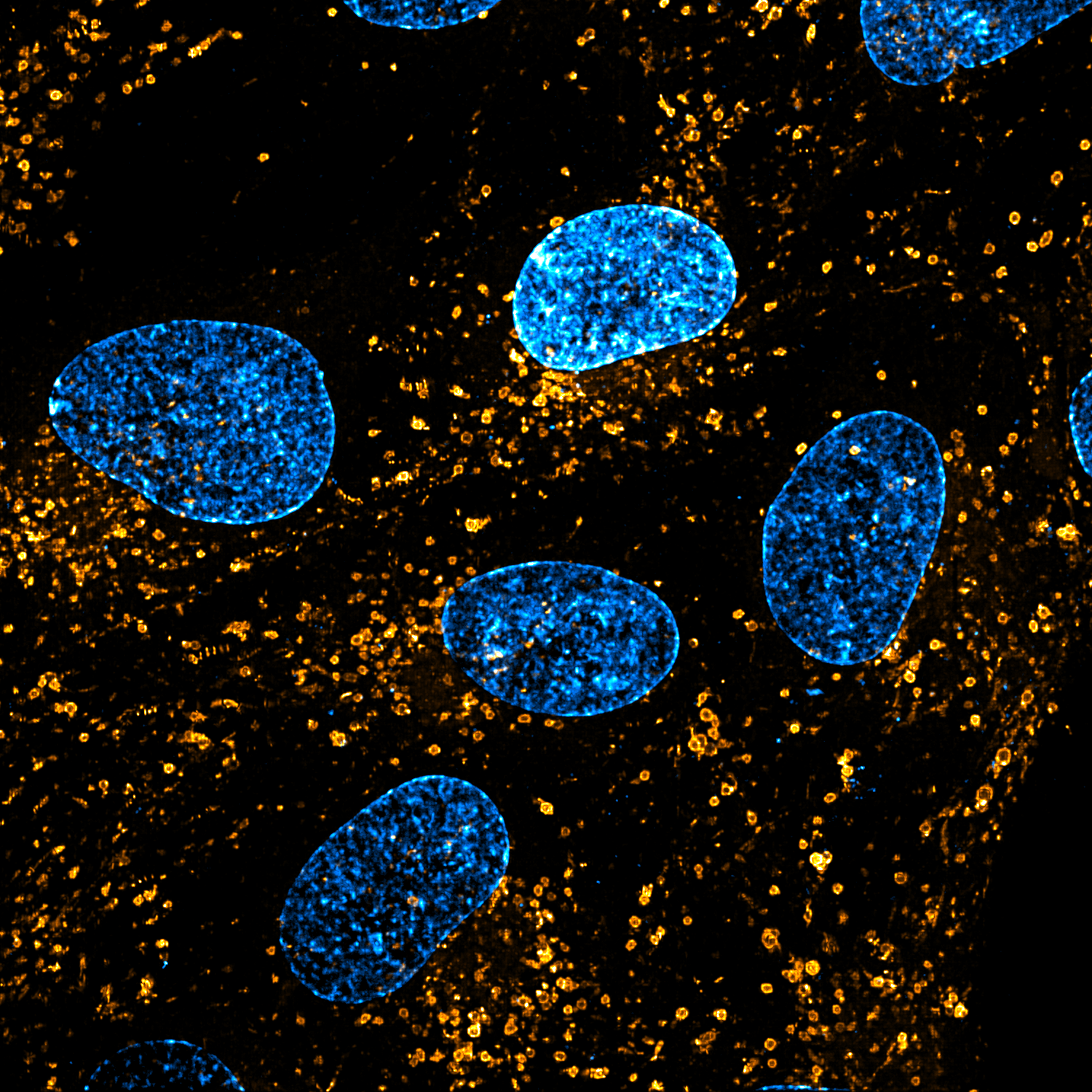

Supplement: Supplementary file 11 — Source data Fig. 5 [file 44318_2024_337_MOESM11_ESM.zip › 05_Figure_05/5D/RAB7A.tif]

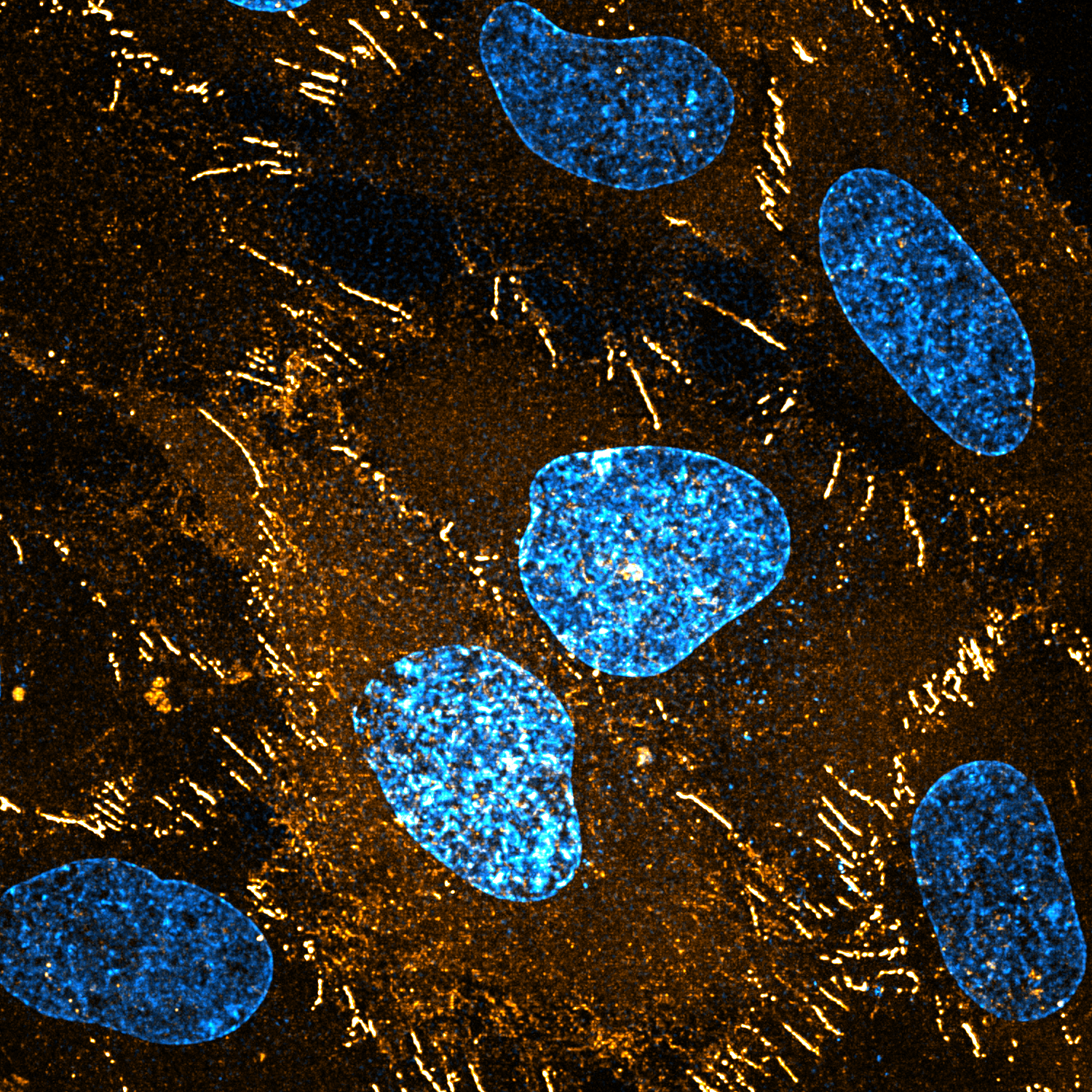

Supplement: Supplementary file 11 — Source data Fig. 5 [file 44318_2024_337_MOESM11_ESM.zip › 05_Figure_05/5D/TJP1.tif]

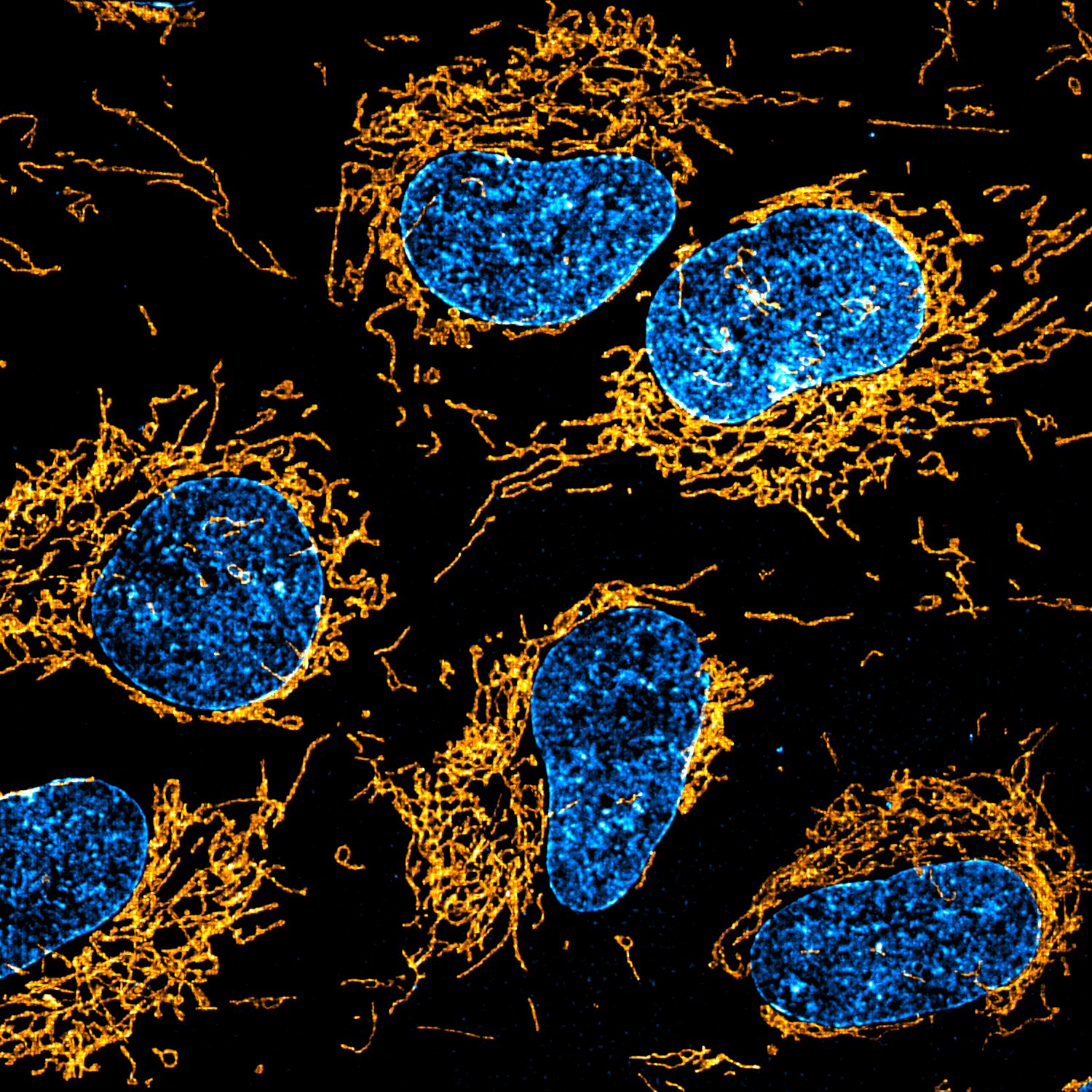

Supplement: Supplementary file 11 — Source data Fig. 5 [file 44318_2024_337_MOESM11_ESM.zip › 05_Figure_05/5D/TOMM20.tif]

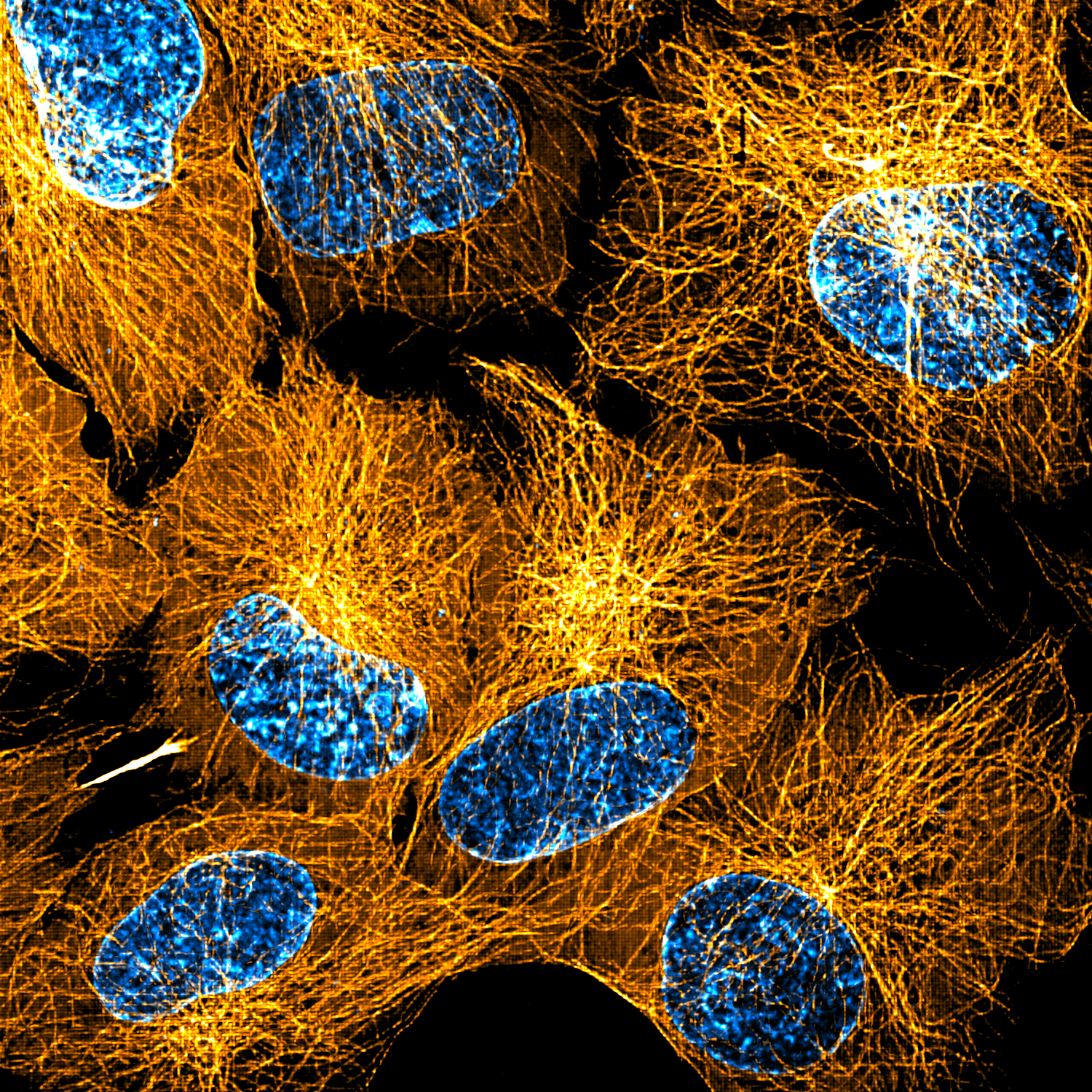

Supplement: Supplementary file 11 — Source data Fig. 5 [file 44318_2024_337_MOESM11_ESM.zip › 05_Figure_05/5D/TUBA1B.tif]

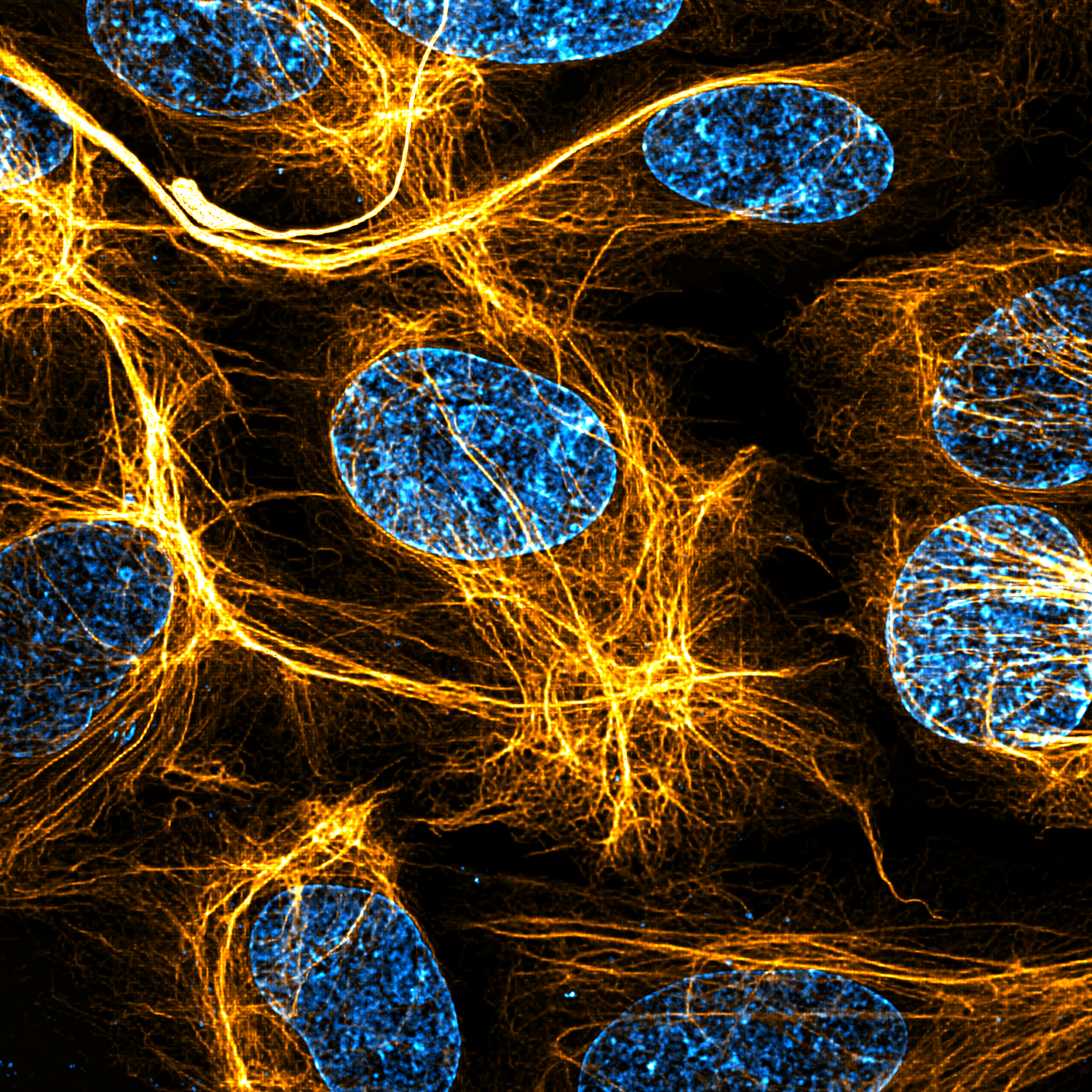

Supplement: Supplementary file 11 — Source data Fig. 5 [file 44318_2024_337_MOESM11_ESM.zip › 05_Figure_05/5D/VIM.tif]

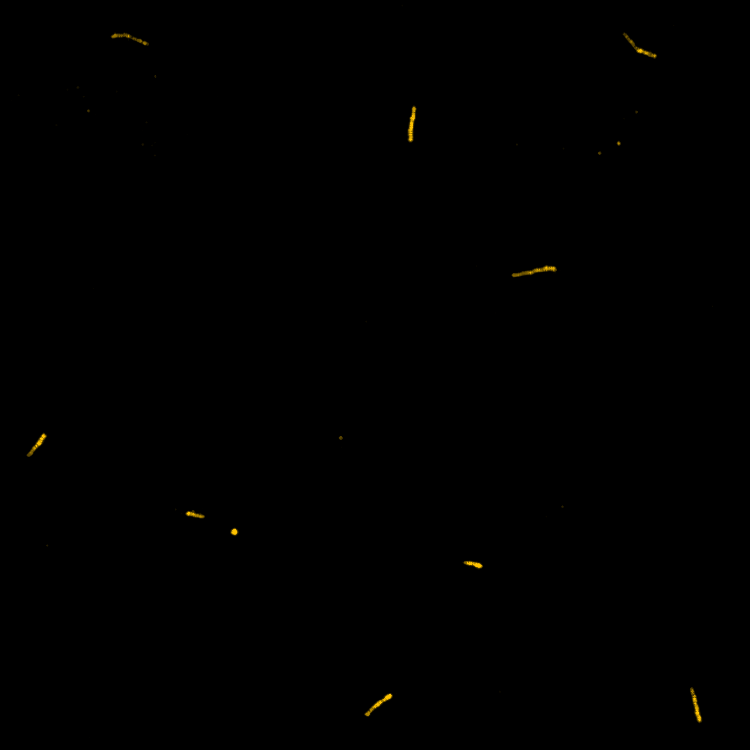

Supplement: Supplementary file 12 — Source data Fig. 6 [file 44318_2024_337_MOESM12_ESM.zip › 06_Figure_06/6C/ARL13B-MINUS-SERUM-CTRL/ARL13B-MINUS-SERUM-CTRL-ARL13B.tif]

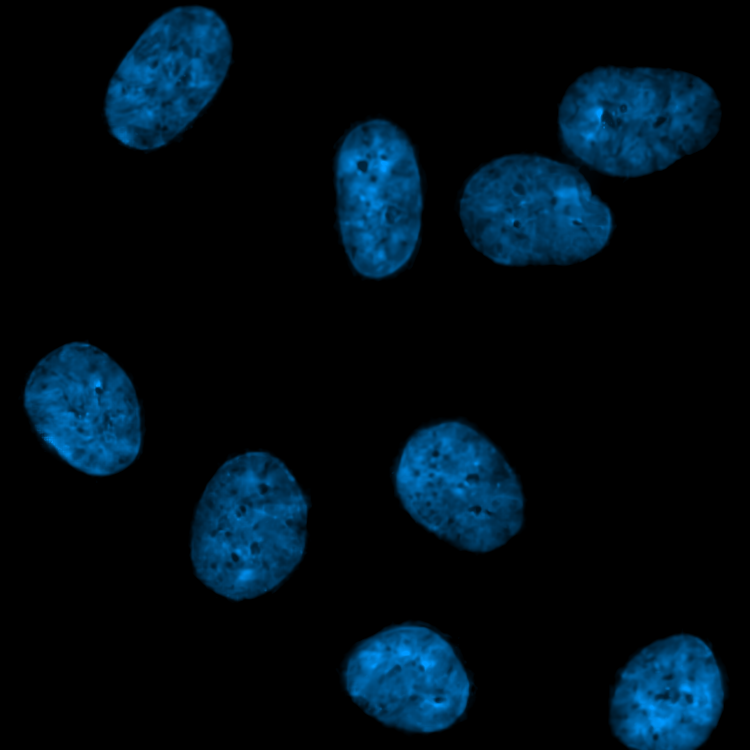

Supplement: Supplementary file 12 — Source data Fig. 6 [file 44318_2024_337_MOESM12_ESM.zip › 06_Figure_06/6C/ARL13B-MINUS-SERUM-CTRL/ARL13B-MINUS-SERUM-CTRL-DAPI.tif]

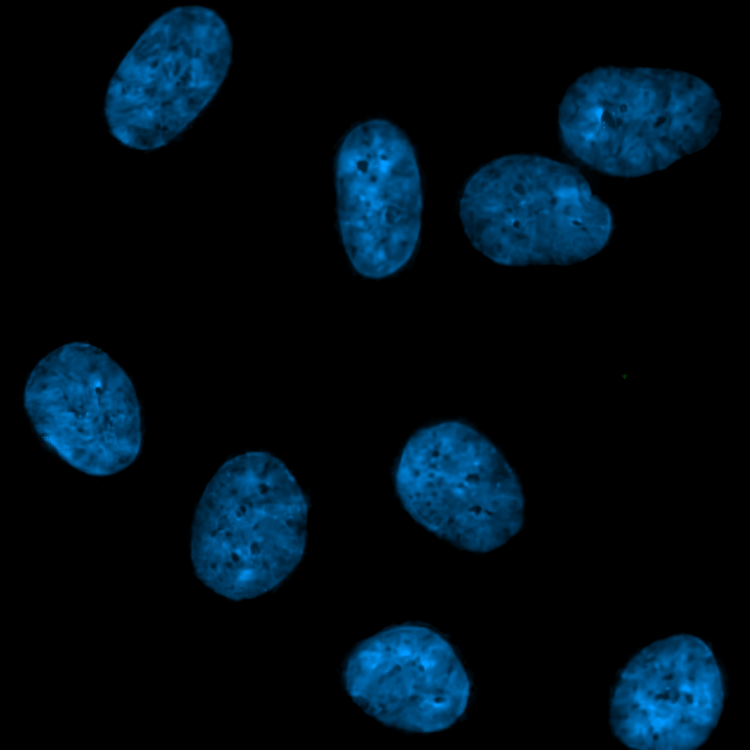

Supplement: Supplementary file 12 — Source data Fig. 6 [file 44318_2024_337_MOESM12_ESM.zip › 06_Figure_06/6C/ARL13B-MINUS-SERUM-CTRL/ARL13B-MINUS-SERUM-CTRL-Merge.tif]

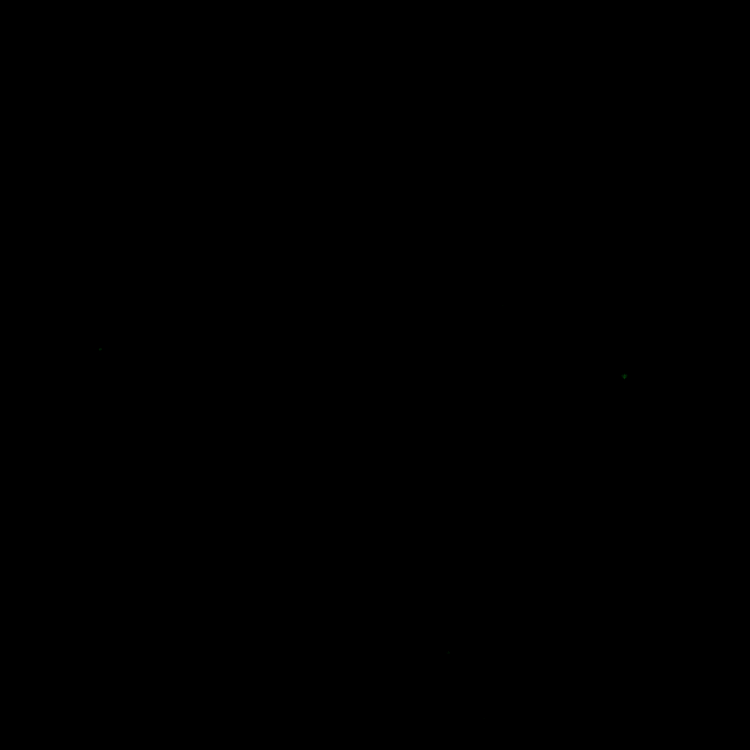

Supplement: Supplementary file 12 — Source data Fig. 6 [file 44318_2024_337_MOESM12_ESM.zip › 06_Figure_06/6C/ARL13B-MINUS-SERUM-CTRL/ARL13B-MINUS-SERUM-CTRL-mStayGold.tif]

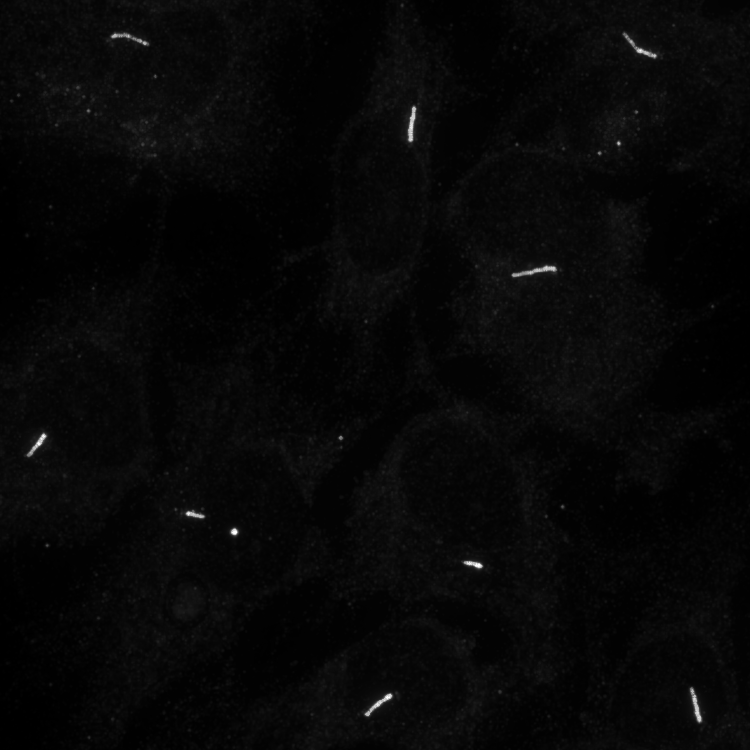

Supplement: Supplementary file 12 — Source data Fig. 6 [file 44318_2024_337_MOESM12_ESM.zip › 06_Figure_06/6C/ARL13B-MINUS-SERUM-CTRL/_FULL-RANGE-ARL13B-MINUS-SERUM-CTRL.tif]

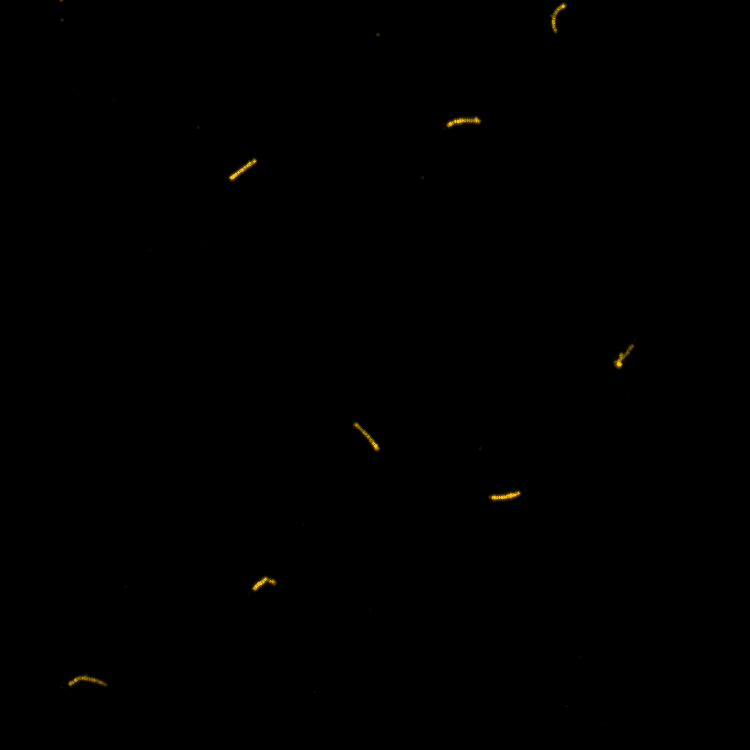

Supplement: Supplementary file 12 — Source data Fig. 6 [file 44318_2024_337_MOESM12_ESM.zip › 06_Figure_06/6C/ARL13B-MINUS-SERUM-STG/ARL13B-MINUS-SERUM-STG-ARL13B.tif]

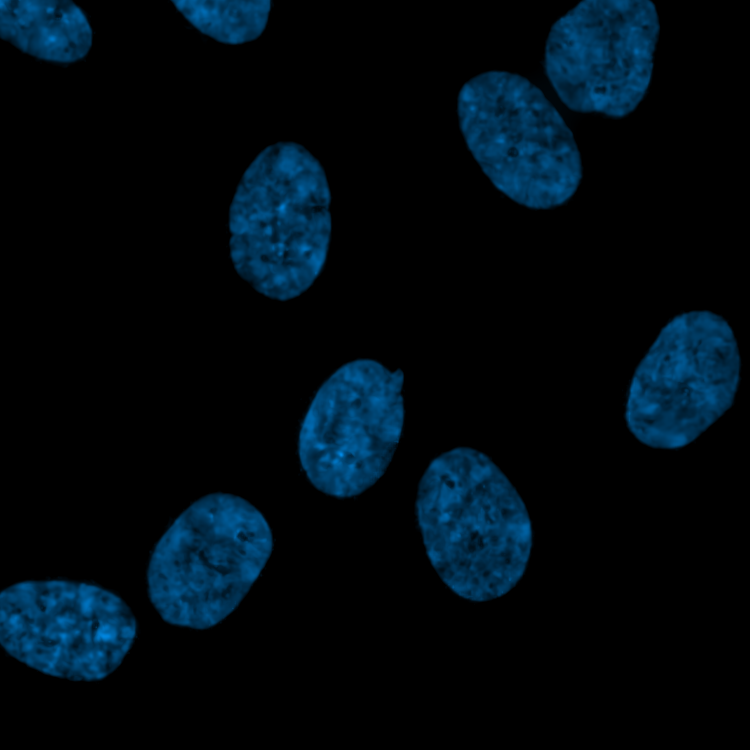

Supplement: Supplementary file 12 — Source data Fig. 6 [file 44318_2024_337_MOESM12_ESM.zip › 06_Figure_06/6C/ARL13B-MINUS-SERUM-STG/ARL13B-MINUS-SERUM-STG-DAPI.tif]

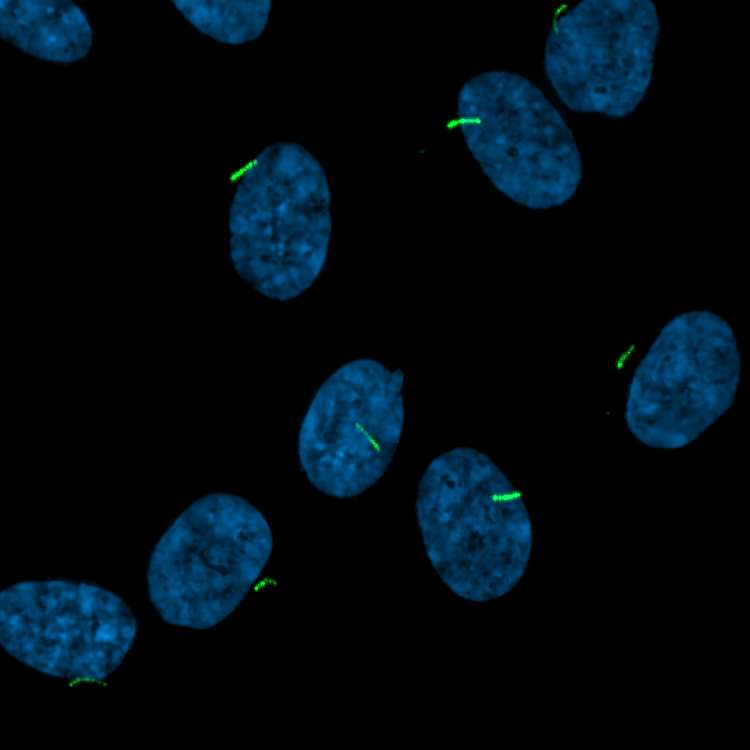

Supplement: Supplementary file 12 — Source data Fig. 6 [file 44318_2024_337_MOESM12_ESM.zip › 06_Figure_06/6C/ARL13B-MINUS-SERUM-STG/ARL13B-MINUS-SERUM-STG-MERGE.tif]

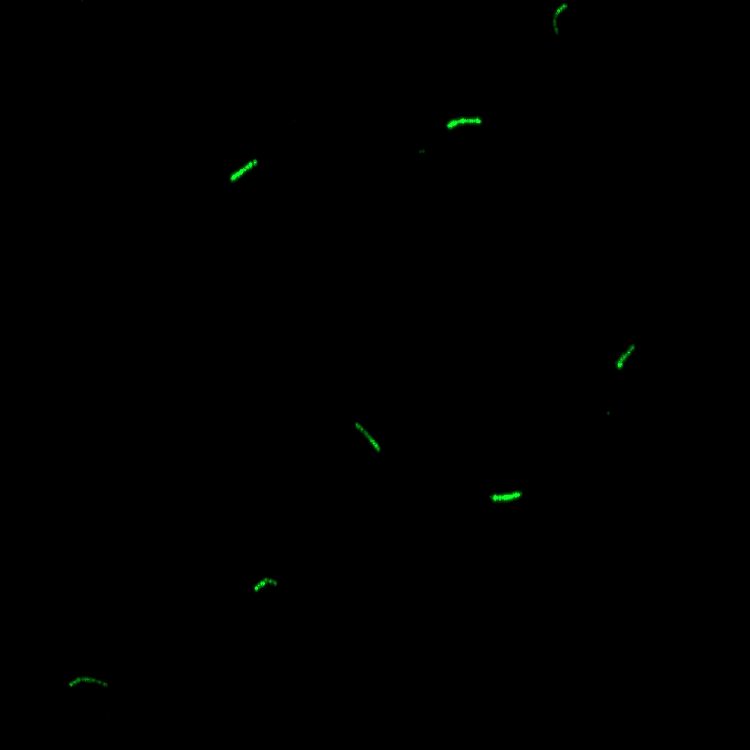

Supplement: Supplementary file 12 — Source data Fig. 6 [file 44318_2024_337_MOESM12_ESM.zip › 06_Figure_06/6C/ARL13B-MINUS-SERUM-STG/ARL13B-MINUS-SERUM-STG-mStayGold.tif]

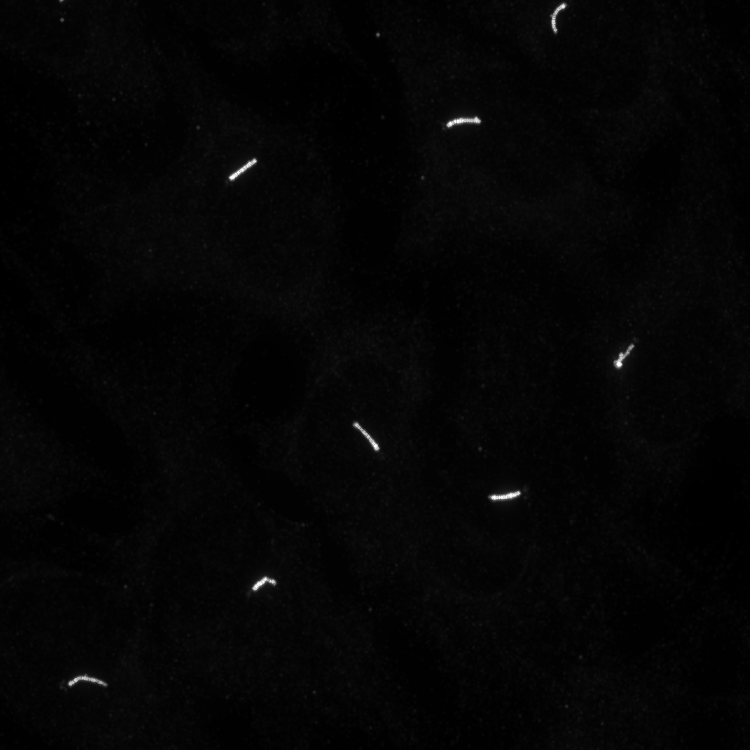

Supplement: Supplementary file 12 — Source data Fig. 6 [file 44318_2024_337_MOESM12_ESM.zip › 06_Figure_06/6C/ARL13B-MINUS-SERUM-STG/_FULL-RANGE-ARL13B-MINUS-SERUM-STG.tif]

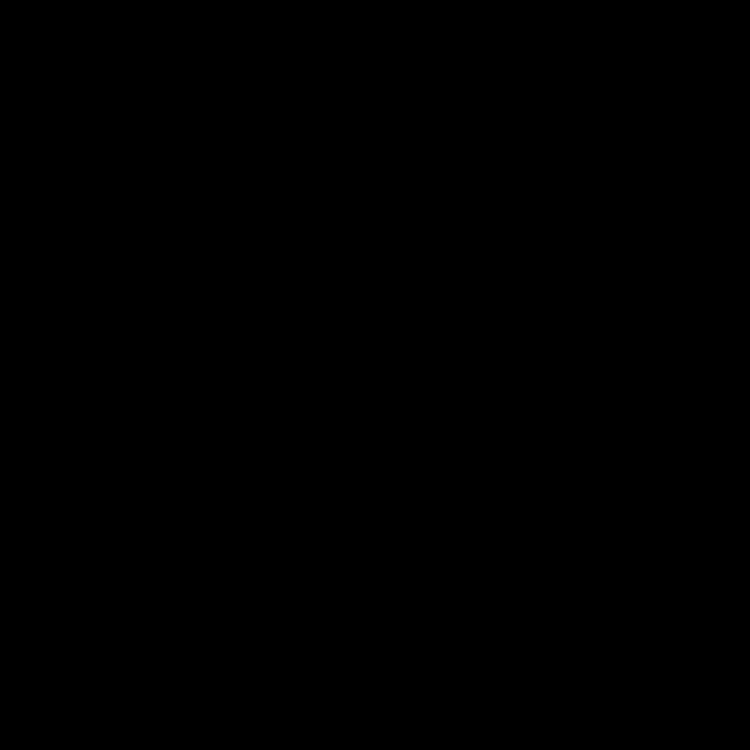

Supplement: Supplementary file 12 — Source data Fig. 6 [file 44318_2024_337_MOESM12_ESM.zip › 06_Figure_06/6C/ARL13B-PLUS-SERUM-CTRL/ARL13B-PLUS-SERUM-CTRL-ARL13B.tif]

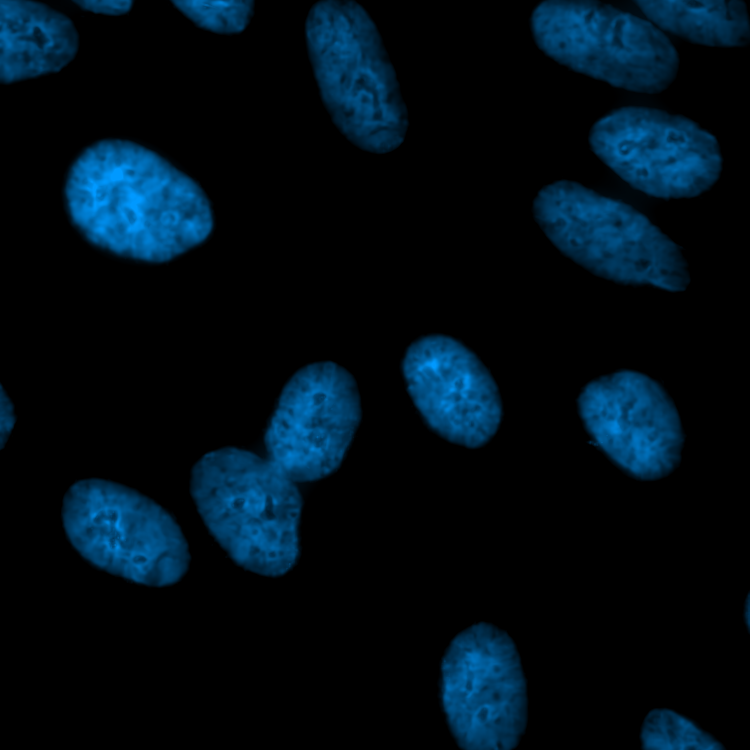

Supplement: Supplementary file 12 — Source data Fig. 6 [file 44318_2024_337_MOESM12_ESM.zip › 06_Figure_06/6C/ARL13B-PLUS-SERUM-CTRL/ARL13B-PLUS-SERUM-CTRL-DAPI.tif]

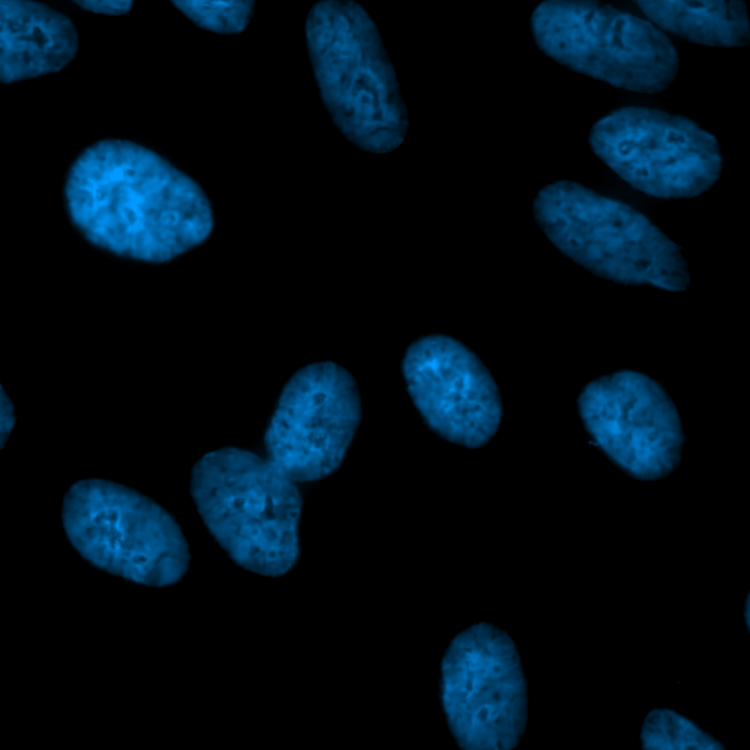

Supplement: Supplementary file 12 — Source data Fig. 6 [file 44318_2024_337_MOESM12_ESM.zip › 06_Figure_06/6C/ARL13B-PLUS-SERUM-CTRL/ARL13B-PLUS-SERUM-CTRL-Merge.tif]

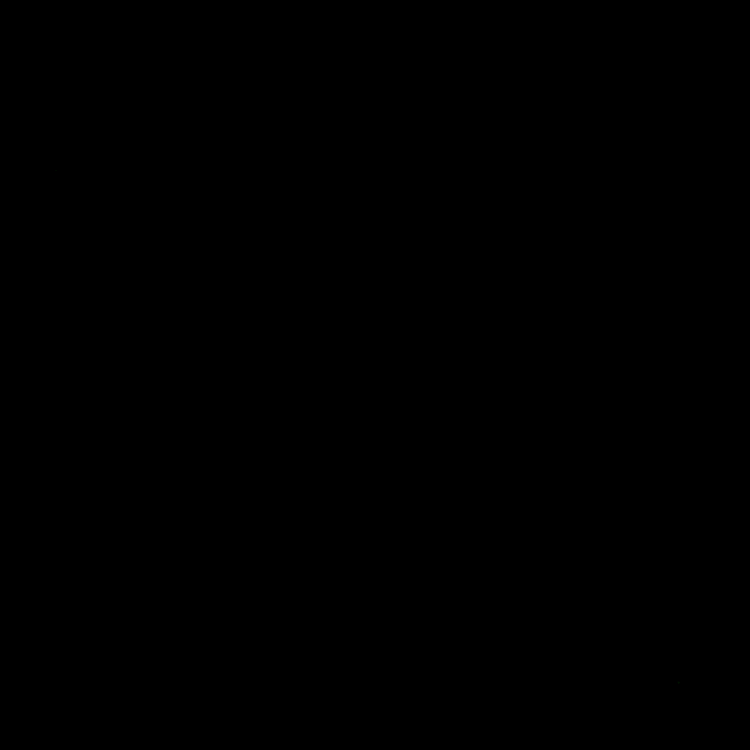

Supplement: Supplementary file 12 — Source data Fig. 6 [file 44318_2024_337_MOESM12_ESM.zip › 06_Figure_06/6C/ARL13B-PLUS-SERUM-CTRL/ARL13B-PLUS-SERUM-CTRL-mStayGold.tif]

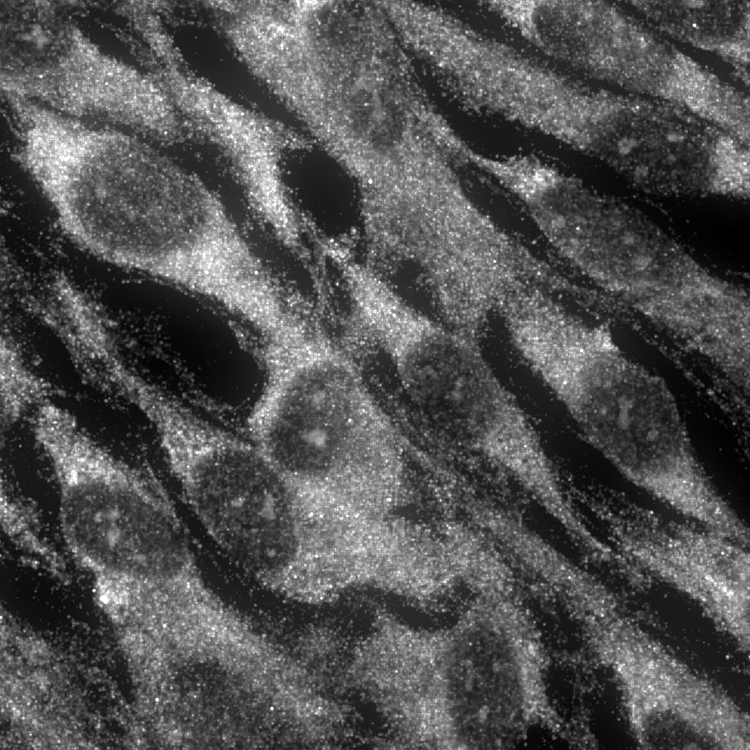

Supplement: Supplementary file 12 — Source data Fig. 6 [file 44318_2024_337_MOESM12_ESM.zip › 06_Figure_06/6C/ARL13B-PLUS-SERUM-CTRL/_FULL-RANGE-ARL13B-PLUS-SERUM-CTRL.tif]

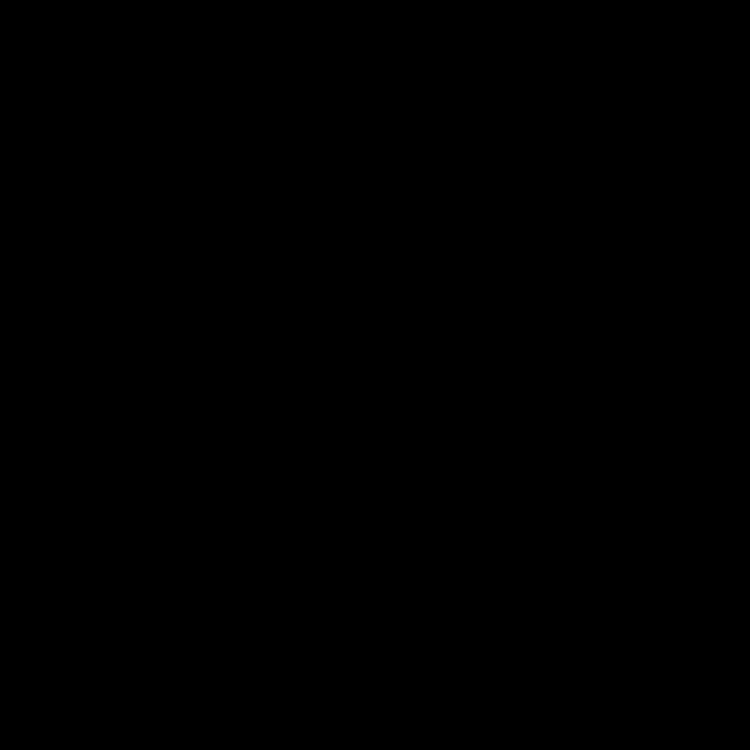

Supplement: Supplementary file 12 — Source data Fig. 6 [file 44318_2024_337_MOESM12_ESM.zip › 06_Figure_06/6C/ARL13B-PLUS-SERUM-STG/ARL13B-PLUS-SERUM-STG-ARL13B.tif]
