# Supplementary figures and images for: qTAG: an adaptable plasmid scaffold for CRISPR-based endogenous tagging (part 3 of 5)
Source: EMBO J. 2024 Dec 12;44(3):947–74. doi: 10.1038/s44318-024-00337-5 (PMC11790981; doi:10.1038/s44318-024-00337-5)

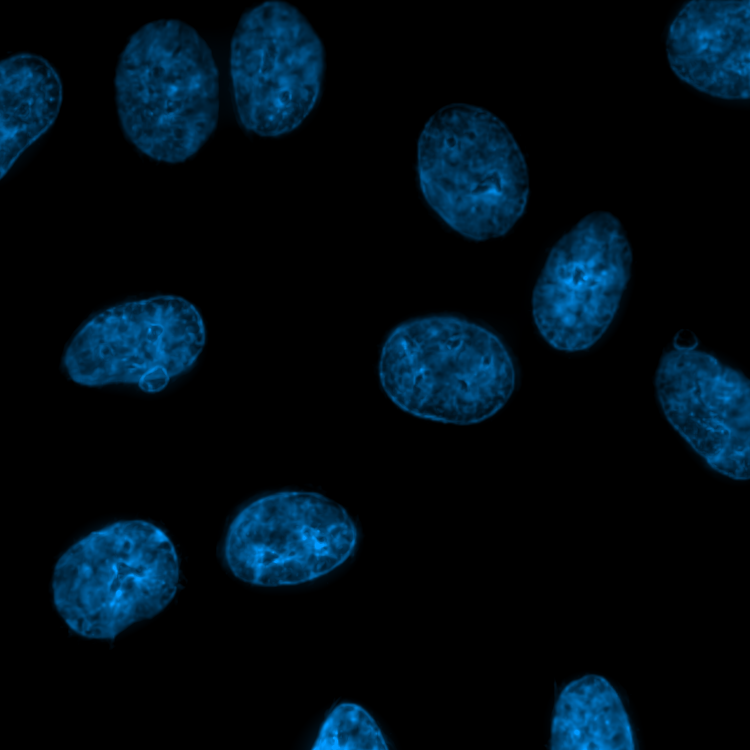

Supplement: Supplementary file 12 — Source data Fig. 6 [file 44318_2024_337_MOESM12_ESM.zip › 06_Figure_06/6C/ARL13B-PLUS-SERUM-STG/ARL13B-PLUS-SERUM-STG-DAPI.tif]

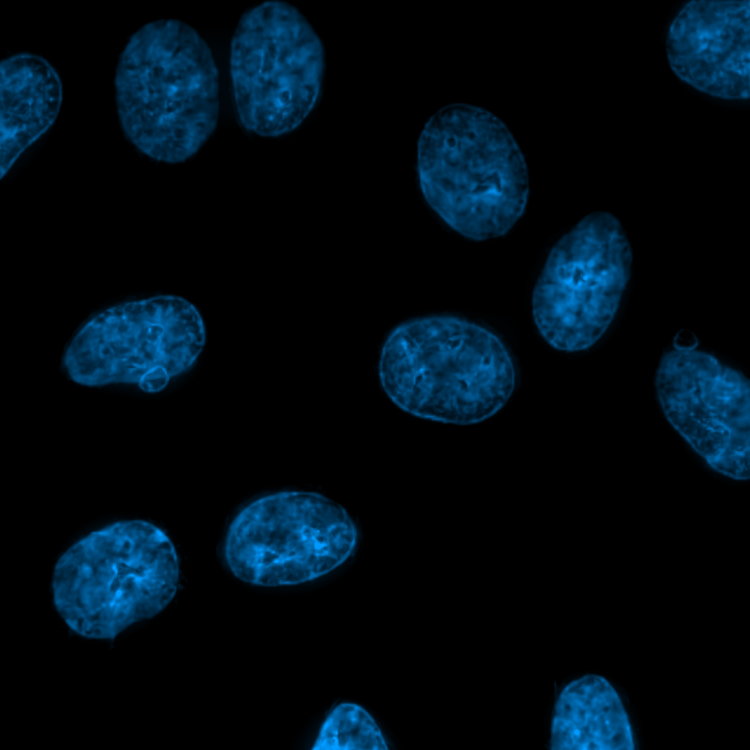

Supplement: Supplementary file 12 — Source data Fig. 6 [file 44318_2024_337_MOESM12_ESM.zip › 06_Figure_06/6C/ARL13B-PLUS-SERUM-STG/ARL13B-PLUS-SERUM-STG-Merge.tif]

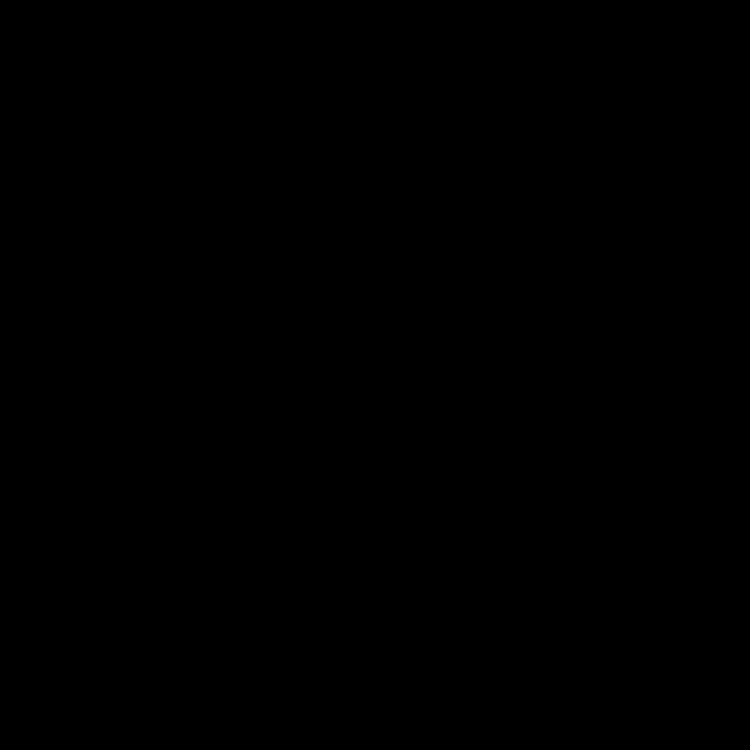

Supplement: Supplementary file 12 — Source data Fig. 6 [file 44318_2024_337_MOESM12_ESM.zip › 06_Figure_06/6C/ARL13B-PLUS-SERUM-STG/ARL13B-PLUS-SERUM-STG-mStayGold.tif]

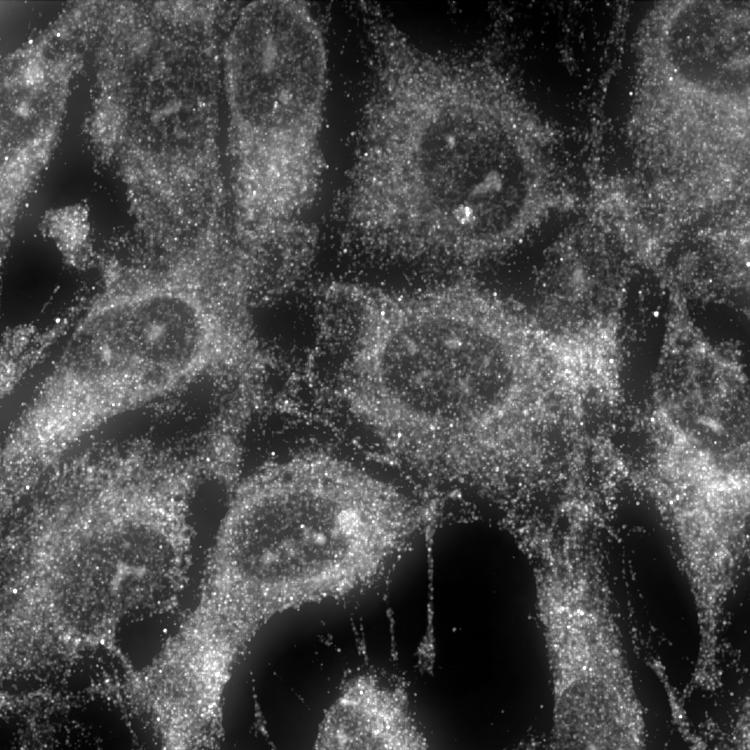

Supplement: Supplementary file 12 — Source data Fig. 6 [file 44318_2024_337_MOESM12_ESM.zip › 06_Figure_06/6C/ARL13B-PLUS-SERUM-STG/_FULL-RANGE-ARL13B-PLUS-SERUM-STG.tif]

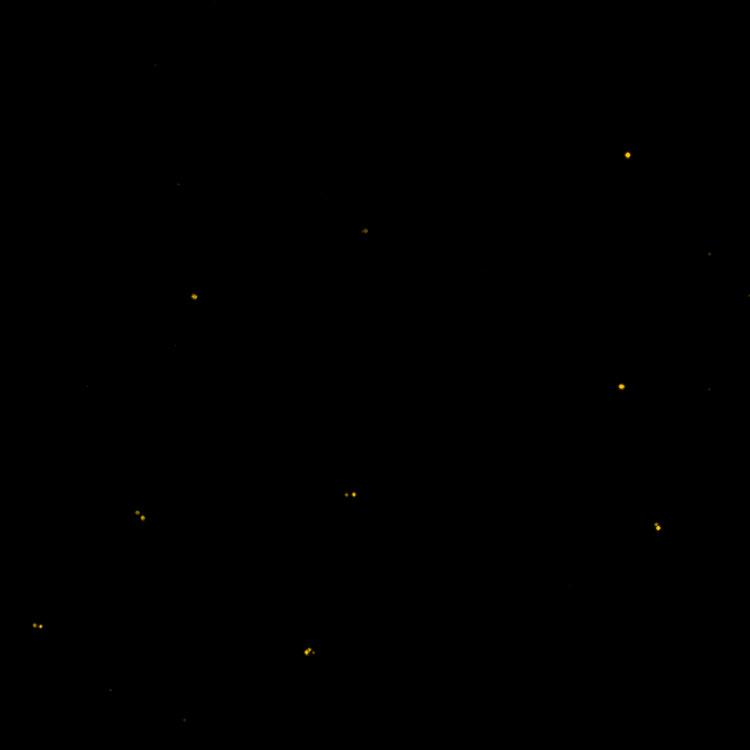

Supplement: Supplementary file 12 — Source data Fig. 6 [file 44318_2024_337_MOESM12_ESM.zip › 06_Figure_06/6E/Images/PLK4-CTRL/PLK4-CTRL-CEP135.tif]

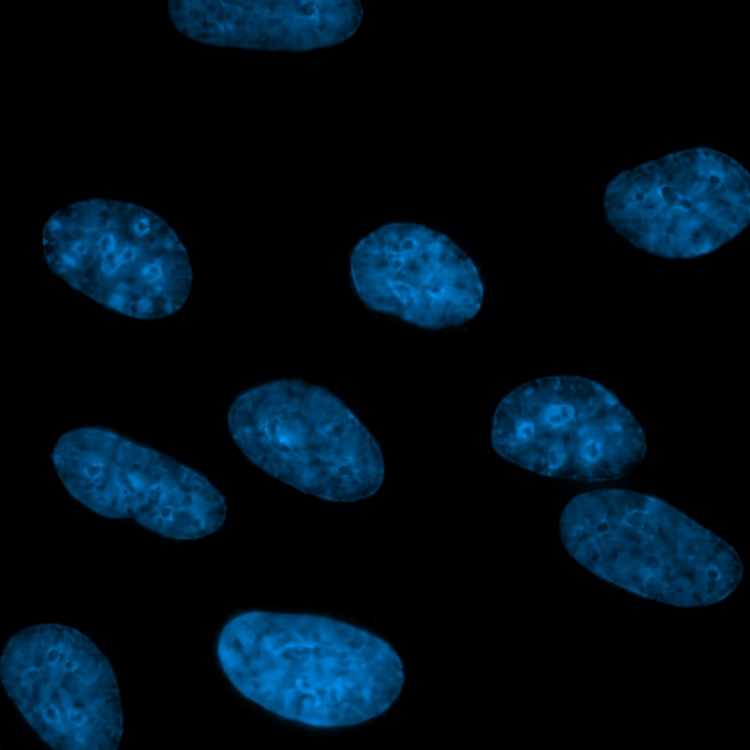

Supplement: Supplementary file 12 — Source data Fig. 6 [file 44318_2024_337_MOESM12_ESM.zip › 06_Figure_06/6E/Images/PLK4-CTRL/PLK4-CTRL-DAPI.tif]

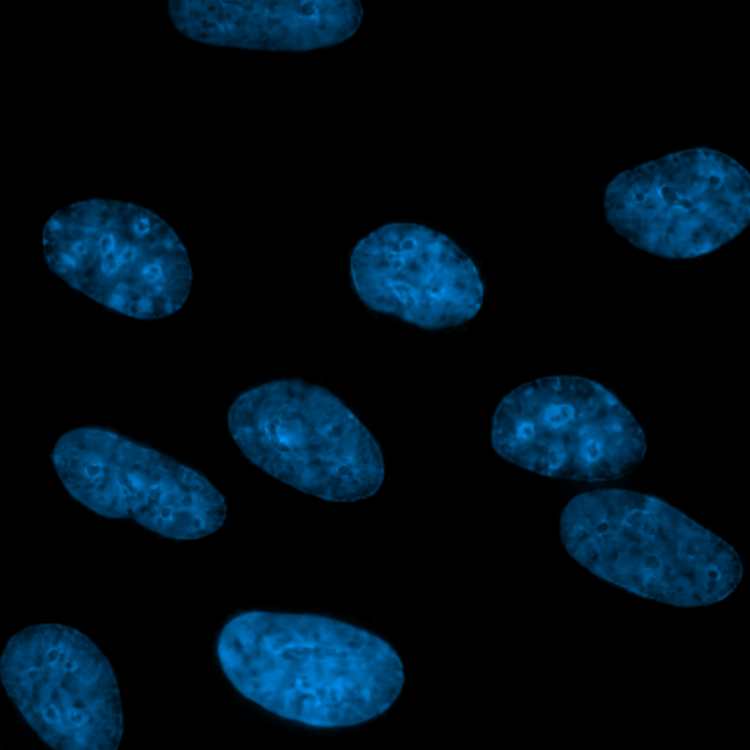

Supplement: Supplementary file 12 — Source data Fig. 6 [file 44318_2024_337_MOESM12_ESM.zip › 06_Figure_06/6E/Images/PLK4-CTRL/PLK4-CTRL-MErge.tif]

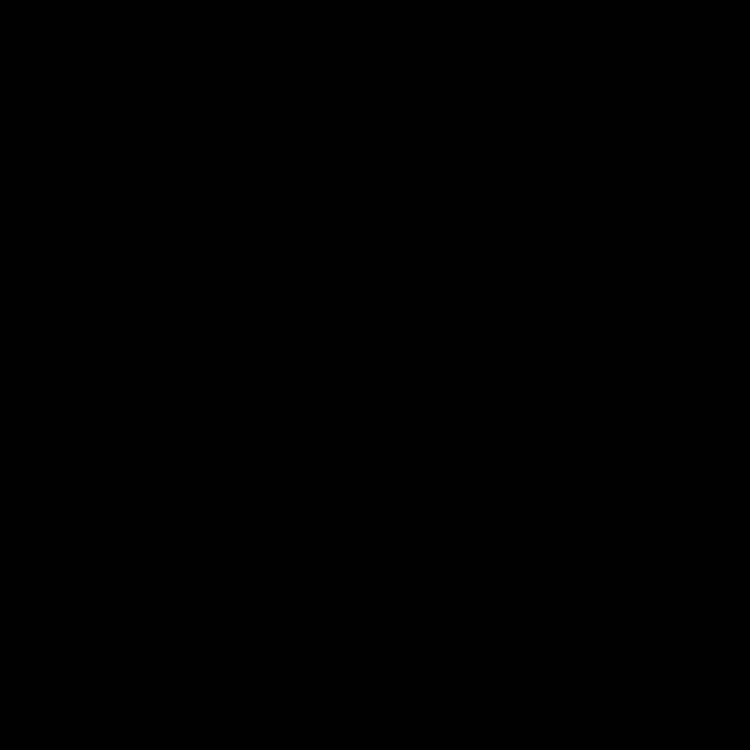

Supplement: Supplementary file 12 — Source data Fig. 6 [file 44318_2024_337_MOESM12_ESM.zip › 06_Figure_06/6E/Images/PLK4-CTRL/PLK4-CTRL-mStayGold.tif]

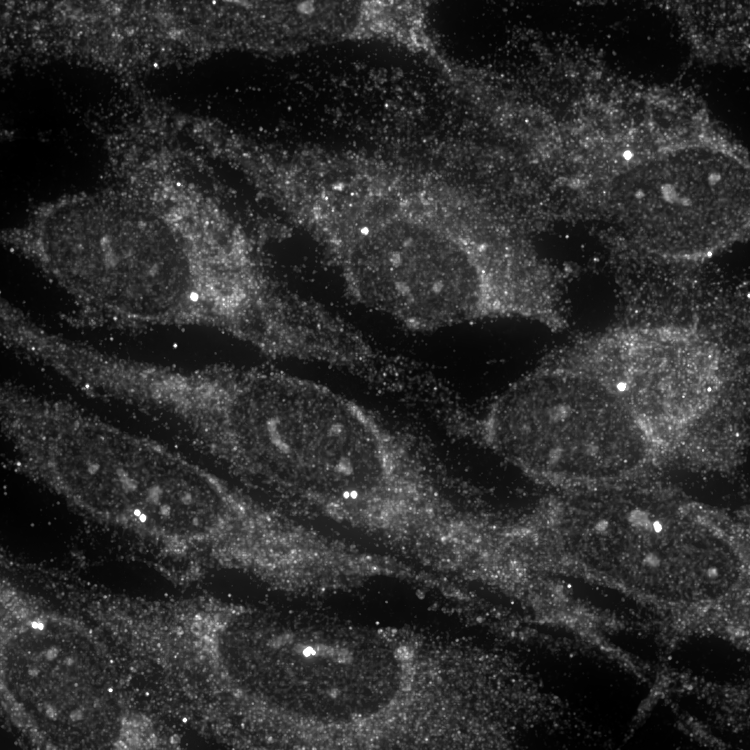

Supplement: Supplementary file 12 — Source data Fig. 6 [file 44318_2024_337_MOESM12_ESM.zip › 06_Figure_06/6E/Images/PLK4-CTRL/_FULL-RANGE-PLK4-CTRL.tif]

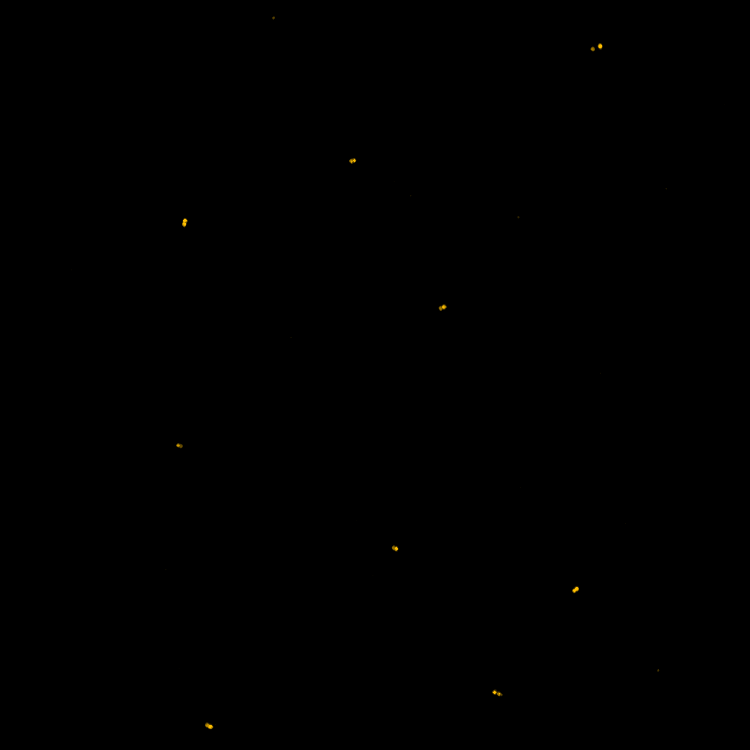

Supplement: Supplementary file 12 — Source data Fig. 6 [file 44318_2024_337_MOESM12_ESM.zip › 06_Figure_06/6E/Images/PLK4-STG/PLK4-STG-CEP135.tif]

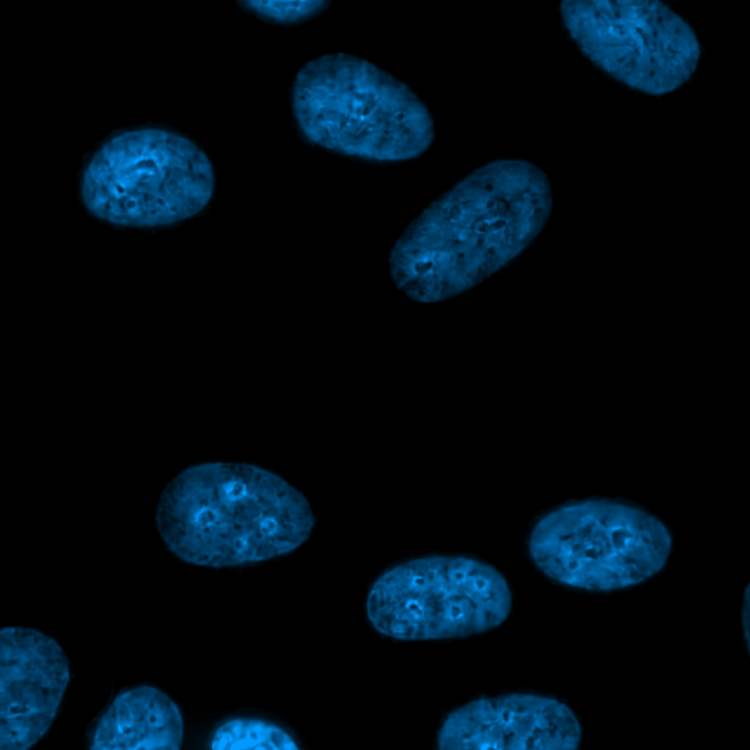

Supplement: Supplementary file 12 — Source data Fig. 6 [file 44318_2024_337_MOESM12_ESM.zip › 06_Figure_06/6E/Images/PLK4-STG/PLK4-STG-DAPI.tif]

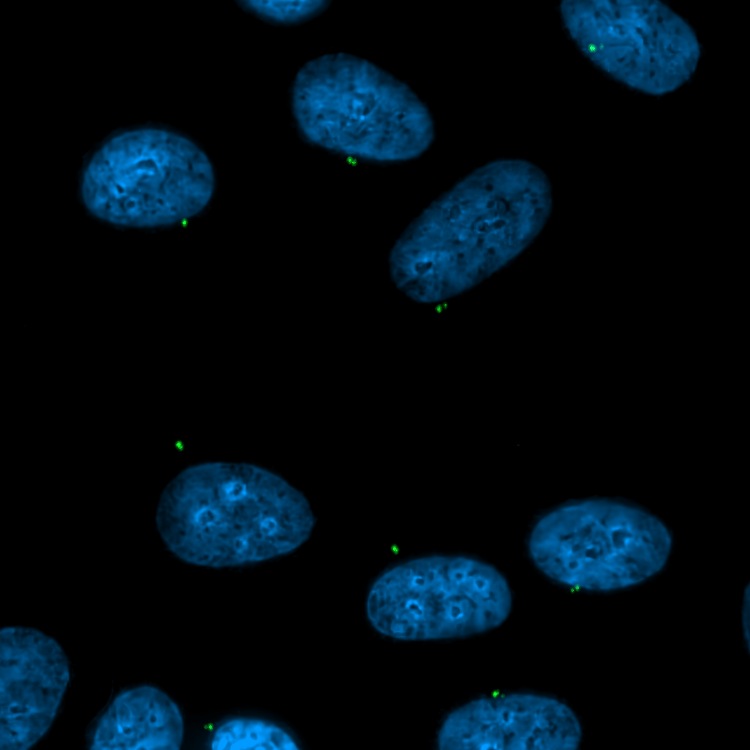

Supplement: Supplementary file 12 — Source data Fig. 6 [file 44318_2024_337_MOESM12_ESM.zip › 06_Figure_06/6E/Images/PLK4-STG/PLK4-STG-Merge.tif]

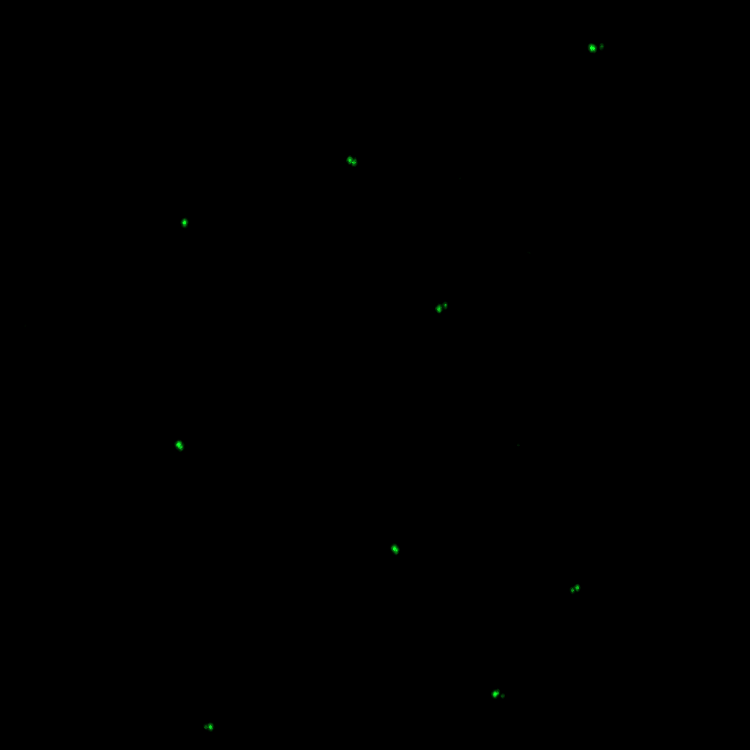

Supplement: Supplementary file 12 — Source data Fig. 6 [file 44318_2024_337_MOESM12_ESM.zip › 06_Figure_06/6E/Images/PLK4-STG/PLK4-STG-mStayGold.tif]

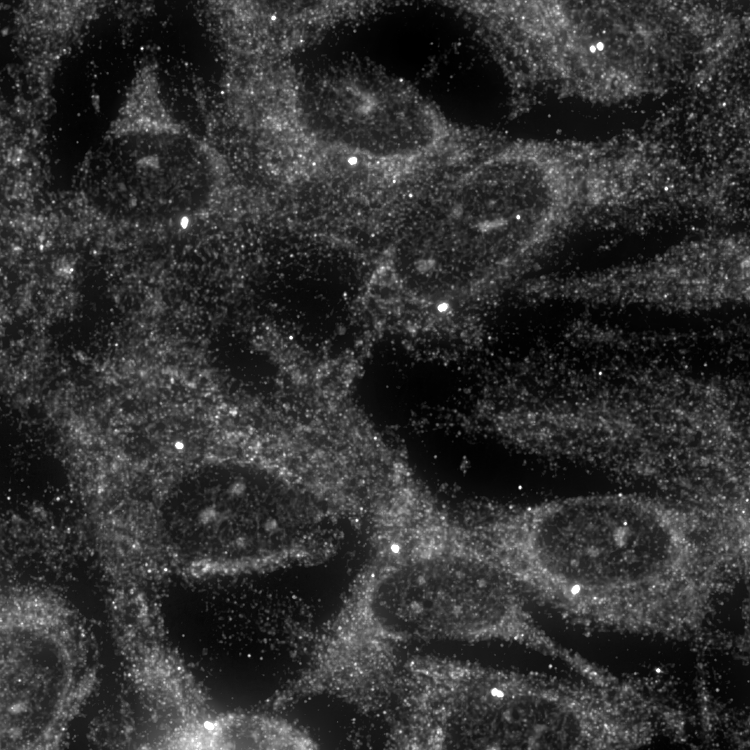

Supplement: Supplementary file 12 — Source data Fig. 6 [file 44318_2024_337_MOESM12_ESM.zip › 06_Figure_06/6E/Images/PLK4-STG/_FULL-RANGE-PLK4-STG.tif]

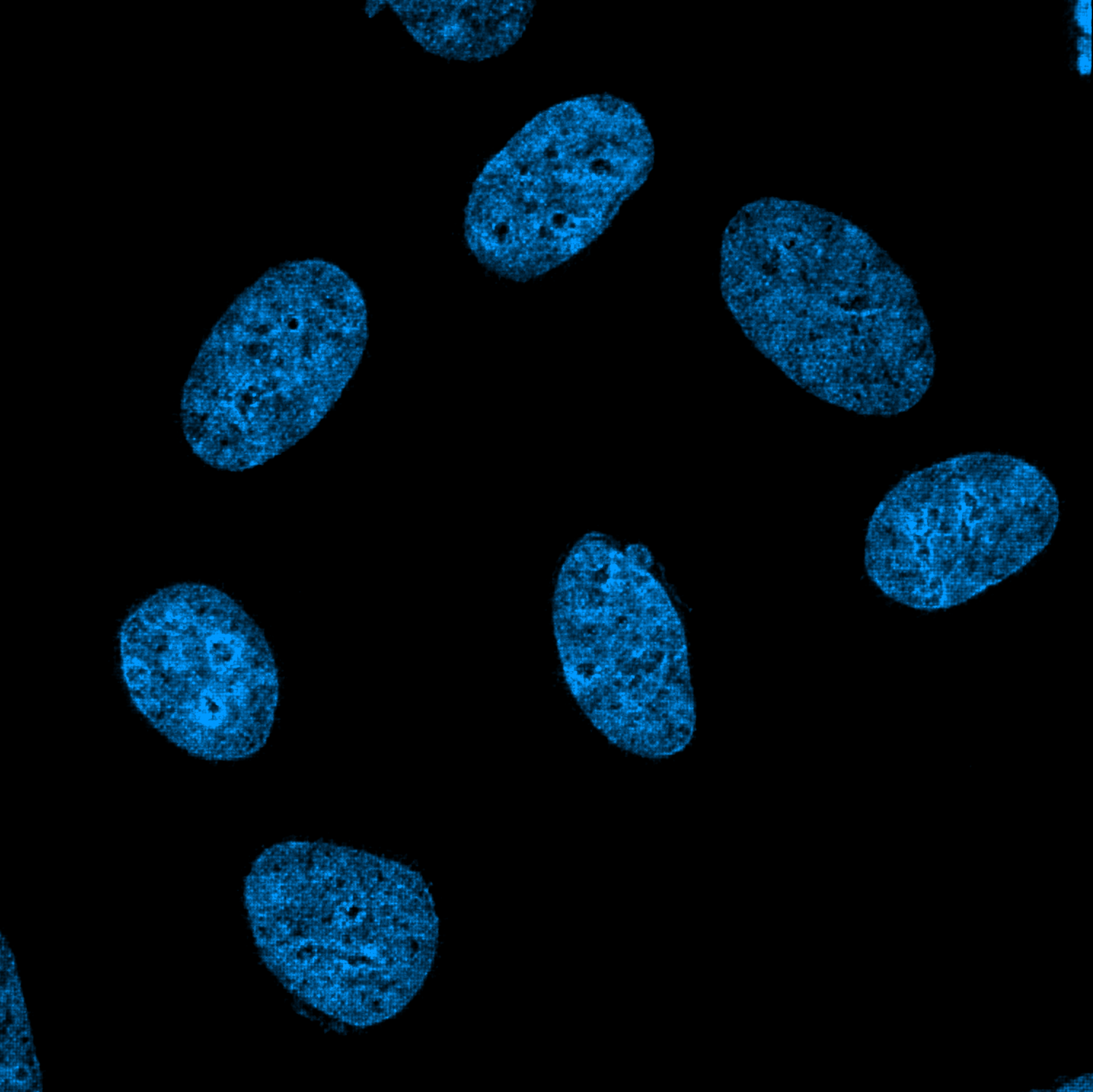

Supplement: Supplementary file 12 — Source data Fig. 6 [file 44318_2024_337_MOESM12_ESM.zip › 06_Figure_06/6H/TP53-gRNA1-CLONE+N/TP53-G1-CLONE+N_DAPI.tif]

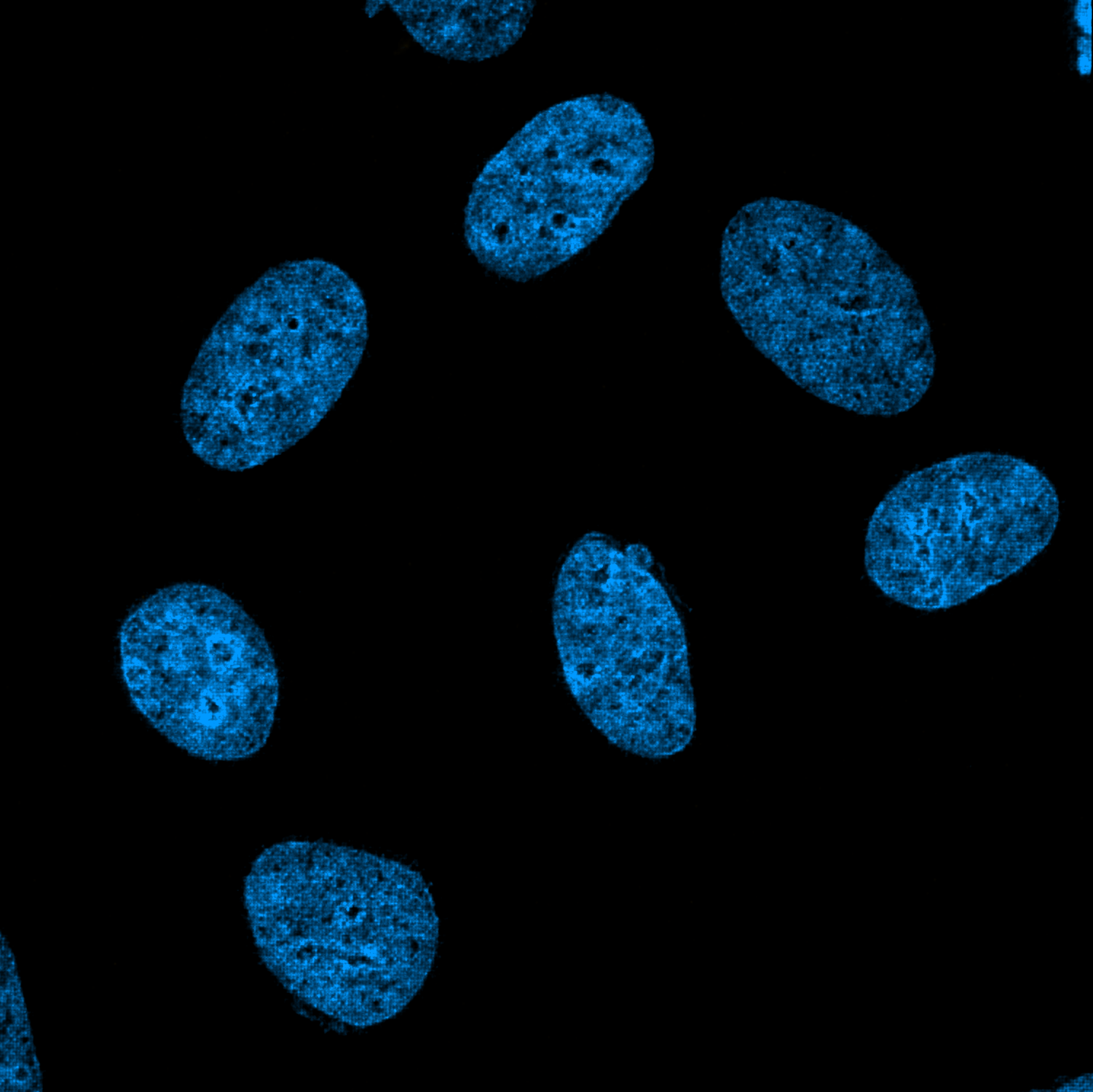

Supplement: Supplementary file 12 — Source data Fig. 6 [file 44318_2024_337_MOESM12_ESM.zip › 06_Figure_06/6H/TP53-gRNA1-CLONE+N/TP53-G1-CLONE+N_Merge.tif]

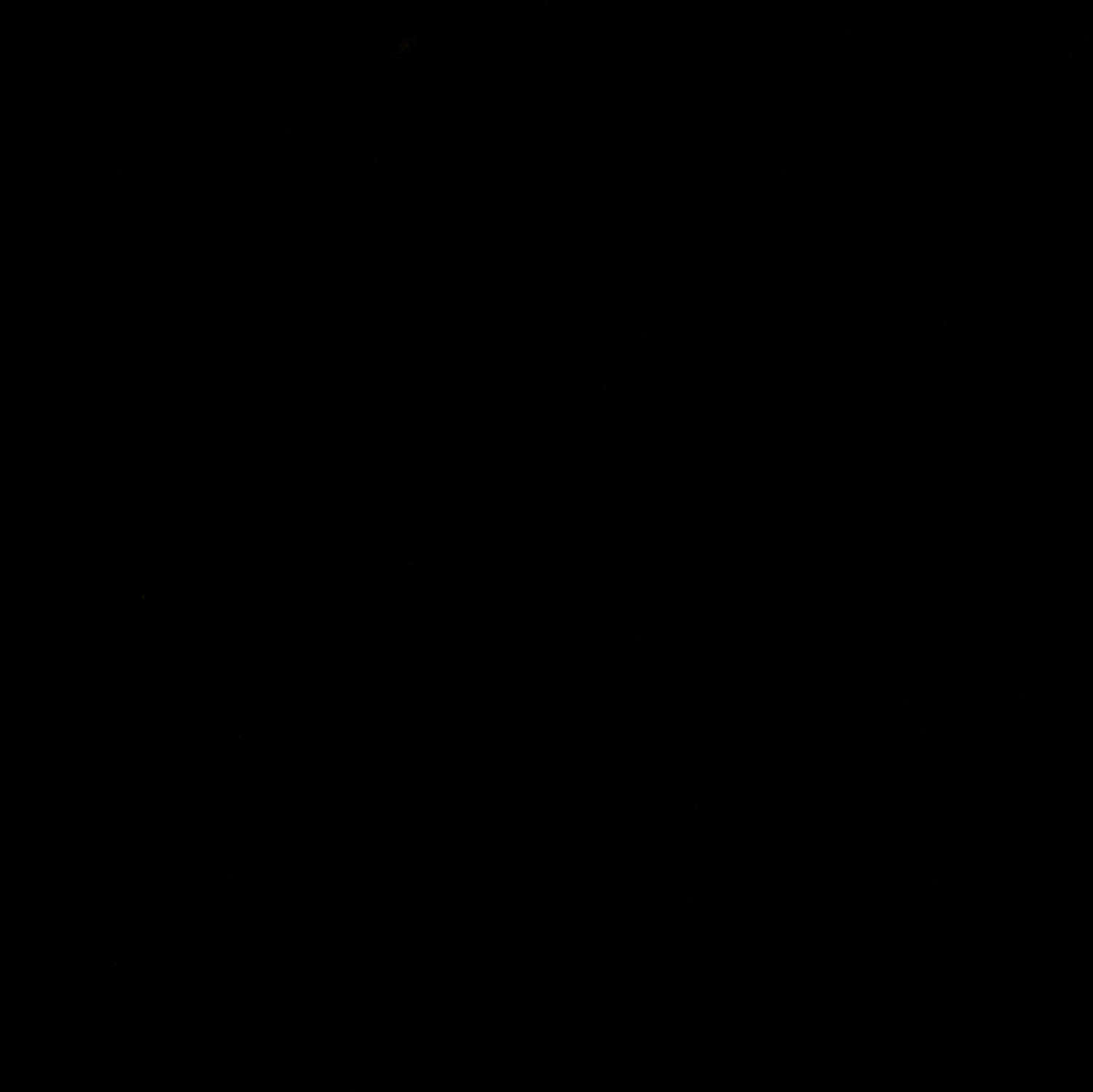

Supplement: Supplementary file 12 — Source data Fig. 6 [file 44318_2024_337_MOESM12_ESM.zip › 06_Figure_06/6H/TP53-gRNA1-CLONE+N/TP53-G1-CLONE+N_RGB_TP53.tif]

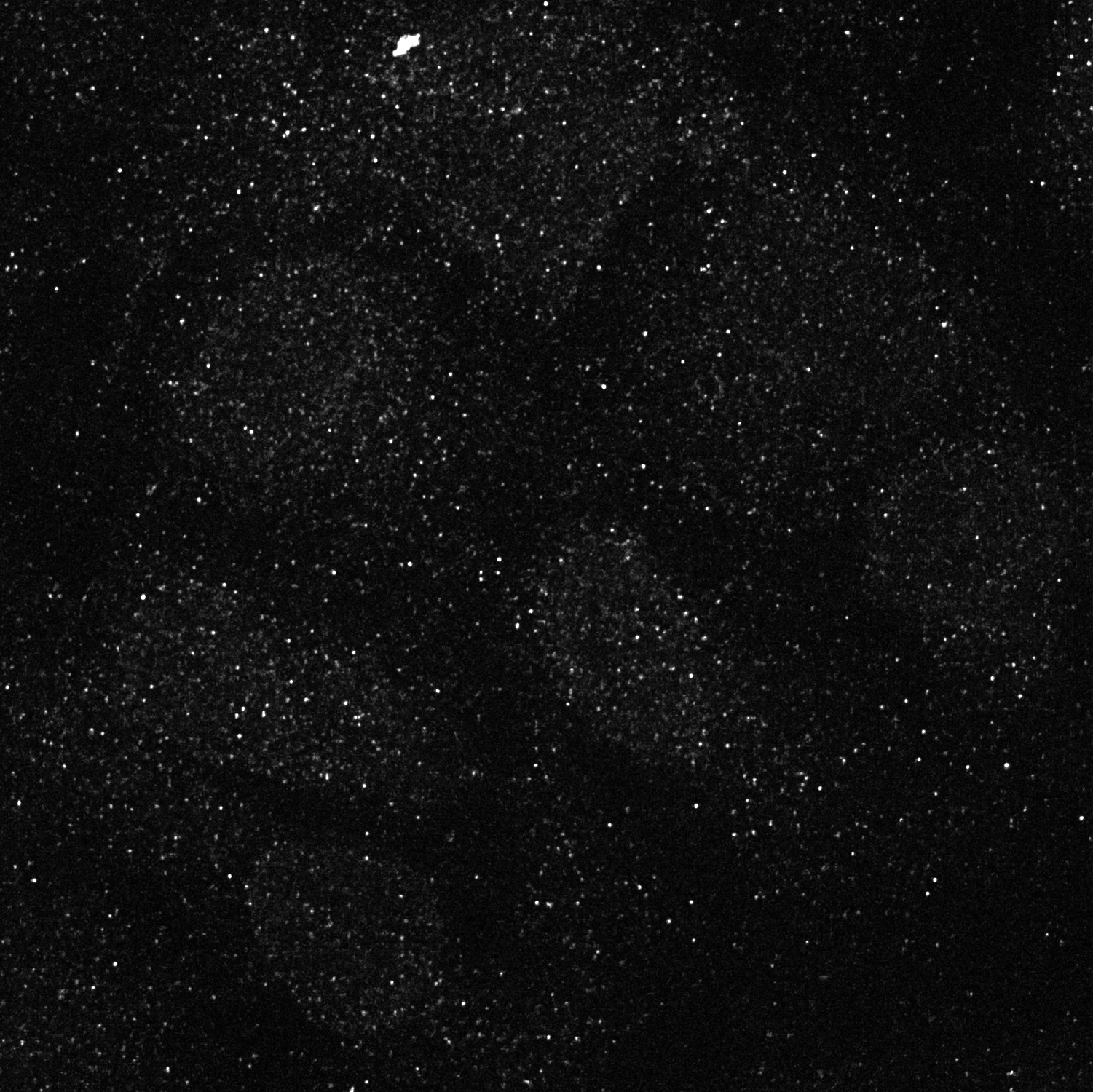

Supplement: Supplementary file 12 — Source data Fig. 6 [file 44318_2024_337_MOESM12_ESM.zip › 06_Figure_06/6H/TP53-gRNA1-CLONE+N/_FULL-RANGE-TP53-G1-CLONE+N.tif]

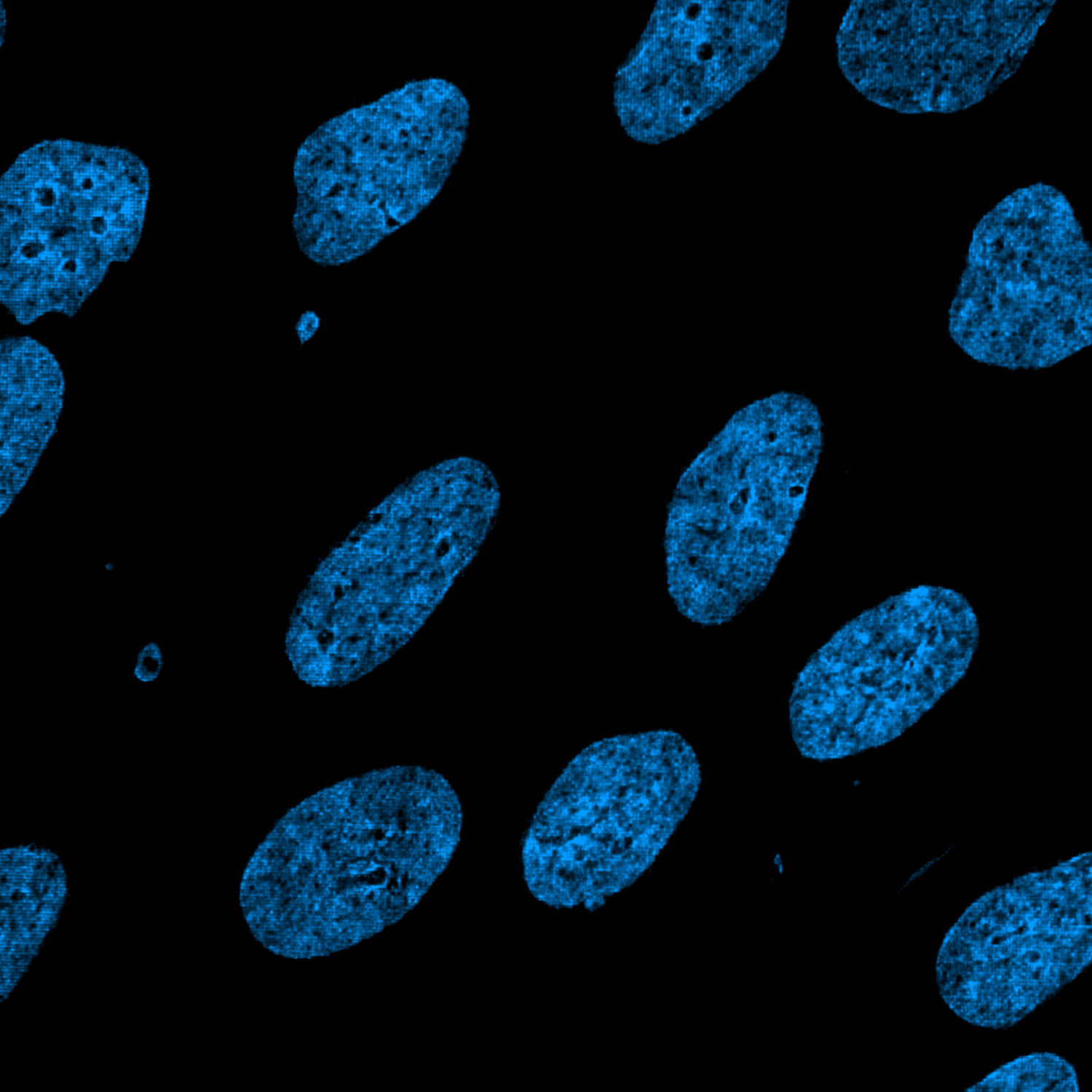

Supplement: Supplementary file 12 — Source data Fig. 6 [file 44318_2024_337_MOESM12_ESM.zip › 06_Figure_06/6H/TP53-gRNA1-CLONE-N/TP53-G1-CLONE-N_DAPI.tif]

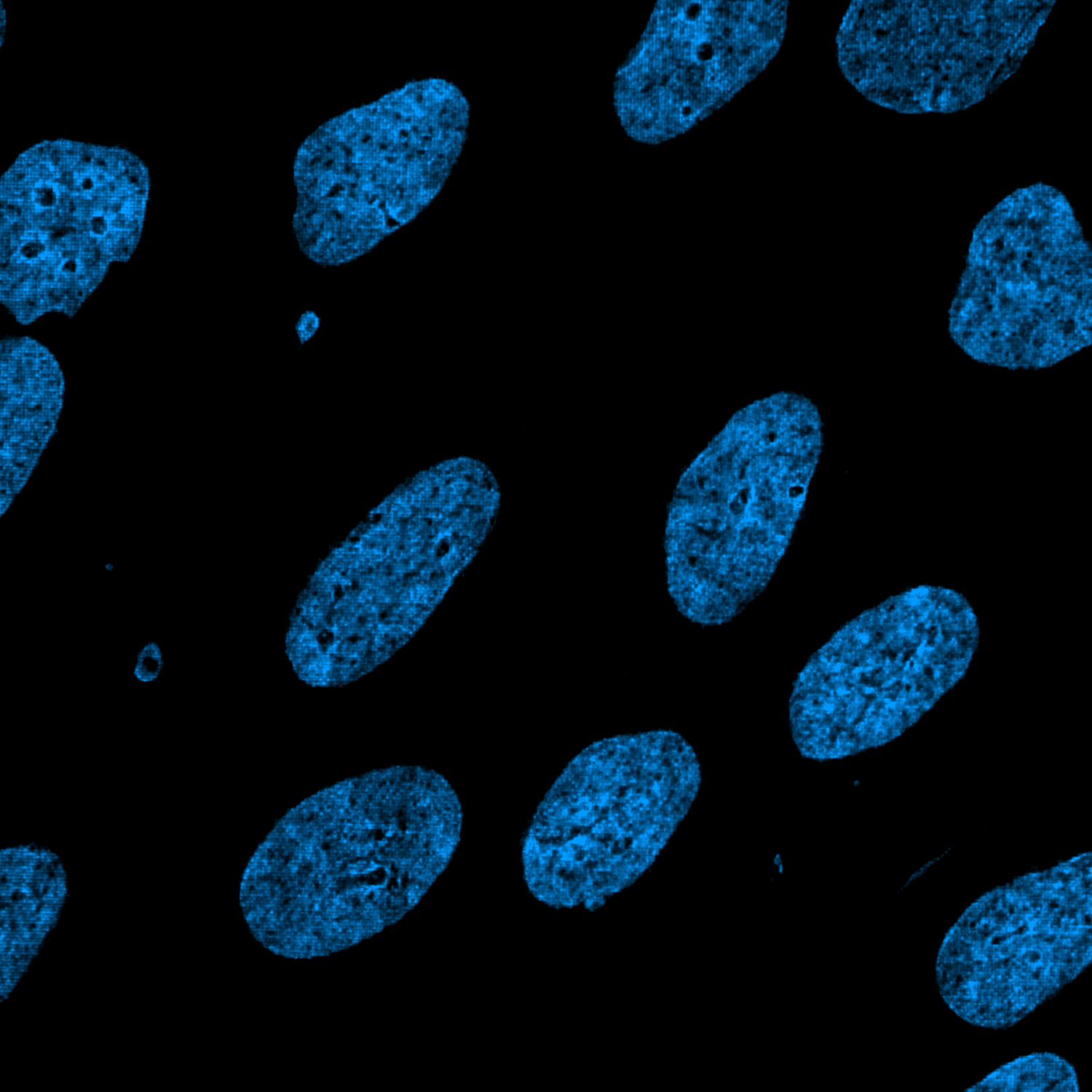

Supplement: Supplementary file 12 — Source data Fig. 6 [file 44318_2024_337_MOESM12_ESM.zip › 06_Figure_06/6H/TP53-gRNA1-CLONE-N/TP53-G1-CLONE-N_Merge.tif]

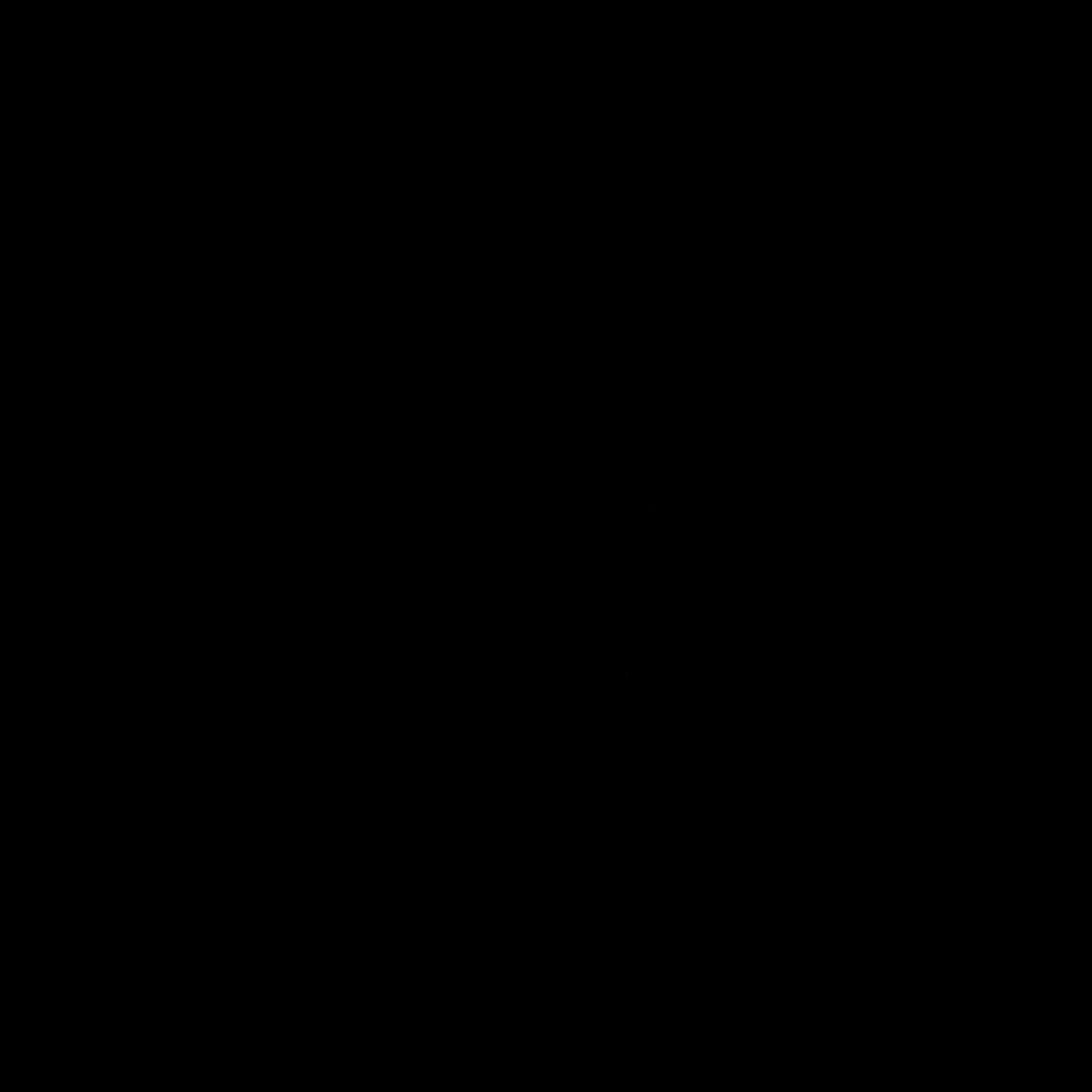

Supplement: Supplementary file 12 — Source data Fig. 6 [file 44318_2024_337_MOESM12_ESM.zip › 06_Figure_06/6H/TP53-gRNA1-CLONE-N/TP53-G1-CLONE-N_TP53.tif]

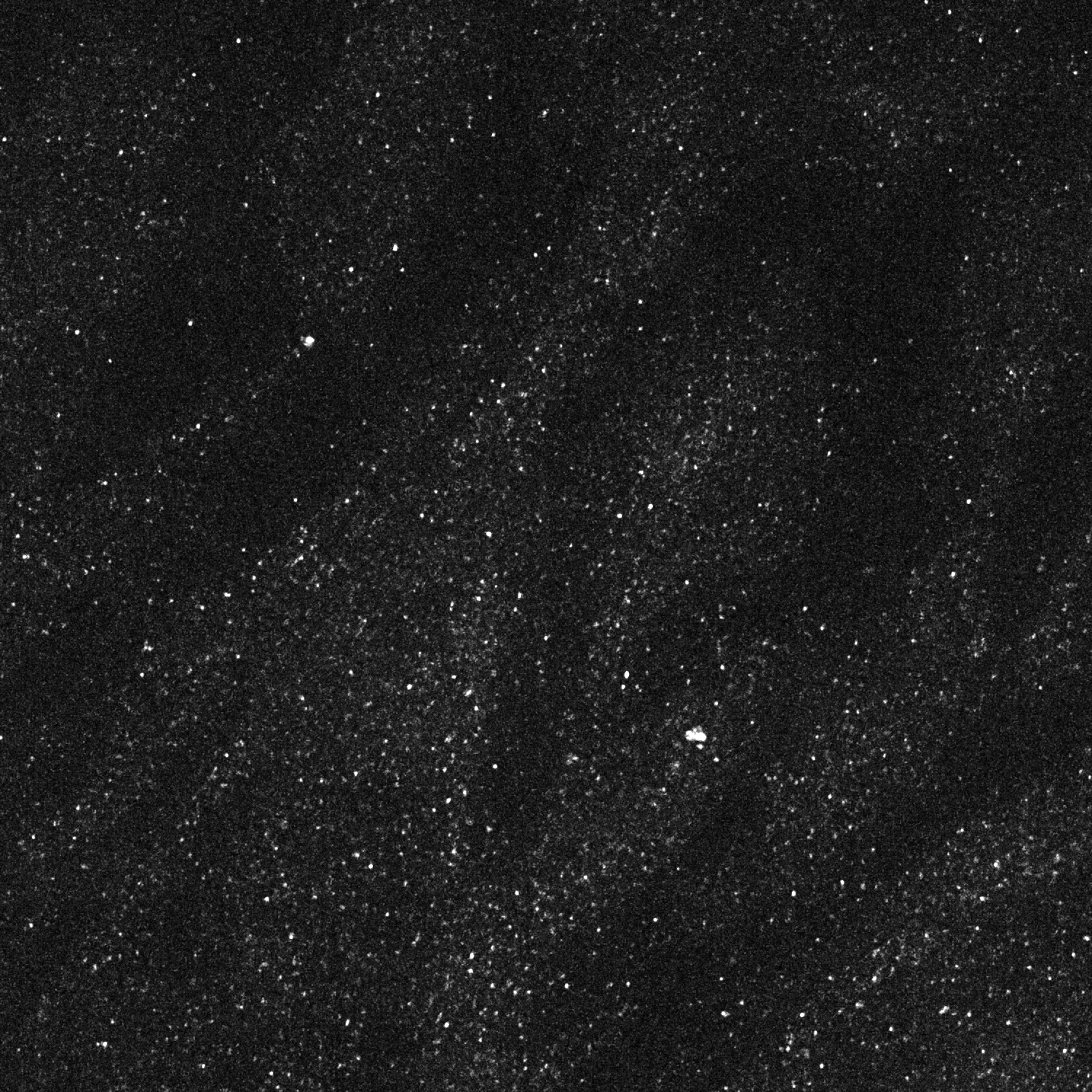

Supplement: Supplementary file 12 — Source data Fig. 6 [file 44318_2024_337_MOESM12_ESM.zip › 06_Figure_06/6H/TP53-gRNA1-CLONE-N/_FULL-RANGE-TP53-G1-CLONE-N.tif]

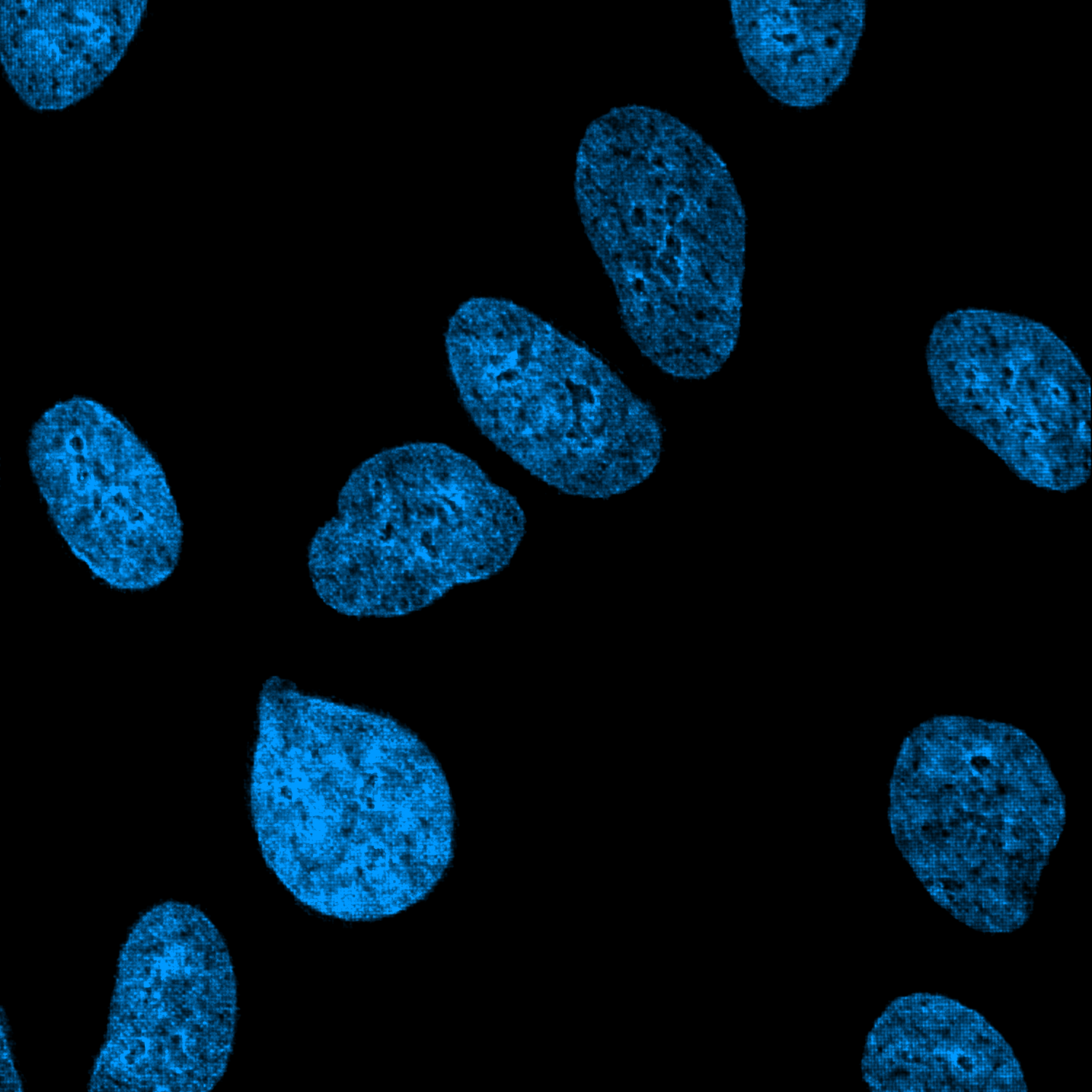

Supplement: Supplementary file 12 — Source data Fig. 6 [file 44318_2024_337_MOESM12_ESM.zip › 06_Figure_06/6H/TP53-gRNA1-POOL+N/TP53-G1-POOL+N_DAPI.tif]

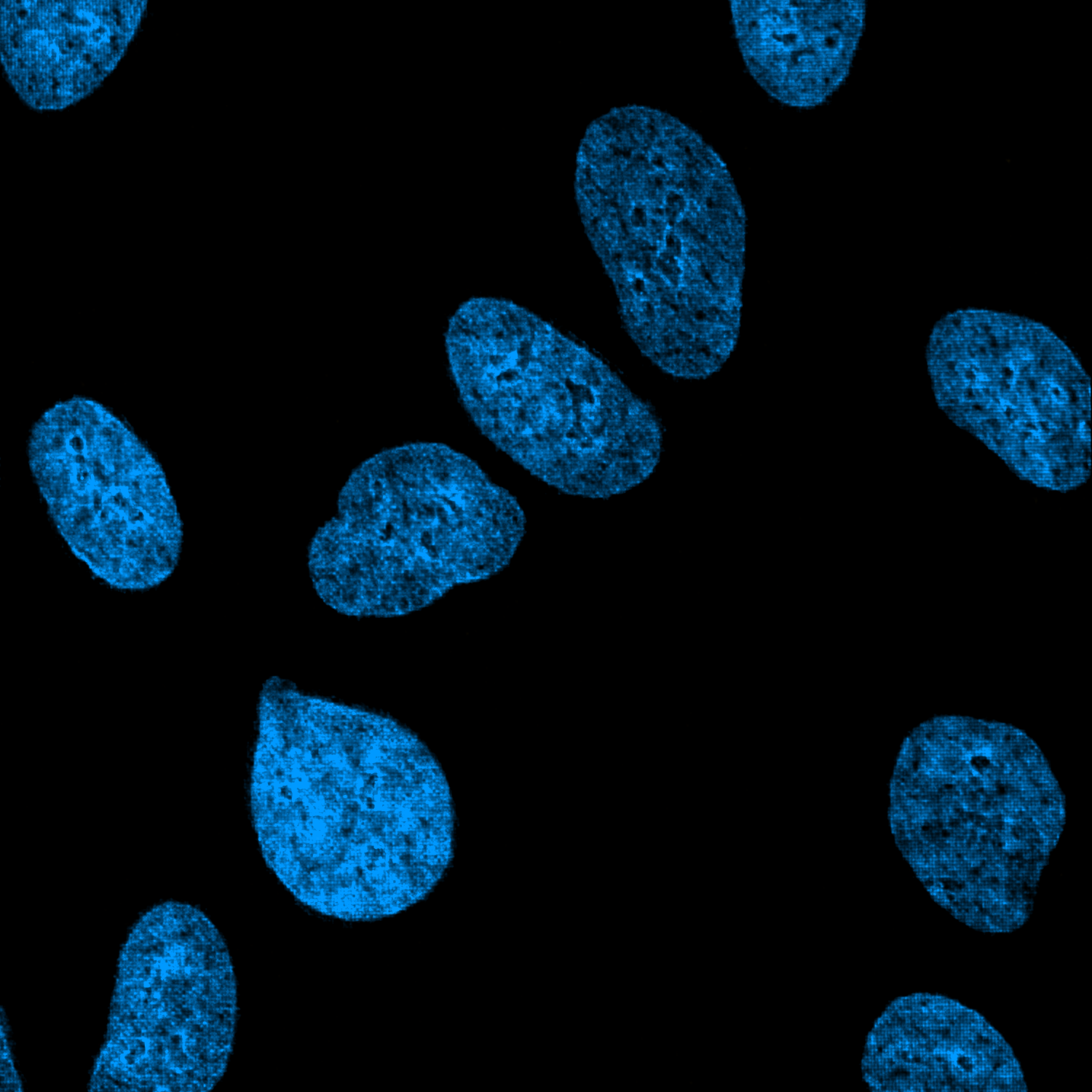

Supplement: Supplementary file 12 — Source data Fig. 6 [file 44318_2024_337_MOESM12_ESM.zip › 06_Figure_06/6H/TP53-gRNA1-POOL+N/TP53-G1-POOL+N_Merge.tif]

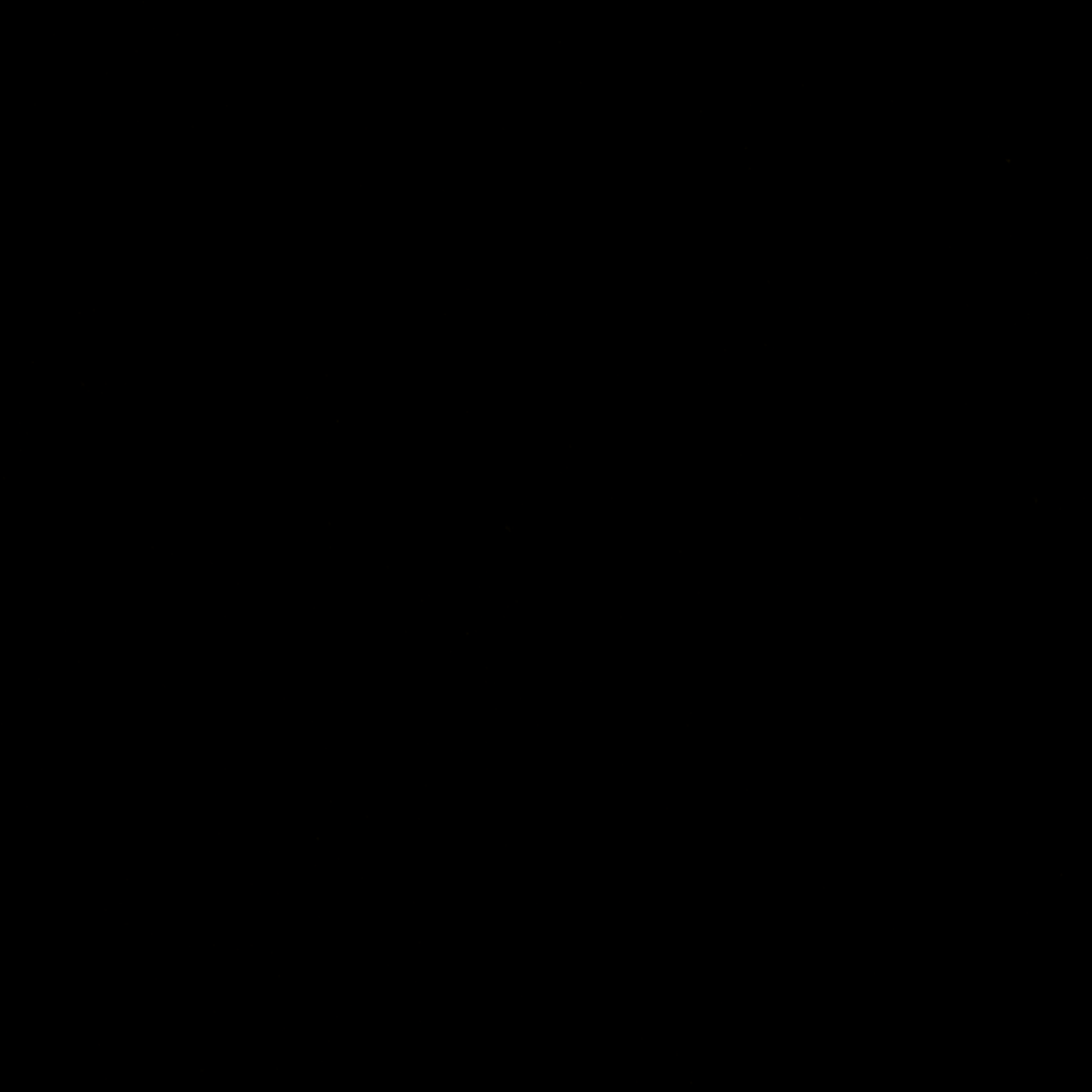

Supplement: Supplementary file 12 — Source data Fig. 6 [file 44318_2024_337_MOESM12_ESM.zip › 06_Figure_06/6H/TP53-gRNA1-POOL+N/TP53-G1-POOL+N_TP53.tif]

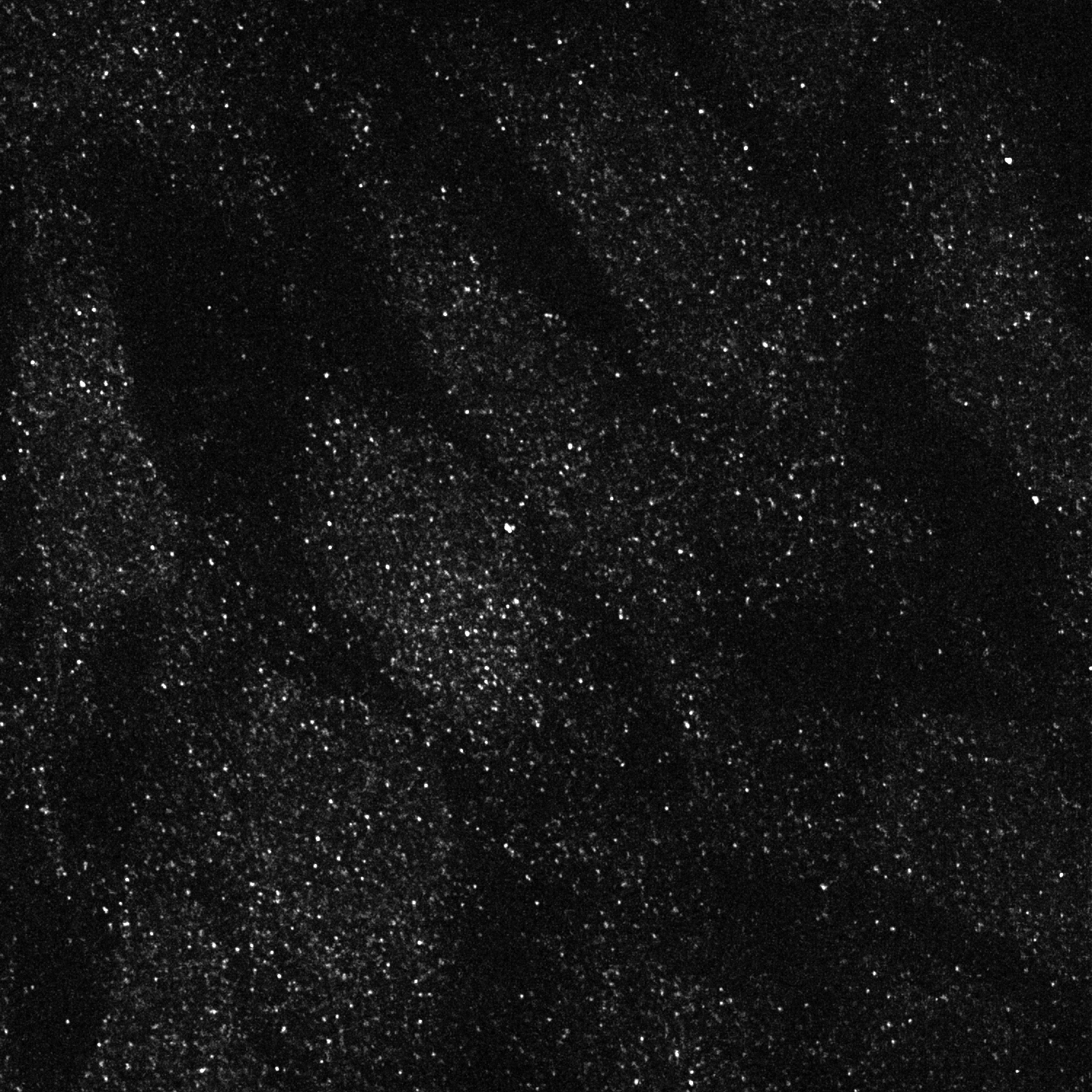

Supplement: Supplementary file 12 — Source data Fig. 6 [file 44318_2024_337_MOESM12_ESM.zip › 06_Figure_06/6H/TP53-gRNA1-POOL+N/_FULL-RANGE-TP53-G1-POOL+N.tif]

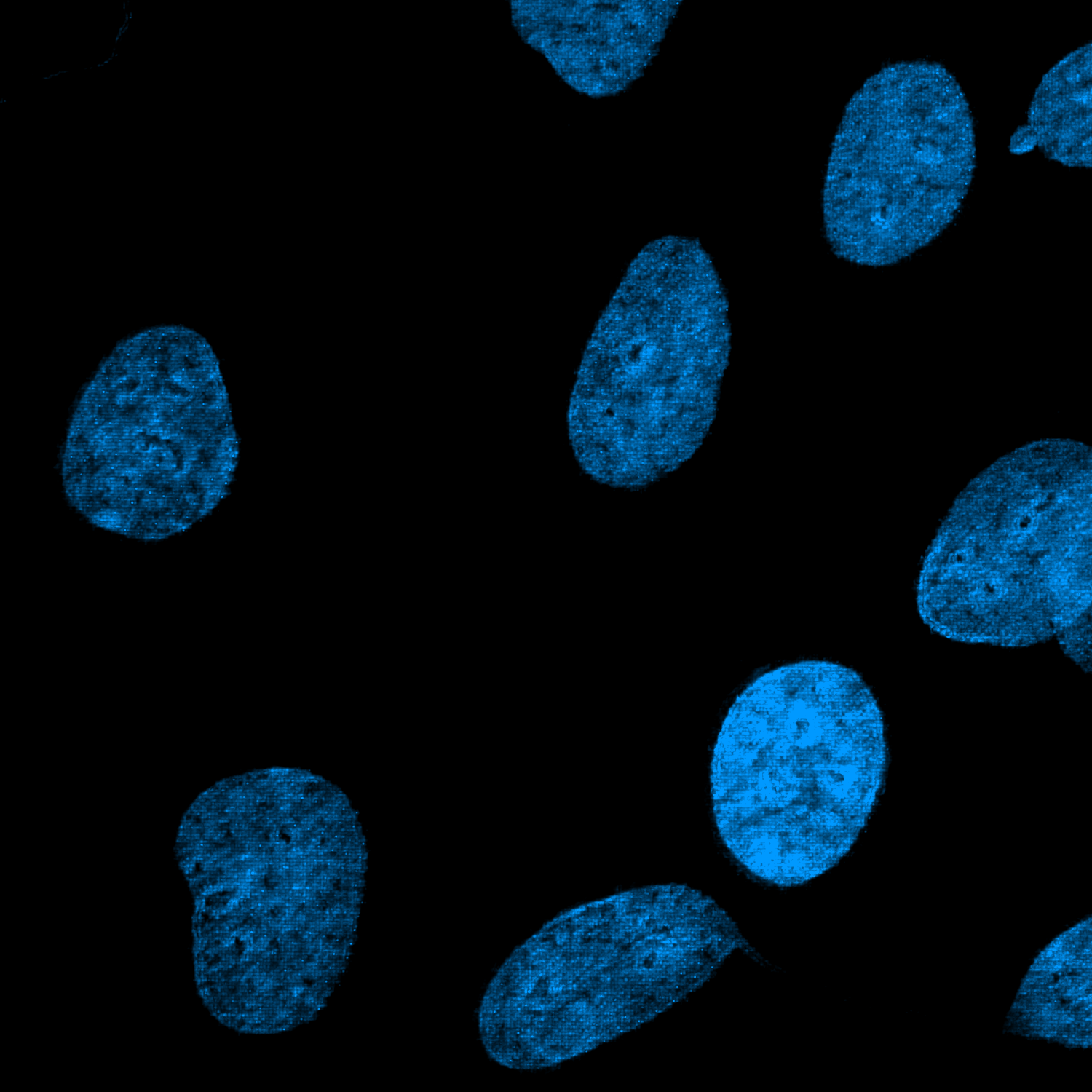

Supplement: Supplementary file 12 — Source data Fig. 6 [file 44318_2024_337_MOESM12_ESM.zip › 06_Figure_06/6H/TP53-gRNA1-POOL-N/TP53-G1-POOL-N_DAPI.tif]

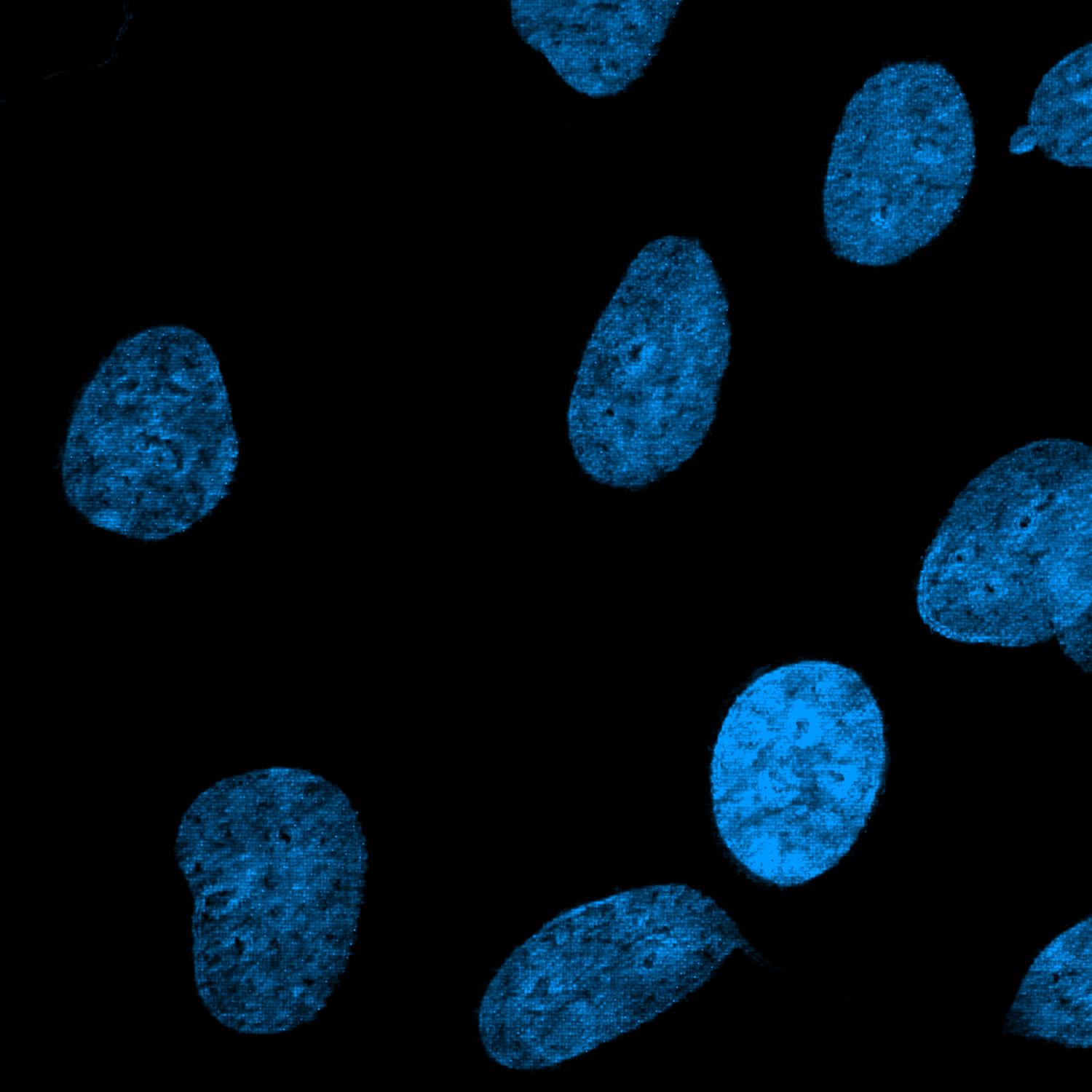

Supplement: Supplementary file 12 — Source data Fig. 6 [file 44318_2024_337_MOESM12_ESM.zip › 06_Figure_06/6H/TP53-gRNA1-POOL-N/TP53-G1-POOL-N_Merge.tif]

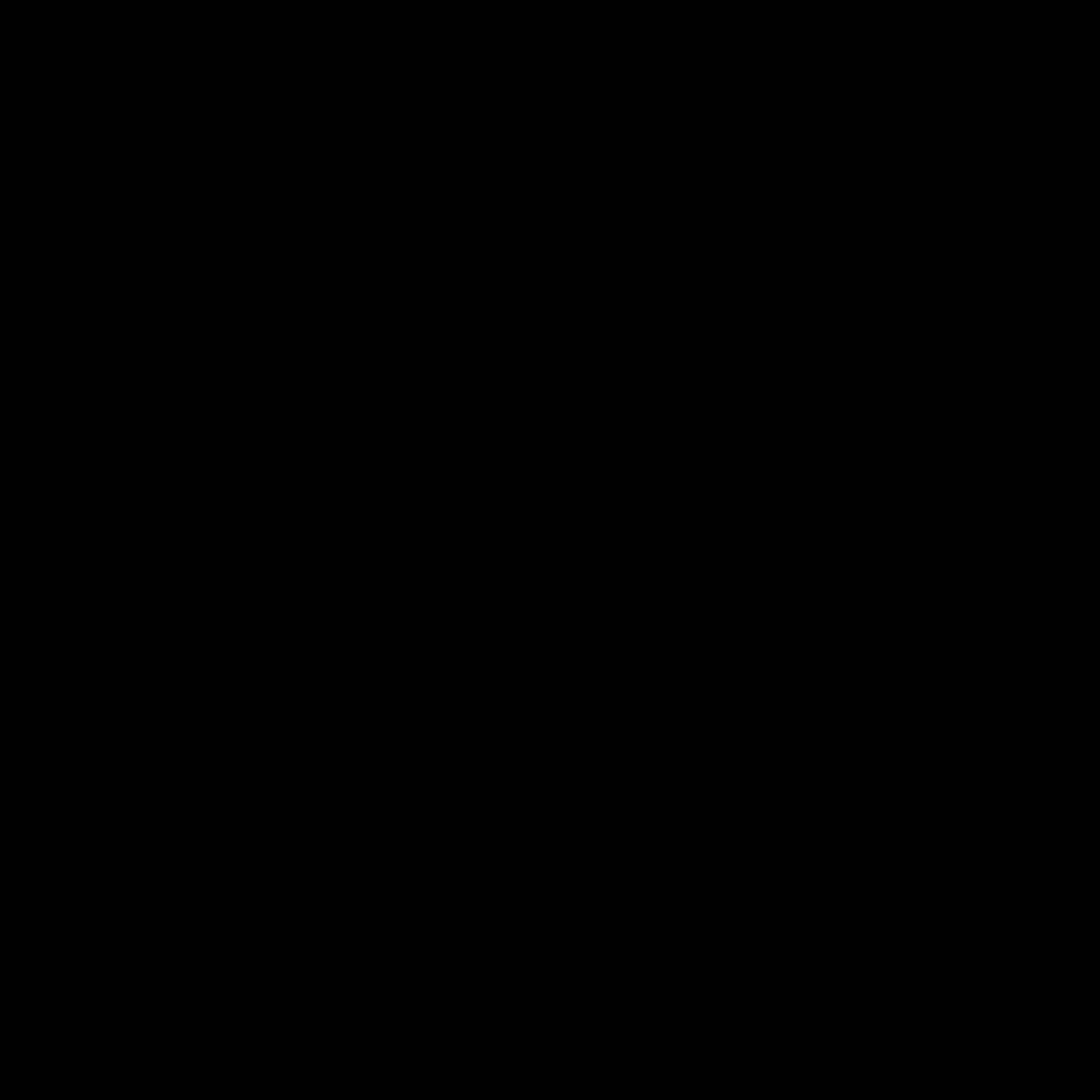

Supplement: Supplementary file 12 — Source data Fig. 6 [file 44318_2024_337_MOESM12_ESM.zip › 06_Figure_06/6H/TP53-gRNA1-POOL-N/TP53-G1-POOL-N_TP53.tif]

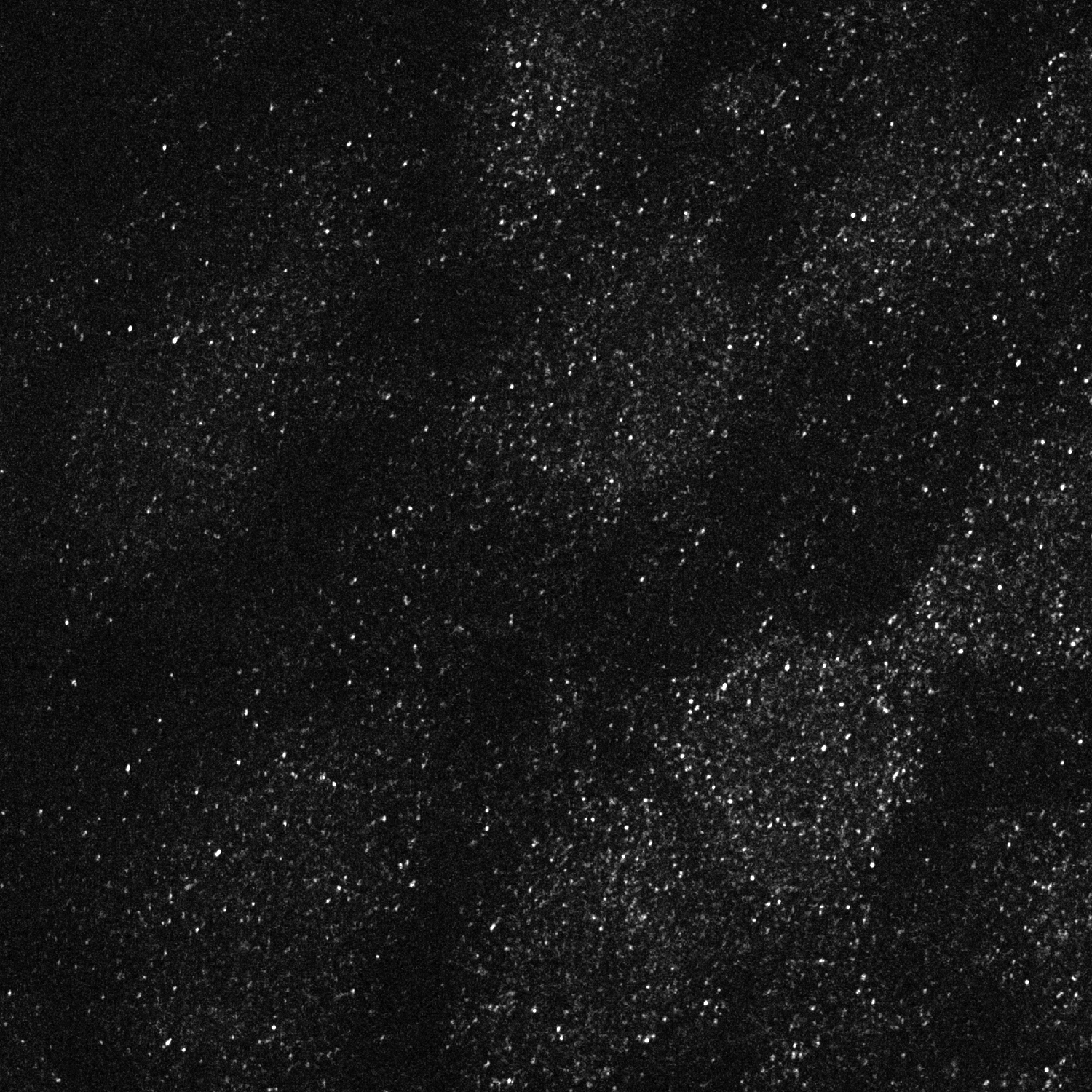

Supplement: Supplementary file 12 — Source data Fig. 6 [file 44318_2024_337_MOESM12_ESM.zip › 06_Figure_06/6H/TP53-gRNA1-POOL-N/_FULL-RANGE-TP53-G1-POOL-N.tif]

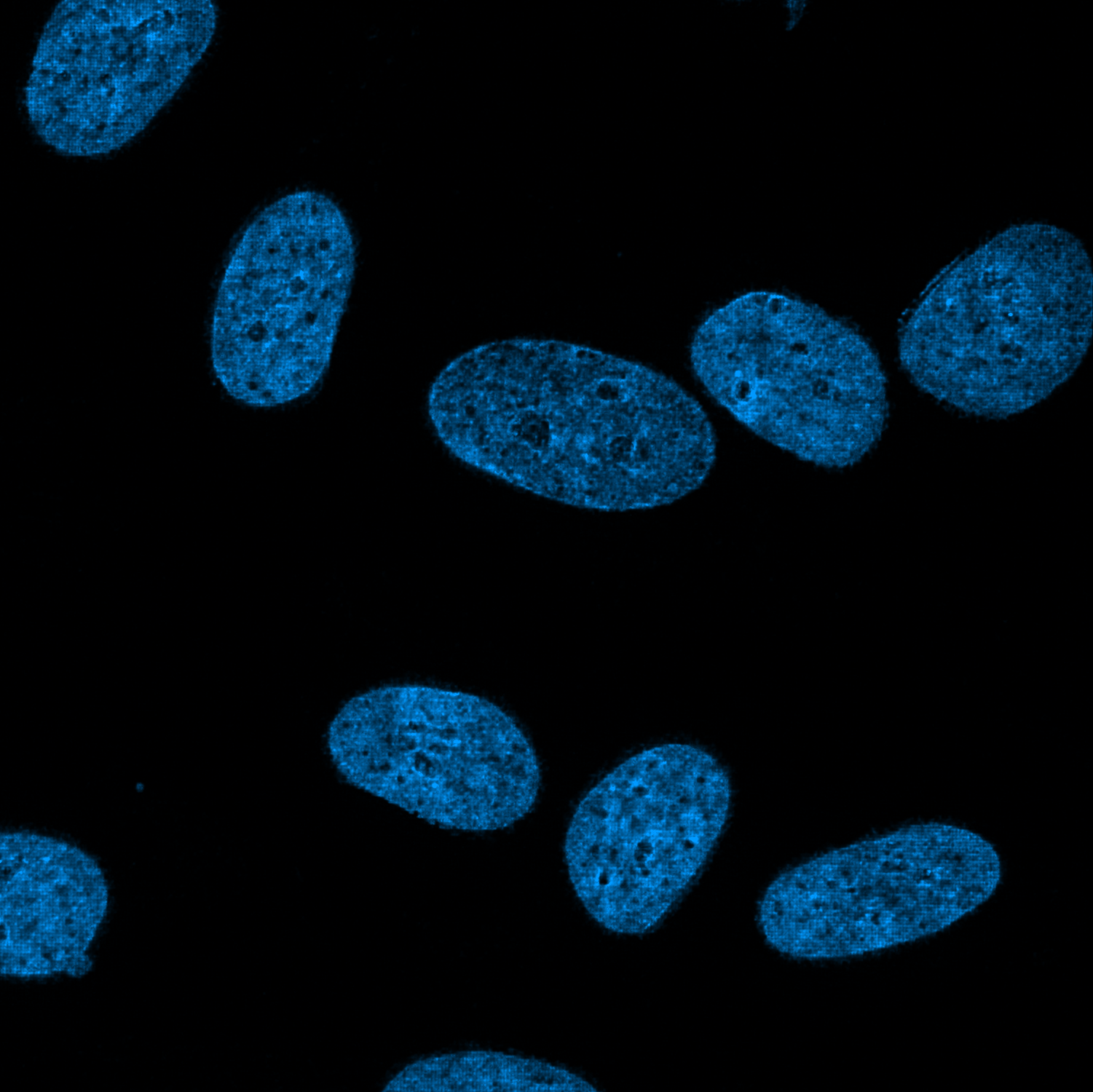

Supplement: Supplementary file 12 — Source data Fig. 6 [file 44318_2024_337_MOESM12_ESM.zip › 06_Figure_06/6H/TP53-gRNA2-CLONE+N/TP53-G2-CLONE+N_DAPI.tif]

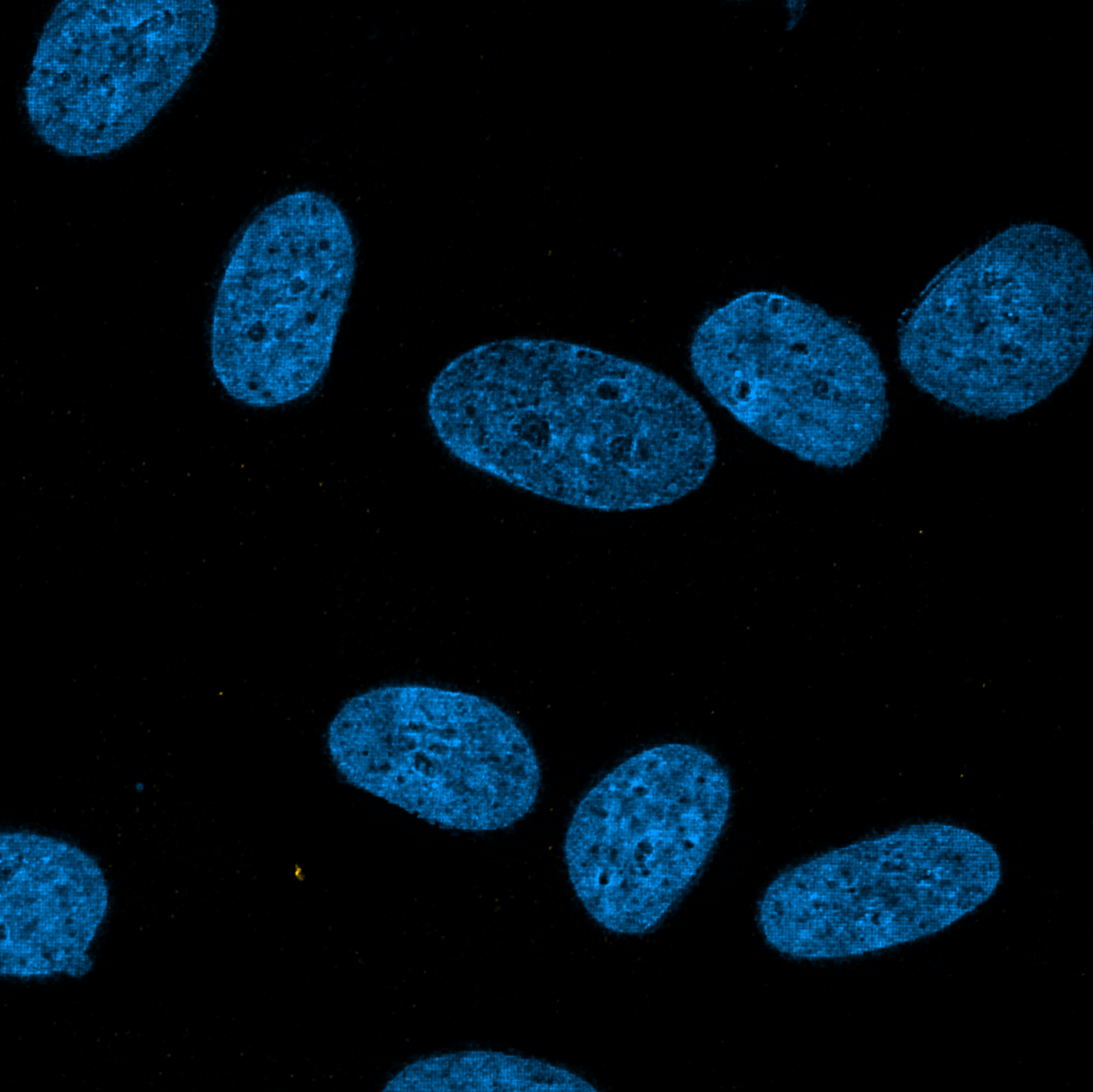

Supplement: Supplementary file 12 — Source data Fig. 6 [file 44318_2024_337_MOESM12_ESM.zip › 06_Figure_06/6H/TP53-gRNA2-CLONE+N/TP53-G2-CLONE+N_Merge.tif]

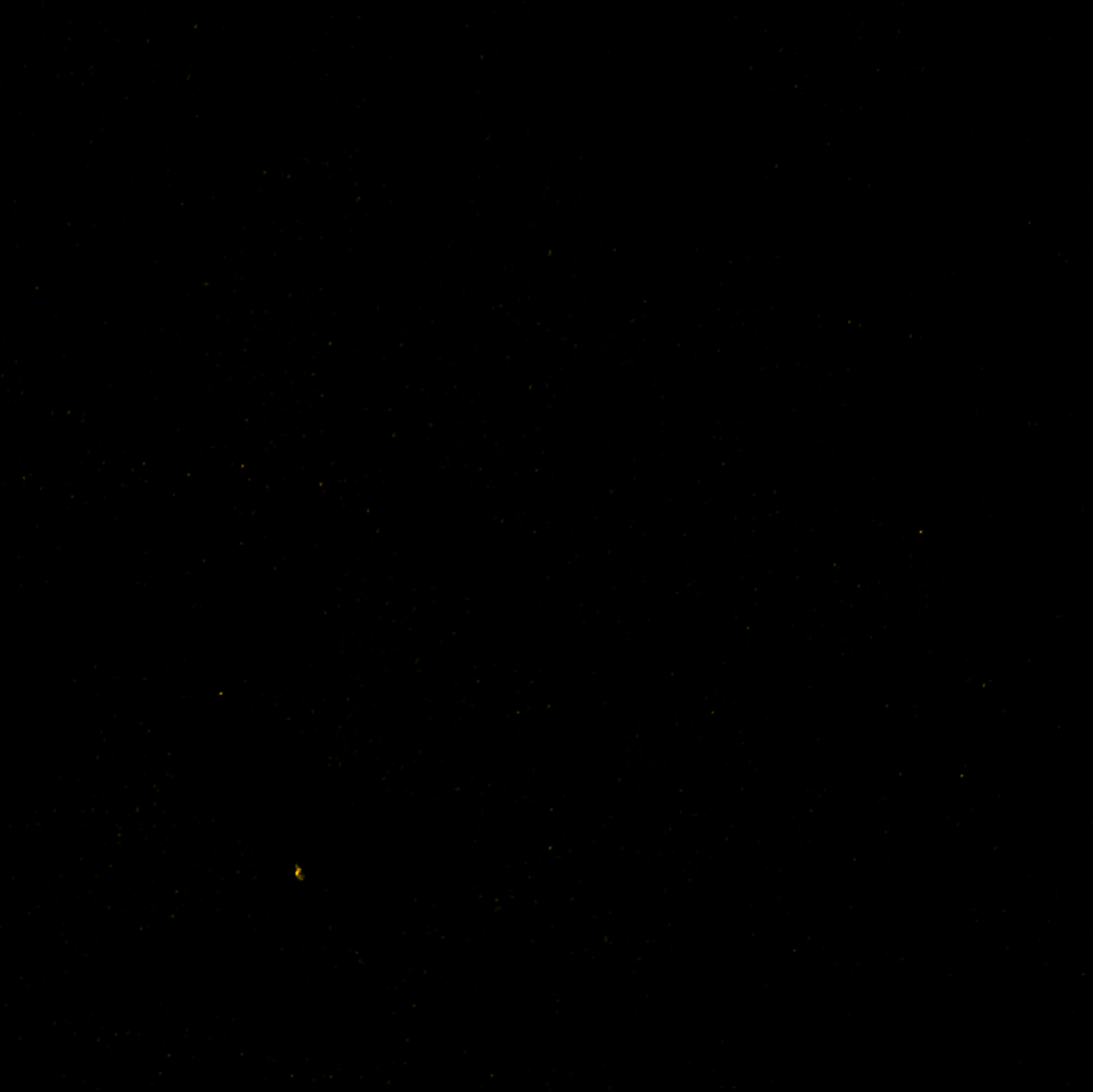

Supplement: Supplementary file 12 — Source data Fig. 6 [file 44318_2024_337_MOESM12_ESM.zip › 06_Figure_06/6H/TP53-gRNA2-CLONE+N/TP53-G2-CLONE+N_TP53.tif]

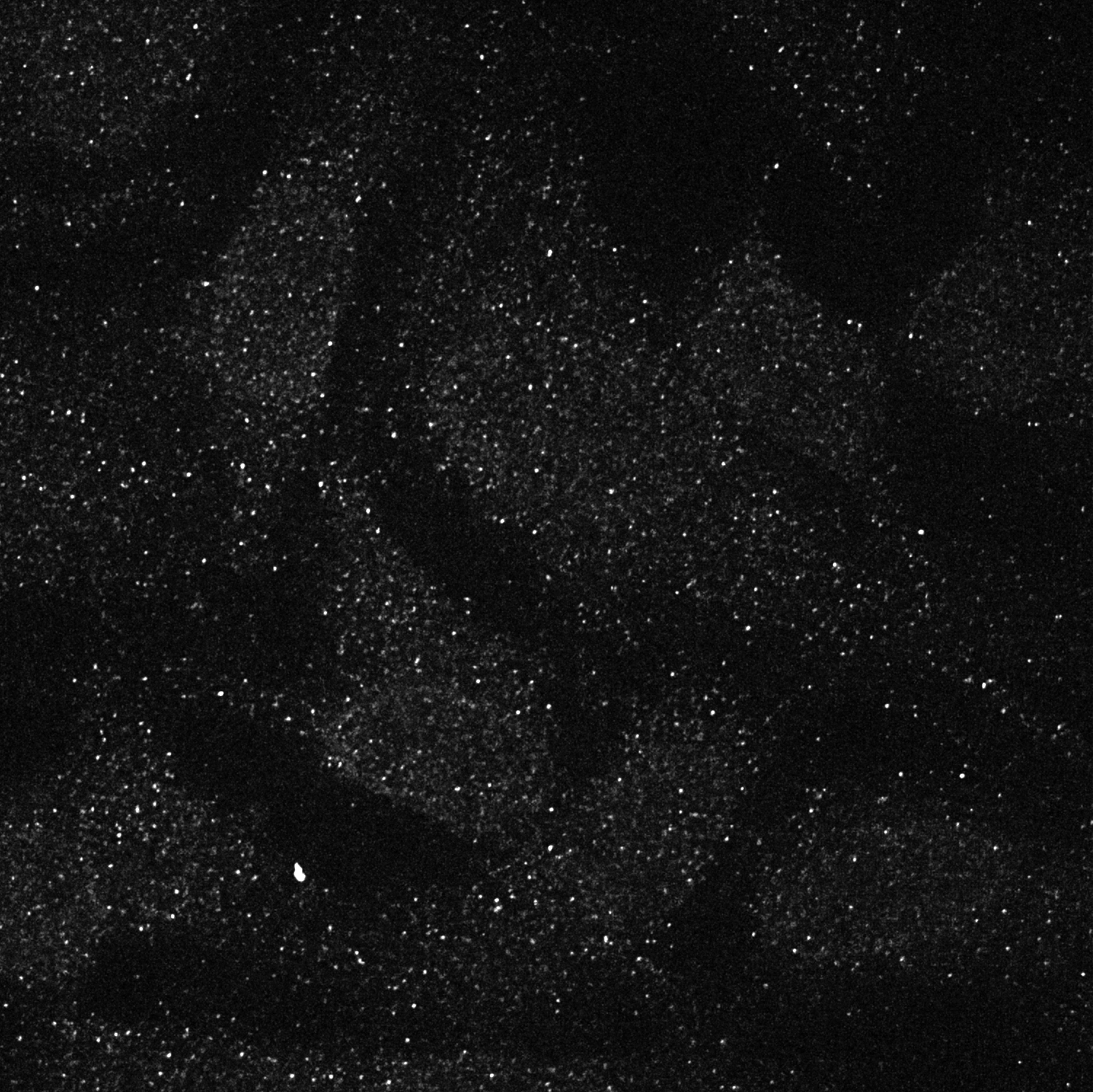

Supplement: Supplementary file 12 — Source data Fig. 6 [file 44318_2024_337_MOESM12_ESM.zip › 06_Figure_06/6H/TP53-gRNA2-CLONE+N/_FULL-RANGE-TP53-G2-CLONE+N.tif]

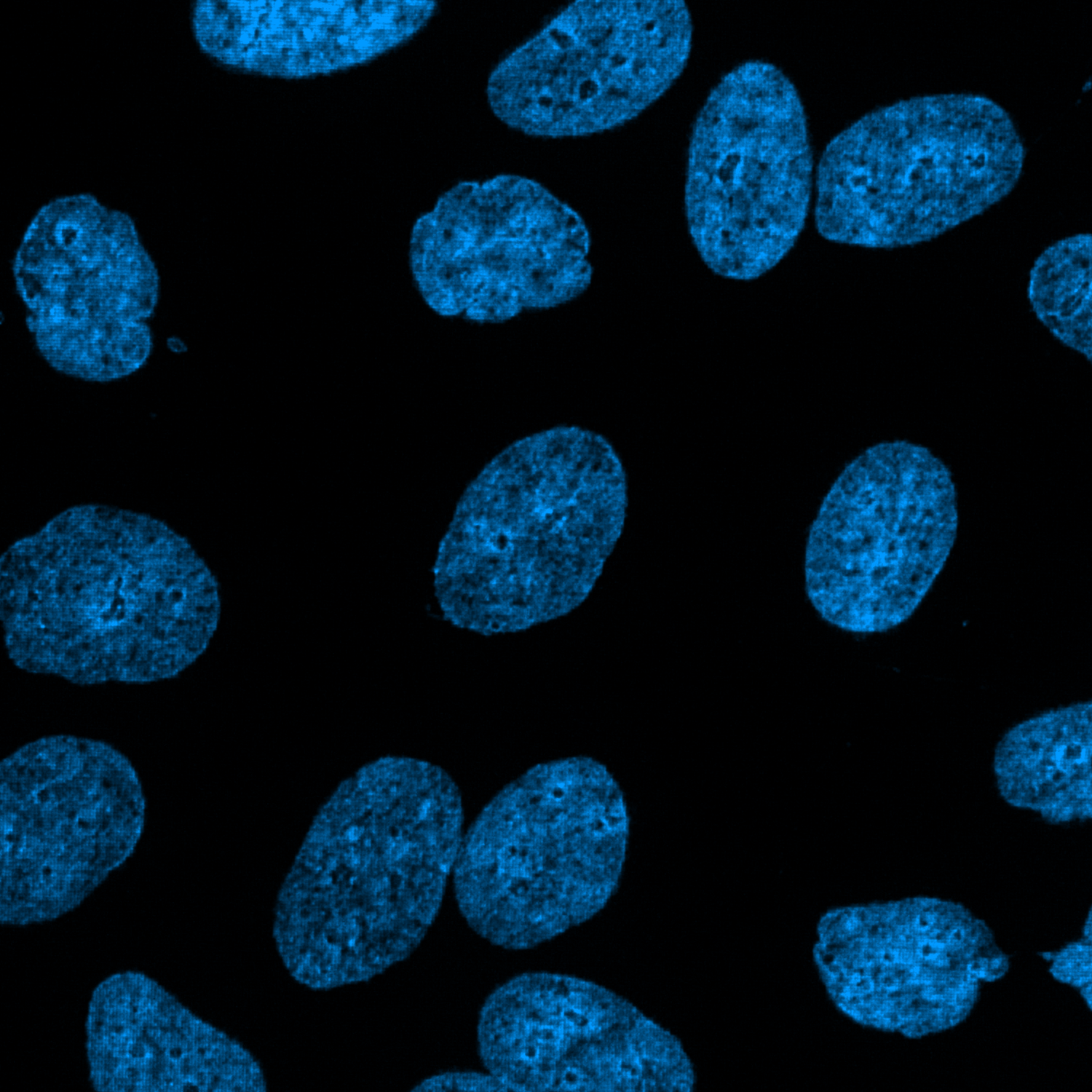

Supplement: Supplementary file 12 — Source data Fig. 6 [file 44318_2024_337_MOESM12_ESM.zip › 06_Figure_06/6H/TP53-gRNA2-CLONE-N/TP53-G2-CLONE-N_DAPI.tif]

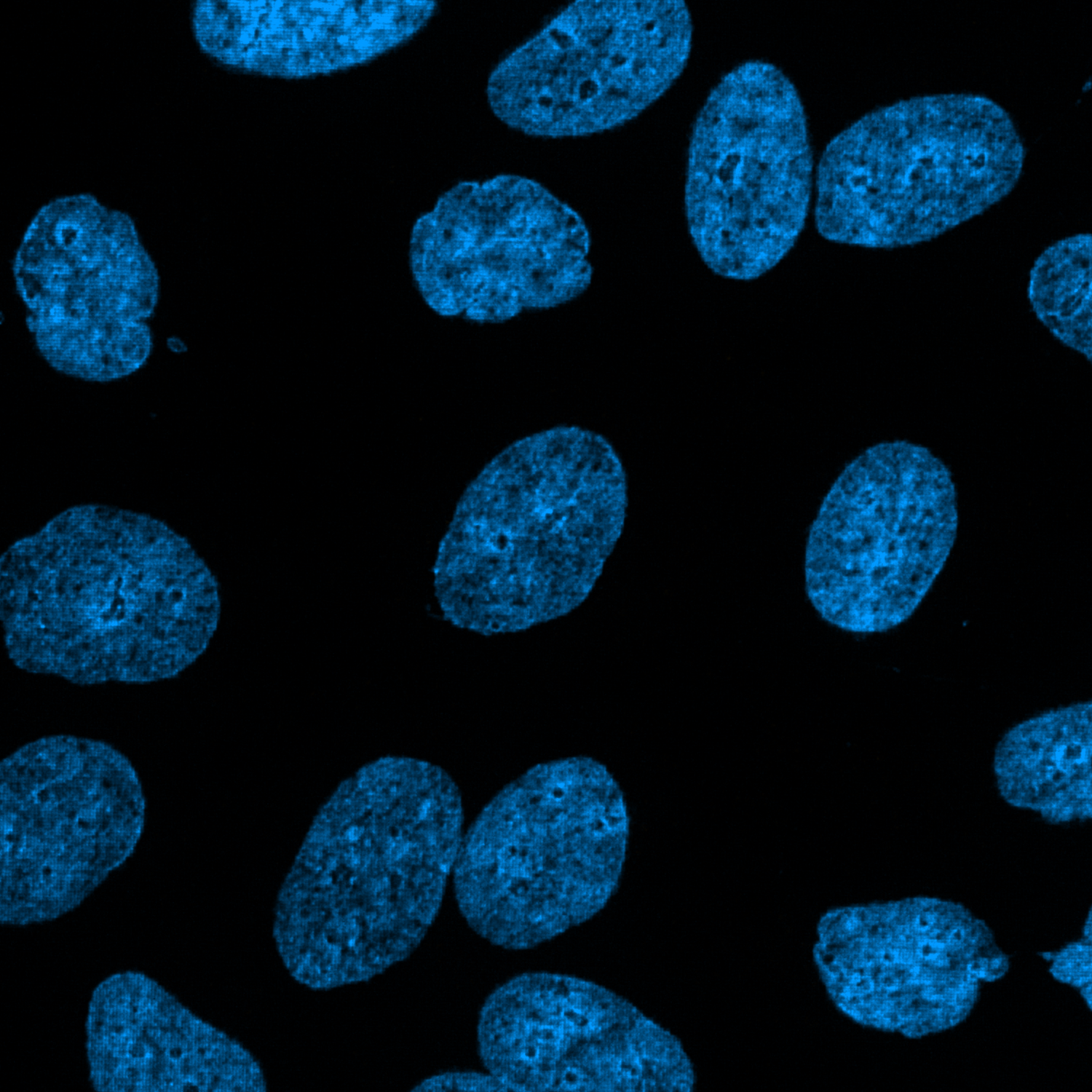

Supplement: Supplementary file 12 — Source data Fig. 6 [file 44318_2024_337_MOESM12_ESM.zip › 06_Figure_06/6H/TP53-gRNA2-CLONE-N/TP53-G2-CLONE-N_Merge.tif]

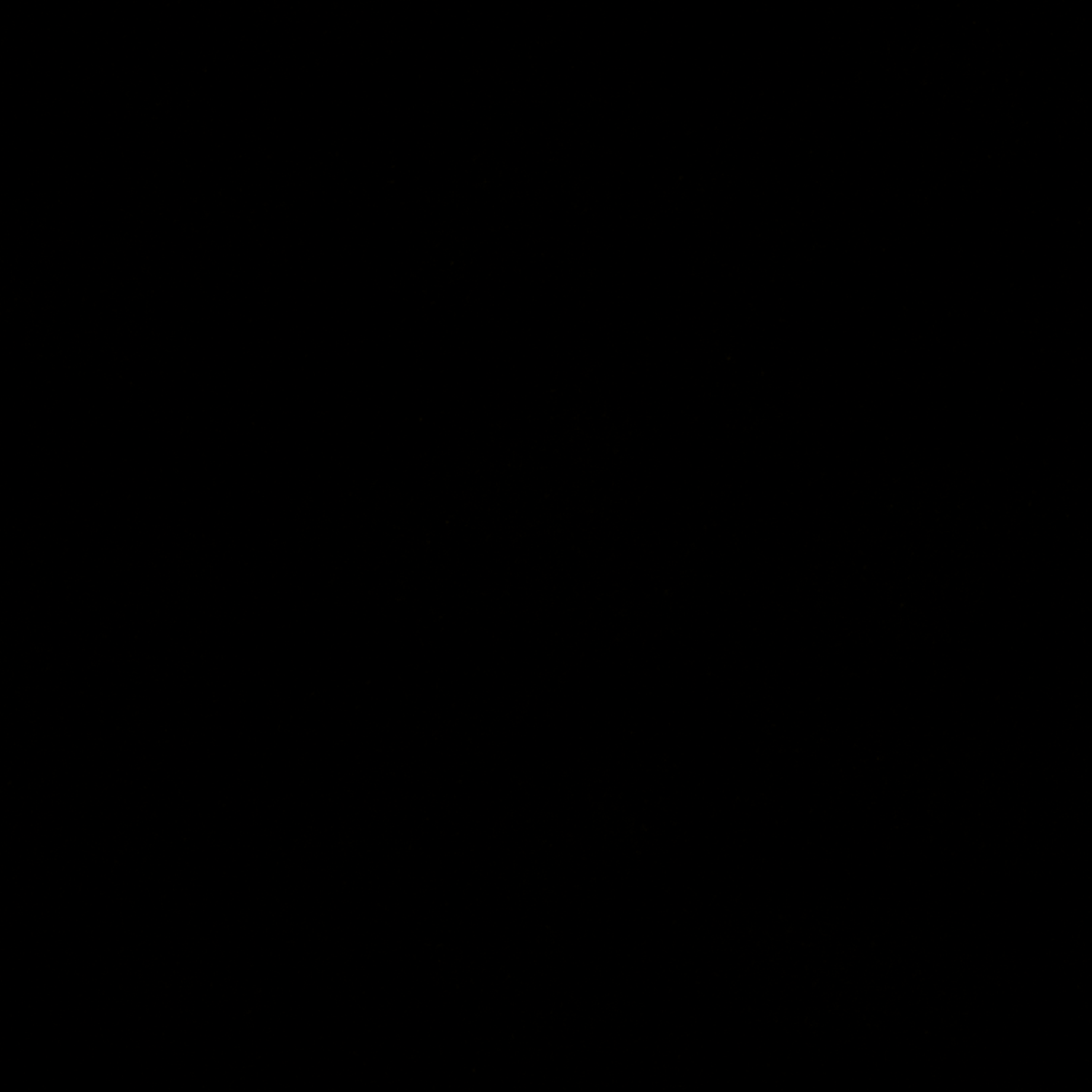

Supplement: Supplementary file 12 — Source data Fig. 6 [file 44318_2024_337_MOESM12_ESM.zip › 06_Figure_06/6H/TP53-gRNA2-CLONE-N/TP53-G2-CLONE-N_TP53.tif]

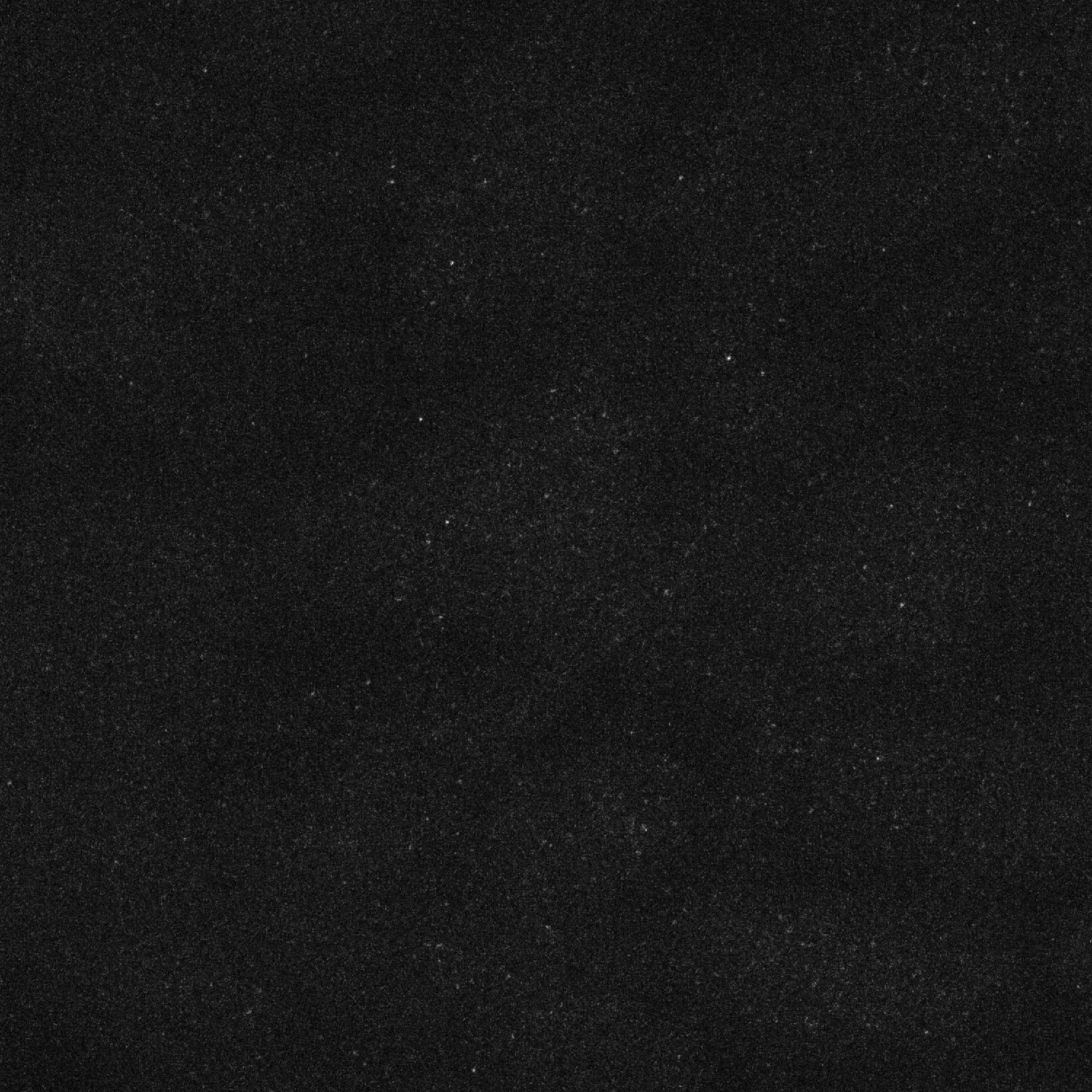

Supplement: Supplementary file 12 — Source data Fig. 6 [file 44318_2024_337_MOESM12_ESM.zip › 06_Figure_06/6H/TP53-gRNA2-CLONE-N/_FULL-RANGE-TP53-G2-CLONE-N.tif]

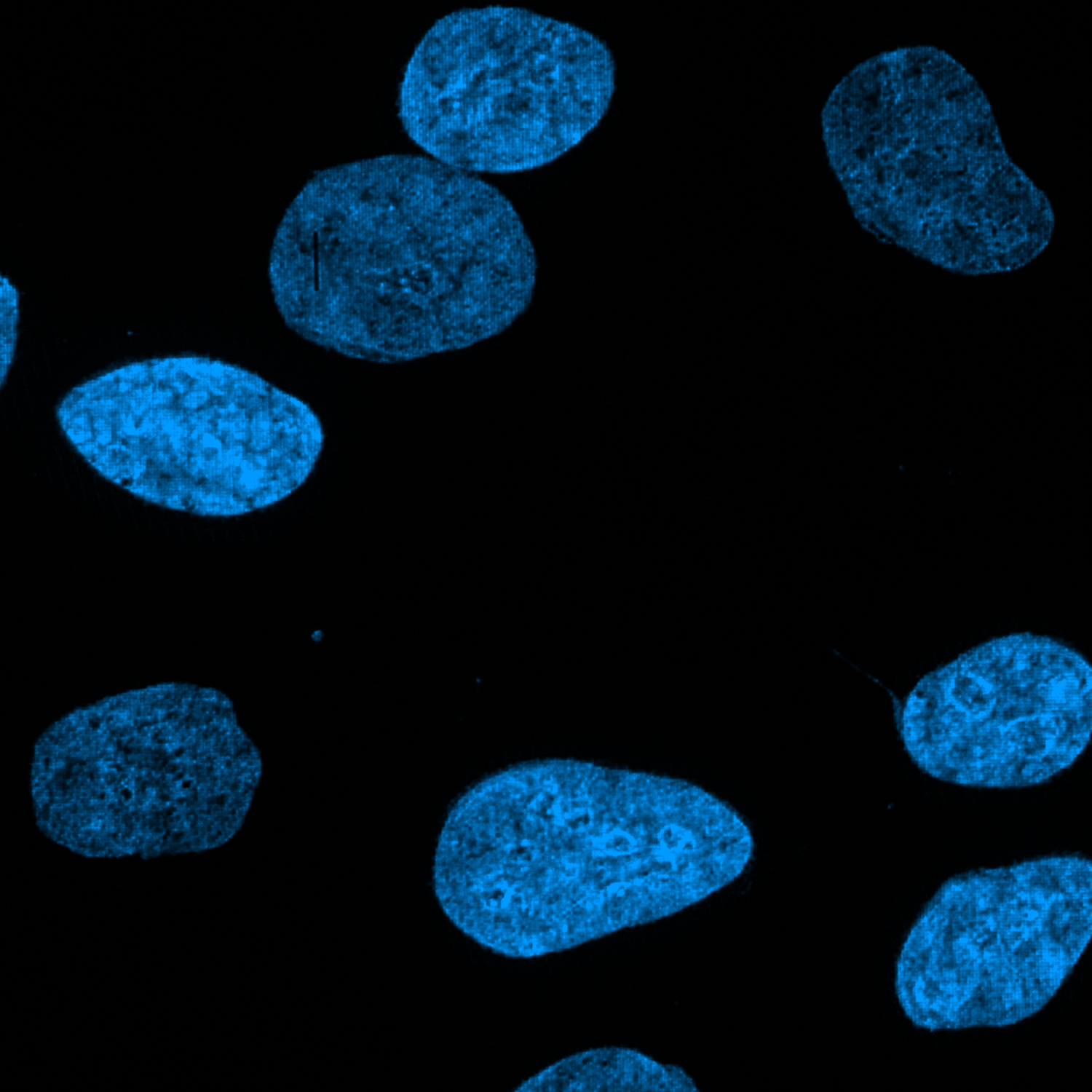

Supplement: Supplementary file 12 — Source data Fig. 6 [file 44318_2024_337_MOESM12_ESM.zip › 06_Figure_06/6H/TP53-gRNA2-POOL+N/TP53-G2-POOL+N_DAPI.tif]

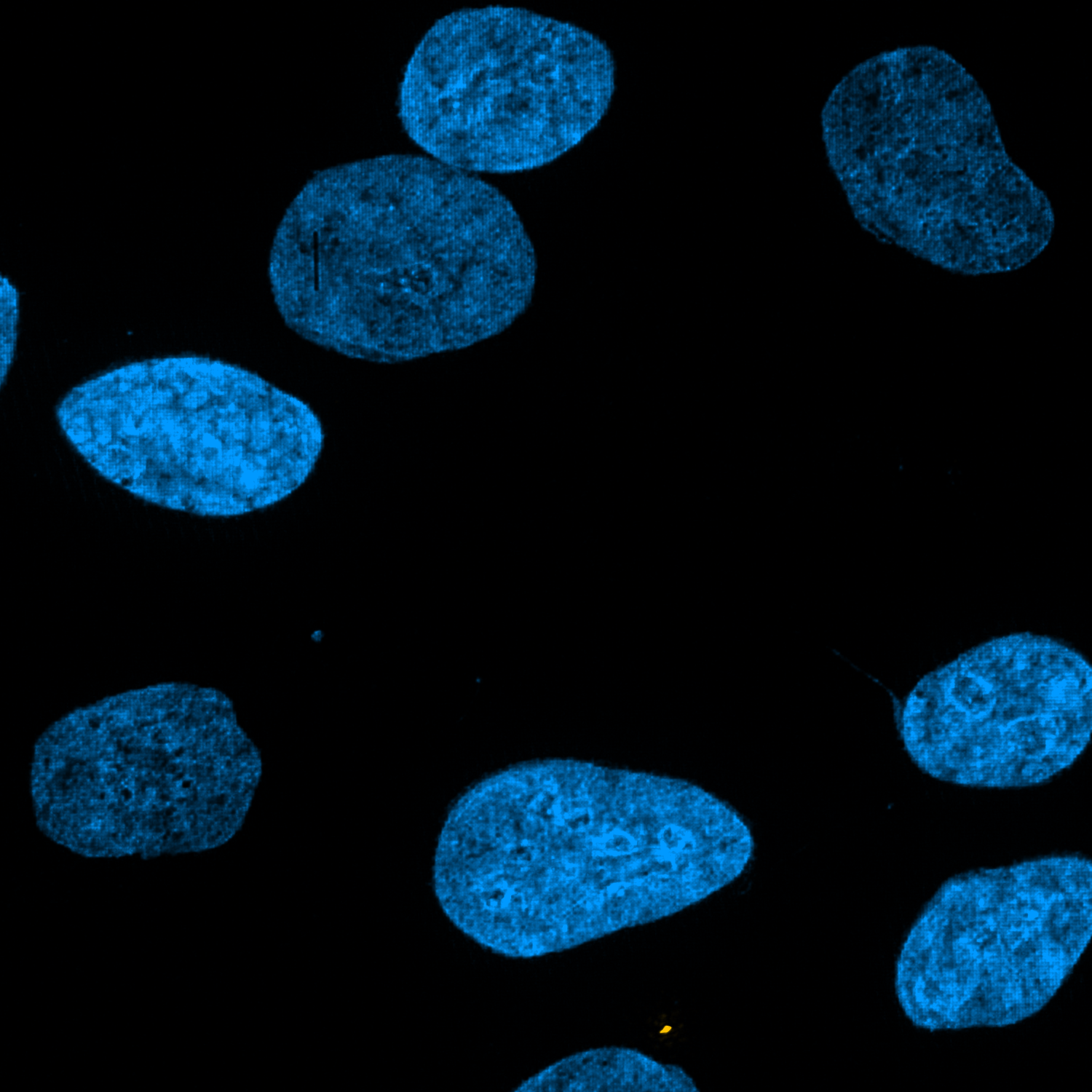

Supplement: Supplementary file 12 — Source data Fig. 6 [file 44318_2024_337_MOESM12_ESM.zip › 06_Figure_06/6H/TP53-gRNA2-POOL+N/TP53-G2-POOL+N_Merge.tif]

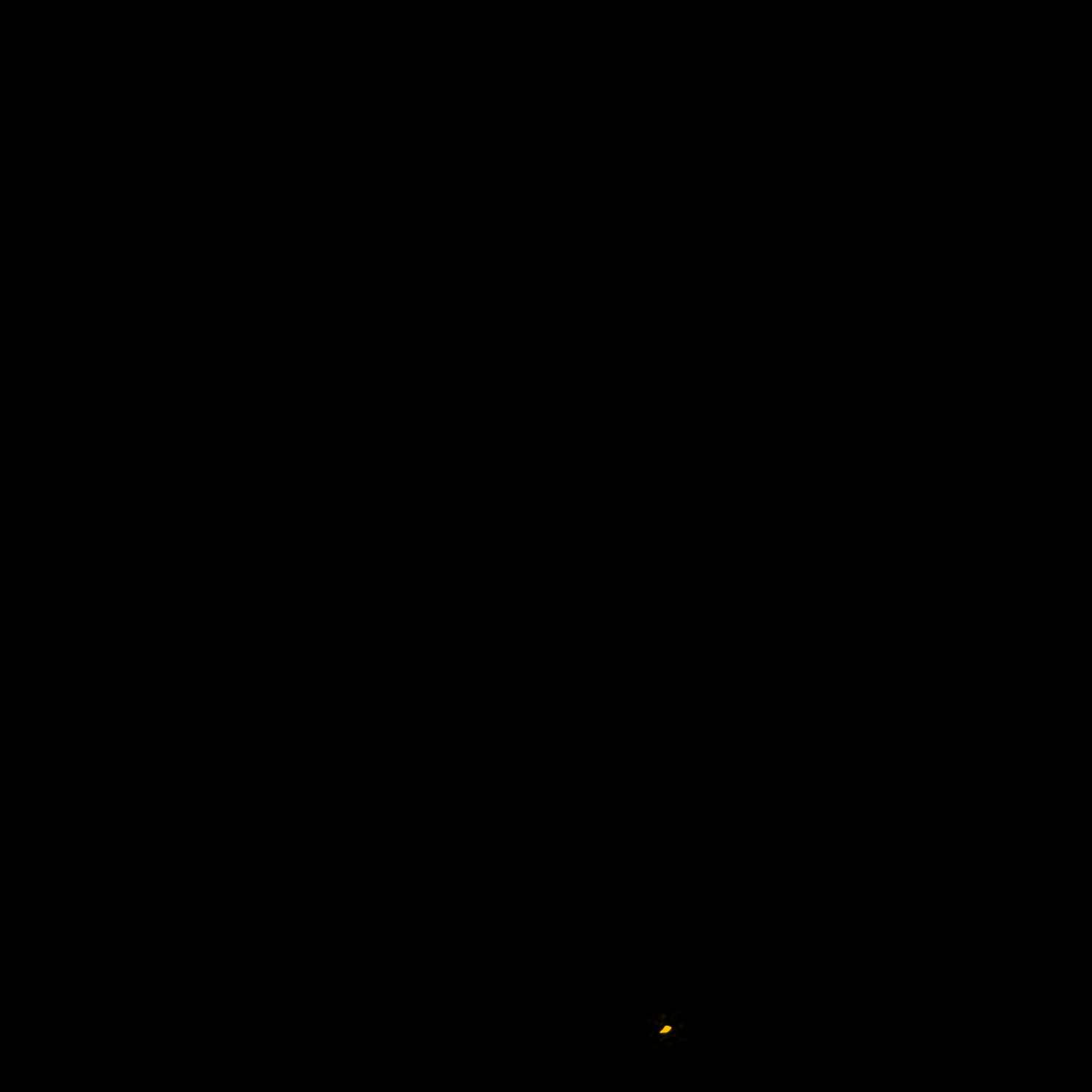

Supplement: Supplementary file 12 — Source data Fig. 6 [file 44318_2024_337_MOESM12_ESM.zip › 06_Figure_06/6H/TP53-gRNA2-POOL+N/TP53-G2-POOL+N_TP53.tif]

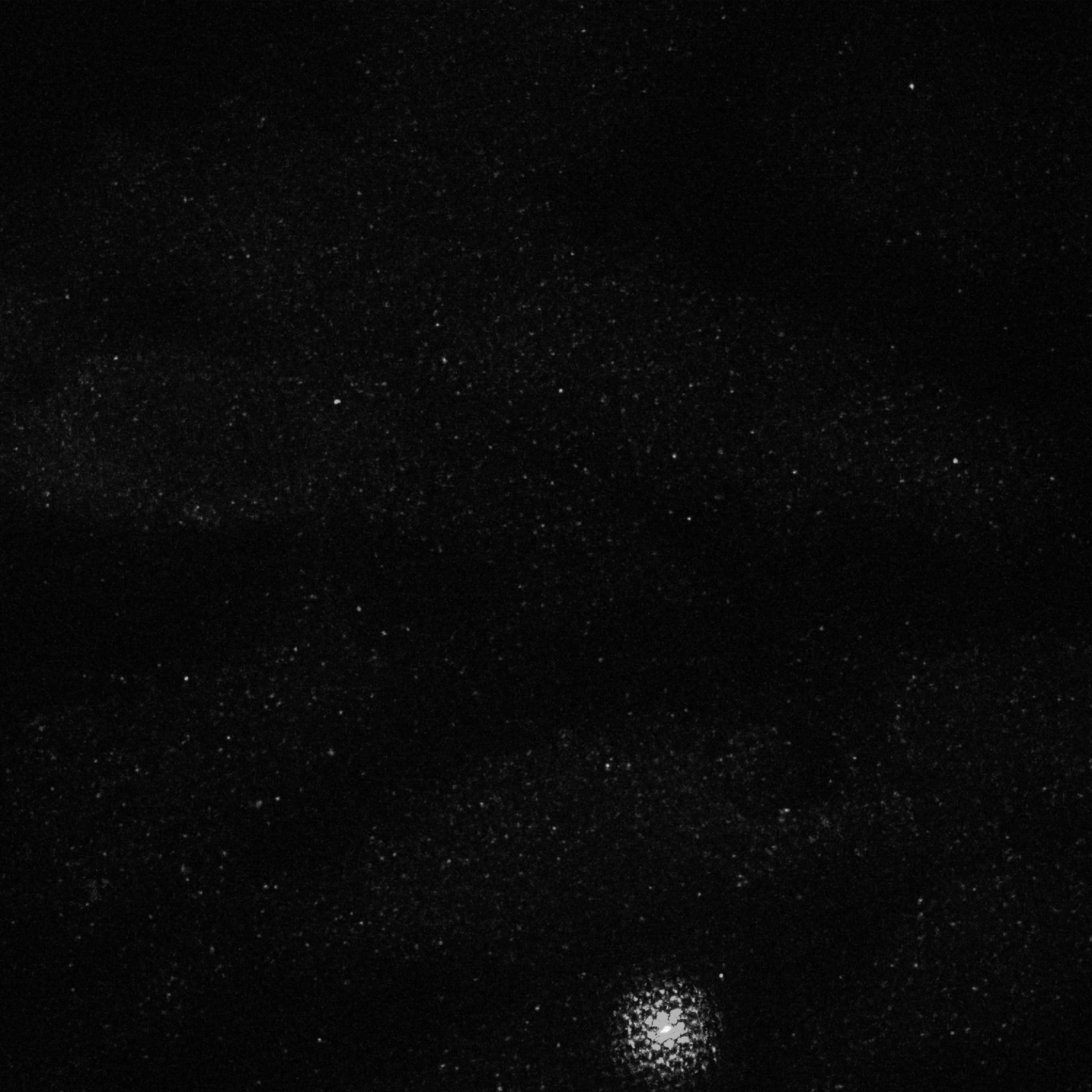

Supplement: Supplementary file 12 — Source data Fig. 6 [file 44318_2024_337_MOESM12_ESM.zip › 06_Figure_06/6H/TP53-gRNA2-POOL+N/_FULL-RANGE-TP53-G2-POOL+N.tif]

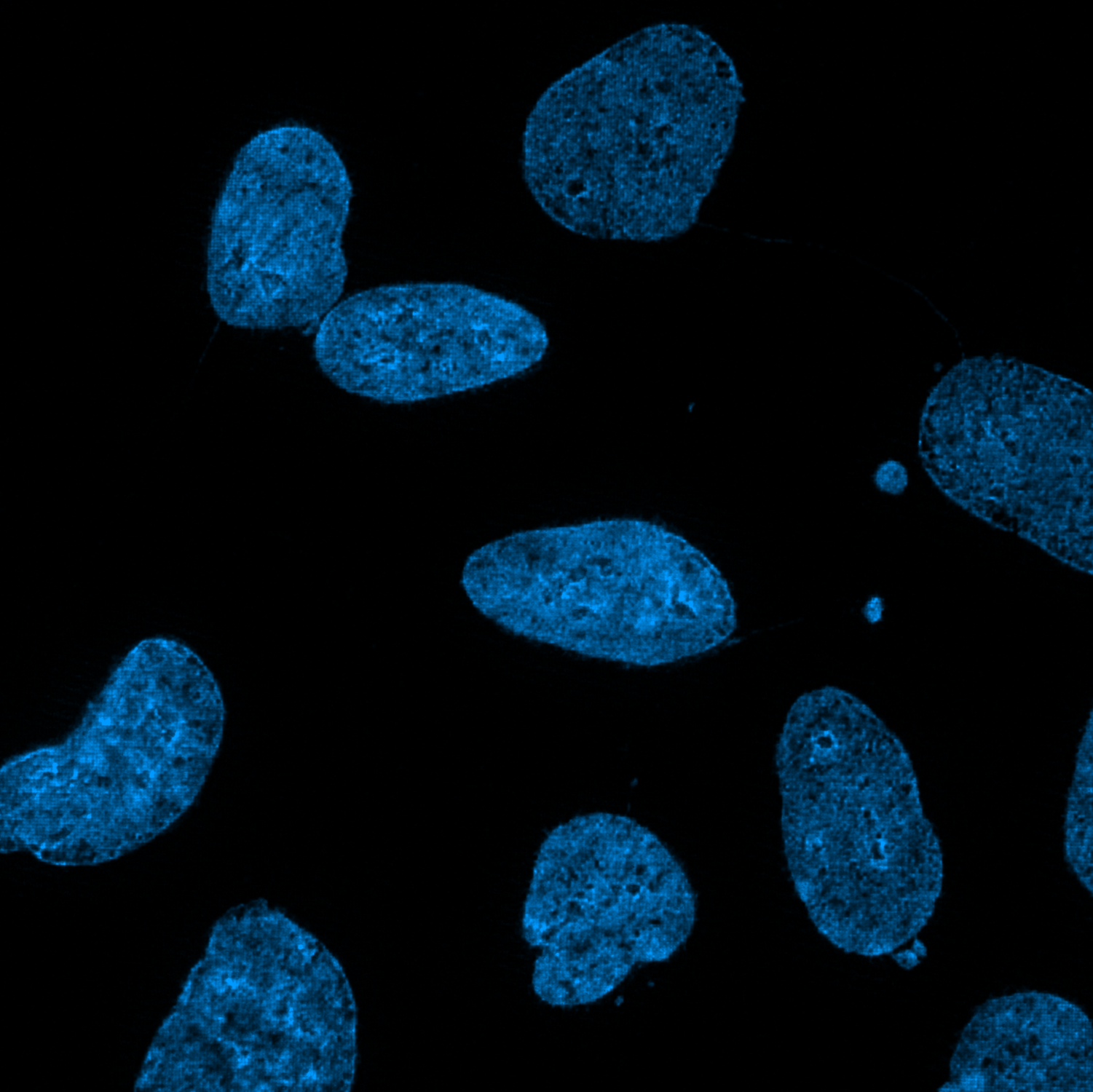

Supplement: Supplementary file 12 — Source data Fig. 6 [file 44318_2024_337_MOESM12_ESM.zip › 06_Figure_06/6H/TP53-gRNA2-POOL-N/TP53-G2-POOL-N_DAPI.tif]

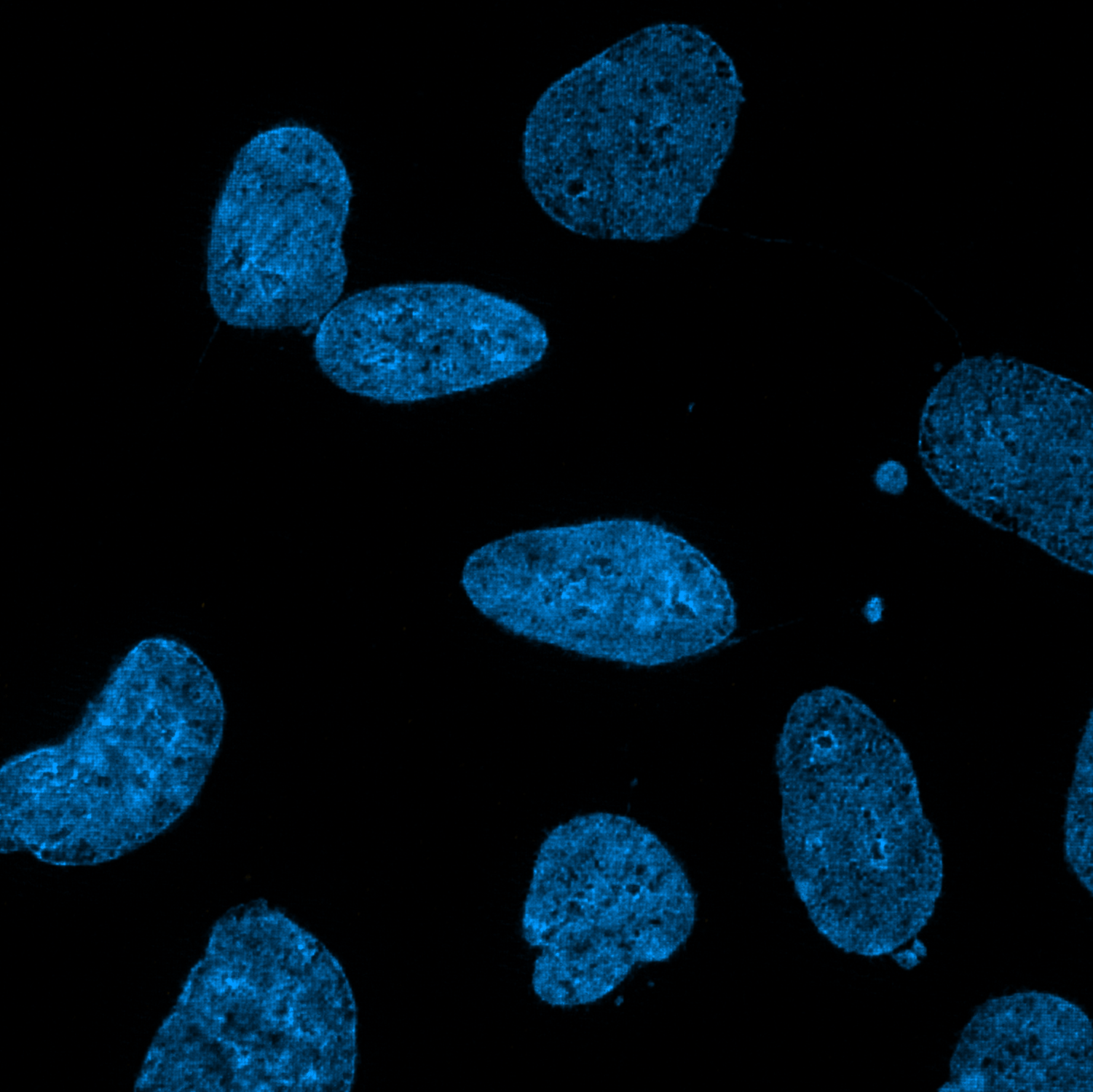

Supplement: Supplementary file 12 — Source data Fig. 6 [file 44318_2024_337_MOESM12_ESM.zip › 06_Figure_06/6H/TP53-gRNA2-POOL-N/TP53-G2-POOL-N_Merge.tif]

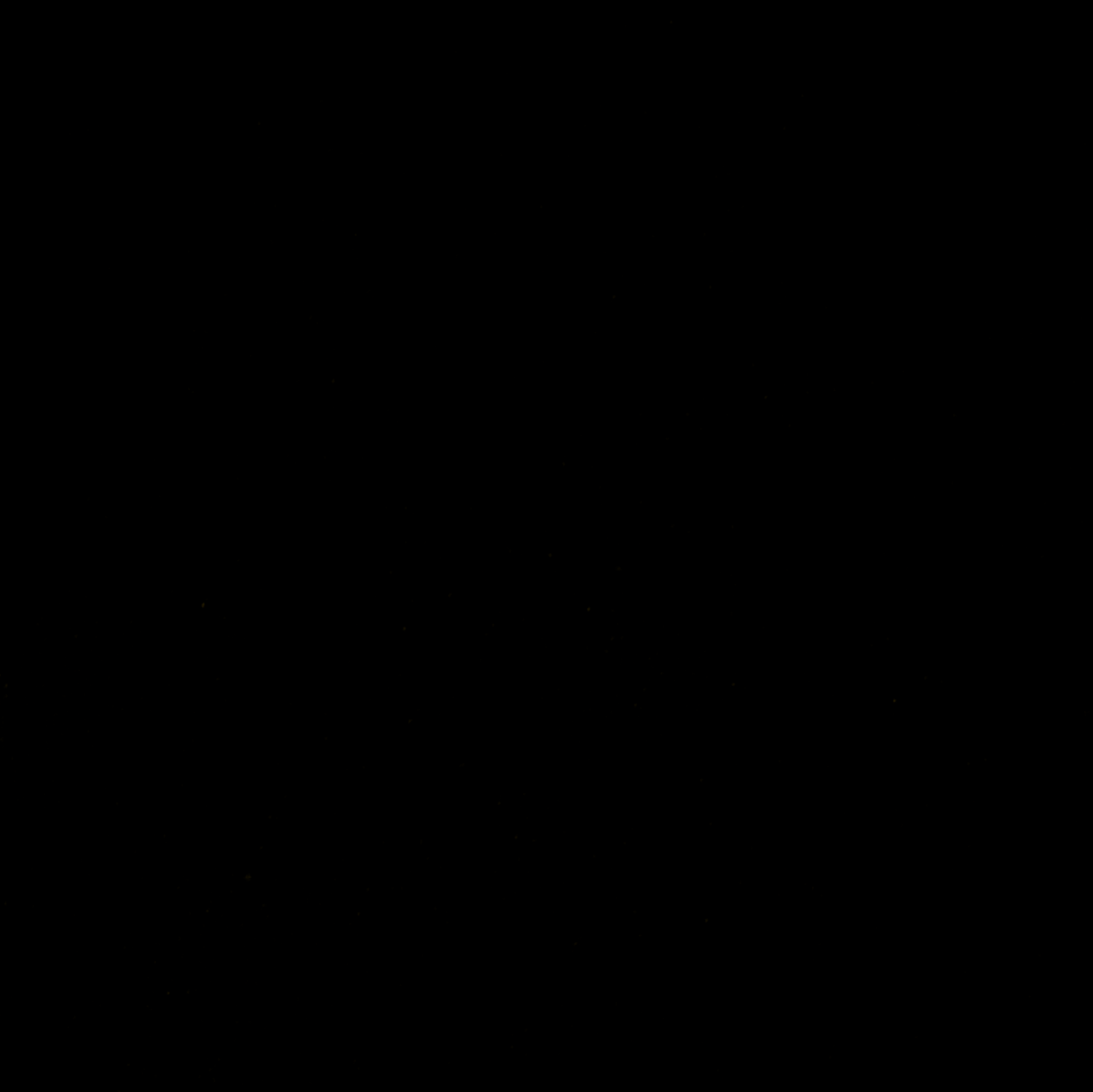

Supplement: Supplementary file 12 — Source data Fig. 6 [file 44318_2024_337_MOESM12_ESM.zip › 06_Figure_06/6H/TP53-gRNA2-POOL-N/TP53-G2-POOL-N_TP53.tif]

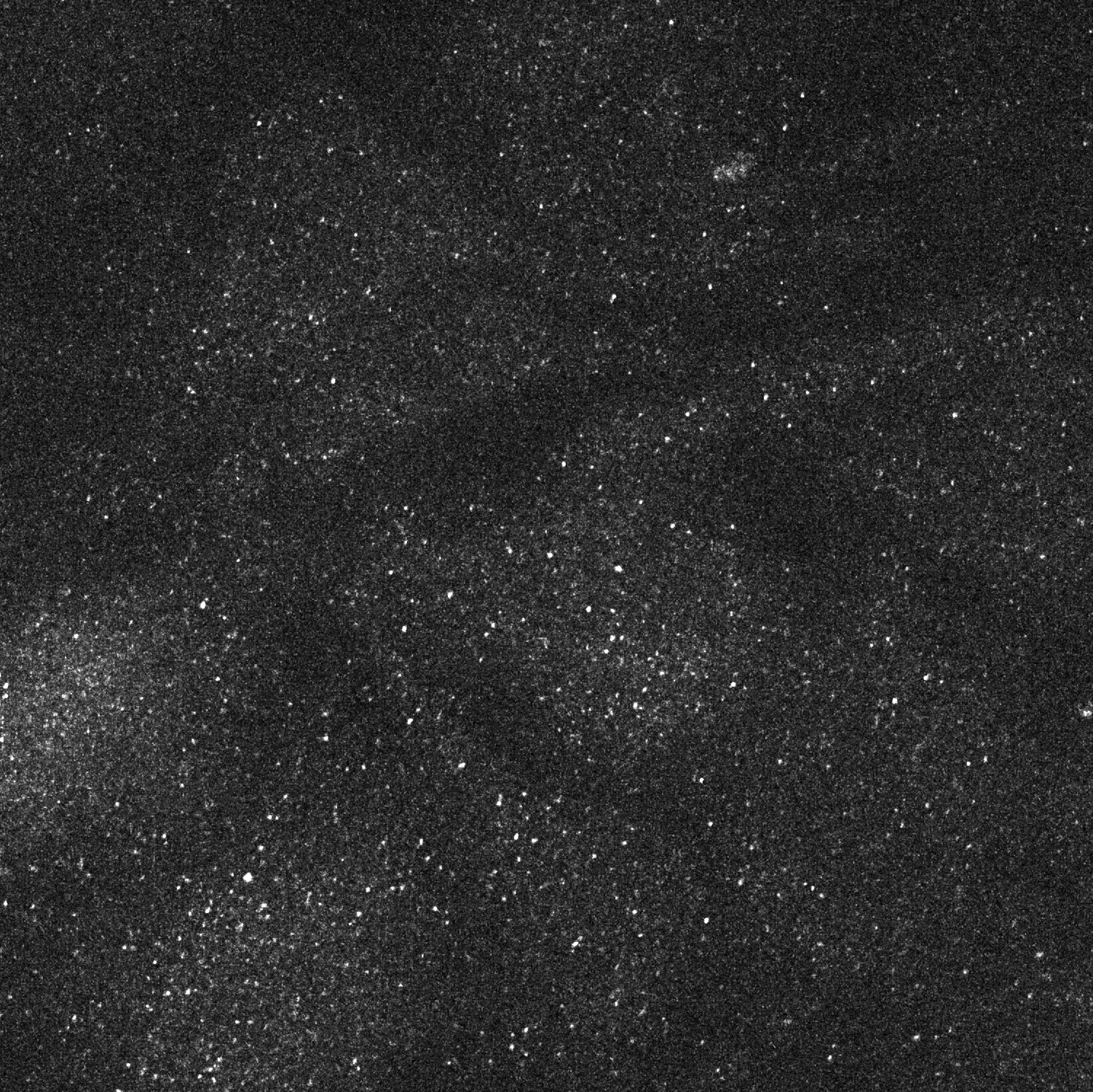

Supplement: Supplementary file 12 — Source data Fig. 6 [file 44318_2024_337_MOESM12_ESM.zip › 06_Figure_06/6H/TP53-gRNA2-POOL-N/_FULL-RANGE-TP53-G2-POOL-N.tif]

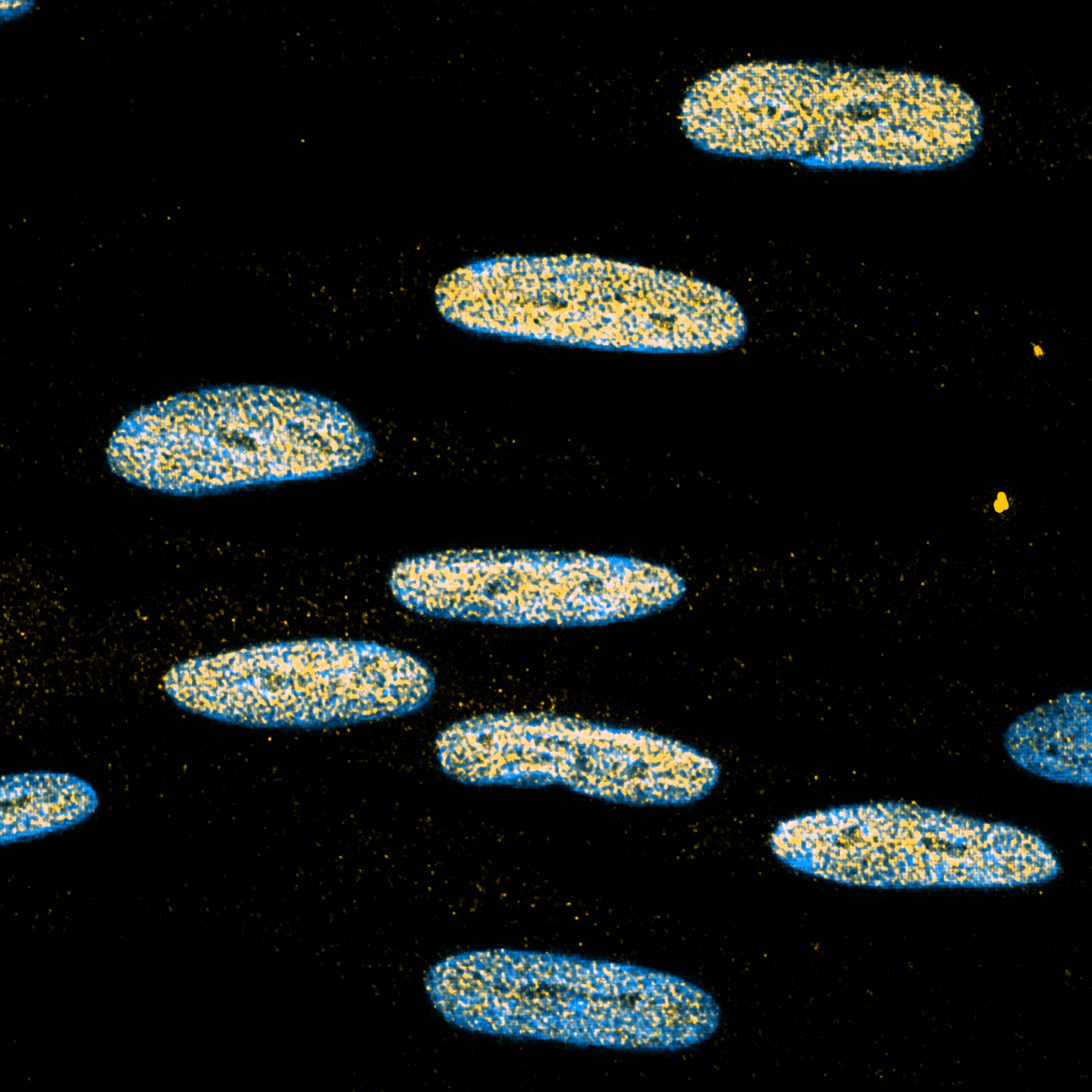

Supplement: Supplementary file 12 — Source data Fig. 6 [file 44318_2024_337_MOESM12_ESM.zip › 06_Figure_06/6H/TP53-WT+N/TP53-WT+N_Merge.tif]

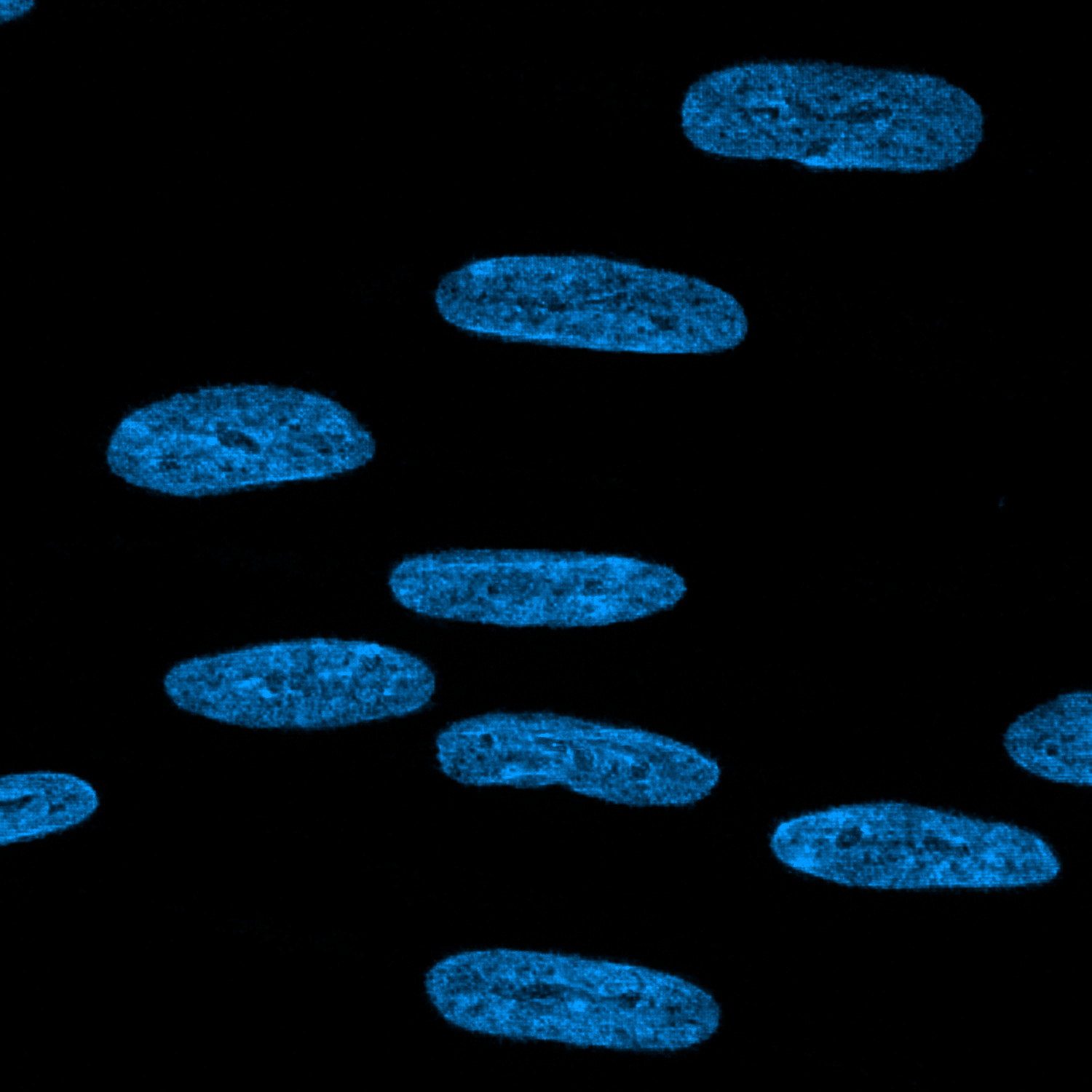

Supplement: Supplementary file 12 — Source data Fig. 6 [file 44318_2024_337_MOESM12_ESM.zip › 06_Figure_06/6H/TP53-WT+N/TP53-WT+N_RGB_DAPI.tif]

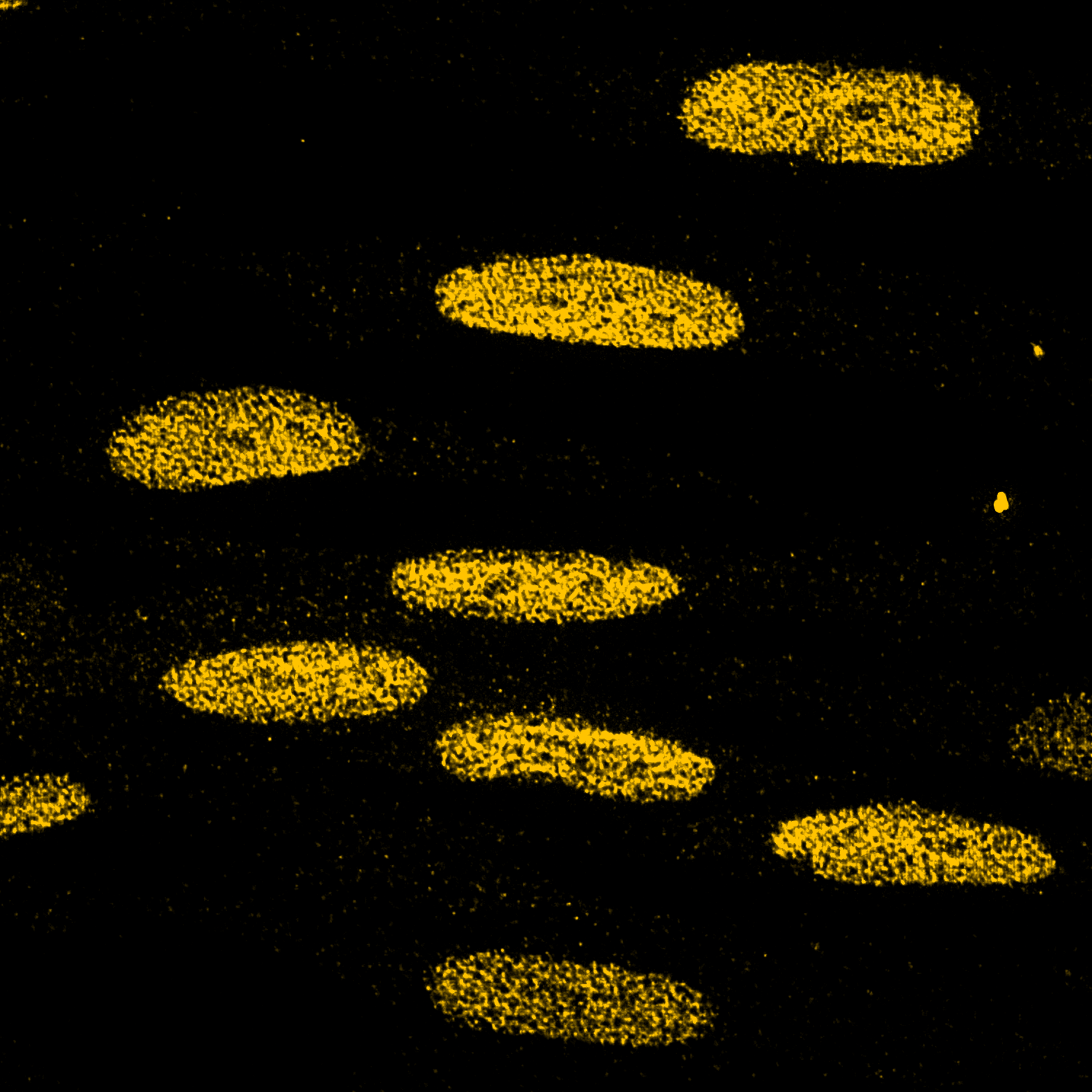

Supplement: Supplementary file 12 — Source data Fig. 6 [file 44318_2024_337_MOESM12_ESM.zip › 06_Figure_06/6H/TP53-WT+N/TP53-WT+N_TP53.tif]

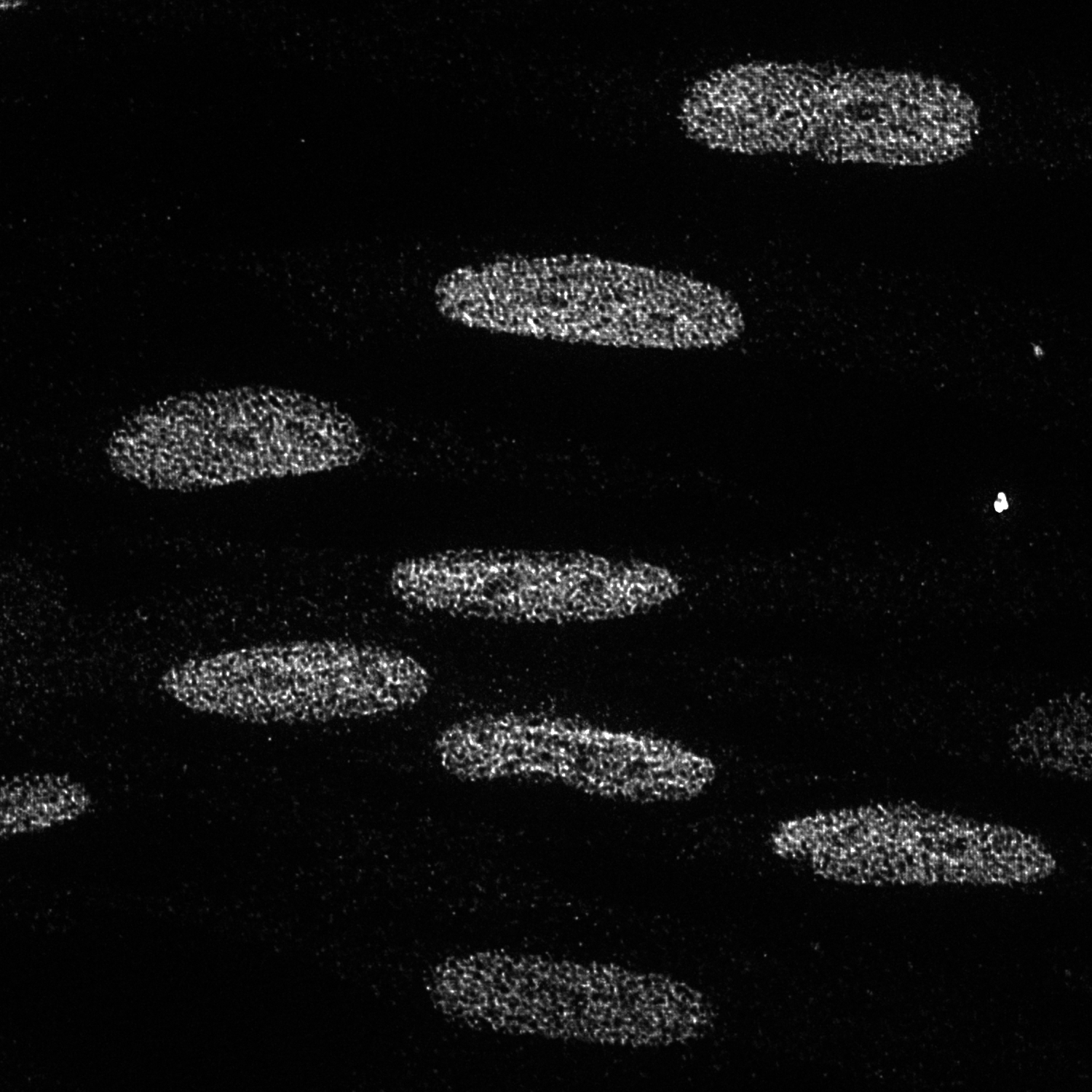

Supplement: Supplementary file 12 — Source data Fig. 6 [file 44318_2024_337_MOESM12_ESM.zip › 06_Figure_06/6H/TP53-WT+N/_FULL-RANGE-TP53-WT+N.tif]

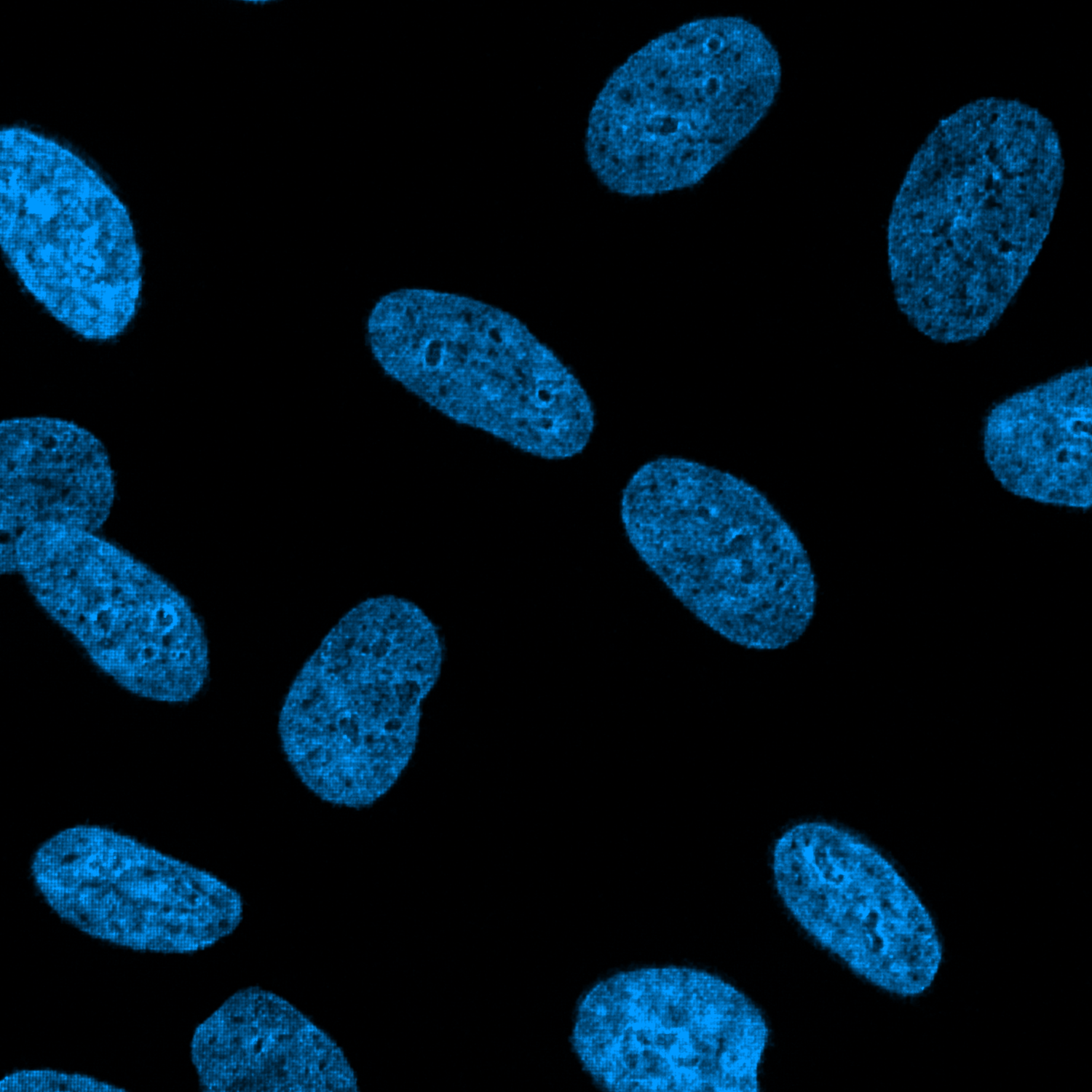

Supplement: Supplementary file 12 — Source data Fig. 6 [file 44318_2024_337_MOESM12_ESM.zip › 06_Figure_06/6H/TP53-WT-N/TP53-WT-N_DAPI.tif]

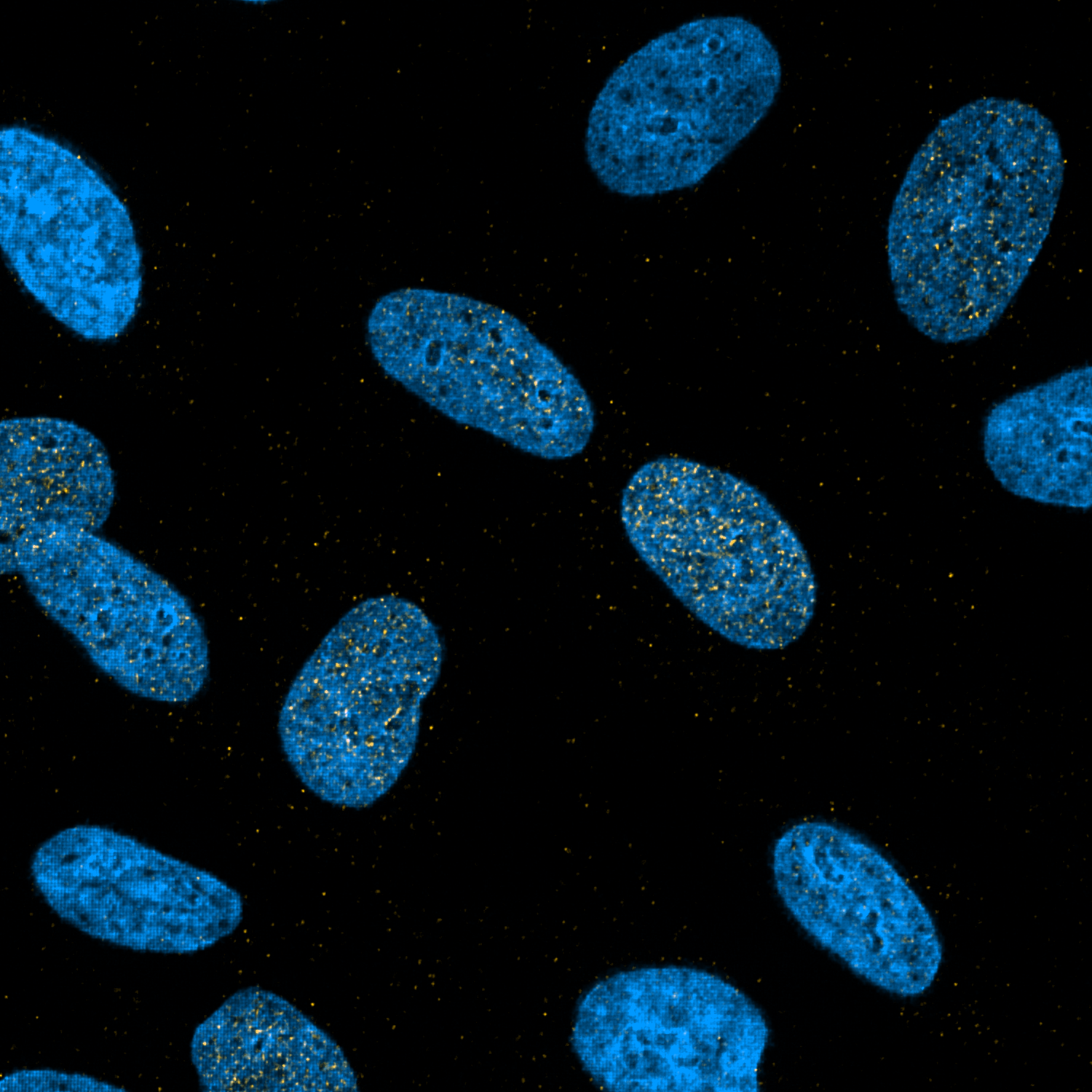

Supplement: Supplementary file 12 — Source data Fig. 6 [file 44318_2024_337_MOESM12_ESM.zip › 06_Figure_06/6H/TP53-WT-N/TP53-WT-N_Merge.tif]

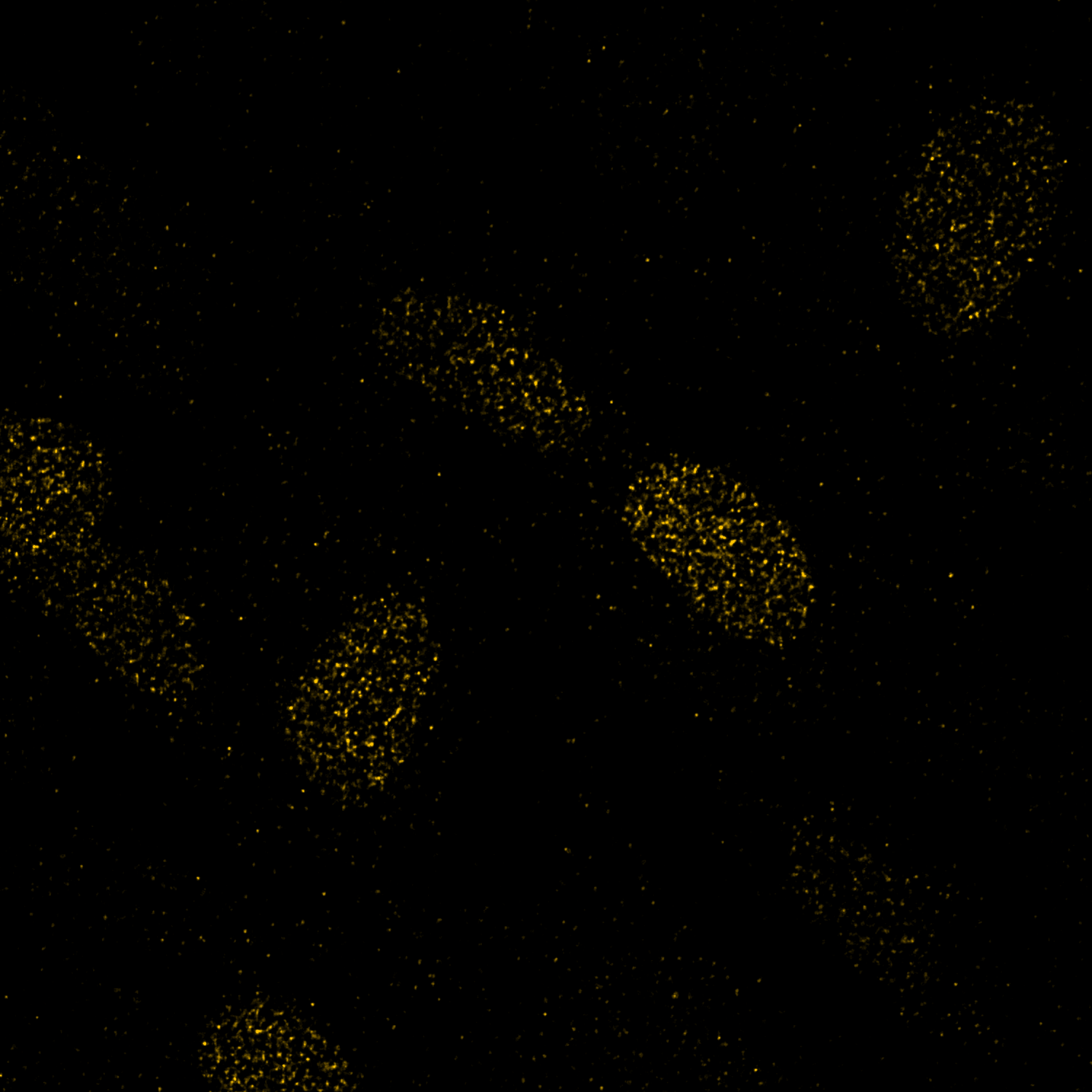

Supplement: Supplementary file 12 — Source data Fig. 6 [file 44318_2024_337_MOESM12_ESM.zip › 06_Figure_06/6H/TP53-WT-N/TP53-WT-N_TP53.tif]

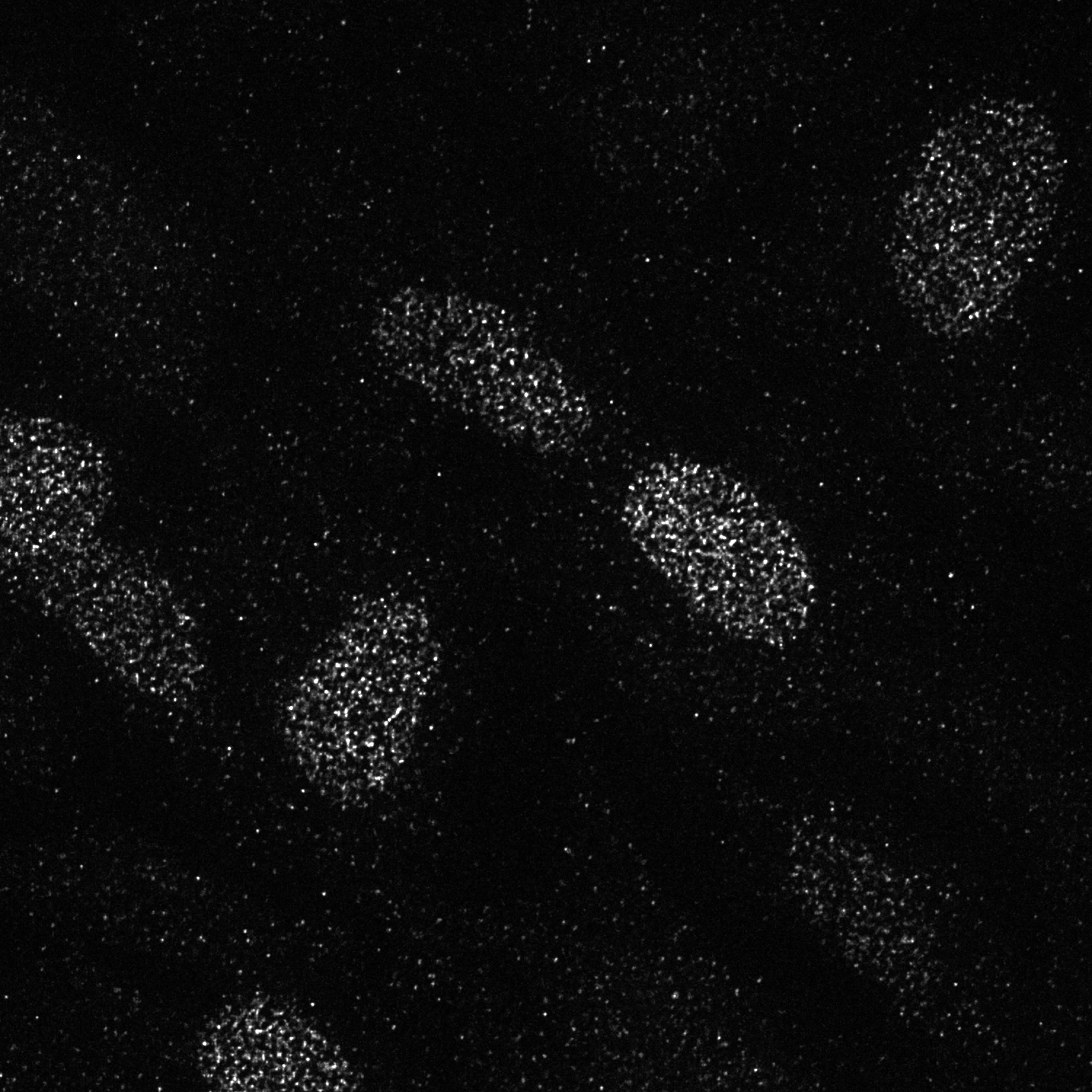

Supplement: Supplementary file 12 — Source data Fig. 6 [file 44318_2024_337_MOESM12_ESM.zip › 06_Figure_06/6H/TP53-WT-N/_FULL-RANGE-TP53-WT-N.tif]

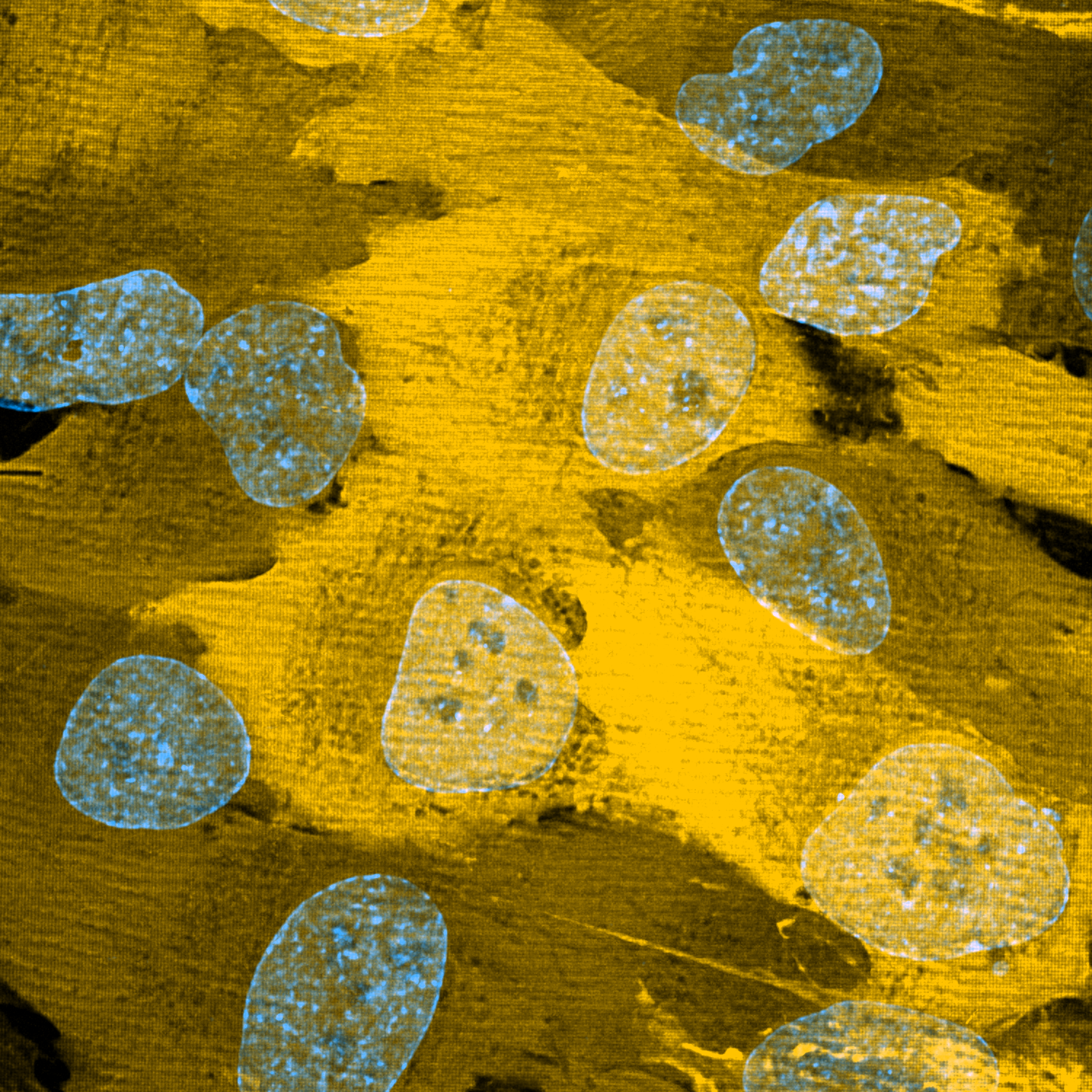

Supplement: Supplementary file 12 — Source data Fig. 6 [file 44318_2024_337_MOESM12_ESM.zip › 06_Figure_06/6K/AAVS1-EF1A-GFP/AAVS1-EF1A-GFP_Merge.tif]

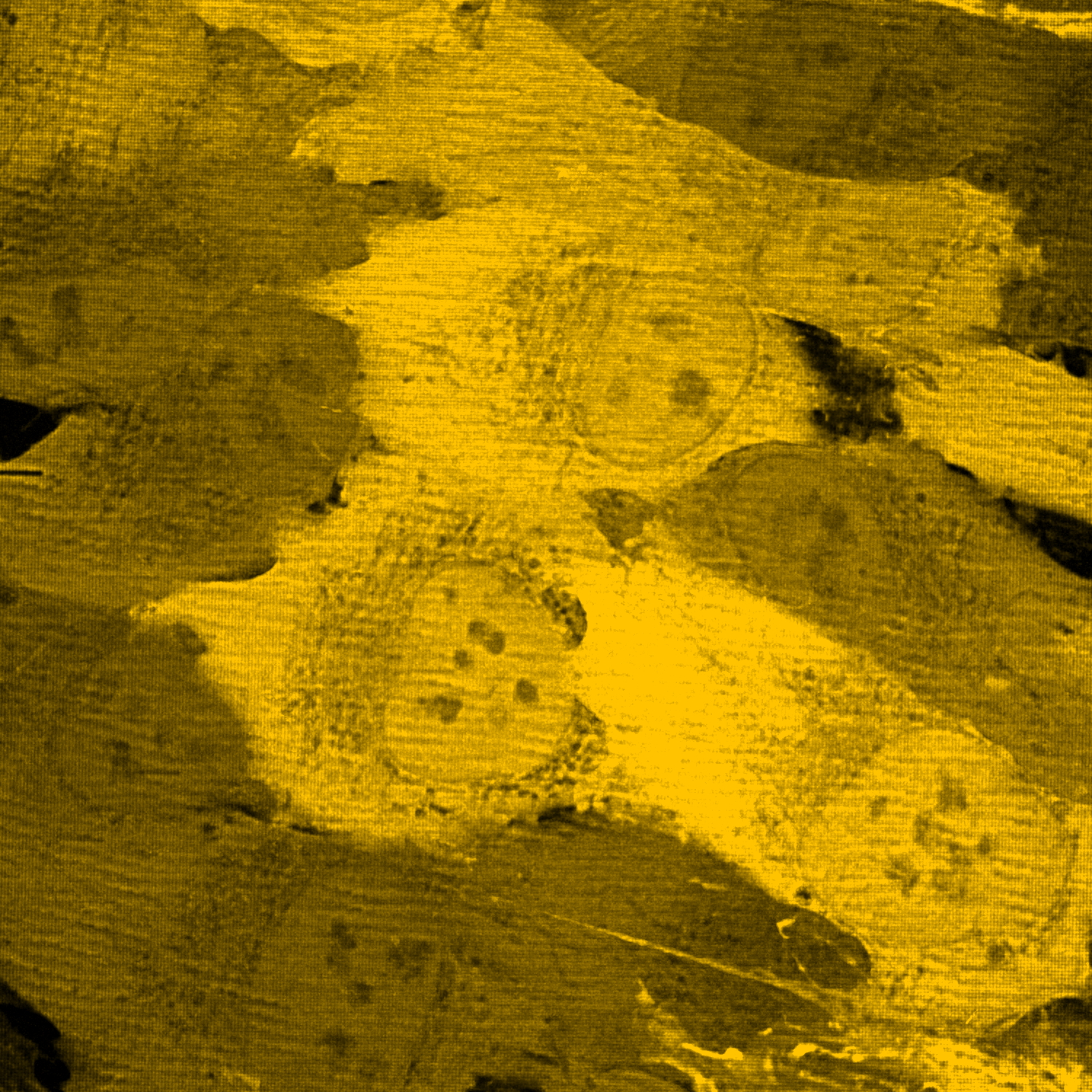

Supplement: Supplementary file 12 — Source data Fig. 6 [file 44318_2024_337_MOESM12_ESM.zip › 06_Figure_06/6K/AAVS1-EF1A-GFP/AAVS1-EF1A-GFP_moxGFP.tif]

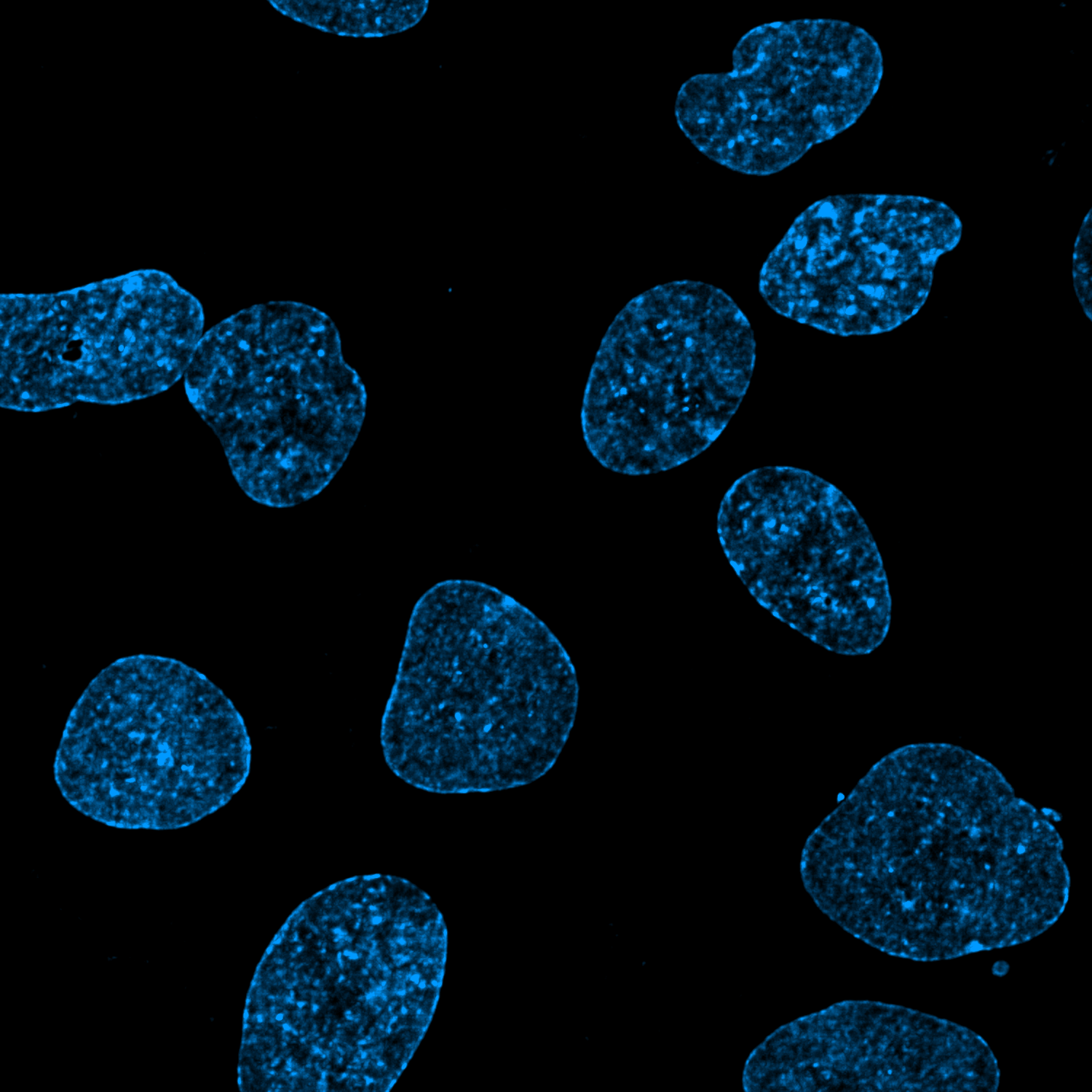

Supplement: Supplementary file 12 — Source data Fig. 6 [file 44318_2024_337_MOESM12_ESM.zip › 06_Figure_06/6K/AAVS1-EF1A-GFP/AAVS1-EF1A-GFP_SiR-DNA.tif]

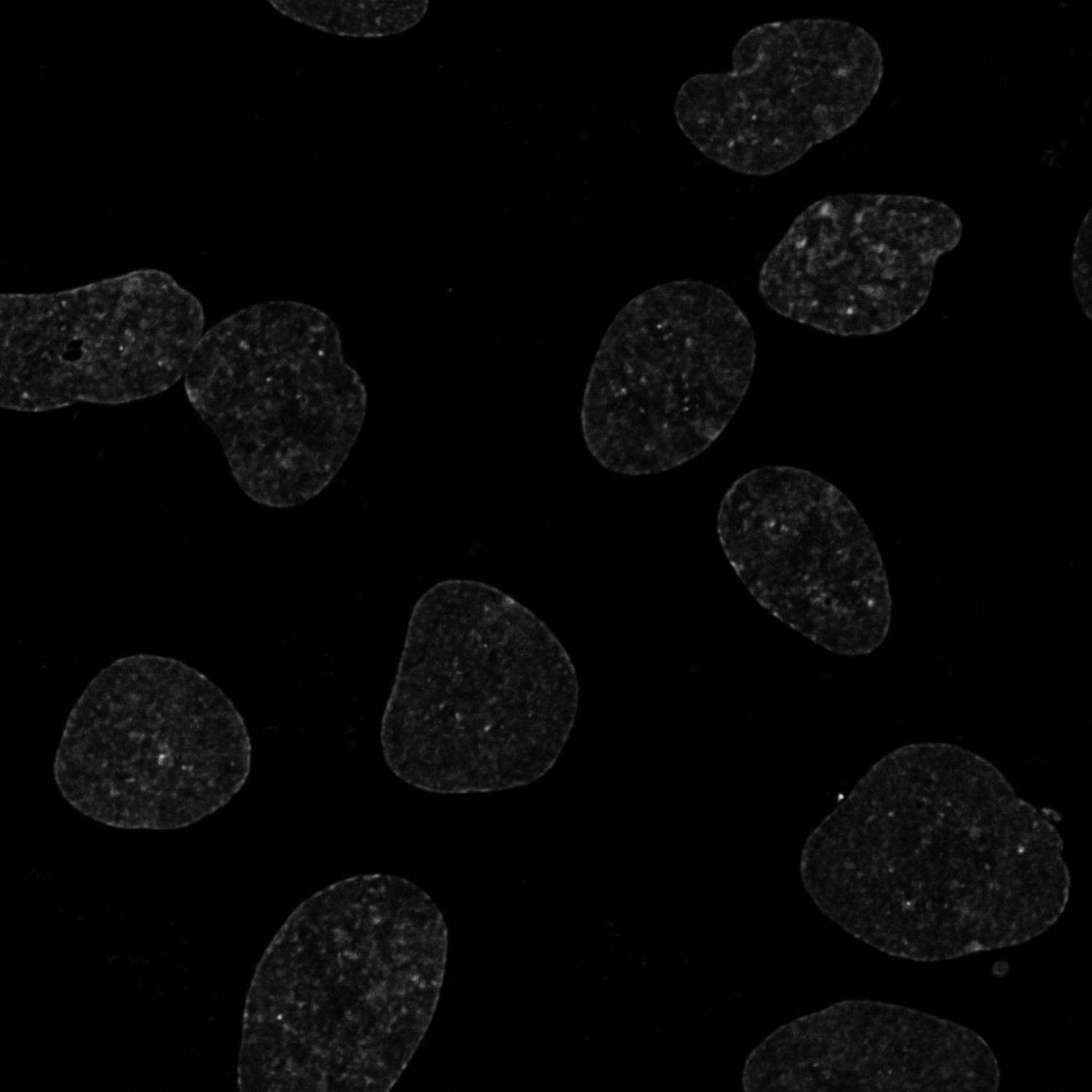

Supplement: Supplementary file 12 — Source data Fig. 6 [file 44318_2024_337_MOESM12_ESM.zip › 06_Figure_06/6K/AAVS1-EF1A-GFP/_FULL-RANGE-AAVS1-EF1A-GFP.tif]

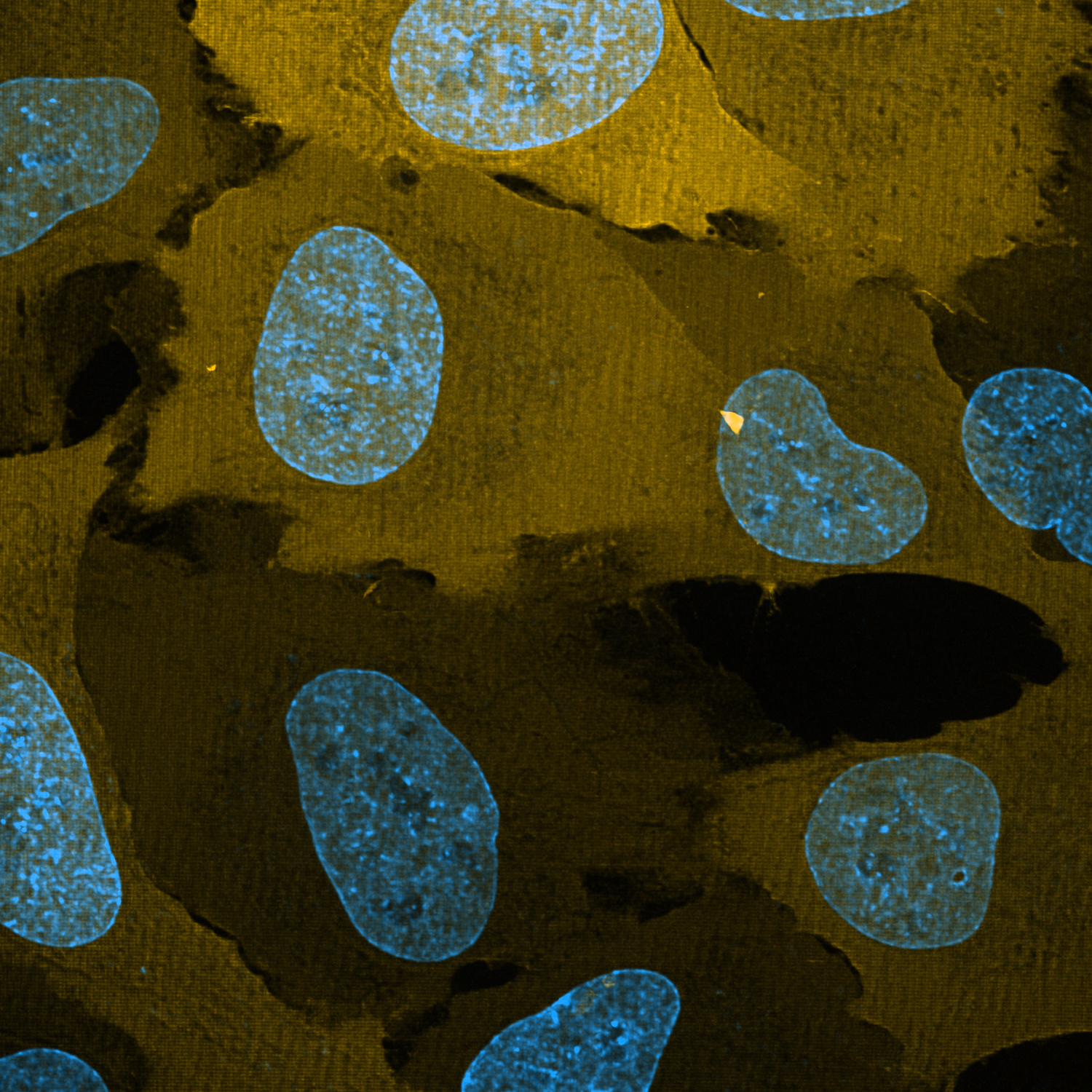

Supplement: Supplementary file 12 — Source data Fig. 6 [file 44318_2024_337_MOESM12_ESM.zip › 06_Figure_06/6K/AAVS1-PGK-GFP/AAVS1-PGK-GFP_Merge.tif]

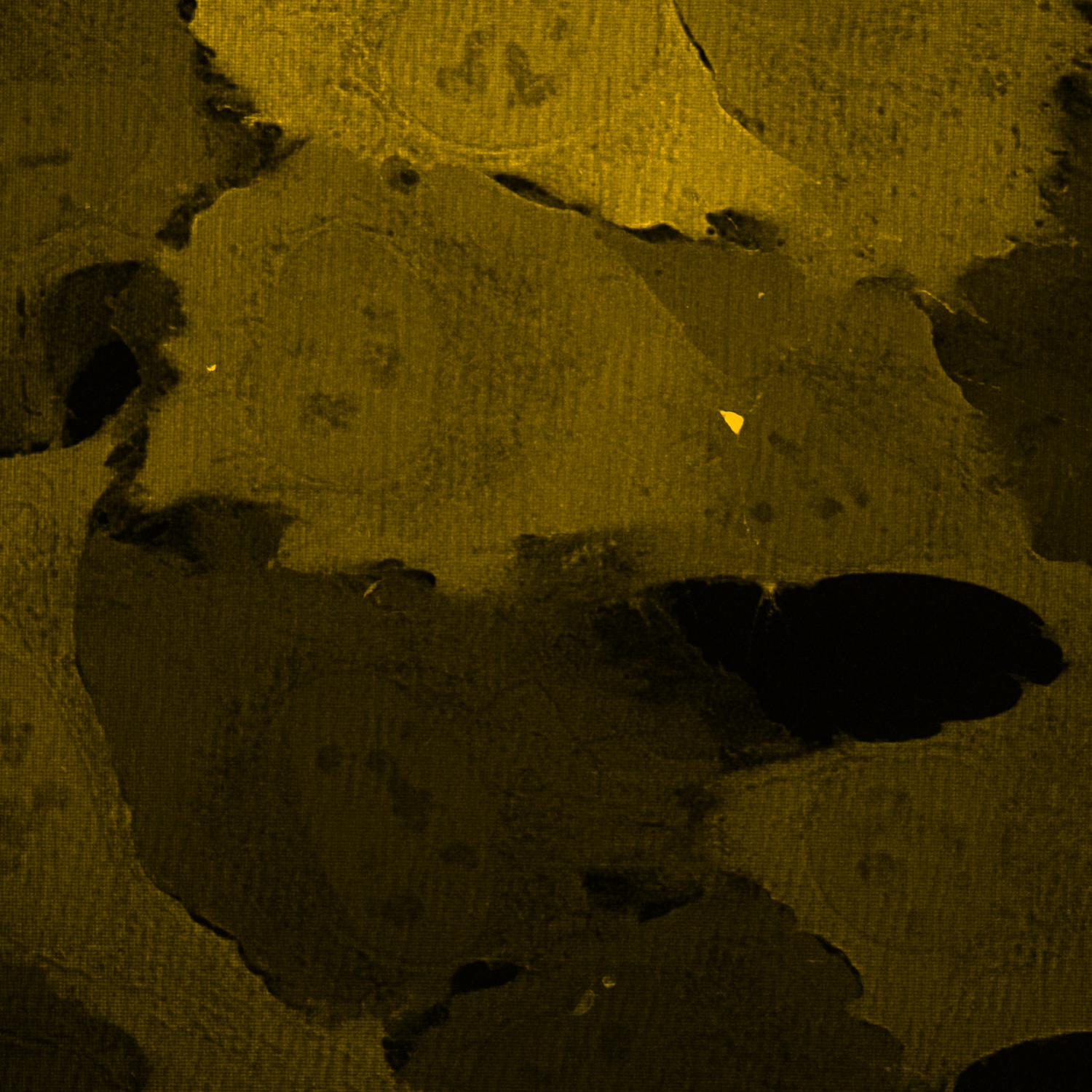

Supplement: Supplementary file 12 — Source data Fig. 6 [file 44318_2024_337_MOESM12_ESM.zip › 06_Figure_06/6K/AAVS1-PGK-GFP/AAVS1-PGK-GFP_moxGFP.tif]

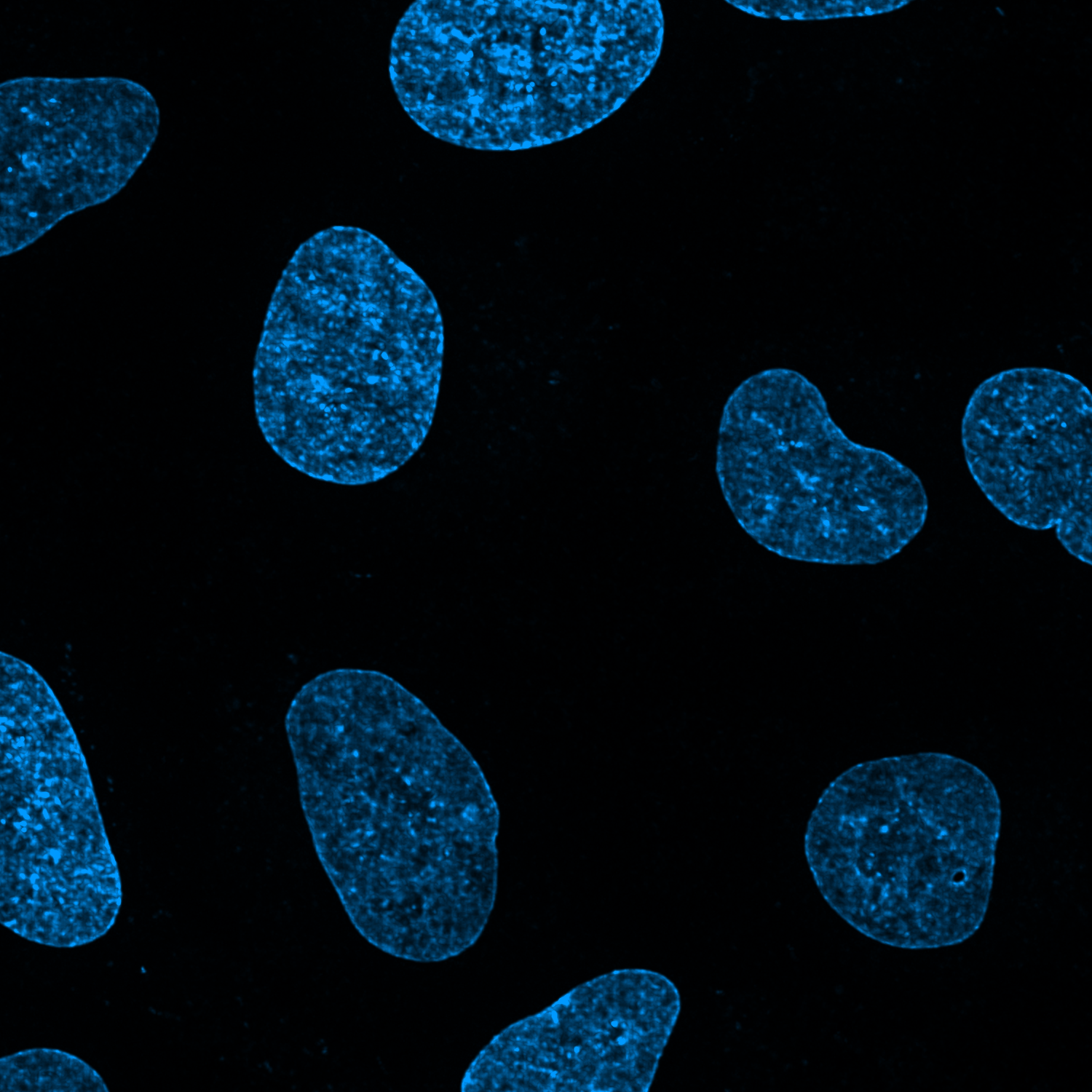

Supplement: Supplementary file 12 — Source data Fig. 6 [file 44318_2024_337_MOESM12_ESM.zip › 06_Figure_06/6K/AAVS1-PGK-GFP/AAVS1-PGK-GFP_SiR-DNA.tif]

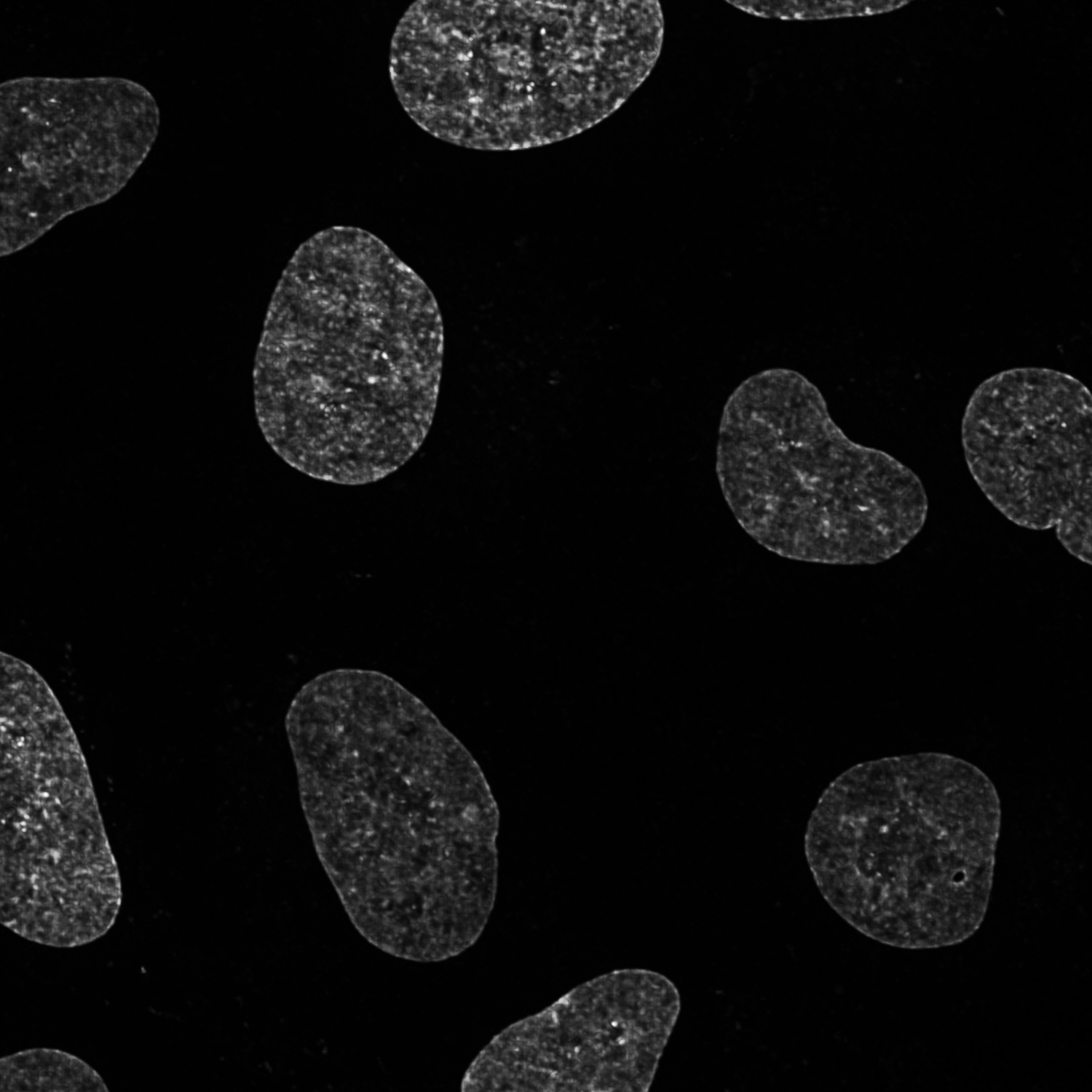

Supplement: Supplementary file 12 — Source data Fig. 6 [file 44318_2024_337_MOESM12_ESM.zip › 06_Figure_06/6K/AAVS1-PGK-GFP/_FULL-RANGE-AAVS1-PGK-GFP.tif]

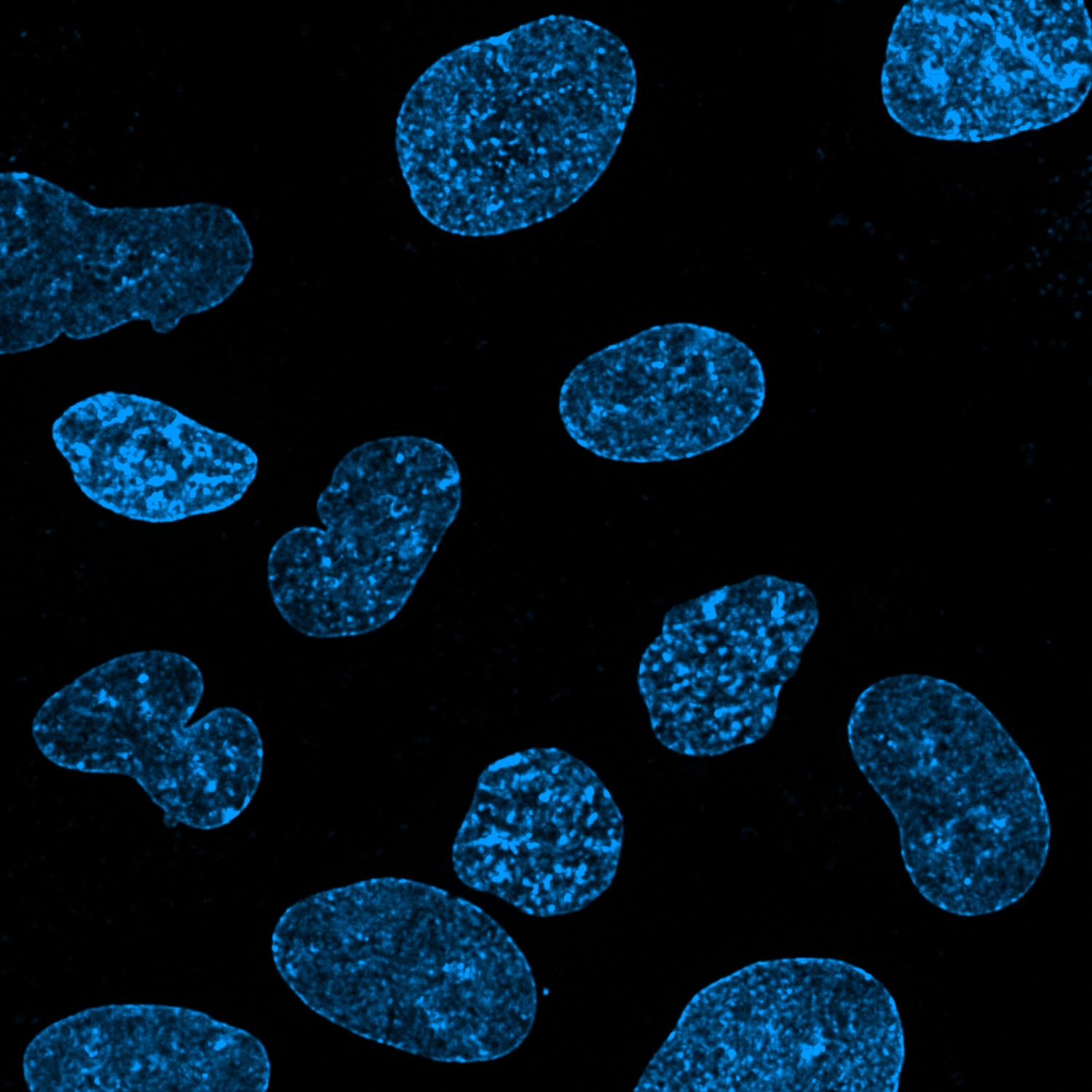

Supplement: Supplementary file 12 — Source data Fig. 6 [file 44318_2024_337_MOESM12_ESM.zip › 06_Figure_06/6K/AAVS1-WT/AAVS1-WT_Merge.tif]

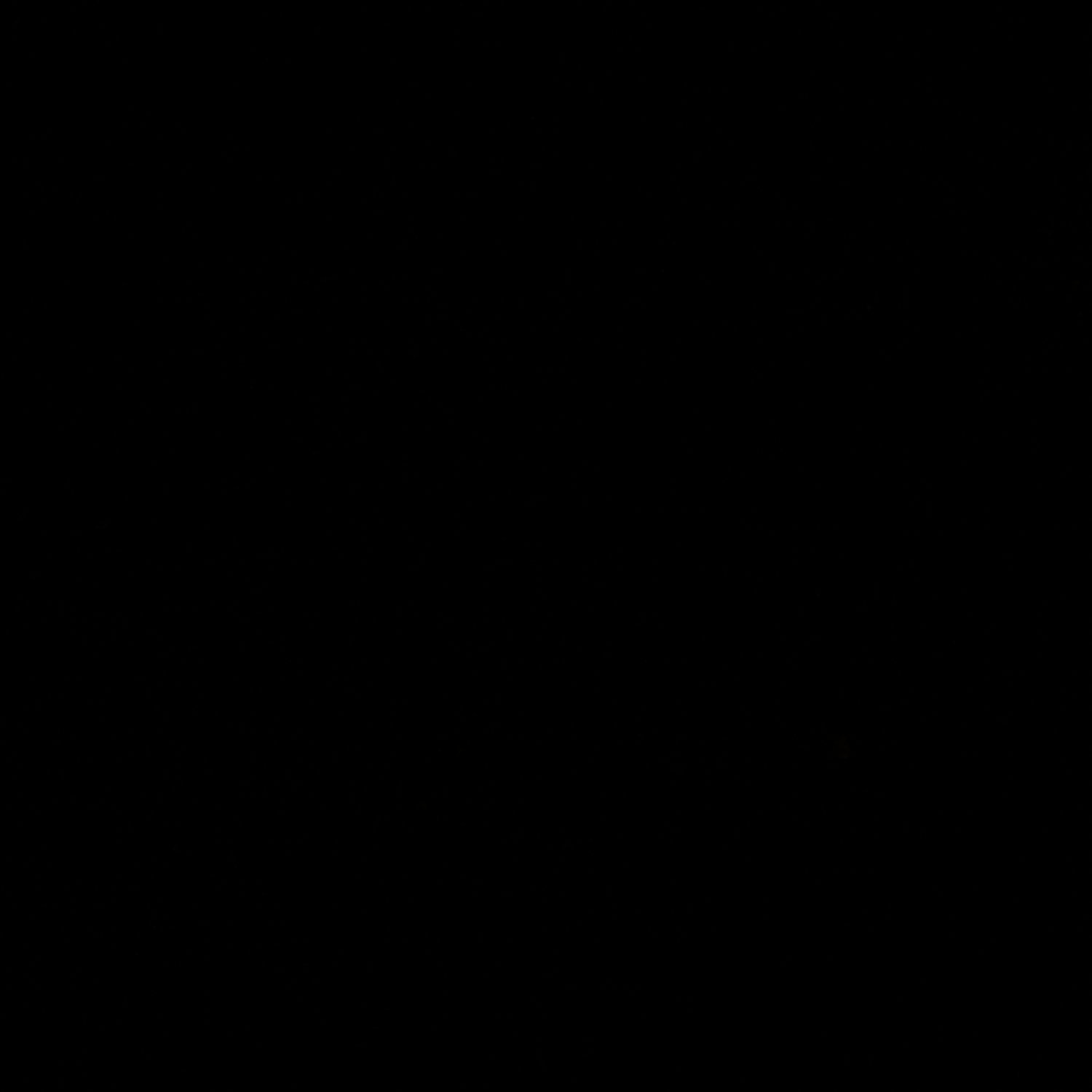

Supplement: Supplementary file 12 — Source data Fig. 6 [file 44318_2024_337_MOESM12_ESM.zip › 06_Figure_06/6K/AAVS1-WT/AAVS1-WT_moxGFP.tif]

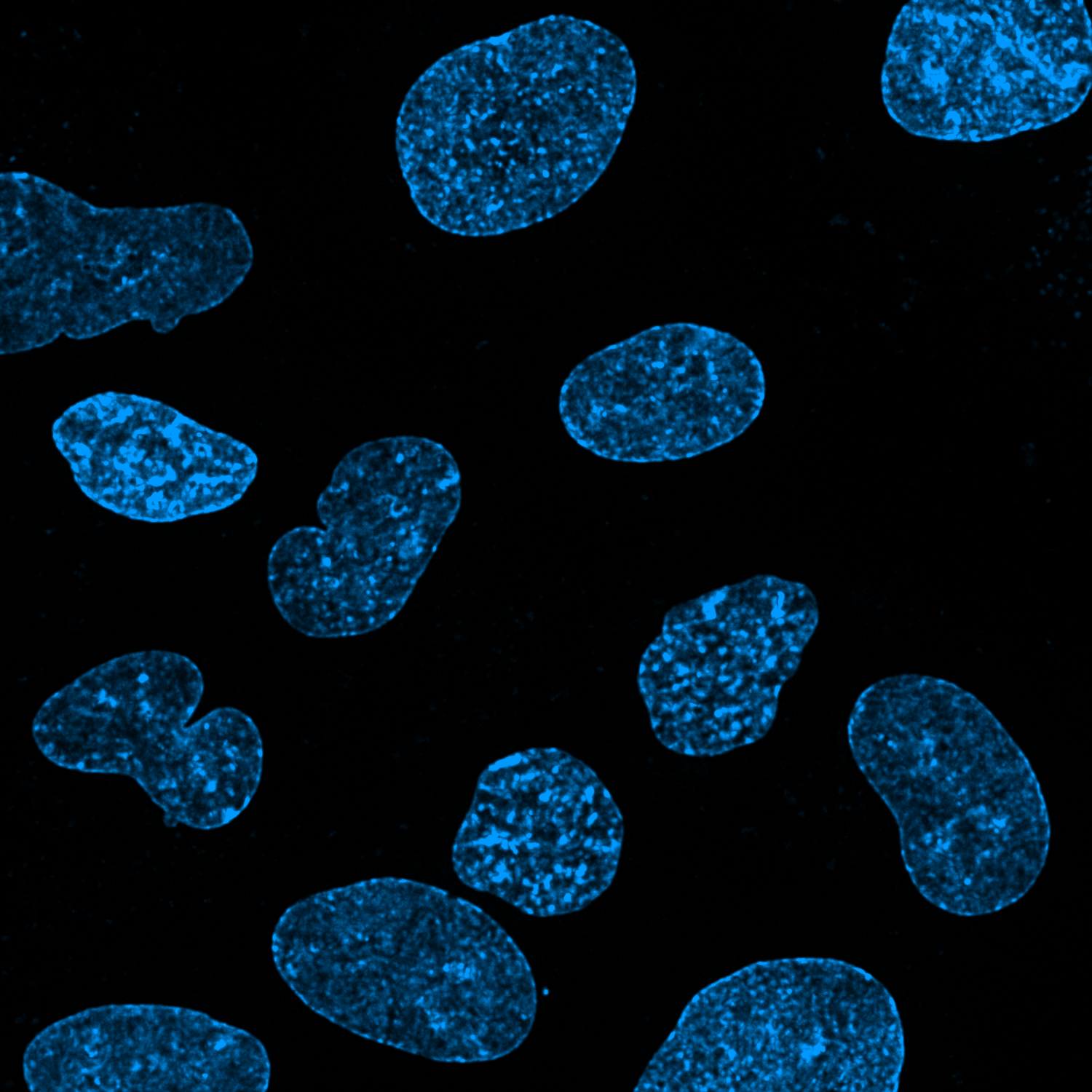

Supplement: Supplementary file 12 — Source data Fig. 6 [file 44318_2024_337_MOESM12_ESM.zip › 06_Figure_06/6K/AAVS1-WT/AAVS1-WT_SiR-DNA.tif]

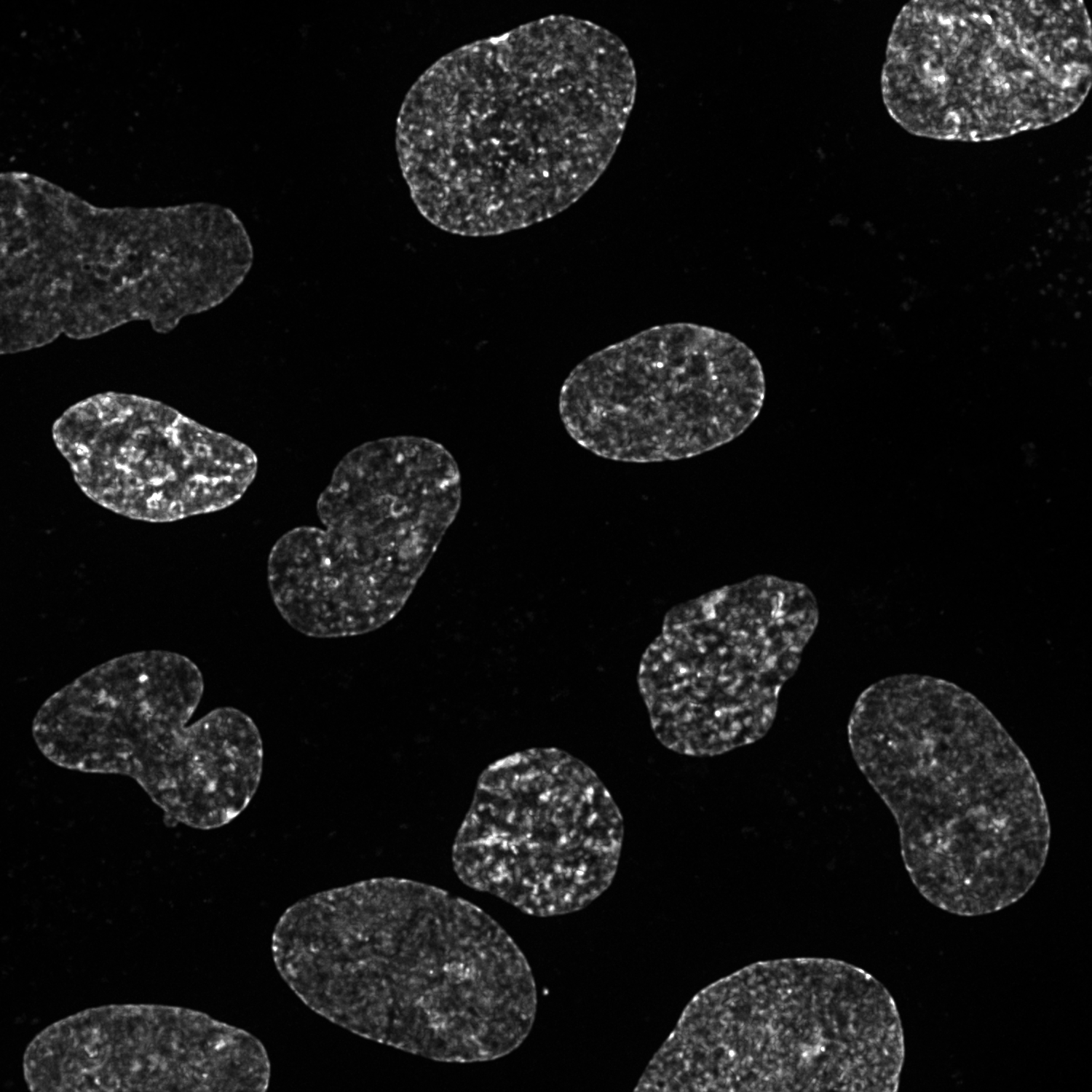

Supplement: Supplementary file 12 — Source data Fig. 6 [file 44318_2024_337_MOESM12_ESM.zip › 06_Figure_06/6K/AAVS1-WT/_FULL-RANGE-AAVS1-WT.tif]

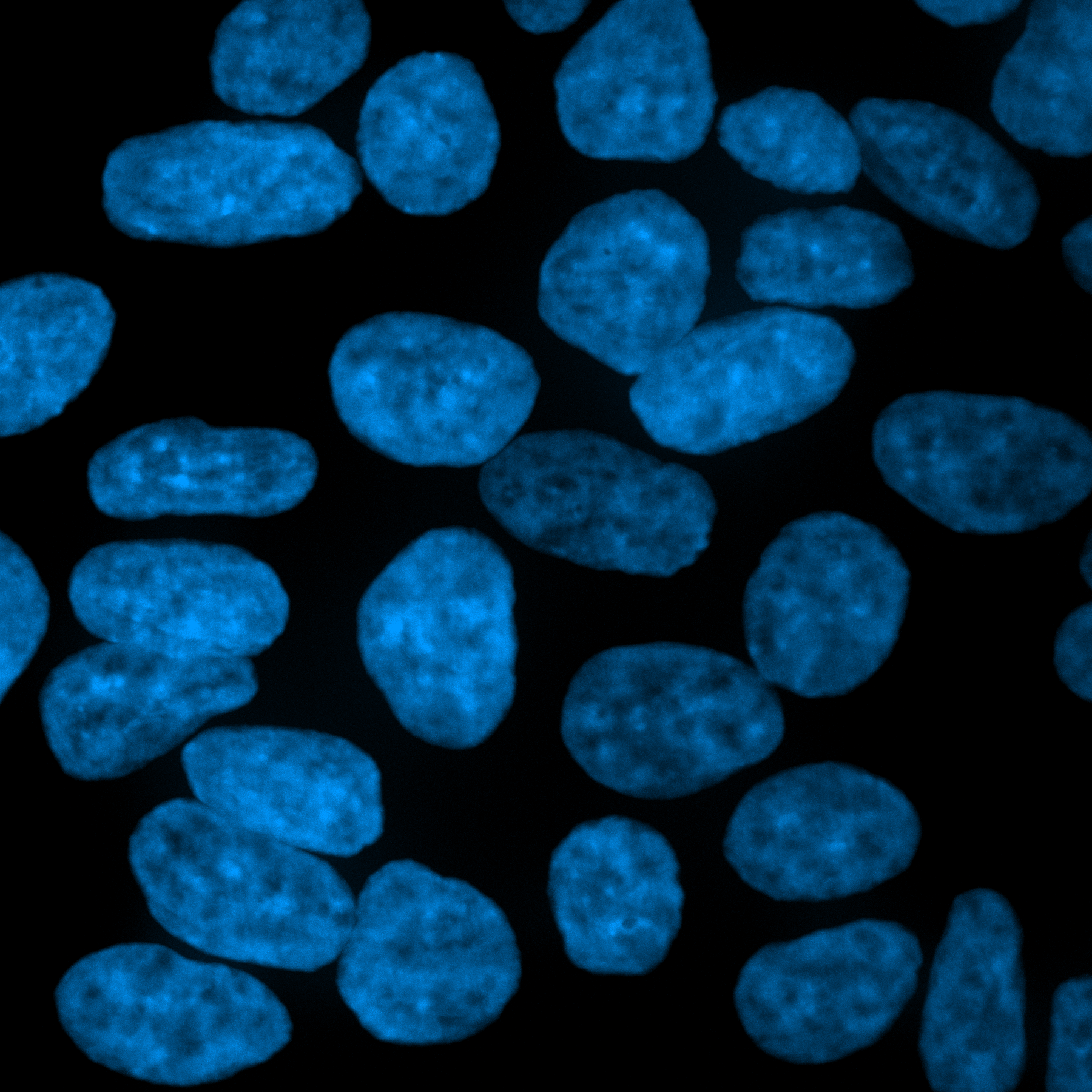

Supplement: Supplementary file 13 — Source data Figure EV1 [file 44318_2024_337_MOESM13_ESM.zip › 07_Figure_EV1/A/Imaging/HAP1-CTRL/HAP1-CTRL_DAPI.tif]

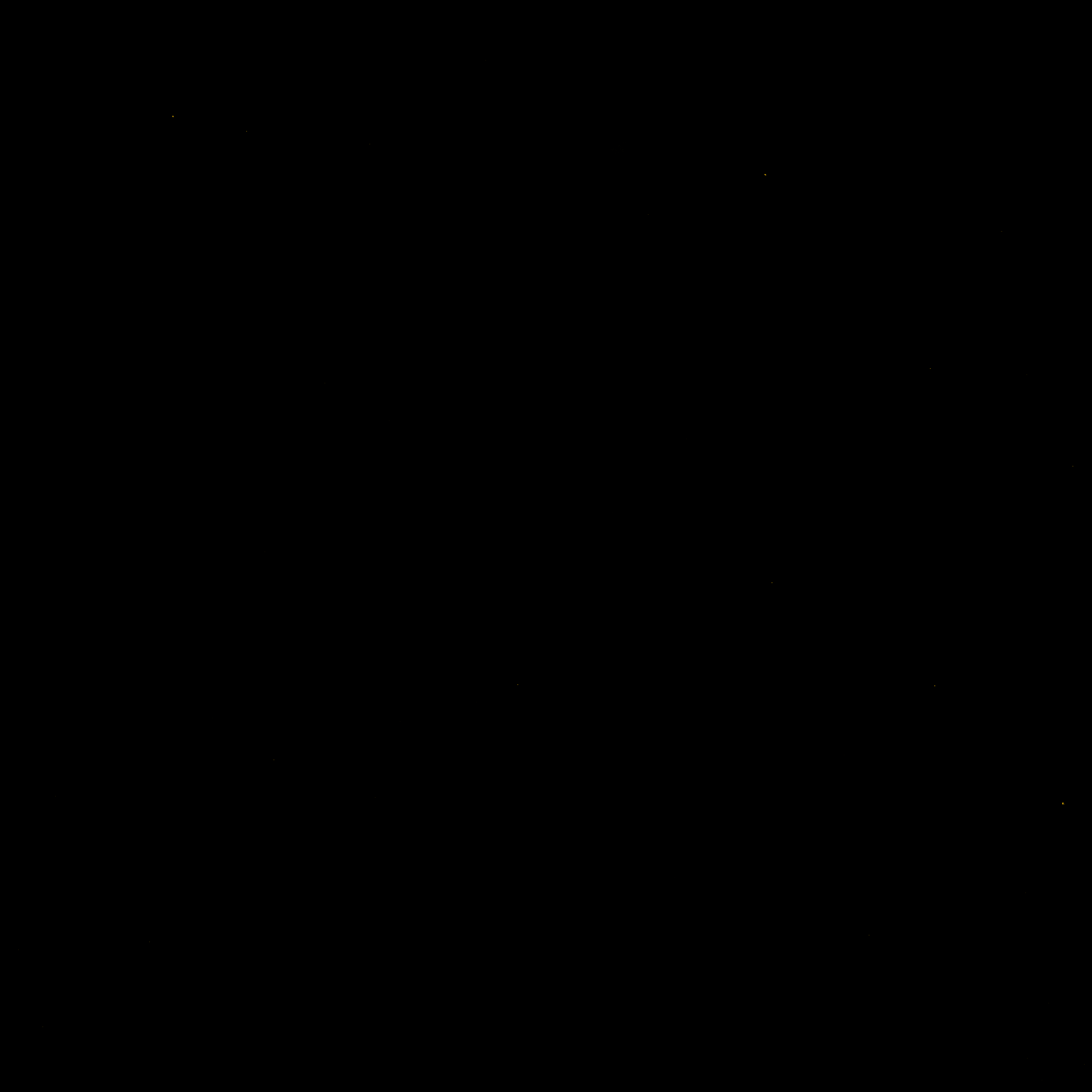

Supplement: Supplementary file 13 — Source data Figure EV1 [file 44318_2024_337_MOESM13_ESM.zip › 07_Figure_EV1/A/Imaging/HAP1-CTRL/HAP1-CTRL_GFP.tif]

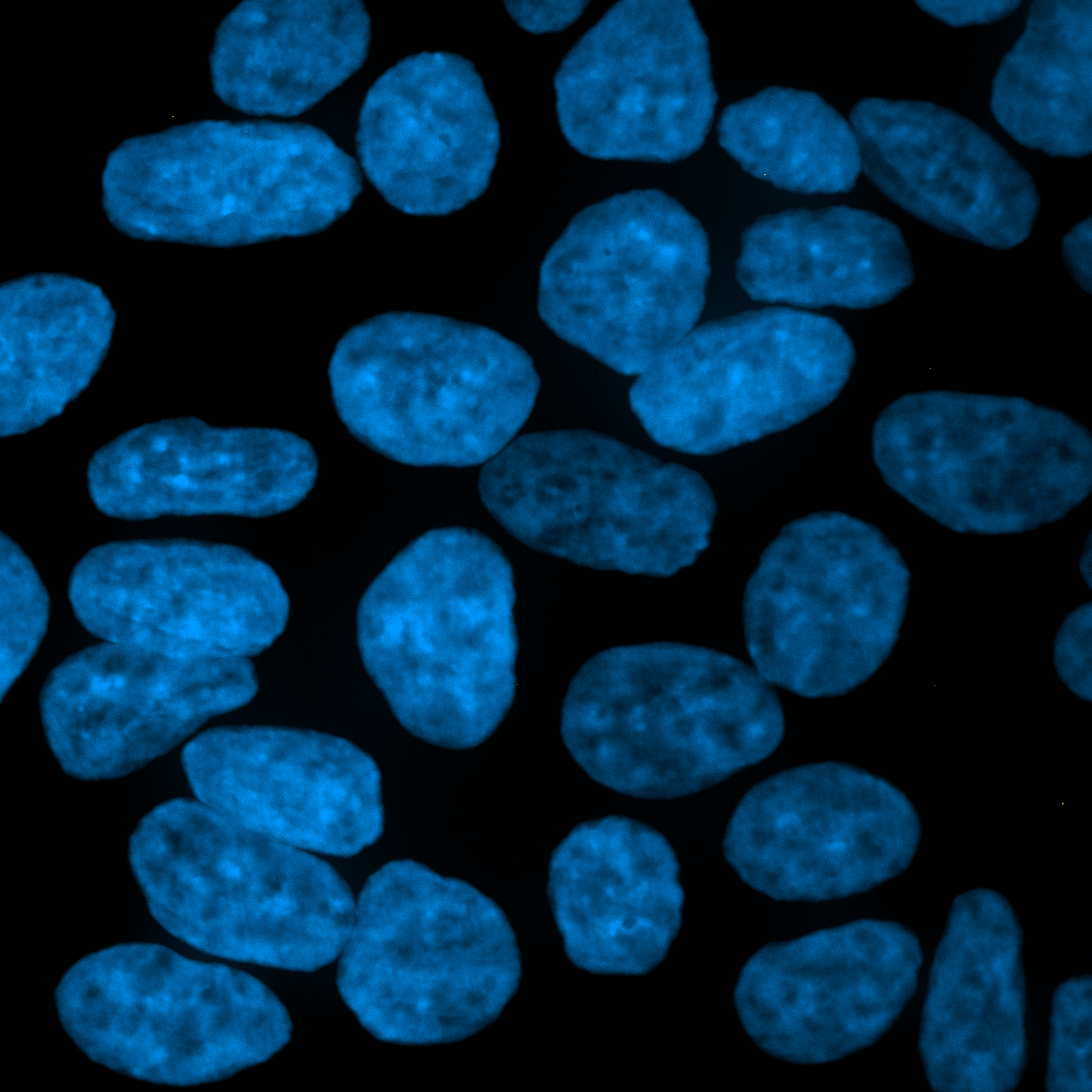

Supplement: Supplementary file 13 — Source data Figure EV1 [file 44318_2024_337_MOESM13_ESM.zip › 07_Figure_EV1/A/Imaging/HAP1-CTRL/HAP1-CTRL_Merge.tif]

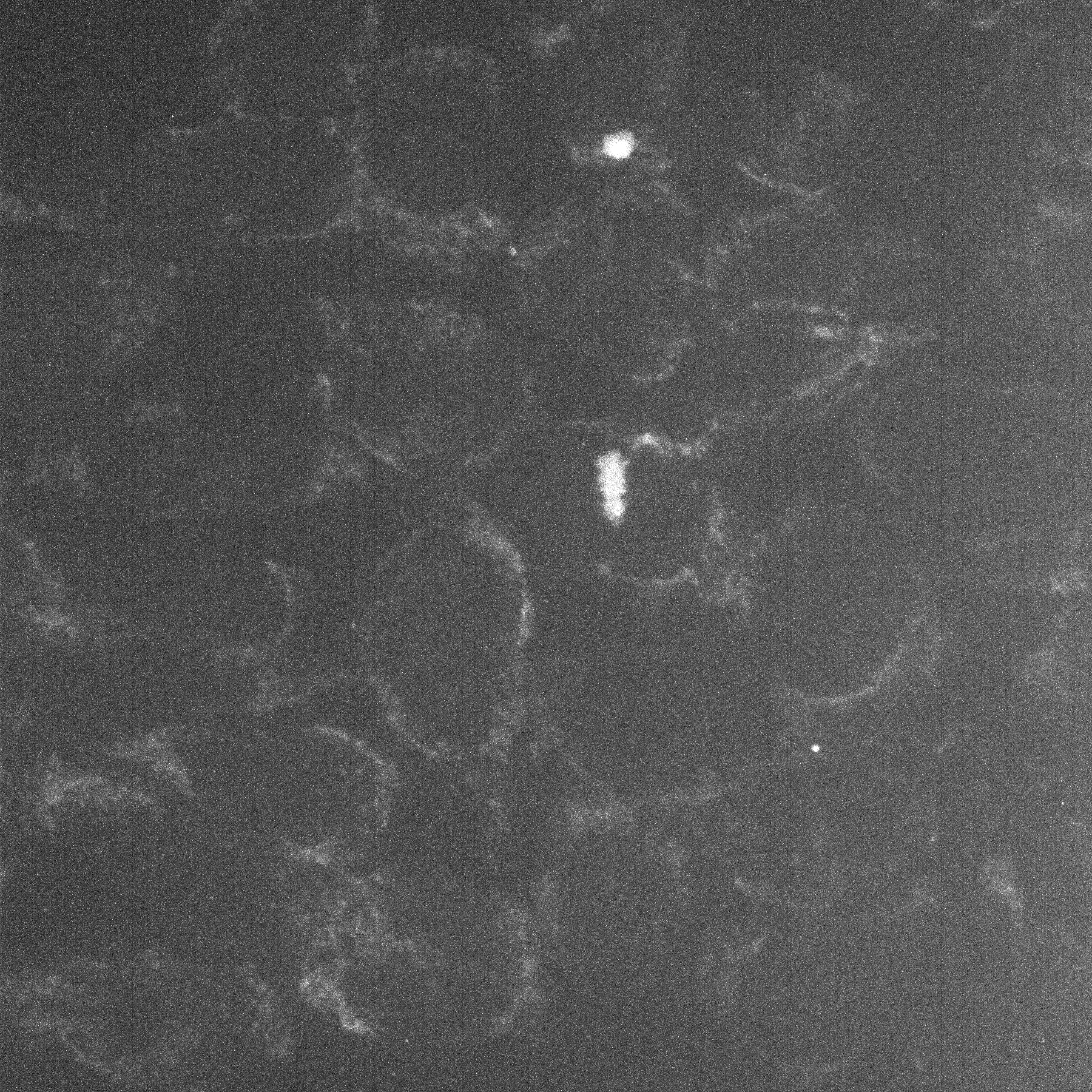

Supplement: Supplementary file 13 — Source data Figure EV1 [file 44318_2024_337_MOESM13_ESM.zip › 07_Figure_EV1/A/Imaging/HAP1-CTRL/_FULL-RANGE-HAP1-CTRL.tif]

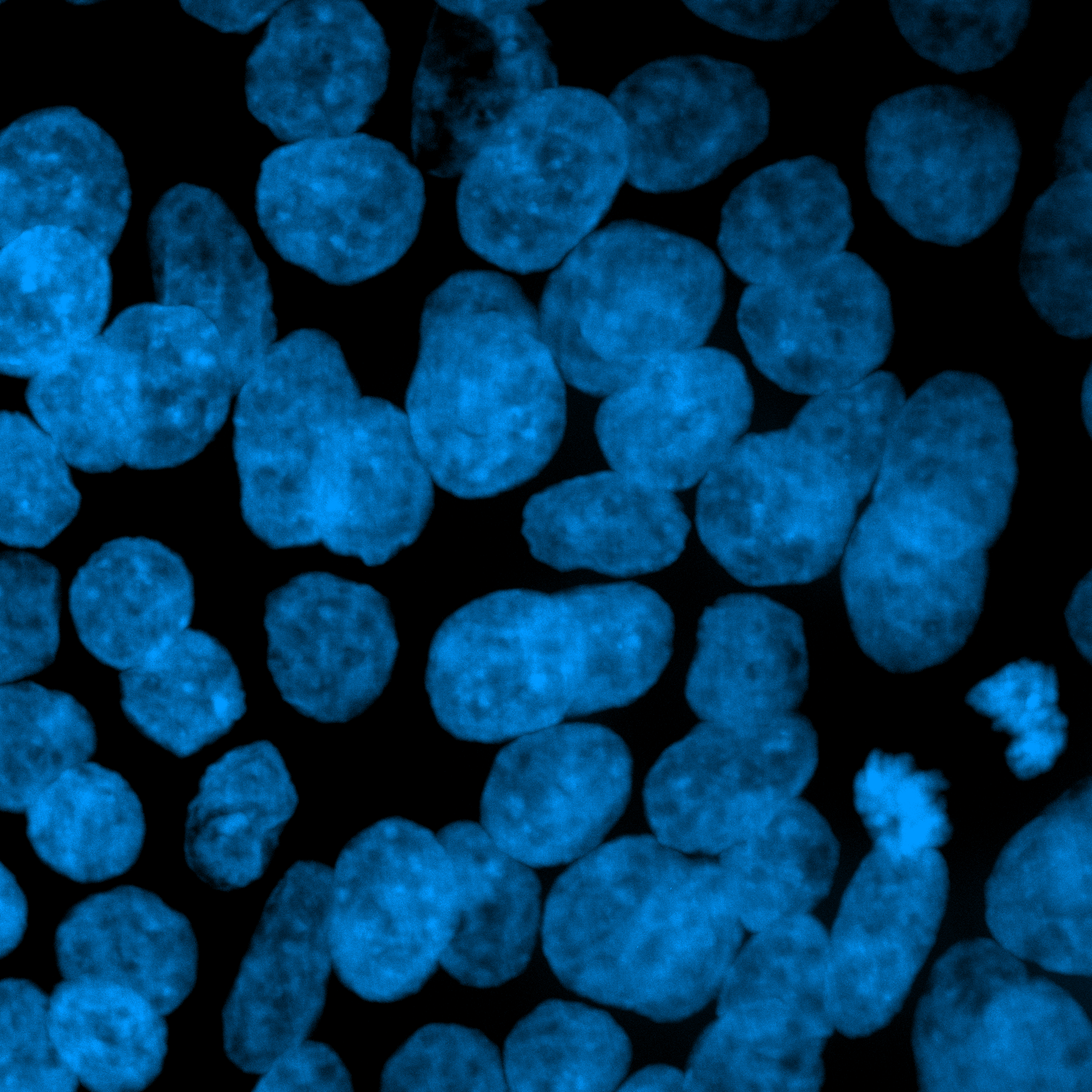

Supplement: Supplementary file 13 — Source data Figure EV1 [file 44318_2024_337_MOESM13_ESM.zip › 07_Figure_EV1/A/Imaging/HAP1-HDR/HAP1-HDR_DAPI.tif]

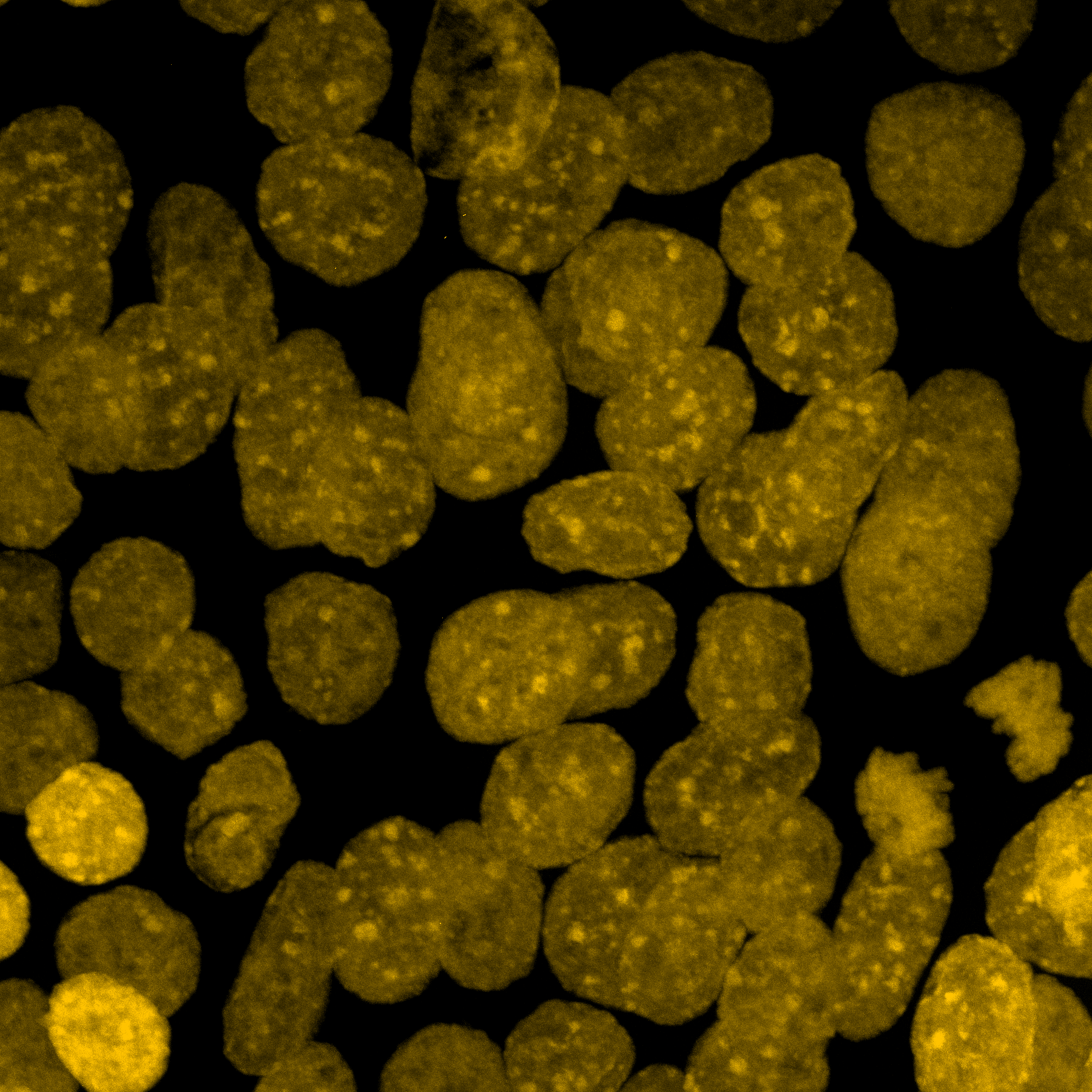

Supplement: Supplementary file 13 — Source data Figure EV1 [file 44318_2024_337_MOESM13_ESM.zip › 07_Figure_EV1/A/Imaging/HAP1-HDR/HAP1-HDR_GFP.tif]

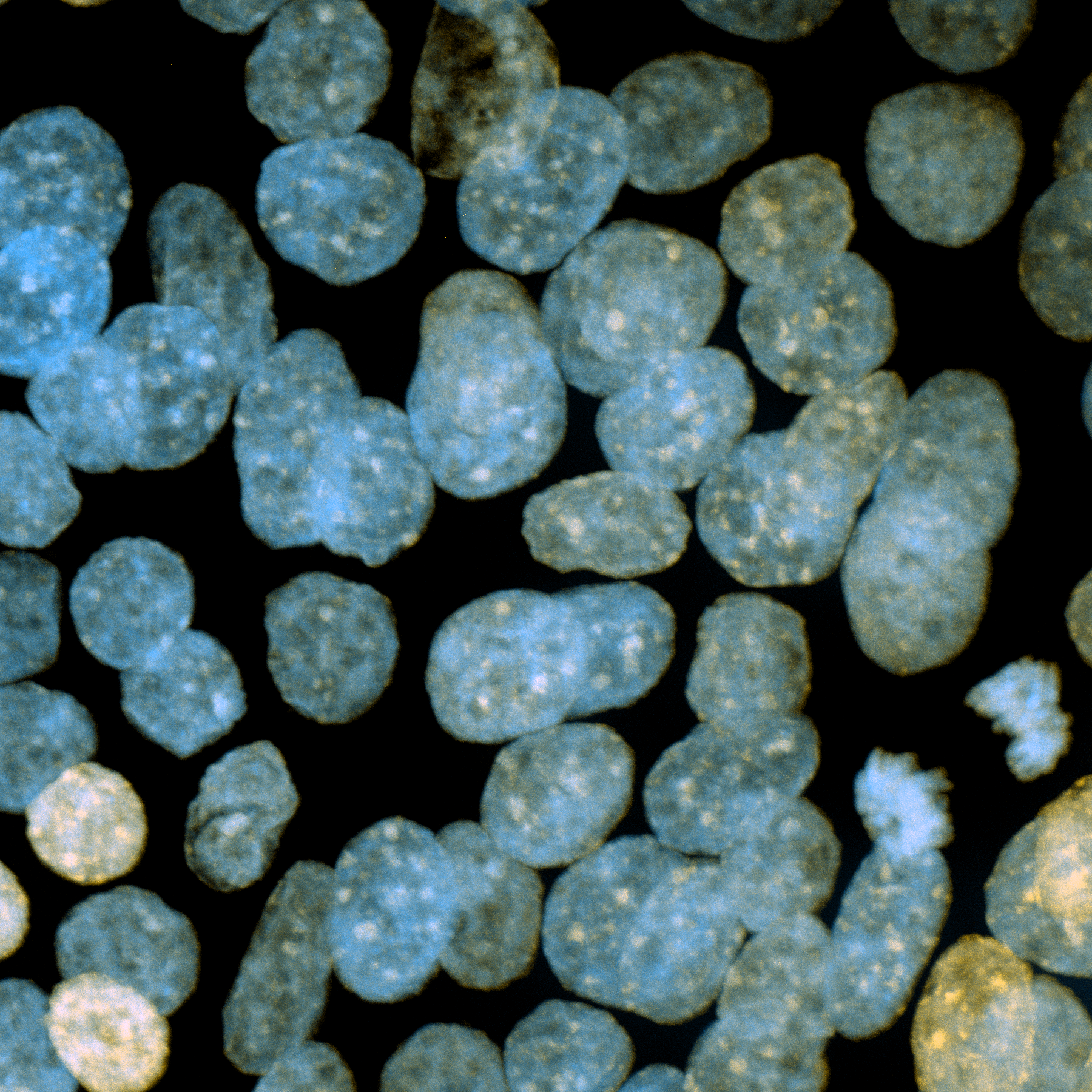

Supplement: Supplementary file 13 — Source data Figure EV1 [file 44318_2024_337_MOESM13_ESM.zip › 07_Figure_EV1/A/Imaging/HAP1-HDR/HAP1-HDR_Merge.tif]

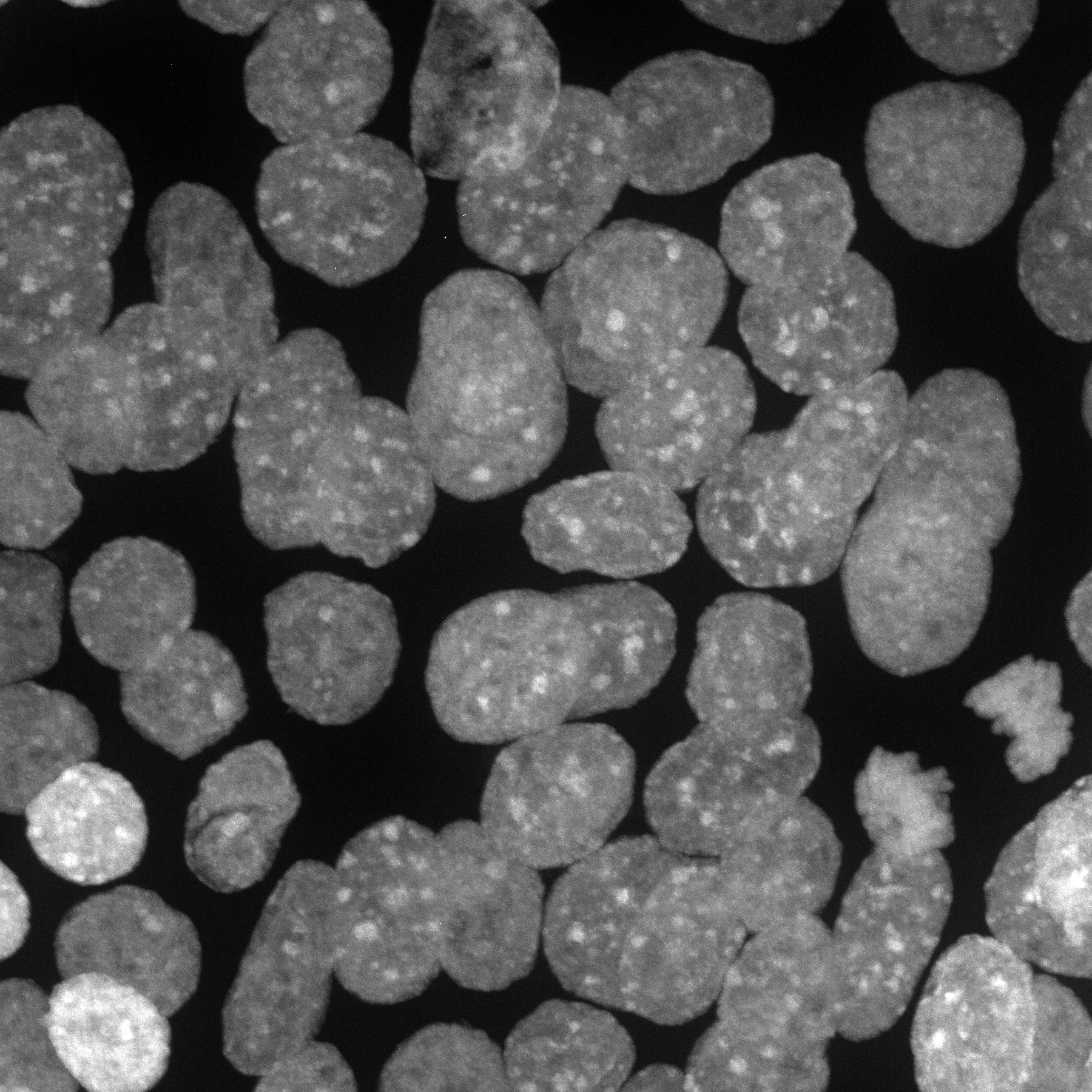

Supplement: Supplementary file 13 — Source data Figure EV1 [file 44318_2024_337_MOESM13_ESM.zip › 07_Figure_EV1/A/Imaging/HAP1-HDR/_FULL-RANGE-HAP1-HDR.tif]

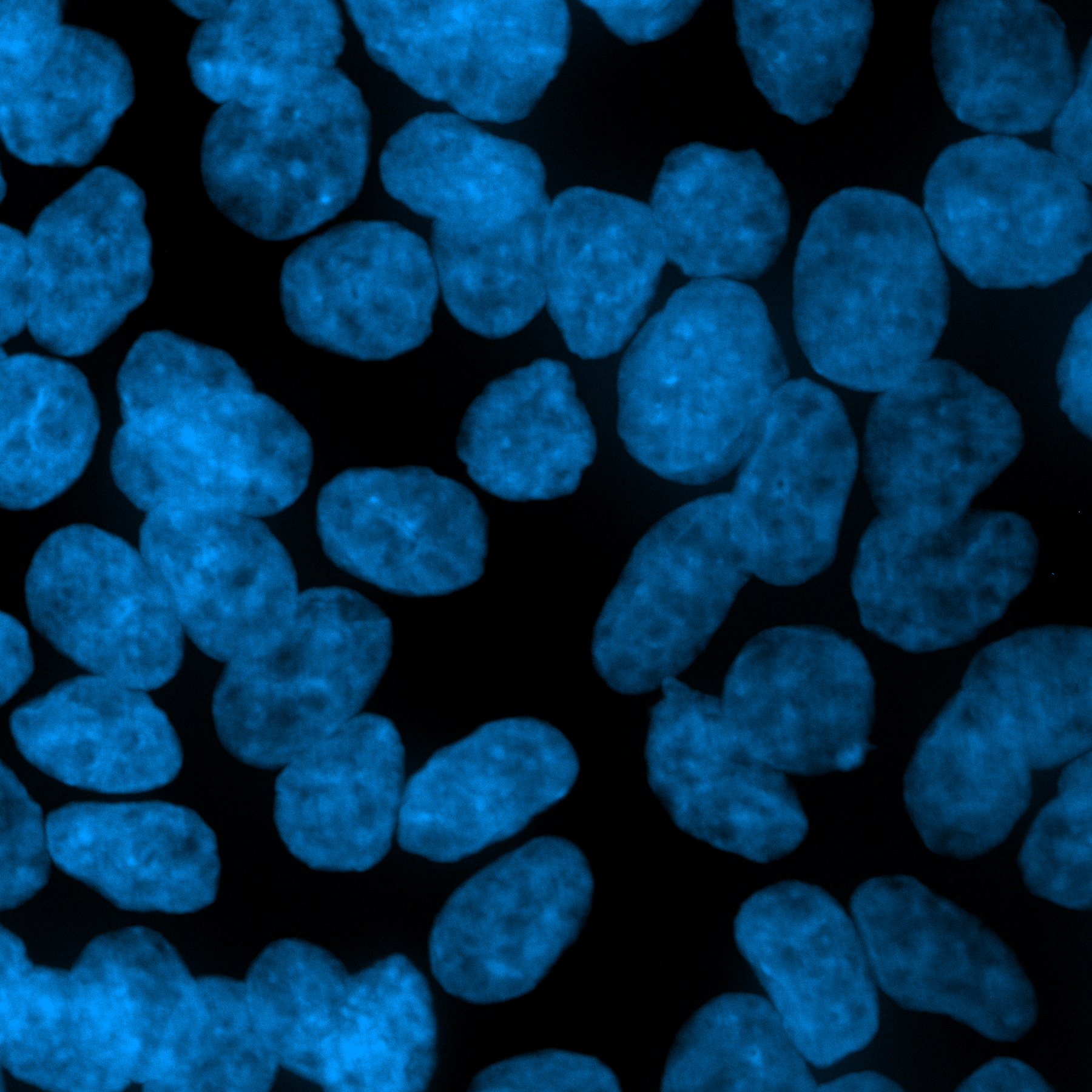

Supplement: Supplementary file 13 — Source data Figure EV1 [file 44318_2024_337_MOESM13_ESM.zip › 07_Figure_EV1/A/Imaging/HAP1-MMEJ/HAP1-MMEJ_DAPI.tif]

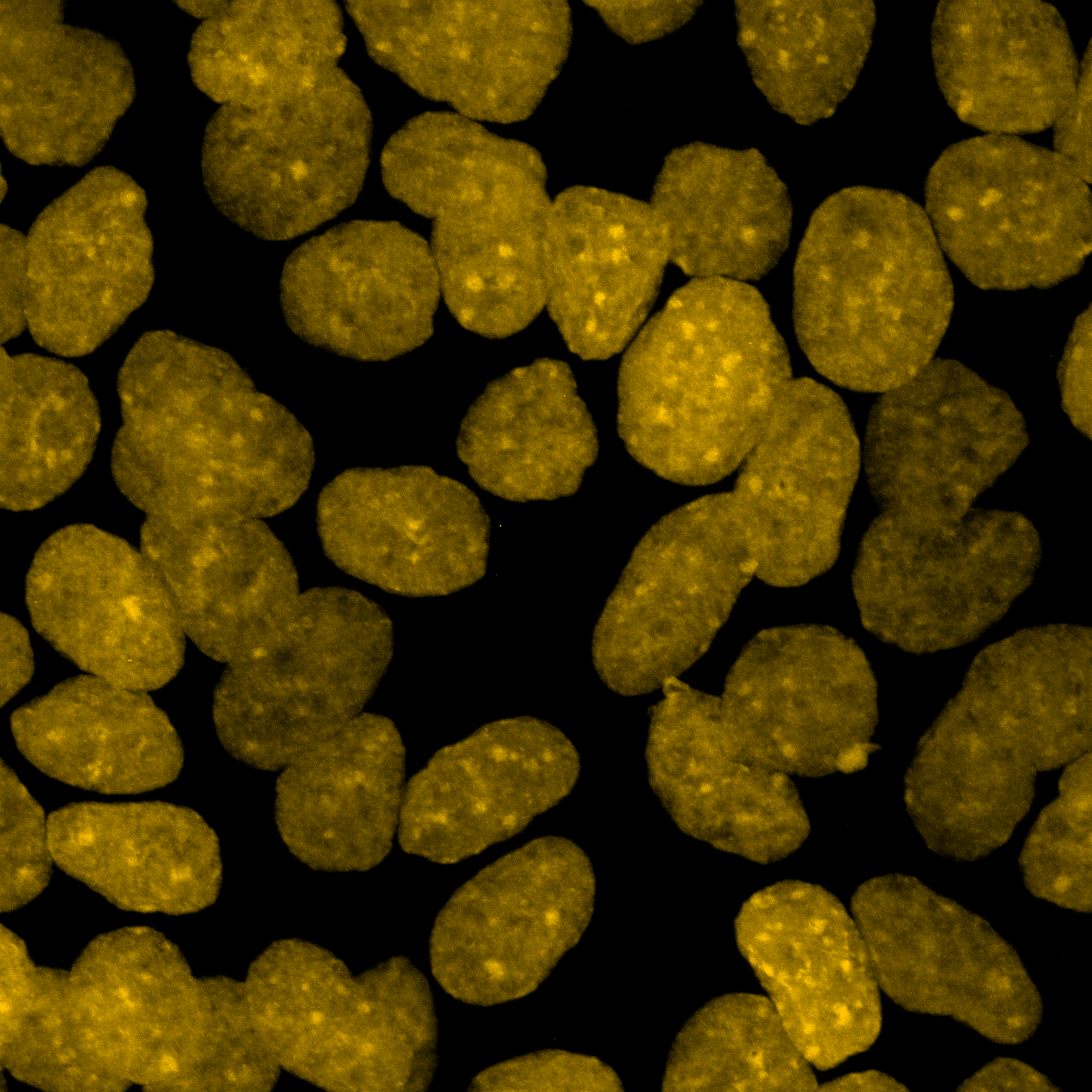

Supplement: Supplementary file 13 — Source data Figure EV1 [file 44318_2024_337_MOESM13_ESM.zip › 07_Figure_EV1/A/Imaging/HAP1-MMEJ/HAP1-MMEJ_GFP.tif]

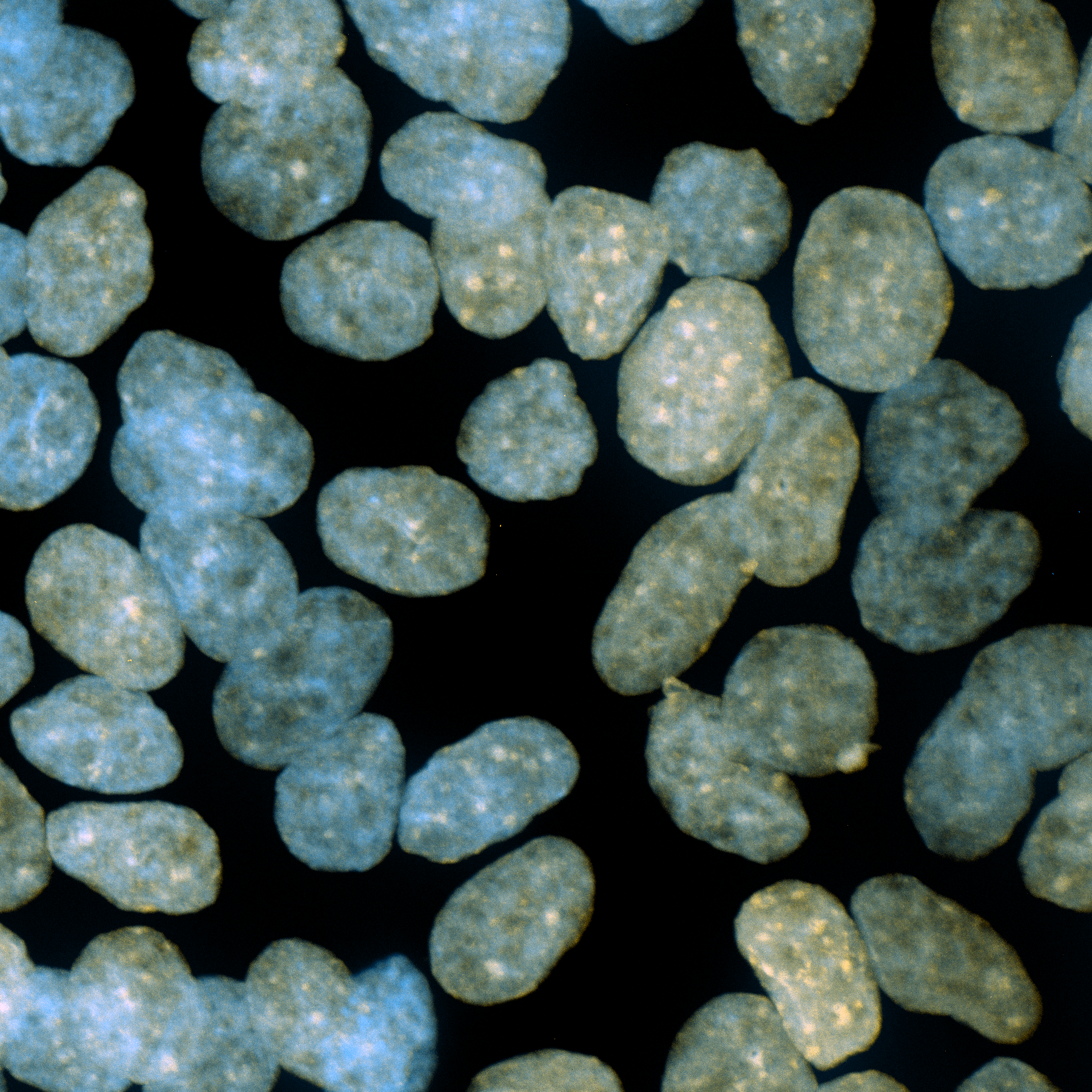

Supplement: Supplementary file 13 — Source data Figure EV1 [file 44318_2024_337_MOESM13_ESM.zip › 07_Figure_EV1/A/Imaging/HAP1-MMEJ/HAP1-MMEJ_Merge_RGB.tif]

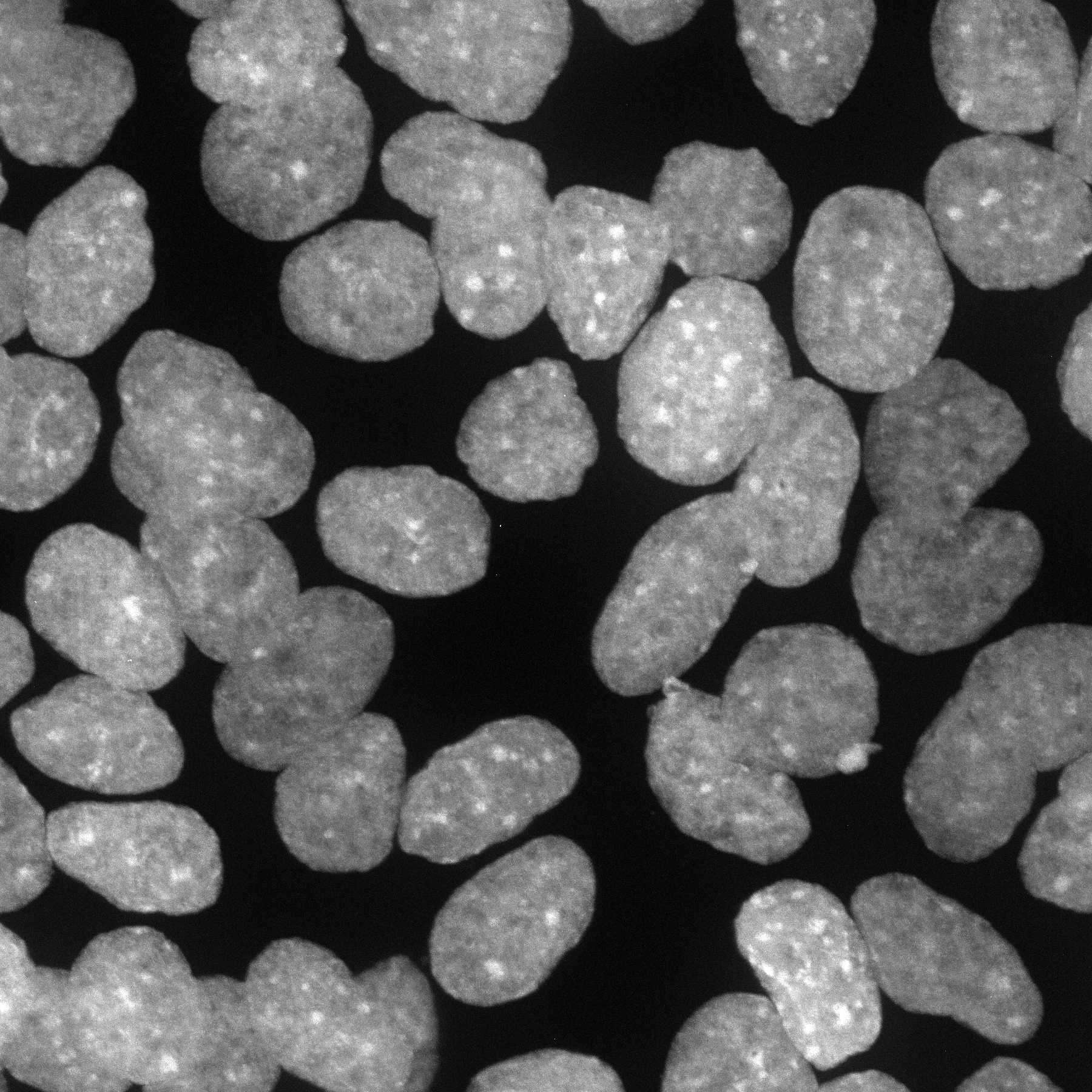

Supplement: Supplementary file 13 — Source data Figure EV1 [file 44318_2024_337_MOESM13_ESM.zip › 07_Figure_EV1/A/Imaging/HAP1-MMEJ/_FULL-RANGE-HAP1-MMEJ.tif]

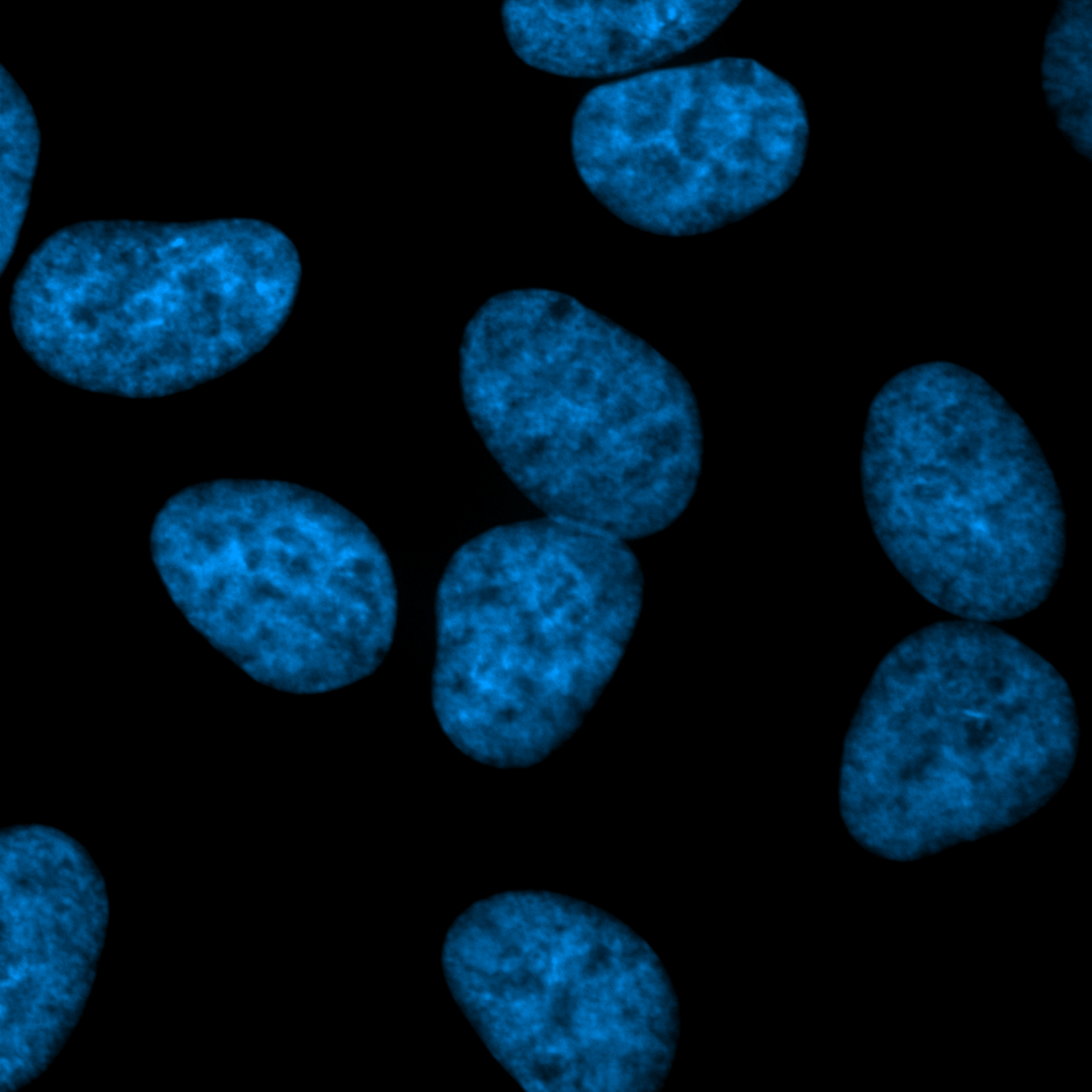

Supplement: Supplementary file 13 — Source data Figure EV1 [file 44318_2024_337_MOESM13_ESM.zip › 07_Figure_EV1/B/Images/ARPE-CTRL/ARPE-CTRL_DAPI.tif]

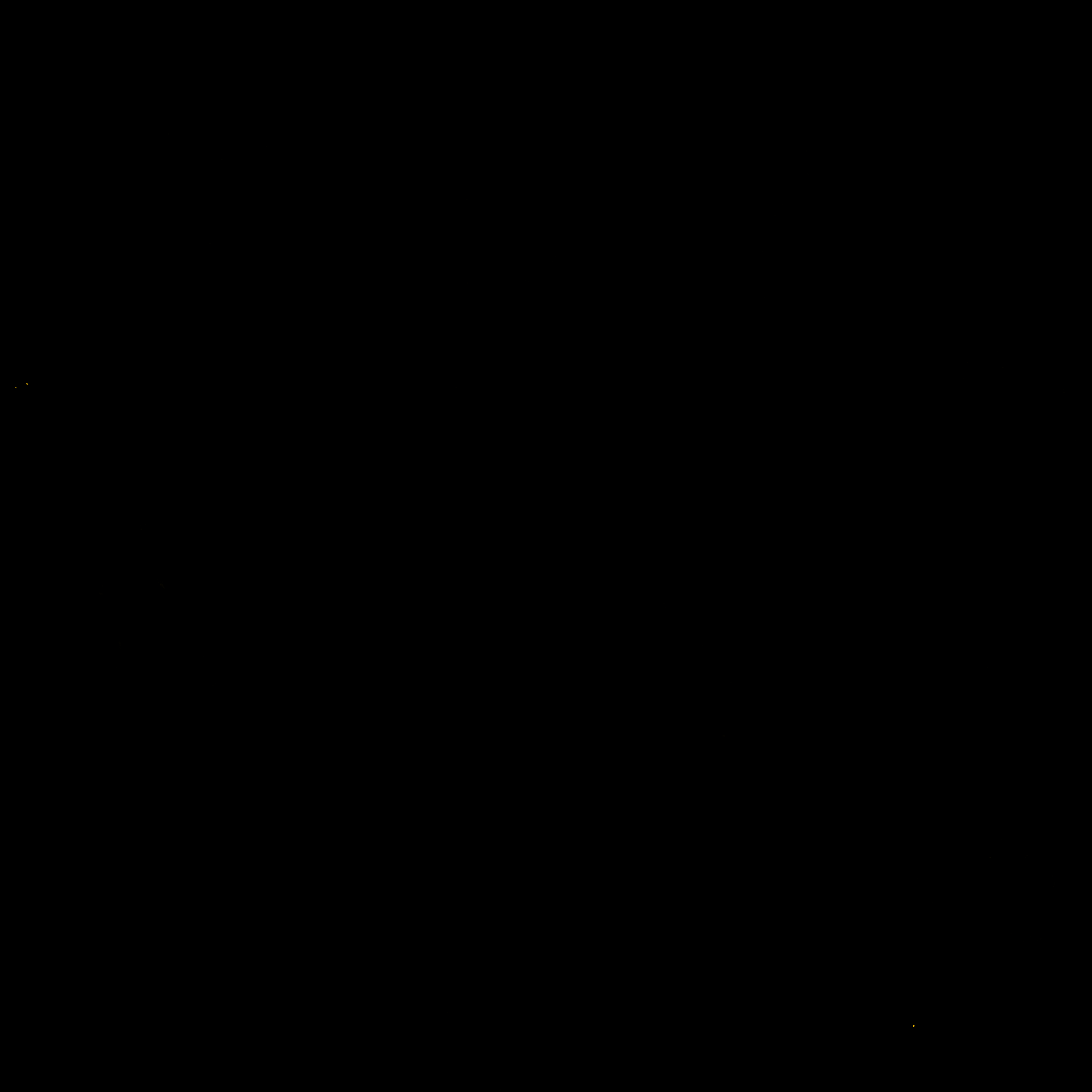

Supplement: Supplementary file 13 — Source data Figure EV1 [file 44318_2024_337_MOESM13_ESM.zip › 07_Figure_EV1/B/Images/ARPE-CTRL/ARPE-CTRL_GFP.tif]

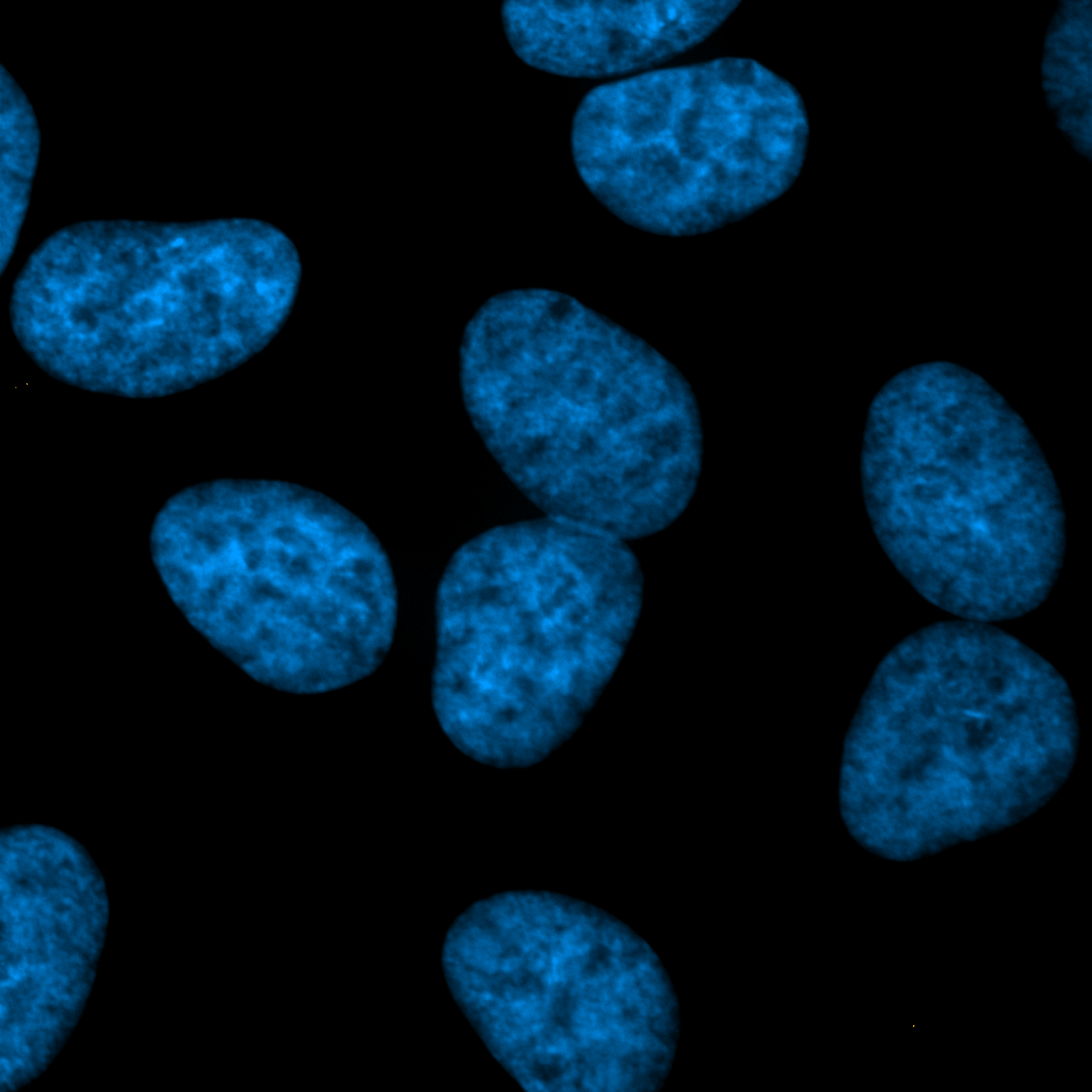

Supplement: Supplementary file 13 — Source data Figure EV1 [file 44318_2024_337_MOESM13_ESM.zip › 07_Figure_EV1/B/Images/ARPE-CTRL/ARPE-CTRL_Merge.tif]

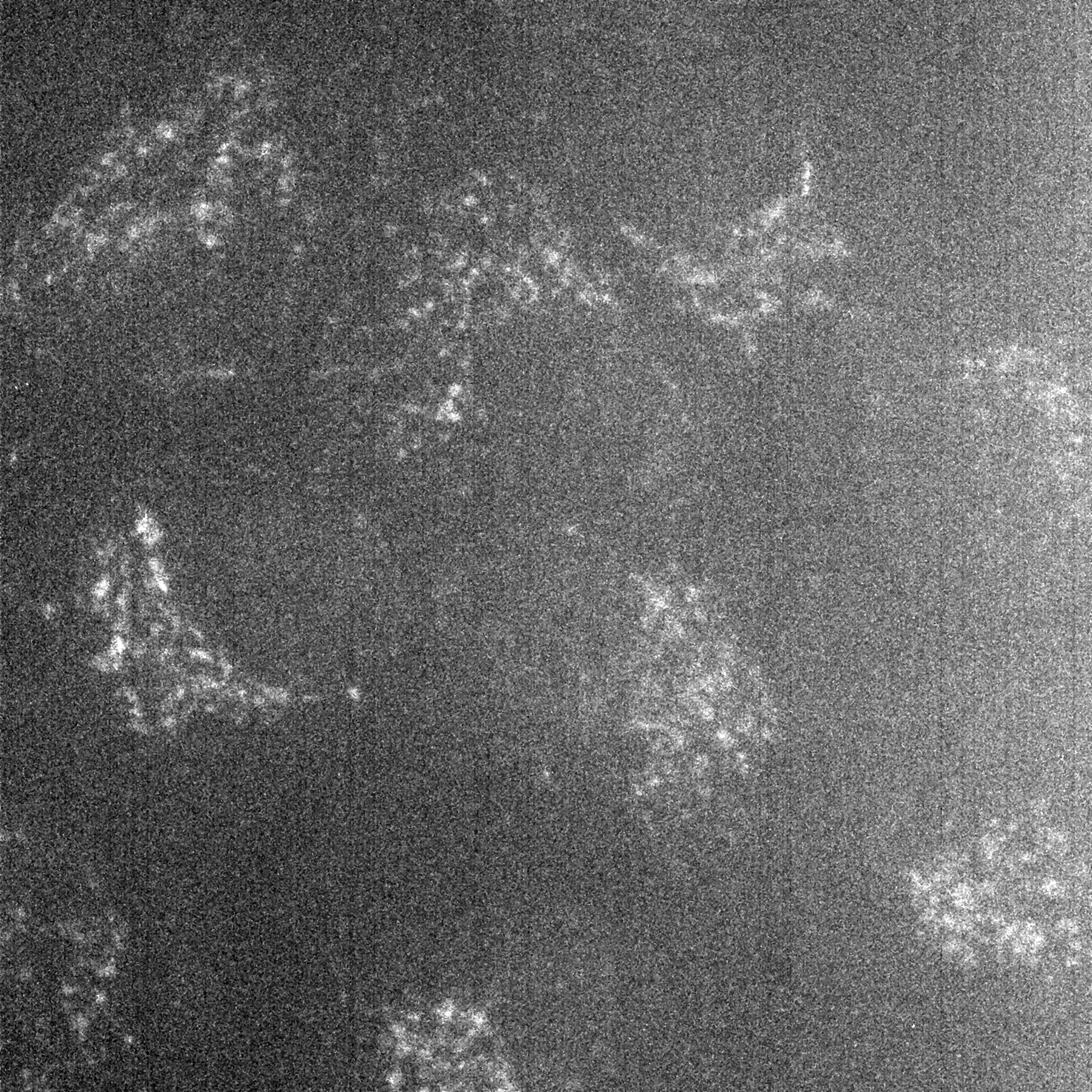

Supplement: Supplementary file 13 — Source data Figure EV1 [file 44318_2024_337_MOESM13_ESM.zip › 07_Figure_EV1/B/Images/ARPE-CTRL/_FULL-RANGE-ARPE-CTRL.tif]

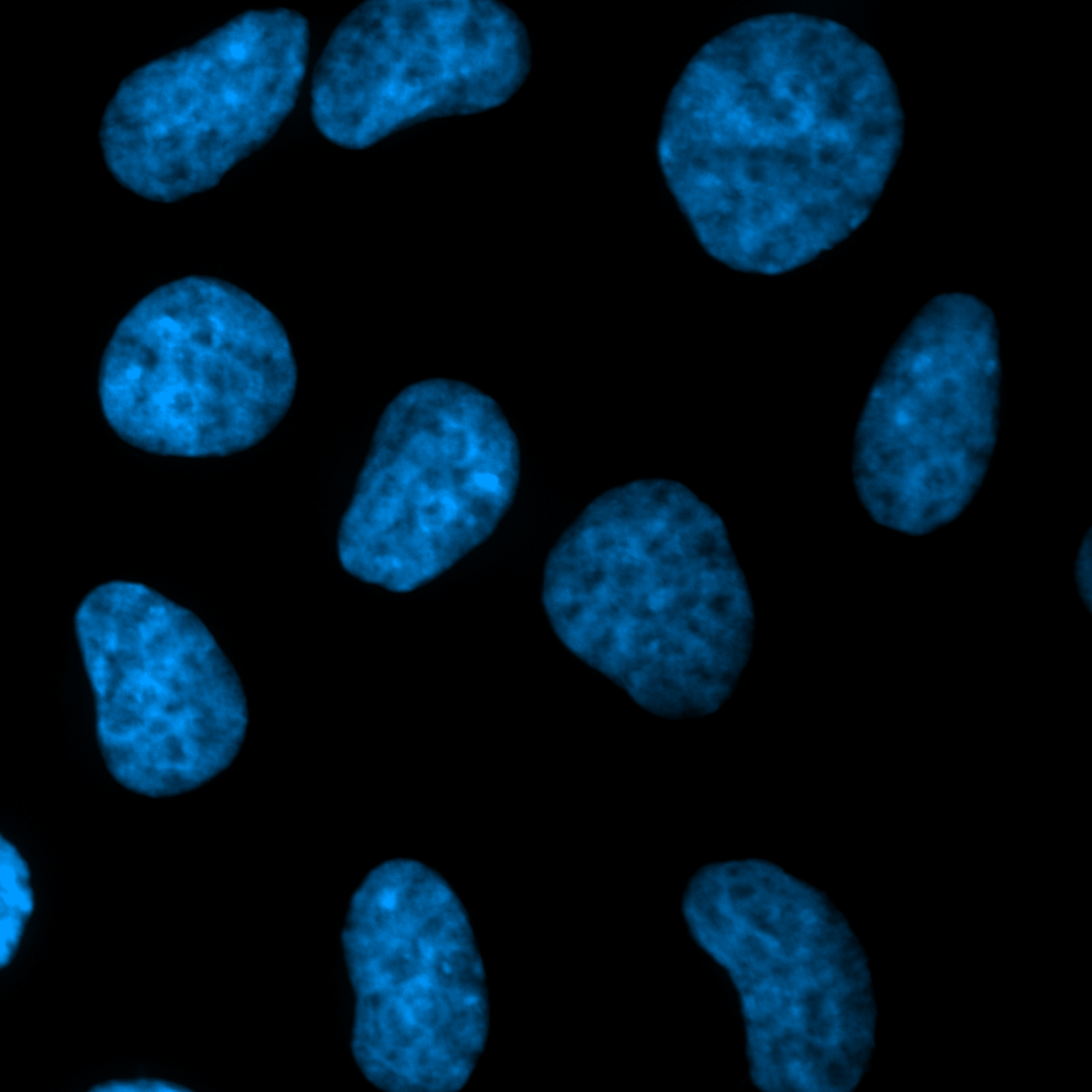

Supplement: Supplementary file 13 — Source data Figure EV1 [file 44318_2024_337_MOESM13_ESM.zip › 07_Figure_EV1/B/Images/ARPE-HDR/ARPE-HDR_DAPI.tif]

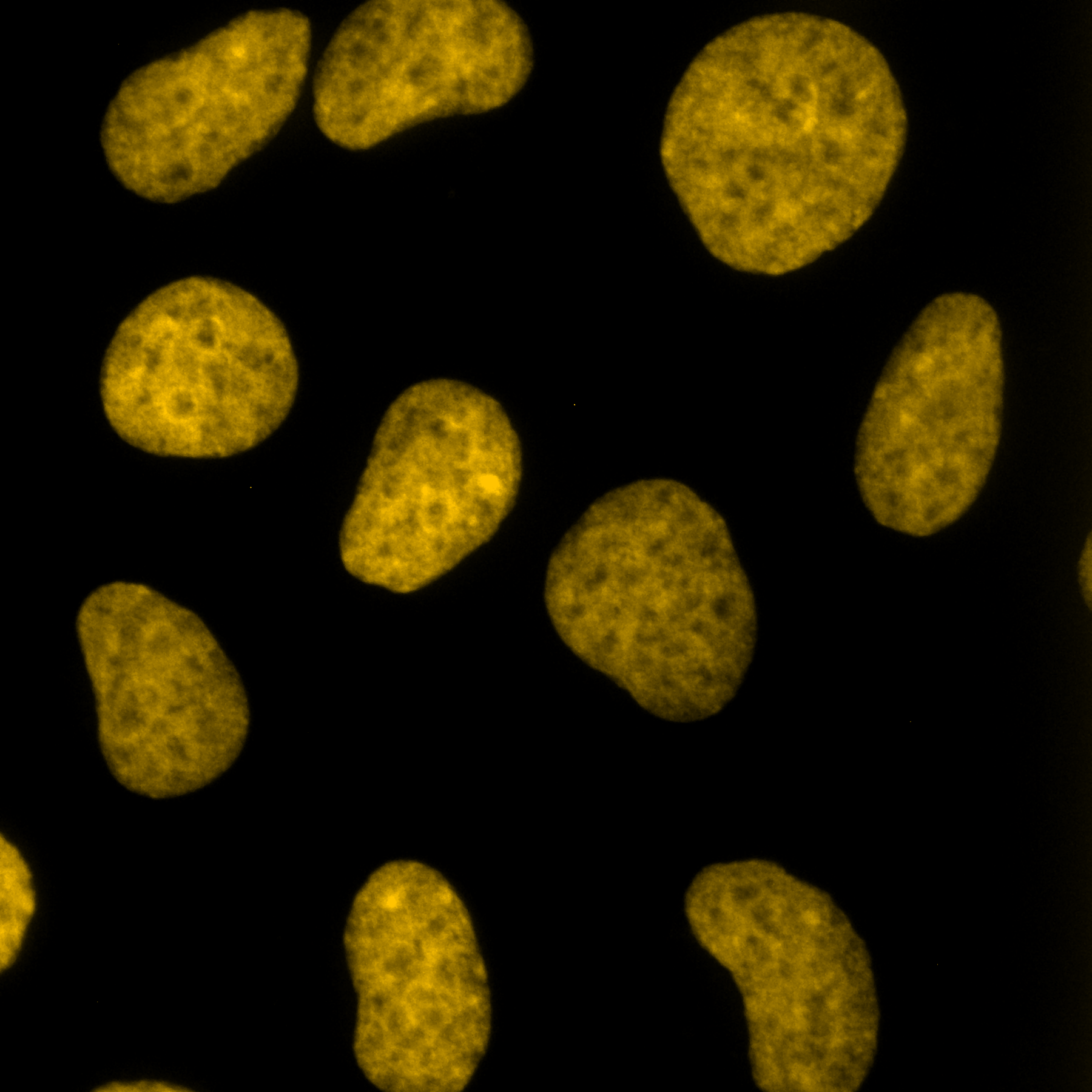

Supplement: Supplementary file 13 — Source data Figure EV1 [file 44318_2024_337_MOESM13_ESM.zip › 07_Figure_EV1/B/Images/ARPE-HDR/ARPE-HDR_GFP.tif]

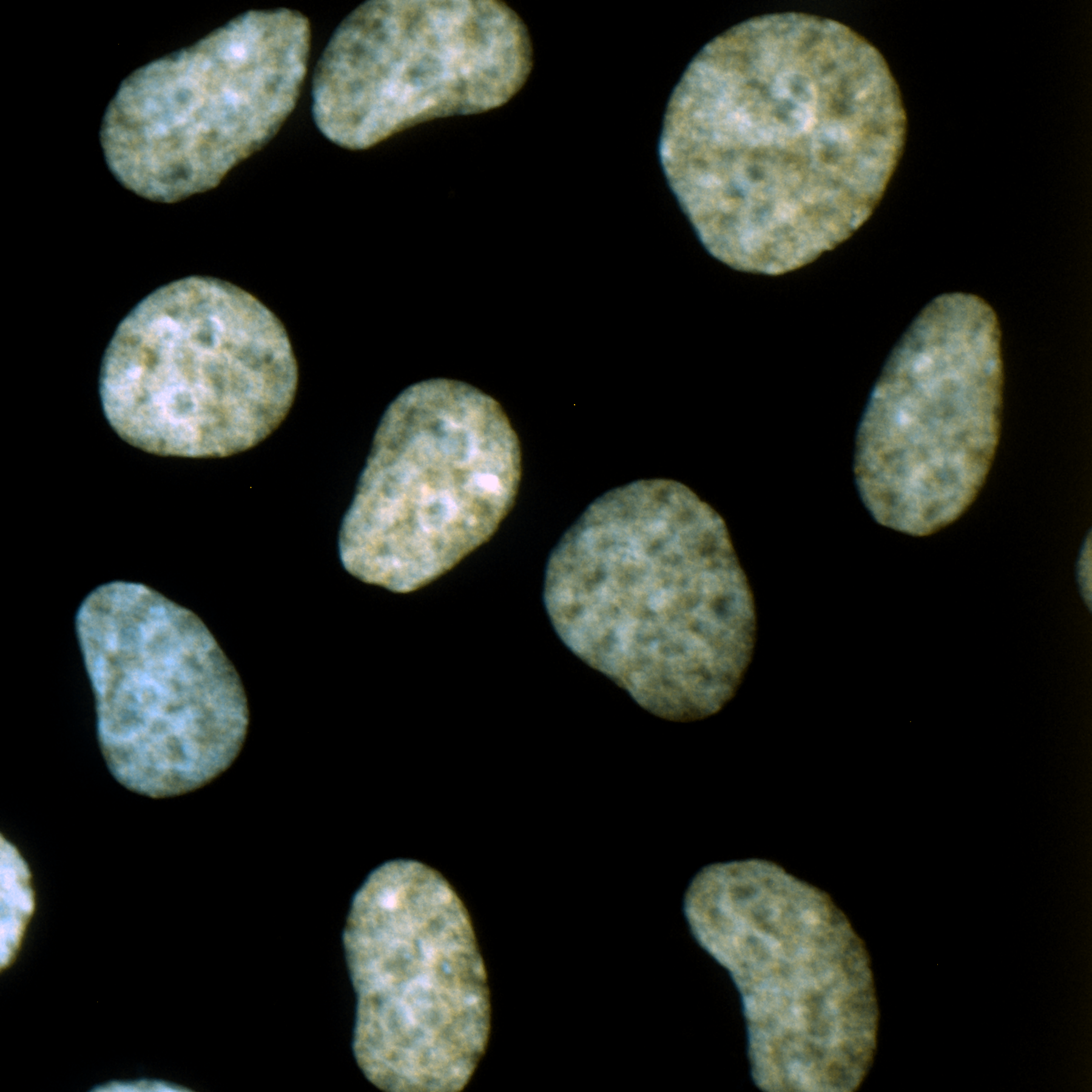

Supplement: Supplementary file 13 — Source data Figure EV1 [file 44318_2024_337_MOESM13_ESM.zip › 07_Figure_EV1/B/Images/ARPE-HDR/ARPE-HDR_Merge.tif]

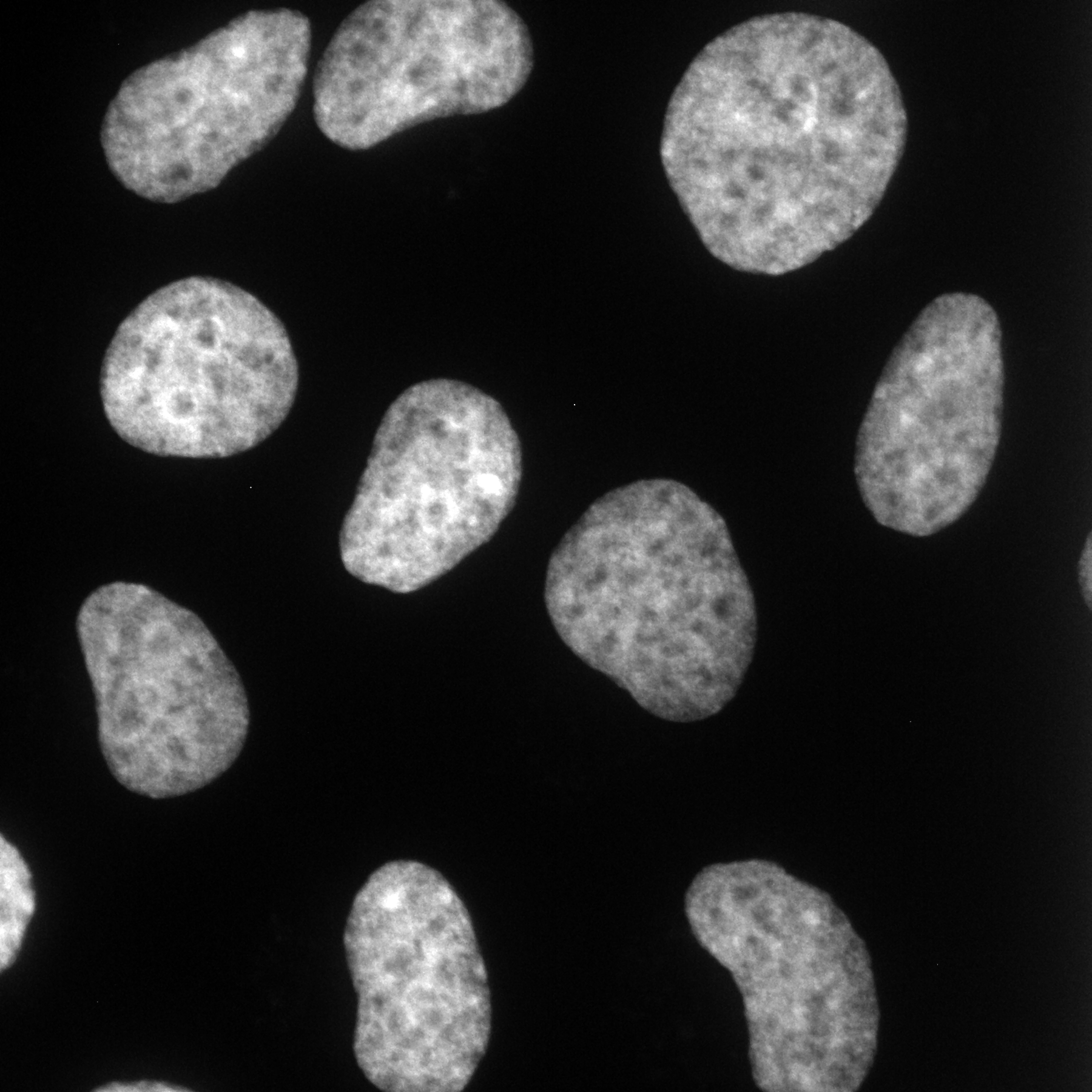

Supplement: Supplementary file 13 — Source data Figure EV1 [file 44318_2024_337_MOESM13_ESM.zip › 07_Figure_EV1/B/Images/ARPE-HDR/_FULL-RANGE-ARPE-HDR.tif]

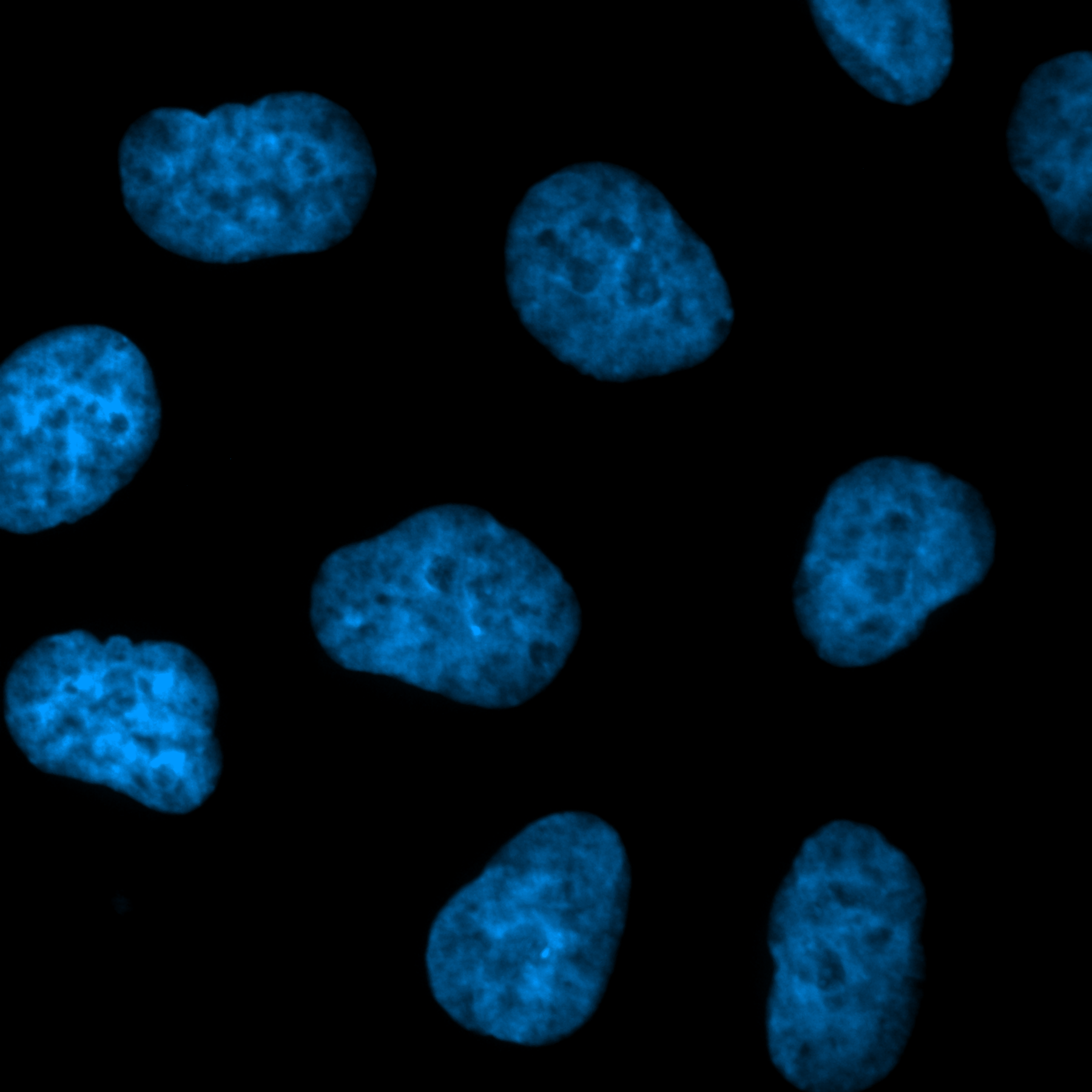

Supplement: Supplementary file 13 — Source data Figure EV1 [file 44318_2024_337_MOESM13_ESM.zip › 07_Figure_EV1/B/Images/ARPE-MMEJ/ARPE-MMEJ_DAPI.tif]

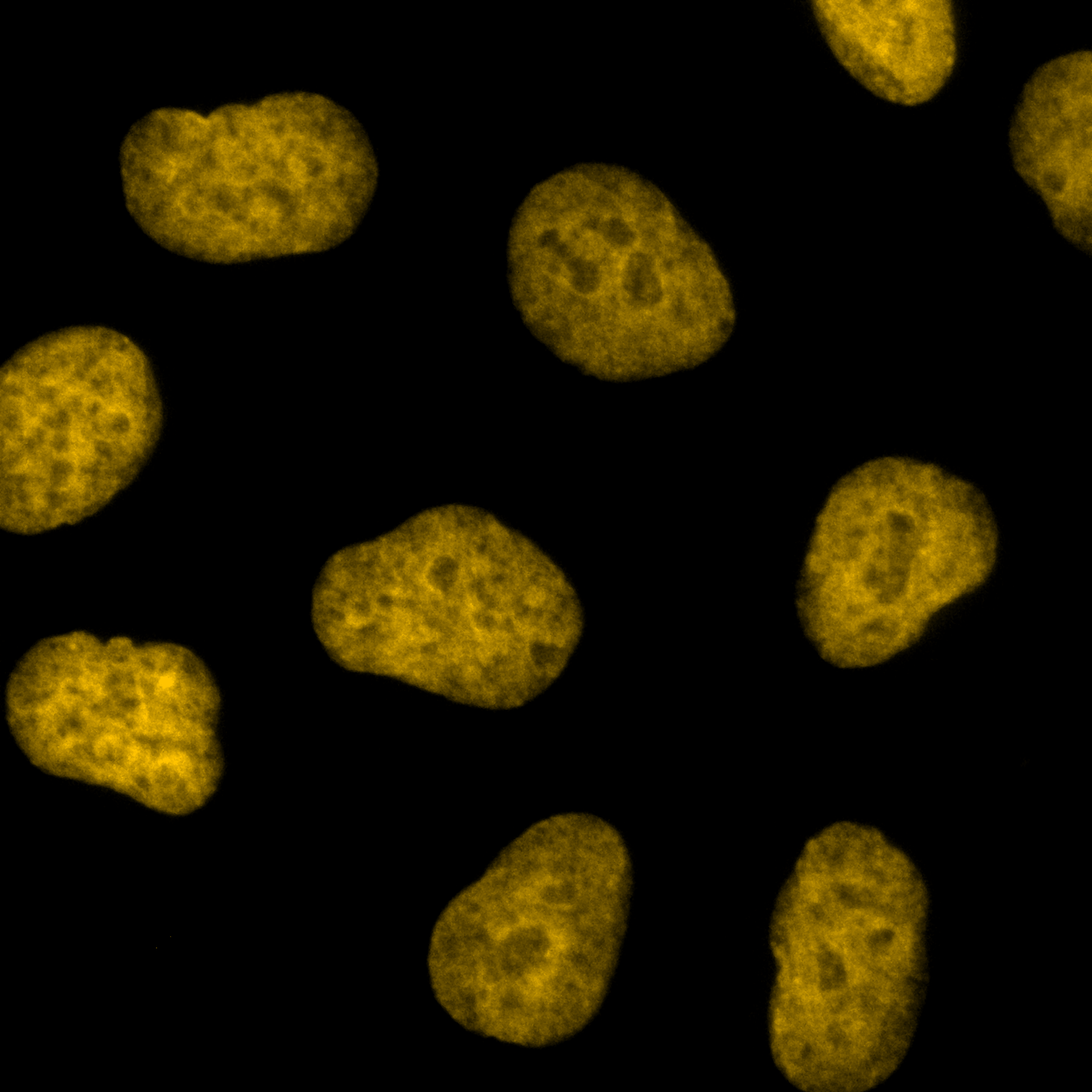

Supplement: Supplementary file 13 — Source data Figure EV1 [file 44318_2024_337_MOESM13_ESM.zip › 07_Figure_EV1/B/Images/ARPE-MMEJ/ARPE-MMEJ_GFP.tif]

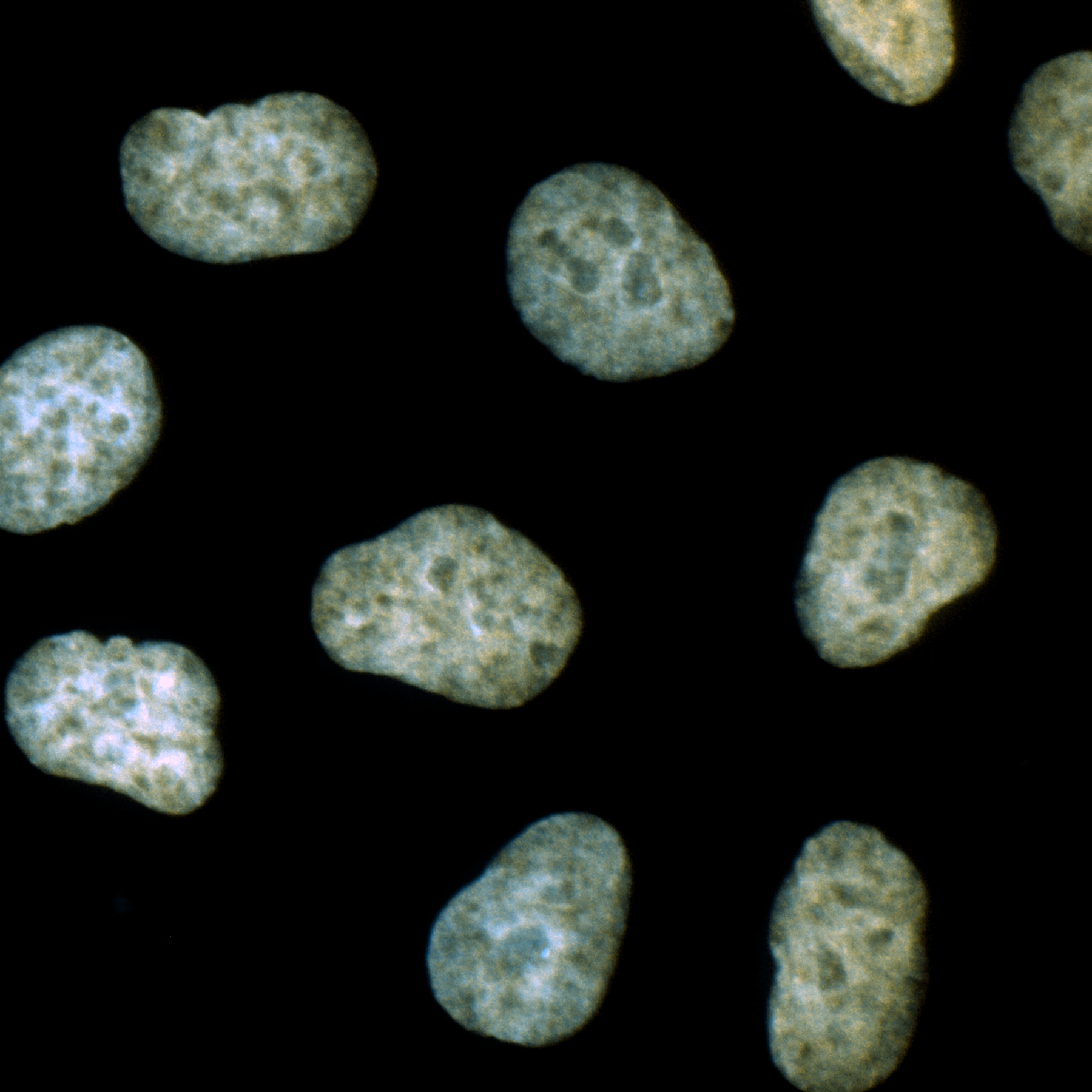

Supplement: Supplementary file 13 — Source data Figure EV1 [file 44318_2024_337_MOESM13_ESM.zip › 07_Figure_EV1/B/Images/ARPE-MMEJ/ARPE-MMEJ_Merge.tif]

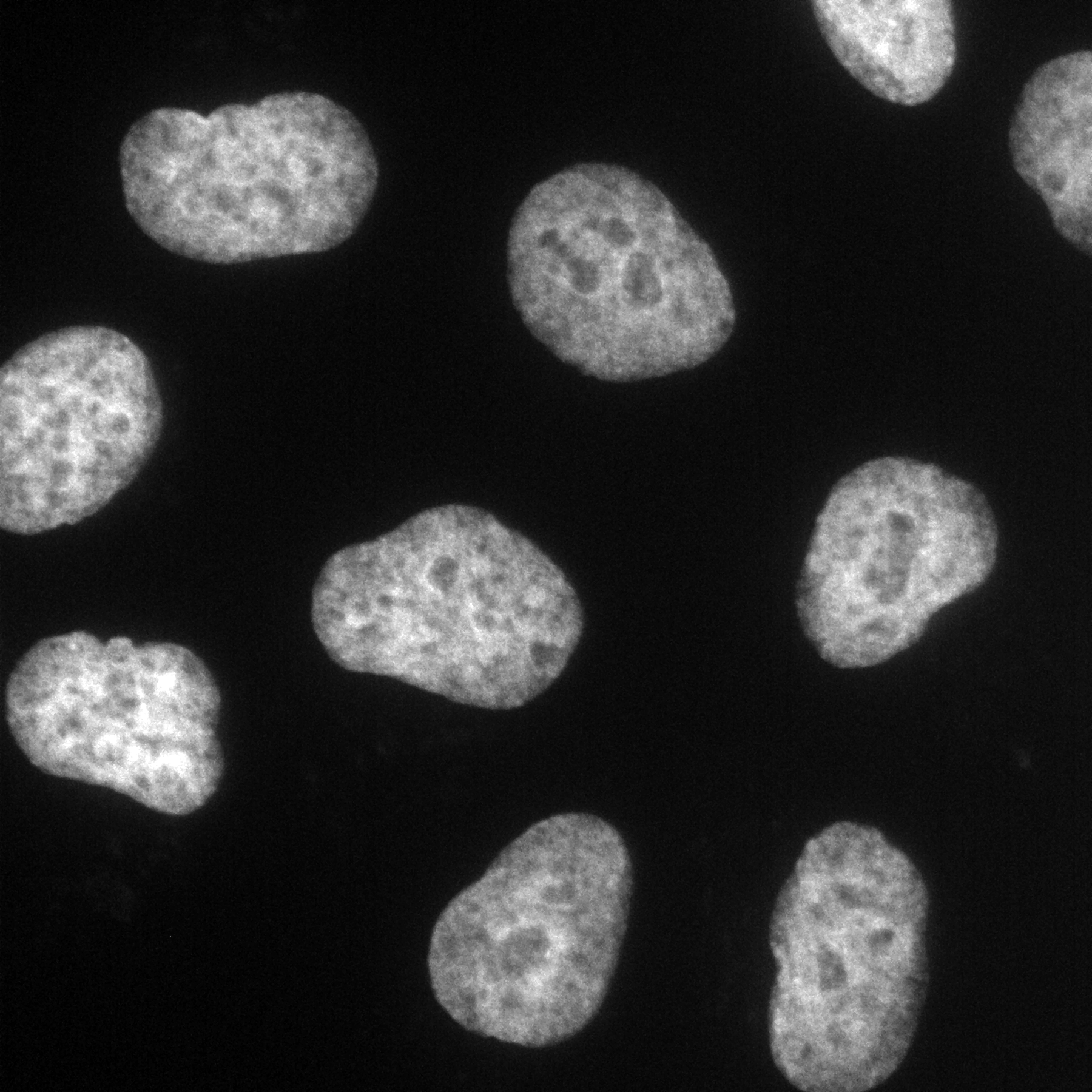

Supplement: Supplementary file 13 — Source data Figure EV1 [file 44318_2024_337_MOESM13_ESM.zip › 07_Figure_EV1/B/Images/ARPE-MMEJ/_FULL-RANGE-ARPE-MMEJ.tif]

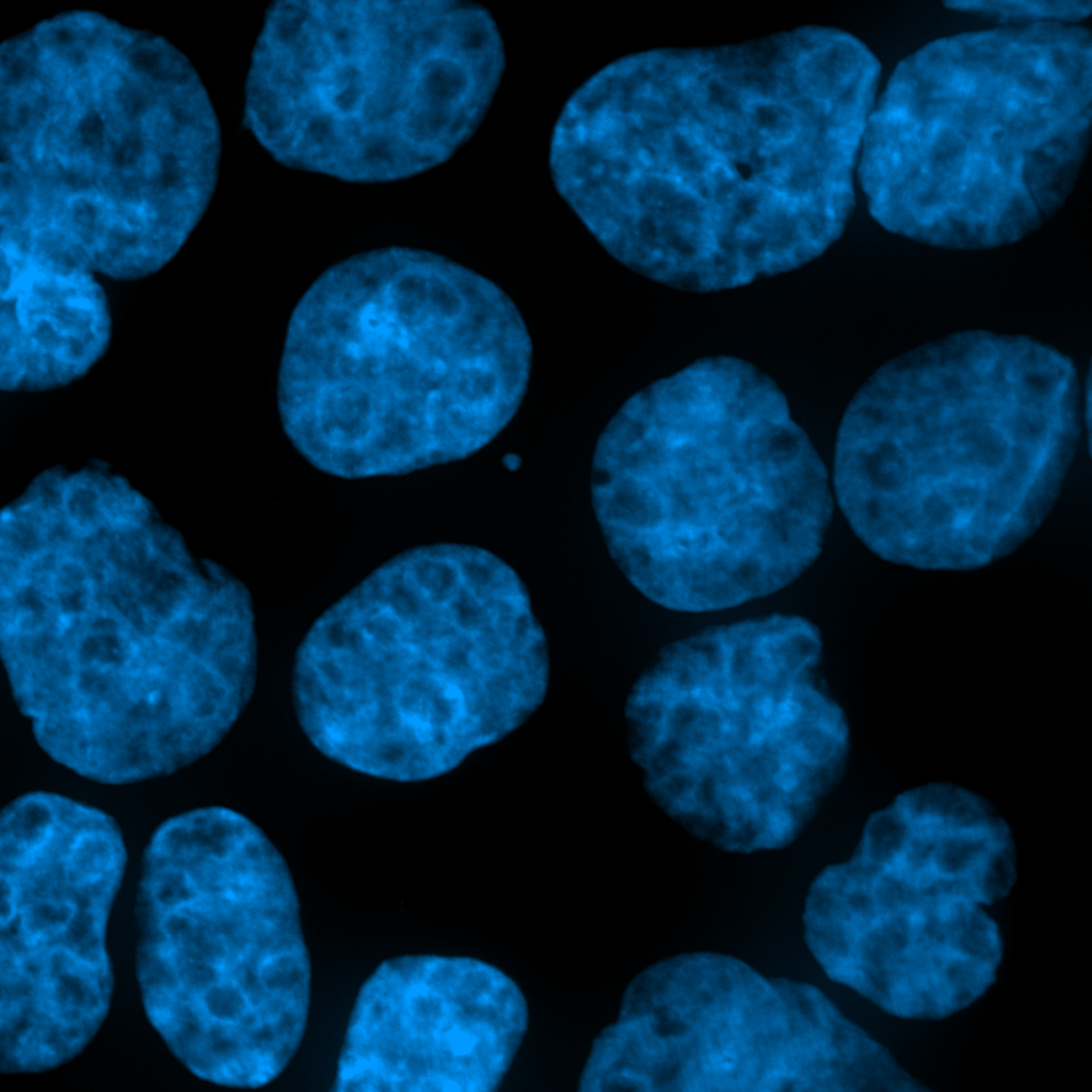

Supplement: Supplementary file 13 — Source data Figure EV1 [file 44318_2024_337_MOESM13_ESM.zip › 07_Figure_EV1/C/Imaging/U2OS-CTRL/U2OS-CTRL_U2OS-CTRL_RGB.tif]

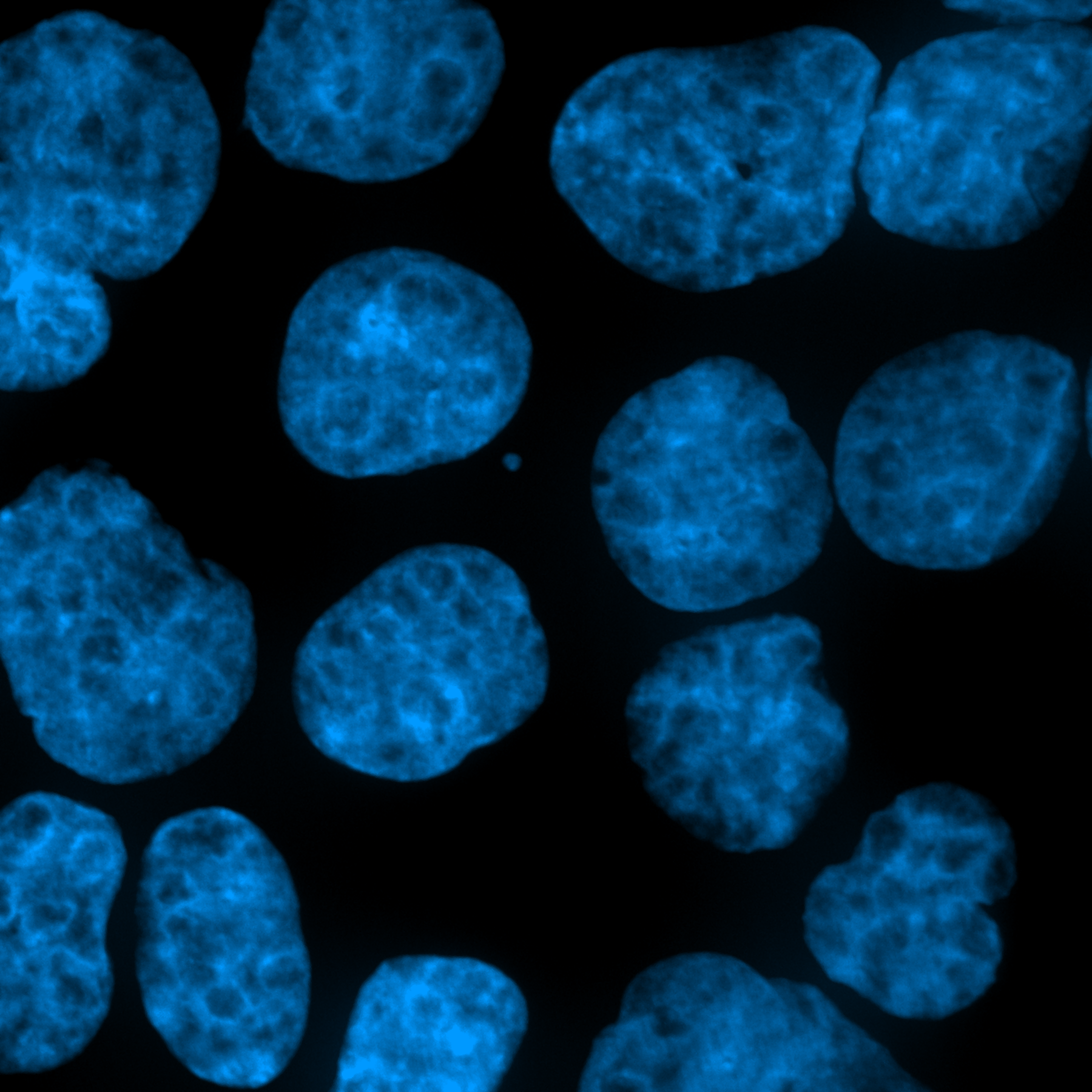

Supplement: Supplementary file 13 — Source data Figure EV1 [file 44318_2024_337_MOESM13_ESM.zip › 07_Figure_EV1/C/Imaging/U2OS-CTRL/U2OS-CTRL_U2OS-CTRL_RGB_QT DAPI.tif]

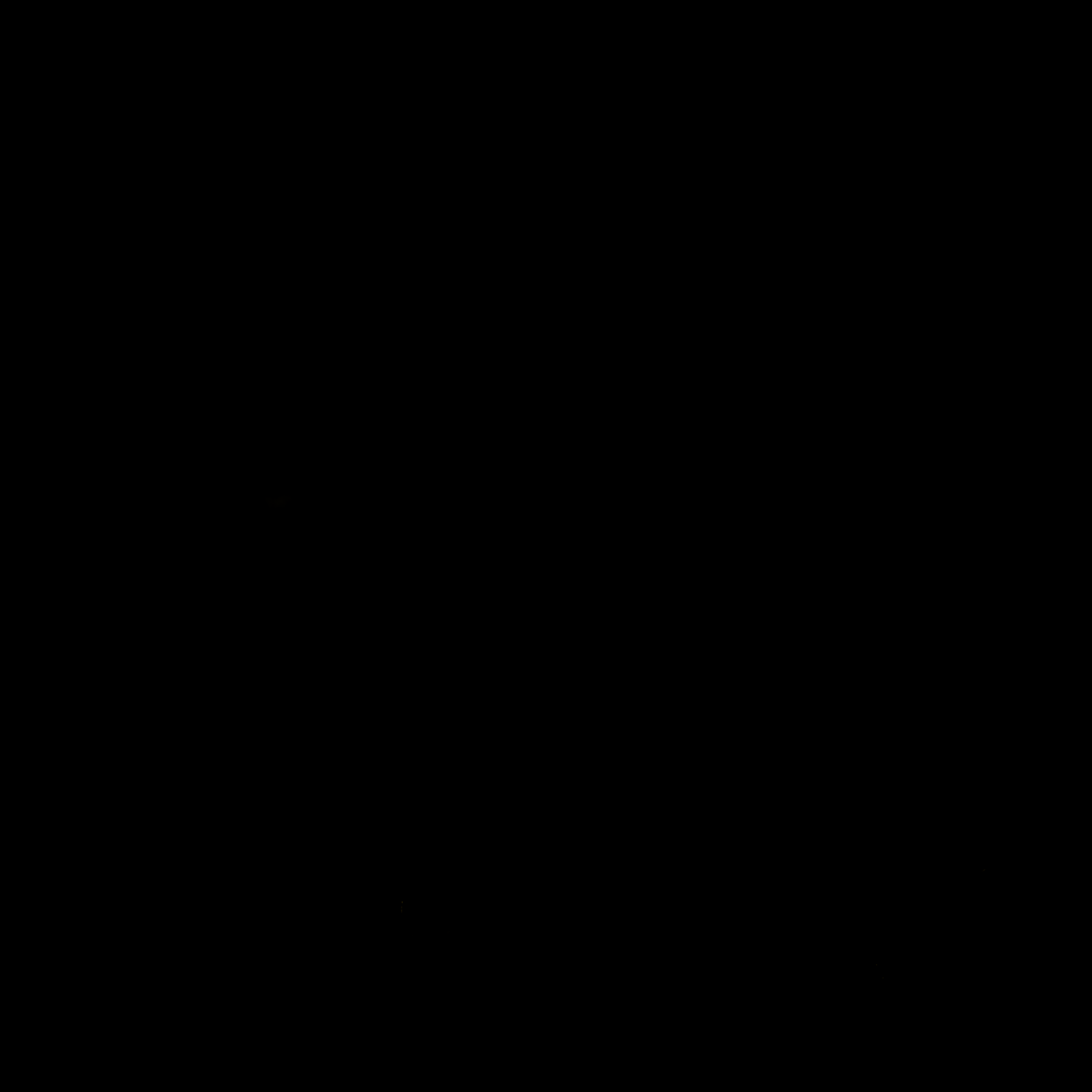

Supplement: Supplementary file 13 — Source data Figure EV1 [file 44318_2024_337_MOESM13_ESM.zip › 07_Figure_EV1/C/Imaging/U2OS-CTRL/U2OS-CTRL_U2OS-CTRL_RGB_QT GFP.tif]

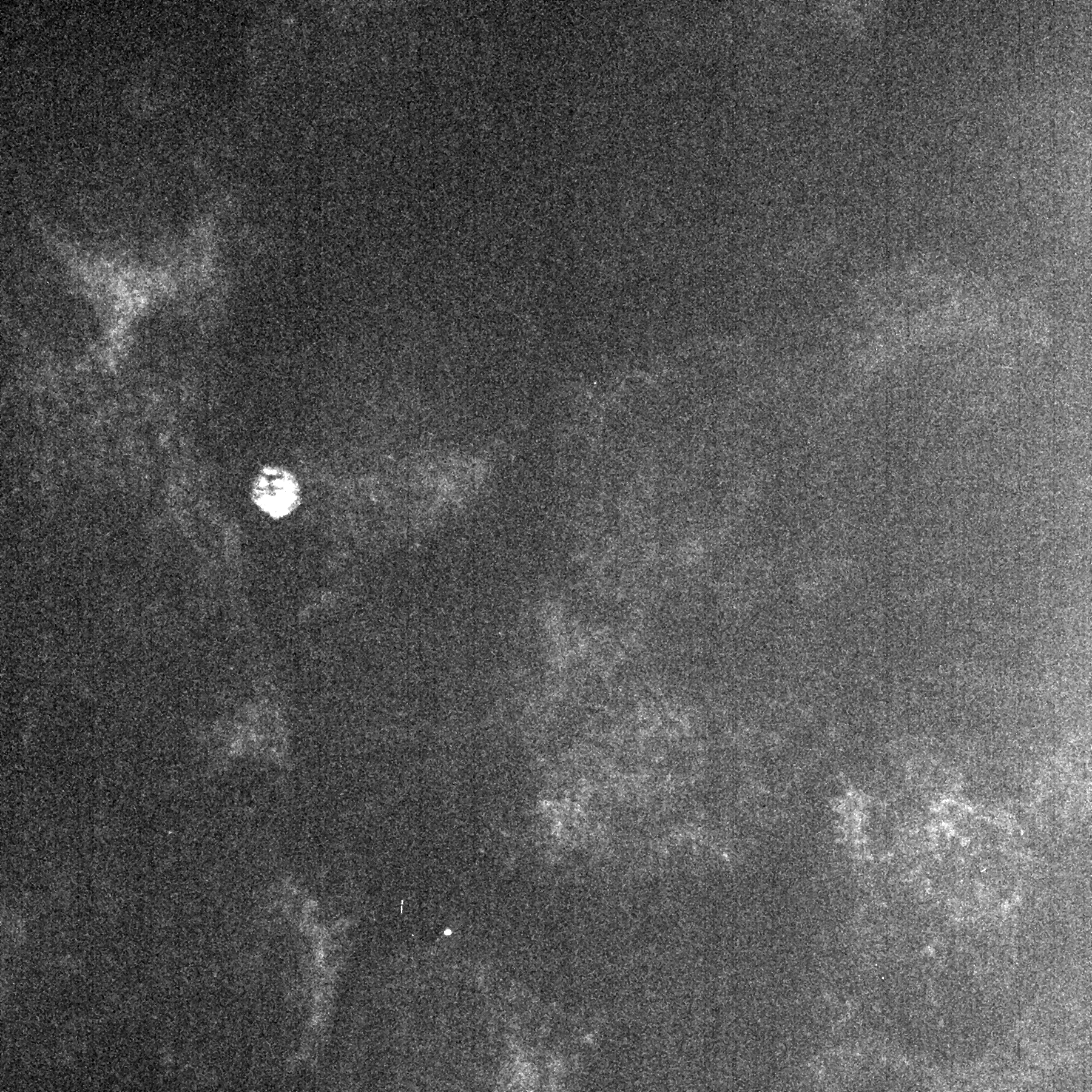

Supplement: Supplementary file 13 — Source data Figure EV1 [file 44318_2024_337_MOESM13_ESM.zip › 07_Figure_EV1/C/Imaging/U2OS-CTRL/_FULL-RANGE-U2OS-CTRL.tif]

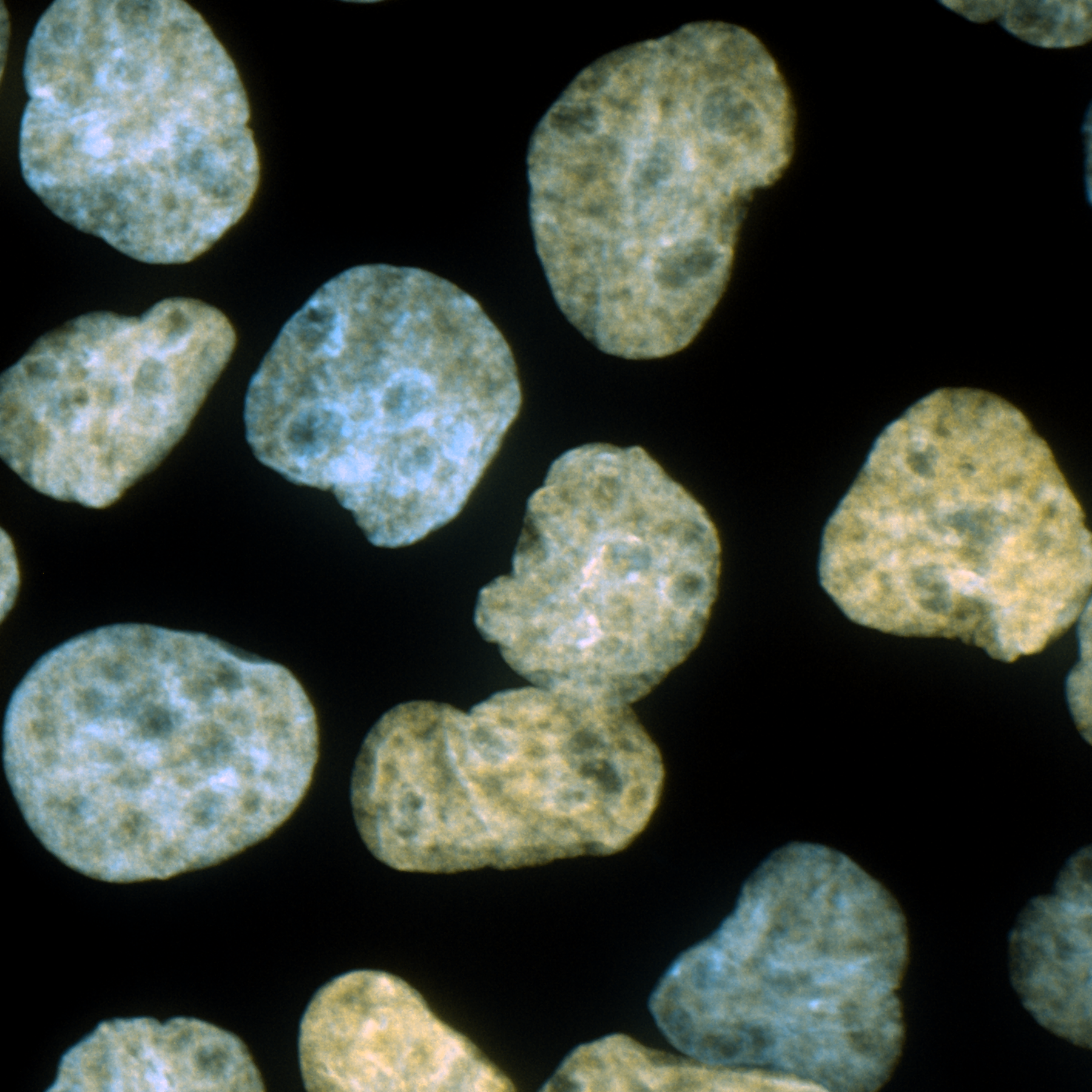

Supplement: Supplementary file 13 — Source data Figure EV1 [file 44318_2024_337_MOESM13_ESM.zip › 07_Figure_EV1/C/Imaging/U2OS-HDR/U2OS-HDR_U2OS-HDR_RGB.tif]

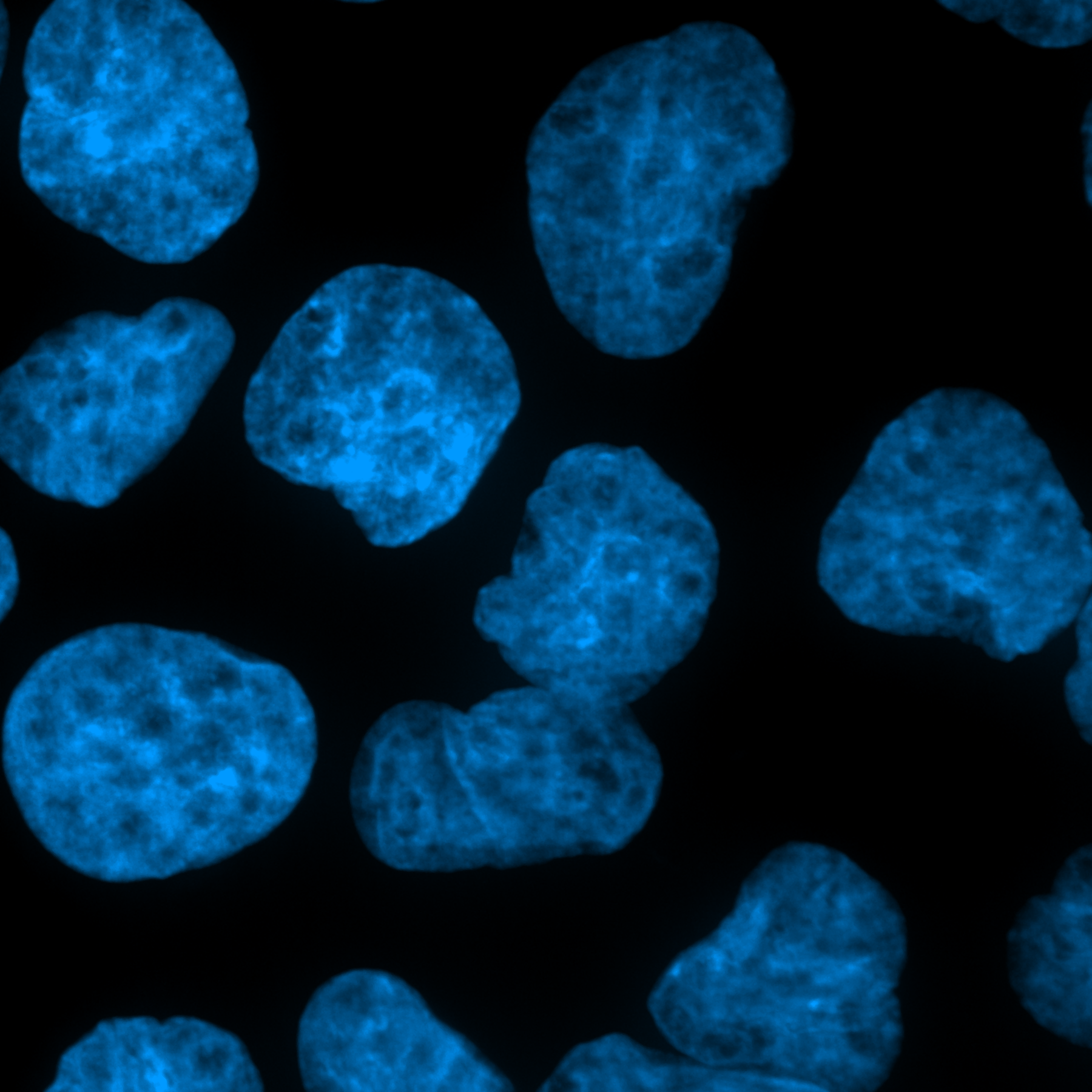

Supplement: Supplementary file 13 — Source data Figure EV1 [file 44318_2024_337_MOESM13_ESM.zip › 07_Figure_EV1/C/Imaging/U2OS-HDR/U2OS-HDR_U2OS-HDR_RGB_QT DAPI.tif]

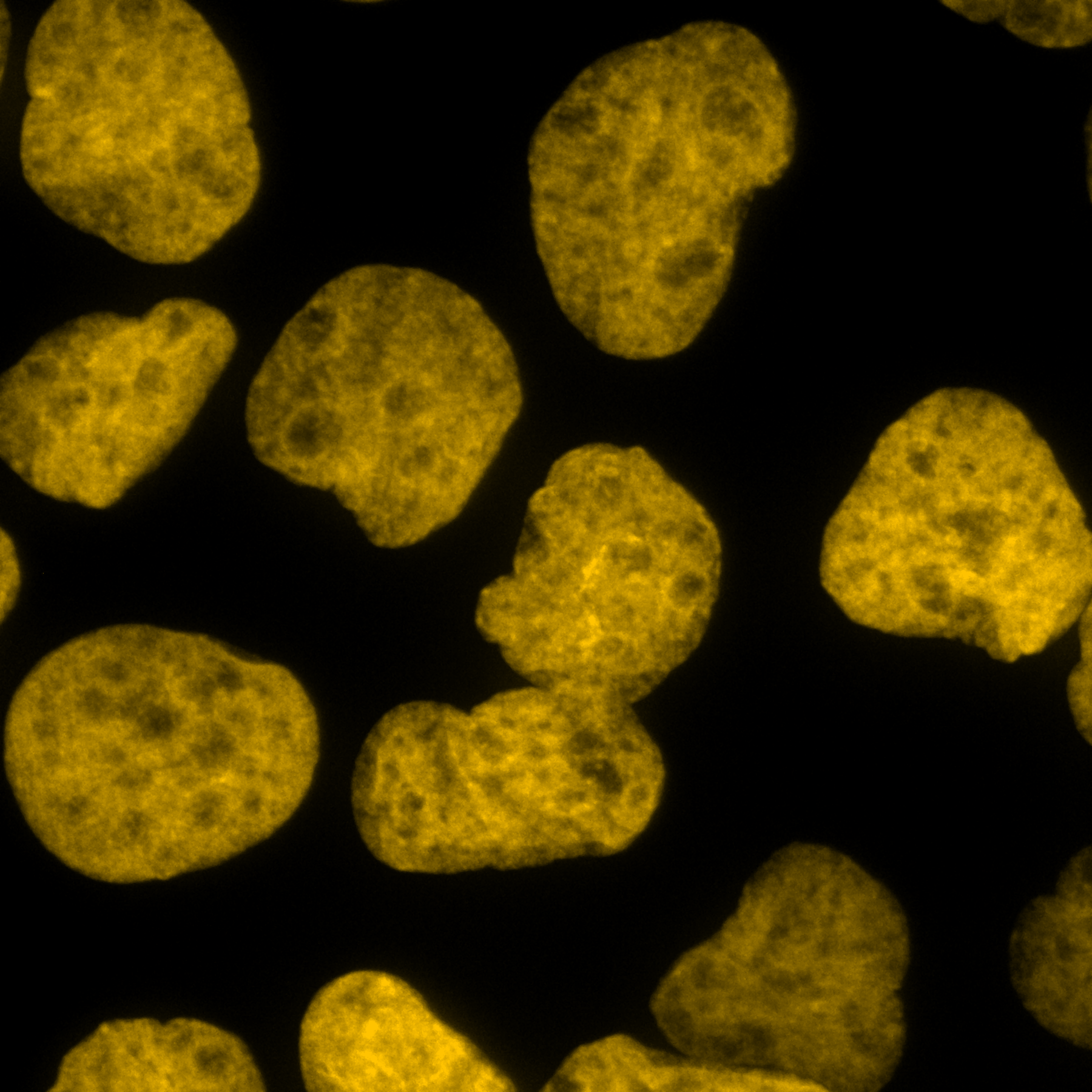

Supplement: Supplementary file 13 — Source data Figure EV1 [file 44318_2024_337_MOESM13_ESM.zip › 07_Figure_EV1/C/Imaging/U2OS-HDR/U2OS-HDR_U2OS-HDR_RGB_QT GFP.tif]

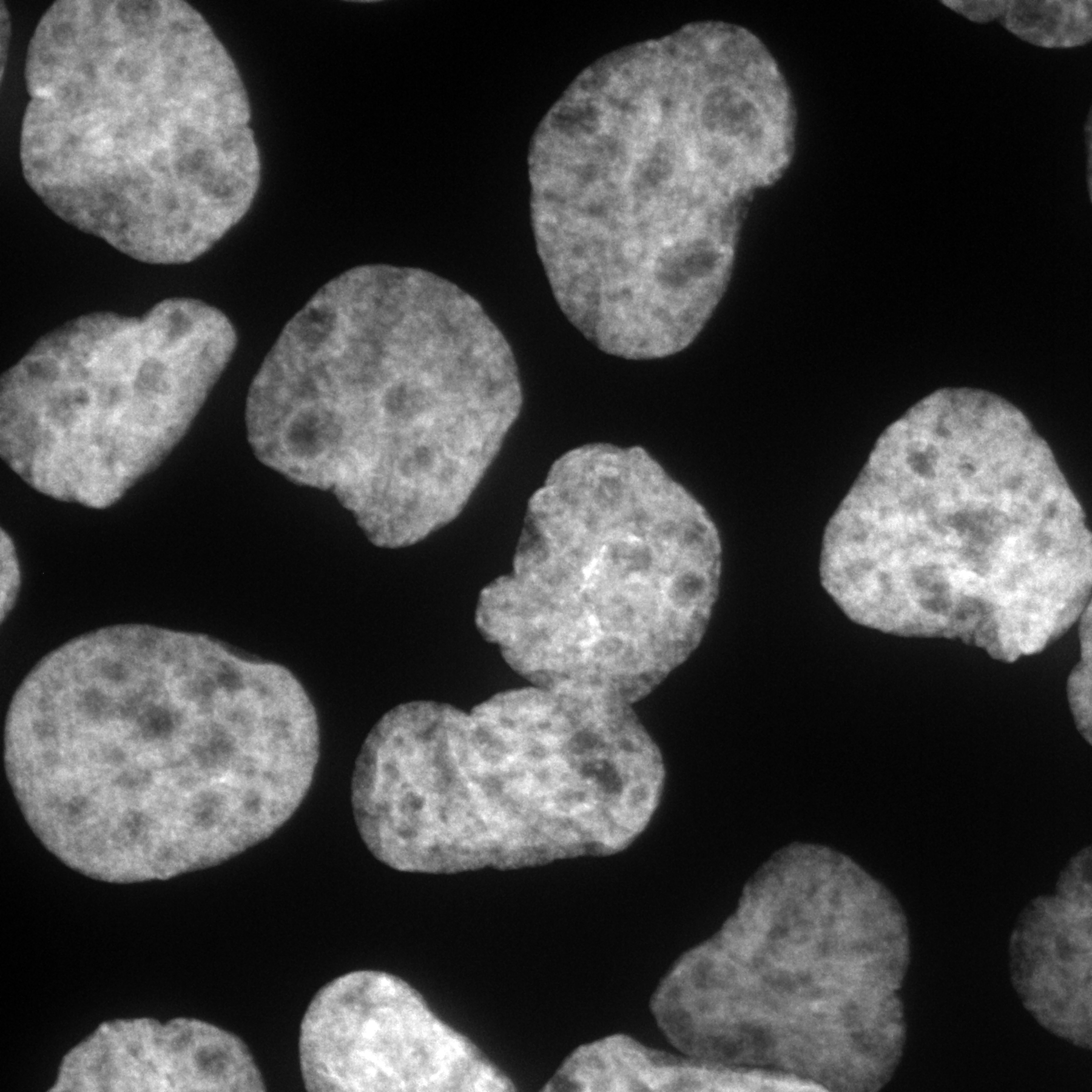

Supplement: Supplementary file 13 — Source data Figure EV1 [file 44318_2024_337_MOESM13_ESM.zip › 07_Figure_EV1/C/Imaging/U2OS-HDR/_FULL-RANGE-U2OS-HDR.tif]

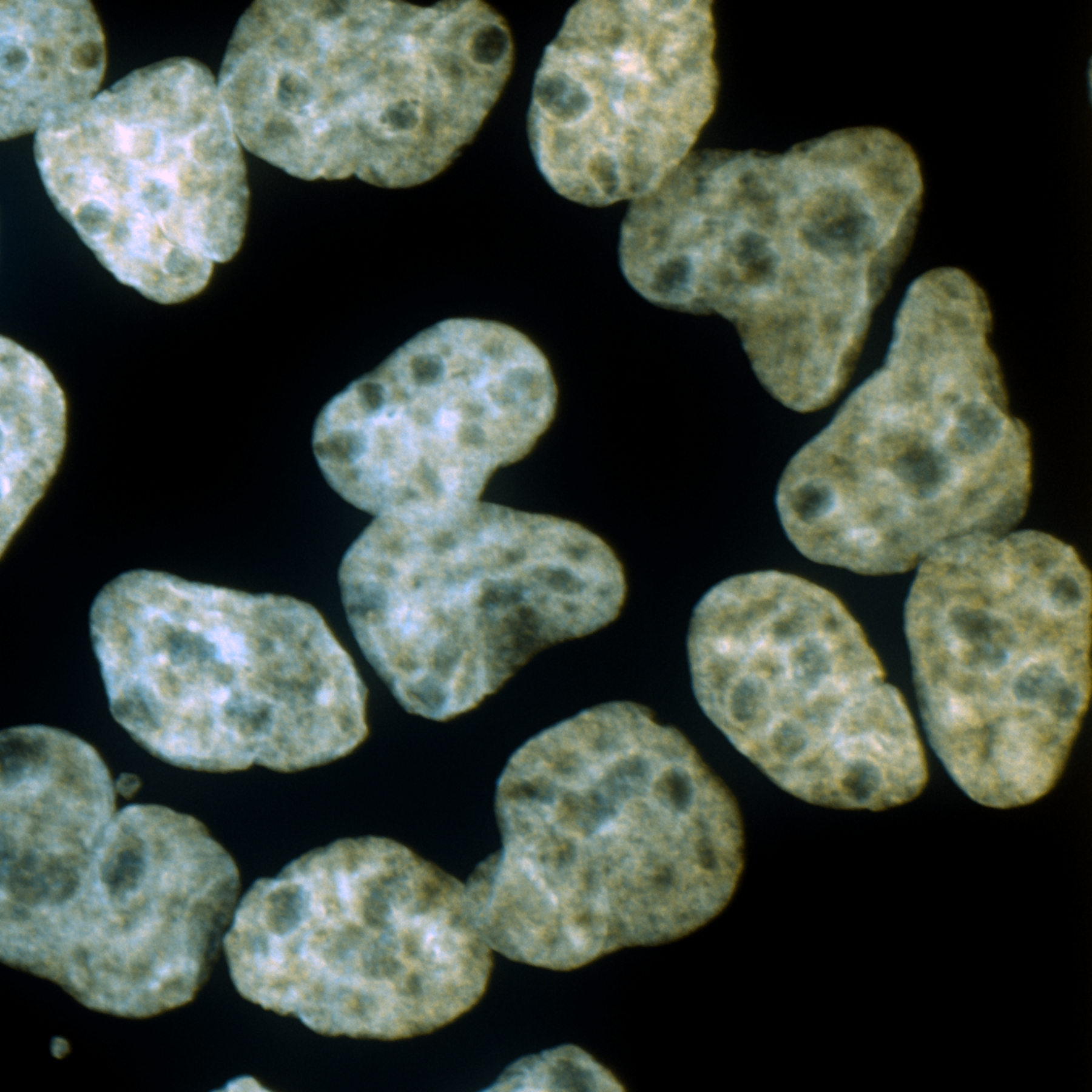

Supplement: Supplementary file 13 — Source data Figure EV1 [file 44318_2024_337_MOESM13_ESM.zip › 07_Figure_EV1/C/Imaging/U2OS-MMEJ/U2OS-MMEJ_U2OS-MMEJ_RGB.tif]

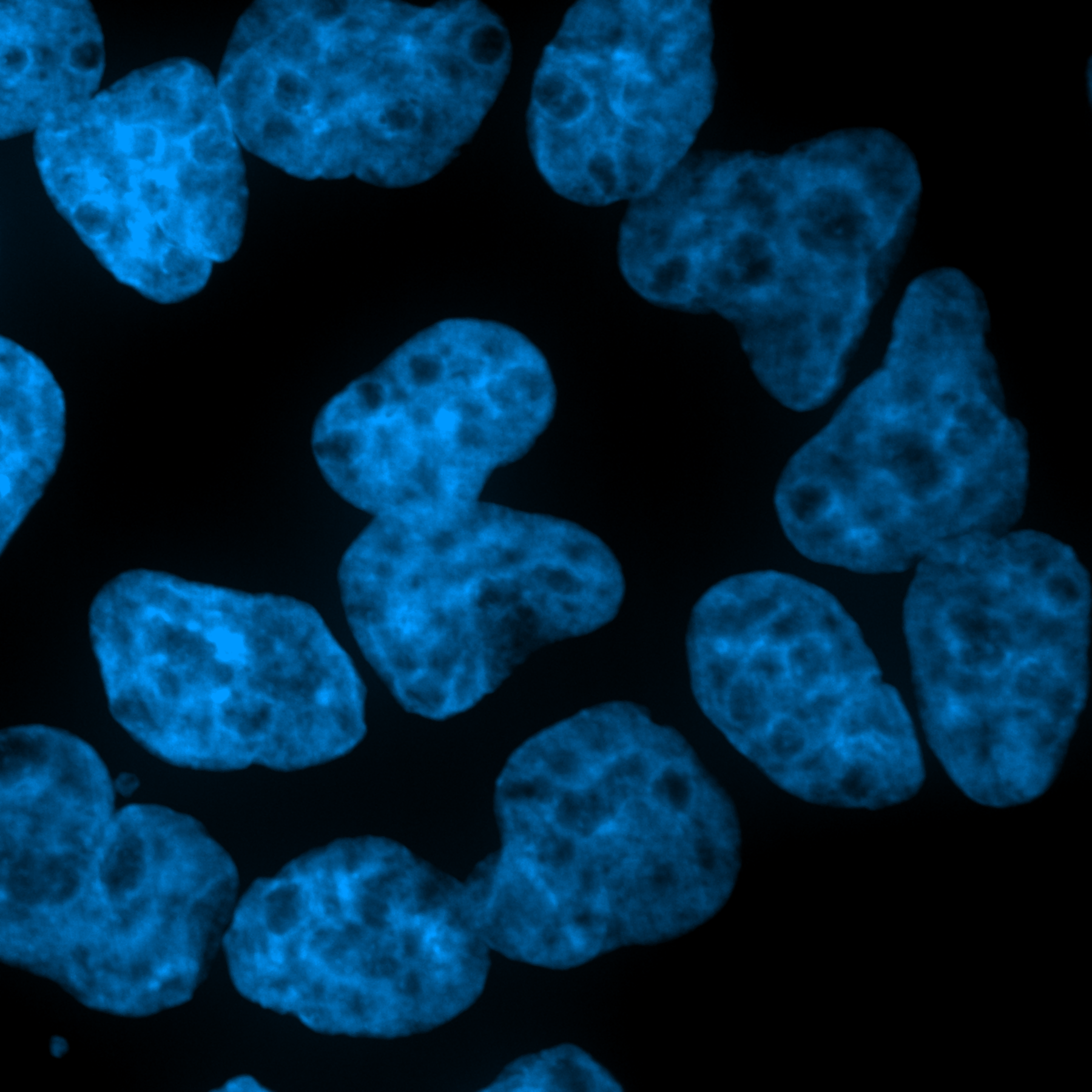

Supplement: Supplementary file 13 — Source data Figure EV1 [file 44318_2024_337_MOESM13_ESM.zip › 07_Figure_EV1/C/Imaging/U2OS-MMEJ/U2OS-MMEJ_U2OS-MMEJ_RGB_QT DAPI.tif]
