# Supplementary figures and images for: qTAG: an adaptable plasmid scaffold for CRISPR-based endogenous tagging (part 4 of 5)
Source: EMBO J. 2024 Dec 12;44(3):947–74. doi: 10.1038/s44318-024-00337-5 (PMC11790981; doi:10.1038/s44318-024-00337-5)

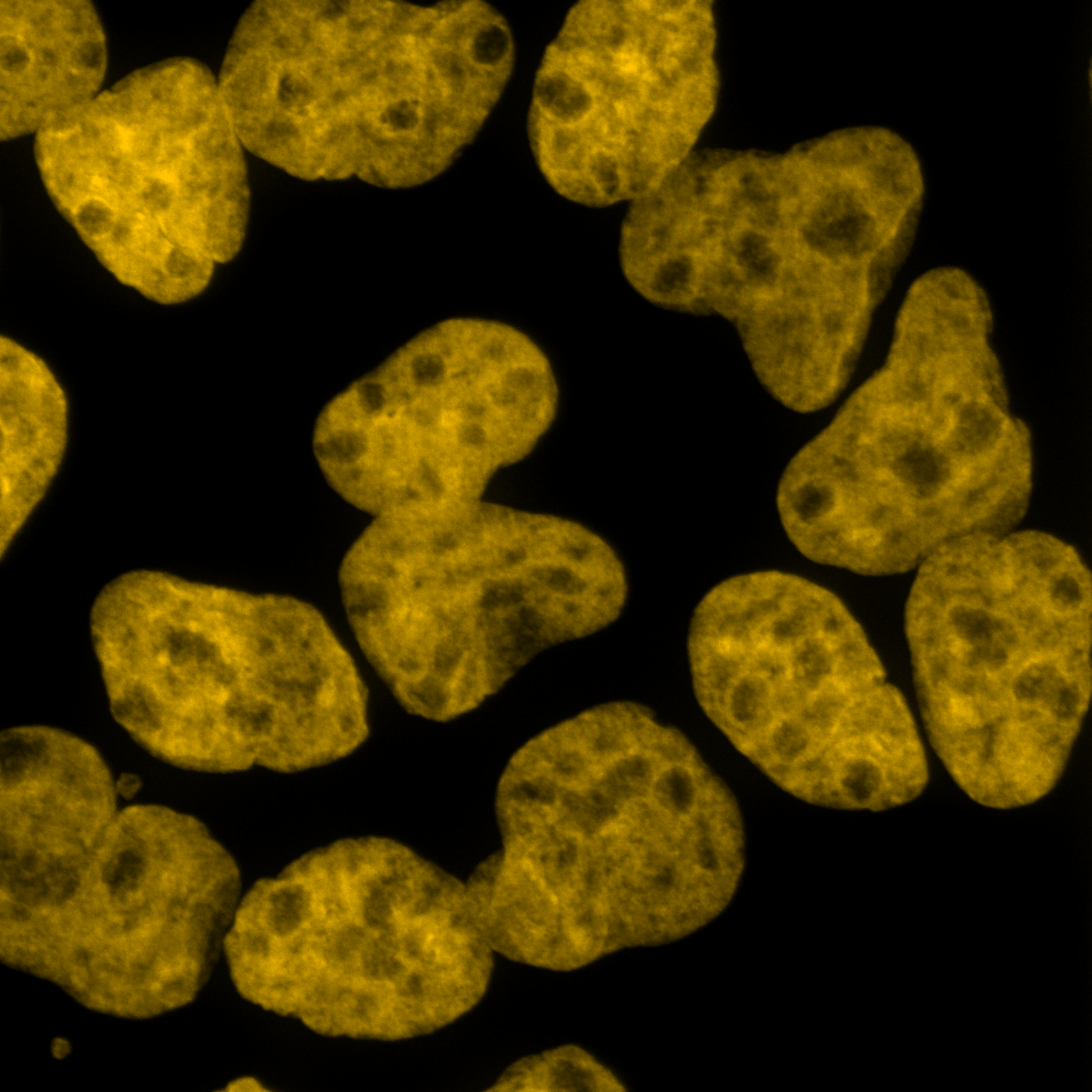

Supplement: Supplementary file 13 — Source data Figure EV1 [file 44318_2024_337_MOESM13_ESM.zip › 07_Figure_EV1/C/Imaging/U2OS-MMEJ/U2OS-MMEJ_U2OS-MMEJ_RGB_QT GFP.tif]

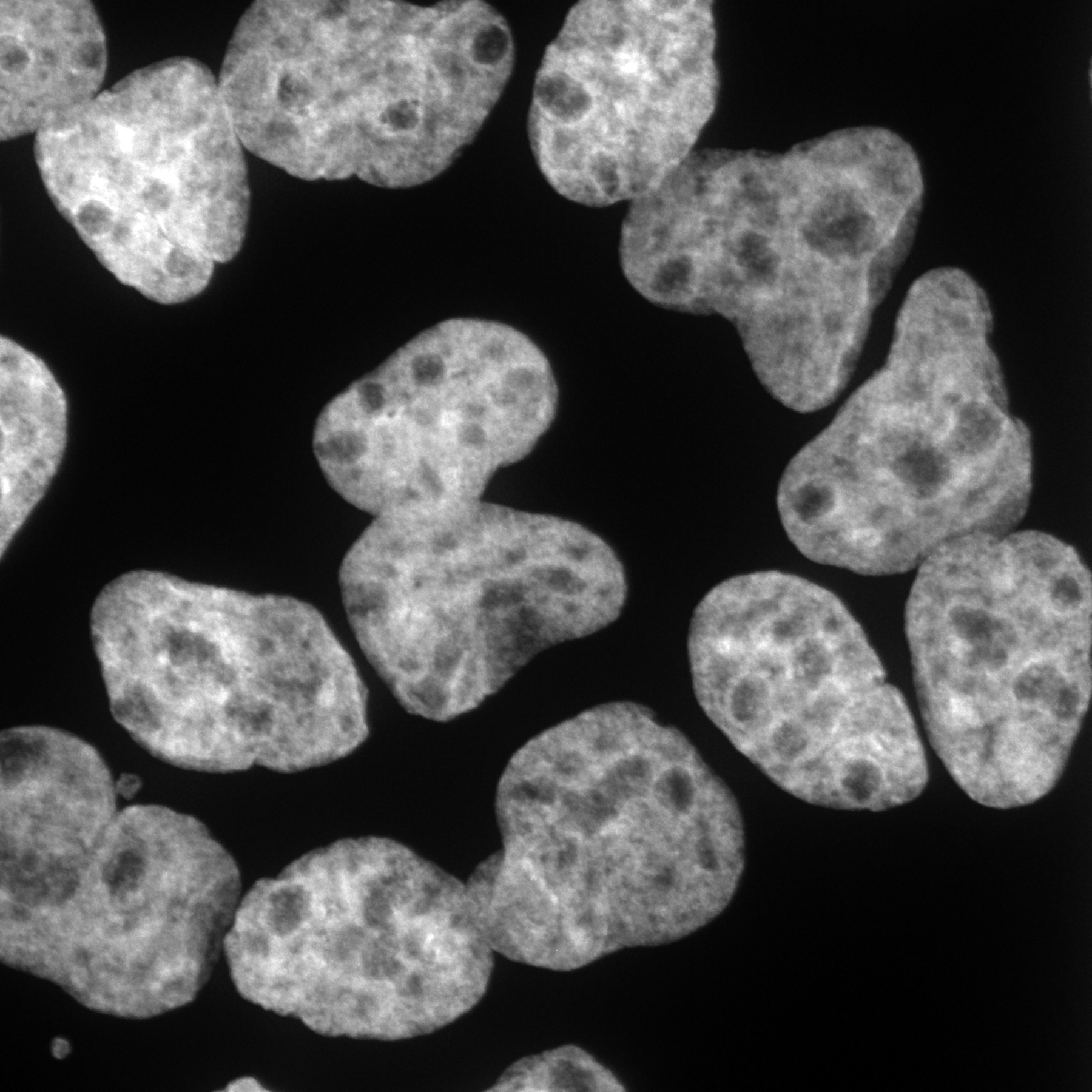

Supplement: Supplementary file 13 — Source data Figure EV1 [file 44318_2024_337_MOESM13_ESM.zip › 07_Figure_EV1/C/Imaging/U2OS-MMEJ/_FULL-RANGE-U2OS-MMEJ.tif]

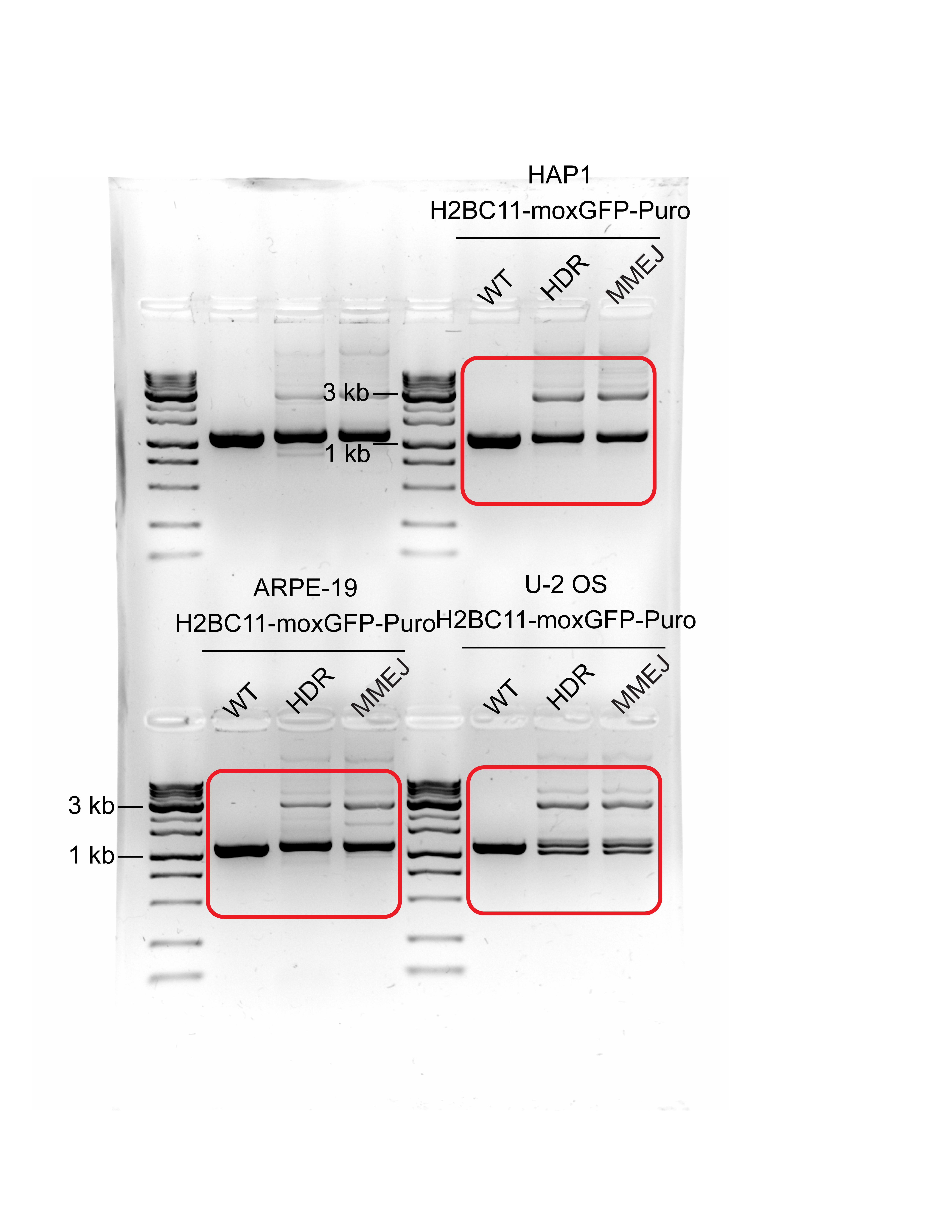

Supplement: Supplementary file 13 — Source data Figure EV1 [file 44318_2024_337_MOESM13_ESM.zip › 07_Figure_EV1/D/Alt-Cells-Selection-Enrichment-Uncropped-Gel.tif]

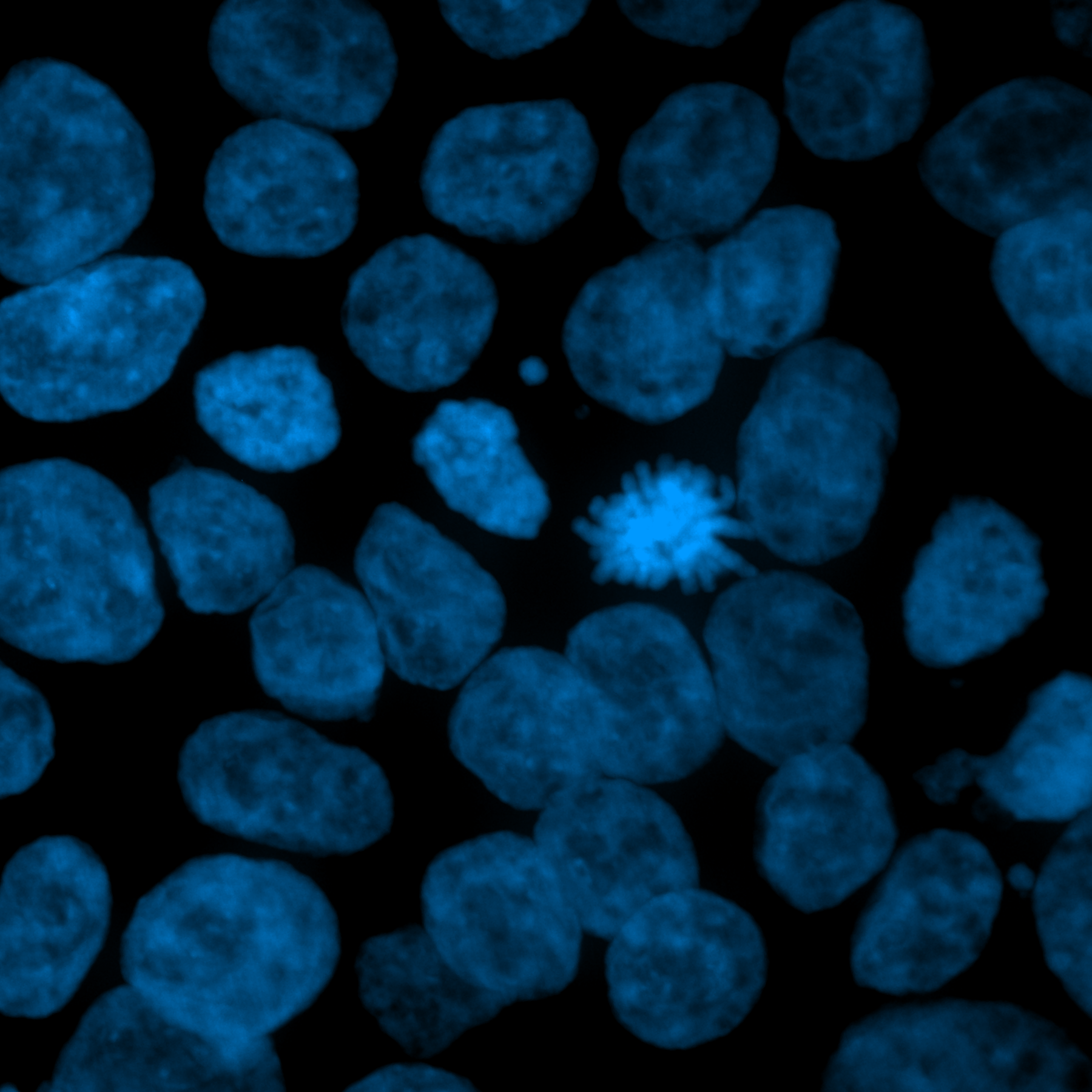

Supplement: Supplementary file 13 — Source data Figure EV1 [file 44318_2024_337_MOESM13_ESM.zip › 07_Figure_EV1/E/Imaging/BLAST_CTRL/BLAST_CTRL_DAPI.tif]

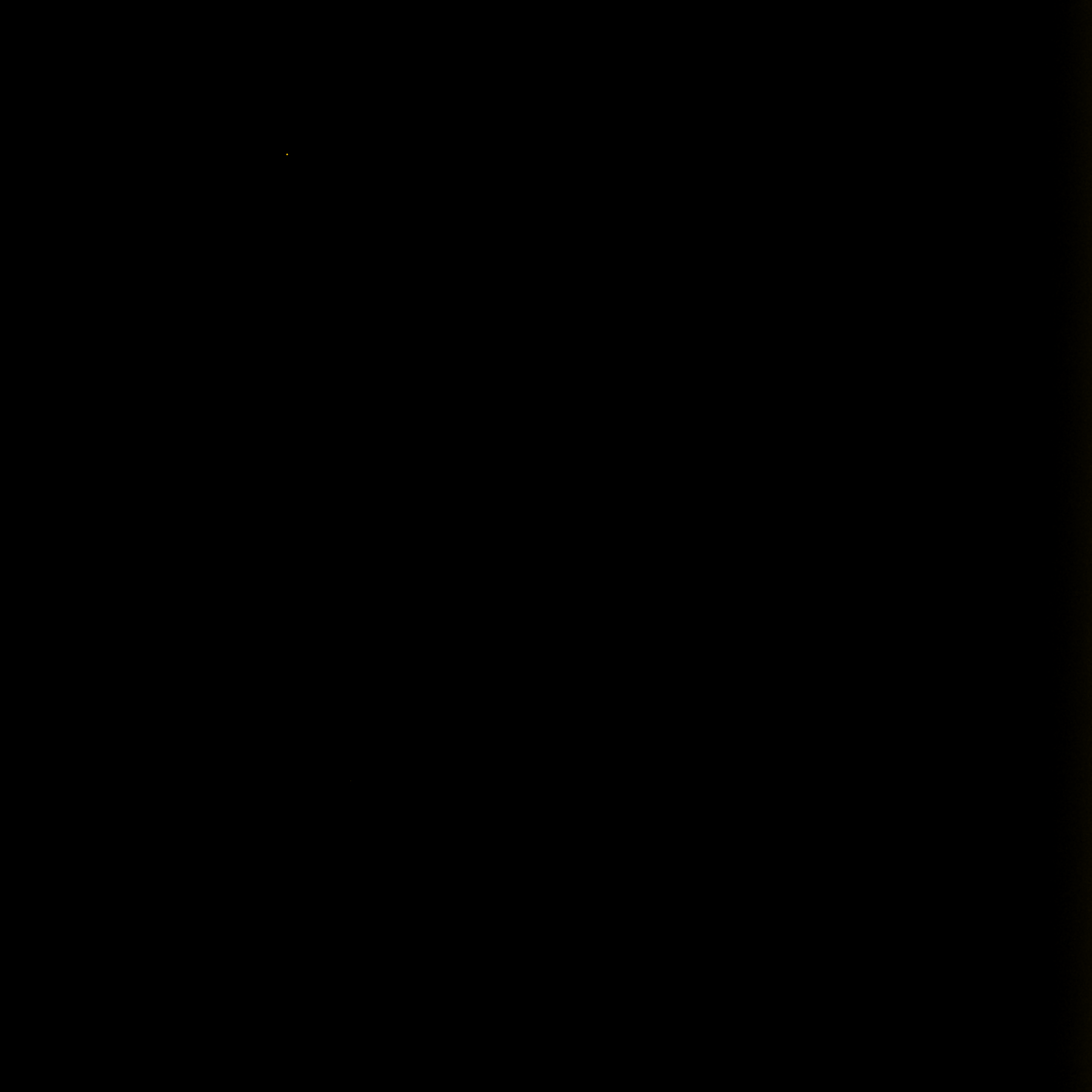

Supplement: Supplementary file 13 — Source data Figure EV1 [file 44318_2024_337_MOESM13_ESM.zip › 07_Figure_EV1/E/Imaging/BLAST_CTRL/BLAST_CTRL_GFP.tif]

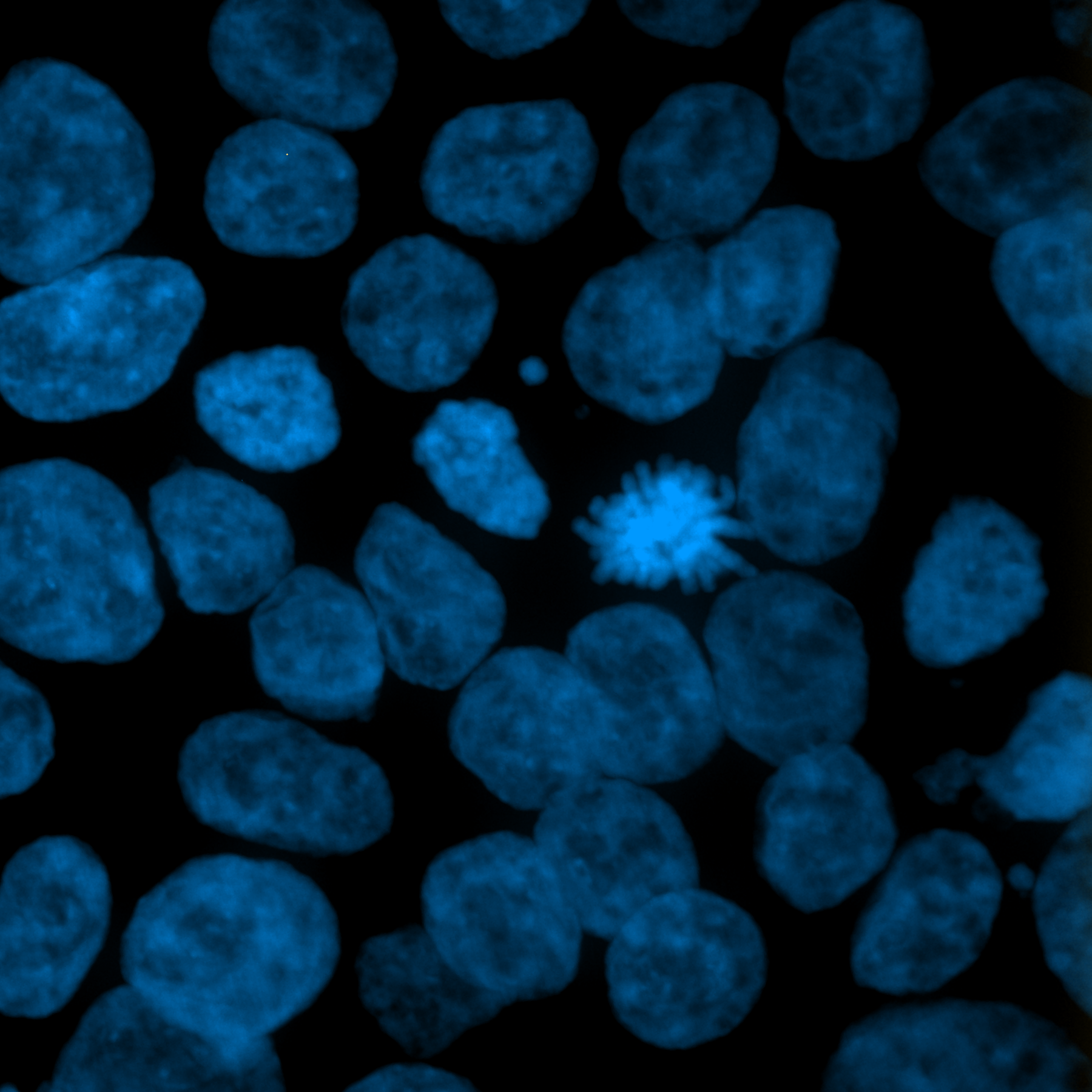

Supplement: Supplementary file 13 — Source data Figure EV1 [file 44318_2024_337_MOESM13_ESM.zip › 07_Figure_EV1/E/Imaging/BLAST_CTRL/BLAST_CTRL_Merge.tif]

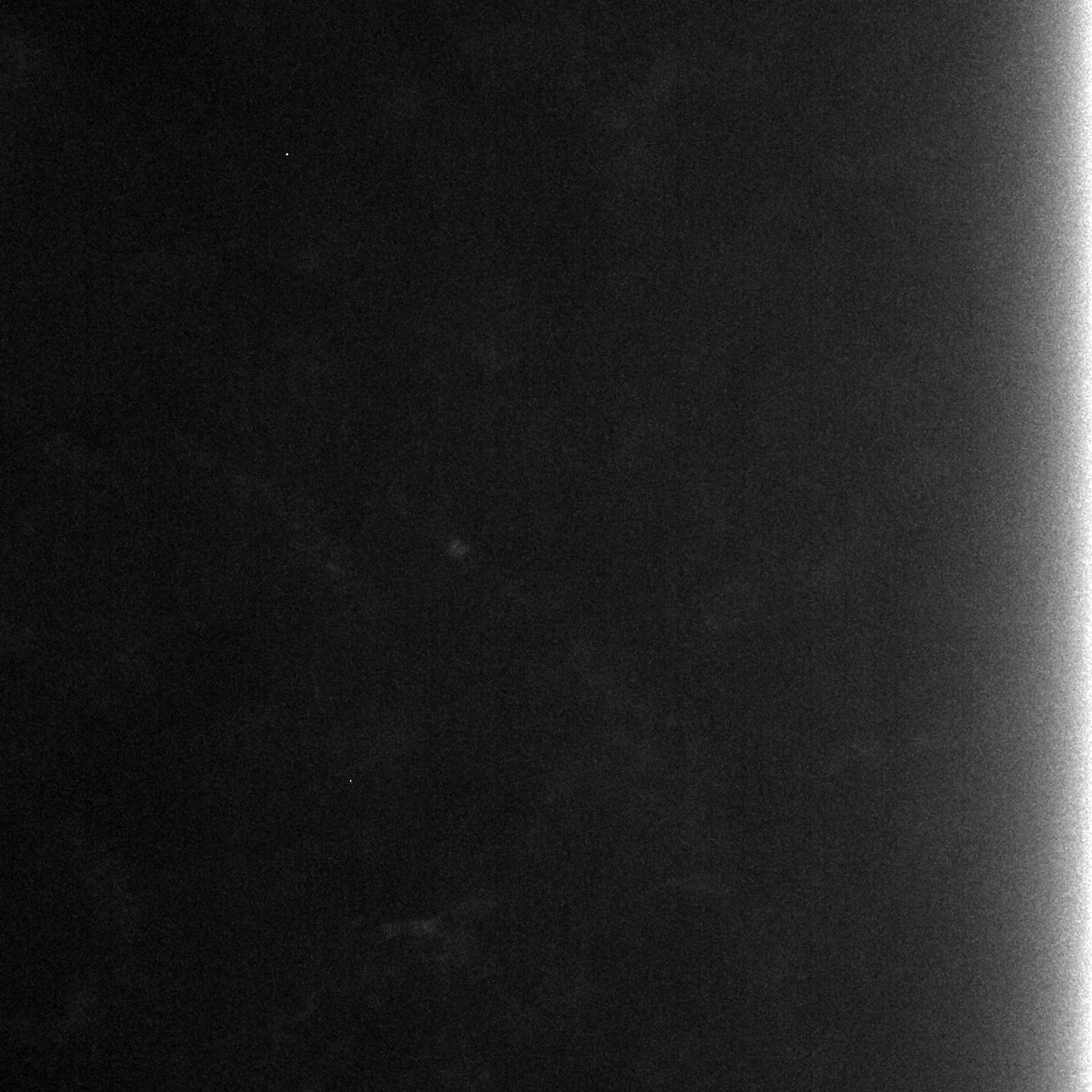

Supplement: Supplementary file 13 — Source data Figure EV1 [file 44318_2024_337_MOESM13_ESM.zip › 07_Figure_EV1/E/Imaging/BLAST_CTRL/_FULL-RANGE-BLAST_CTRL.tif]

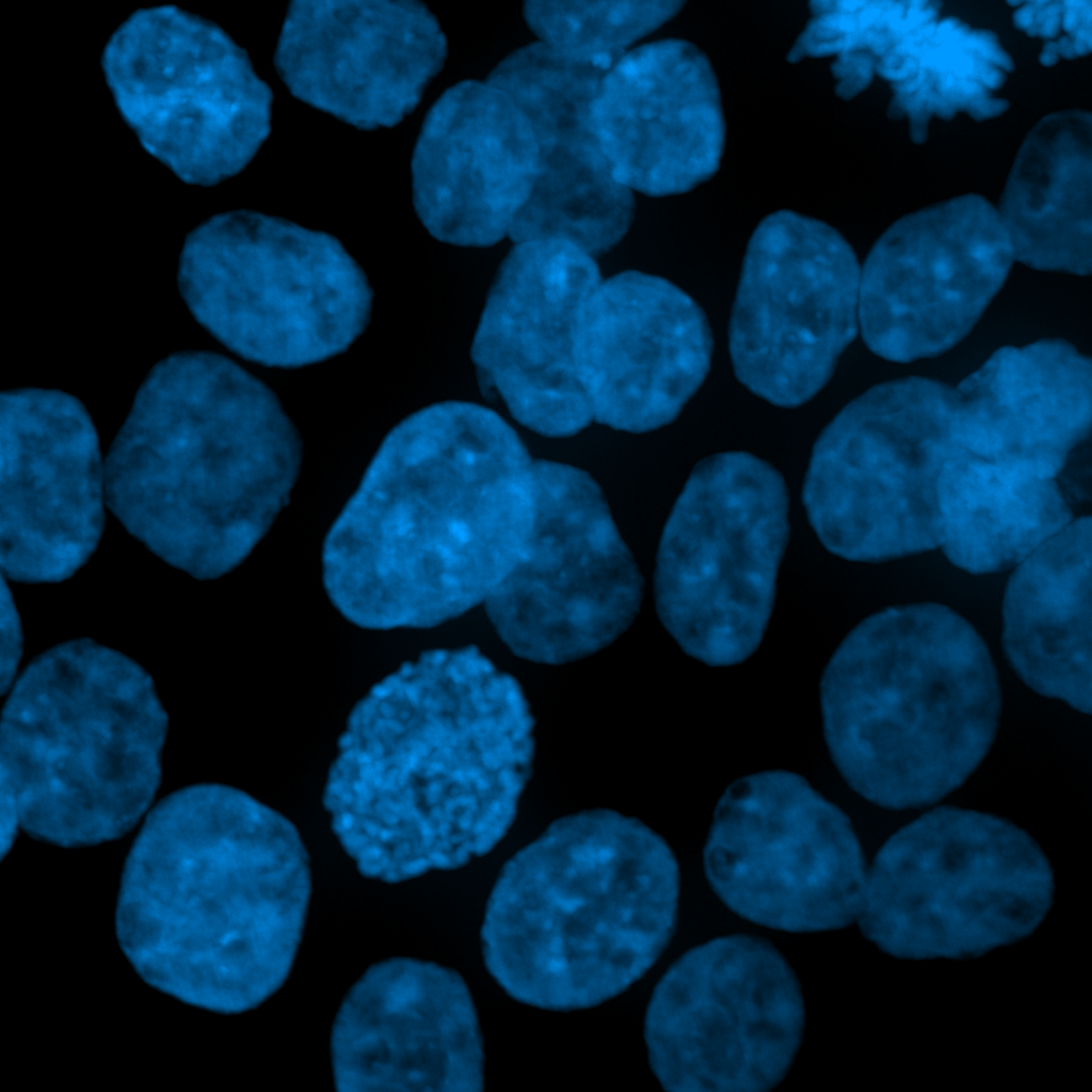

Supplement: Supplementary file 13 — Source data Figure EV1 [file 44318_2024_337_MOESM13_ESM.zip › 07_Figure_EV1/E/Imaging/BLAST_SEL/BLAST_SEL_DAPI.tif]

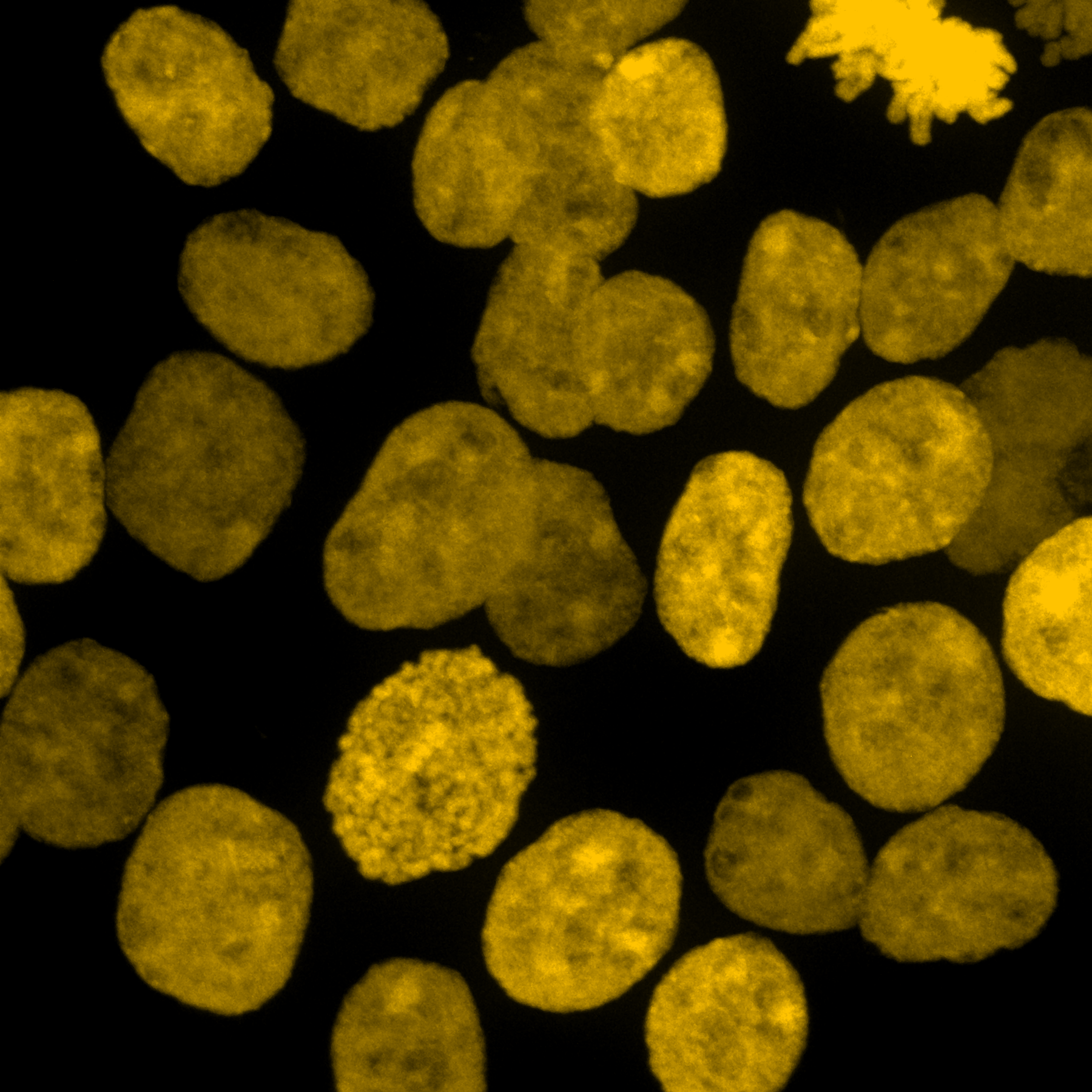

Supplement: Supplementary file 13 — Source data Figure EV1 [file 44318_2024_337_MOESM13_ESM.zip › 07_Figure_EV1/E/Imaging/BLAST_SEL/BLAST_SEL_GFP.tif]

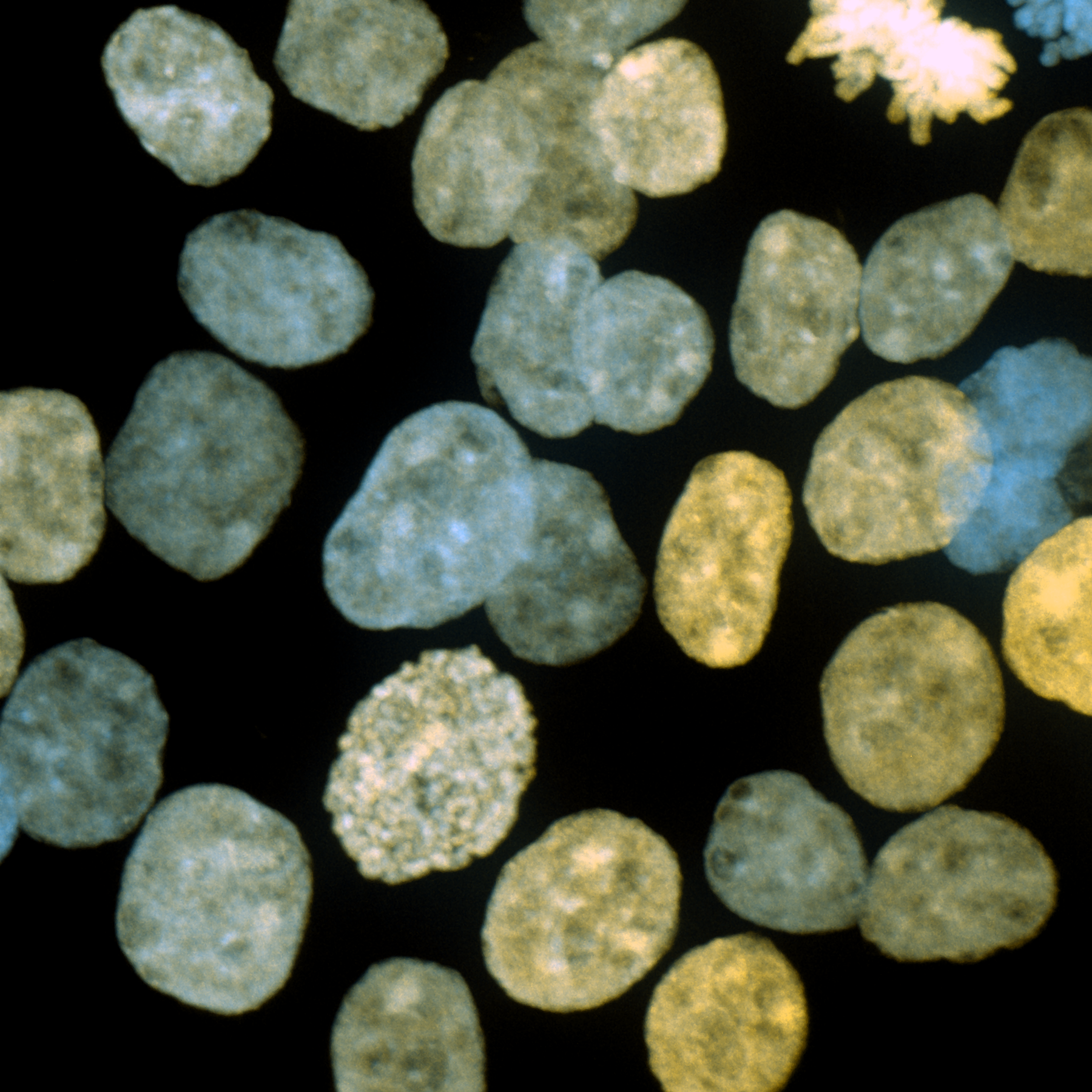

Supplement: Supplementary file 13 — Source data Figure EV1 [file 44318_2024_337_MOESM13_ESM.zip › 07_Figure_EV1/E/Imaging/BLAST_SEL/BLAST_SEL_Merge.tif]

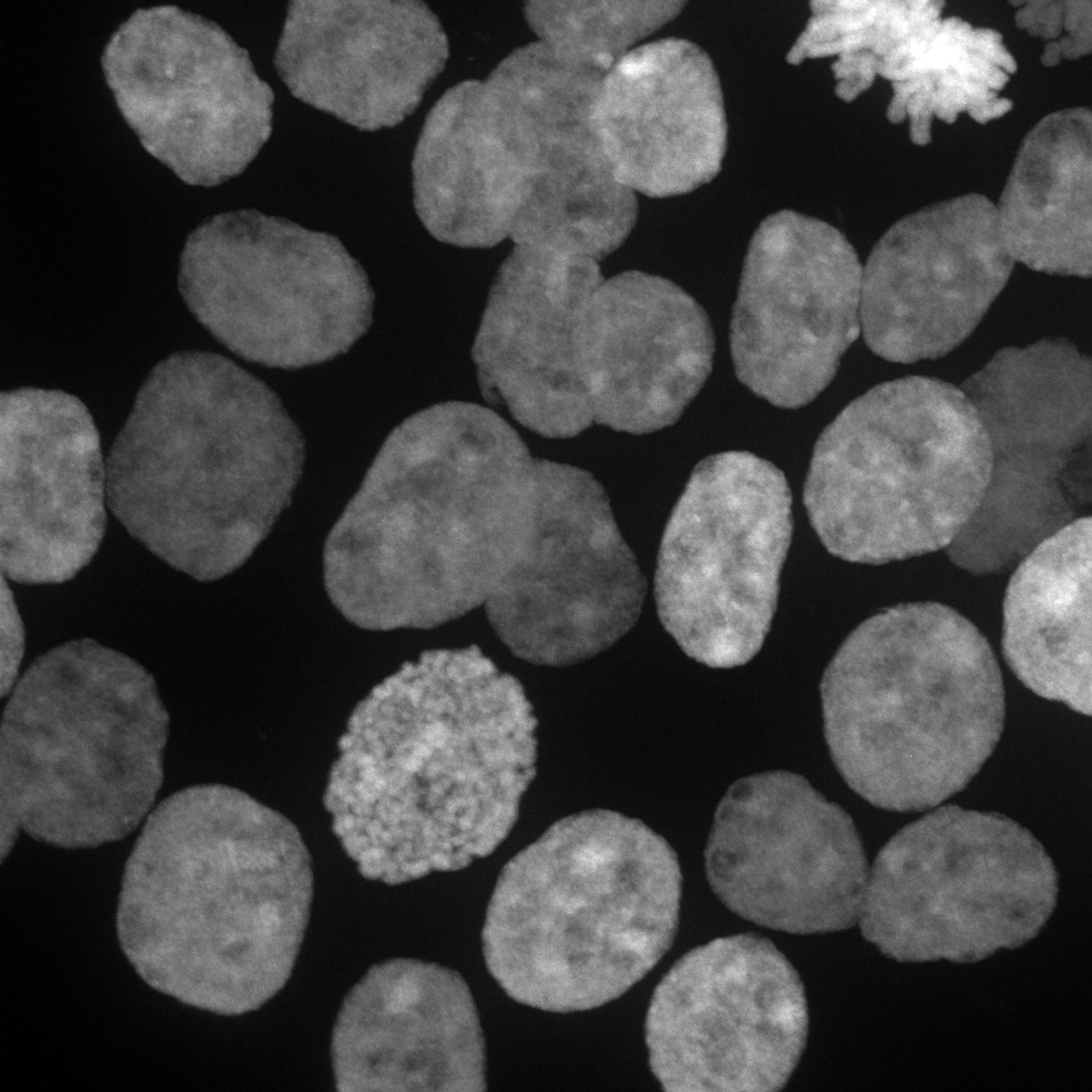

Supplement: Supplementary file 13 — Source data Figure EV1 [file 44318_2024_337_MOESM13_ESM.zip › 07_Figure_EV1/E/Imaging/BLAST_SEL/_FULL-RANGE-BLAST_SEL.tif]

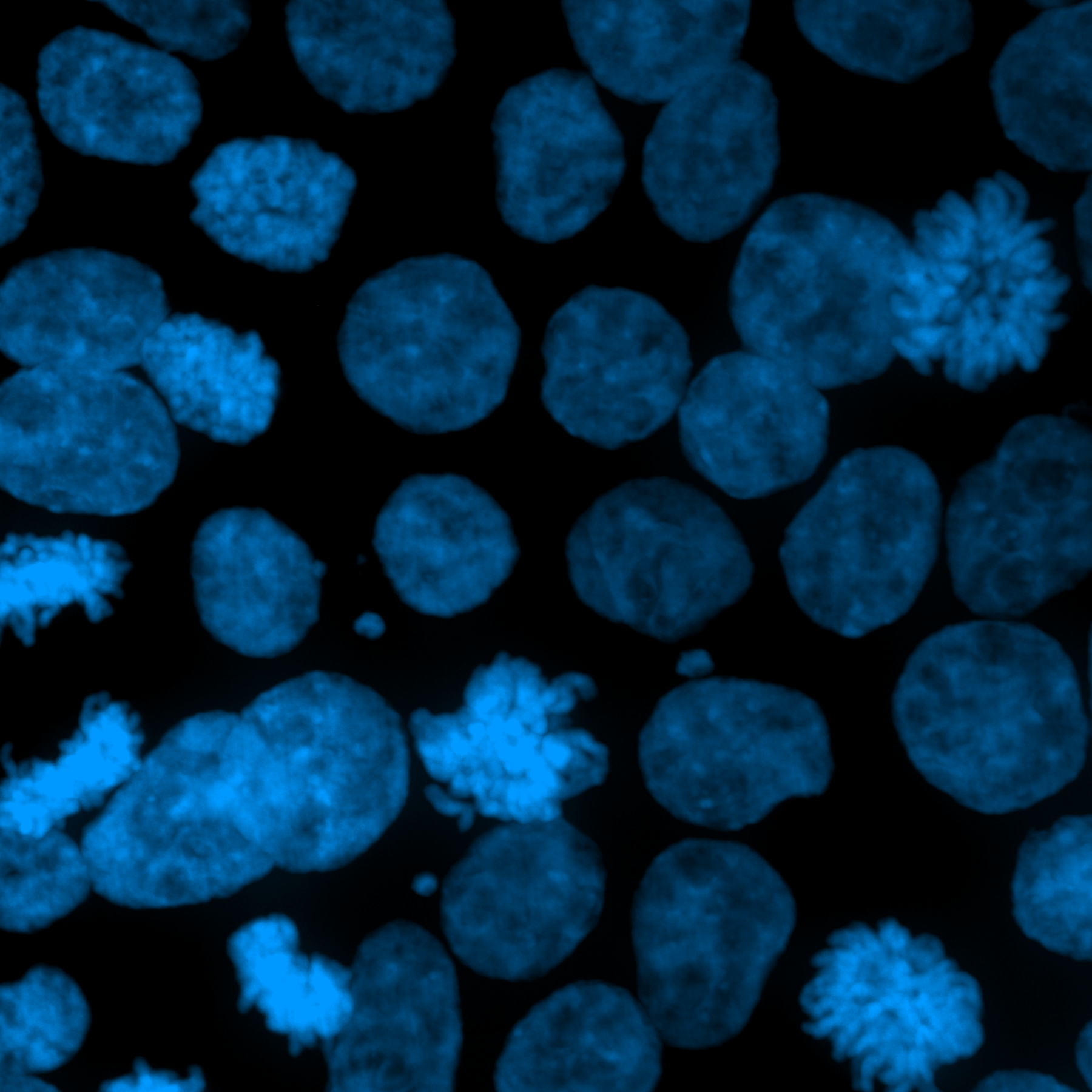

Supplement: Supplementary file 13 — Source data Figure EV1 [file 44318_2024_337_MOESM13_ESM.zip › 07_Figure_EV1/E/Imaging/PURO_CTRL/PURO_CTRL_DAPI.tif]

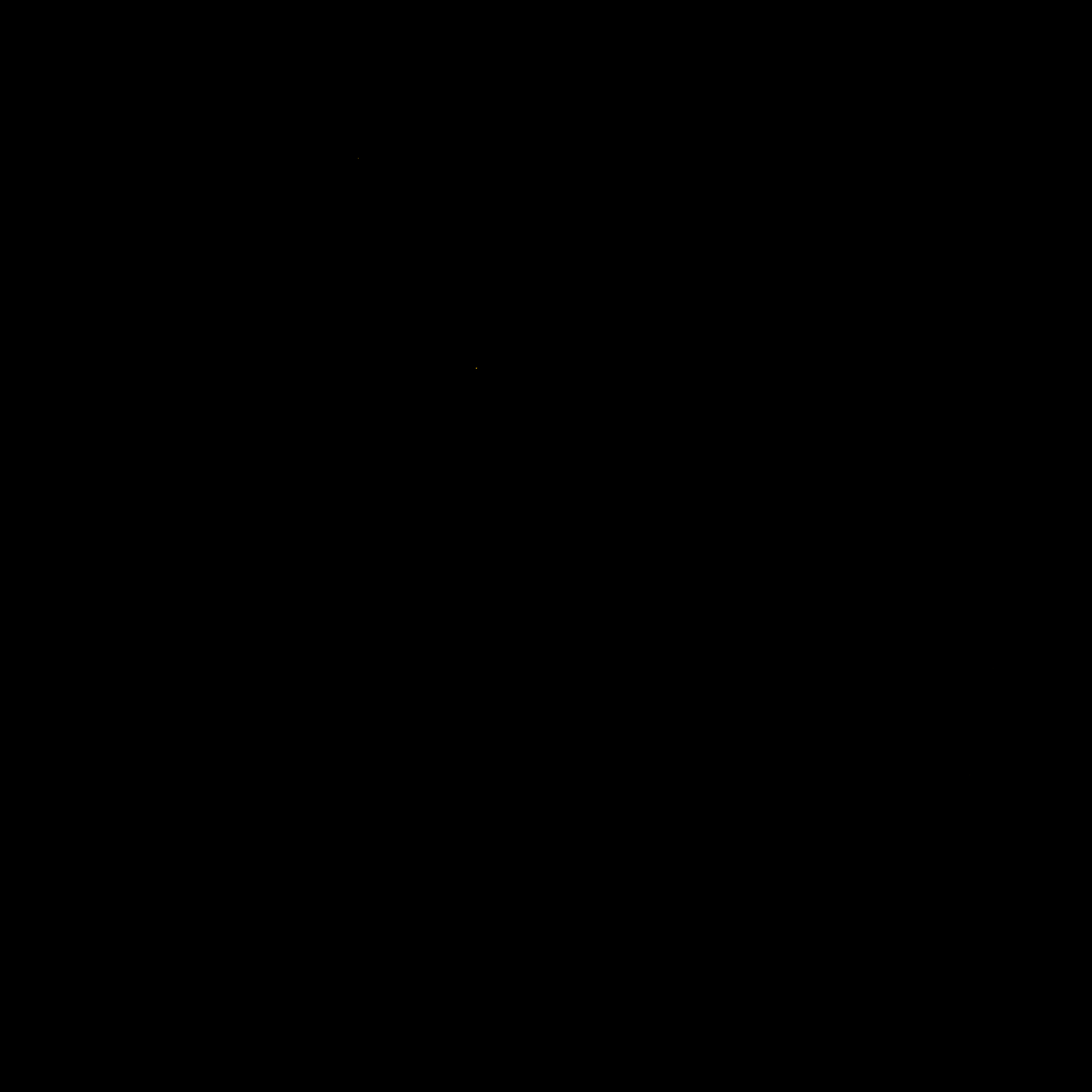

Supplement: Supplementary file 13 — Source data Figure EV1 [file 44318_2024_337_MOESM13_ESM.zip › 07_Figure_EV1/E/Imaging/PURO_CTRL/PURO_CTRL_GFP.tif]

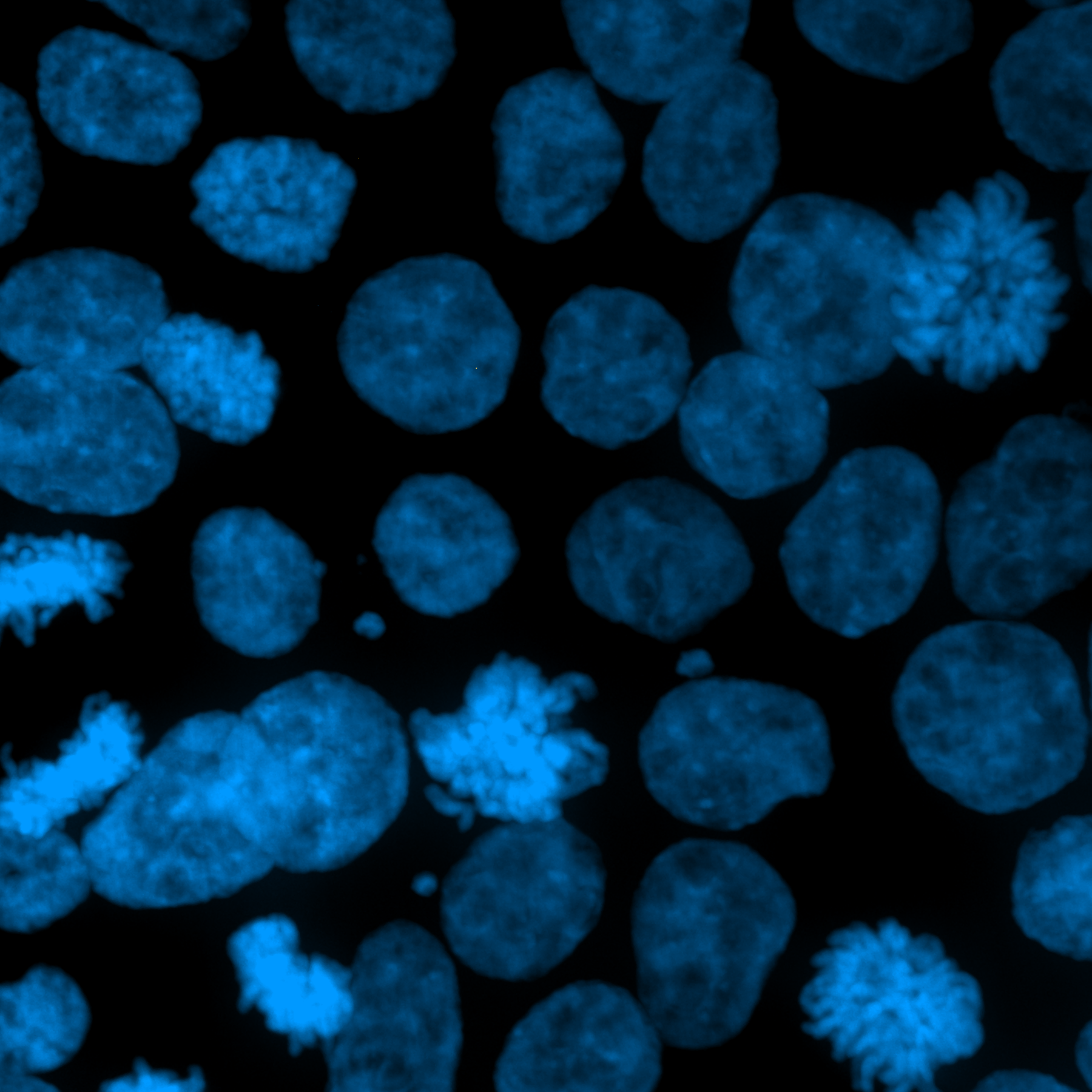

Supplement: Supplementary file 13 — Source data Figure EV1 [file 44318_2024_337_MOESM13_ESM.zip › 07_Figure_EV1/E/Imaging/PURO_CTRL/PURO_CTRL_Merge.tif]

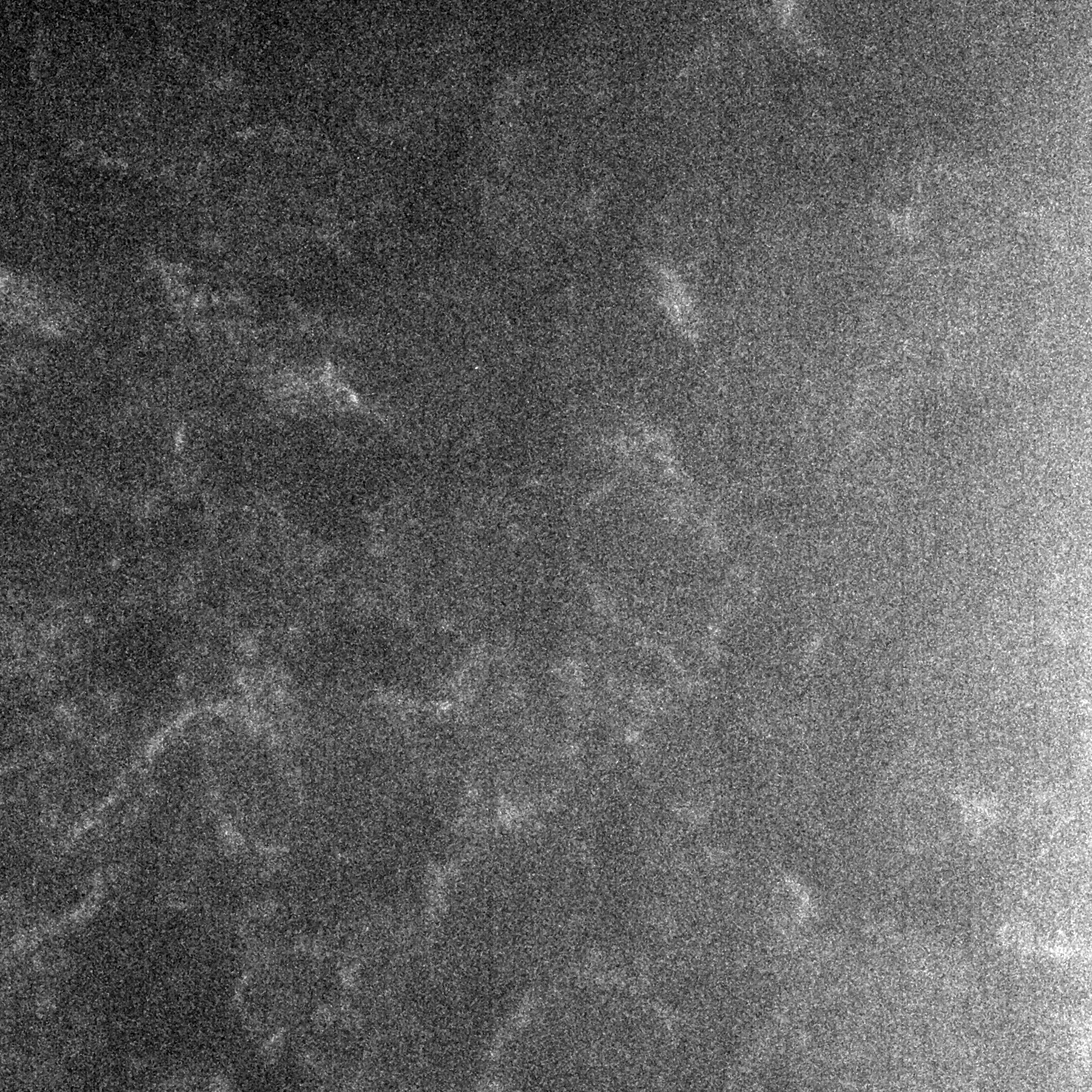

Supplement: Supplementary file 13 — Source data Figure EV1 [file 44318_2024_337_MOESM13_ESM.zip › 07_Figure_EV1/E/Imaging/PURO_CTRL/_FULL-RANGE-PURO_CTRL.tif]

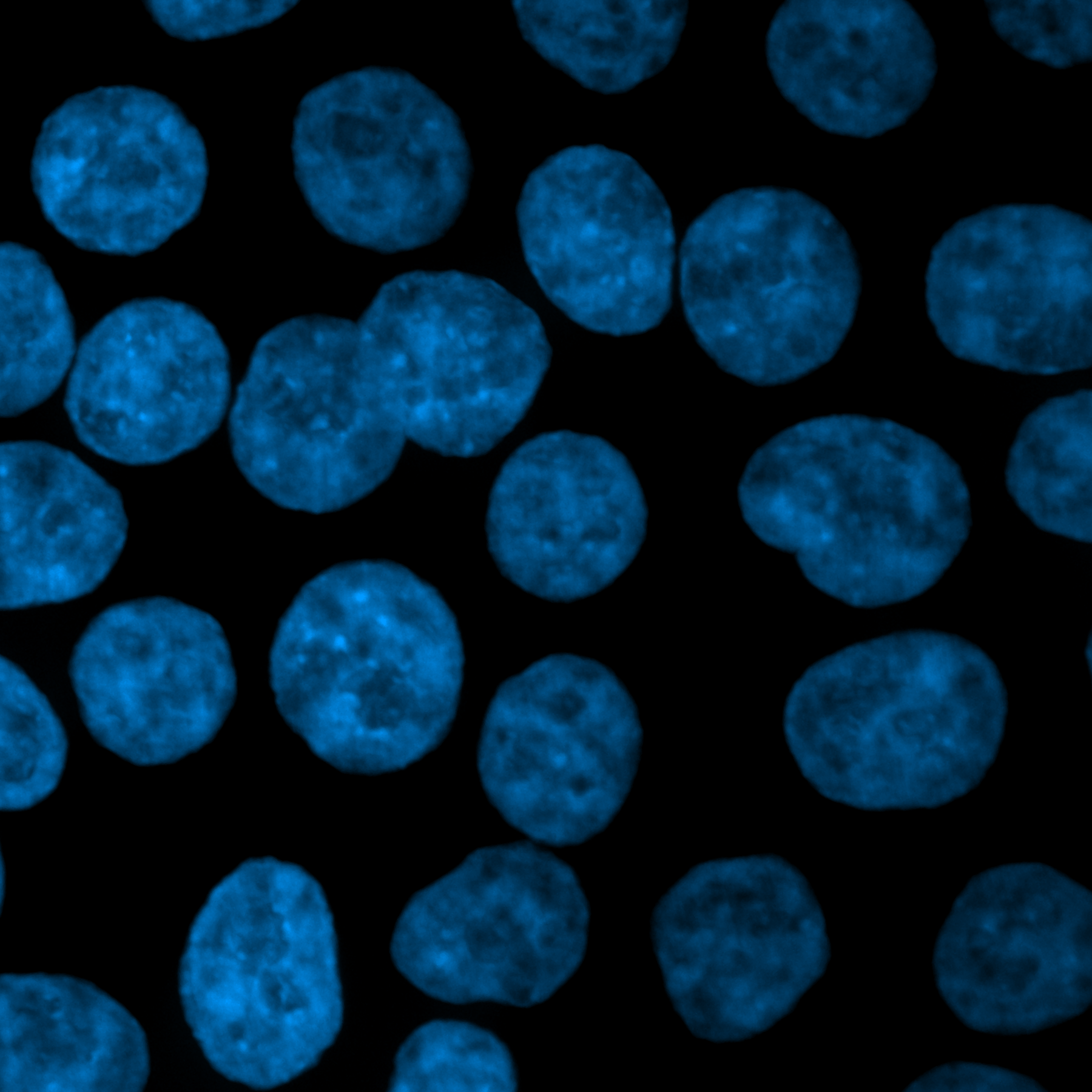

Supplement: Supplementary file 13 — Source data Figure EV1 [file 44318_2024_337_MOESM13_ESM.zip › 07_Figure_EV1/E/Imaging/PURO_SEL/PURO_SEL_DAPI.tif]

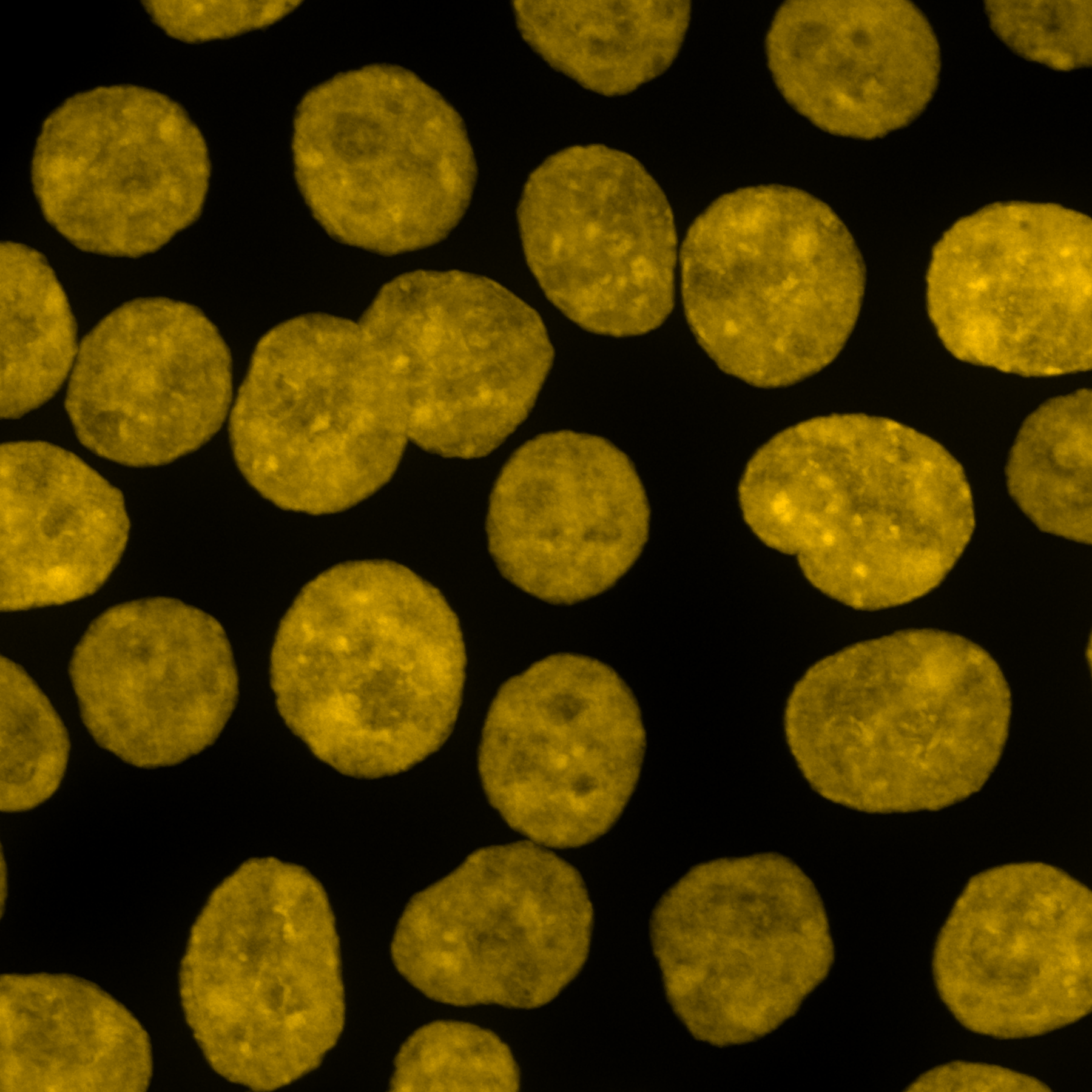

Supplement: Supplementary file 13 — Source data Figure EV1 [file 44318_2024_337_MOESM13_ESM.zip › 07_Figure_EV1/E/Imaging/PURO_SEL/PURO_SEL_GFP.tif]

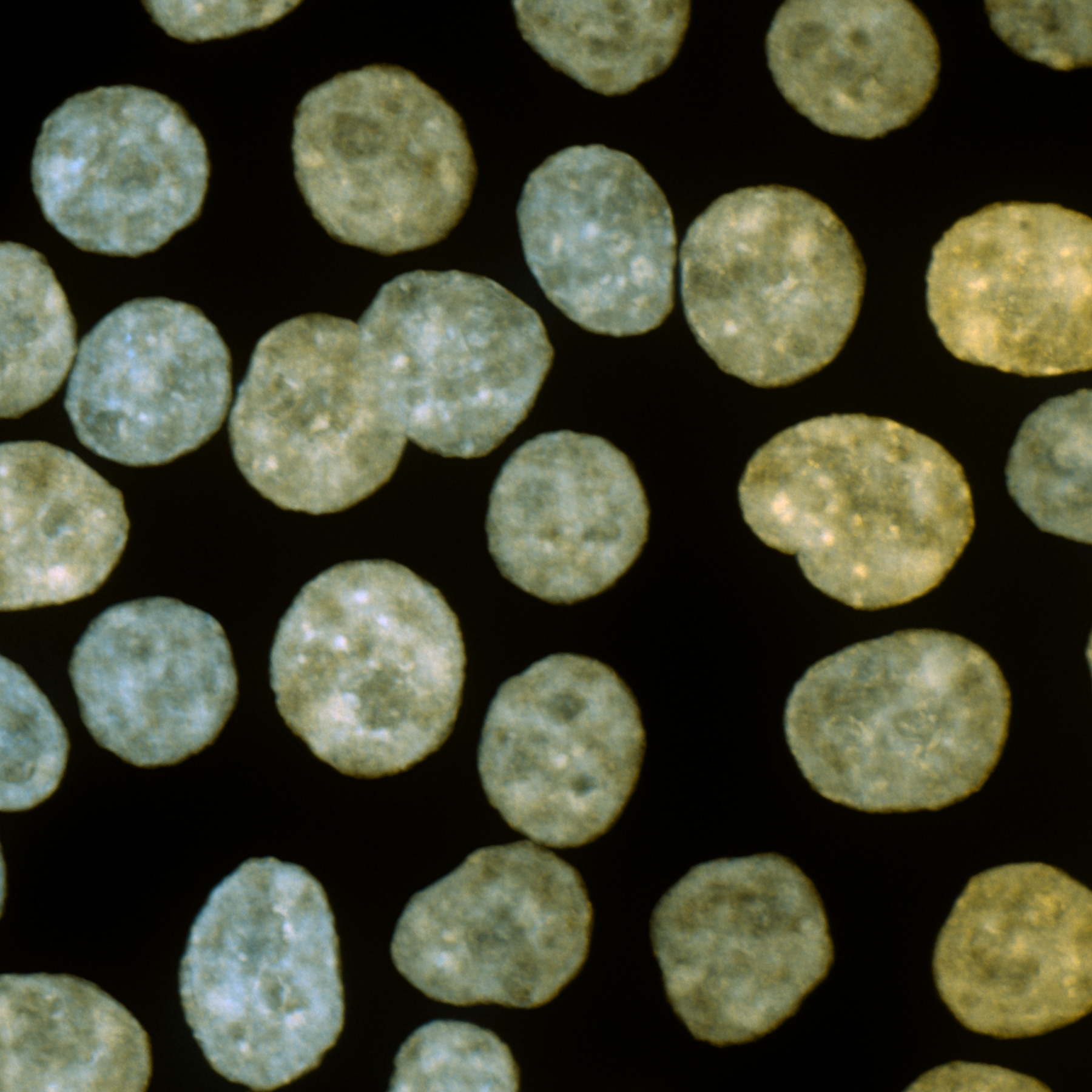

Supplement: Supplementary file 13 — Source data Figure EV1 [file 44318_2024_337_MOESM13_ESM.zip › 07_Figure_EV1/E/Imaging/PURO_SEL/PURO_SEL_Merge.tif]

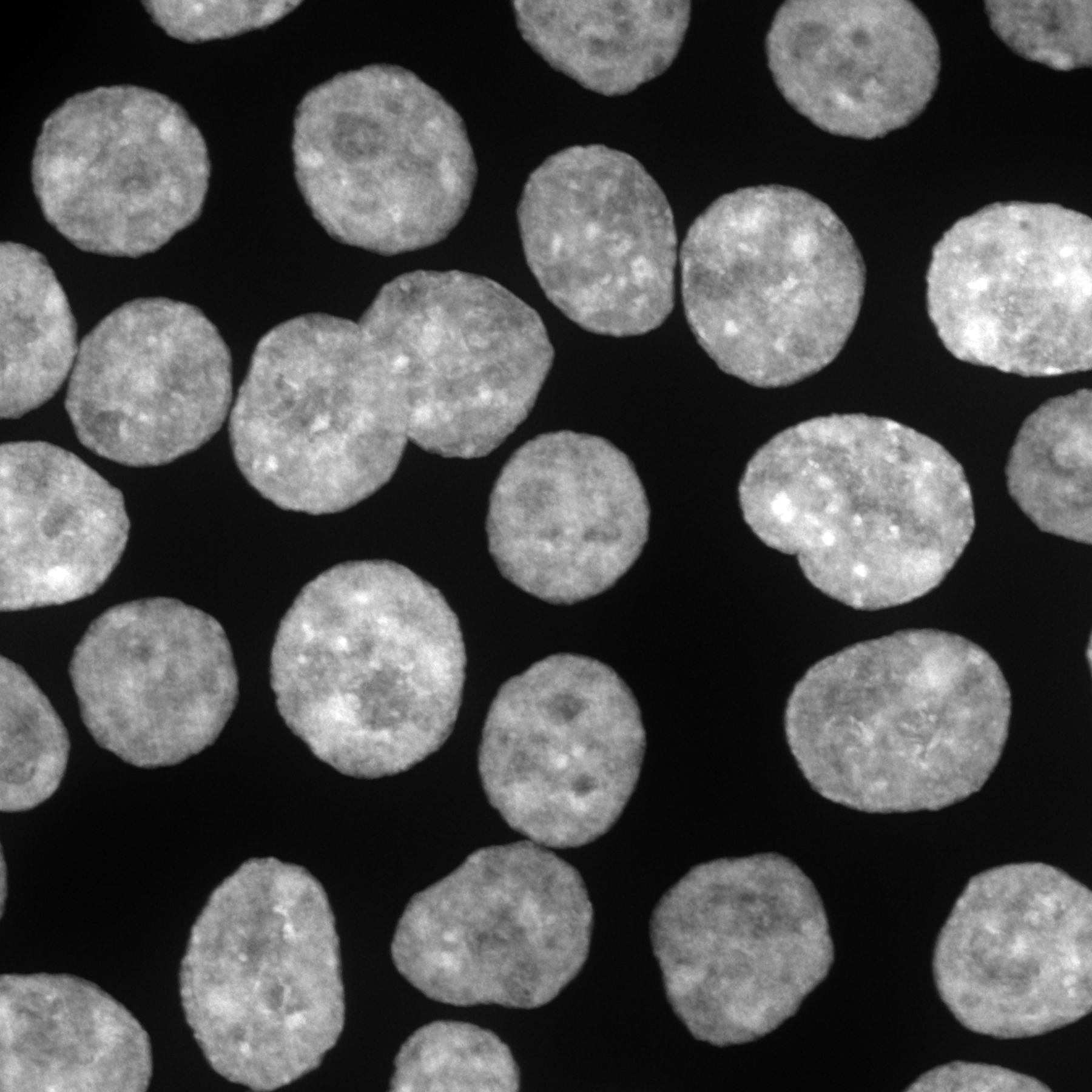

Supplement: Supplementary file 13 — Source data Figure EV1 [file 44318_2024_337_MOESM13_ESM.zip › 07_Figure_EV1/E/Imaging/PURO_SEL/_FULL-RANGE-PURO_SEL.tif]

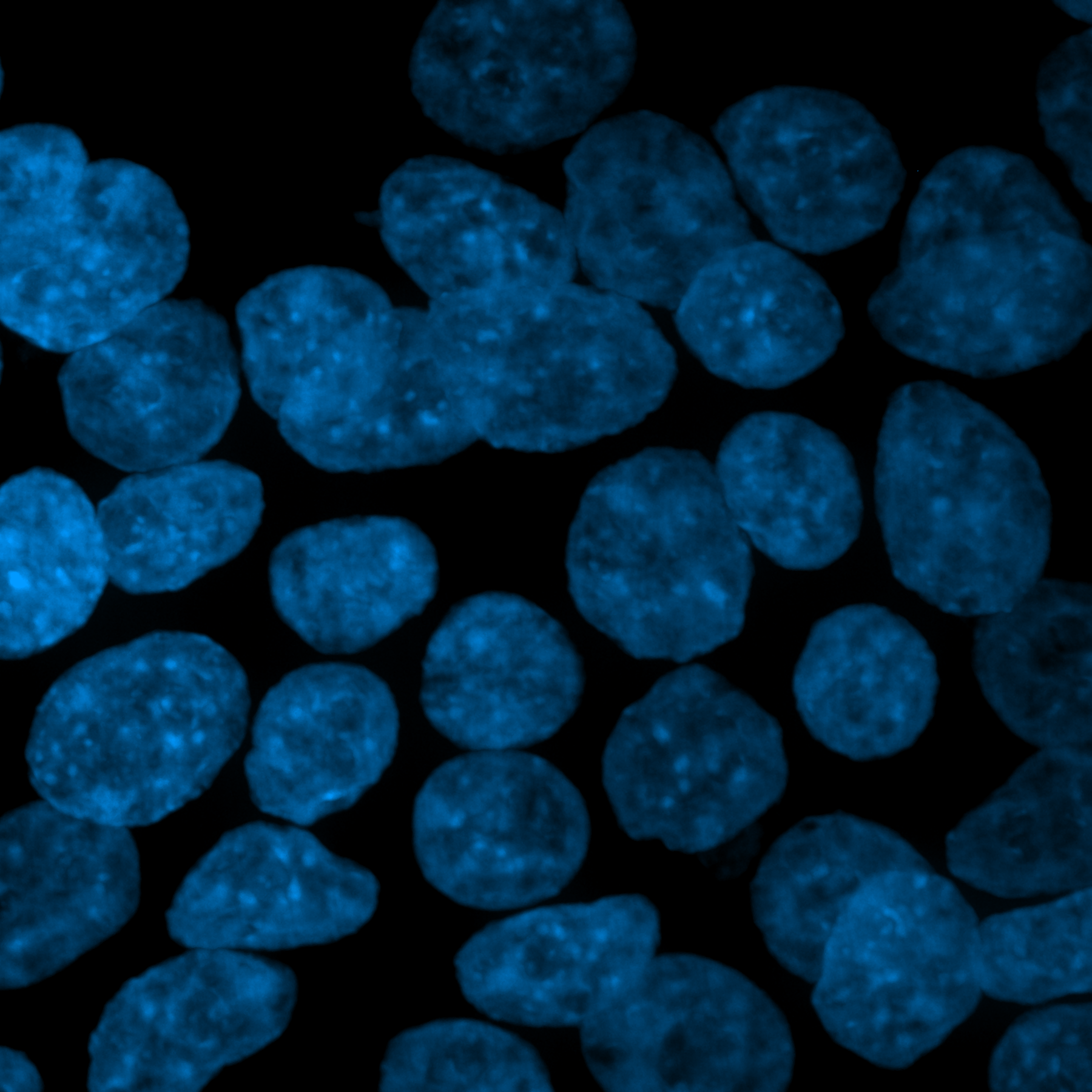

Supplement: Supplementary file 13 — Source data Figure EV1 [file 44318_2024_337_MOESM13_ESM.zip › 07_Figure_EV1/E/Imaging/ZEO_CTRL/ZEO_CTRL_DAPI.tif]

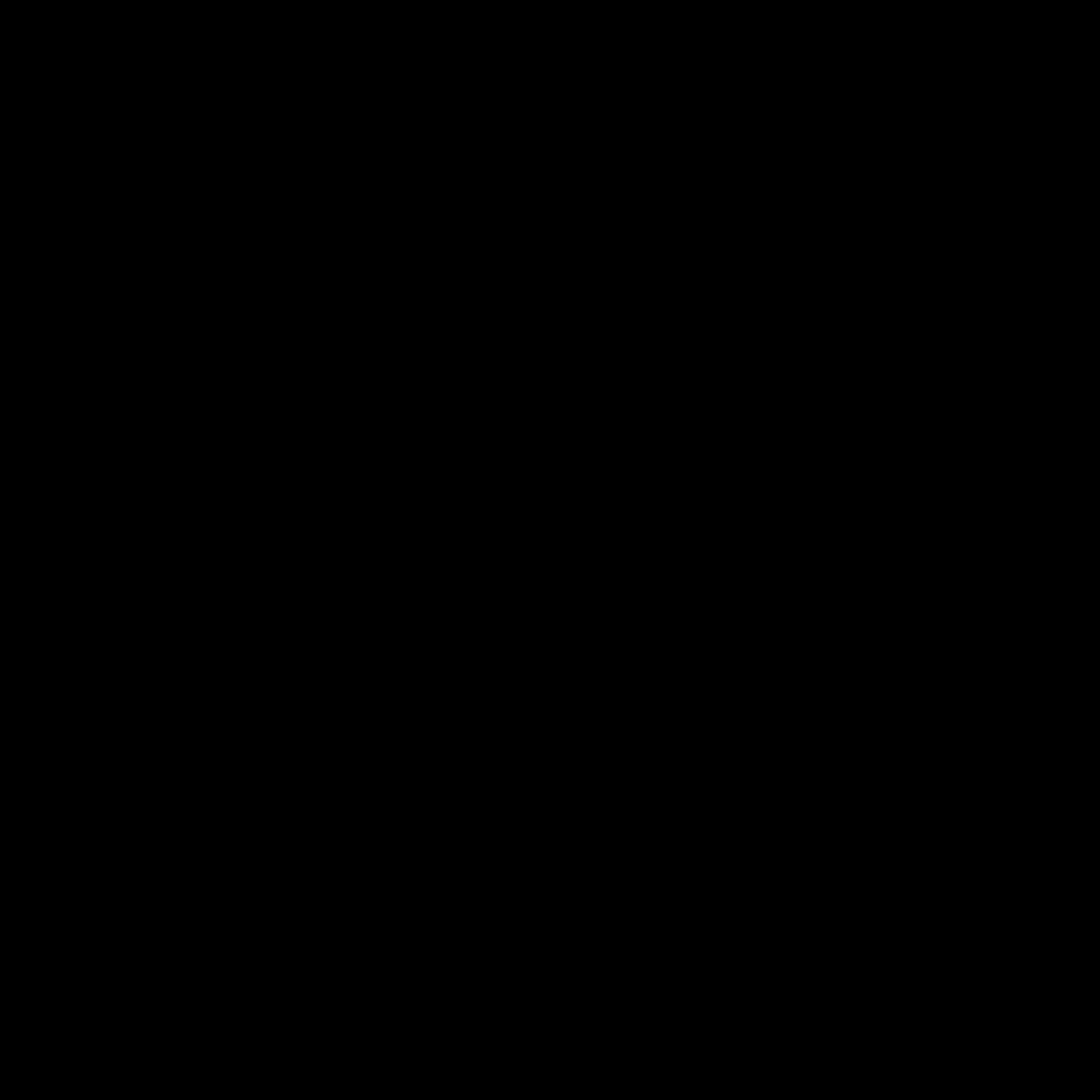

Supplement: Supplementary file 13 — Source data Figure EV1 [file 44318_2024_337_MOESM13_ESM.zip › 07_Figure_EV1/E/Imaging/ZEO_CTRL/ZEO_CTRL_GFP.tif]

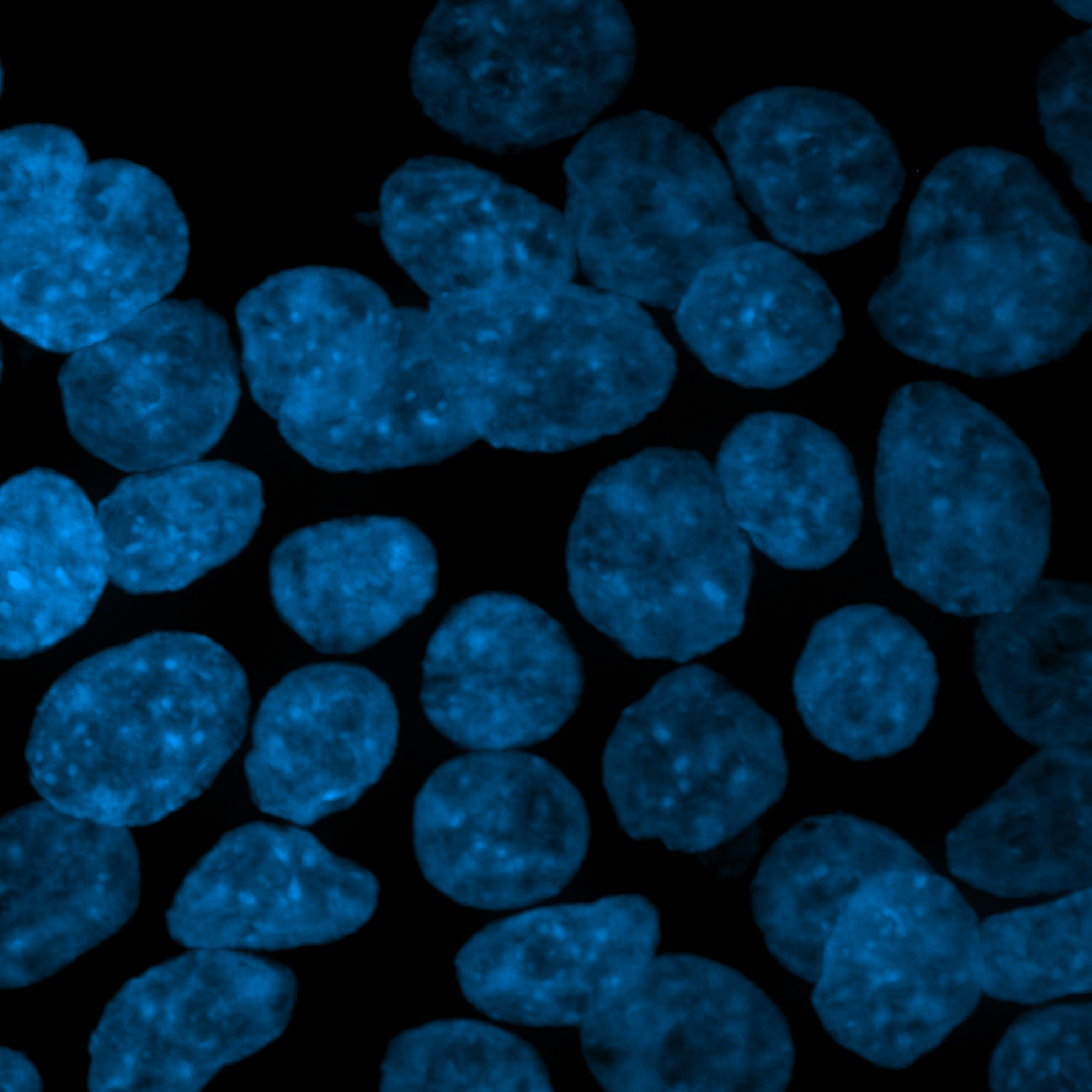

Supplement: Supplementary file 13 — Source data Figure EV1 [file 44318_2024_337_MOESM13_ESM.zip › 07_Figure_EV1/E/Imaging/ZEO_CTRL/ZEO_CTRL_Merge.tif]

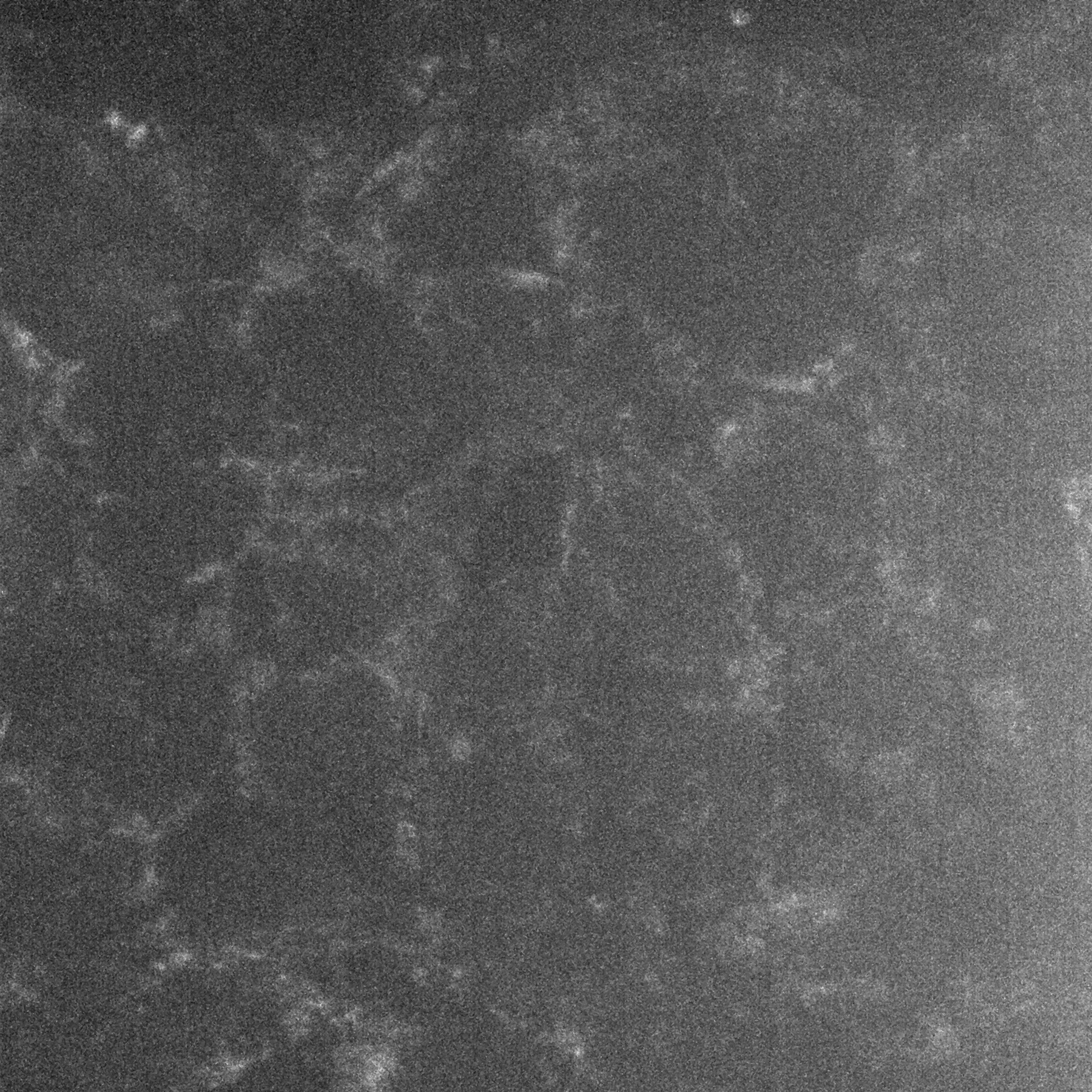

Supplement: Supplementary file 13 — Source data Figure EV1 [file 44318_2024_337_MOESM13_ESM.zip › 07_Figure_EV1/E/Imaging/ZEO_CTRL/_FULL-RANGE-ZEO_CTRL.tif]

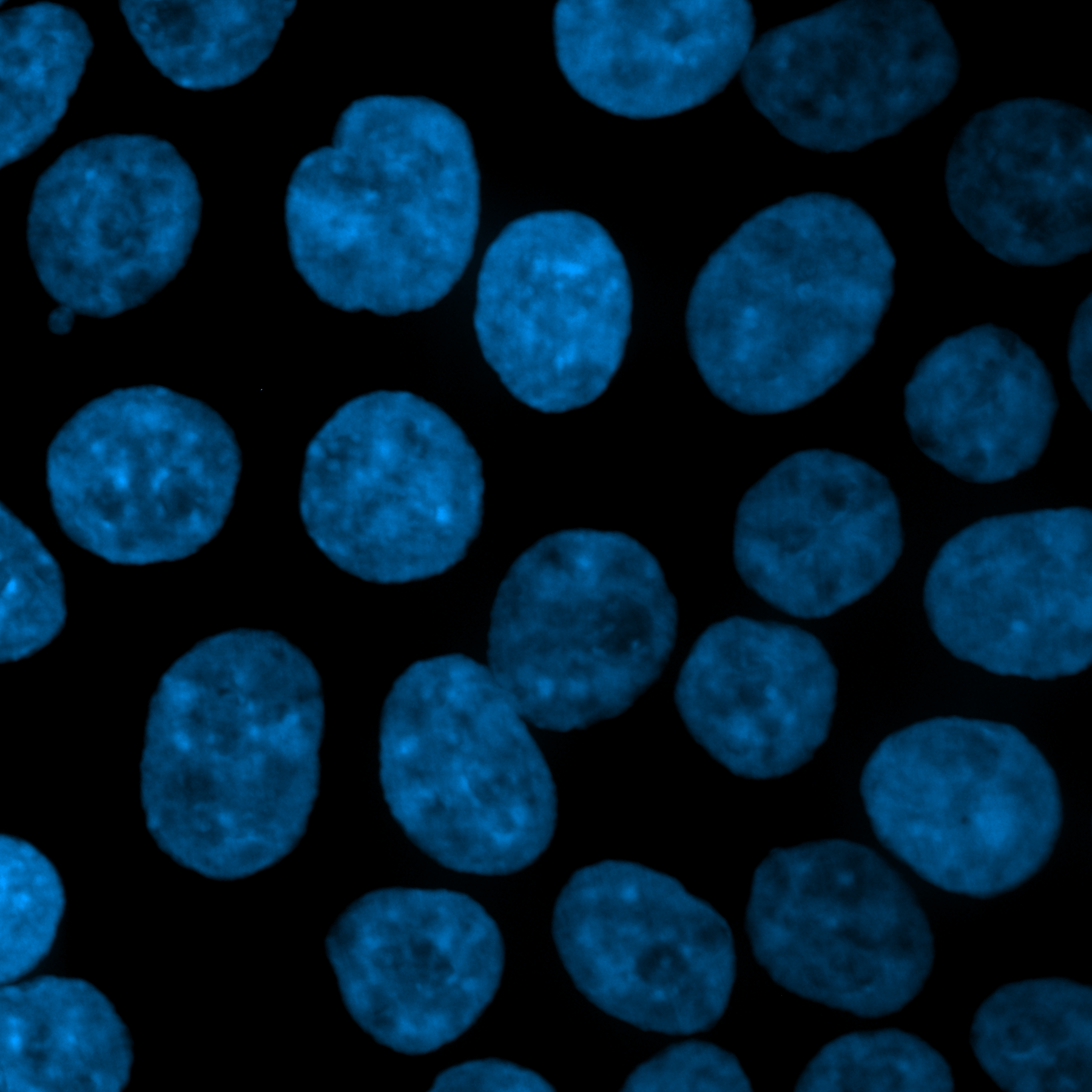

Supplement: Supplementary file 13 — Source data Figure EV1 [file 44318_2024_337_MOESM13_ESM.zip › 07_Figure_EV1/E/Imaging/ZEO_SEL/ZEO_SEL_DAPI.tif]

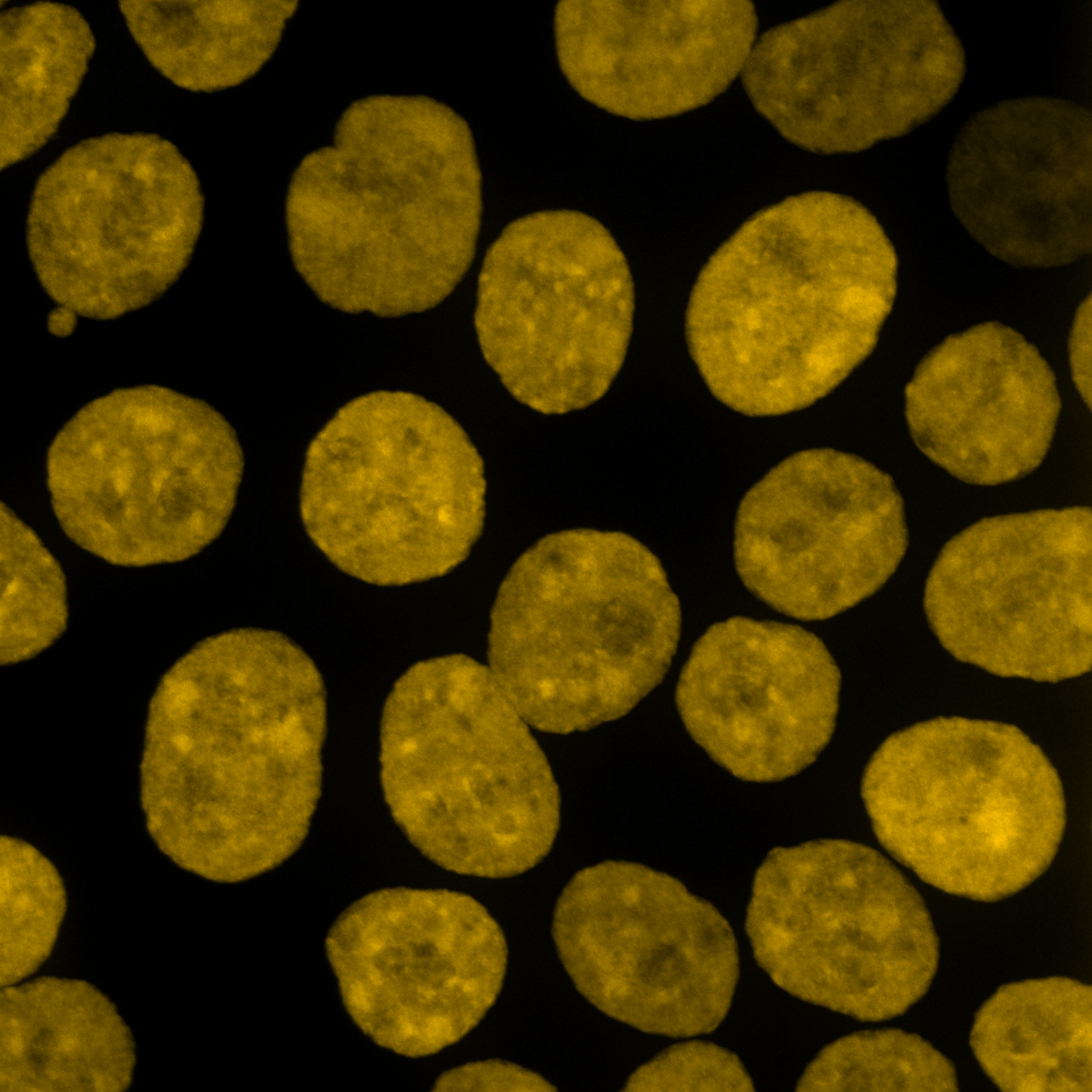

Supplement: Supplementary file 13 — Source data Figure EV1 [file 44318_2024_337_MOESM13_ESM.zip › 07_Figure_EV1/E/Imaging/ZEO_SEL/ZEO_SEL_GFP.tif]

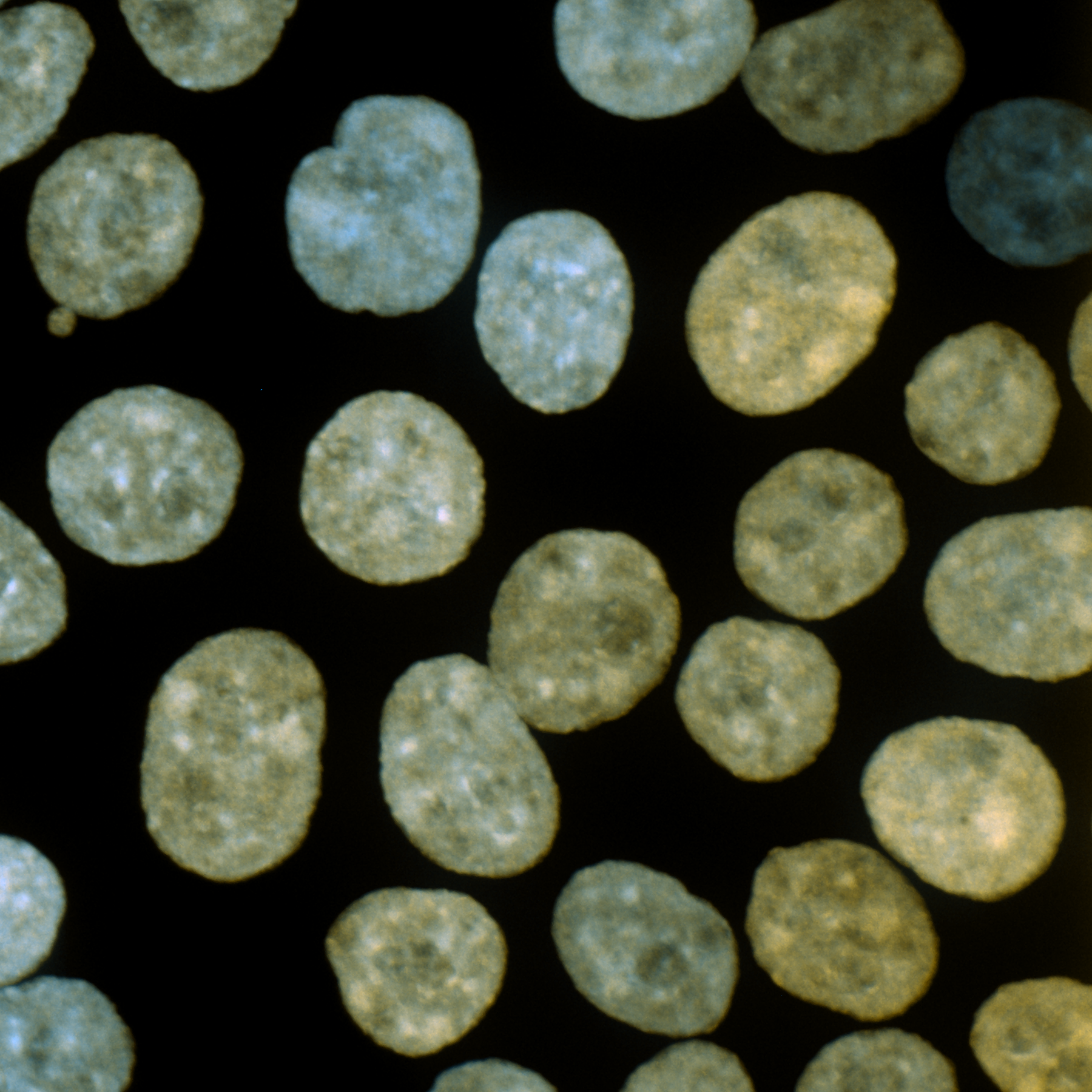

Supplement: Supplementary file 13 — Source data Figure EV1 [file 44318_2024_337_MOESM13_ESM.zip › 07_Figure_EV1/E/Imaging/ZEO_SEL/ZEO_SEL_Merge.tif]

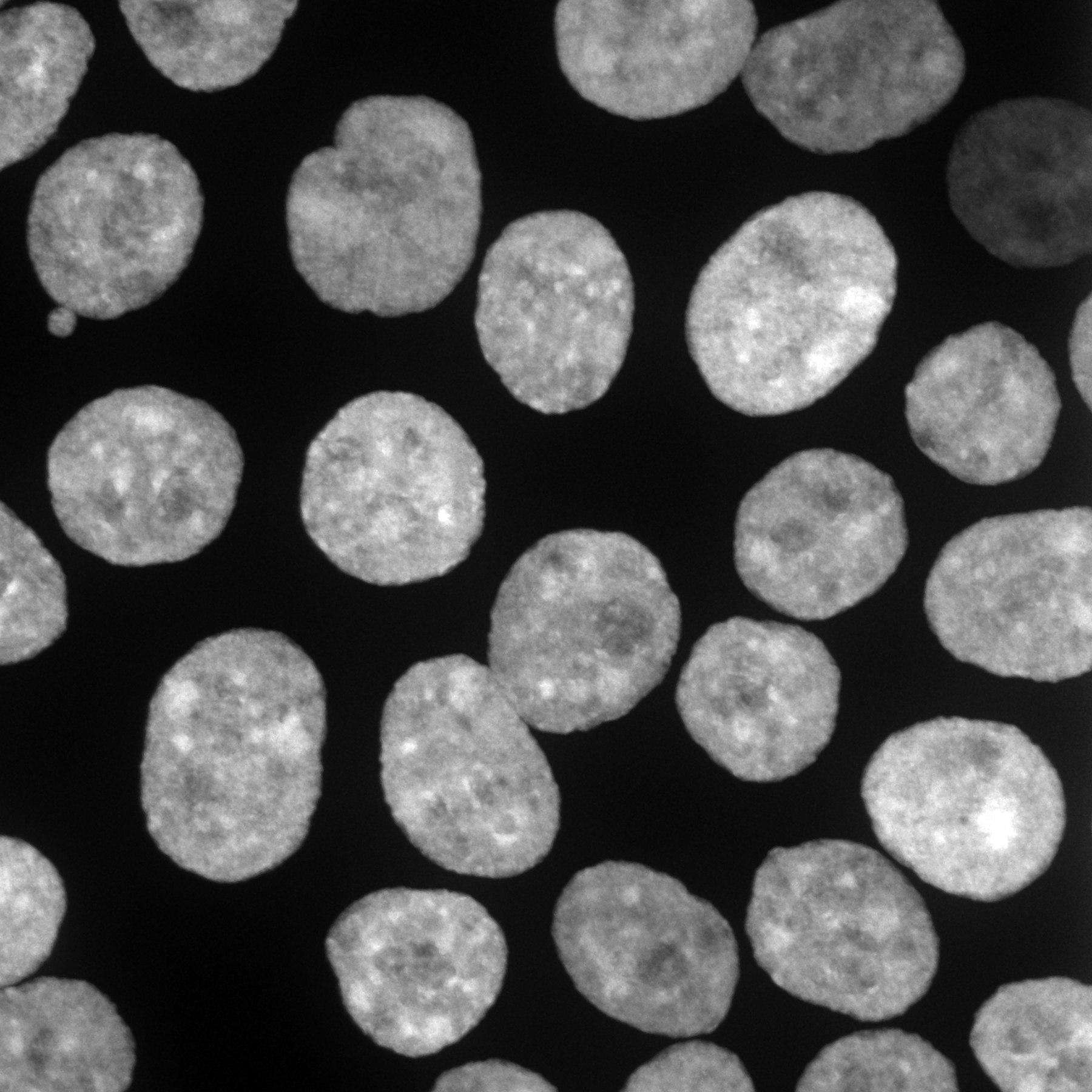

Supplement: Supplementary file 13 — Source data Figure EV1 [file 44318_2024_337_MOESM13_ESM.zip › 07_Figure_EV1/E/Imaging/ZEO_SEL/_FULL-RANGE-ZEO_SEL.tif]

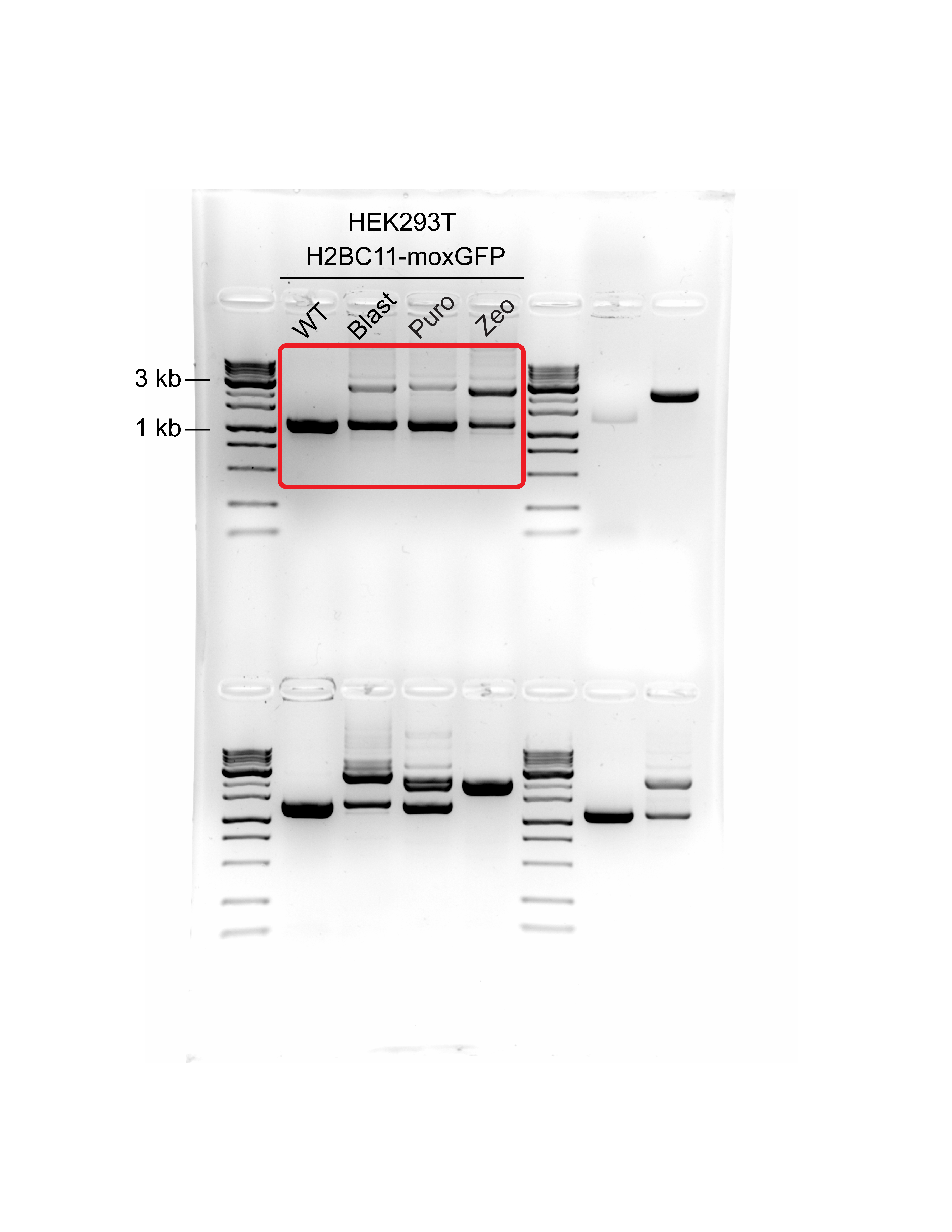

Supplement: Supplementary file 13 — Source data Figure EV1 [file 44318_2024_337_MOESM13_ESM.zip › 07_Figure_EV1/F/Alt-Markers-Selections-Uncropped-Gel.tif]

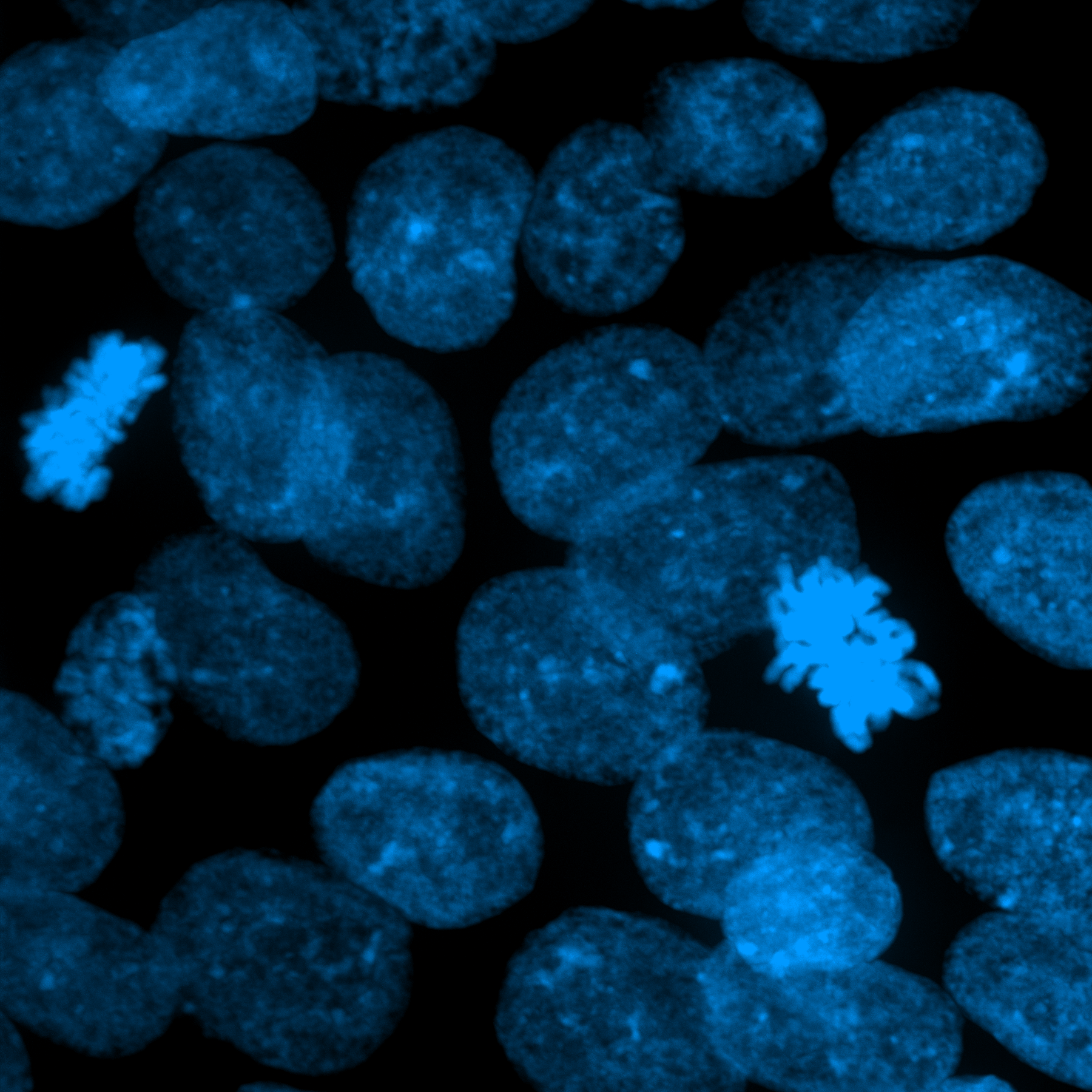

Supplement: Supplementary file 13 — Source data Figure EV1 [file 44318_2024_337_MOESM13_ESM.zip › 07_Figure_EV1/H/TUBA TAG/TUBA TAG_DAPI.tif]

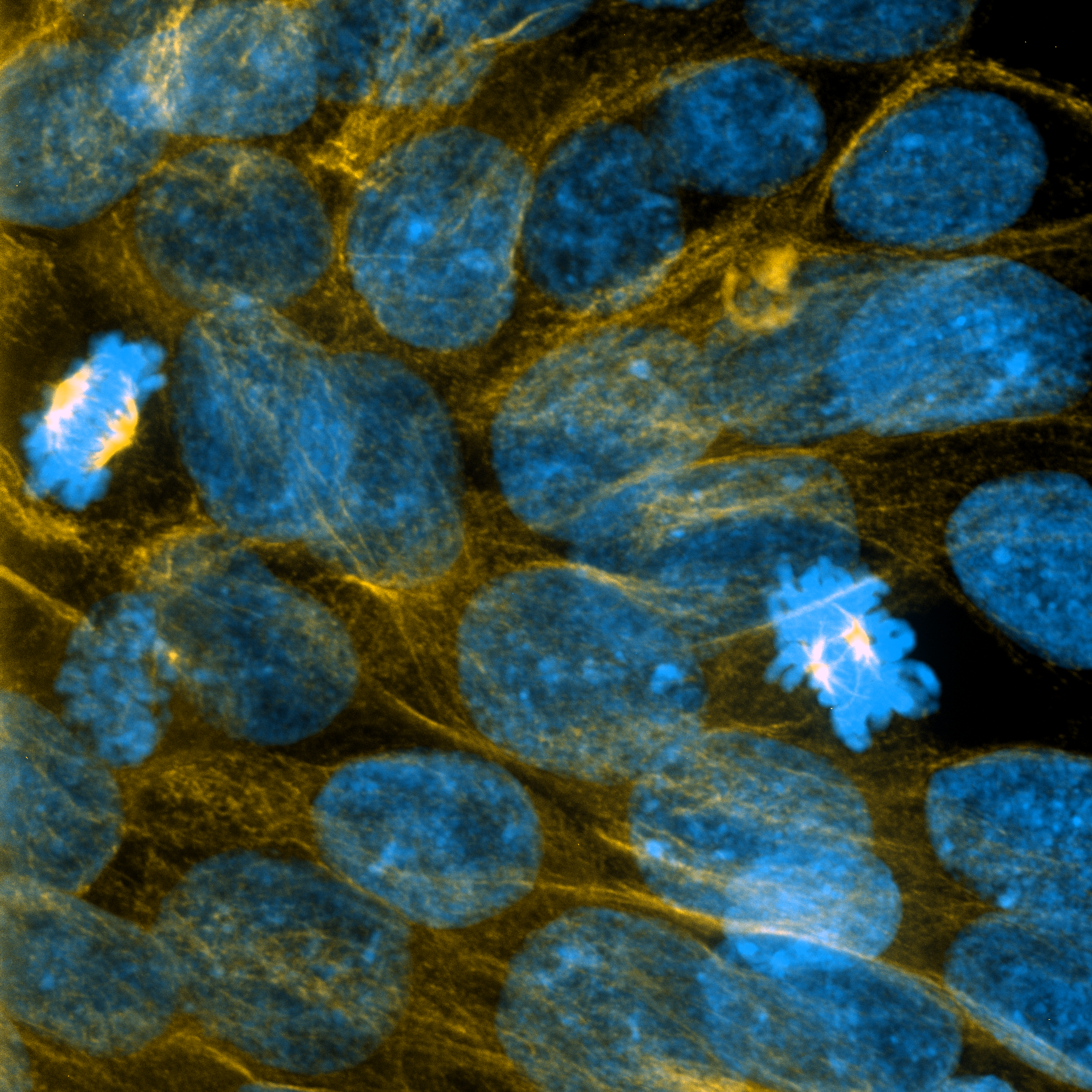

Supplement: Supplementary file 13 — Source data Figure EV1 [file 44318_2024_337_MOESM13_ESM.zip › 07_Figure_EV1/H/TUBA TAG/TUBA TAG_Merge.tif]

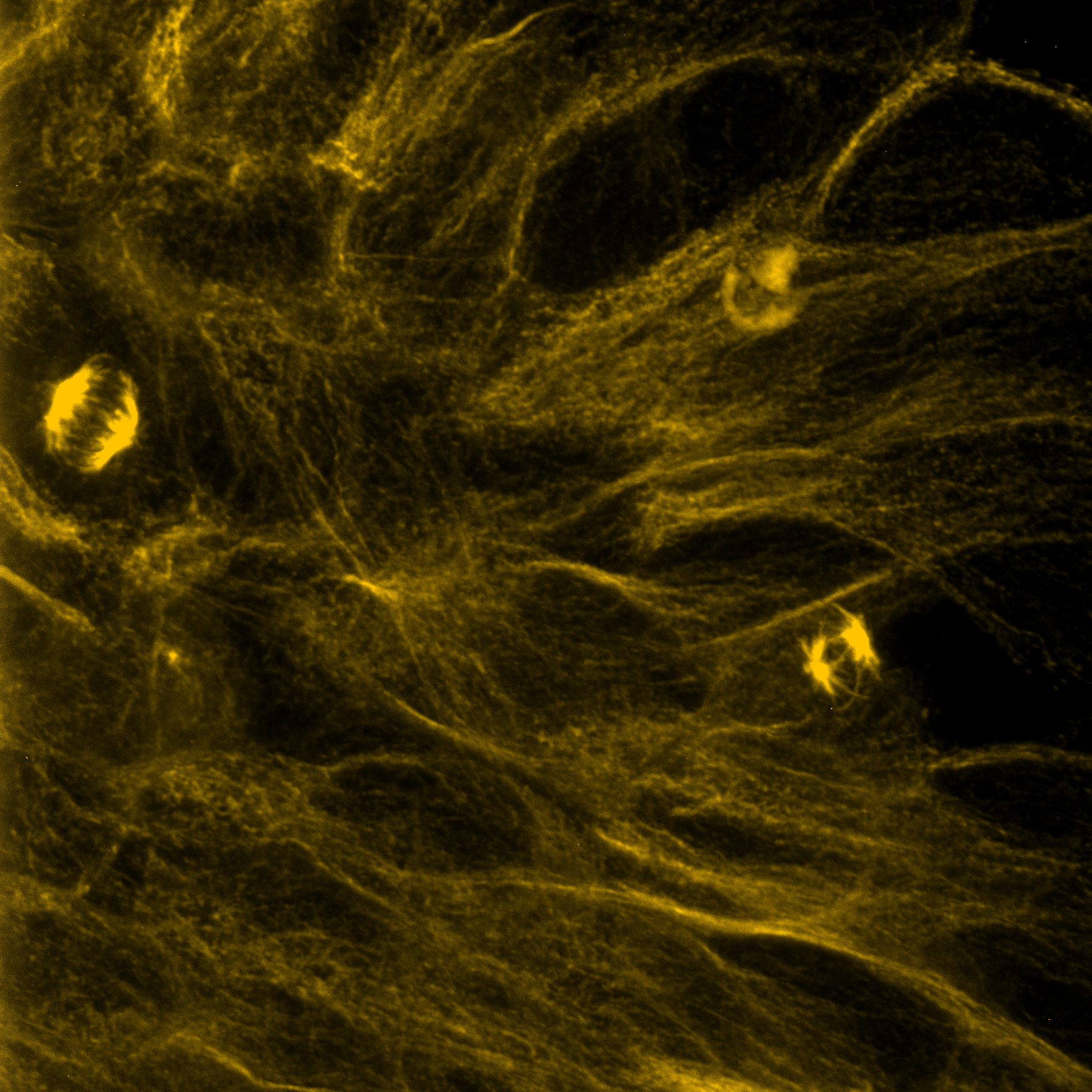

Supplement: Supplementary file 13 — Source data Figure EV1 [file 44318_2024_337_MOESM13_ESM.zip › 07_Figure_EV1/H/TUBA TAG/TUBA TAG_mScarlet.tif]

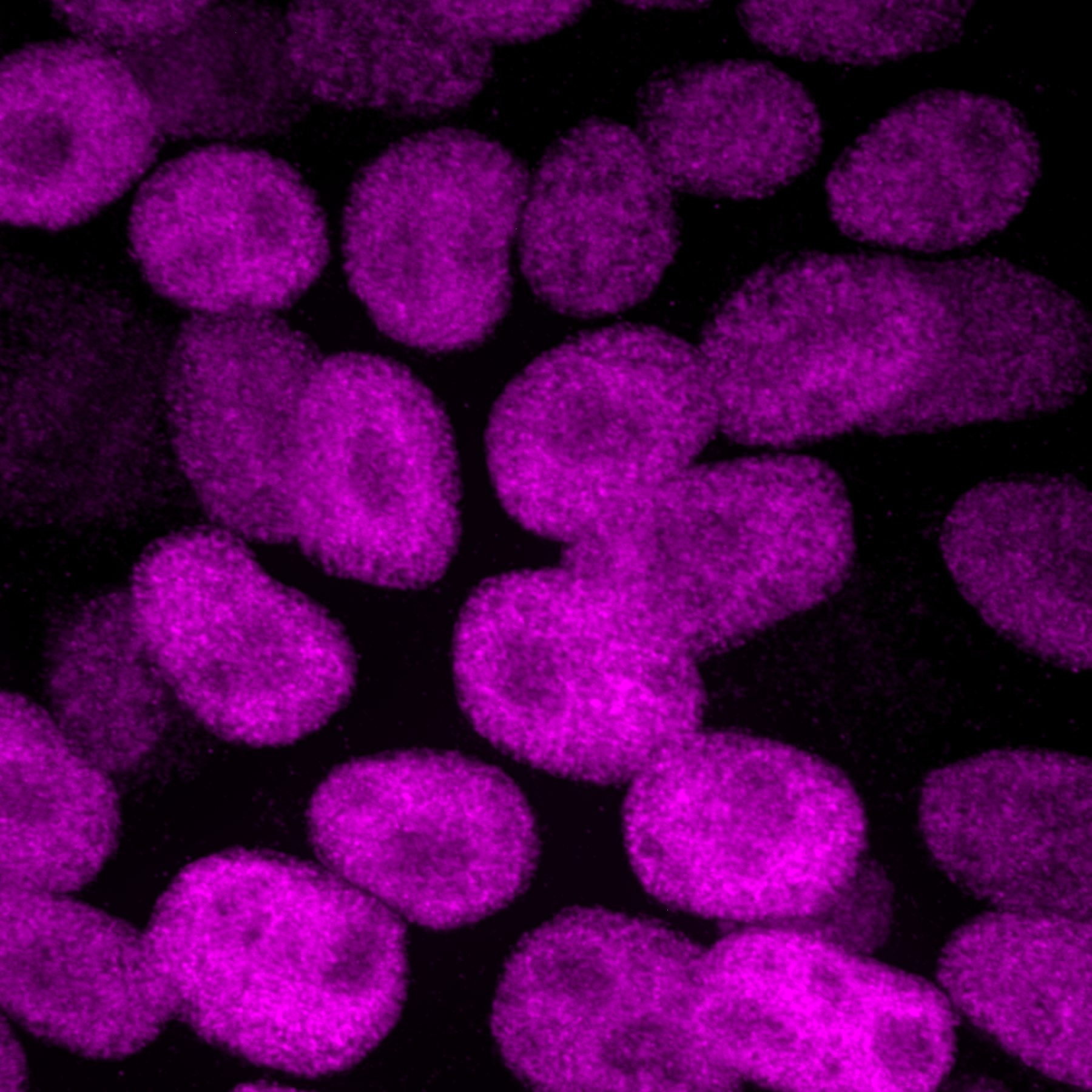

Supplement: Supplementary file 13 — Source data Figure EV1 [file 44318_2024_337_MOESM13_ESM.zip › 07_Figure_EV1/H/TUBA TAG/TUBA TAG_OCT4.tif]

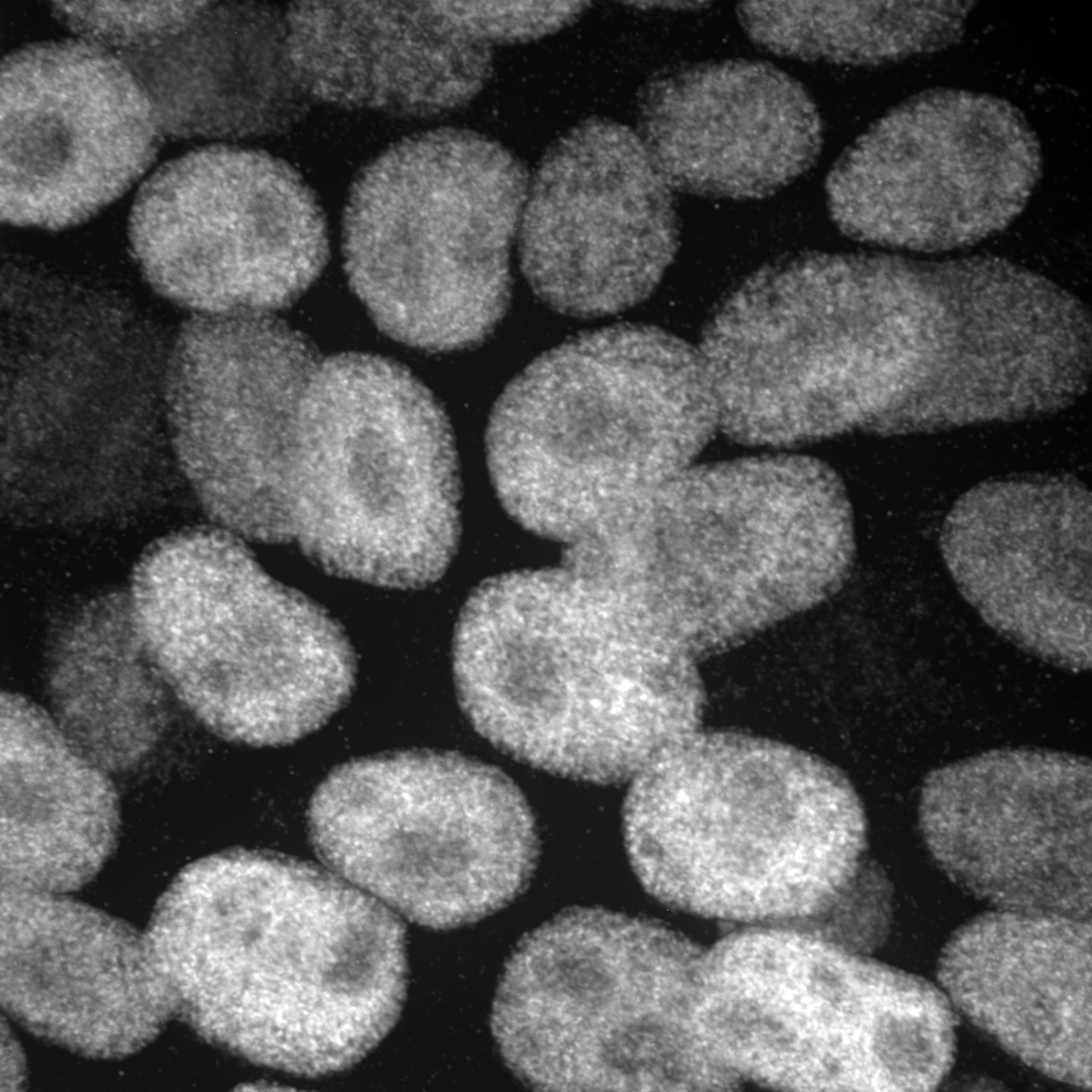

Supplement: Supplementary file 13 — Source data Figure EV1 [file 44318_2024_337_MOESM13_ESM.zip › 07_Figure_EV1/H/TUBA TAG/_FULL-RANGE-TUBA TAG.tif]

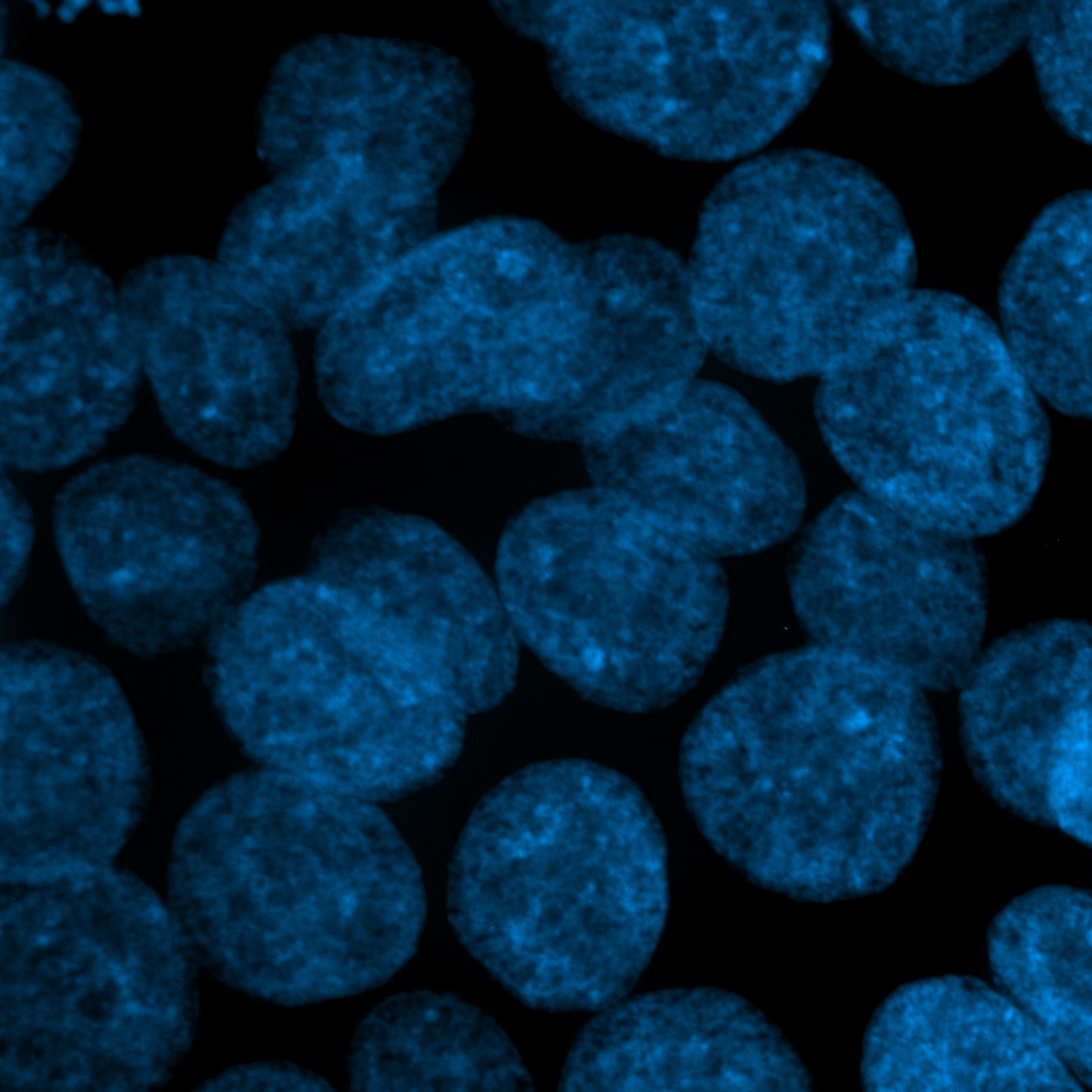

Supplement: Supplementary file 13 — Source data Figure EV1 [file 44318_2024_337_MOESM13_ESM.zip › 07_Figure_EV1/H/TUBA WT/TUBA WT_DAPI.tif]

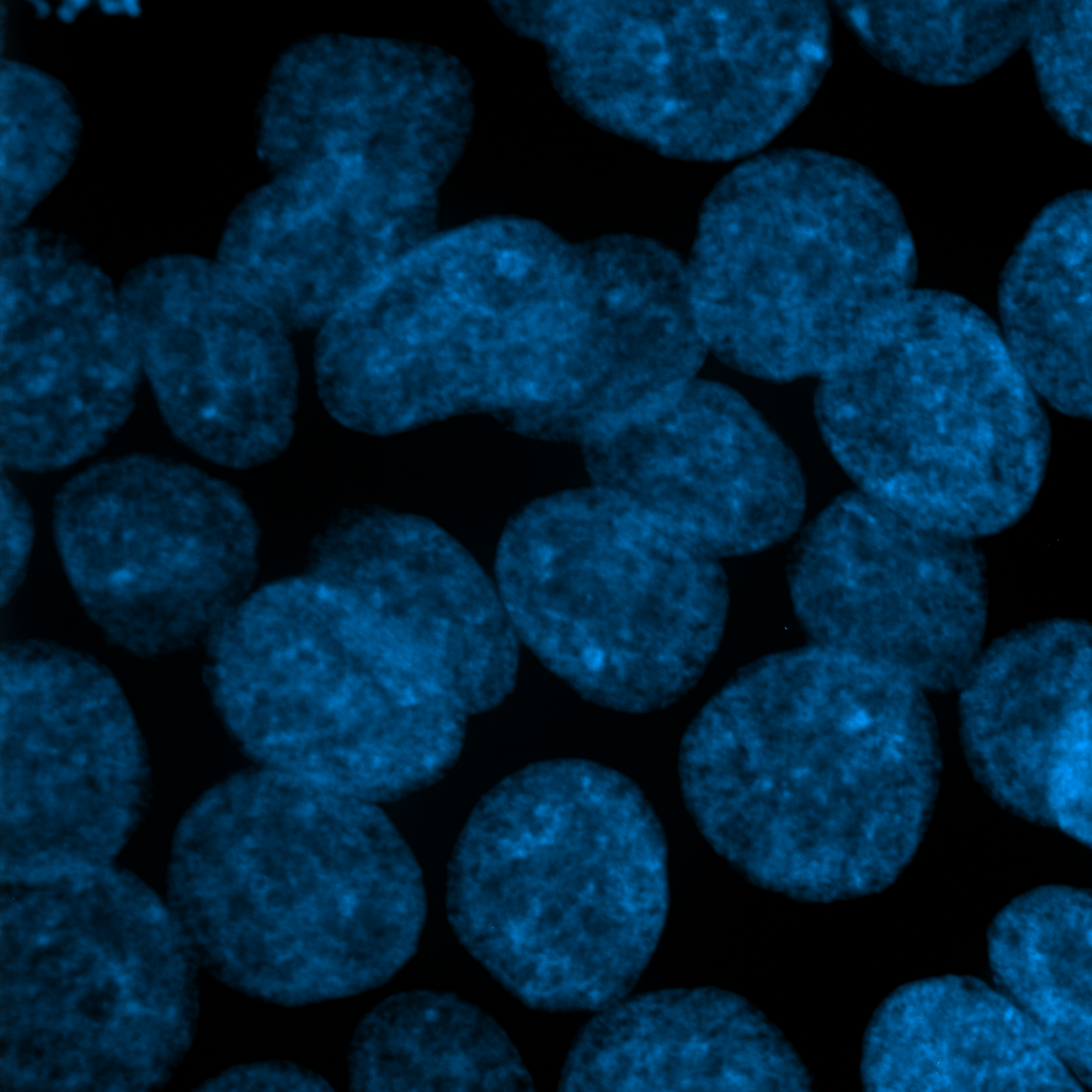

Supplement: Supplementary file 13 — Source data Figure EV1 [file 44318_2024_337_MOESM13_ESM.zip › 07_Figure_EV1/H/TUBA WT/TUBA WT_Merge.tif]

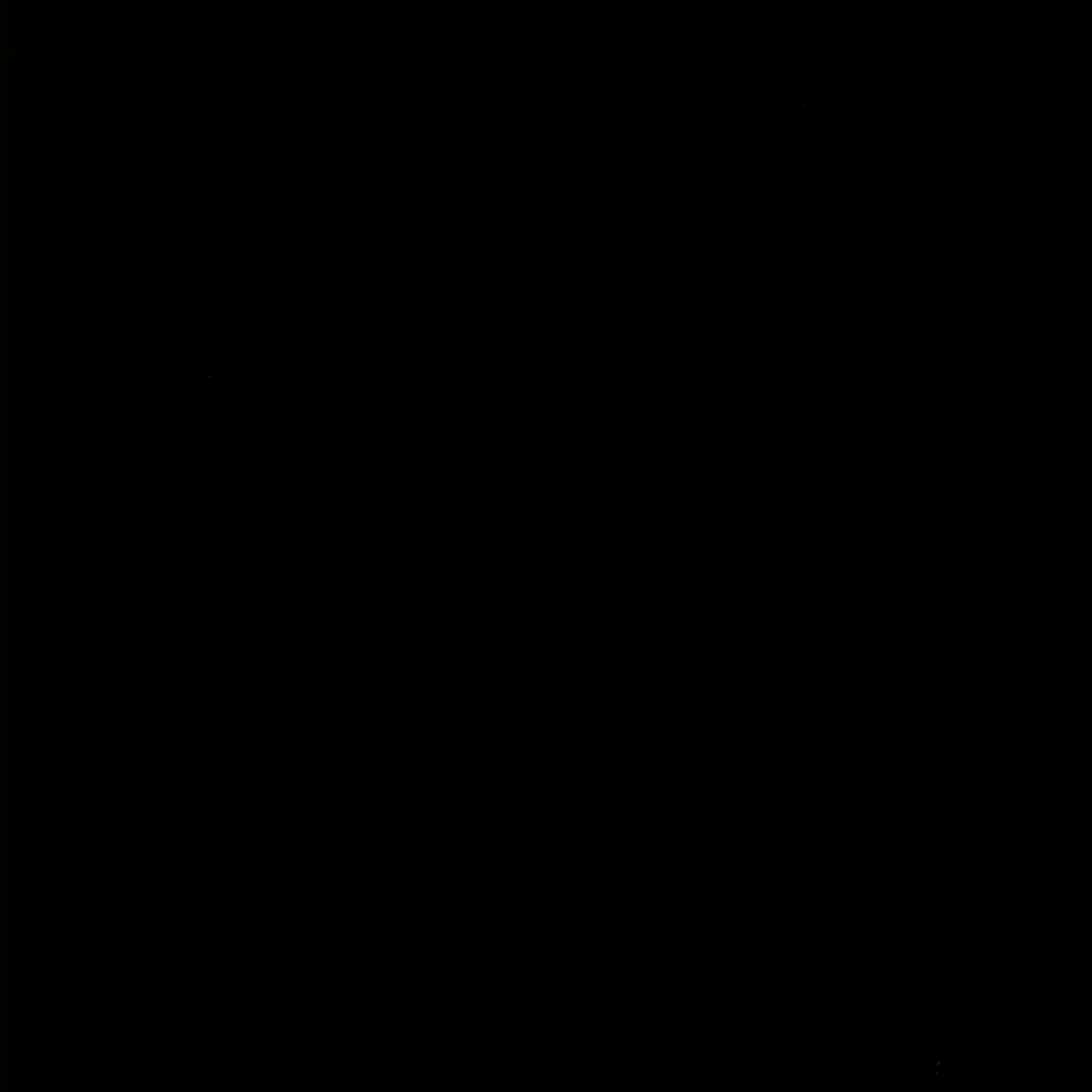

Supplement: Supplementary file 13 — Source data Figure EV1 [file 44318_2024_337_MOESM13_ESM.zip › 07_Figure_EV1/H/TUBA WT/TUBA WT_mScarlet.tif]

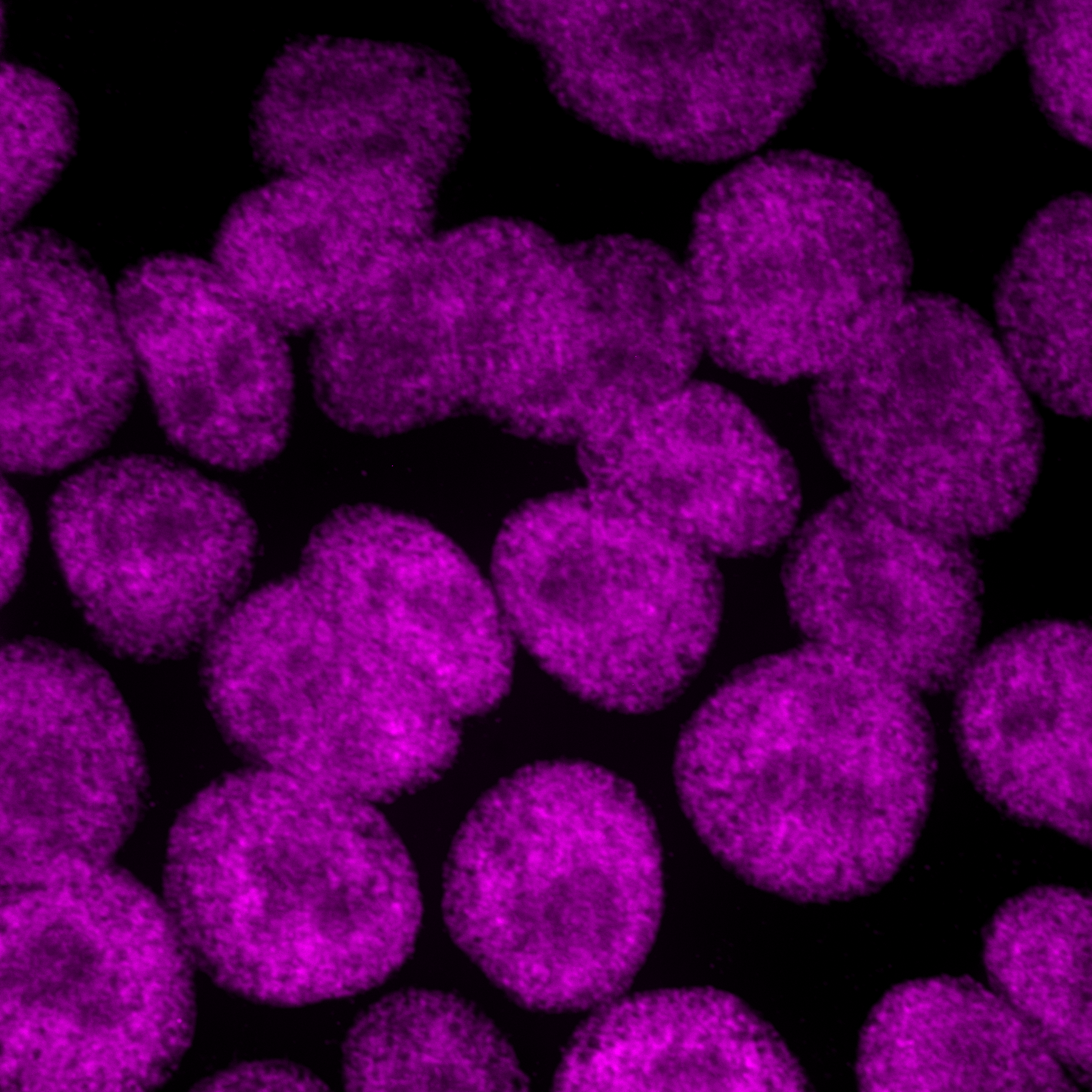

Supplement: Supplementary file 13 — Source data Figure EV1 [file 44318_2024_337_MOESM13_ESM.zip › 07_Figure_EV1/H/TUBA WT/TUBA WT_OCT4.tif]

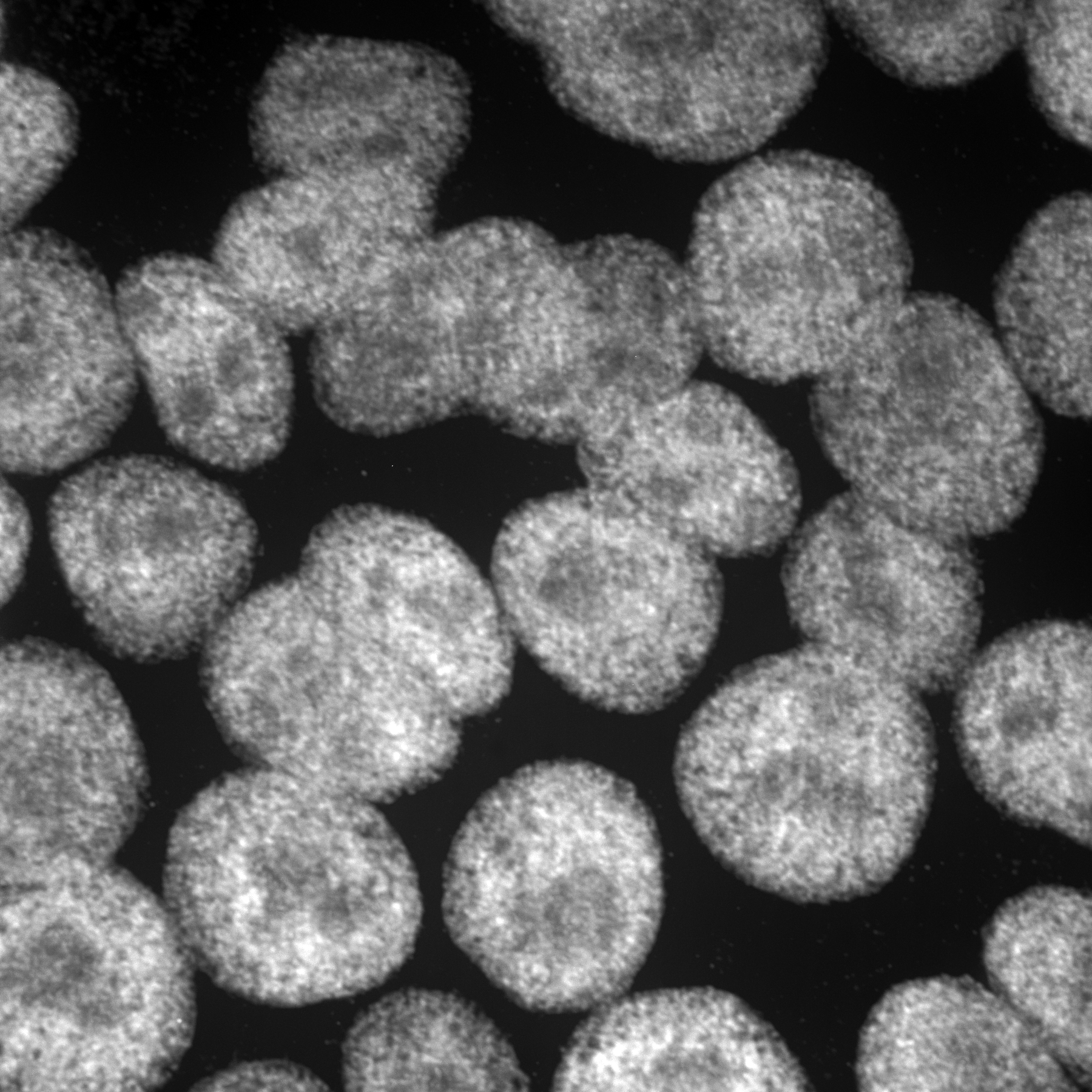

Supplement: Supplementary file 13 — Source data Figure EV1 [file 44318_2024_337_MOESM13_ESM.zip › 07_Figure_EV1/H/TUBA WT/_FULL-RANGE-TUBA WT.tif]

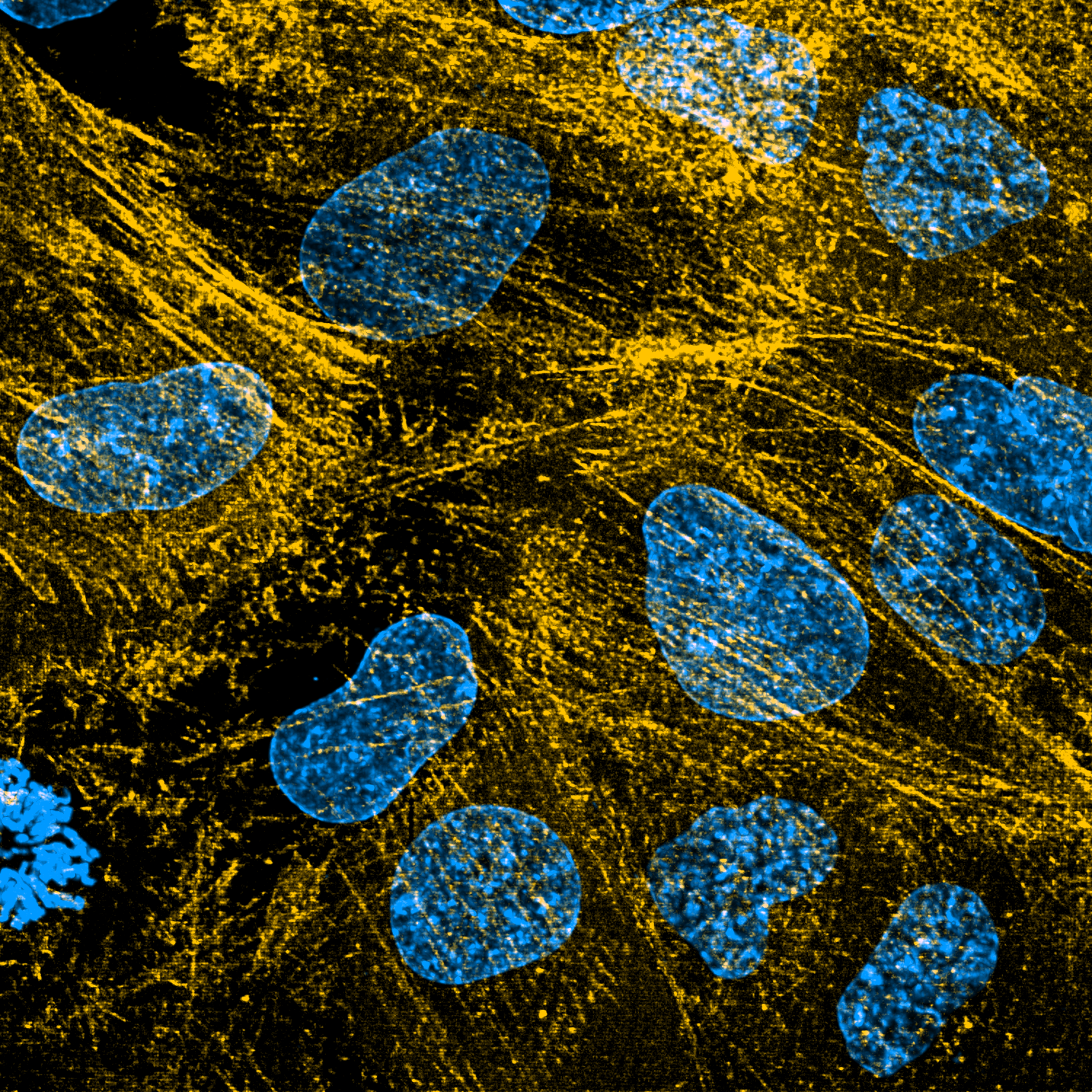

Supplement: Supplementary file 14 — Source data Figure EV2 [file 44318_2024_337_MOESM14_ESM.zip › 08_Figure_EV2/A/FLUO-ACTB/FLUO-ACTB_Merge.tif]

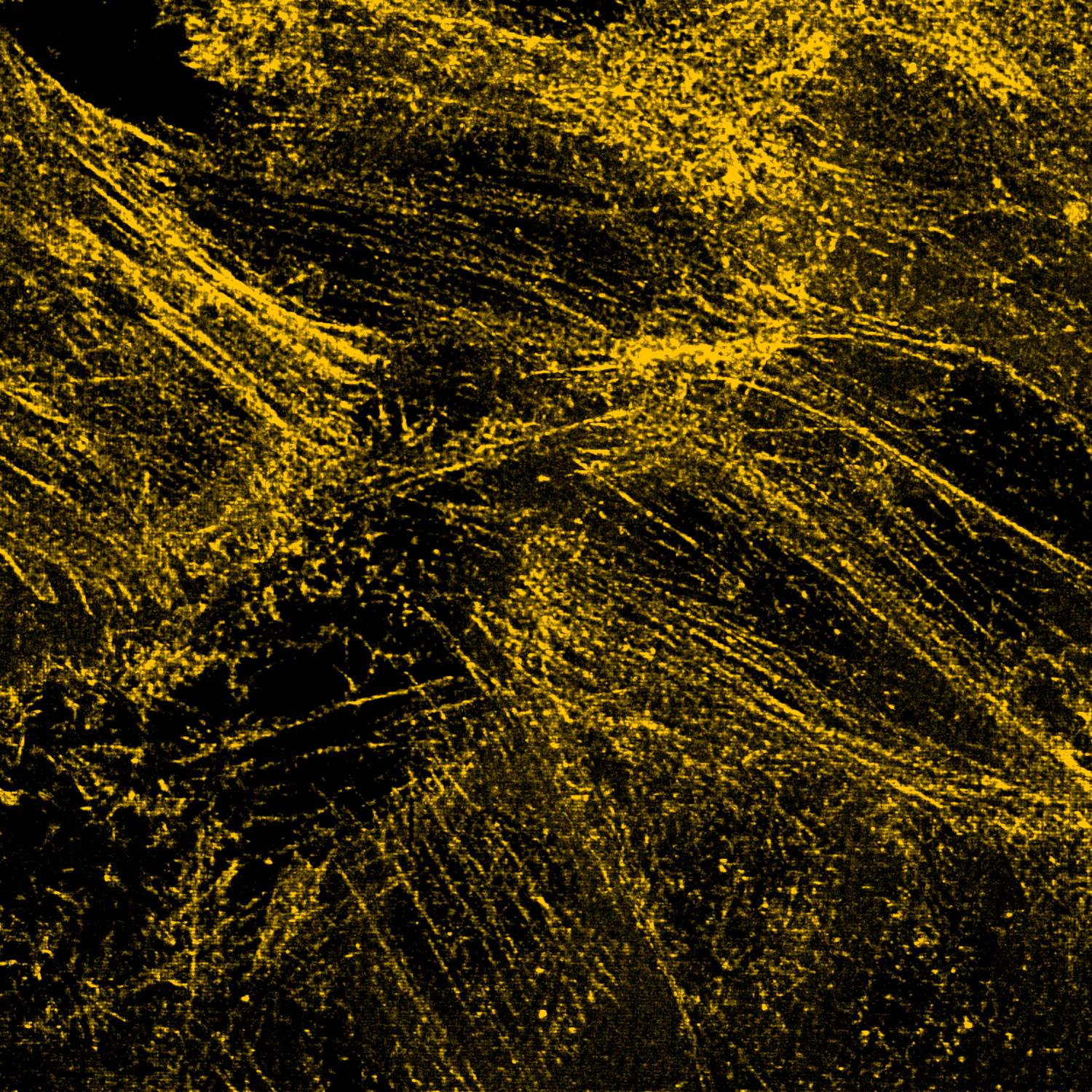

Supplement: Supplementary file 14 — Source data Figure EV2 [file 44318_2024_337_MOESM14_ESM.zip › 08_Figure_EV2/A/FLUO-ACTB/FLUO-ACTB_mStayGold.tif]

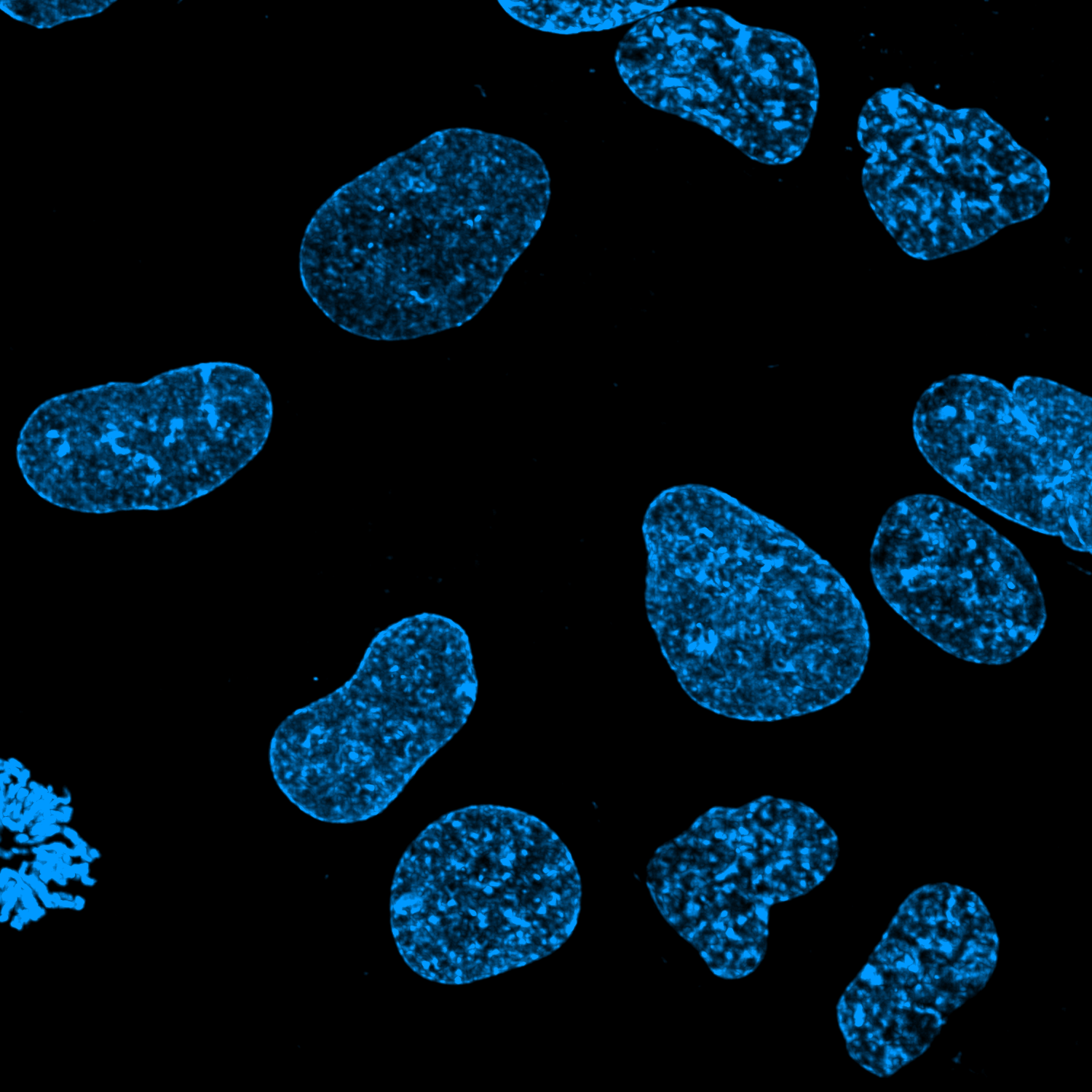

Supplement: Supplementary file 14 — Source data Figure EV2 [file 44318_2024_337_MOESM14_ESM.zip › 08_Figure_EV2/A/FLUO-ACTB/FLUO-ACTB_SiR-DNA.tif]

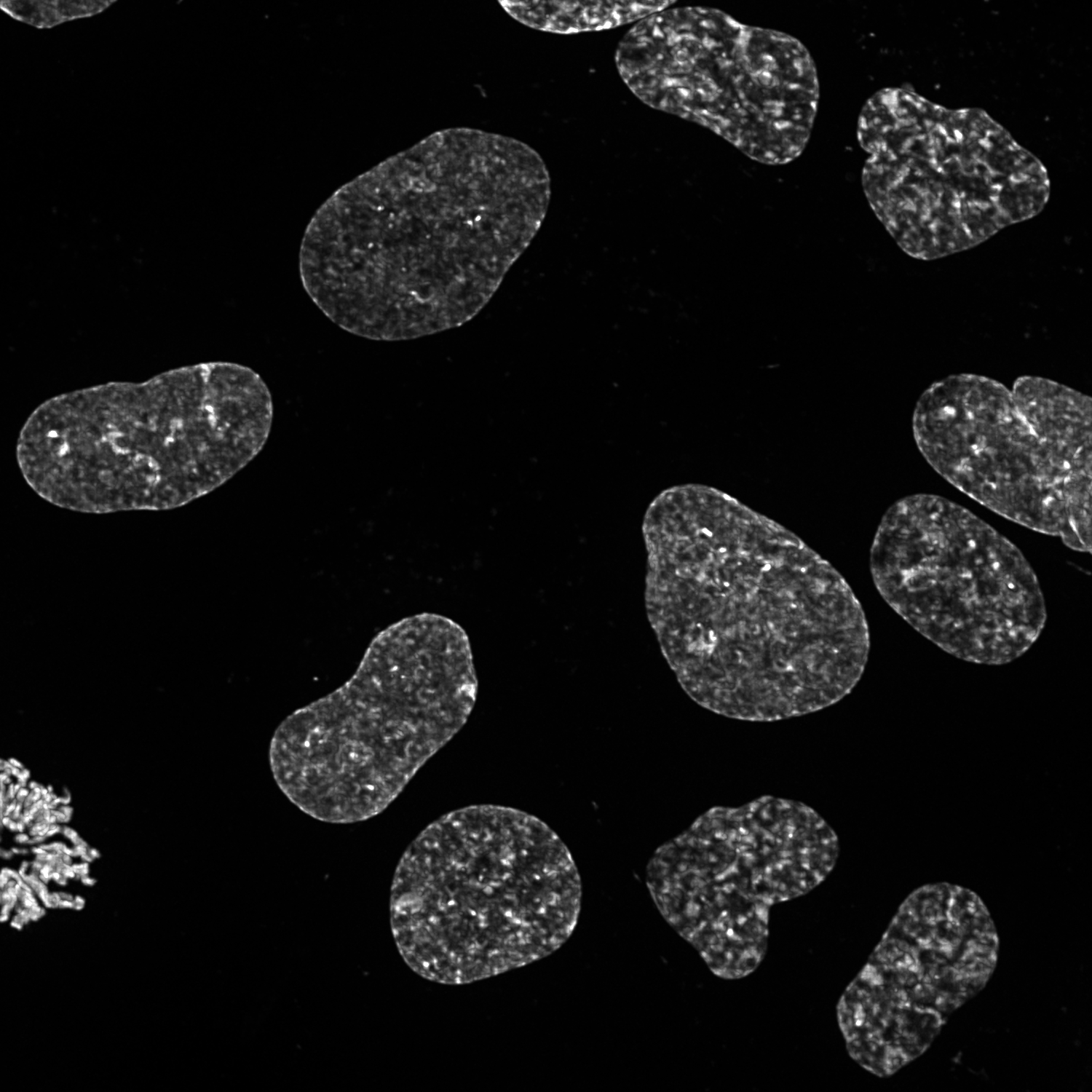

Supplement: Supplementary file 14 — Source data Figure EV2 [file 44318_2024_337_MOESM14_ESM.zip › 08_Figure_EV2/A/FLUO-ACTB/_FULL-RANGE-FLUO-ACTB.tif]

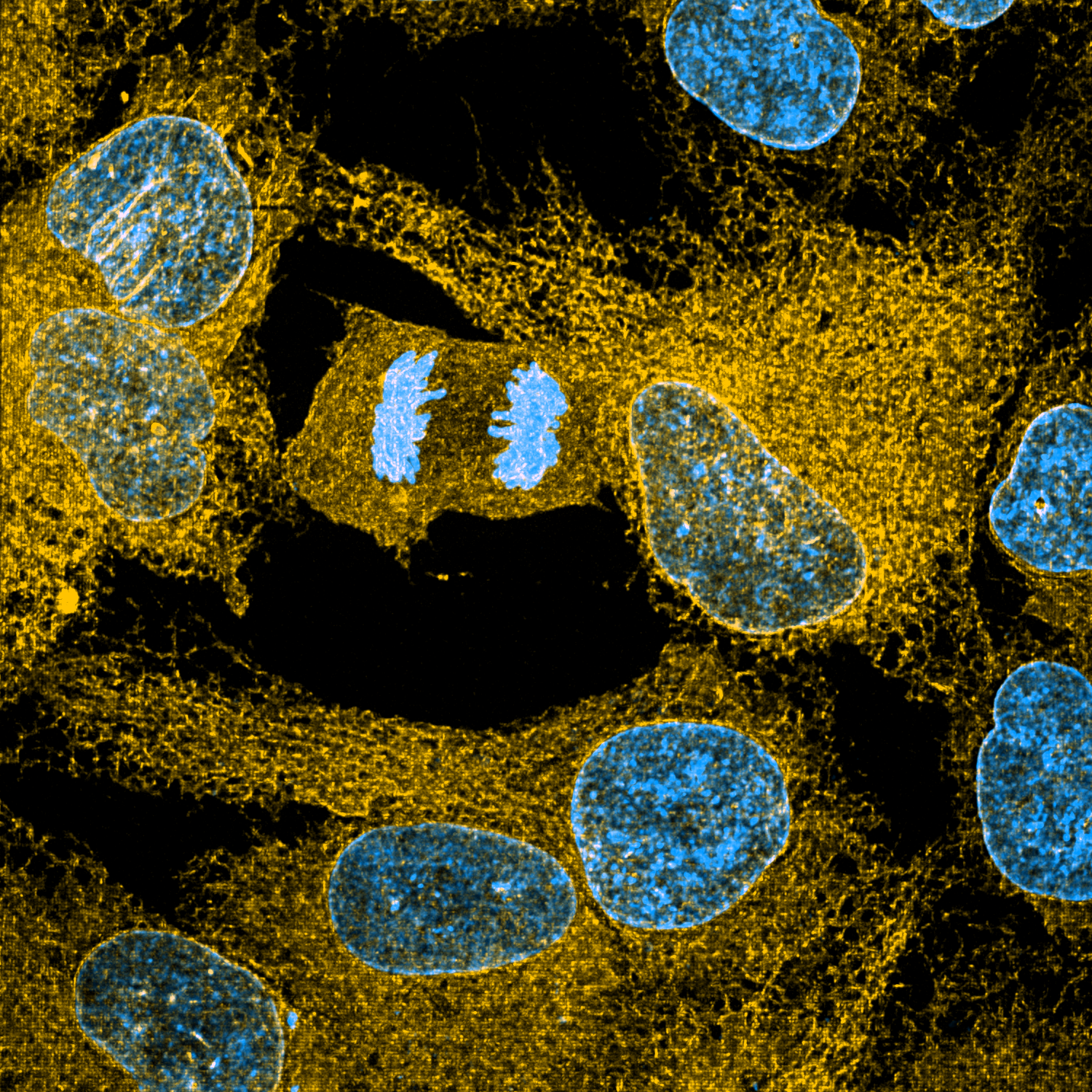

Supplement: Supplementary file 14 — Source data Figure EV2 [file 44318_2024_337_MOESM14_ESM.zip › 08_Figure_EV2/A/FLUO-CANX/FLUO-CANX_Merge.tif]

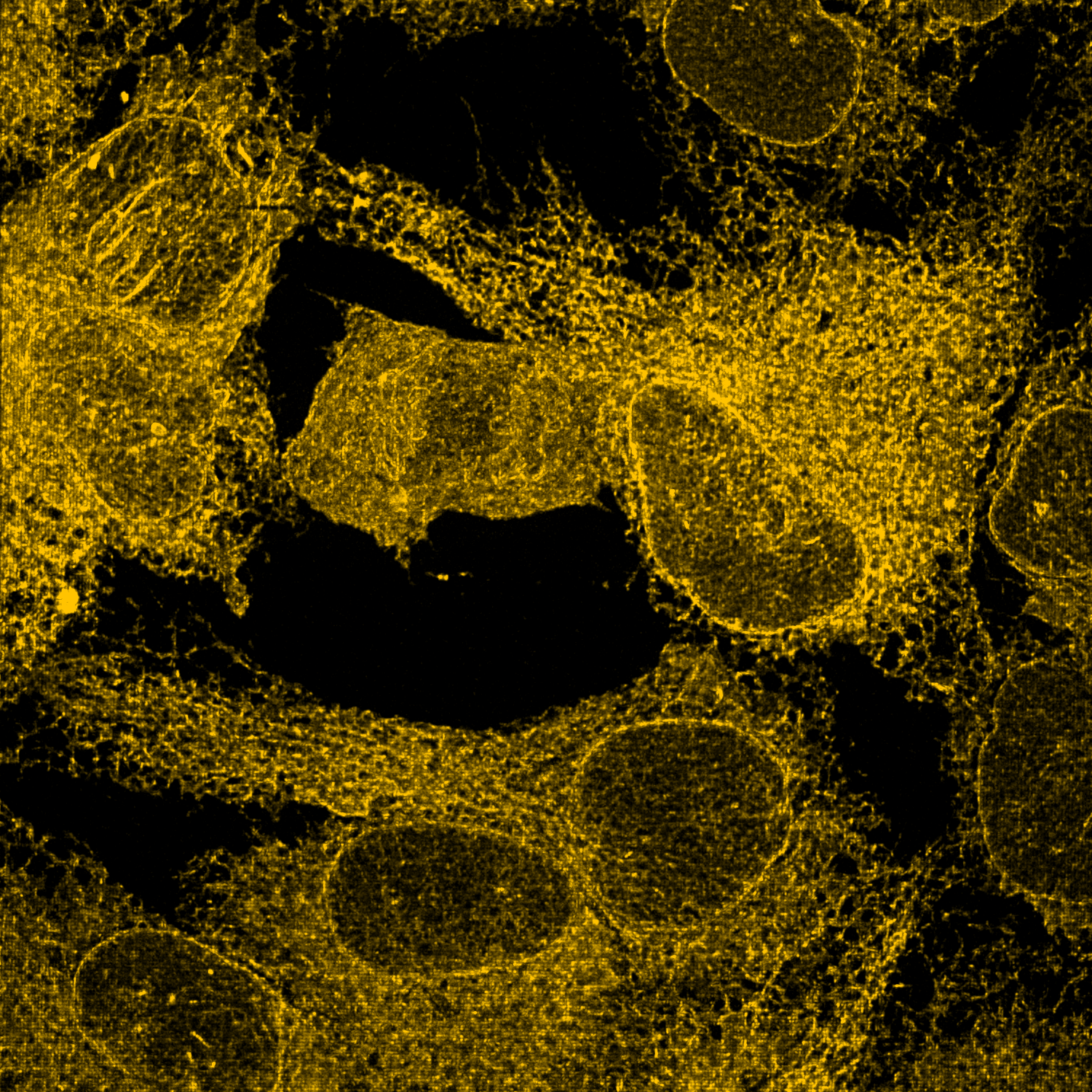

Supplement: Supplementary file 14 — Source data Figure EV2 [file 44318_2024_337_MOESM14_ESM.zip › 08_Figure_EV2/A/FLUO-CANX/FLUO-CANX_mStayGold.tif]

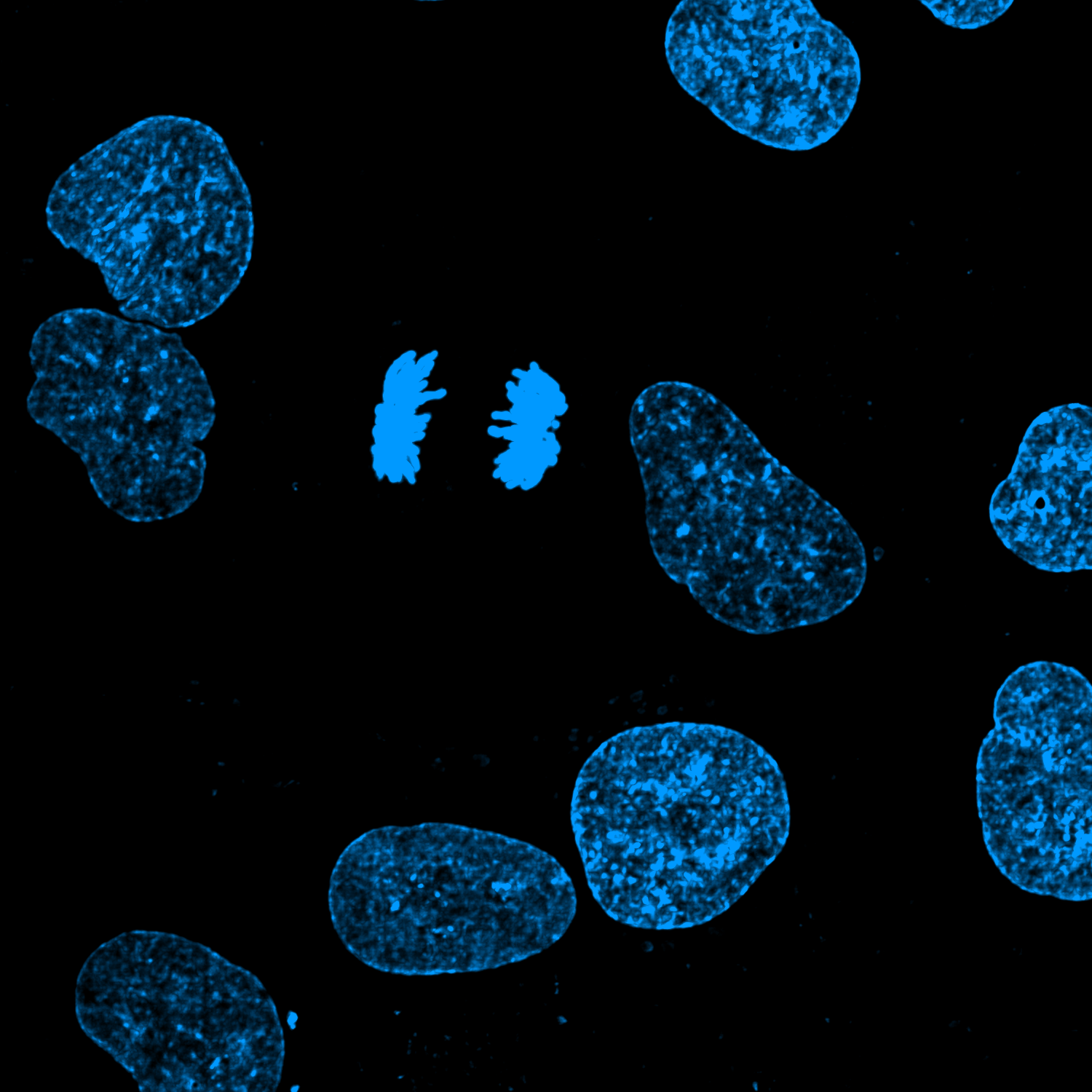

Supplement: Supplementary file 14 — Source data Figure EV2 [file 44318_2024_337_MOESM14_ESM.zip › 08_Figure_EV2/A/FLUO-CANX/FLUO-CANX_SiR-DNA.tif]

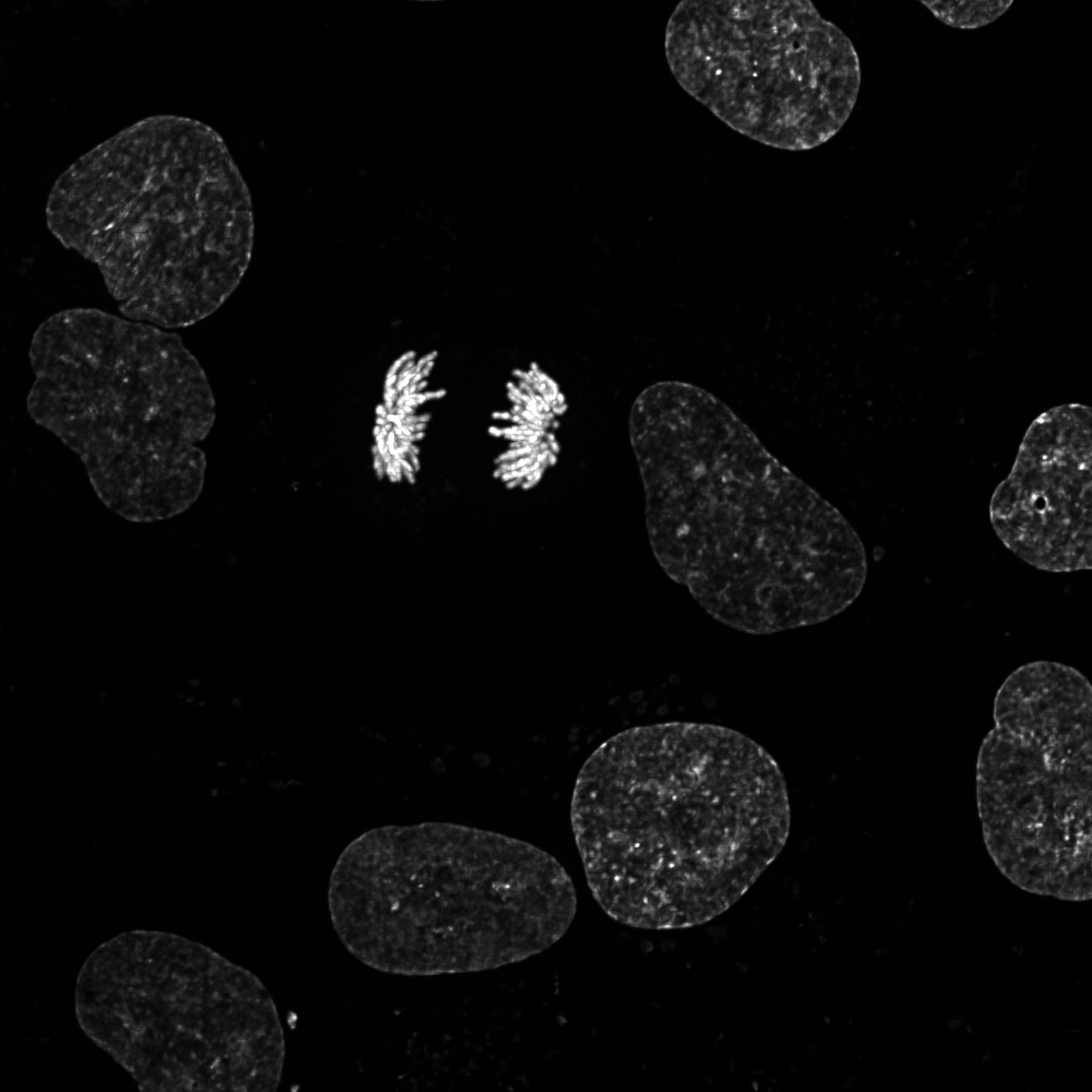

Supplement: Supplementary file 14 — Source data Figure EV2 [file 44318_2024_337_MOESM14_ESM.zip › 08_Figure_EV2/A/FLUO-CANX/_FULL-RANGE-FLUO-CANX.tif]

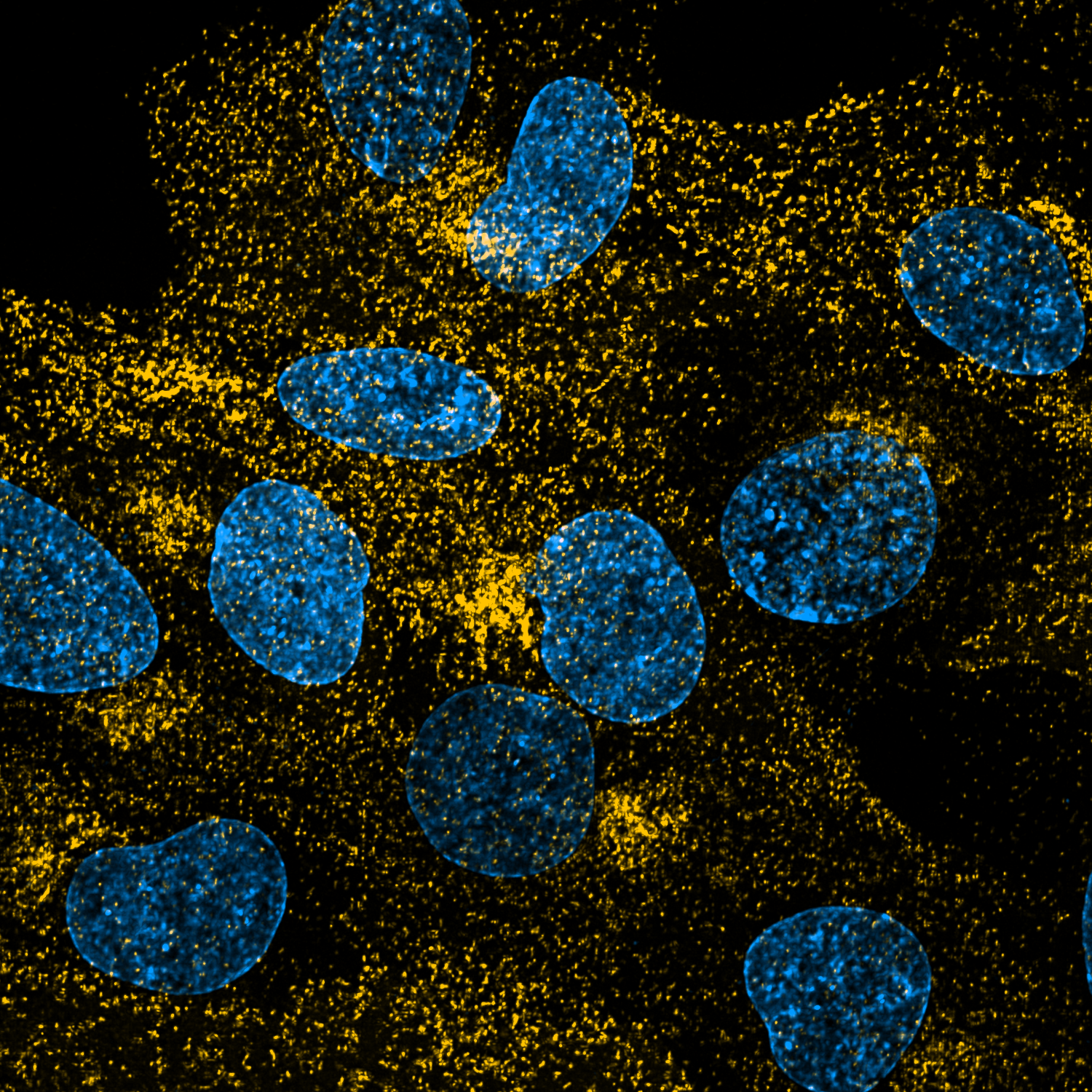

Supplement: Supplementary file 14 — Source data Figure EV2 [file 44318_2024_337_MOESM14_ESM.zip › 08_Figure_EV2/A/FLUO-CLTC/FLUO-CLTC_Merge.tif]

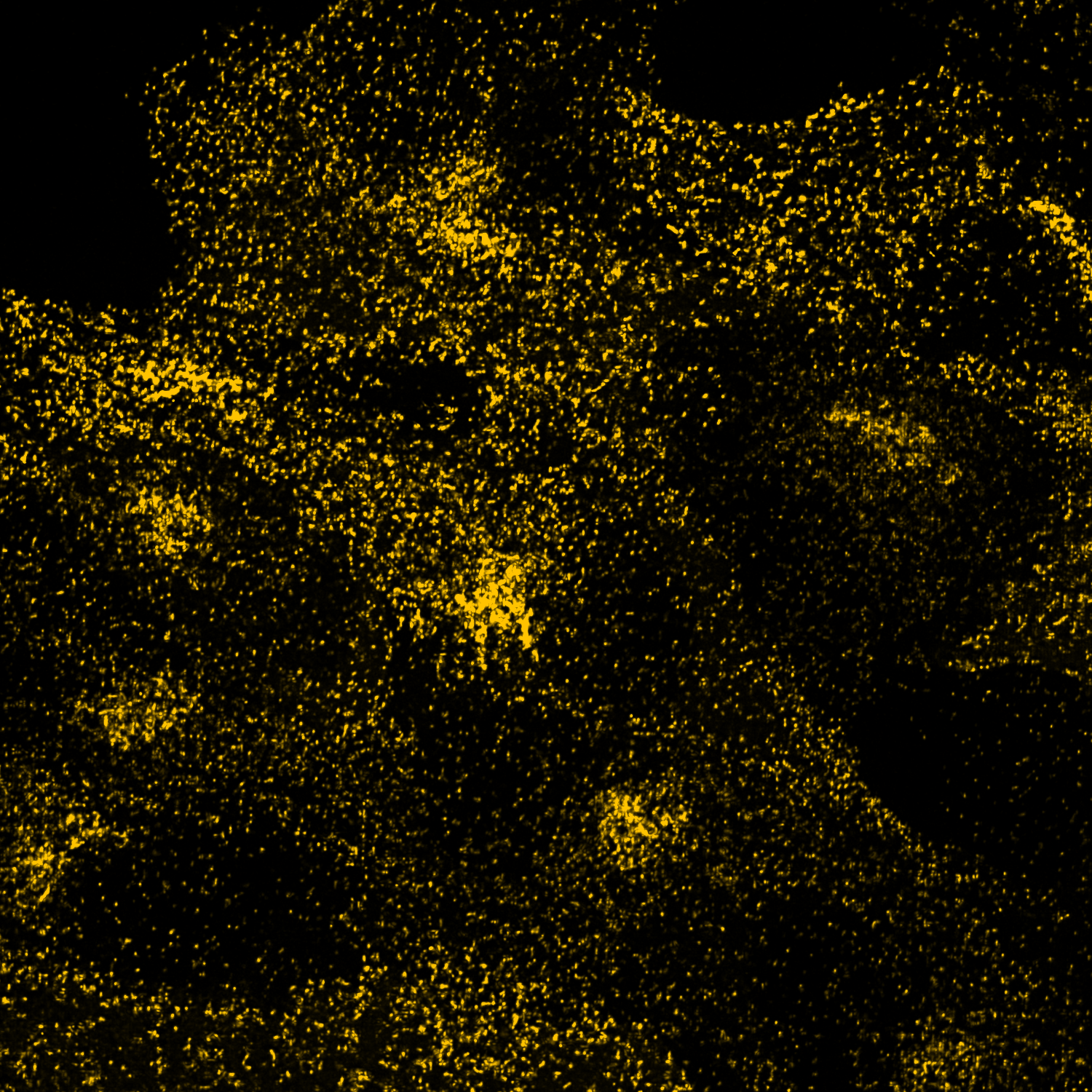

Supplement: Supplementary file 14 — Source data Figure EV2 [file 44318_2024_337_MOESM14_ESM.zip › 08_Figure_EV2/A/FLUO-CLTC/FLUO-CLTC_mStayGold.tif]

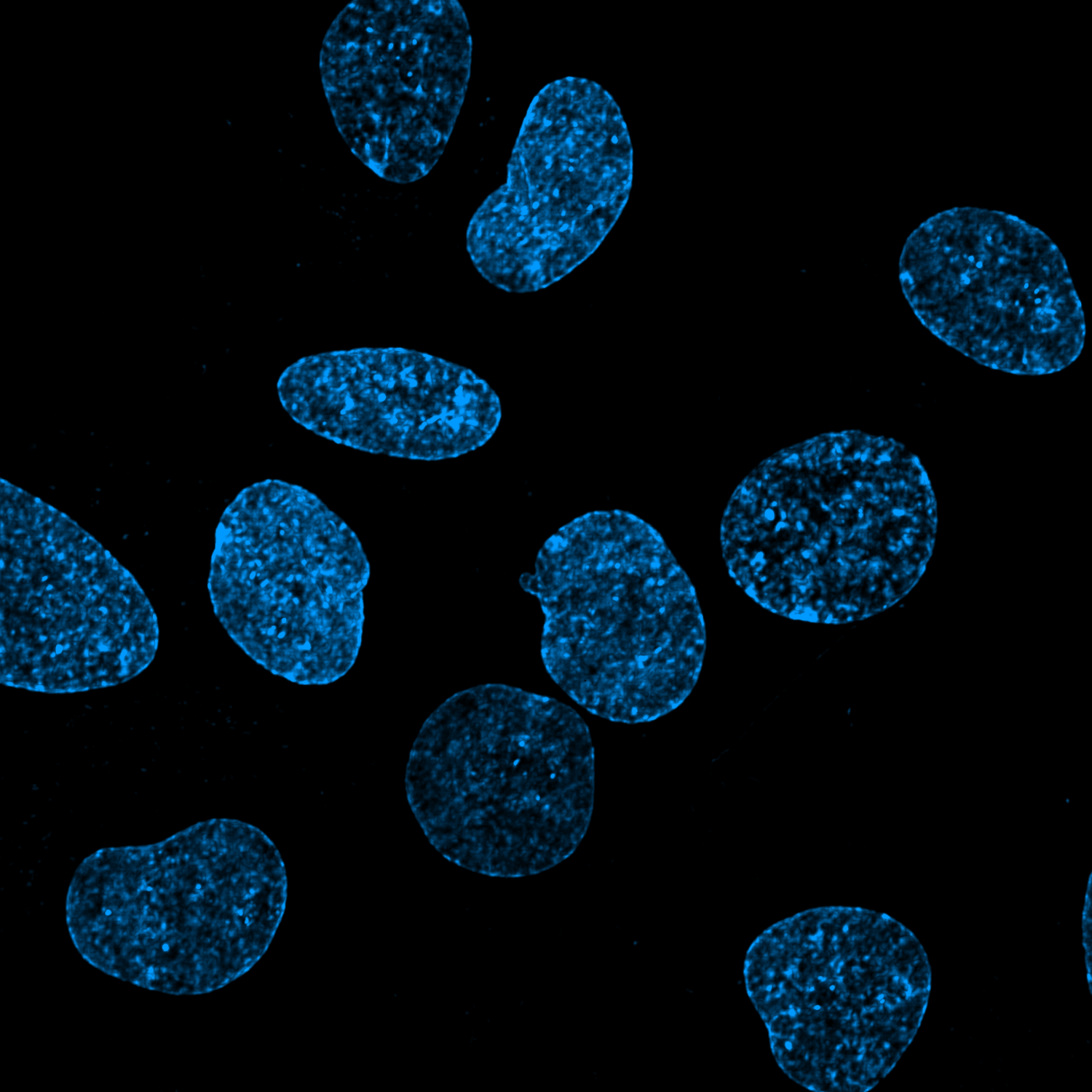

Supplement: Supplementary file 14 — Source data Figure EV2 [file 44318_2024_337_MOESM14_ESM.zip › 08_Figure_EV2/A/FLUO-CLTC/FLUO-CLTC_SiR-DNA.tif]

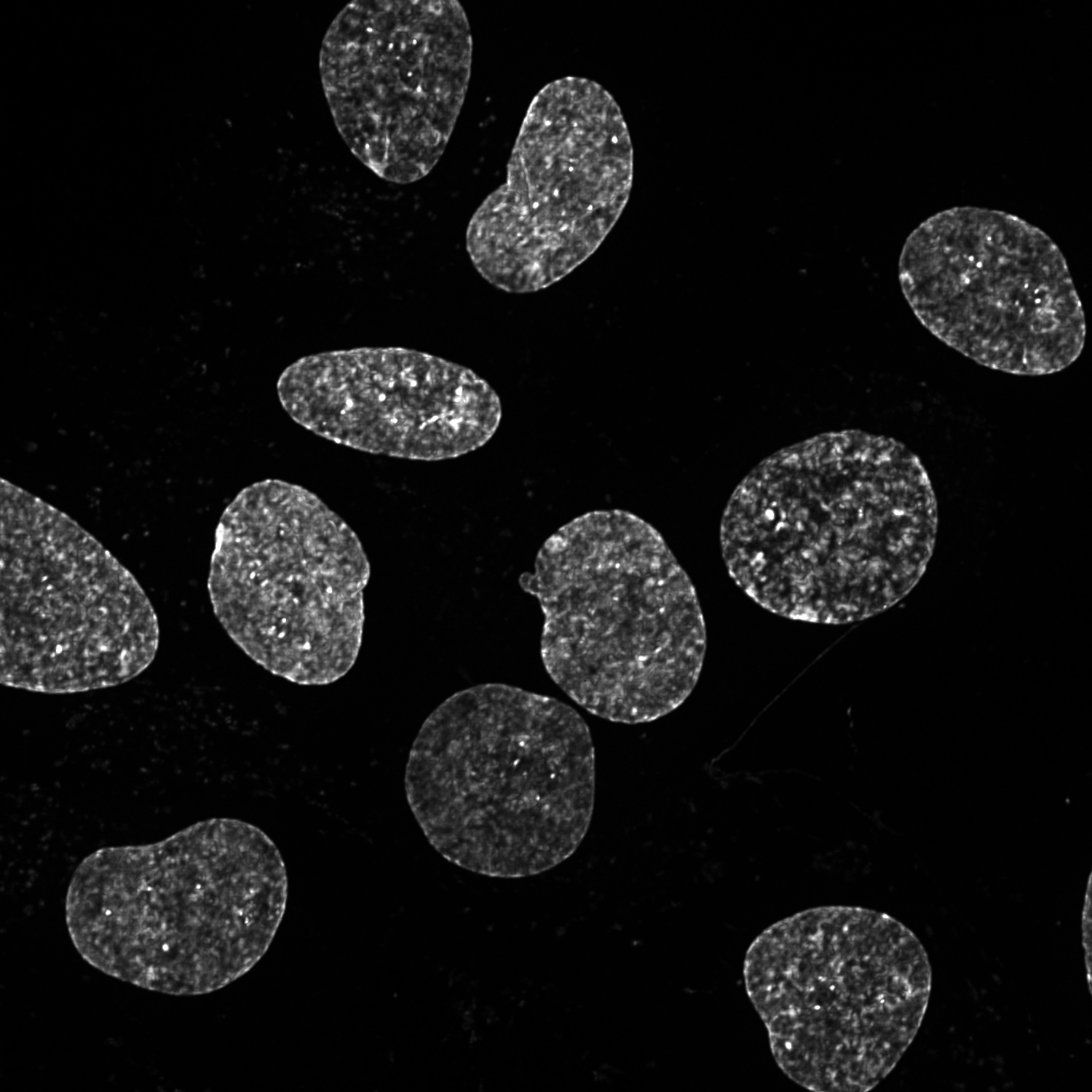

Supplement: Supplementary file 14 — Source data Figure EV2 [file 44318_2024_337_MOESM14_ESM.zip › 08_Figure_EV2/A/FLUO-CLTC/_FULL-RANGE-FLUO-CLTC.tif]

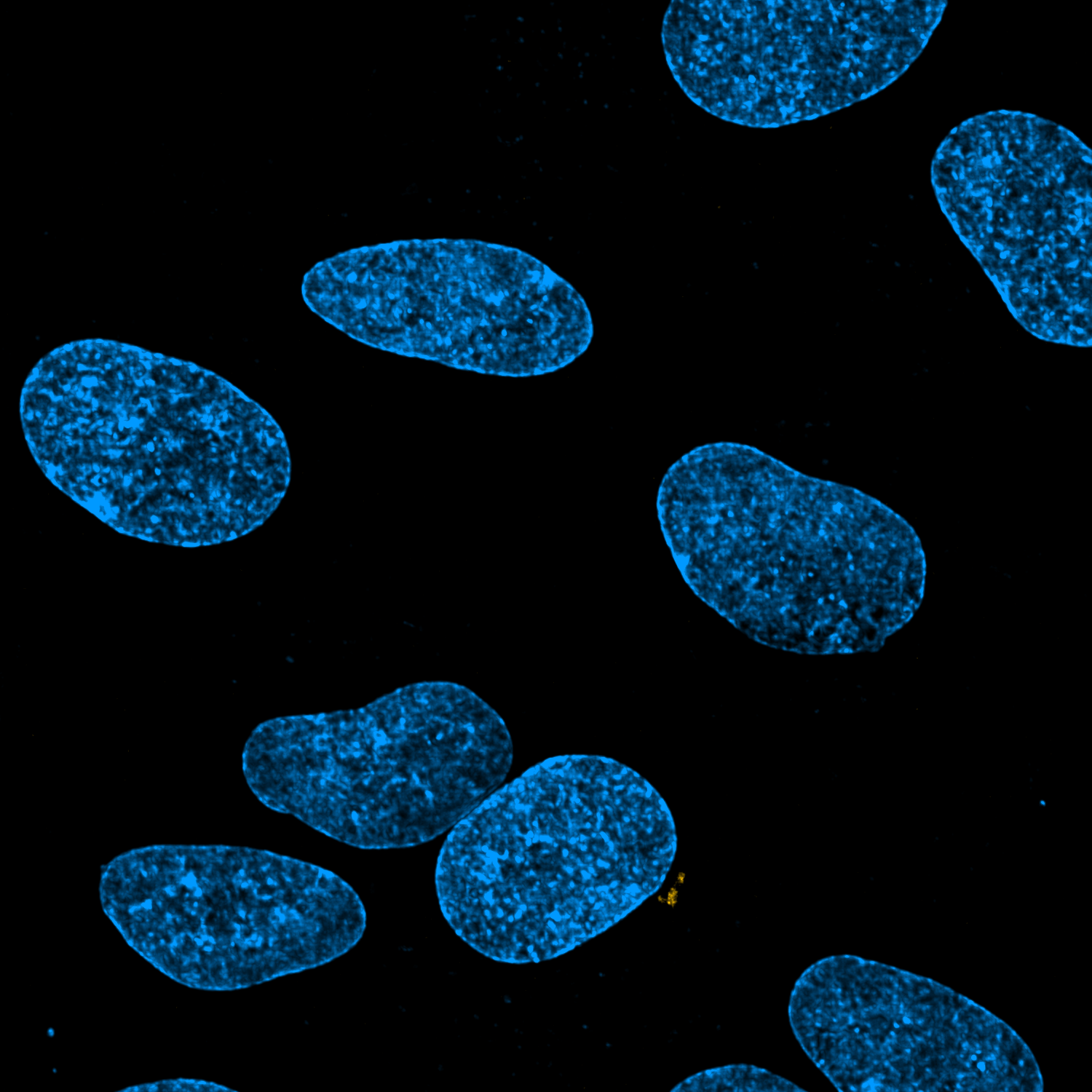

Supplement: Supplementary file 14 — Source data Figure EV2 [file 44318_2024_337_MOESM14_ESM.zip › 08_Figure_EV2/A/FLUO-CTRL/FLUO-CTRL_Merge.tif]

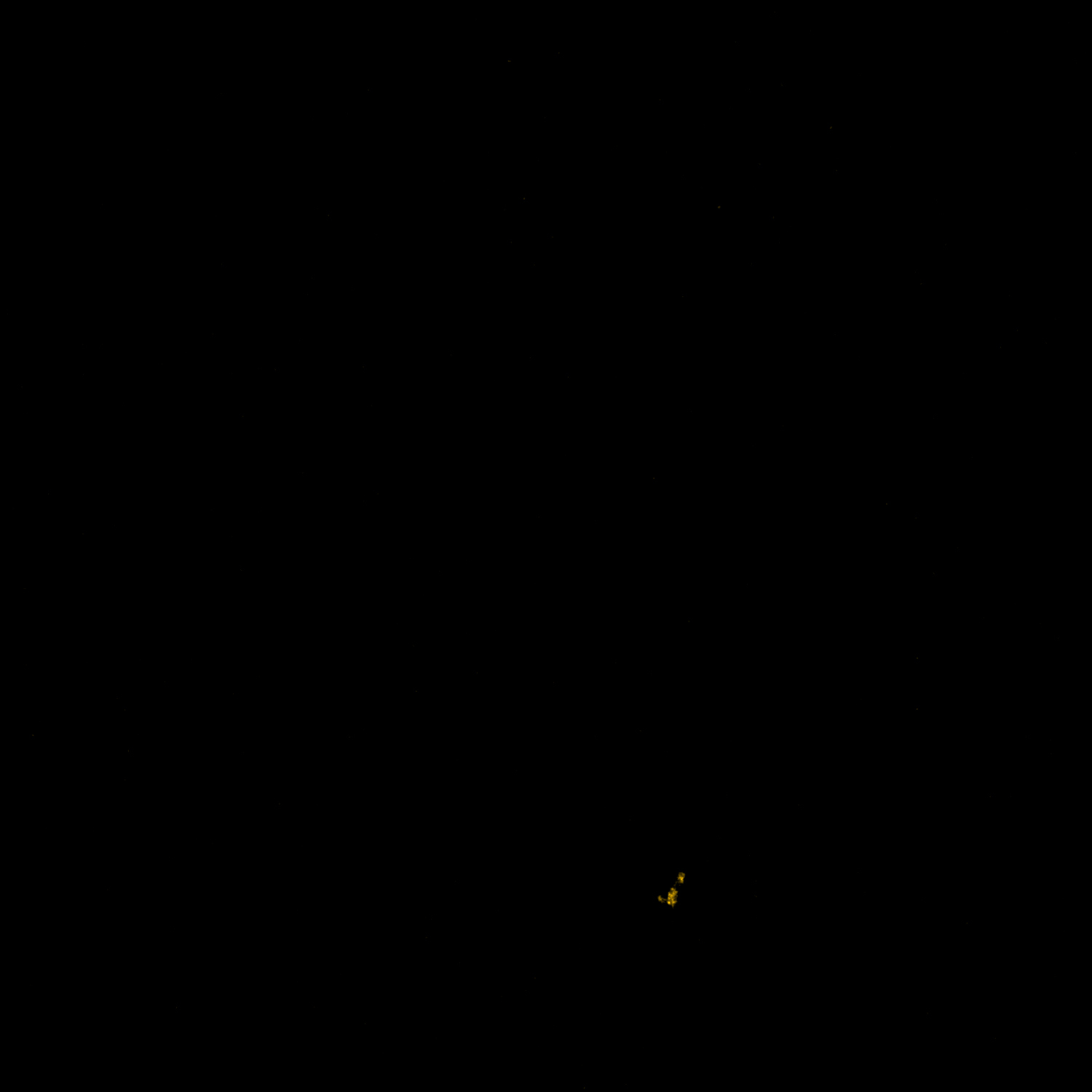

Supplement: Supplementary file 14 — Source data Figure EV2 [file 44318_2024_337_MOESM14_ESM.zip › 08_Figure_EV2/A/FLUO-CTRL/FLUO-CTRL_mStayGold.tif]

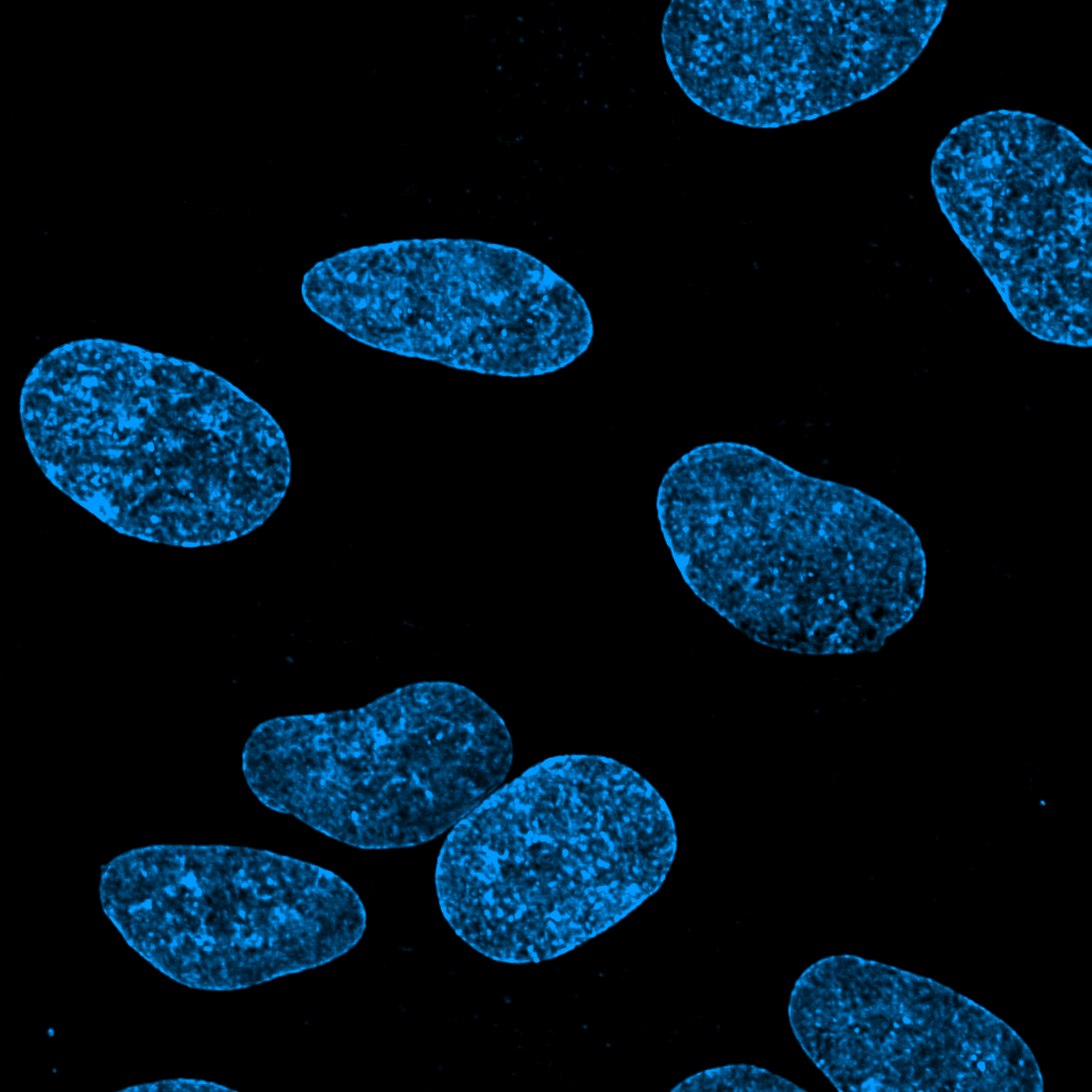

Supplement: Supplementary file 14 — Source data Figure EV2 [file 44318_2024_337_MOESM14_ESM.zip › 08_Figure_EV2/A/FLUO-CTRL/FLUO-CTRL_SiR-DNA.tif]

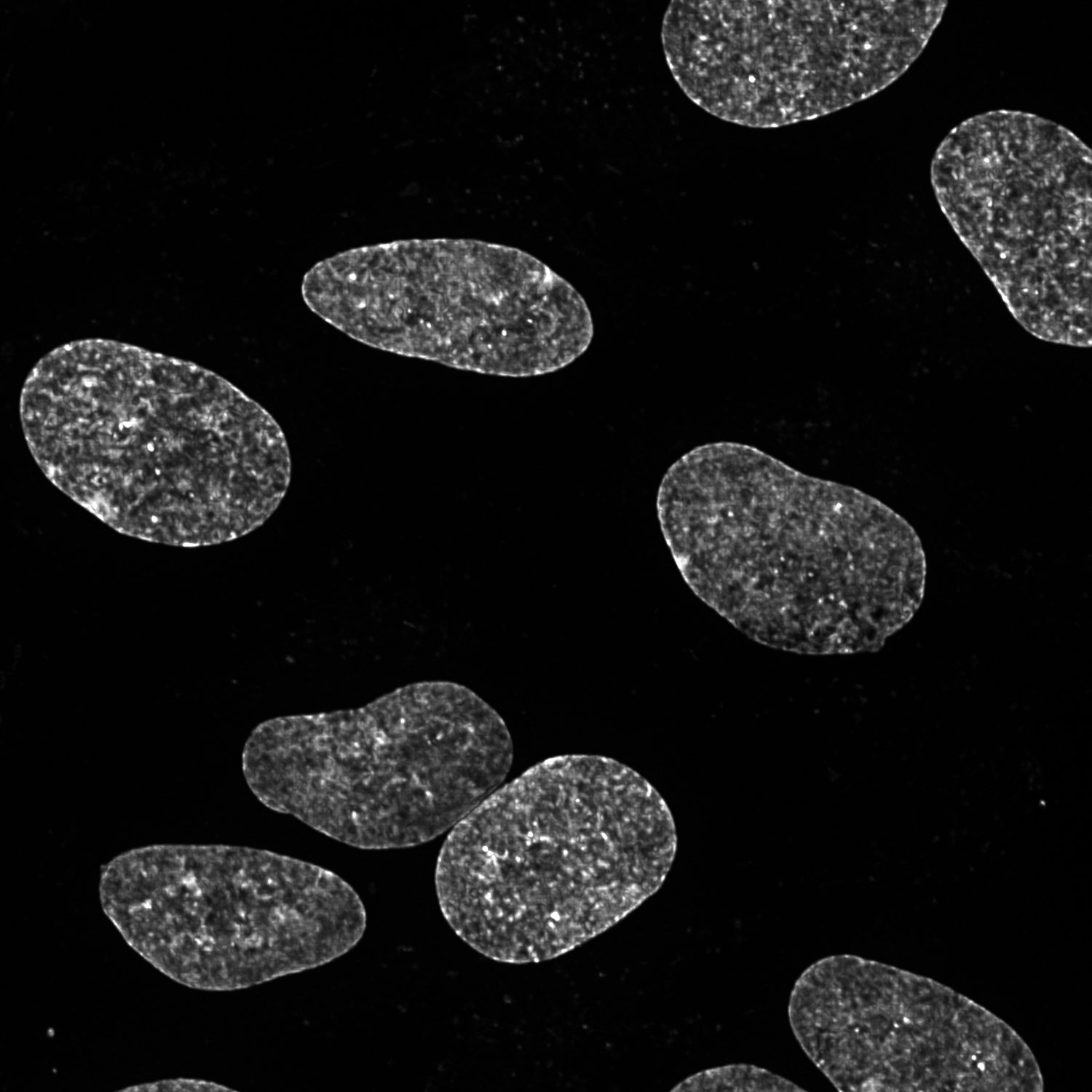

Supplement: Supplementary file 14 — Source data Figure EV2 [file 44318_2024_337_MOESM14_ESM.zip › 08_Figure_EV2/A/FLUO-CTRL/_FULL-RANGE-FLUO-CTRL.tif]

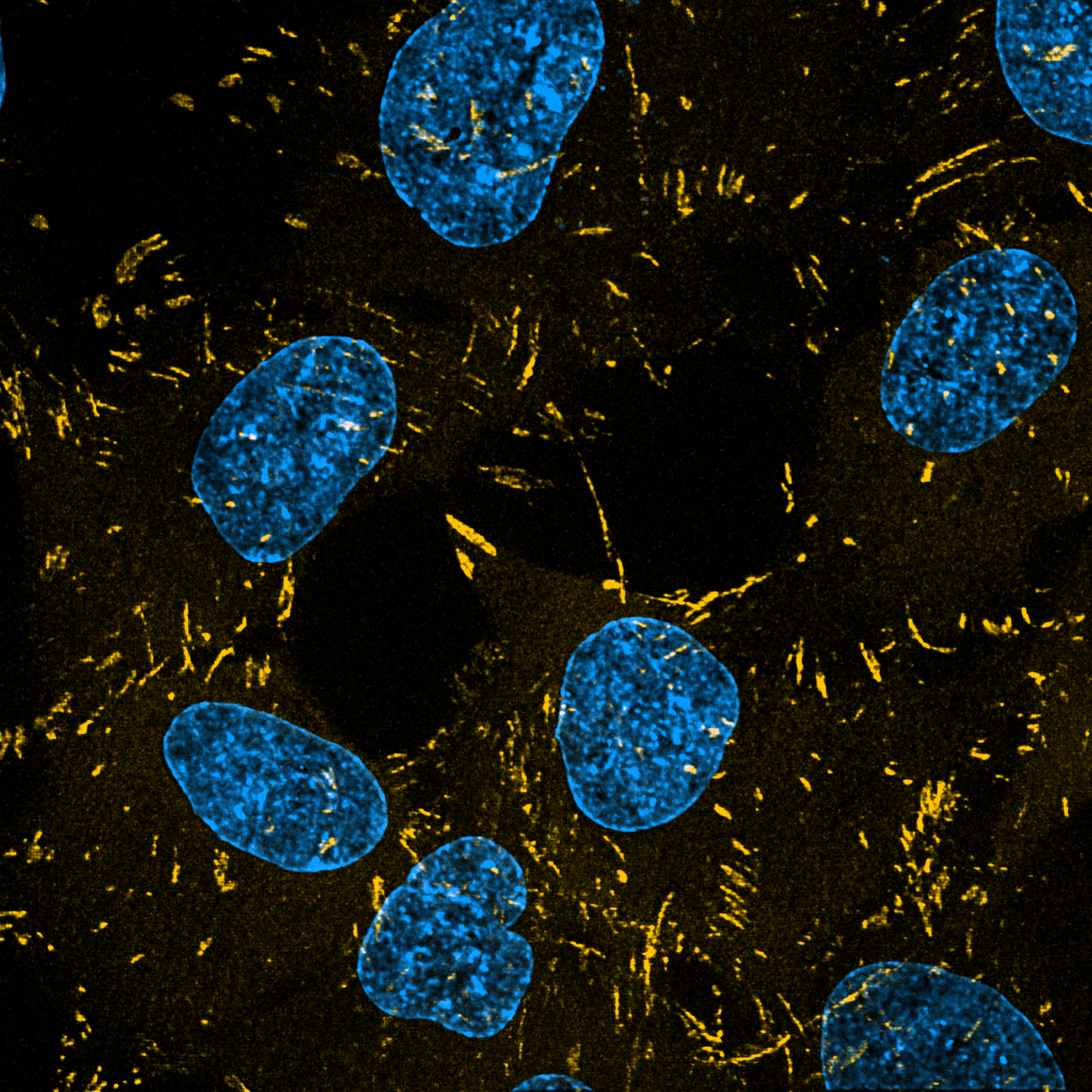

Supplement: Supplementary file 14 — Source data Figure EV2 [file 44318_2024_337_MOESM14_ESM.zip › 08_Figure_EV2/A/FLUO-PXN/FLUO-PXN_Merge.tif]

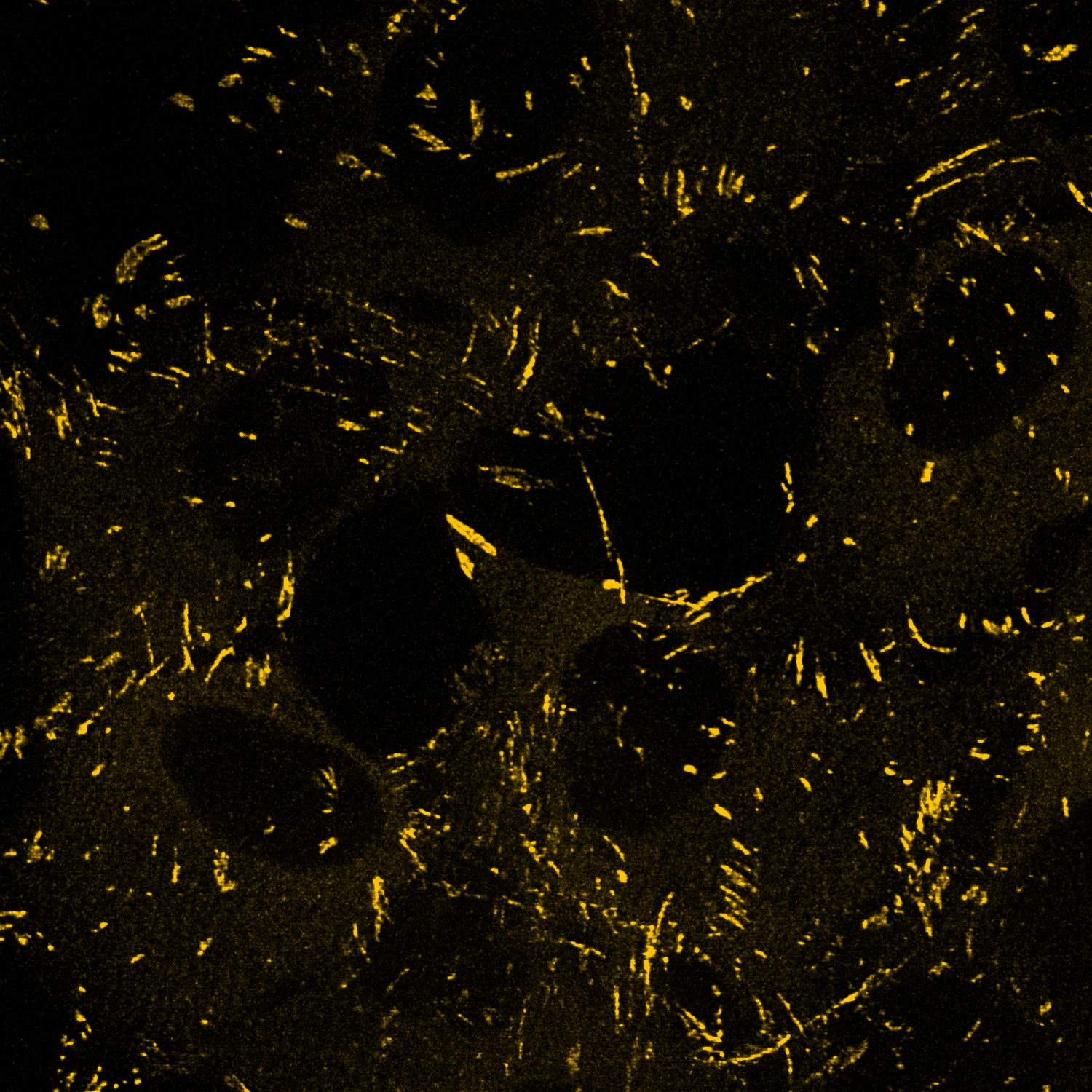

Supplement: Supplementary file 14 — Source data Figure EV2 [file 44318_2024_337_MOESM14_ESM.zip › 08_Figure_EV2/A/FLUO-PXN/FLUO-PXN_mStayGold.tif]

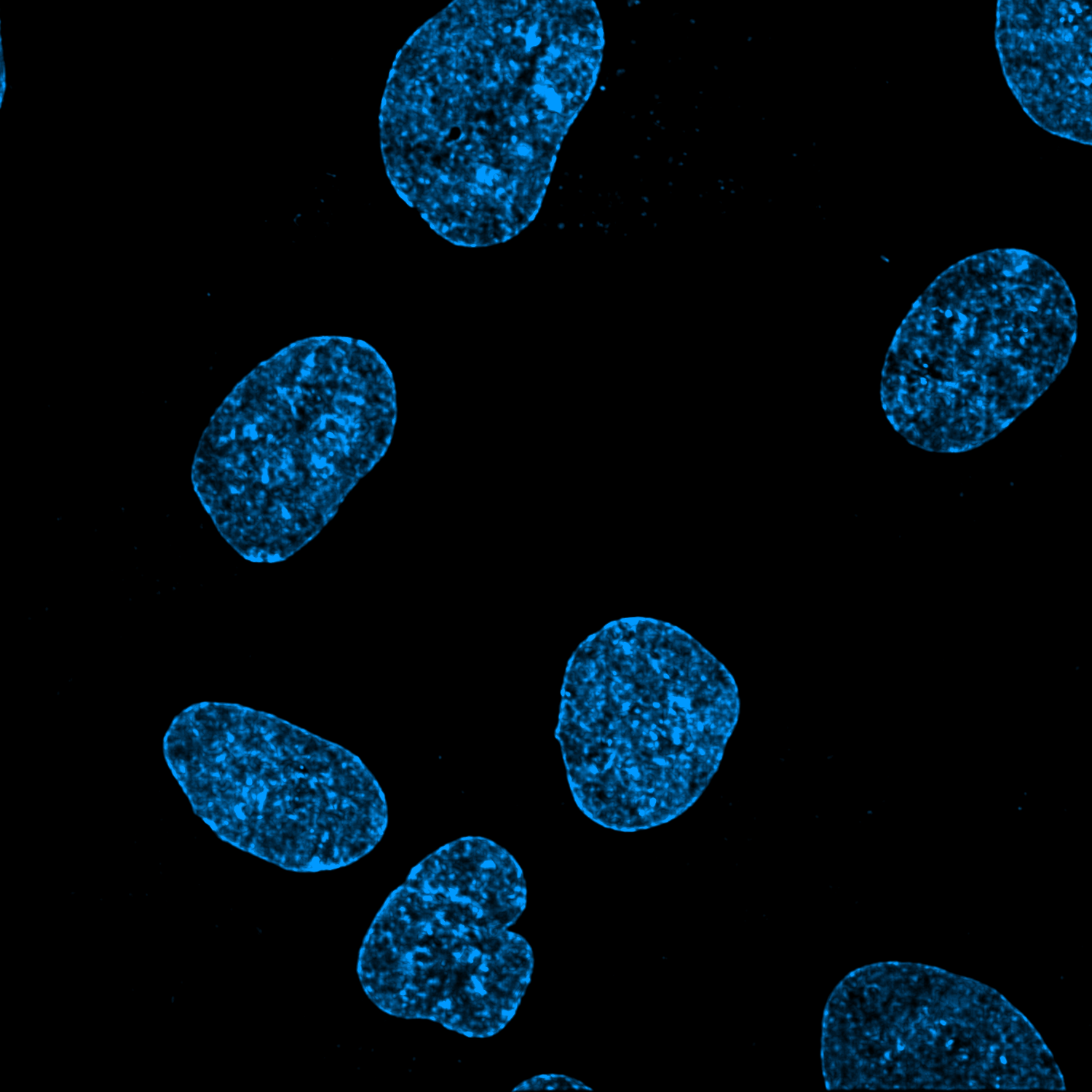

Supplement: Supplementary file 14 — Source data Figure EV2 [file 44318_2024_337_MOESM14_ESM.zip › 08_Figure_EV2/A/FLUO-PXN/FLUO-PXN_SiR-DNA.tif]

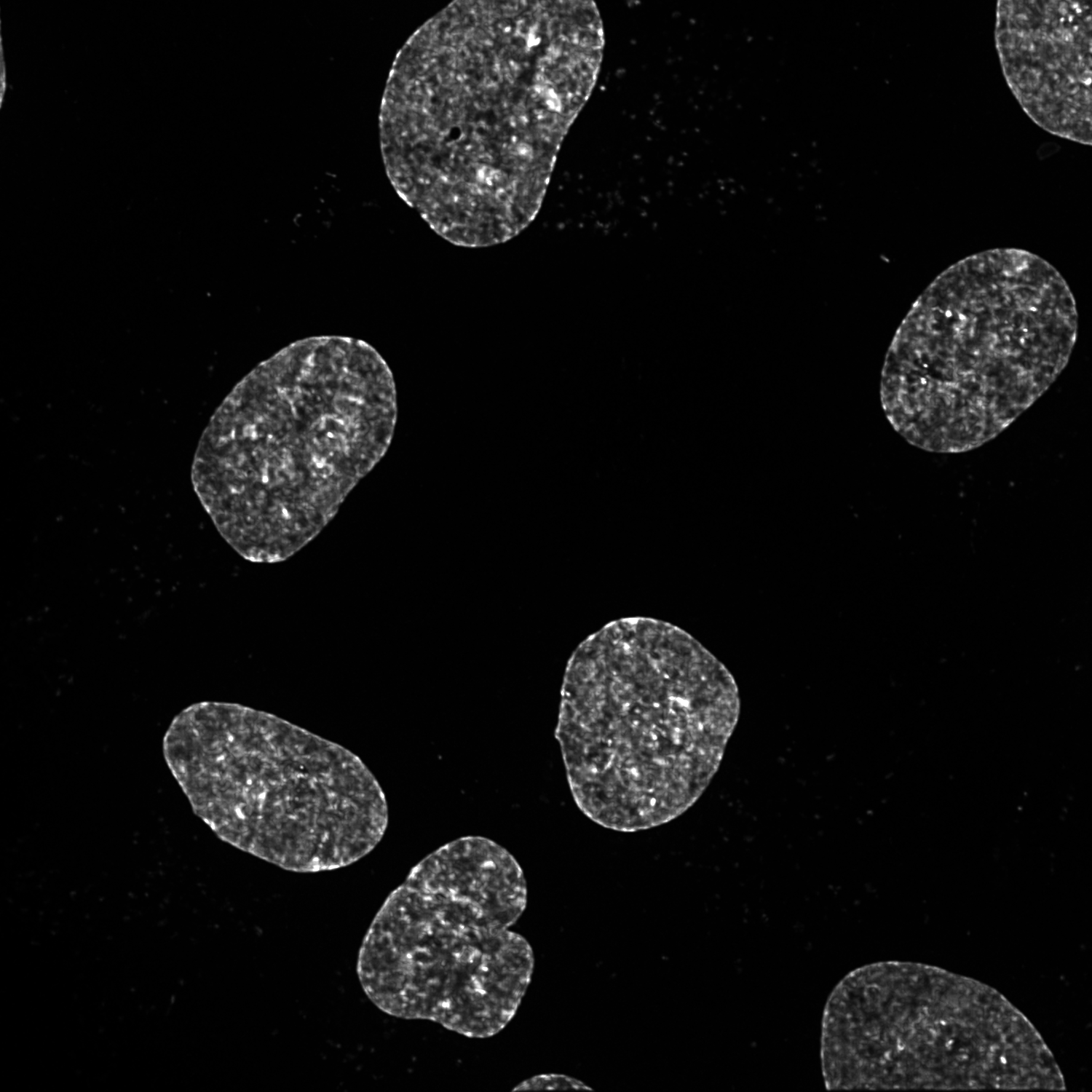

Supplement: Supplementary file 14 — Source data Figure EV2 [file 44318_2024_337_MOESM14_ESM.zip › 08_Figure_EV2/A/FLUO-PXN/_FULL-RANGE-FLUO-PXN.tif]

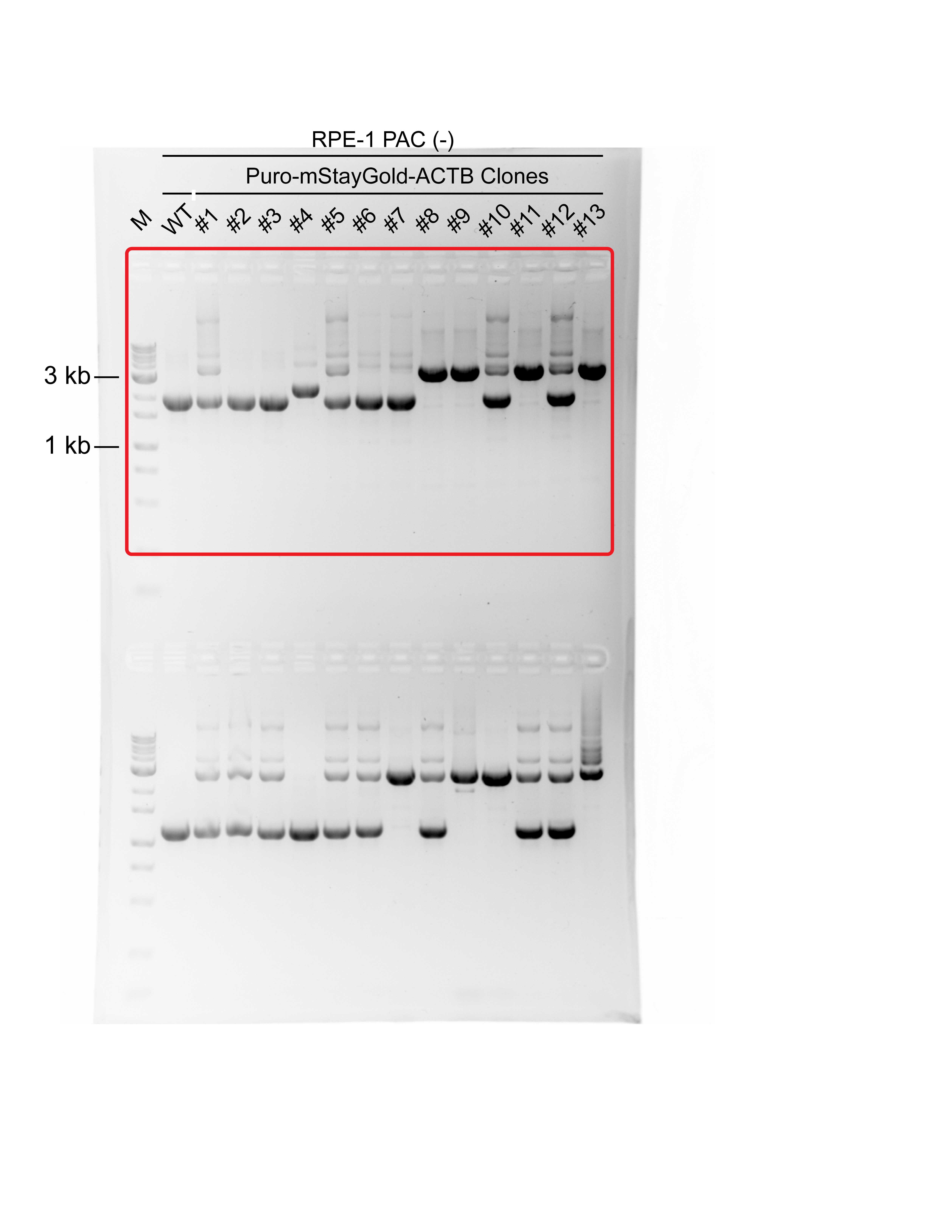

Supplement: Supplementary file 14 — Source data Figure EV2 [file 44318_2024_337_MOESM14_ESM.zip › 08_Figure_EV2/D/ACTB-Clonal-Outcomes.tif]

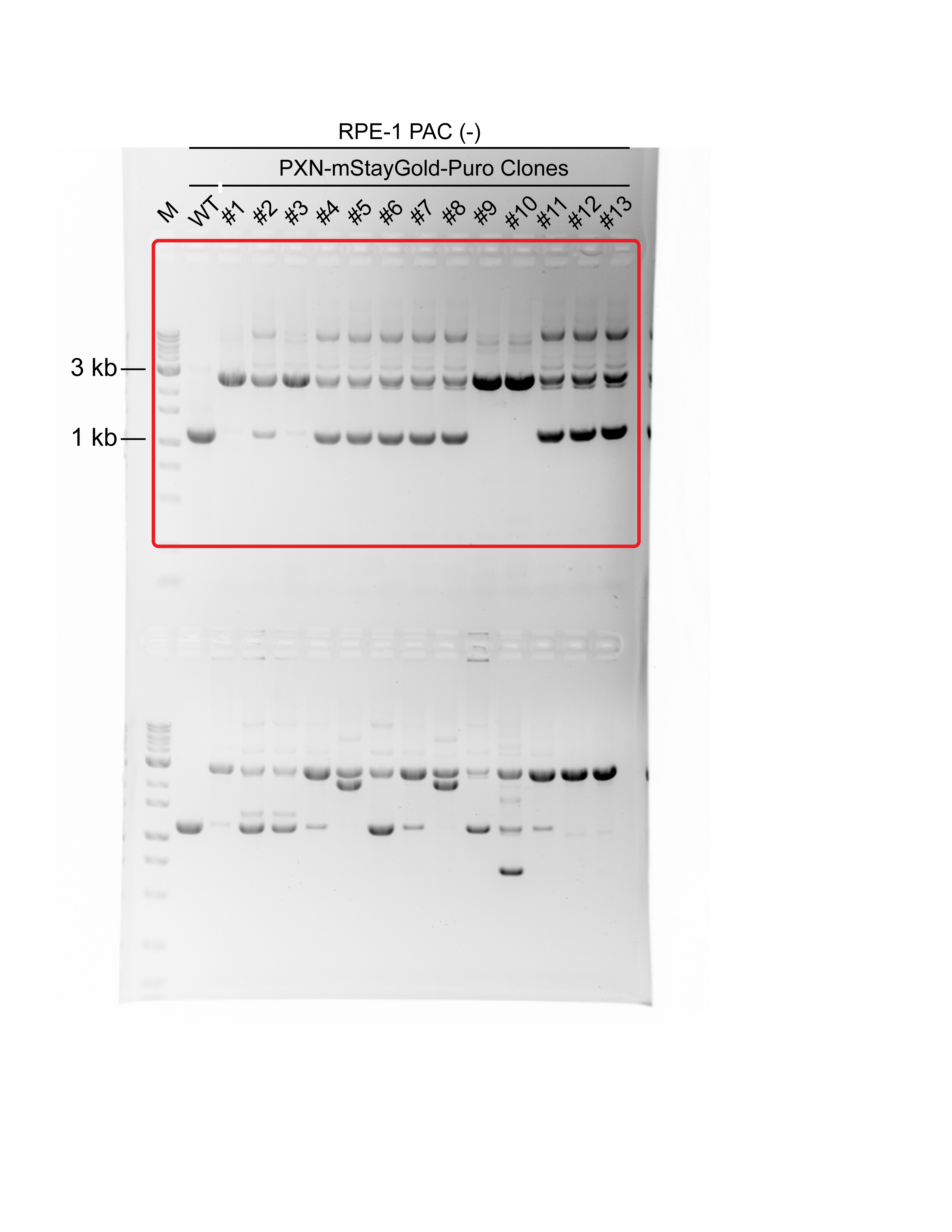

Supplement: Supplementary file 14 — Source data Figure EV2 [file 44318_2024_337_MOESM14_ESM.zip › 08_Figure_EV2/E/PXN-Clonal-Outcomes.tif]

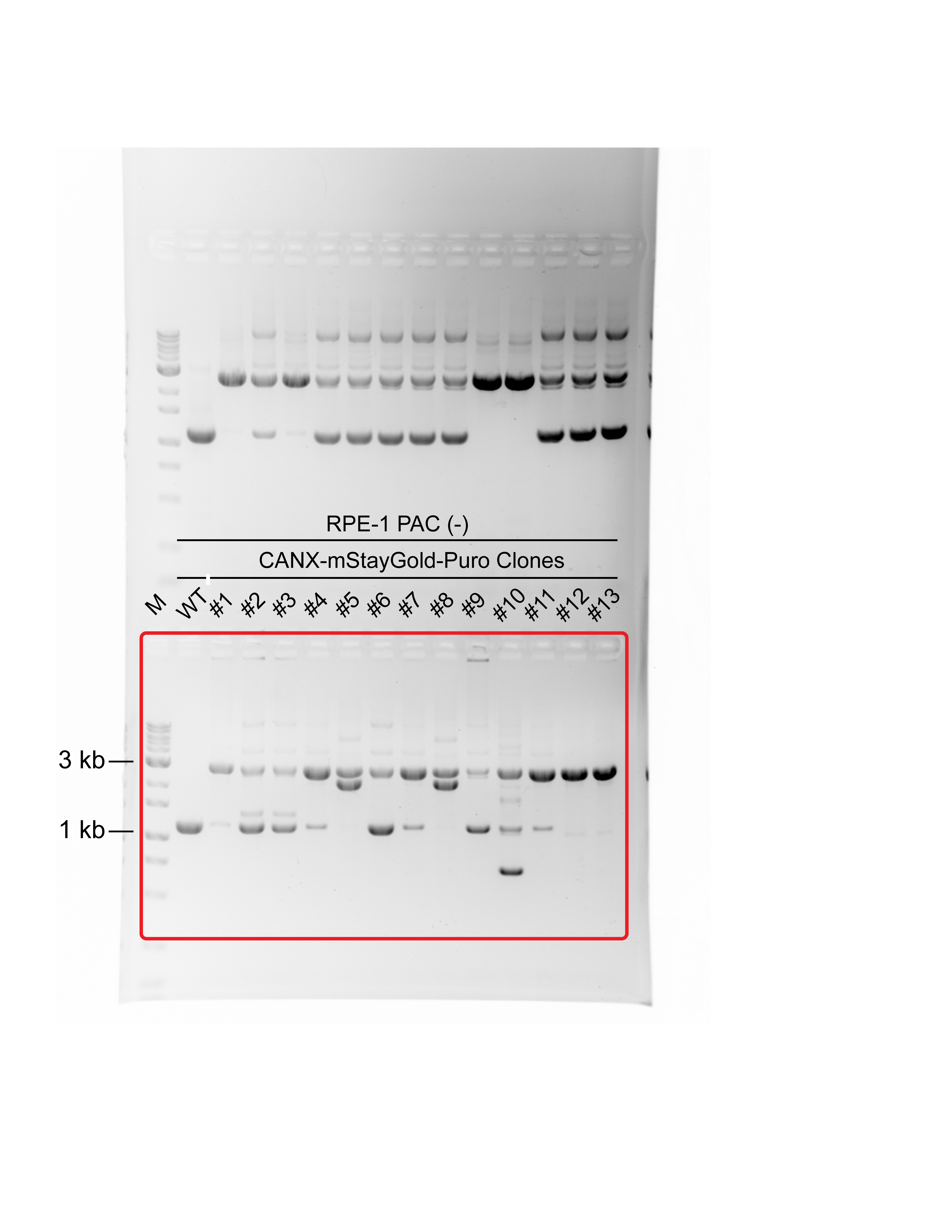

Supplement: Supplementary file 14 — Source data Figure EV2 [file 44318_2024_337_MOESM14_ESM.zip › 08_Figure_EV2/F/CANX-Clonal-Outcomes.tif]

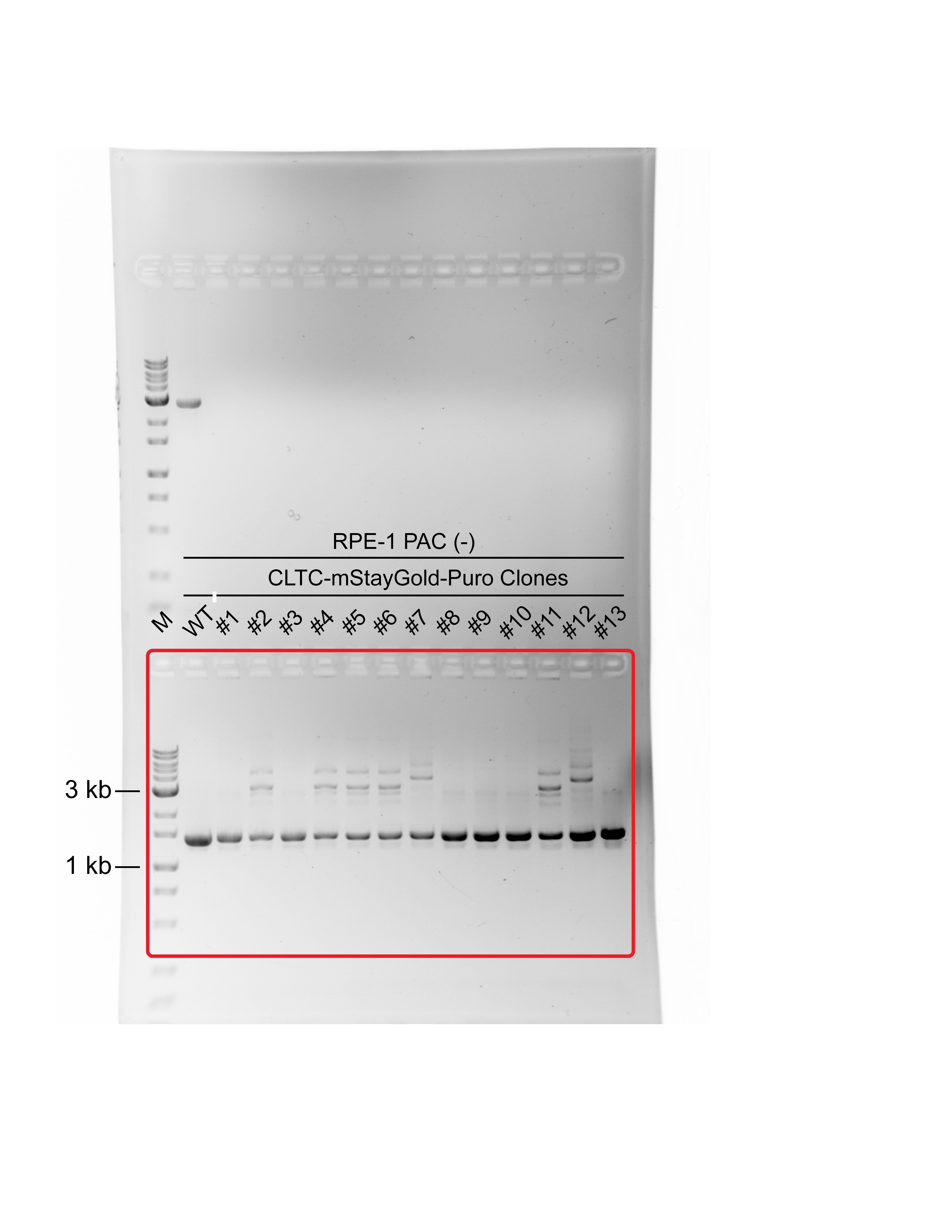

Supplement: Supplementary file 14 — Source data Figure EV2 [file 44318_2024_337_MOESM14_ESM.zip › 08_Figure_EV2/G/CLTC-Clonal-Outcomes.tif]

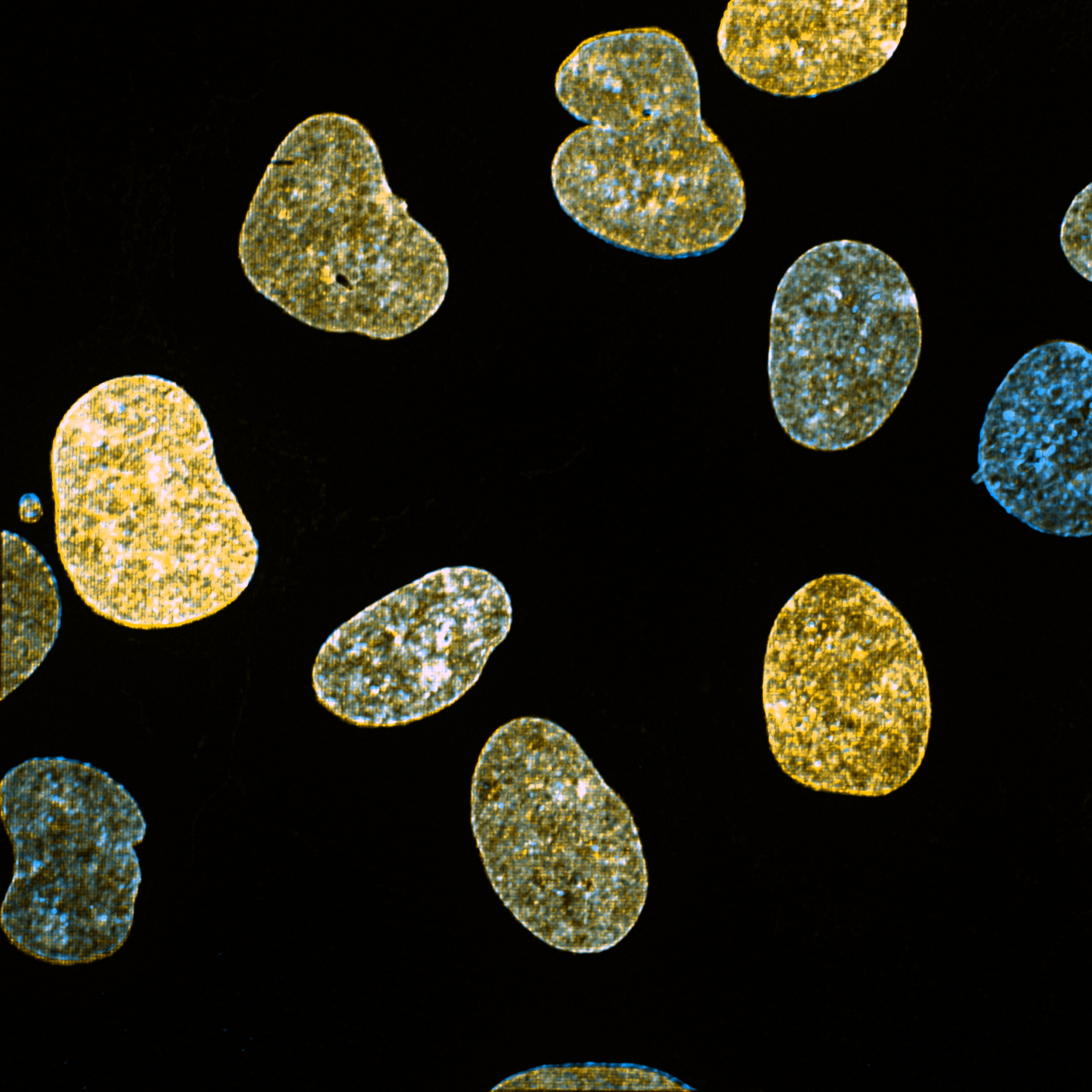

Supplement: Supplementary file 15 — Source data Figure EV3 [file 44318_2024_337_MOESM15_ESM.zip › 09_Figure_EV3/A/HDR-H2BH/HDR-MMEJ-H2BH_Merge.tif]

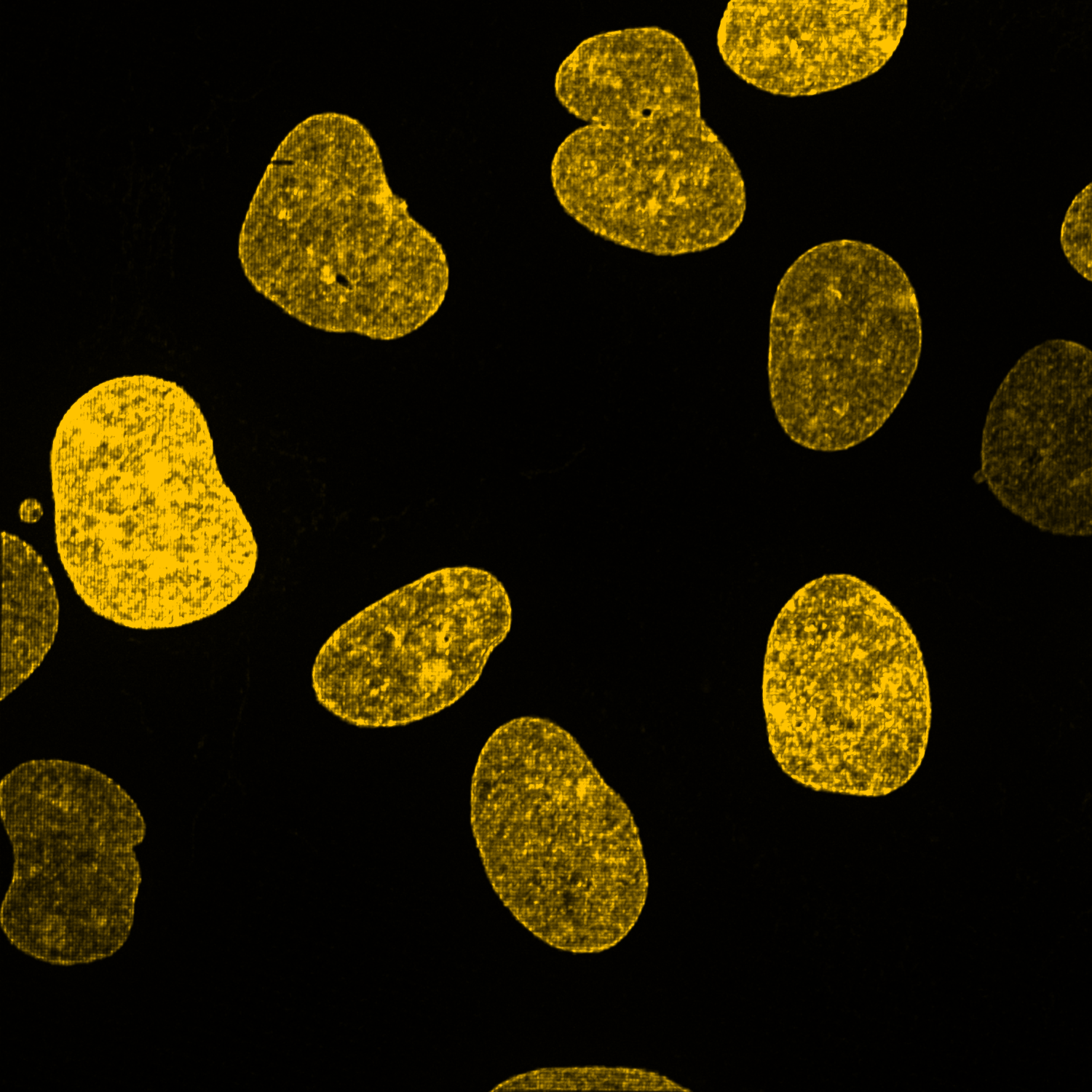

Supplement: Supplementary file 15 — Source data Figure EV3 [file 44318_2024_337_MOESM15_ESM.zip › 09_Figure_EV3/A/HDR-H2BH/HDR-MMEJ-H2BH_mStayGold.tif]

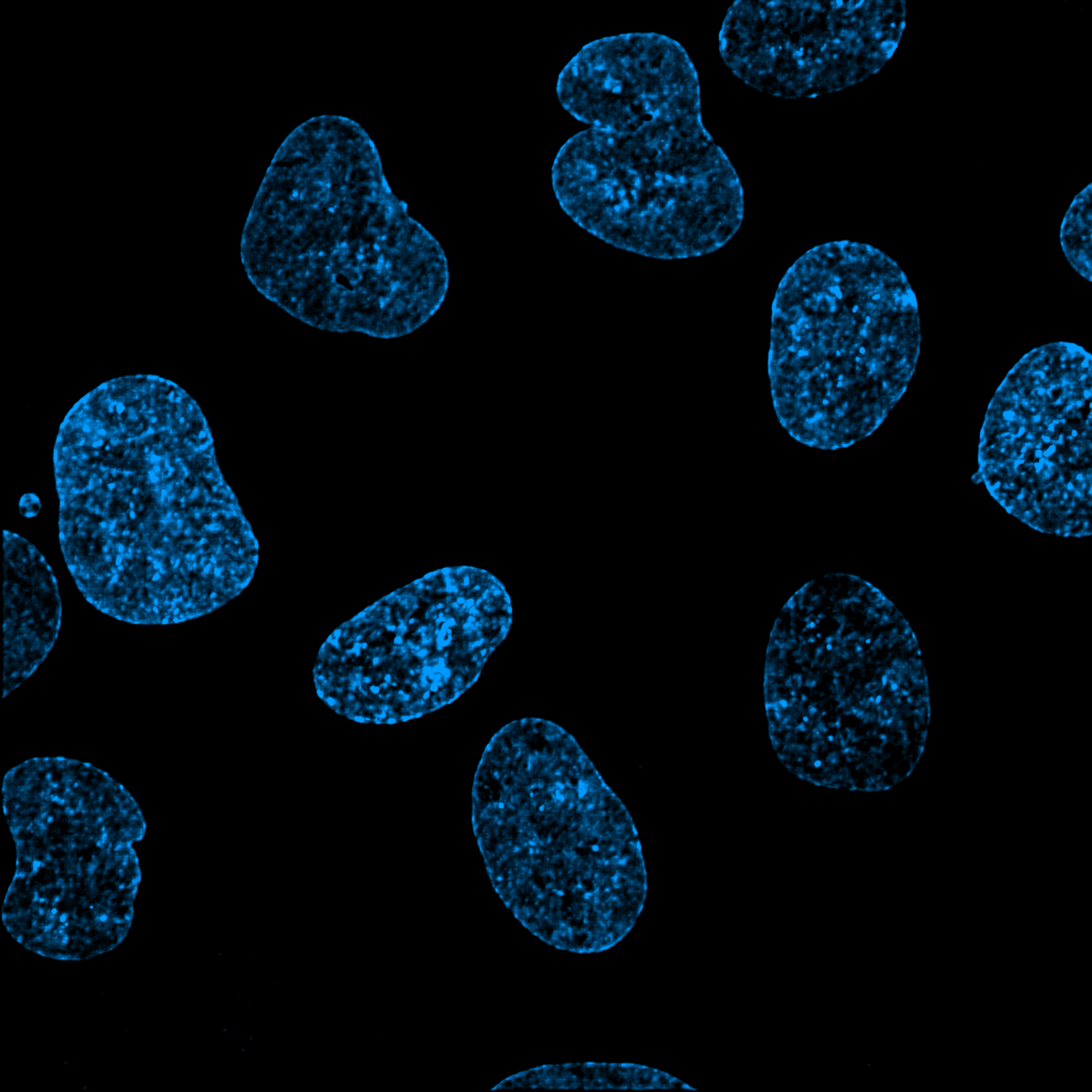

Supplement: Supplementary file 15 — Source data Figure EV3 [file 44318_2024_337_MOESM15_ESM.zip › 09_Figure_EV3/A/HDR-H2BH/HDR-MMEJ-H2BH_SiR-DNA.tif]

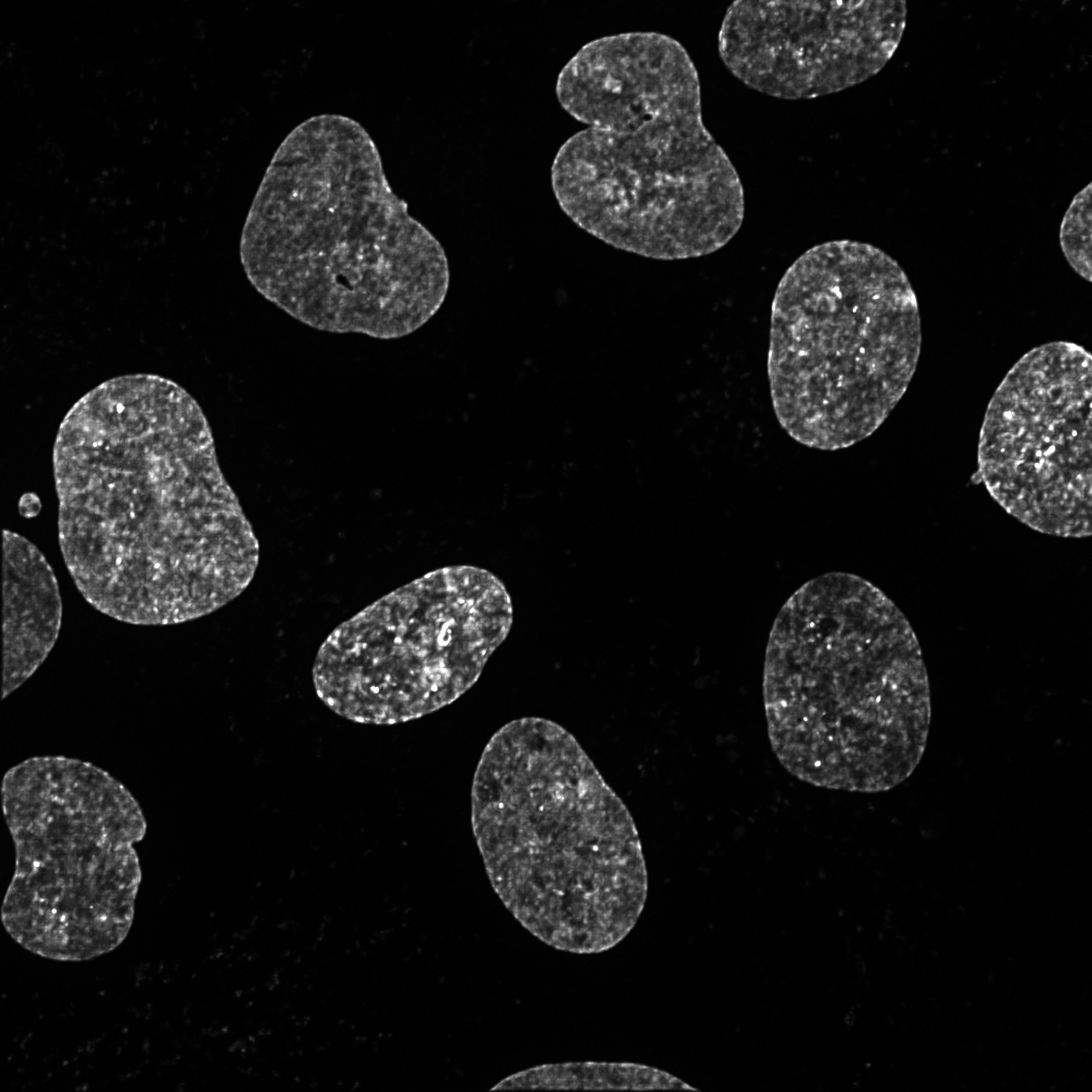

Supplement: Supplementary file 15 — Source data Figure EV3 [file 44318_2024_337_MOESM15_ESM.zip › 09_Figure_EV3/A/HDR-H2BH/_FULL-RANGE-HDR-MMEJ-H2BH.tif]

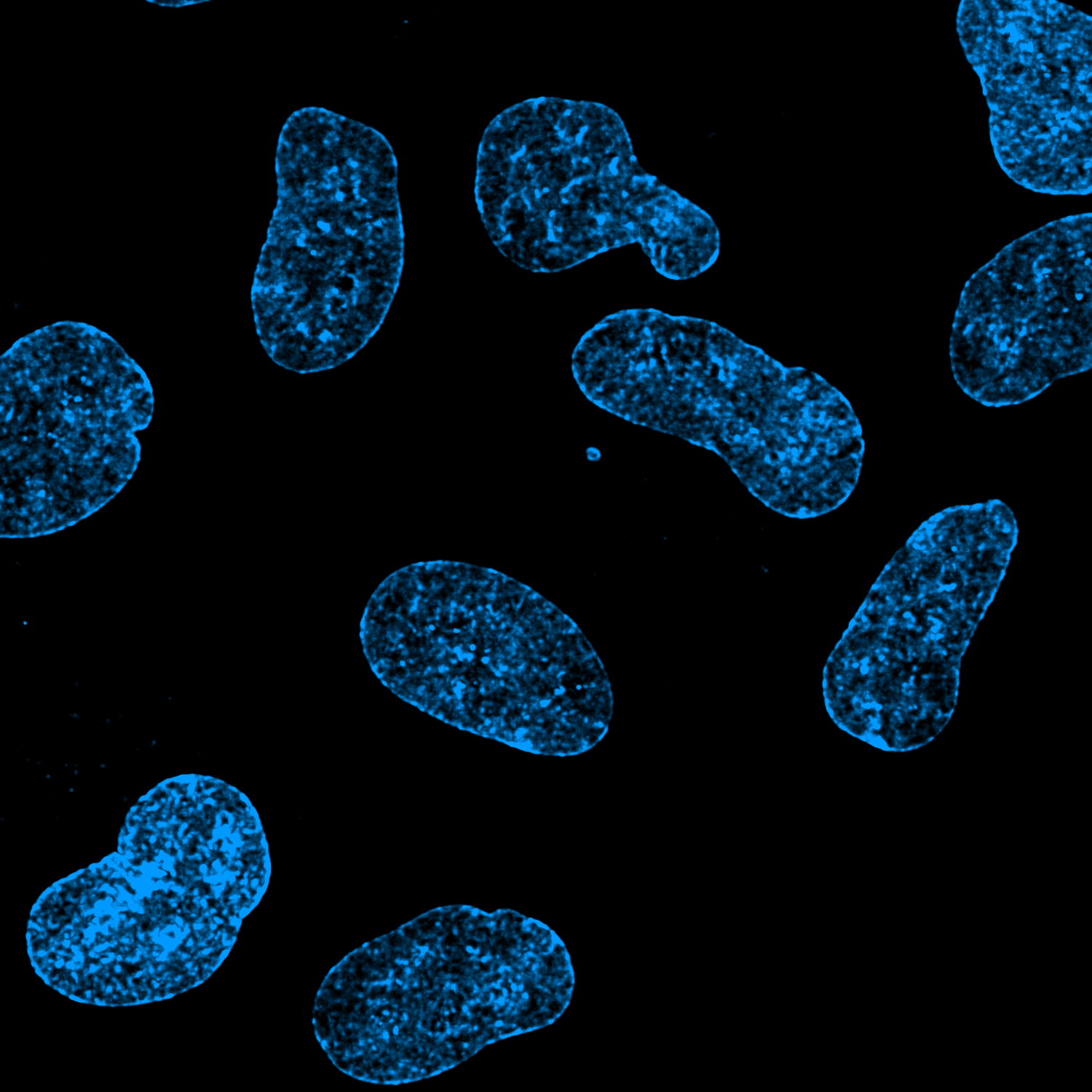

Supplement: Supplementary file 15 — Source data Figure EV3 [file 44318_2024_337_MOESM15_ESM.zip › 09_Figure_EV3/A/HDR-MMEJ-CTRL/HDR-MMEJ-CTRL_Merge.tif]

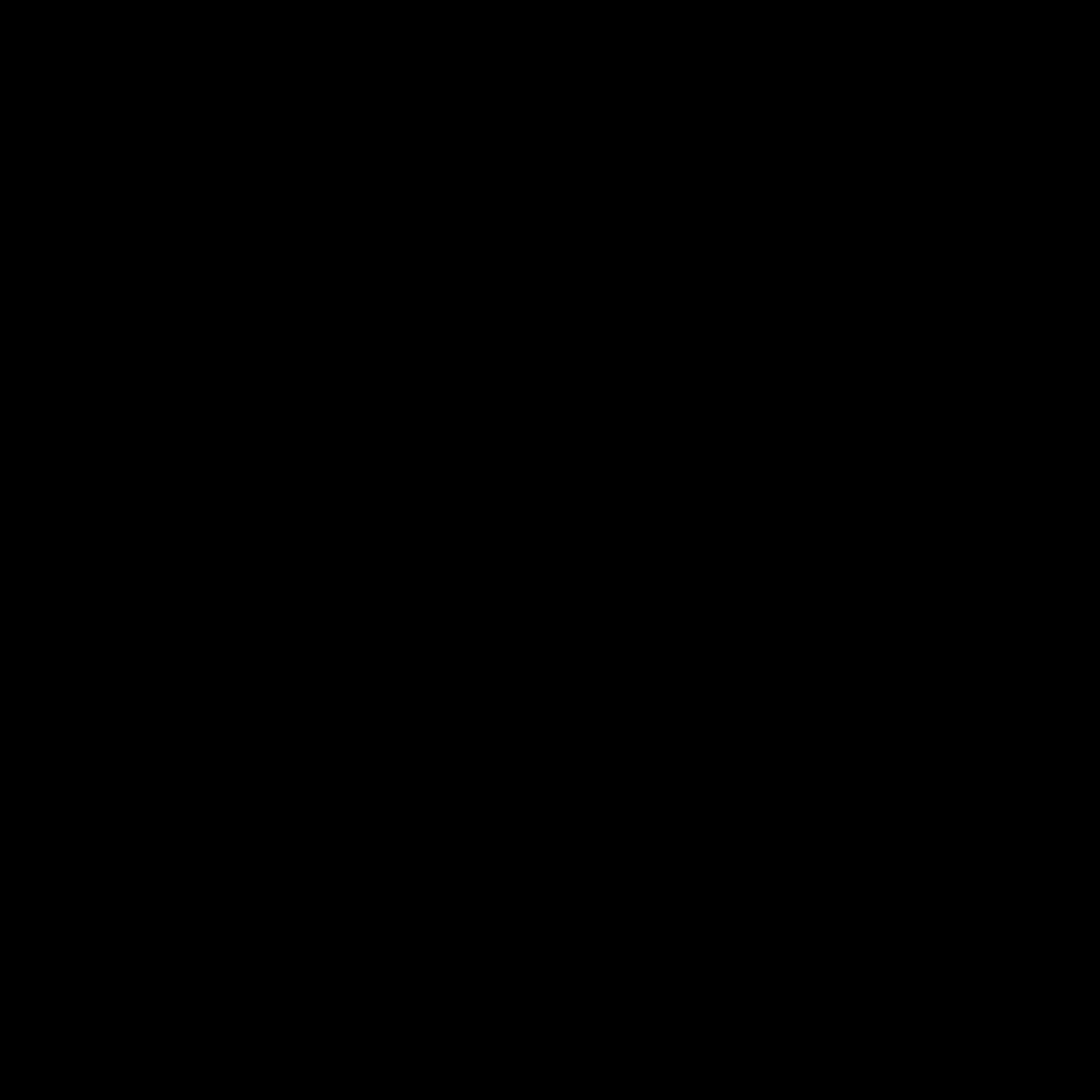

Supplement: Supplementary file 15 — Source data Figure EV3 [file 44318_2024_337_MOESM15_ESM.zip › 09_Figure_EV3/A/HDR-MMEJ-CTRL/HDR-MMEJ-CTRL_mStayGold.tif]

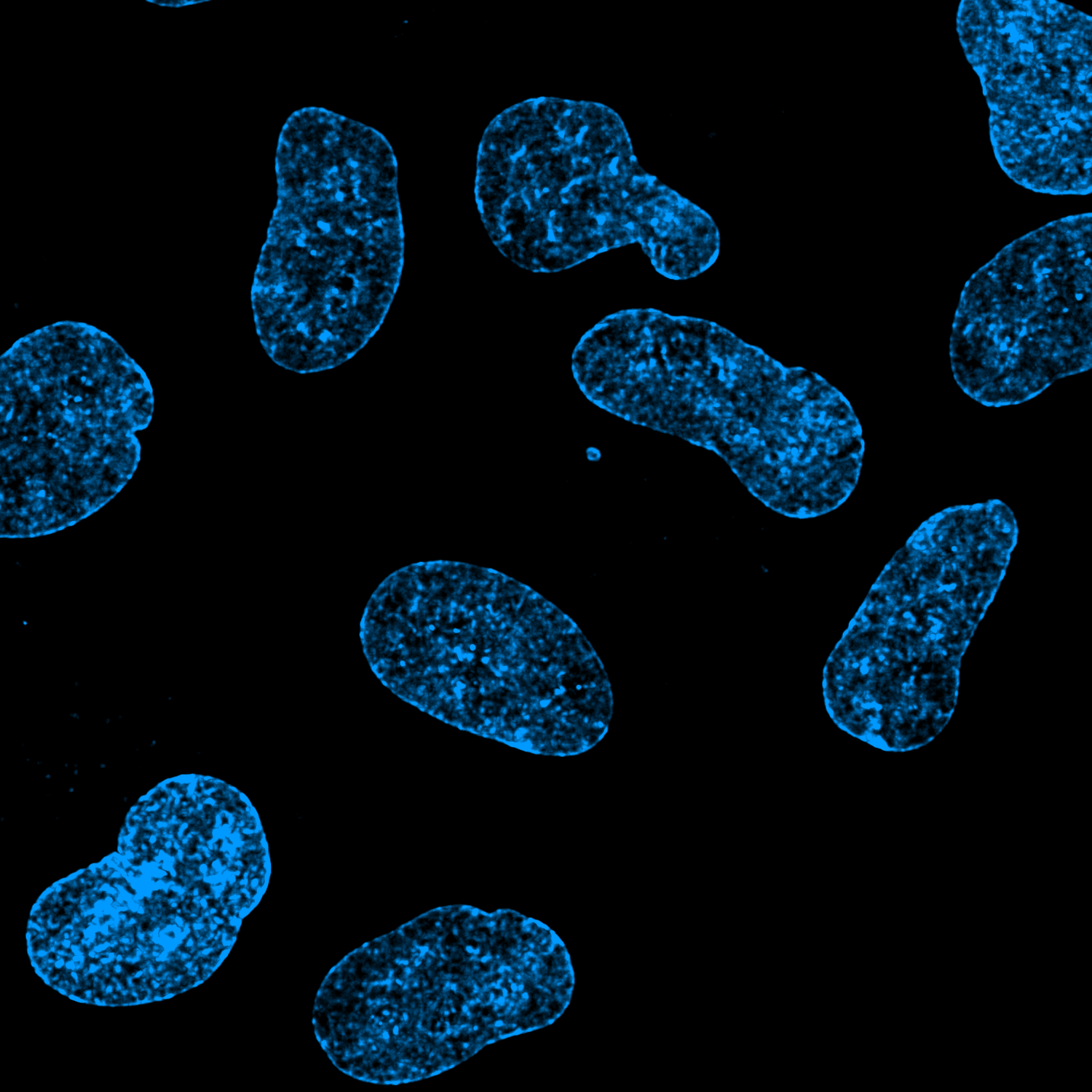

Supplement: Supplementary file 15 — Source data Figure EV3 [file 44318_2024_337_MOESM15_ESM.zip › 09_Figure_EV3/A/HDR-MMEJ-CTRL/HDR-MMEJ-CTRL_SiR-DNA.tif]

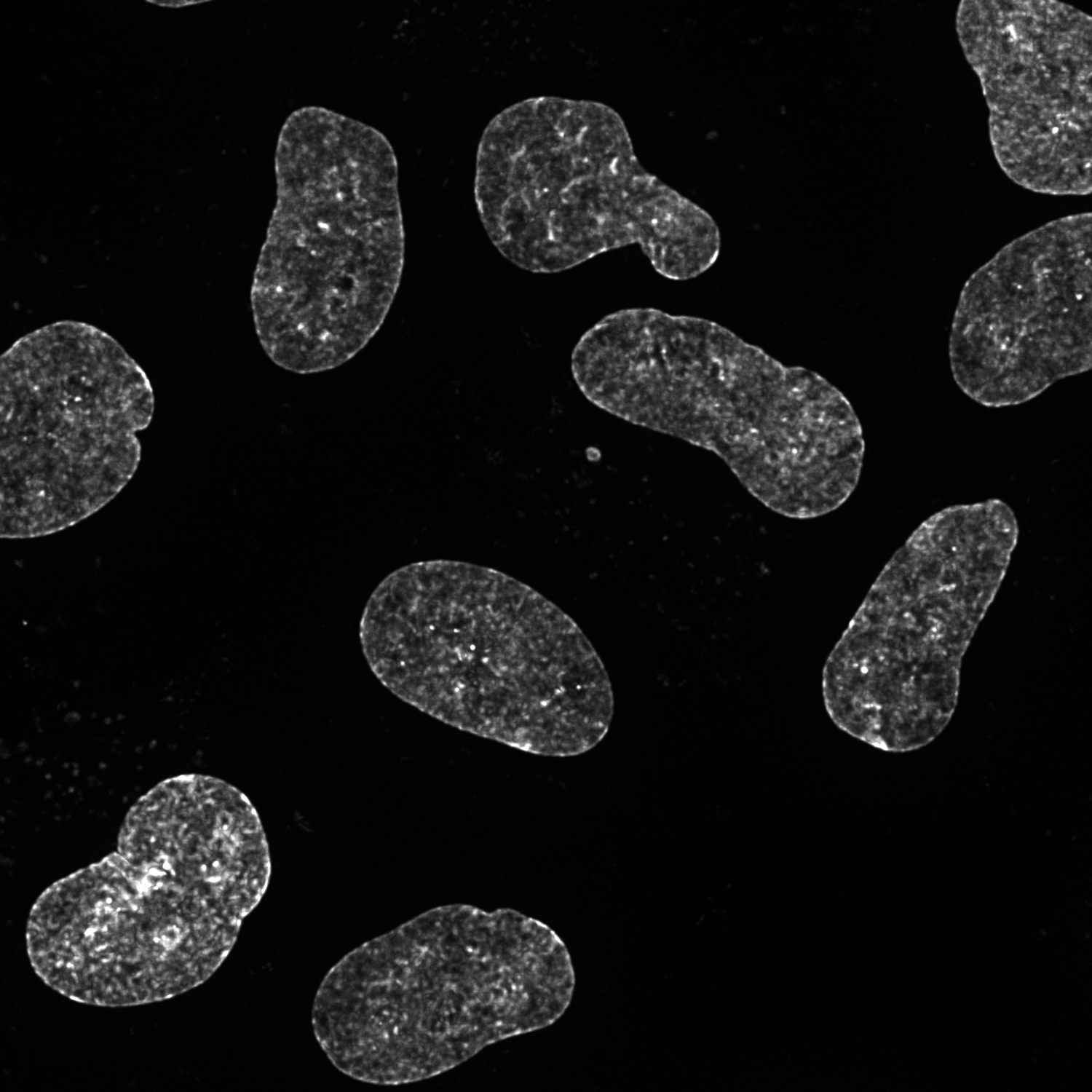

Supplement: Supplementary file 15 — Source data Figure EV3 [file 44318_2024_337_MOESM15_ESM.zip › 09_Figure_EV3/A/HDR-MMEJ-CTRL/_FULL-RANGE-HDR-MMEJ-CTRL.tif]

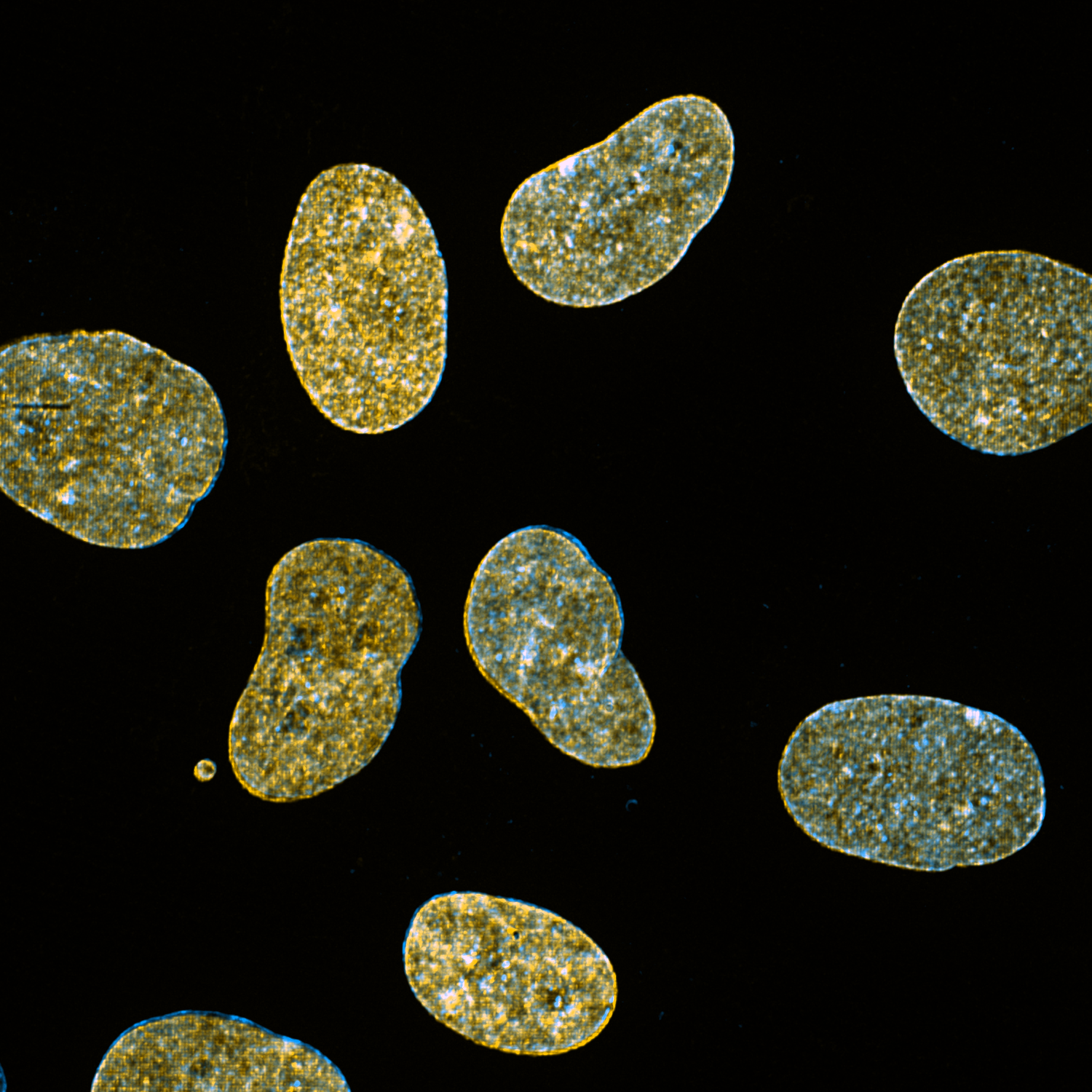

Supplement: Supplementary file 15 — Source data Figure EV3 [file 44318_2024_337_MOESM15_ESM.zip › 09_Figure_EV3/A/HDR-MMEJ-H2BM/HDR-MMEJ-H2BM_Merge.tif]

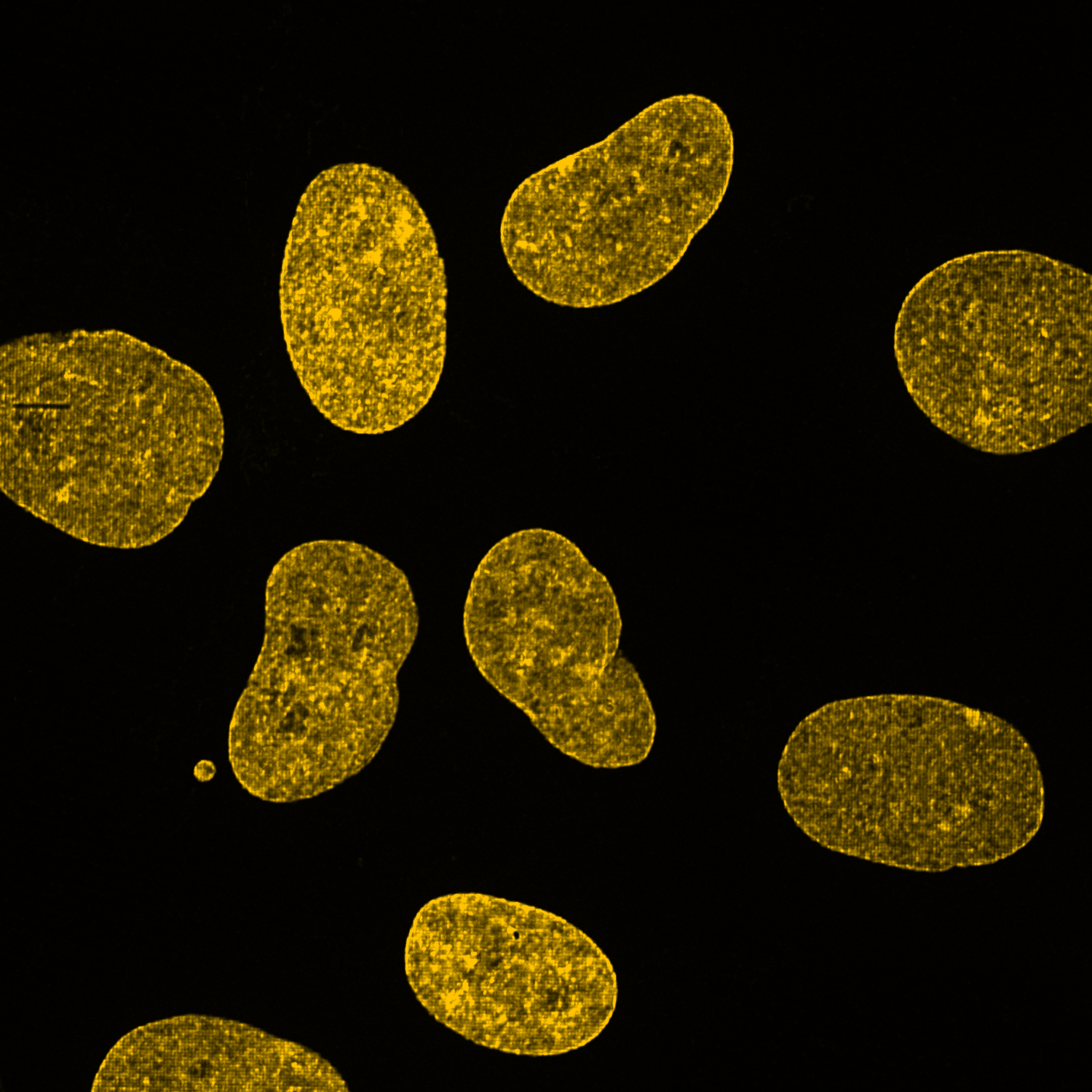

Supplement: Supplementary file 15 — Source data Figure EV3 [file 44318_2024_337_MOESM15_ESM.zip › 09_Figure_EV3/A/HDR-MMEJ-H2BM/HDR-MMEJ-H2BM_mStayGold.tif]

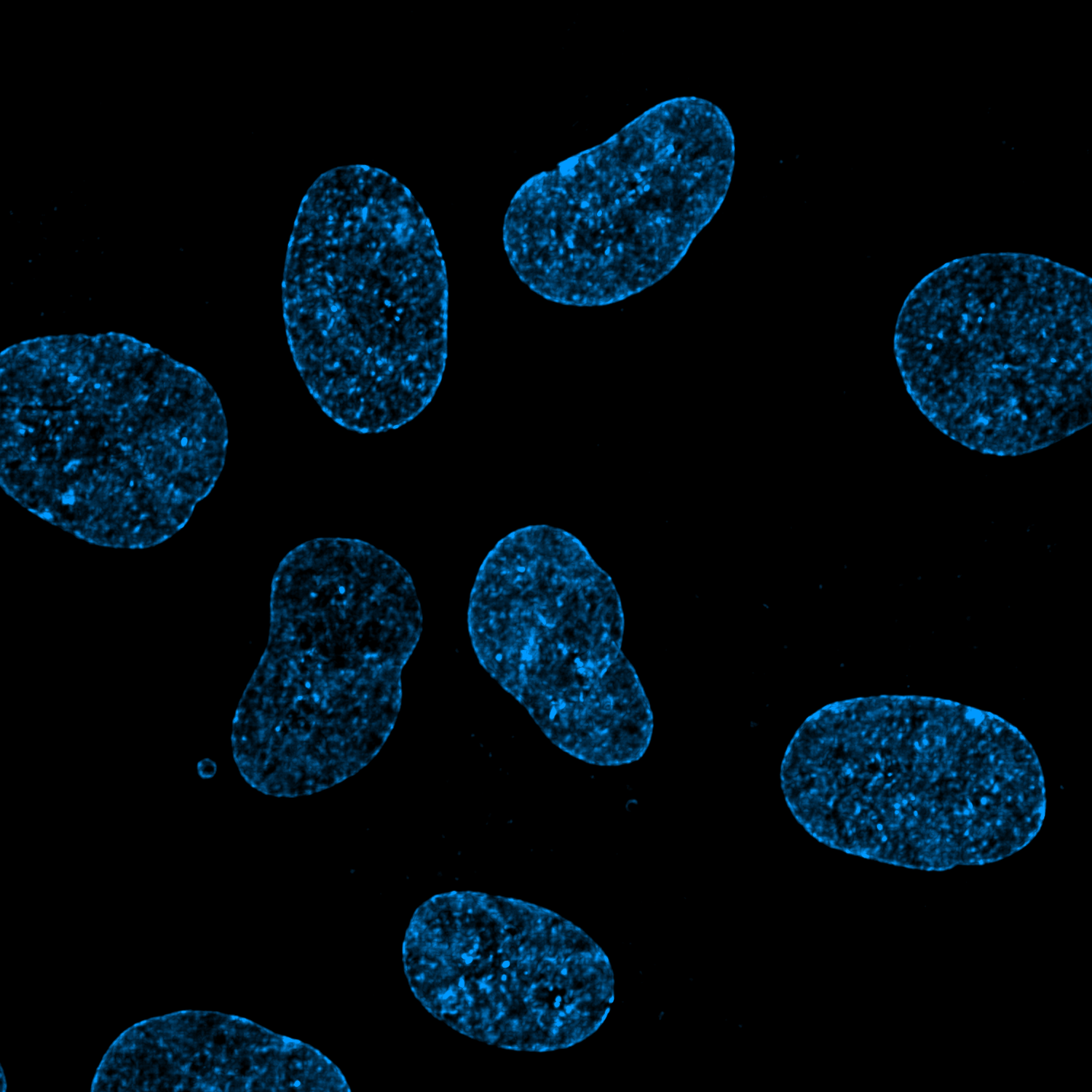

Supplement: Supplementary file 15 — Source data Figure EV3 [file 44318_2024_337_MOESM15_ESM.zip › 09_Figure_EV3/A/HDR-MMEJ-H2BM/HDR-MMEJ-H2BM_SiR-DNA.tif]

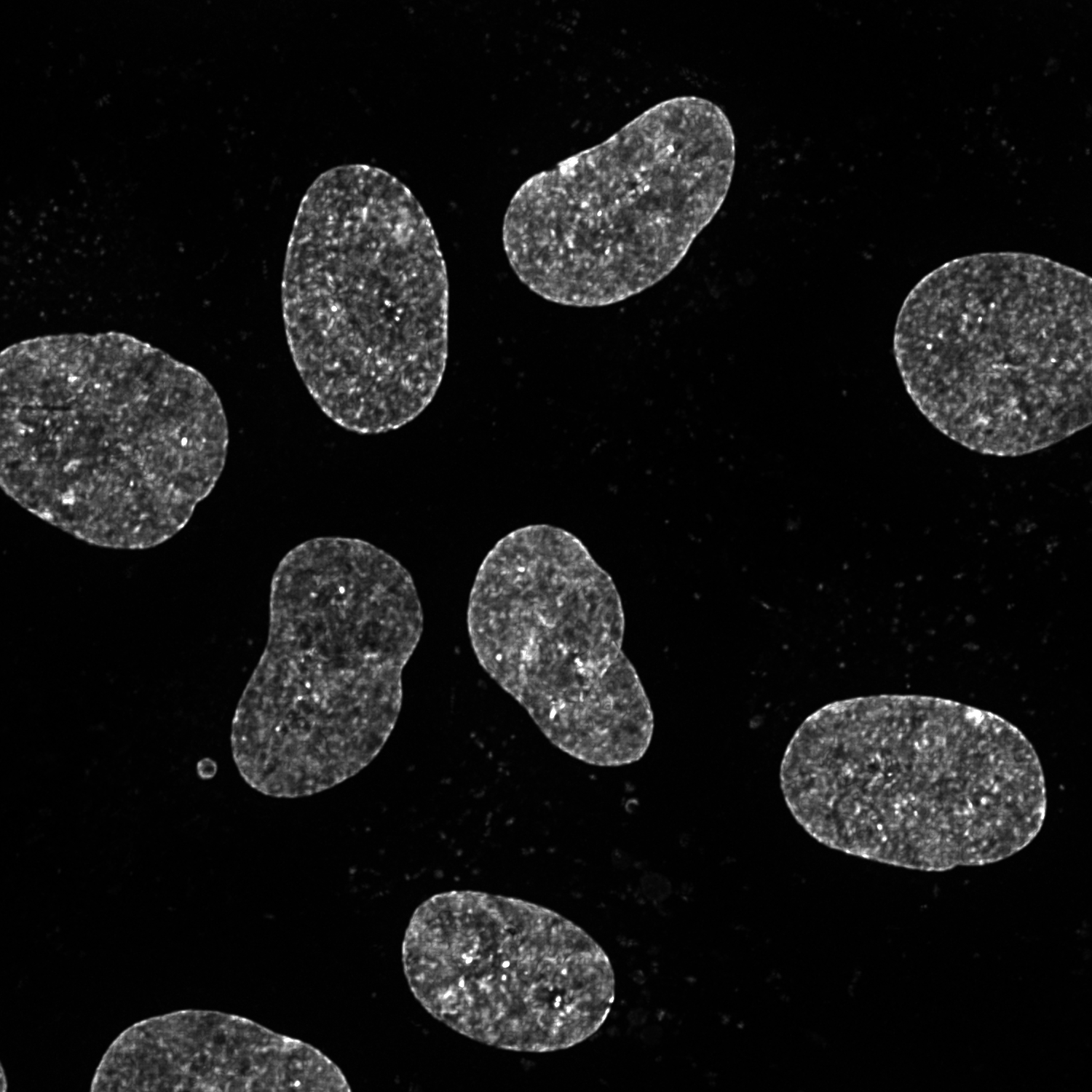

Supplement: Supplementary file 15 — Source data Figure EV3 [file 44318_2024_337_MOESM15_ESM.zip › 09_Figure_EV3/A/HDR-MMEJ-H2BM/_FULL-RANGE-HDR-MMEJ-H2BM.tif]

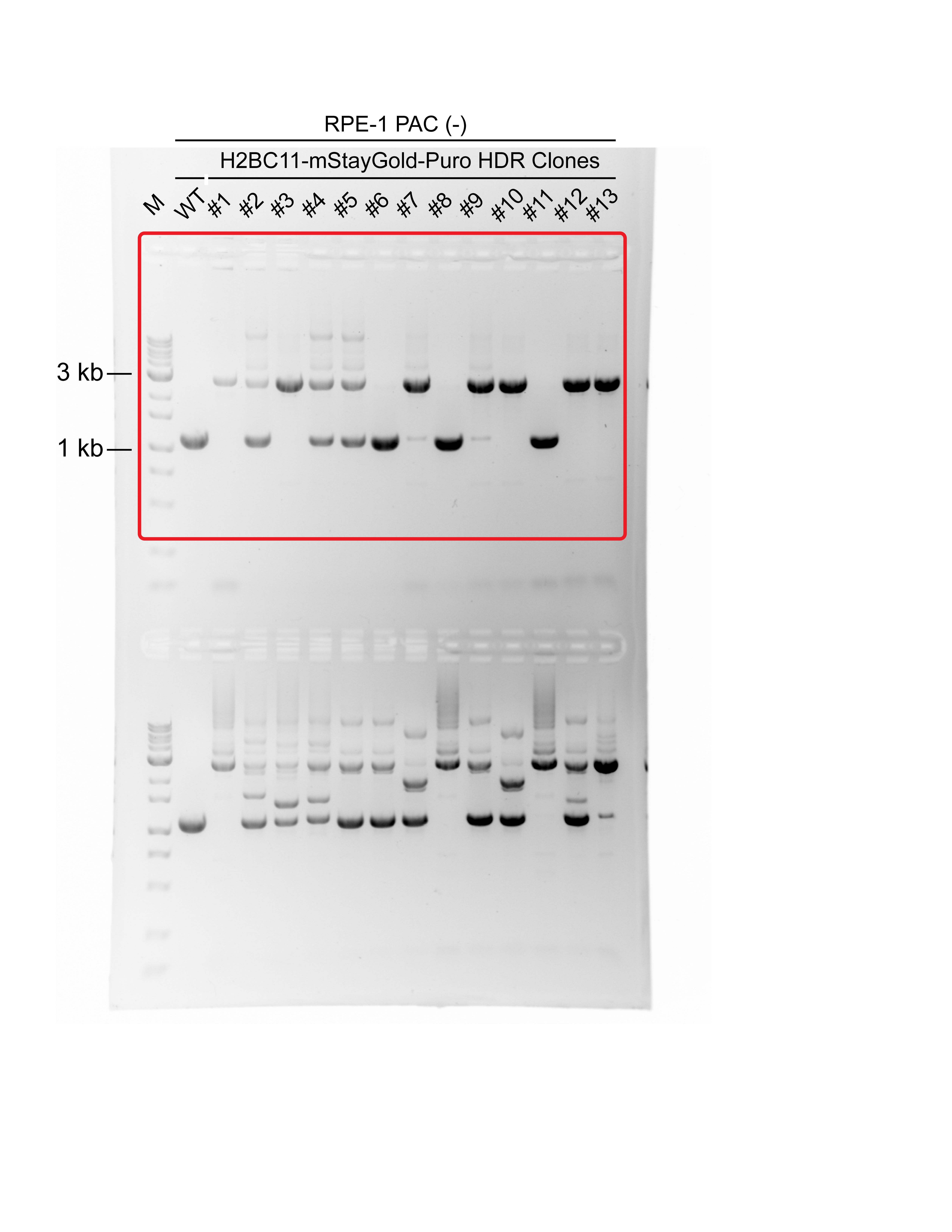

Supplement: Supplementary file 15 — Source data Figure EV3 [file 44318_2024_337_MOESM15_ESM.zip › 09_Figure_EV3/D/H2BC11-HDR-Clonal-Outcomes.tif]

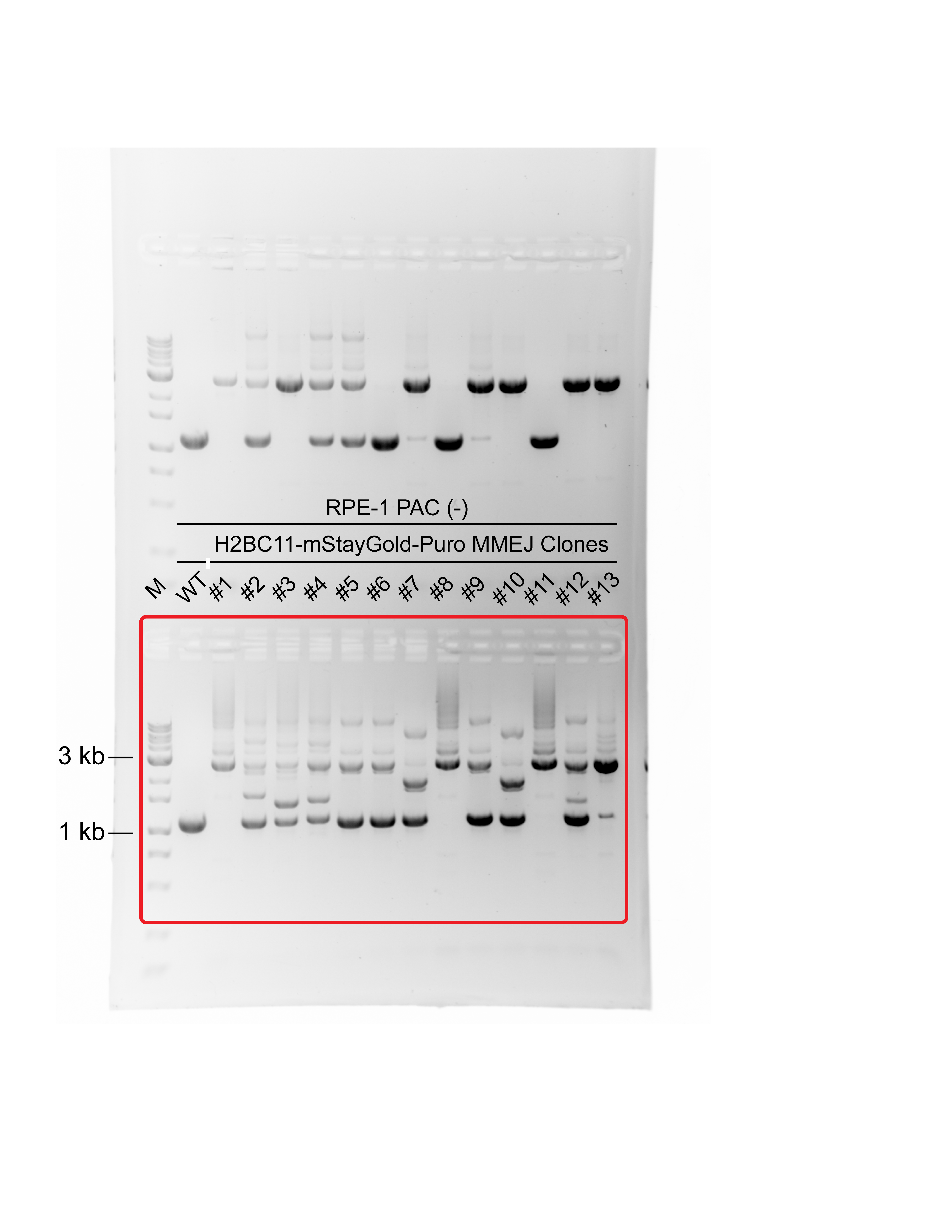

Supplement: Supplementary file 15 — Source data Figure EV3 [file 44318_2024_337_MOESM15_ESM.zip › 09_Figure_EV3/E/H2BC11-MMEJ-Clonal-Outcomes.tif]

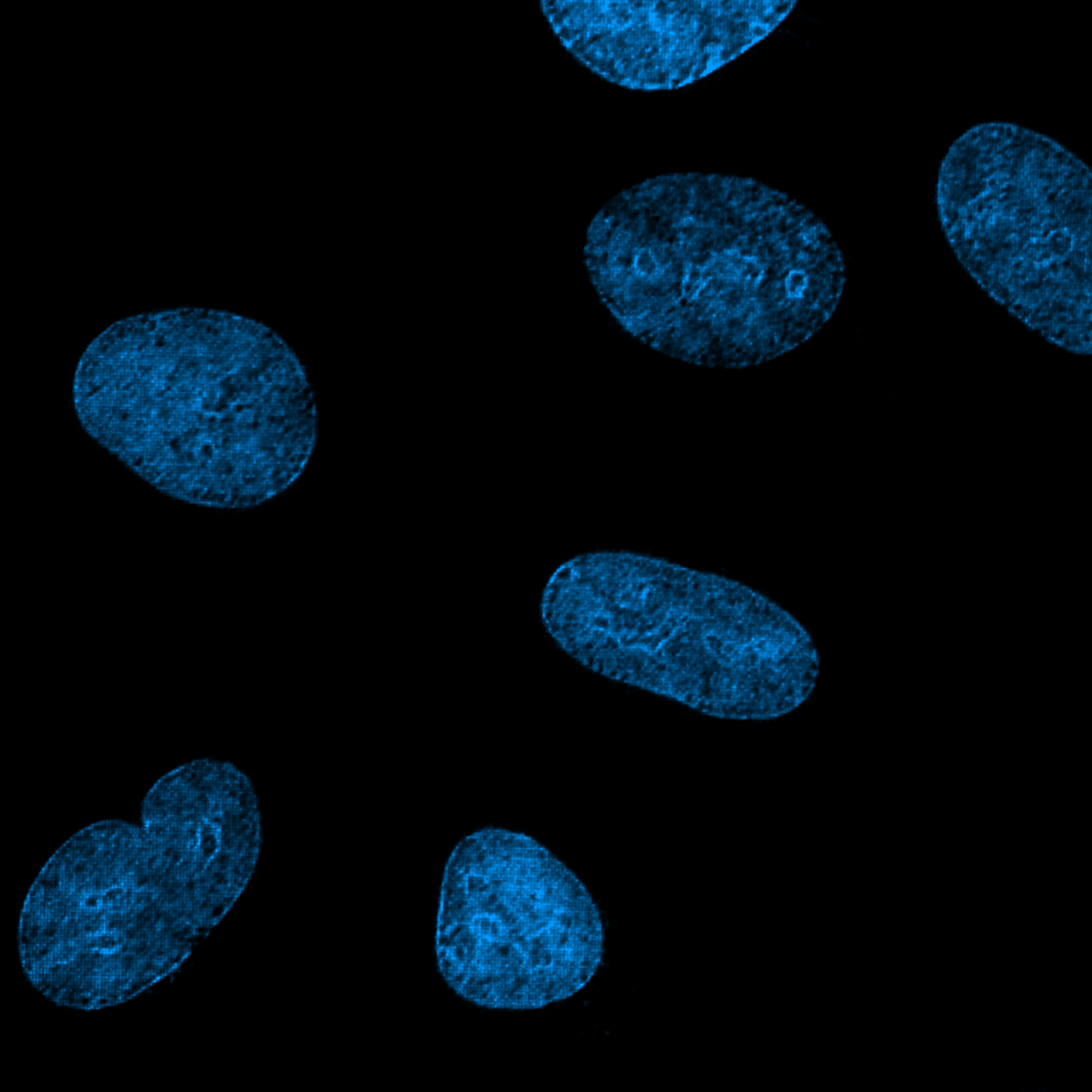

Supplement: Supplementary file 16 — Source data Figure EV4 [file 44318_2024_337_MOESM16_ESM.zip › 10_Figure_EV4/A/NONFLUO-FLUO-CTRL/NONFLUO-FLUO-CTRL_DAPI.tif]

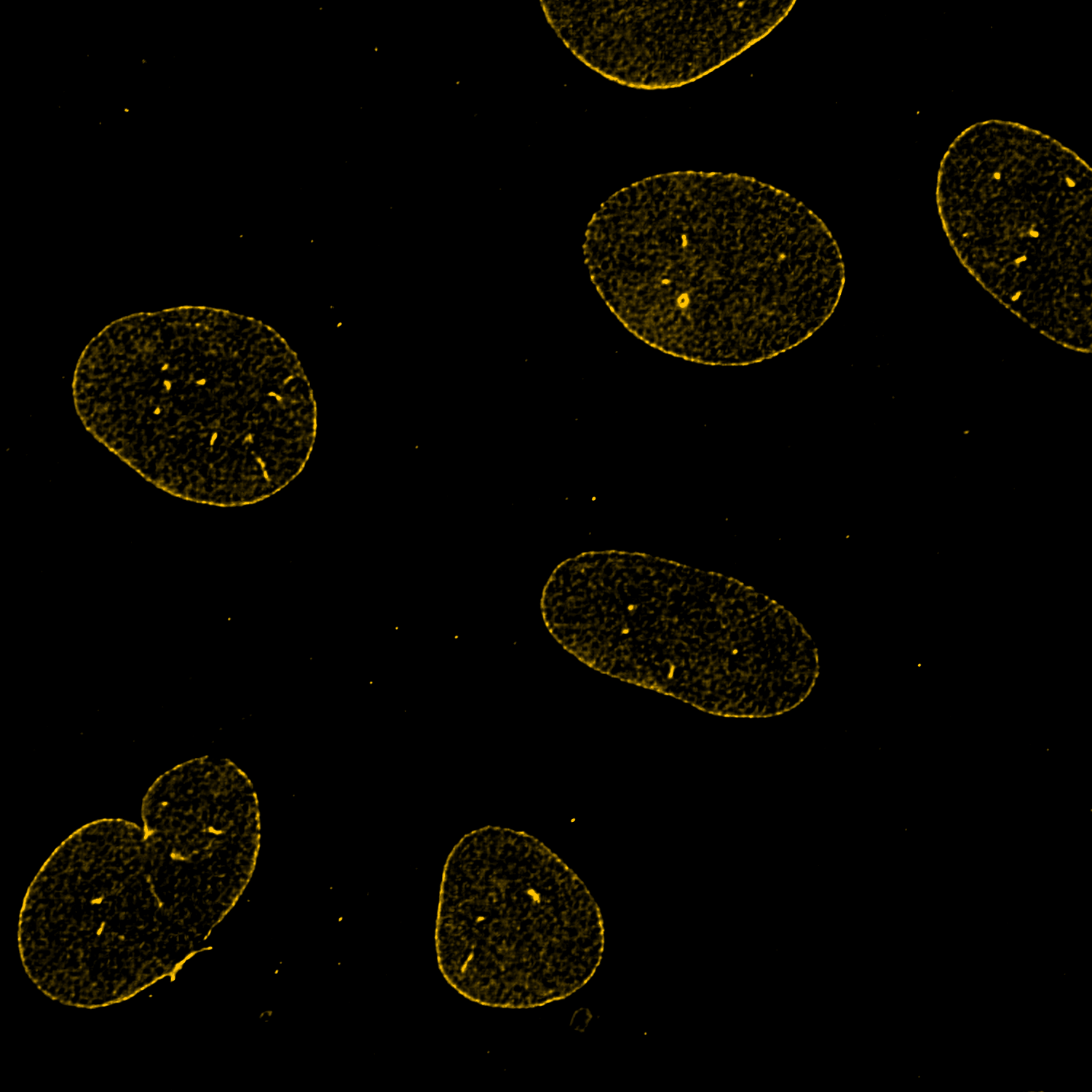

Supplement: Supplementary file 16 — Source data Figure EV4 [file 44318_2024_337_MOESM16_ESM.zip › 10_Figure_EV4/A/NONFLUO-FLUO-CTRL/NONFLUO-FLUO-CTRL_LMNB1.tif]

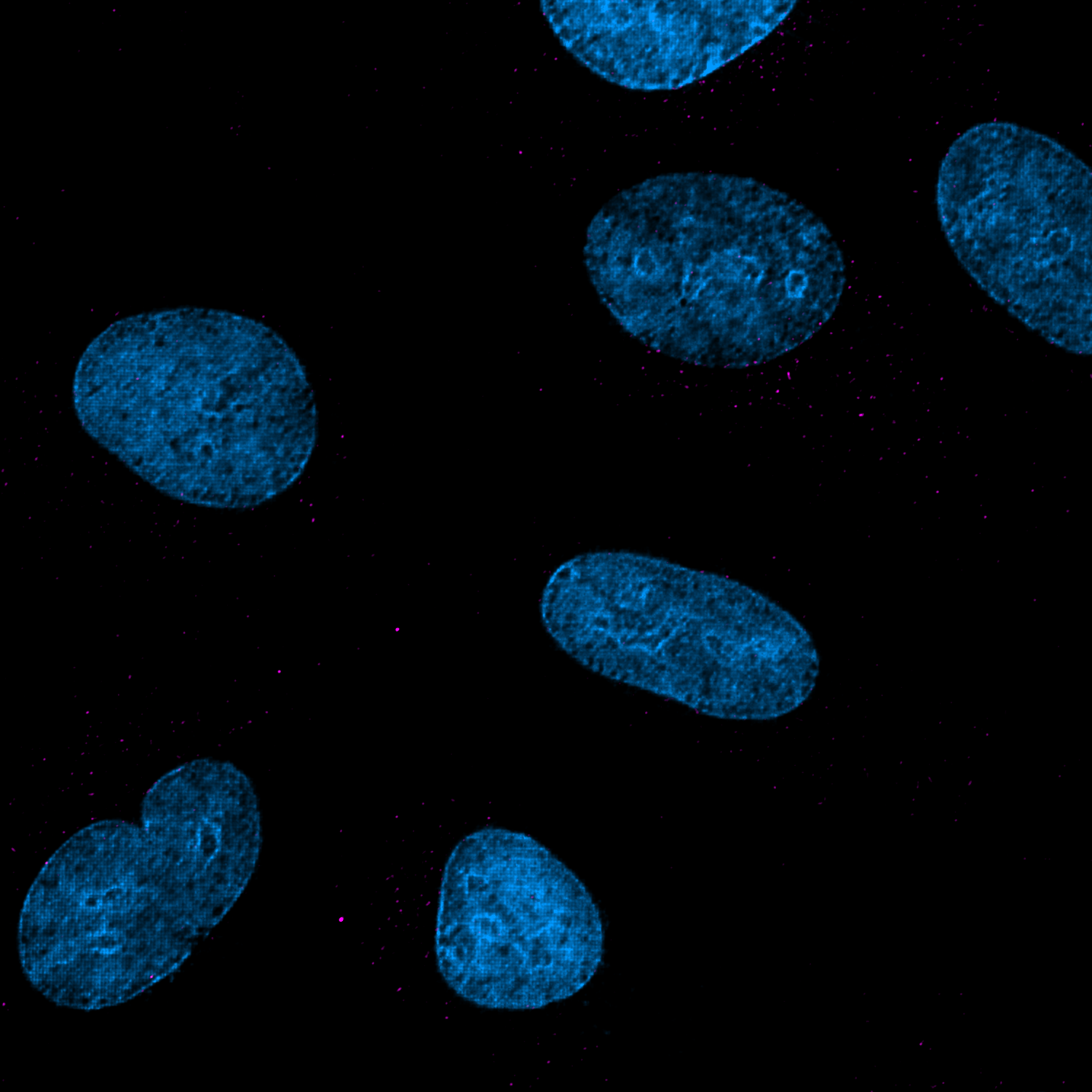

Supplement: Supplementary file 16 — Source data Figure EV4 [file 44318_2024_337_MOESM16_ESM.zip › 10_Figure_EV4/A/NONFLUO-FLUO-CTRL/NONFLUO-FLUO-CTRL_Merge.tif]

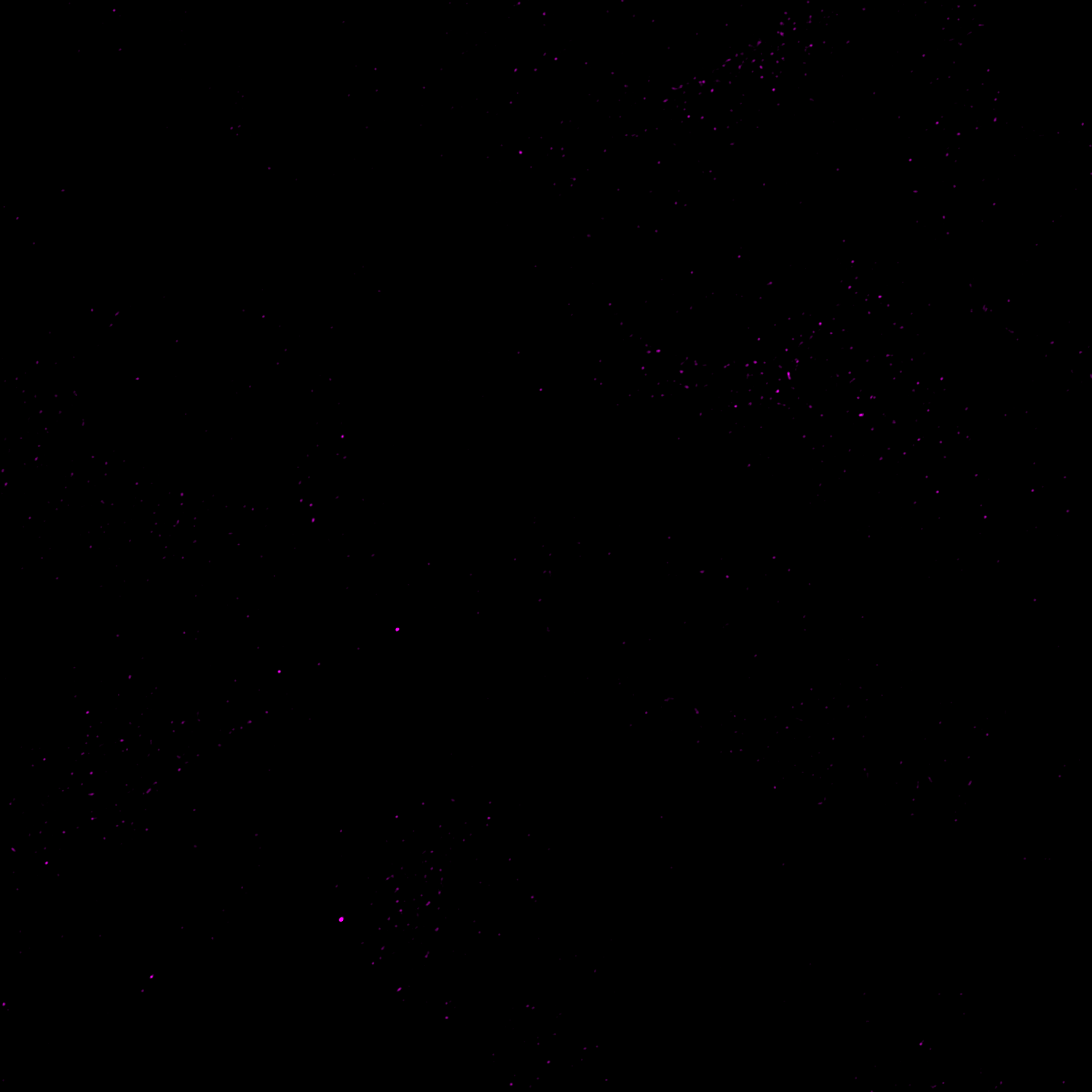

Supplement: Supplementary file 16 — Source data Figure EV4 [file 44318_2024_337_MOESM16_ESM.zip › 10_Figure_EV4/A/NONFLUO-FLUO-CTRL/NONFLUO-FLUO-CTRL_V5.tif]

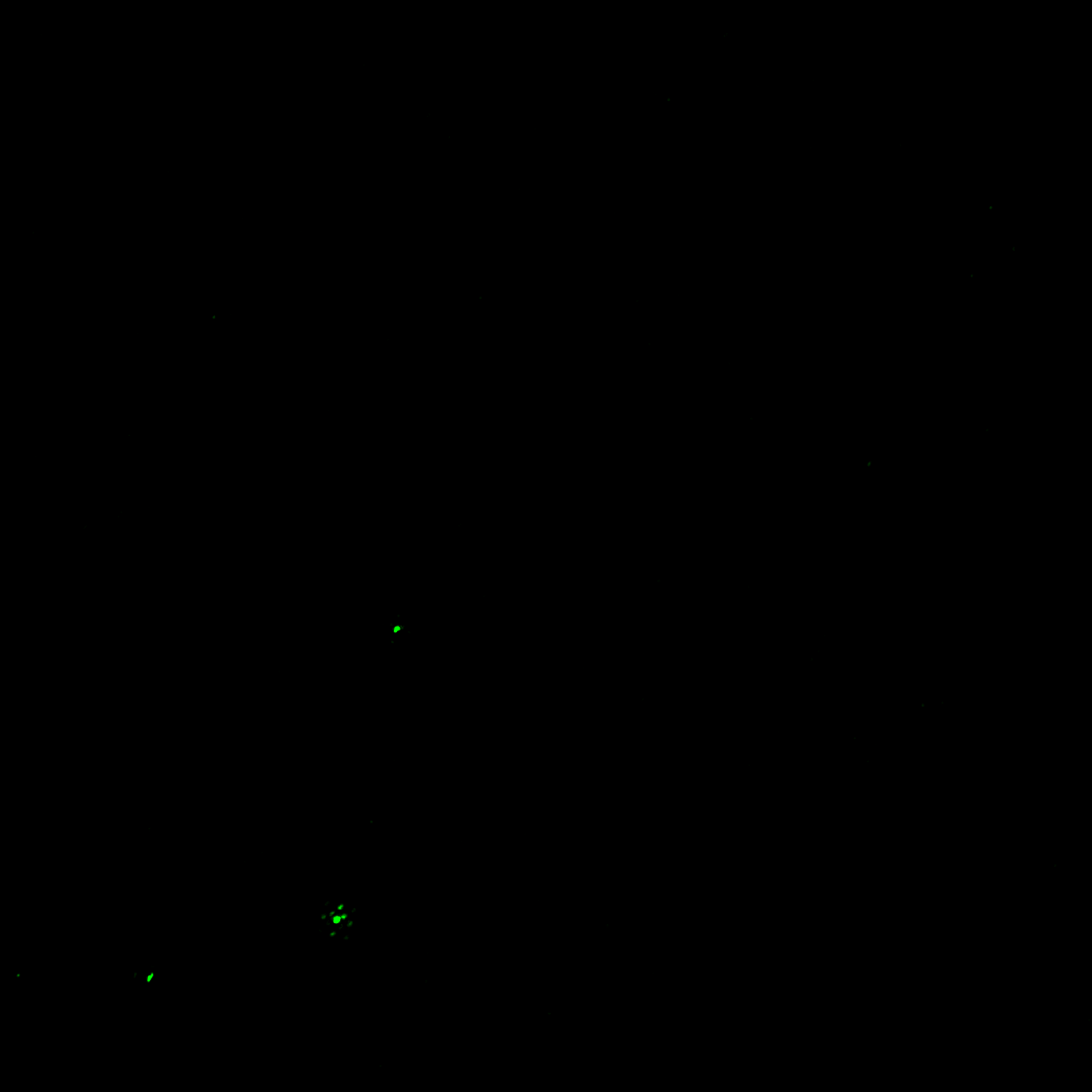

Supplement: Supplementary file 16 — Source data Figure EV4 [file 44318_2024_337_MOESM16_ESM.zip › 10_Figure_EV4/A/NONFLUO-FLUO-CTRL/NONFLUO-FLUO-mStayGold.tif]

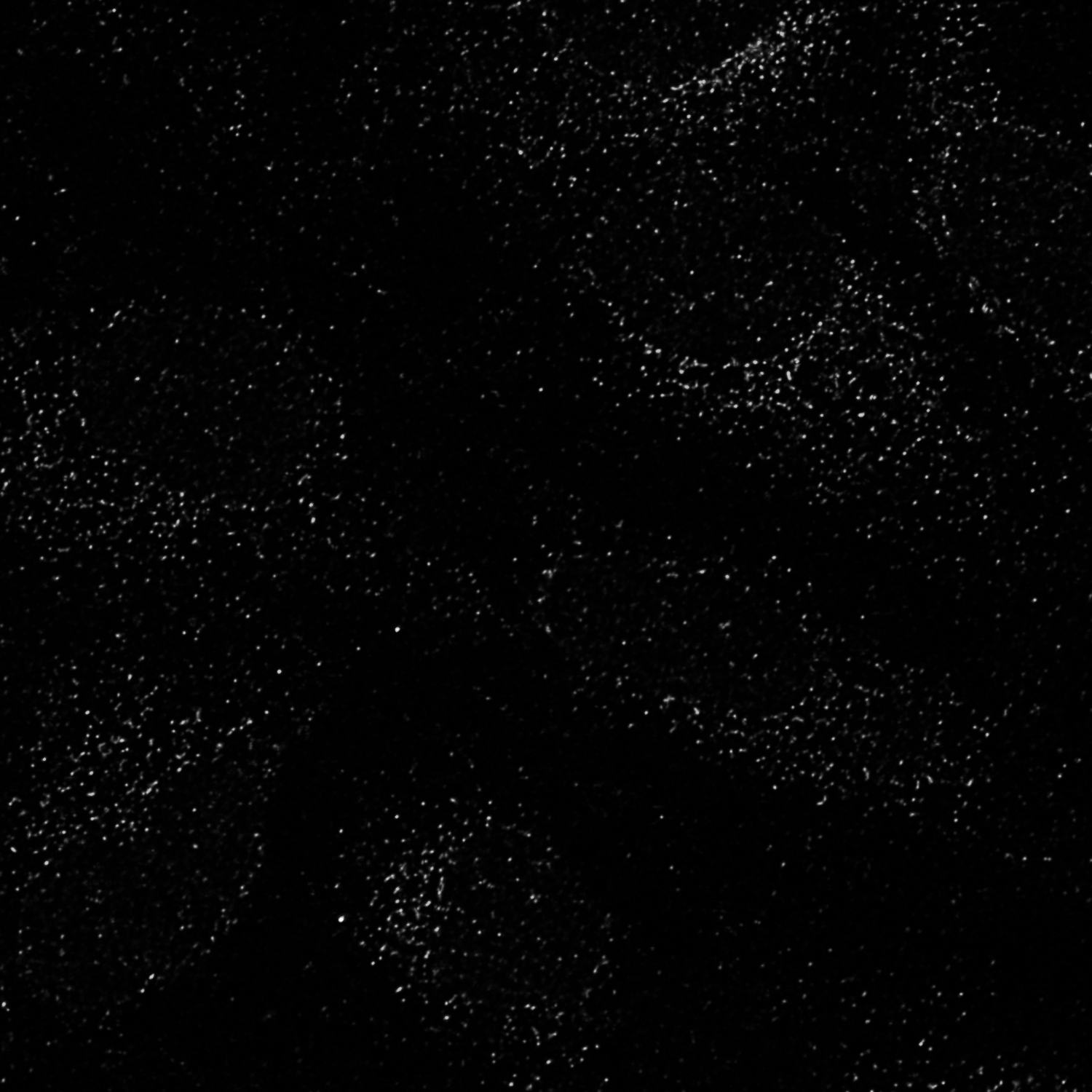

Supplement: Supplementary file 16 — Source data Figure EV4 [file 44318_2024_337_MOESM16_ESM.zip › 10_Figure_EV4/A/NONFLUO-FLUO-CTRL/_FULL-RANGE-NONFLUO-FLUO-CTRL.tif]

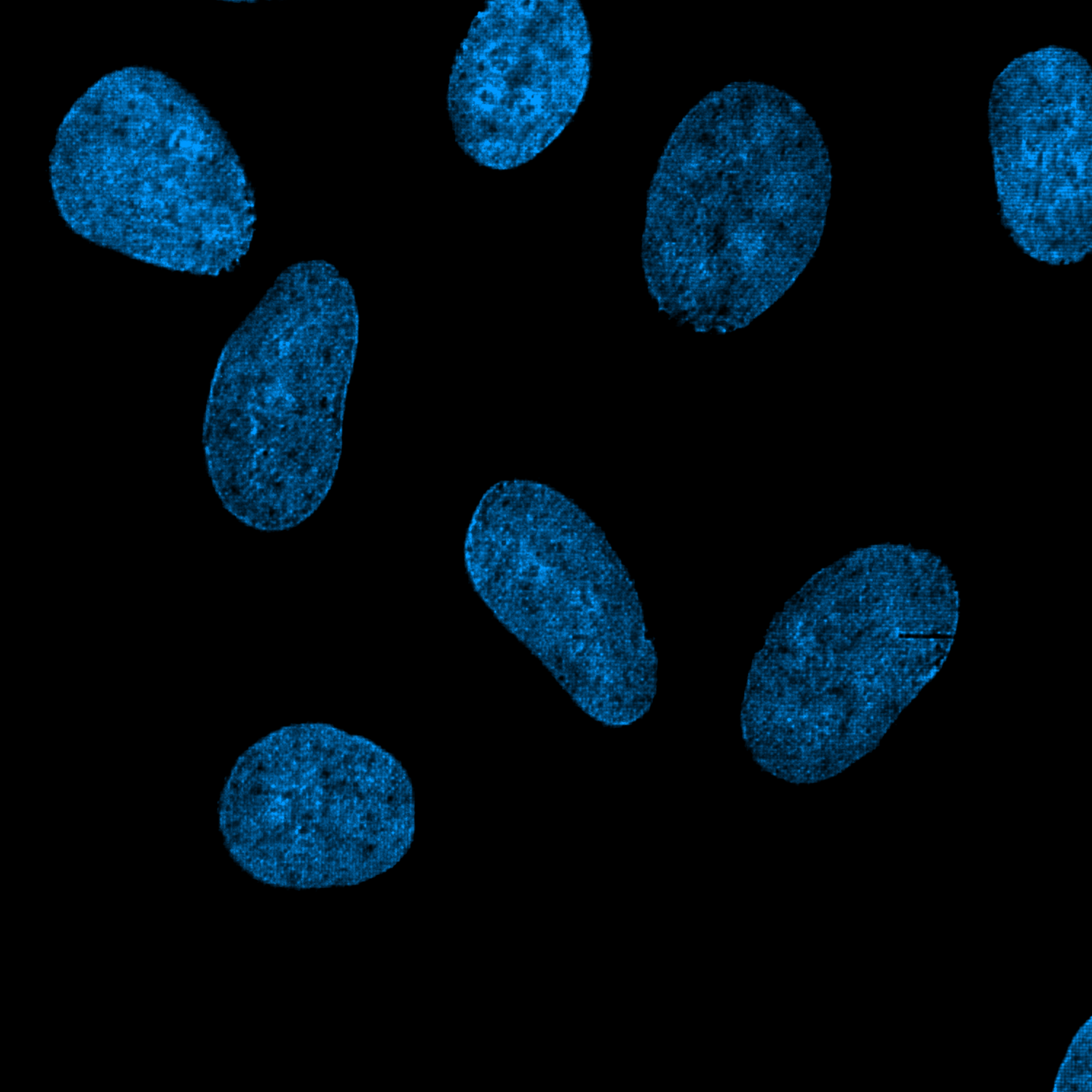

Supplement: Supplementary file 16 — Source data Figure EV4 [file 44318_2024_337_MOESM16_ESM.zip › 10_Figure_EV4/A/NONFLUO-FLUO-dTAG/NONFLUO-FLUO-dTAG_DAPI.tif]

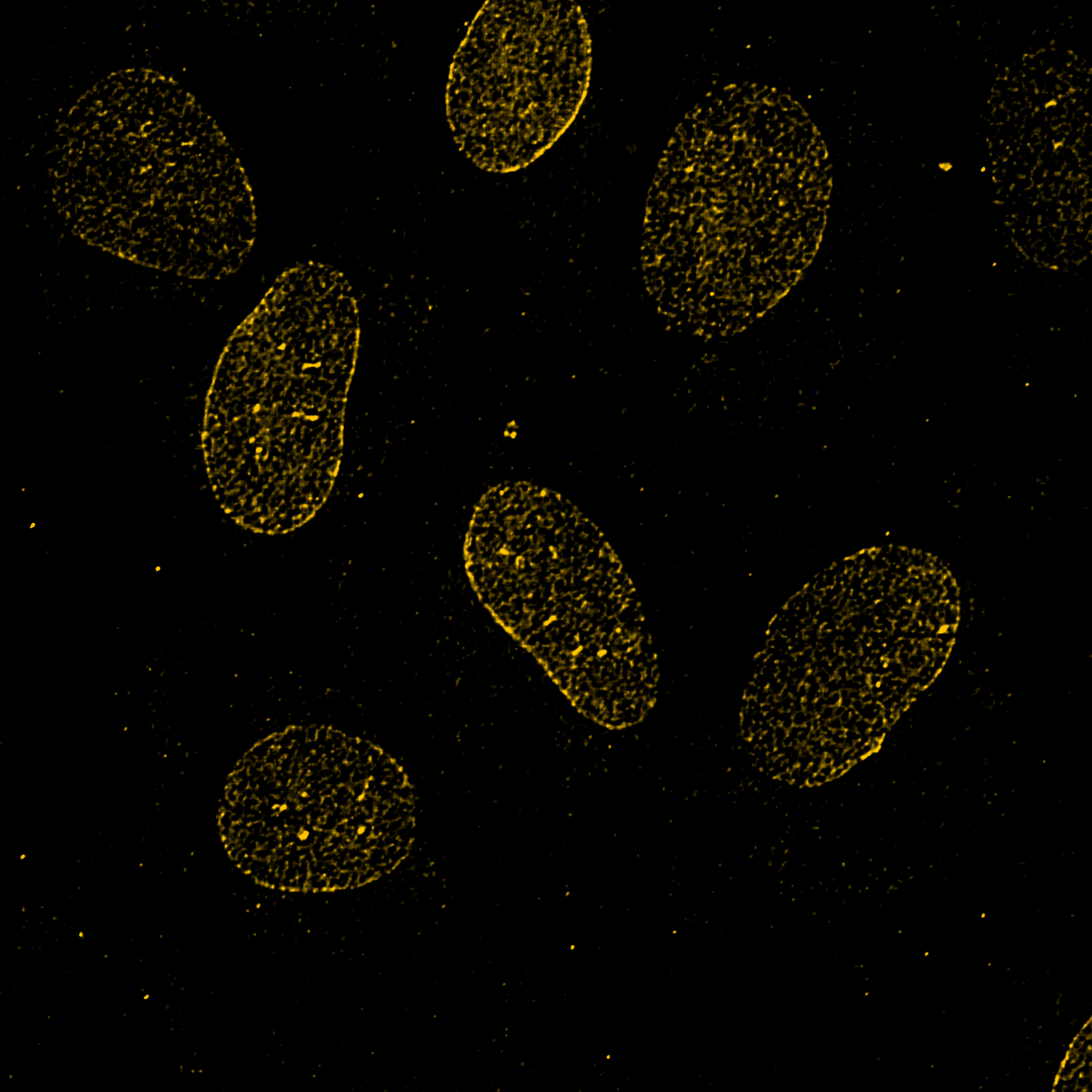

Supplement: Supplementary file 16 — Source data Figure EV4 [file 44318_2024_337_MOESM16_ESM.zip › 10_Figure_EV4/A/NONFLUO-FLUO-dTAG/NONFLUO-FLUO-dTAG_LMNB1.tif]

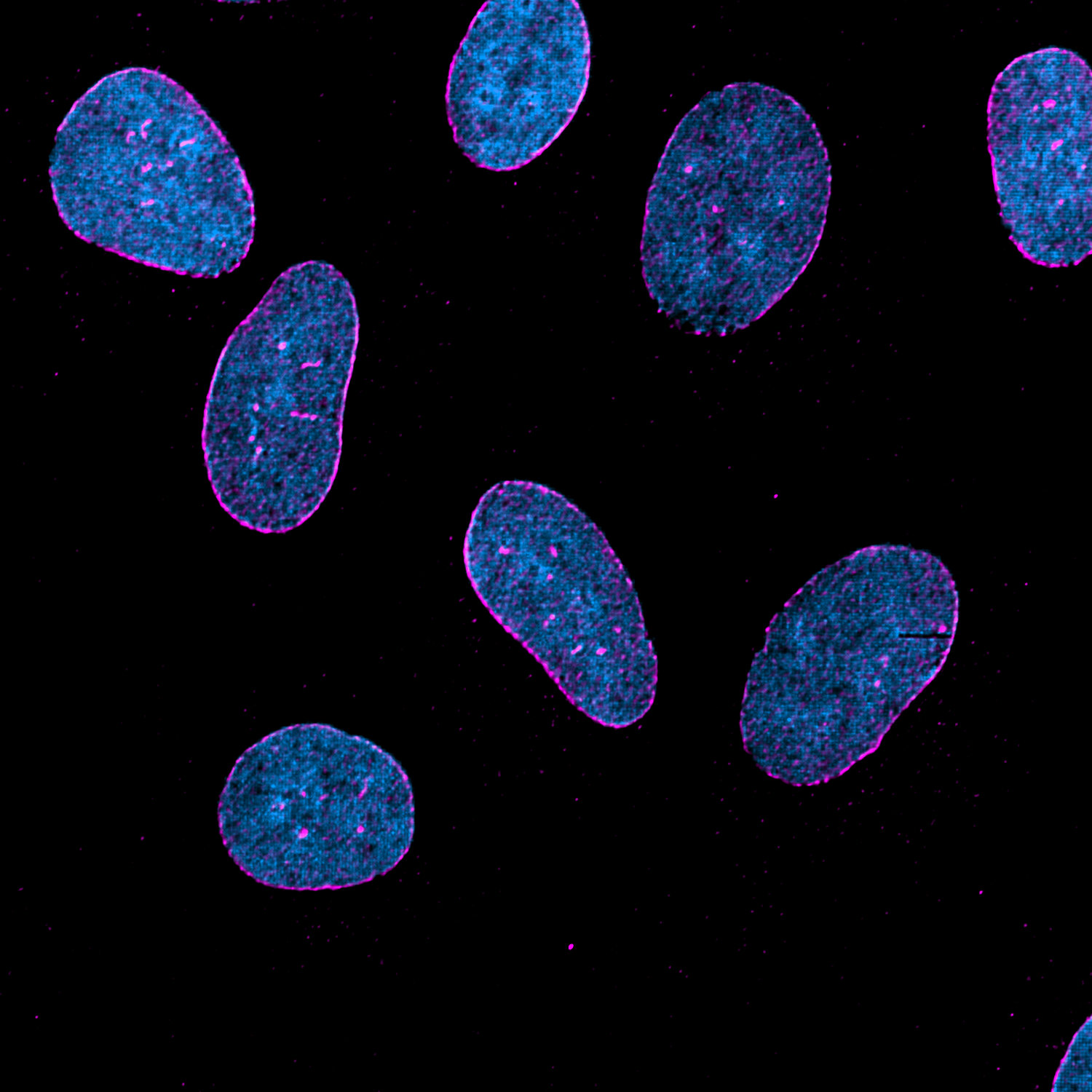

Supplement: Supplementary file 16 — Source data Figure EV4 [file 44318_2024_337_MOESM16_ESM.zip › 10_Figure_EV4/A/NONFLUO-FLUO-dTAG/NONFLUO-FLUO-dTAG_Merge.tif]

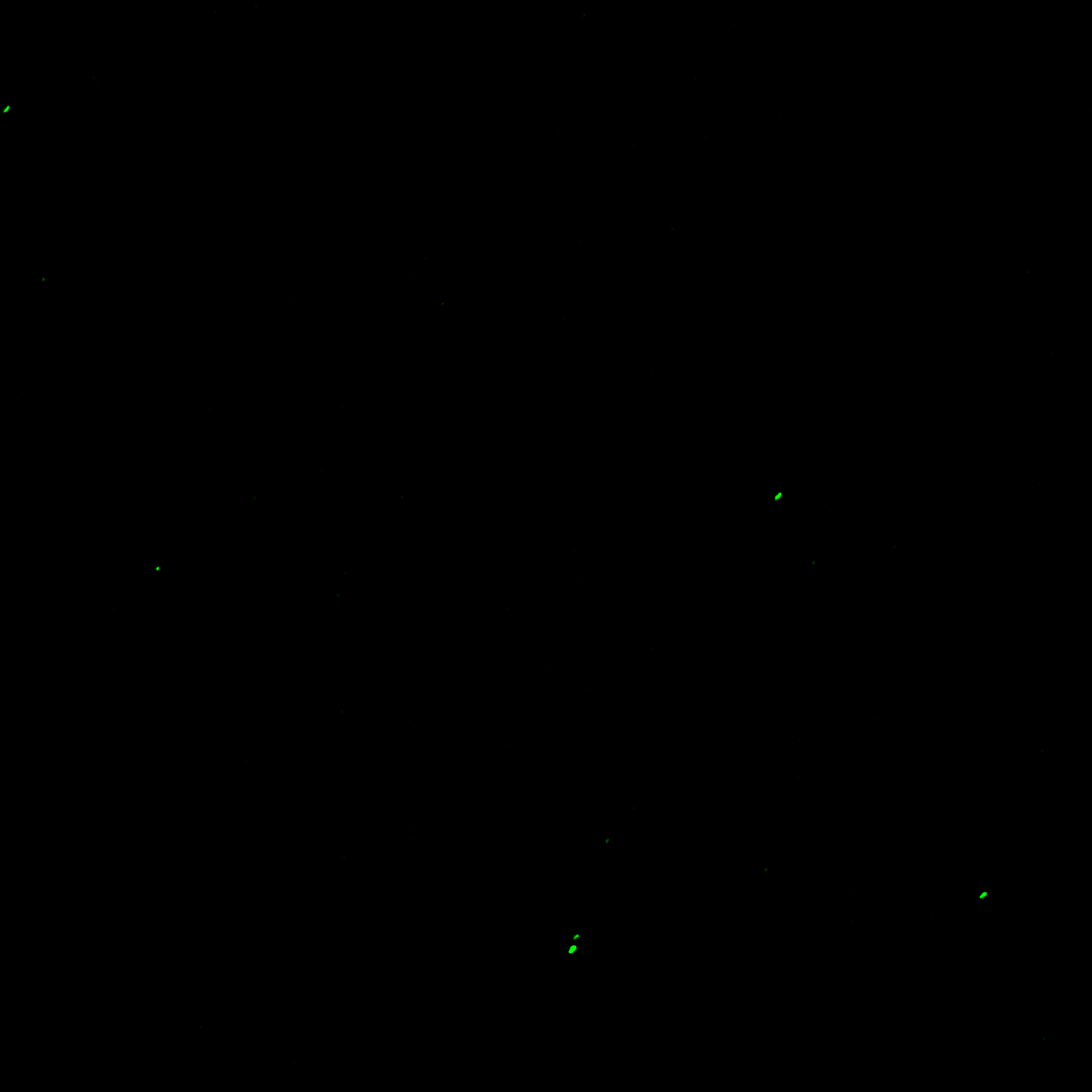

Supplement: Supplementary file 16 — Source data Figure EV4 [file 44318_2024_337_MOESM16_ESM.zip › 10_Figure_EV4/A/NONFLUO-FLUO-dTAG/NONFLUO-FLUO-dTAG_mStayGold.tif]

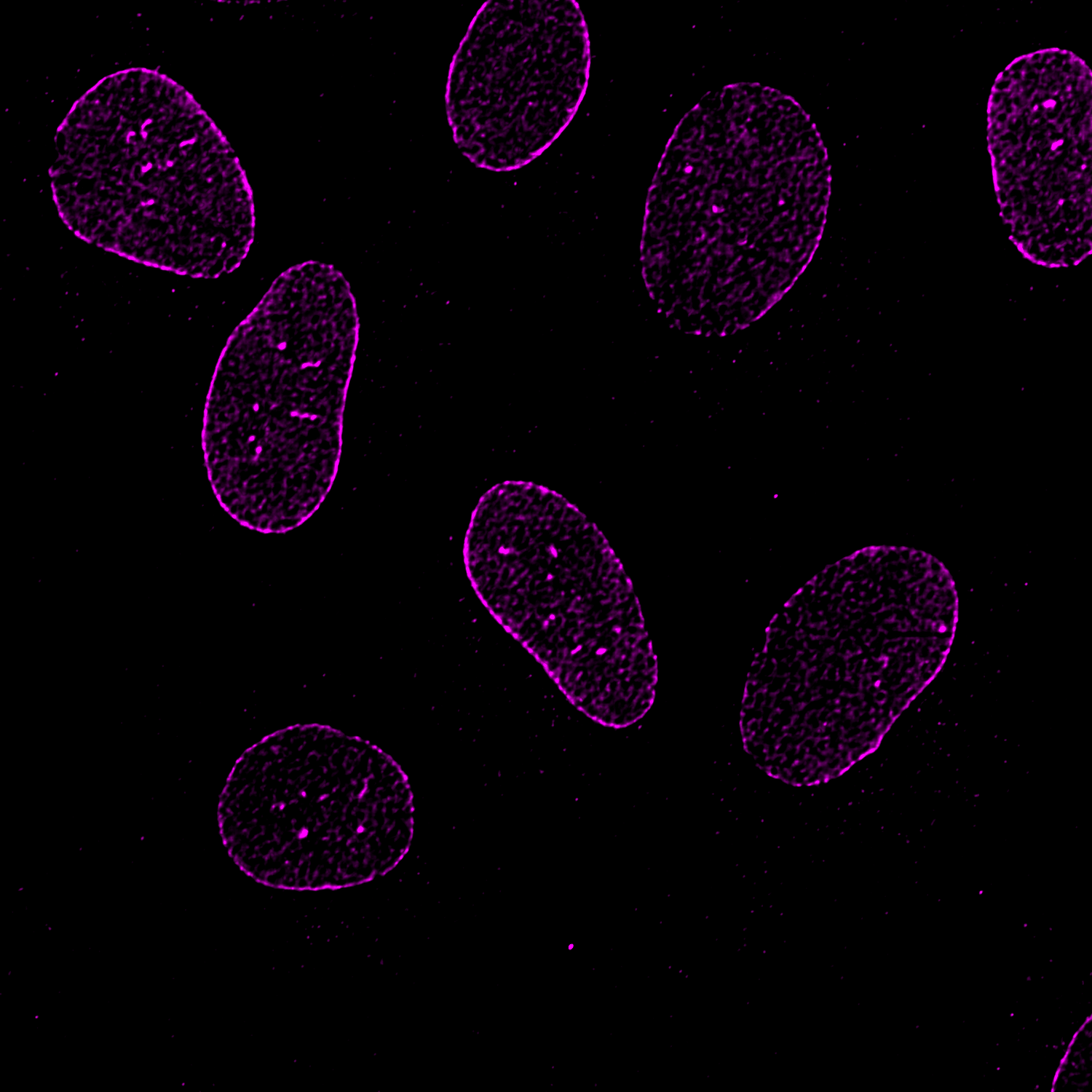

Supplement: Supplementary file 16 — Source data Figure EV4 [file 44318_2024_337_MOESM16_ESM.zip › 10_Figure_EV4/A/NONFLUO-FLUO-dTAG/NONFLUO-FLUO-dTAG_V5.tif]

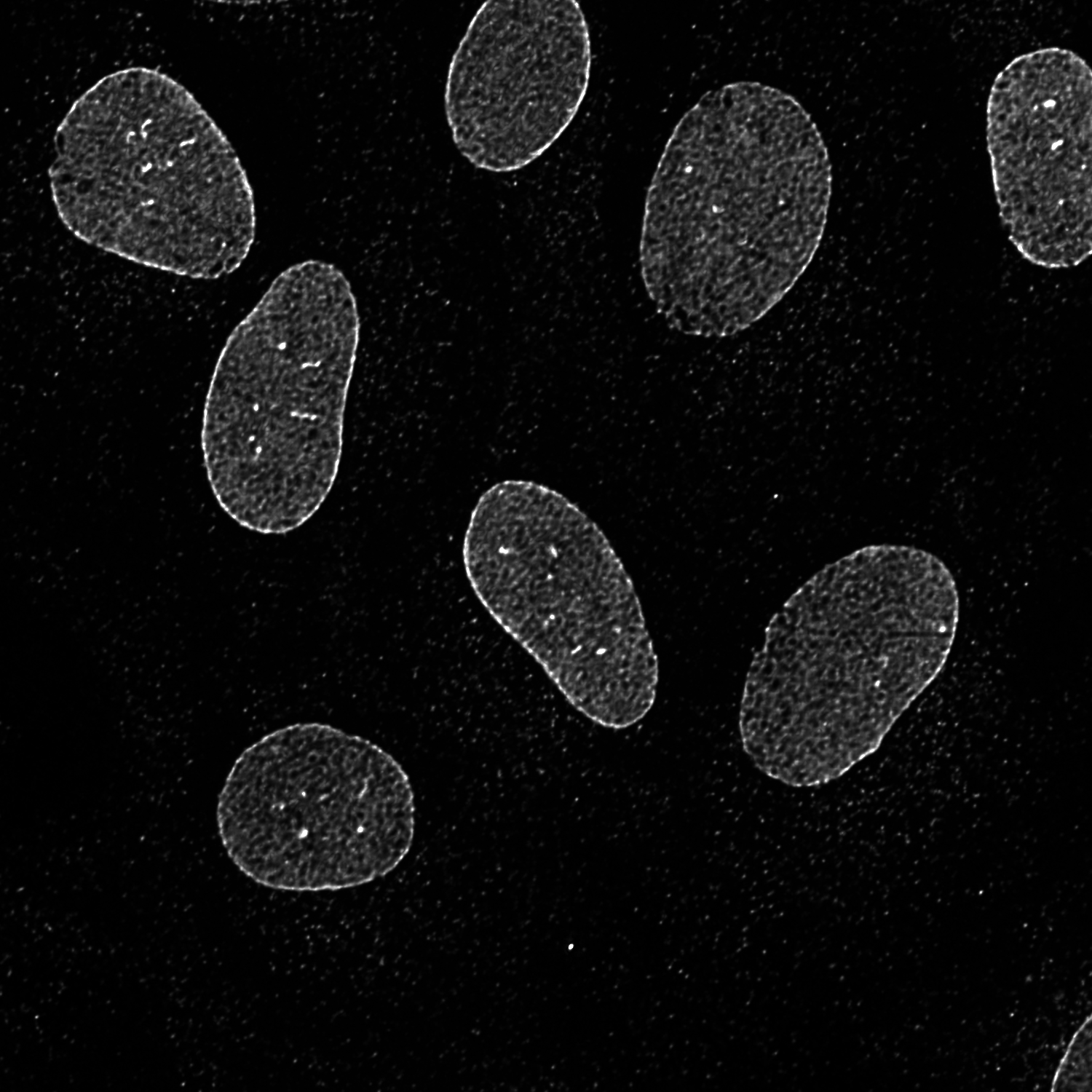

Supplement: Supplementary file 16 — Source data Figure EV4 [file 44318_2024_337_MOESM16_ESM.zip › 10_Figure_EV4/A/NONFLUO-FLUO-dTAG/_FULL-RANGE-NONFLUO-FLUO-dTAG.tif]

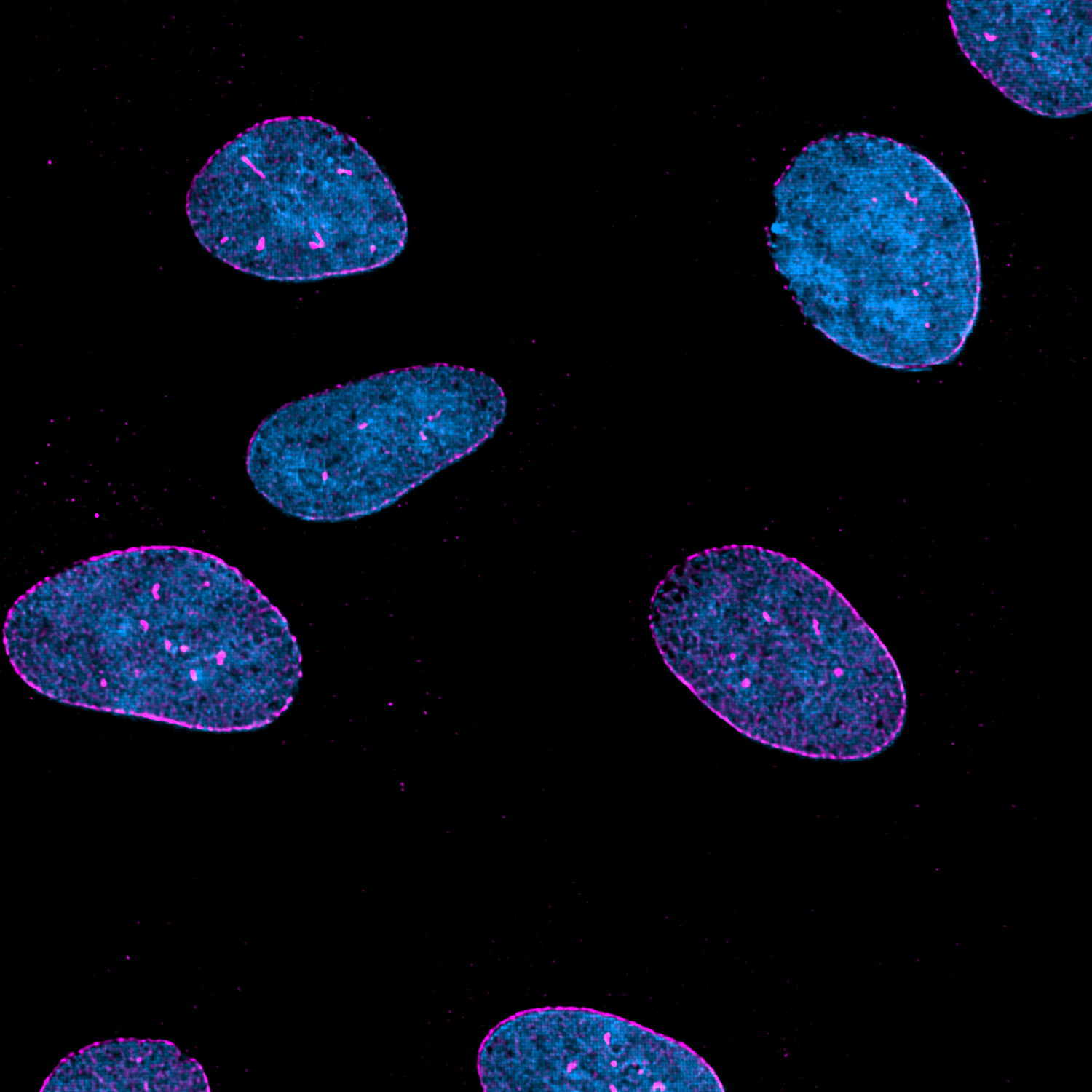

Supplement: Supplementary file 16 — Source data Figure EV4 [file 44318_2024_337_MOESM16_ESM.zip › 10_Figure_EV4/A/NONFLUO-FLUO-STG/NONFLUO-FLUO-STG_RGB.tif]

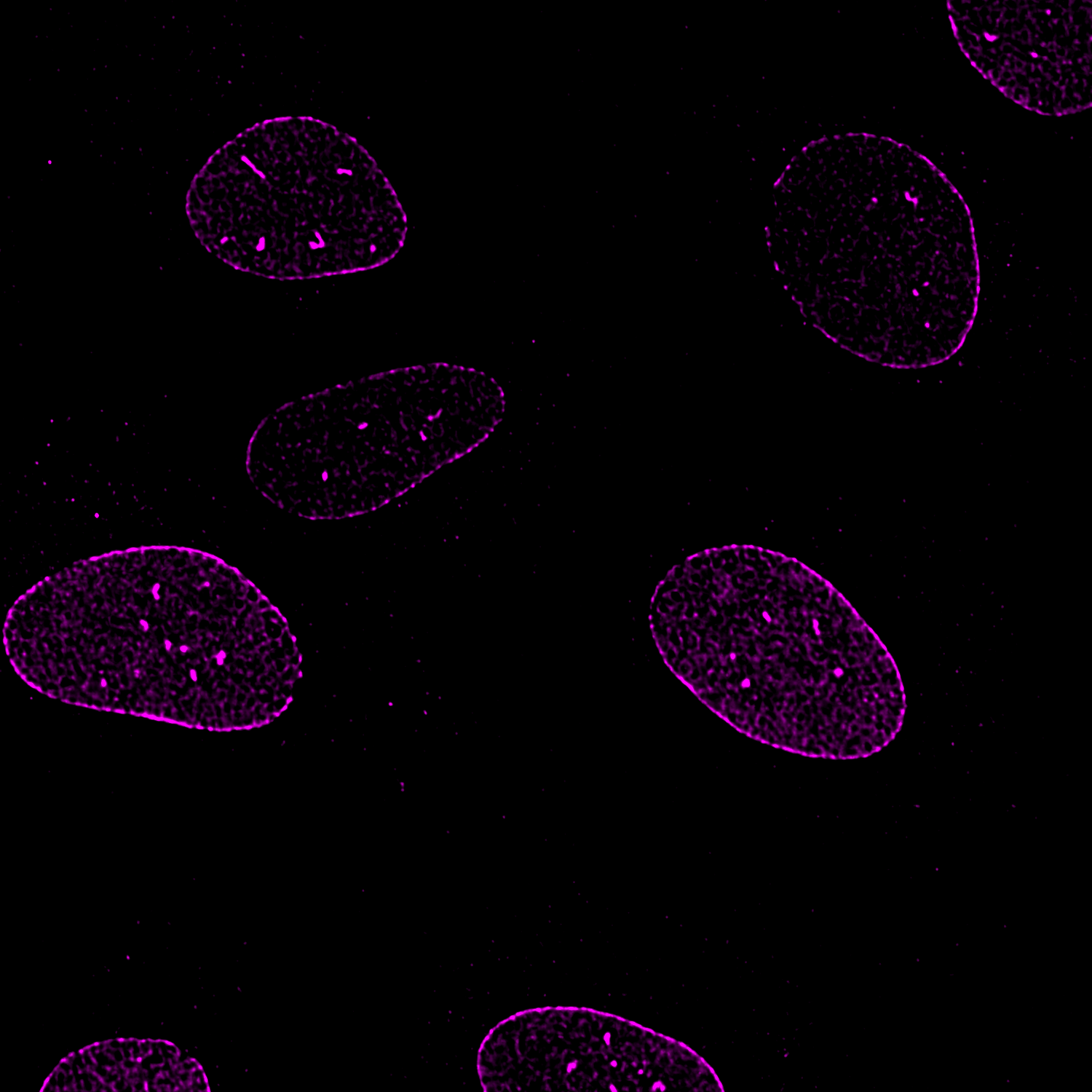

Supplement: Supplementary file 16 — Source data Figure EV4 [file 44318_2024_337_MOESM16_ESM.zip › 10_Figure_EV4/A/NONFLUO-FLUO-STG/NONFLUO-FLUO-STG_RGB_DS CY5 FIX.tif]

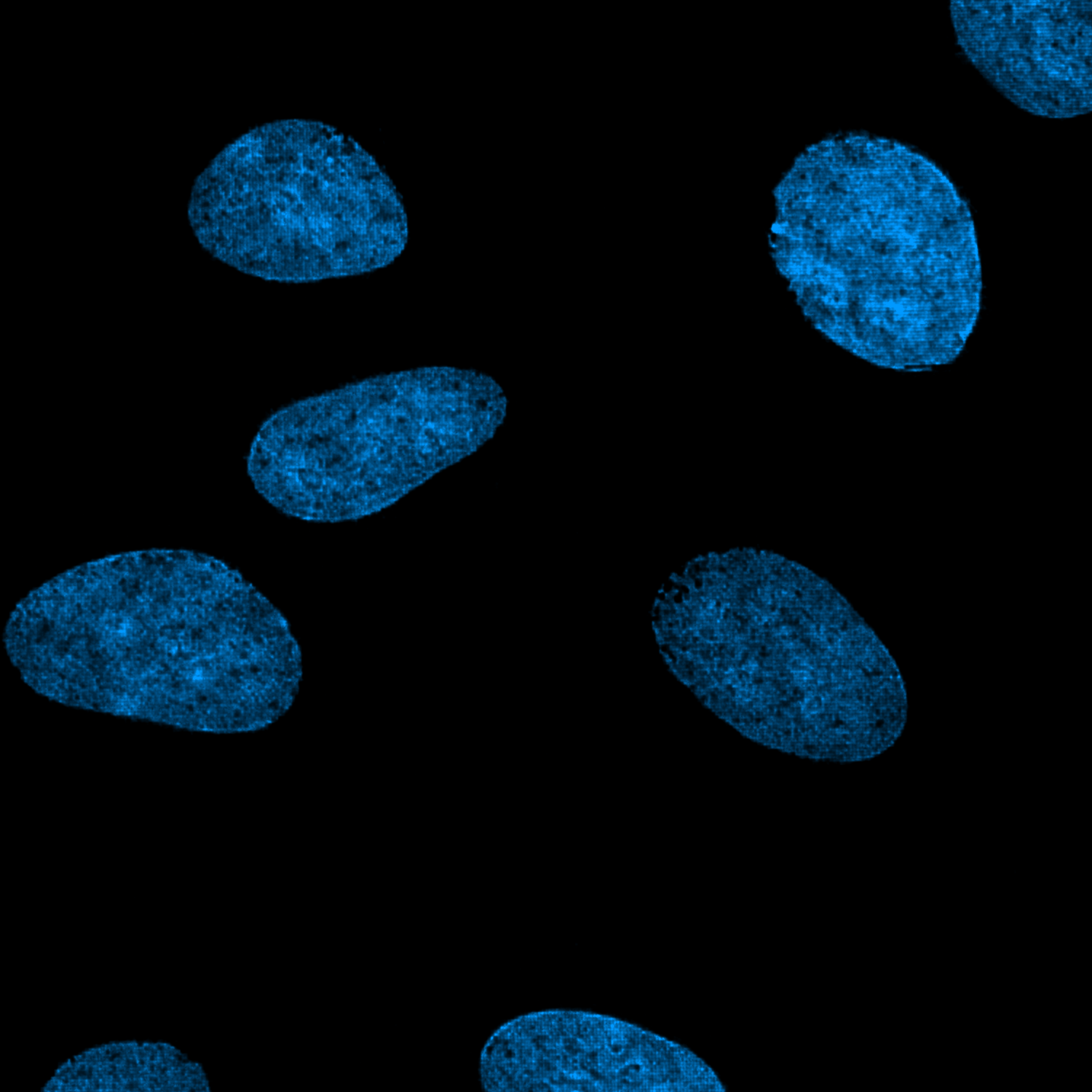

Supplement: Supplementary file 16 — Source data Figure EV4 [file 44318_2024_337_MOESM16_ESM.zip › 10_Figure_EV4/A/NONFLUO-FLUO-STG/NONFLUO-FLUO-STG_RGB_DS DAPI FIX.tif]

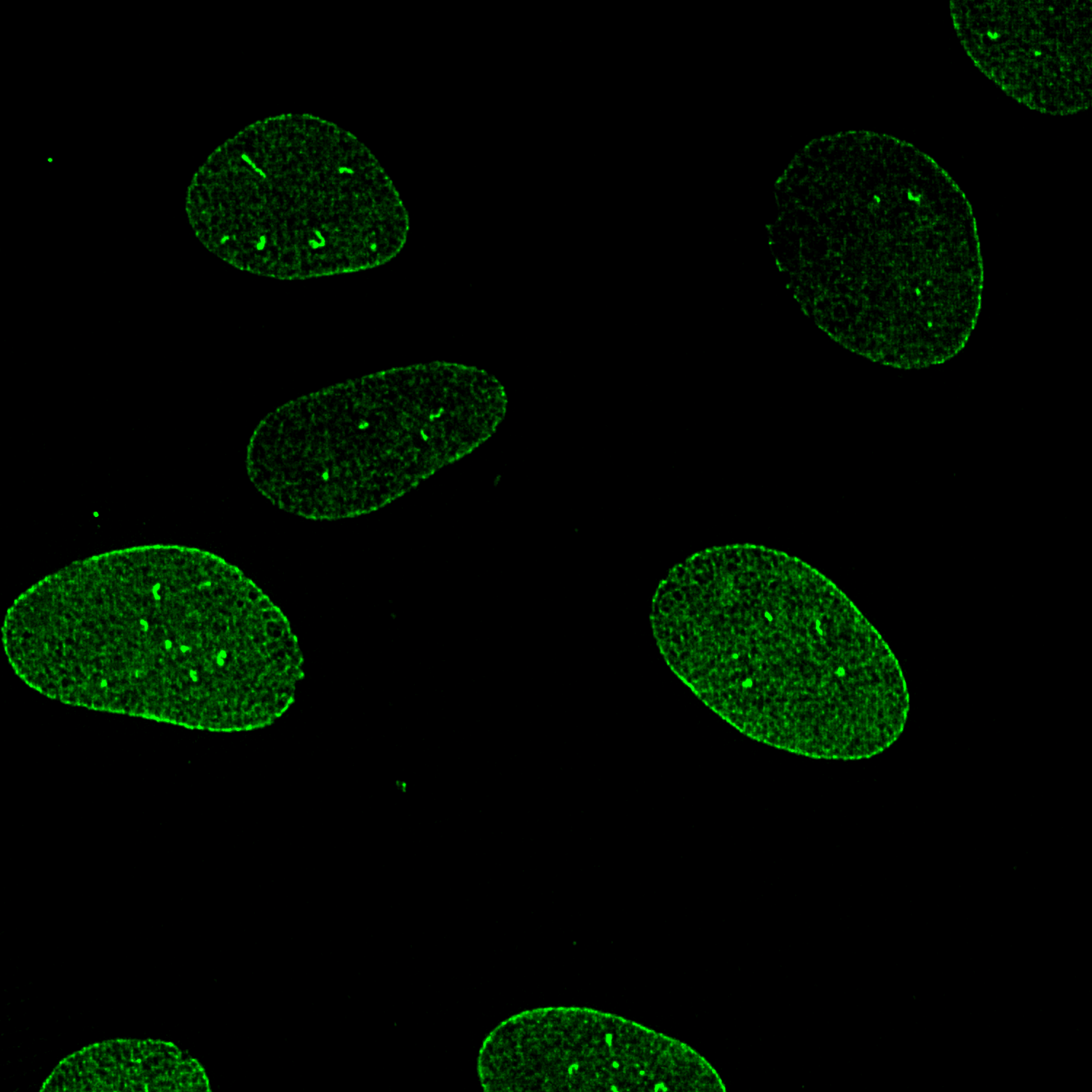

Supplement: Supplementary file 16 — Source data Figure EV4 [file 44318_2024_337_MOESM16_ESM.zip › 10_Figure_EV4/A/NONFLUO-FLUO-STG/NONFLUO-FLUO-STG_RGB_DS GFP FIX.tif]

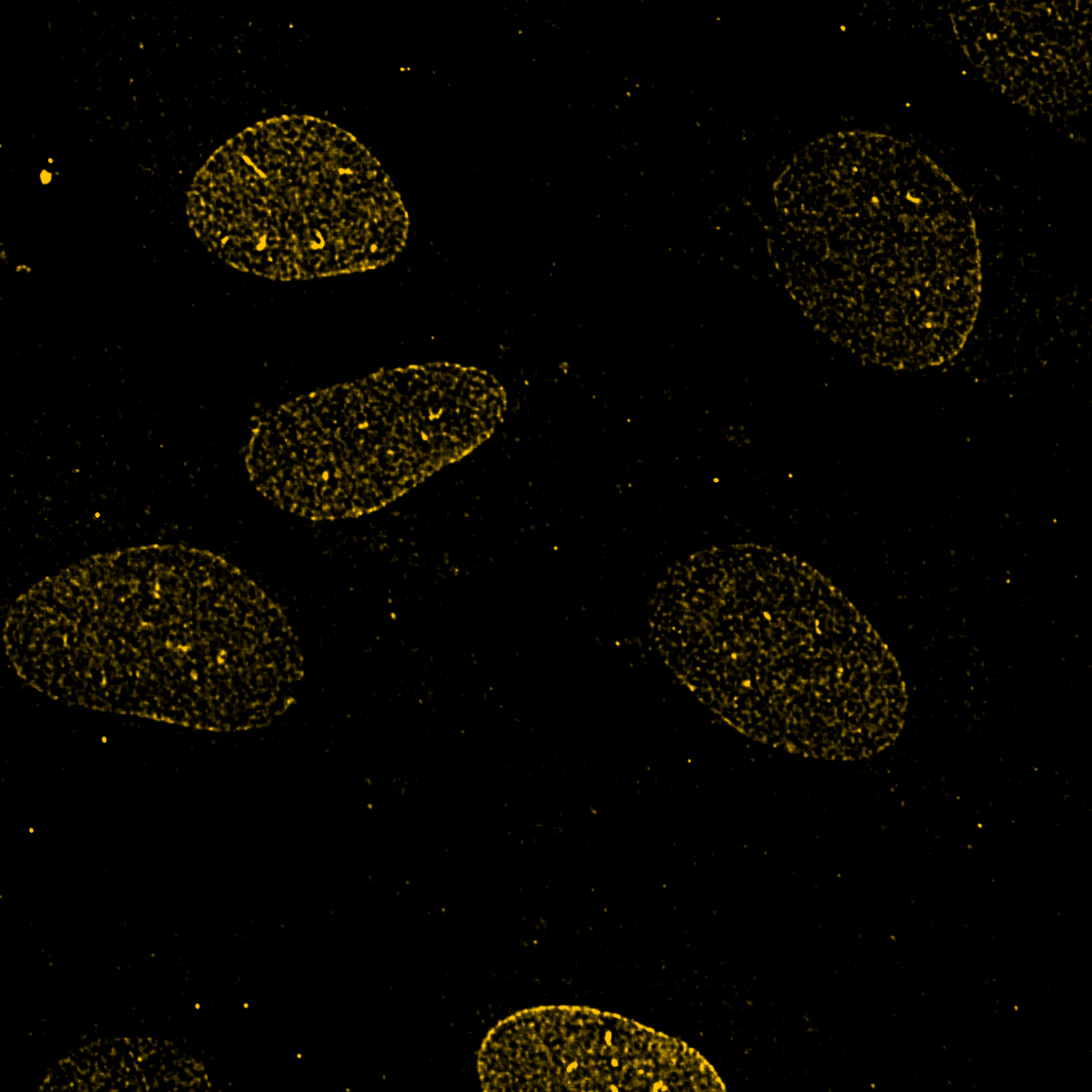

Supplement: Supplementary file 16 — Source data Figure EV4 [file 44318_2024_337_MOESM16_ESM.zip › 10_Figure_EV4/A/NONFLUO-FLUO-STG/NONFLUO-FLUO-STG_RGB_DS RFP FIX.tif]

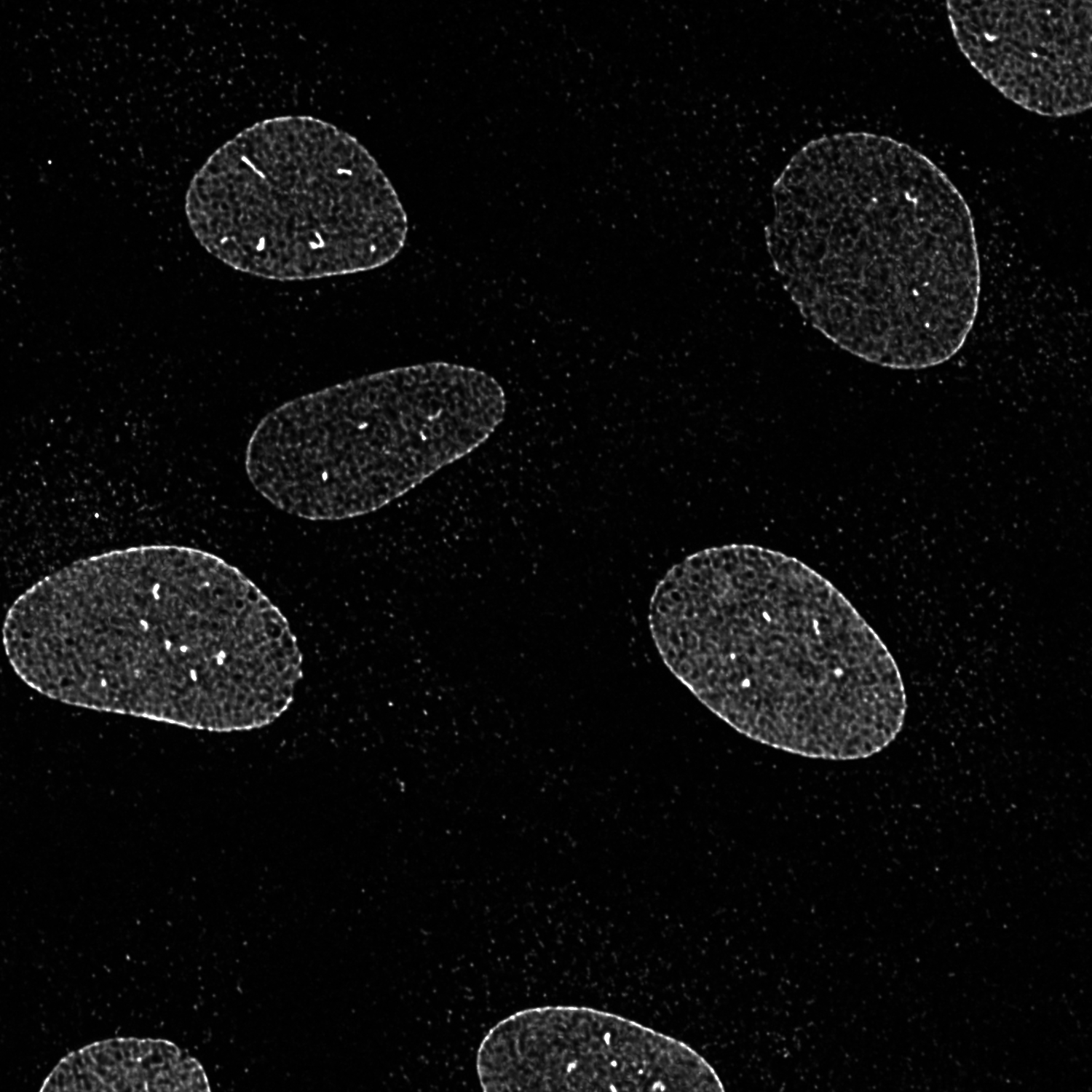

Supplement: Supplementary file 16 — Source data Figure EV4 [file 44318_2024_337_MOESM16_ESM.zip › 10_Figure_EV4/A/NONFLUO-FLUO-STG/_FULL-RANGE-NONFLUO-FLUO-STG.tif]

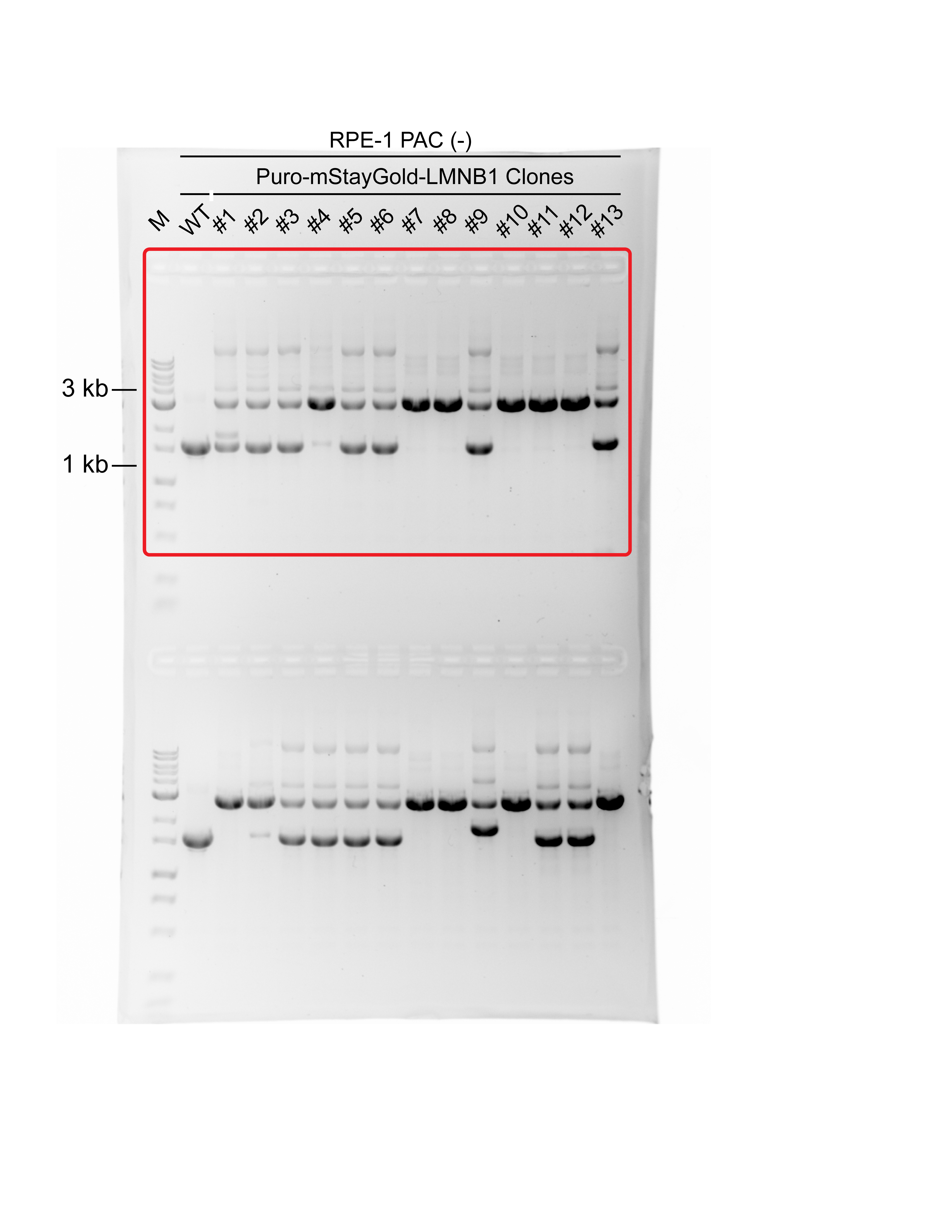

Supplement: Supplementary file 16 — Source data Figure EV4 [file 44318_2024_337_MOESM16_ESM.zip › 10_Figure_EV4/D/mStayGold-LMNB1-Clonal-Outcomes.tif]

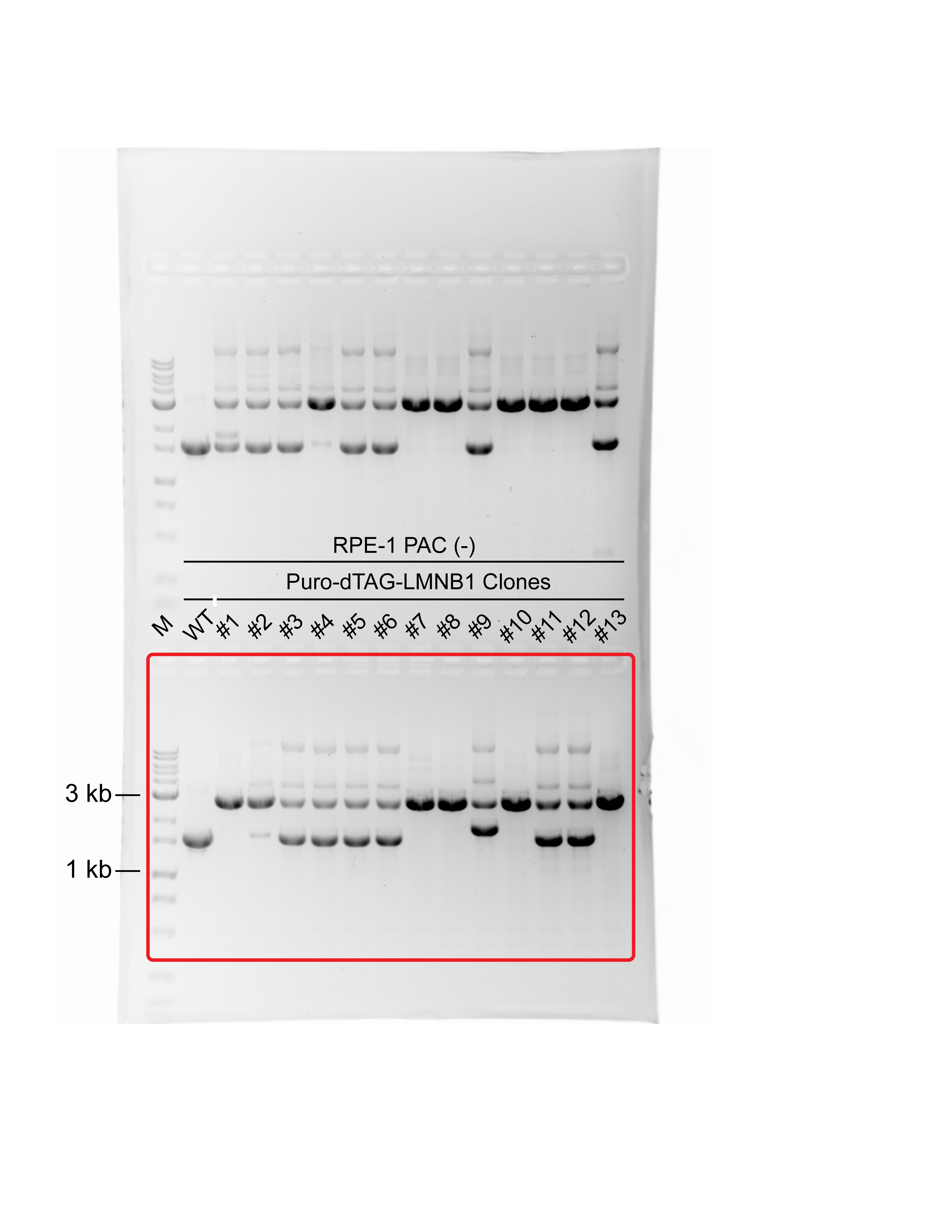

Supplement: Supplementary file 16 — Source data Figure EV4 [file 44318_2024_337_MOESM16_ESM.zip › 10_Figure_EV4/E/dTAG-LMNB1-Clonal-Outcomes.tif]

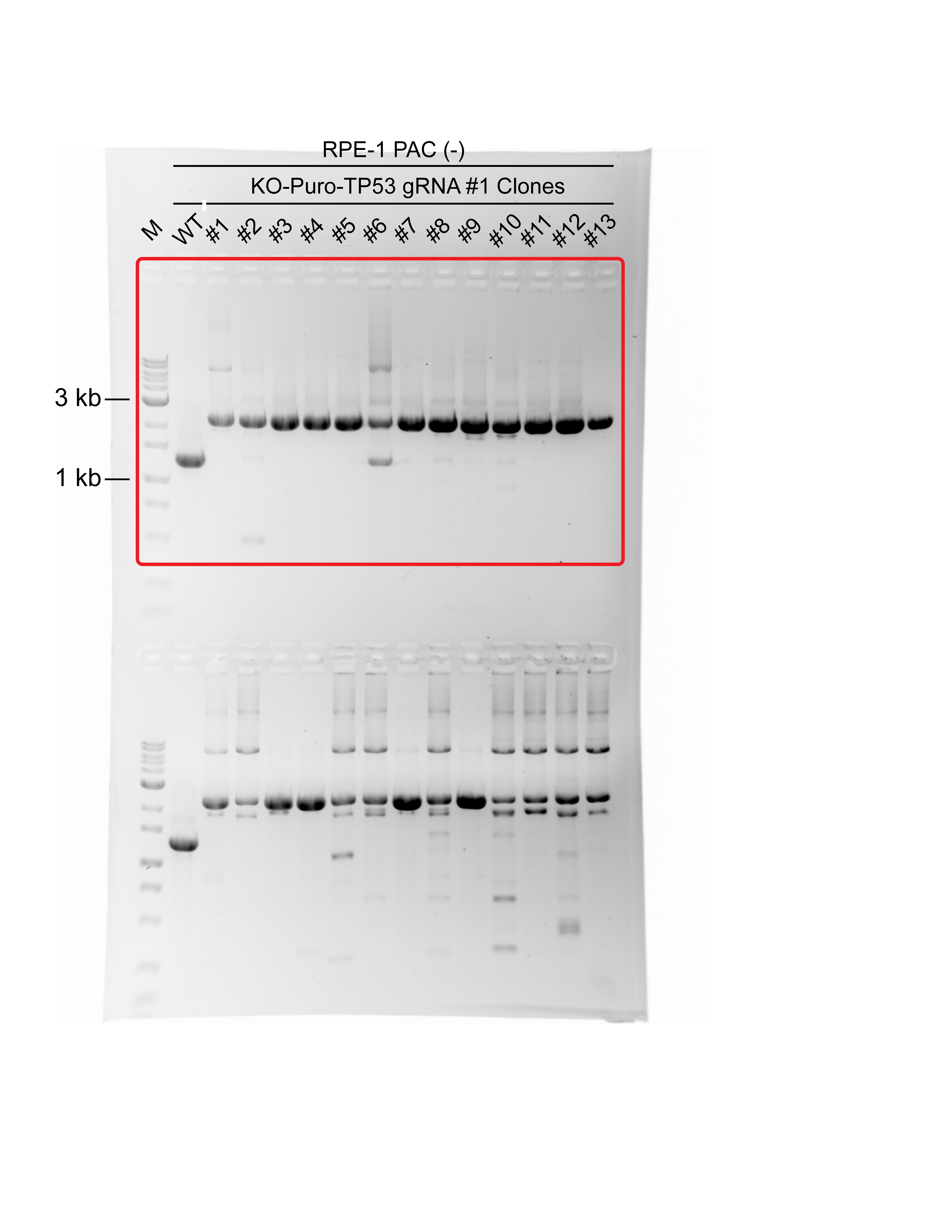

Supplement: Supplementary file 17 — Source data Figure EV5 [file 44318_2024_337_MOESM17_ESM.zip › 11_Figure_EV5/A/KO-TP53-G1-Clonal-Outcomes.tif]

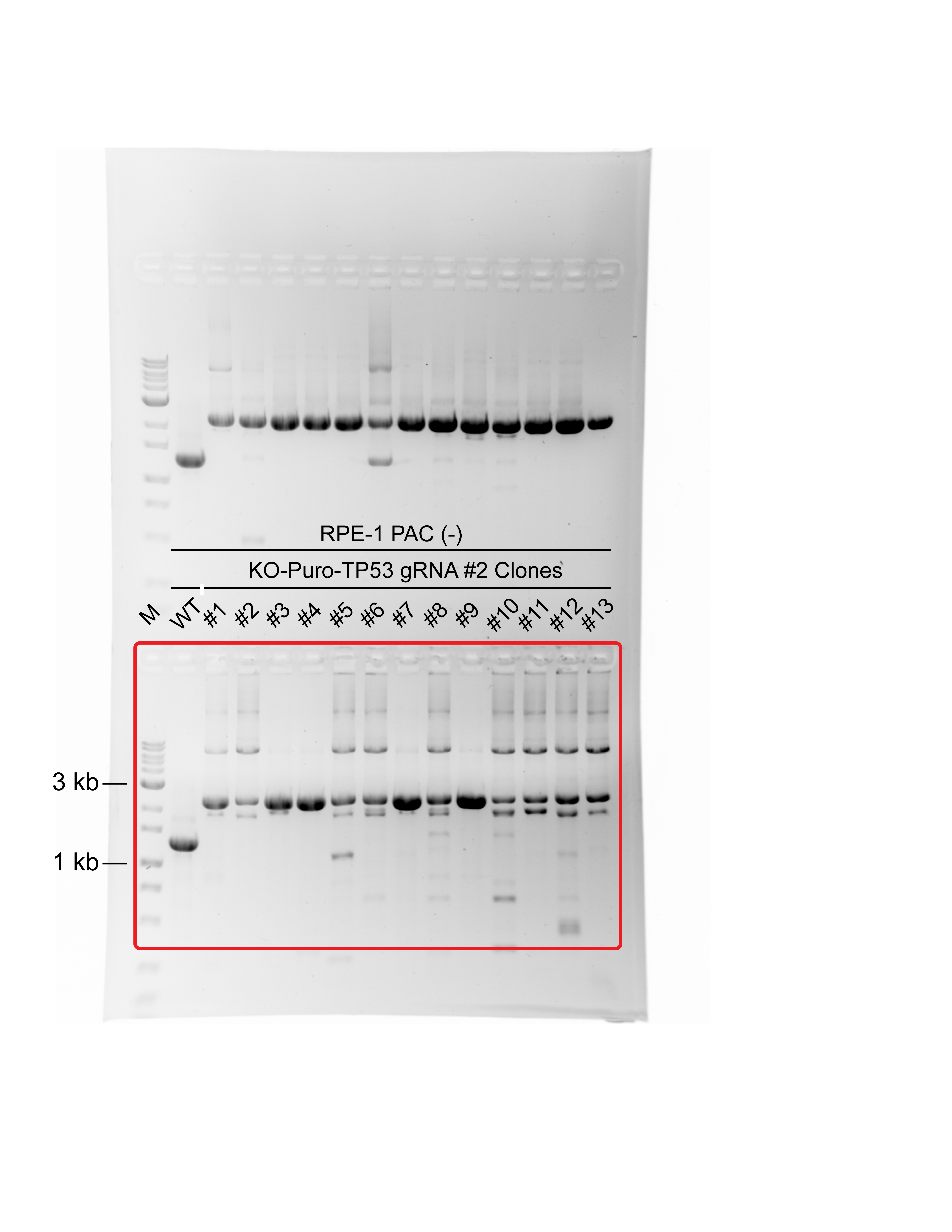

Supplement: Supplementary file 17 — Source data Figure EV5 [file 44318_2024_337_MOESM17_ESM.zip › 11_Figure_EV5/B/KO-TP53-G2-Clonal-Outcomes.tif]

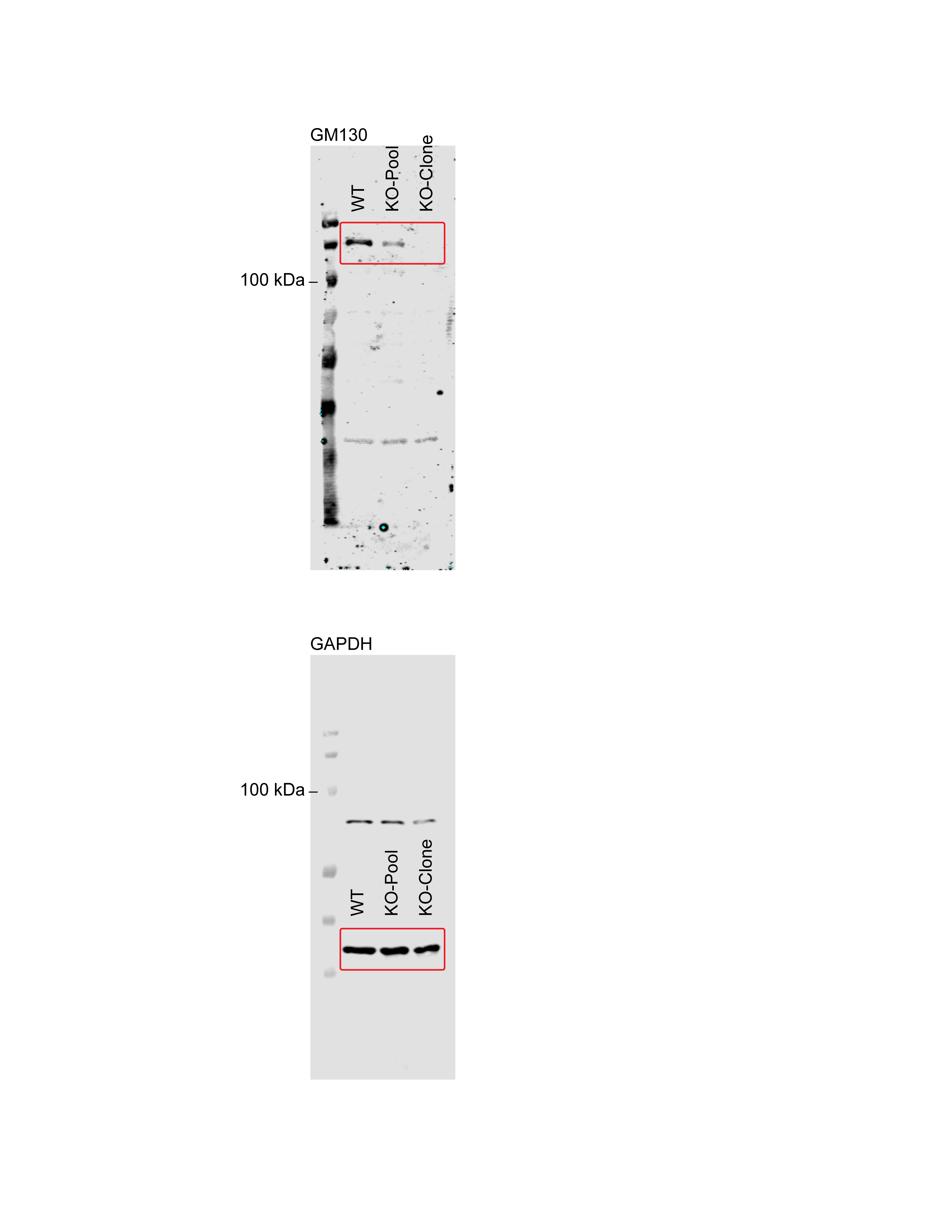

Supplement: Supplementary file 17 — Source data Figure EV5 [file 44318_2024_337_MOESM17_ESM.zip › 11_Figure_EV5/C/GOLGA2-KO-Uncropped-Blot.tif]

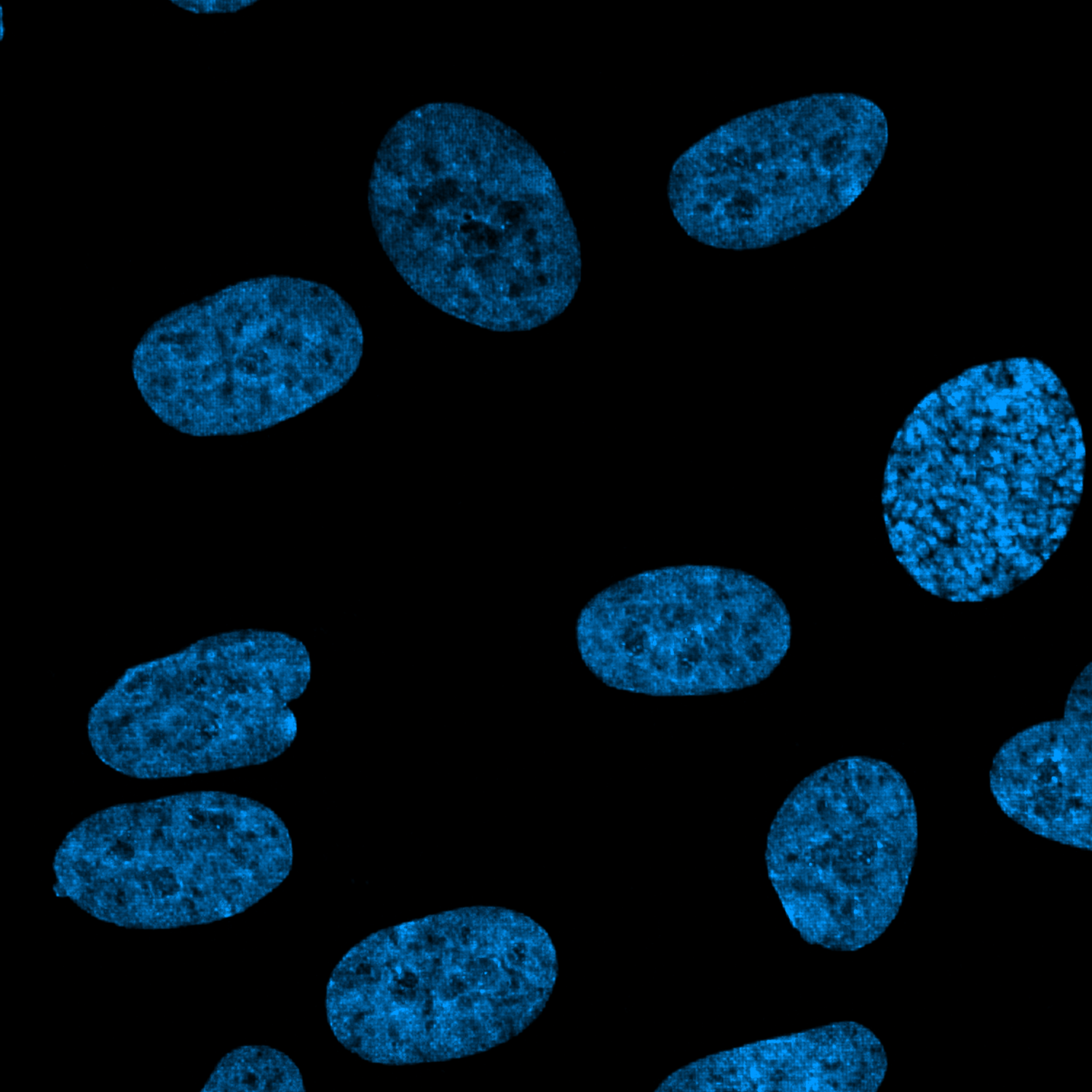

Supplement: Supplementary file 17 — Source data Figure EV5 [file 44318_2024_337_MOESM17_ESM.zip › 11_Figure_EV5/D/GOLGA-KO-CLONE/GOLGA-KO-CLONE_DAPI.tif]
