# Supplementary material for: qTAG: an adaptable plasmid scaffold for CRISPR-based endogenous tagging
Source: EMBO J. 2024 Dec 12;44(3):947–74. doi: 10.1038/s44318-024-00337-5 (PMC11790981; doi:10.1038/s44318-024-00337-5)
Supplement: Supplementary file 14 — Source data Figure EV2 [file 44318_2024_337_MOESM14_ESM.zip › 08_Figure_EV2/C/README.docx]

Sequencing results and summary for Figure EV2,EV3, and EV4 is provided in Dataset EV2.
