# Supplementary figures and images for: qTAG: an adaptable plasmid scaffold for CRISPR-based endogenous tagging (part 5 of 5)
Source: EMBO J. 2024 Dec 12;44(3):947–74. doi: 10.1038/s44318-024-00337-5 (PMC11790981; doi:10.1038/s44318-024-00337-5)

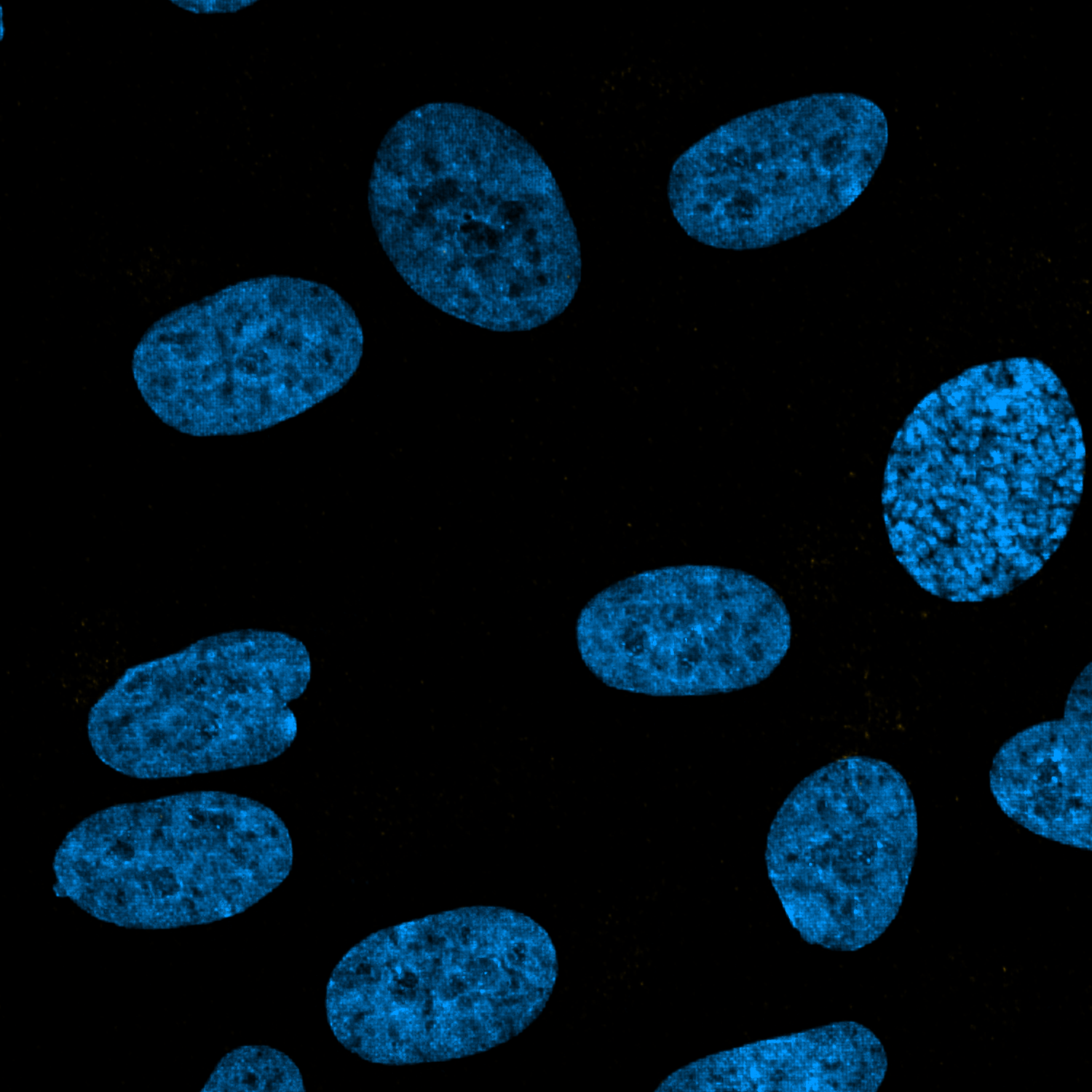

Supplement: Supplementary file 17 — Source data Figure EV5 [file 44318_2024_337_MOESM17_ESM.zip › 11_Figure_EV5/D/GOLGA-KO-CLONE/GOLGA-KO-CLONE_Merge.tif]

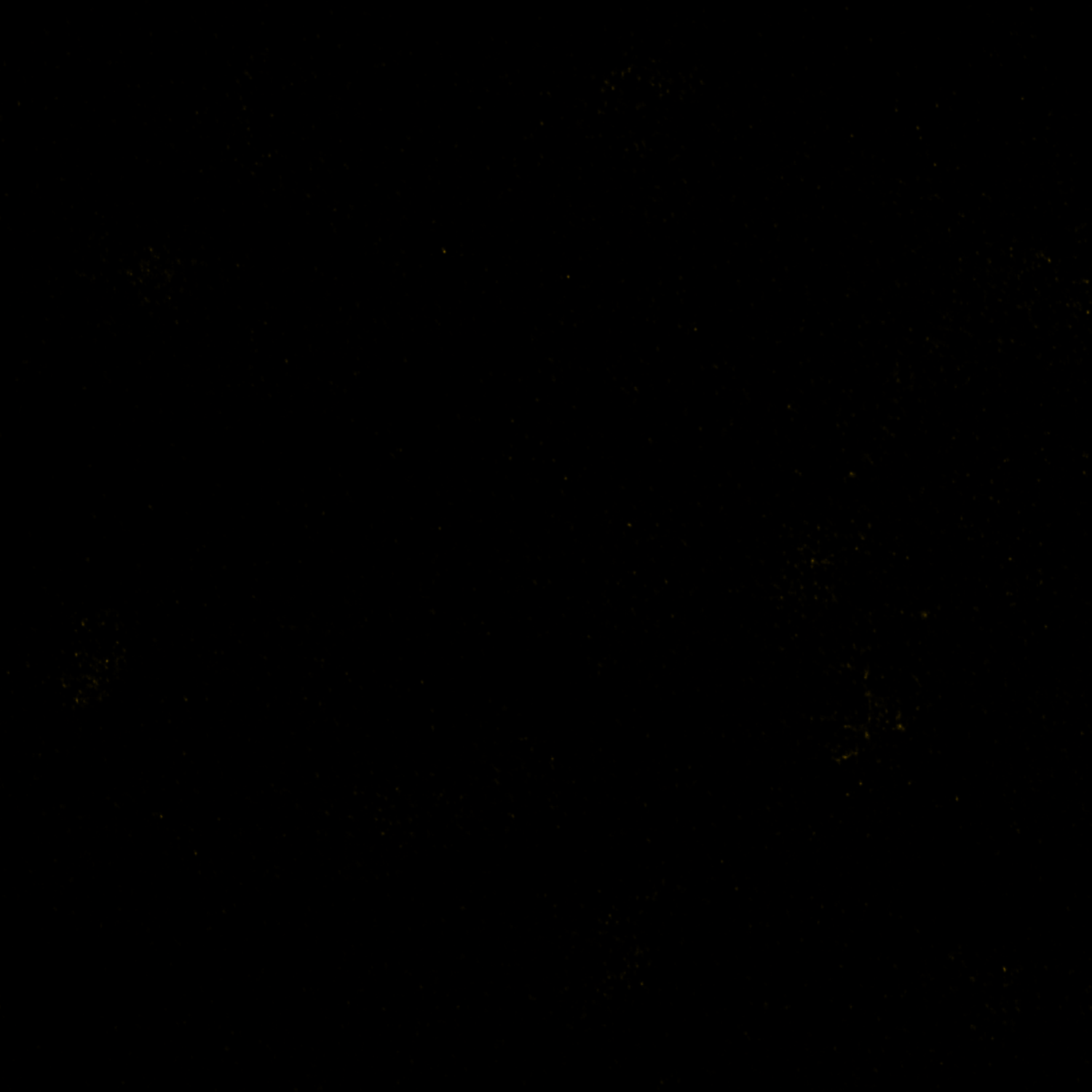

Supplement: Supplementary file 17 — Source data Figure EV5 [file 44318_2024_337_MOESM17_ESM.zip › 11_Figure_EV5/D/GOLGA-KO-CLONE/GOLGA-KO-CLONE_RGB_GM130.tif]

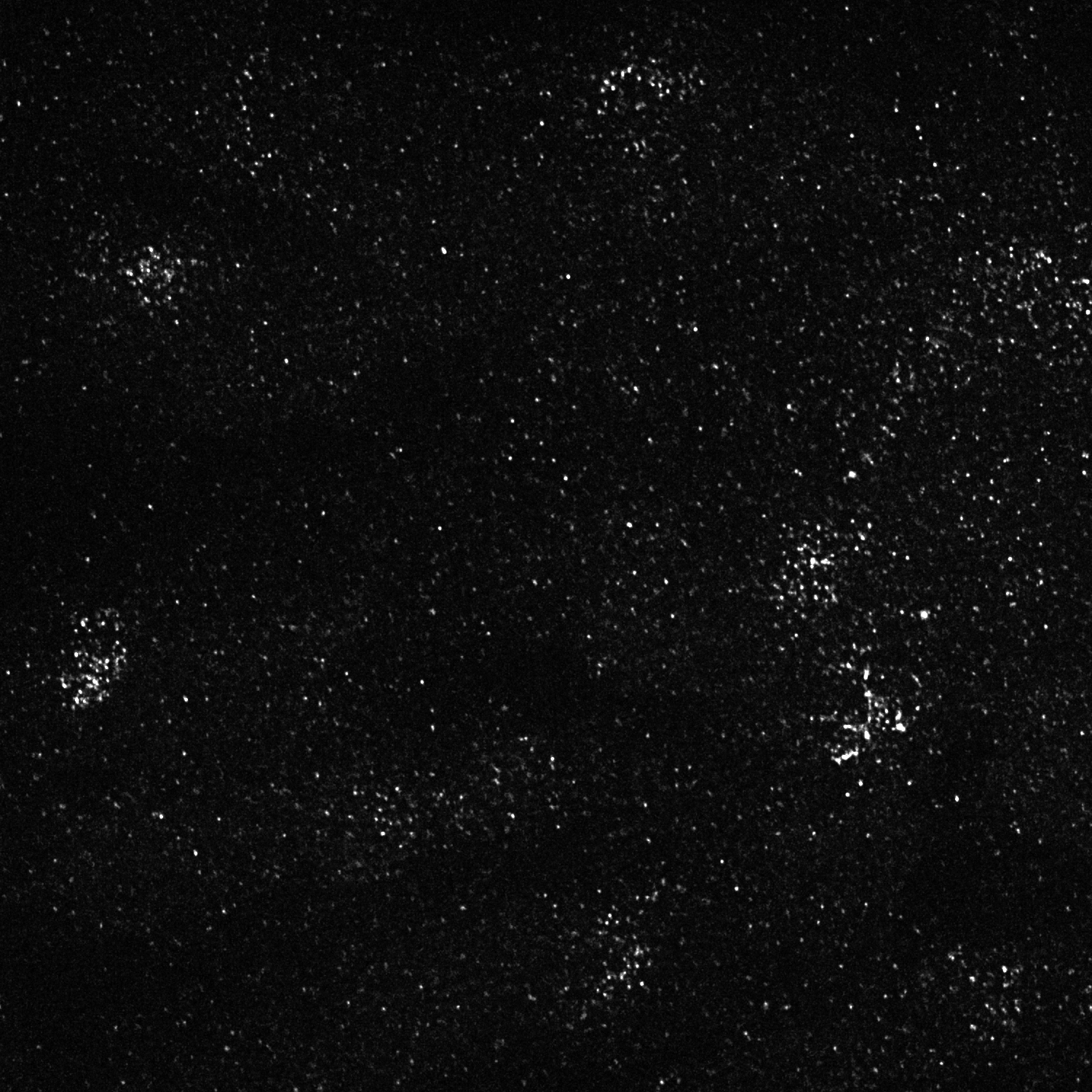

Supplement: Supplementary file 17 — Source data Figure EV5 [file 44318_2024_337_MOESM17_ESM.zip › 11_Figure_EV5/D/GOLGA-KO-CLONE/_FULL-RANGE-GOLGA-KO-CLONE.tif]

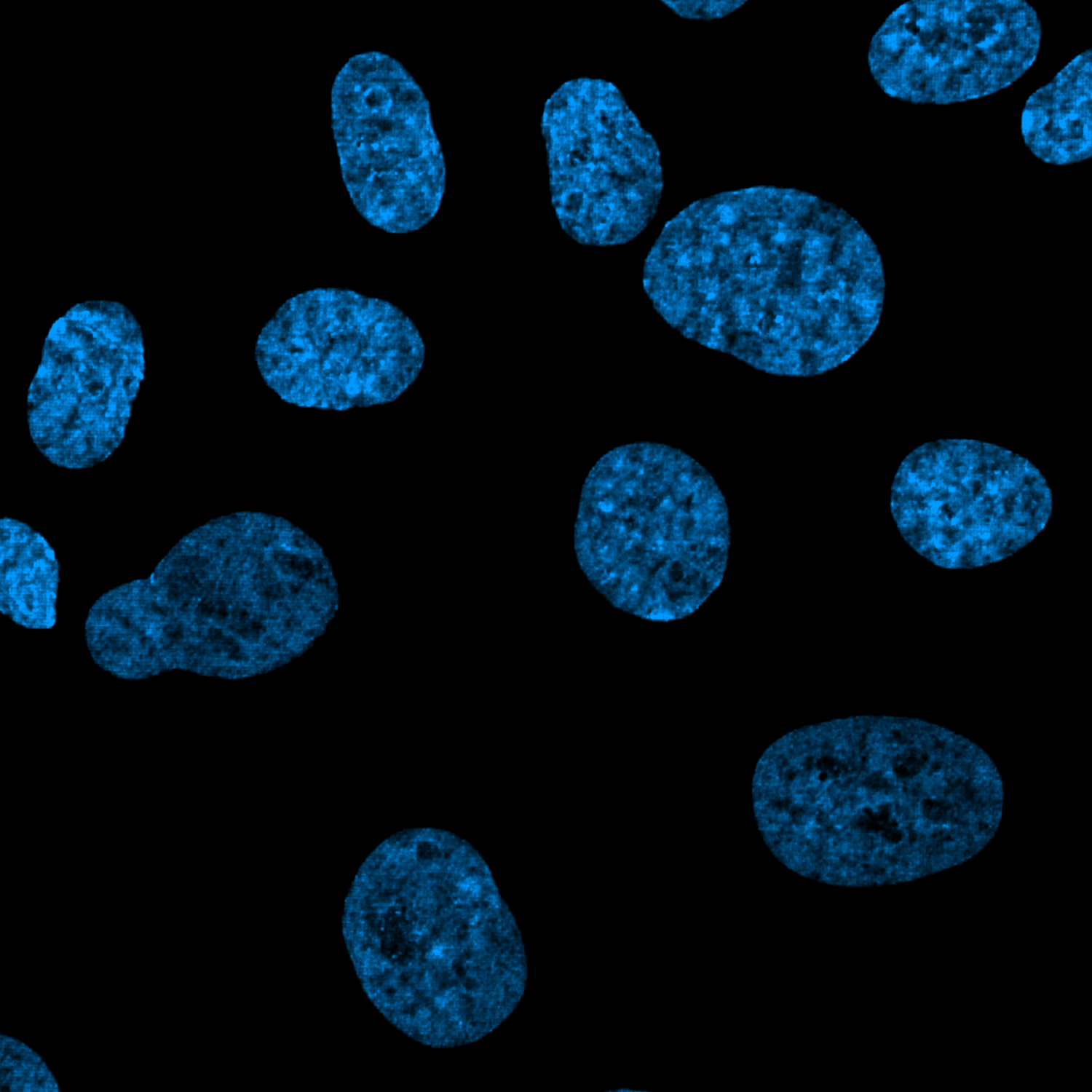

Supplement: Supplementary file 17 — Source data Figure EV5 [file 44318_2024_337_MOESM17_ESM.zip › 11_Figure_EV5/D/GOLGA-KO-POOL/GOLGA-KO-POOL_DAPI.tif]

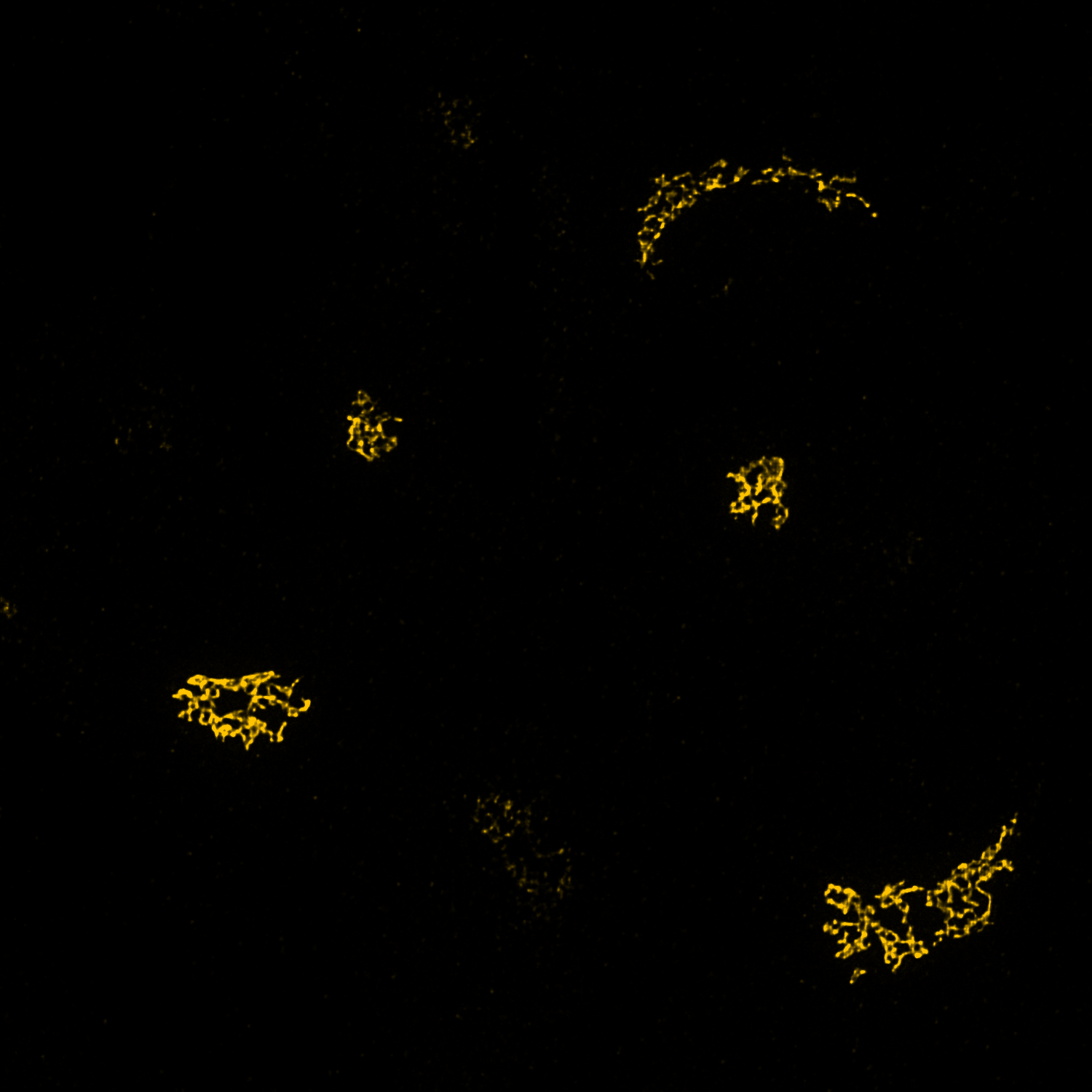

Supplement: Supplementary file 17 — Source data Figure EV5 [file 44318_2024_337_MOESM17_ESM.zip › 11_Figure_EV5/D/GOLGA-KO-POOL/GOLGA-KO-POOL_GM130.tif]

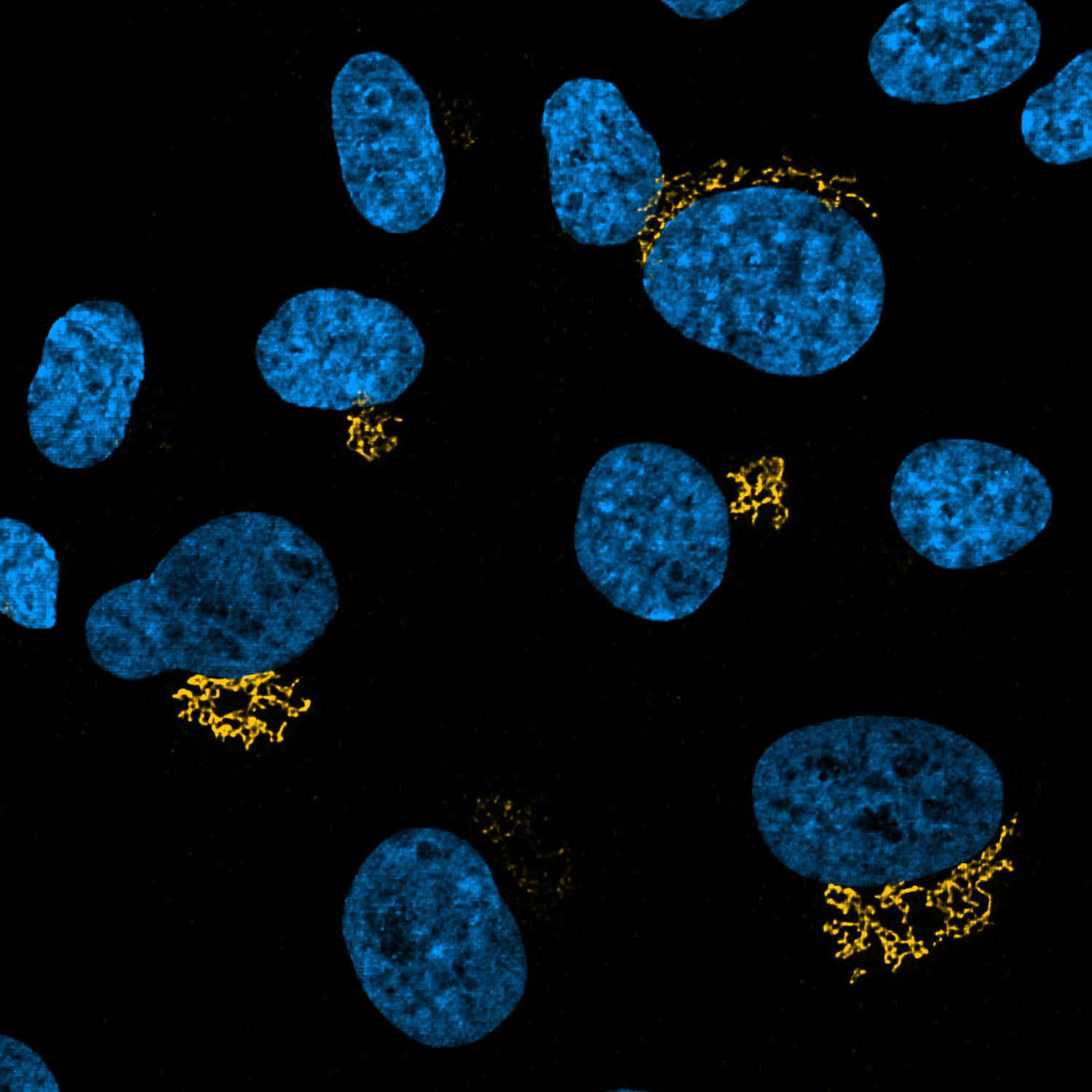

Supplement: Supplementary file 17 — Source data Figure EV5 [file 44318_2024_337_MOESM17_ESM.zip › 11_Figure_EV5/D/GOLGA-KO-POOL/GOLGA-KO-POOL_Merge.tif]

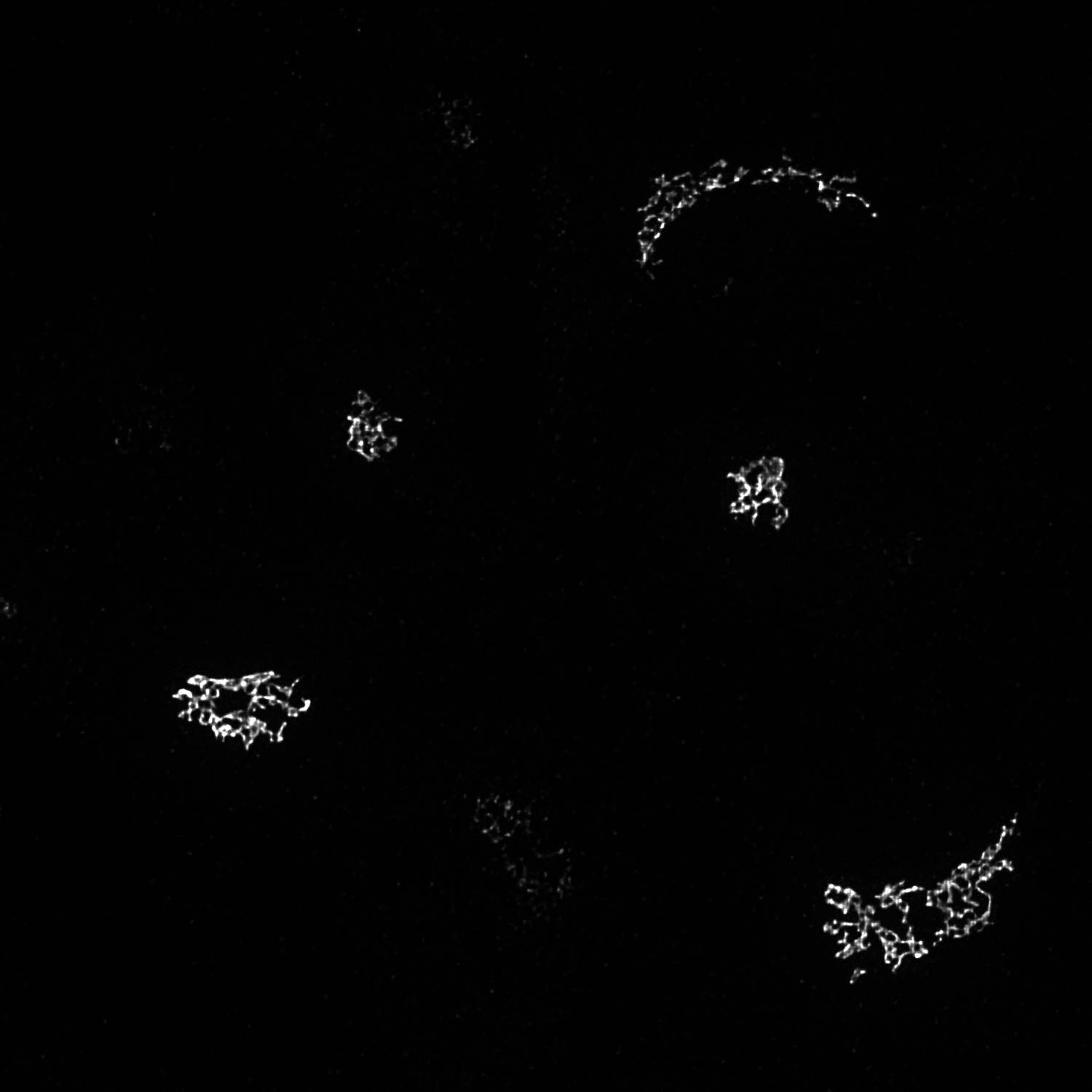

Supplement: Supplementary file 17 — Source data Figure EV5 [file 44318_2024_337_MOESM17_ESM.zip › 11_Figure_EV5/D/GOLGA-KO-POOL/_FULL-RANGE-GOLGA-KO-POOL.tif]

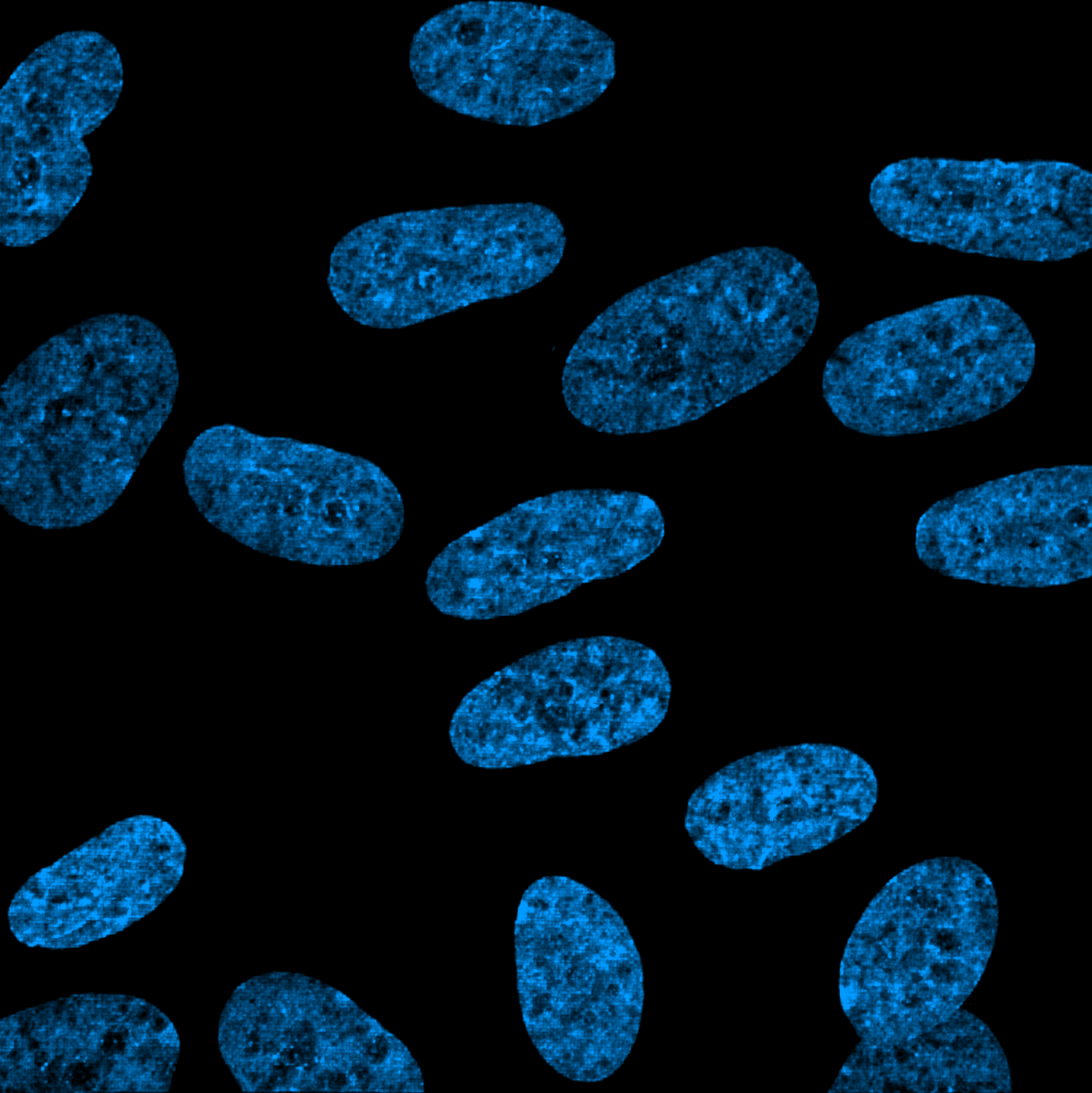

Supplement: Supplementary file 17 — Source data Figure EV5 [file 44318_2024_337_MOESM17_ESM.zip › 11_Figure_EV5/D/GOLGA-WT/GOLGA-WT_DAPI.tif]

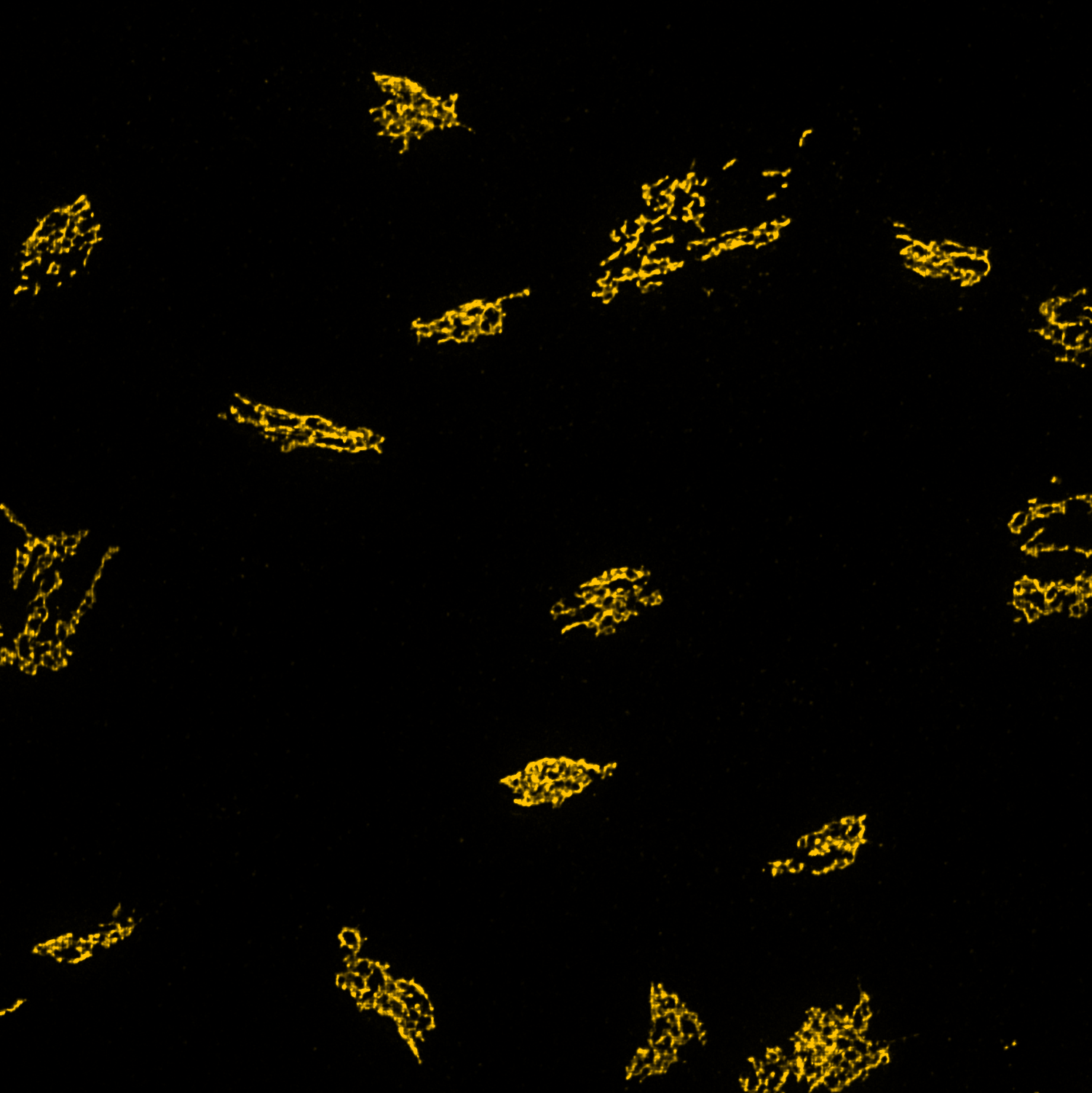

Supplement: Supplementary file 17 — Source data Figure EV5 [file 44318_2024_337_MOESM17_ESM.zip › 11_Figure_EV5/D/GOLGA-WT/GOLGA-WT_GM130.tif]

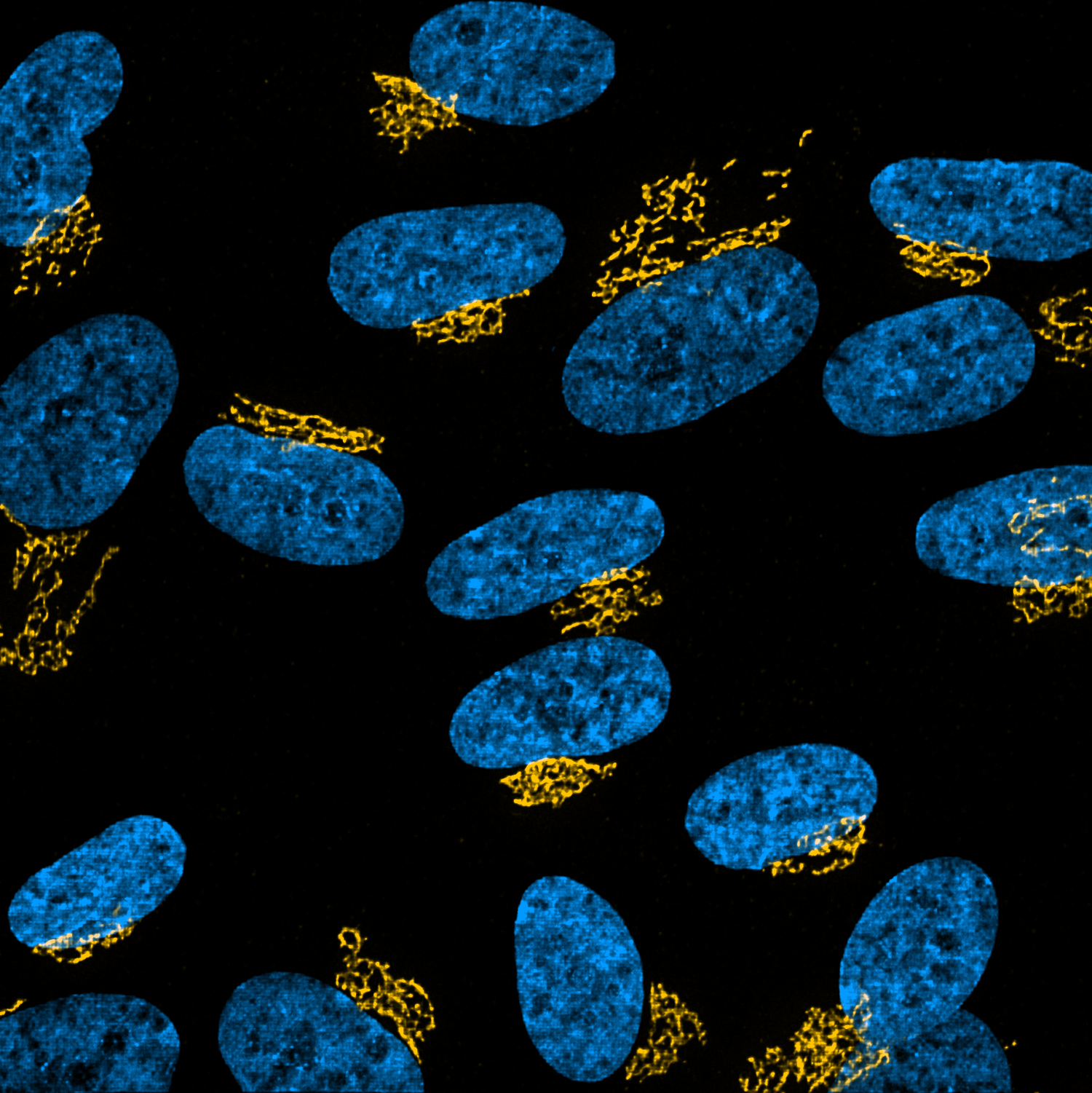

Supplement: Supplementary file 17 — Source data Figure EV5 [file 44318_2024_337_MOESM17_ESM.zip › 11_Figure_EV5/D/GOLGA-WT/GOLGA-WT_Merge.tif]

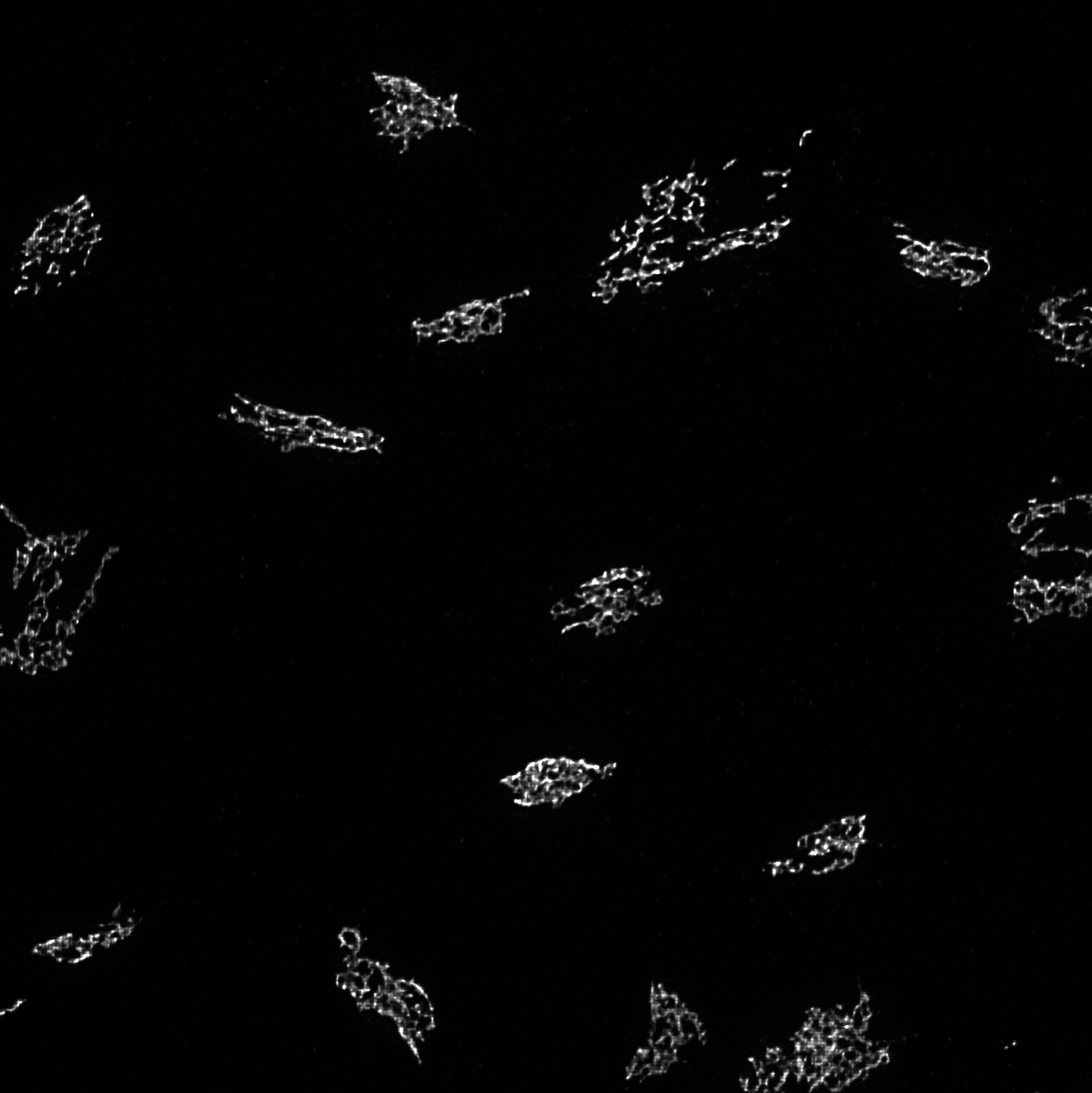

Supplement: Supplementary file 17 — Source data Figure EV5 [file 44318_2024_337_MOESM17_ESM.zip › 11_Figure_EV5/D/GOLGA-WT/_FULL-RANGE-GOLGA-WT.tif]

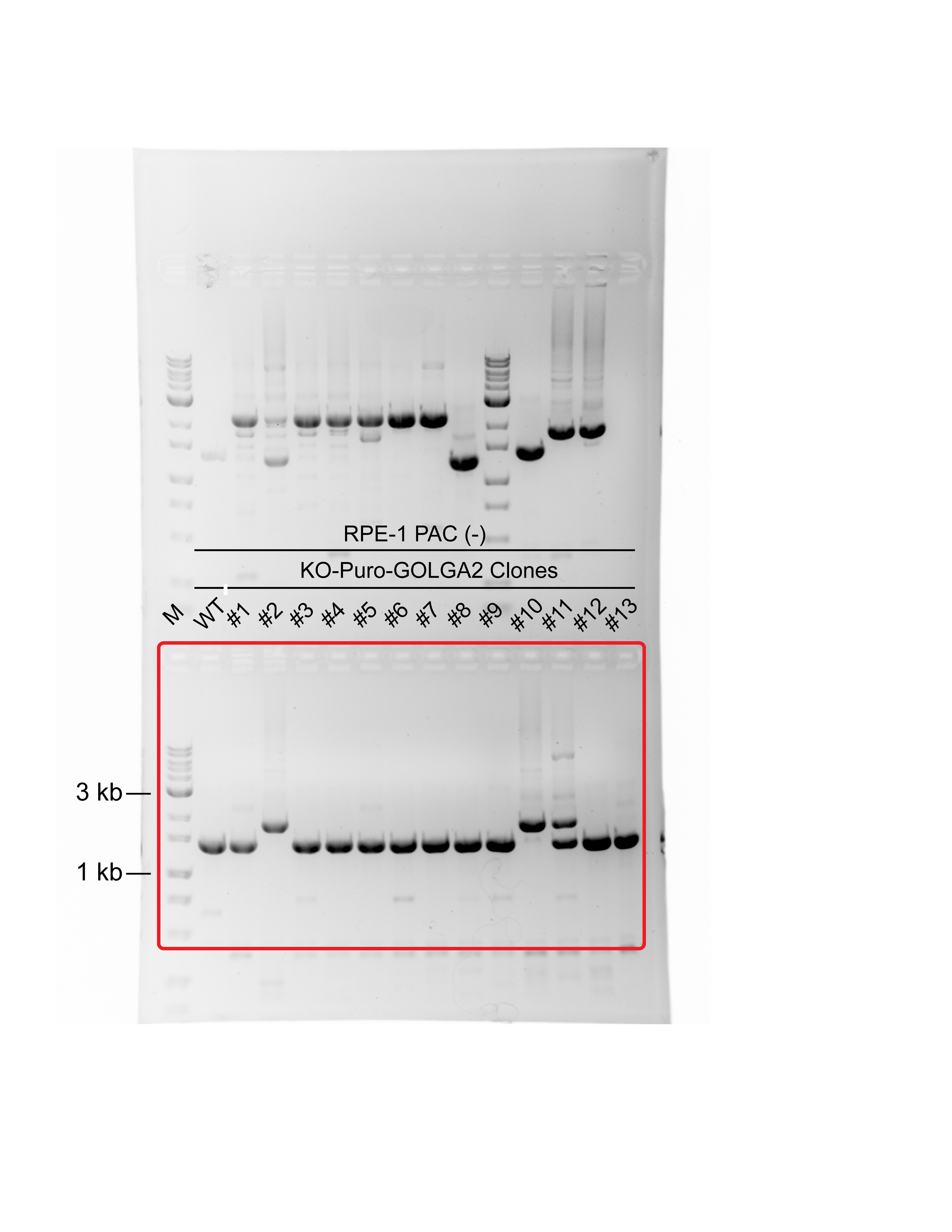

Supplement: Supplementary file 17 — Source data Figure EV5 [file 44318_2024_337_MOESM17_ESM.zip › 11_Figure_EV5/E/KO-GOLGA2-Clonal-Outcomes.tif]
