# Supplementary material for: qTAG: an adaptable plasmid scaffold for CRISPR-based endogenous tagging
Source: EMBO J. 2024 Dec 12;44(3):947–74. doi: 10.1038/s44318-024-00337-5 (PMC11790981; doi:10.1038/s44318-024-00337-5)
Supplement: Supplementary file 18 — Expanded View Figures [file 44318_2024_337_MOESM18_ESM.pdf]

Expanded View Figures

Figure EV1. Editing, enrichment, and validation of fluorescent knock-ins with qTAG cassettes across different human cell lines using various mammalian selections.

(**A–C**) Left—Representative images of WT, HDR, and MMEJ-targeted H2BC11-moxGFP-Puro tagging in HAP1, ARPE-19, and U-2 OS cells co-stained with DAPI. Scale bars: 10 µm. Right—Flow cytometry quantifications of GFP-positive cells, based on three distinct biological replicates with each measurement encompassing 200,000 cells (Right). Data were mean ± SEM. (**D**) Genomic PCR outside the homology arms probing for locus-specific integration of the qTAG cassette in HAP1, ARPE-19, and U-2 OS cells. (**E**) Left—Representative images of WT, selected H2BC11-moxGFP-Blast, selected H2BC11-moxGFP-Puro, and selected H2BC11-moxGFP-Zeo HEK293T cells co-stained with DAPI (Left). Scale bars: 10 µm. Right—Flow cytometry quantifications of GFP-positive cells, based on three distinct biological replicates with each measurement encompassing 200,000 cells (Right). Data were mean ± SEM. (**F**) Genomic PCR outside the homology arms probing for locus-specific integration of the qTAG cassettes with alternative mammalian selectable markers in HEK293T cells. (**G**) Overview of an alternate strategy using electroporation and RNPs to edit the tubulin TUBA1B gene with a qTAG-Blast-mScarlet cassette in H9 stem cells. (**H**) Representative images of WT and Blast-mScarlet-TUBA1B H9 cells co-stained with DAPI and probed for pluripotency marker OCT4. Scale bar: 10 µm. Source data are available online for this figure.

 © The Author(s)

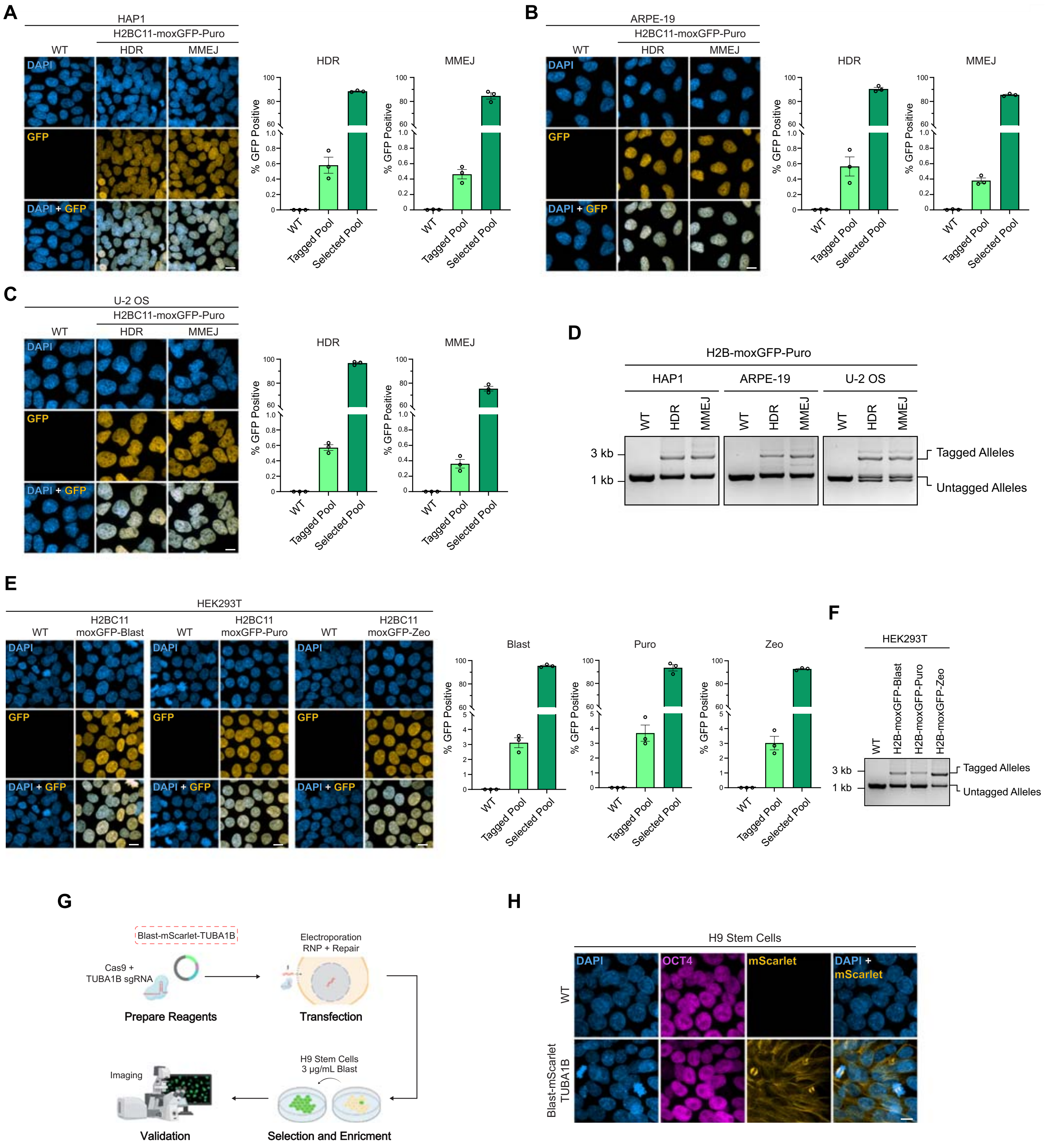
A
HAP1
H2BC11-moxGFP-Puro
WT
HDR
MMEJ
DAPI
GFP
DAPI + GFP
% GFP Positive
WT
Tagged Pool
Selected Pool
B
ARPE-19
C
U-2 OS
D
H2B-moxGFP-Puro
HAP1
ARPE-19
U-2 OS
3 kb
1 kb
Tagged Alleles
Untagged Alleles
E
HEK293T
H2BC11 moxGFP-Blast
H2BC11 moxGFP-Puro
H2BC11 moxGFP-Zeo
Blast
Puro
Zeo
F
HEK293T
H2B-moxGFP-Blast
H2B-moxGFP-Puro
H2B-moxGFP-Zeo
G
Blast-mScarlet-TUBA1B
Cas9 + TUBA1B sgRNA
Electroporation RNP + Repair
Prepare Reagents
Transfection
H9 Stem Cells 3 µg/mL Blast
Imaging
Validation
Selection and Enricment
H
H9 Stem Cells
OCT4
mScarlet
DAPI + mScarlet
Blast-mScarlet TUBA1B

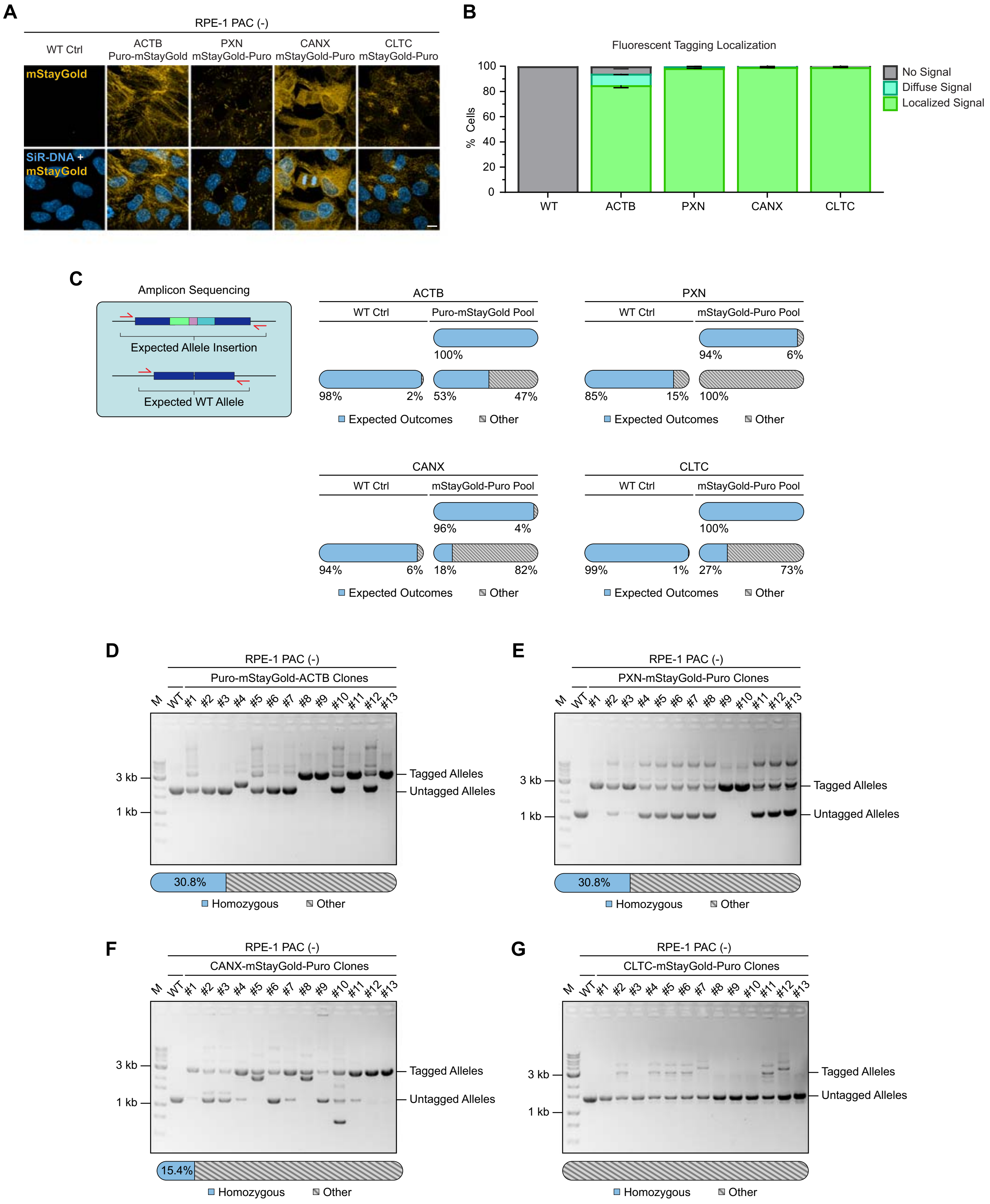
A
RPE-1 PAC (-)
WT Ctrl
ACTB Puro-mStayGold
PXN mStayGold-Puro
CANX mStayGold-Puro
CLTC mStayGold-Puro
mStayGold
SiR-DNA + mStayGold
B
Fluorescent Tagging Localization
% Cells
No Signal
Diffuse Signal
Localized Signal
WT
ACTB
PXN
CANX
CLTC
C
Amplicon Sequencing
Expected Allele Insertion
Expected WT Allele
ACTB
WT Ctrl
Puro-mStayGold Pool
100%
98% 2%
53% 47%
PXN
mStayGold-Puro Pool
94% 6%
85% 15%
100%
CANX
96% 4%
94% 6%
18% 82%
CLTC
100%
99% 1%
27% 73%
Expected Outcomes
Other
D
Puro-mStayGold-ACTB Clones
Tagged Alleles
Untagged Alleles
30.8%
Homozygous
E
PXN-mStayGold-Puro Clones
30.8%
F
CANX-mStayGold-Puro Clones
15.4%
G
CLTC-mStayGold-Puro Clones

 © The Author(s)

Figure EV2. qTAG localization efficiency and editing outcomes using fluorescent cassettes.

(**A**) Representative images of WT, and fluorescently tagged-selected pools of various genes, including ACTB, PXN, CANX, and CLTC co-stained with SiR-DNA. Scale bar: 10 µm. (**B**) Quantification of cells expressing no, diffuse, or localized endogenous fusion signal in antibiotic-selected pools of cells. >500 cells were quantified in each of the three biological replicates for each condition. Data were mean ± SEM. (**C**) Amplicon sequencing results from dominant amplicons in WT and selected edited cell pools. Expected edited outcomes are highlighted in blue, while gray represents the fraction of mutated sequences. (**D–G**) Genomic PCR targeting regions outside the homology arms to assess locus-specific integration of the qTAG cassette, serving as an indicator of clonal efficiency. Homozygous cells are highlighted in blue, while the proportion of other outcomes are shown in gray. Source data are available online for this figure.

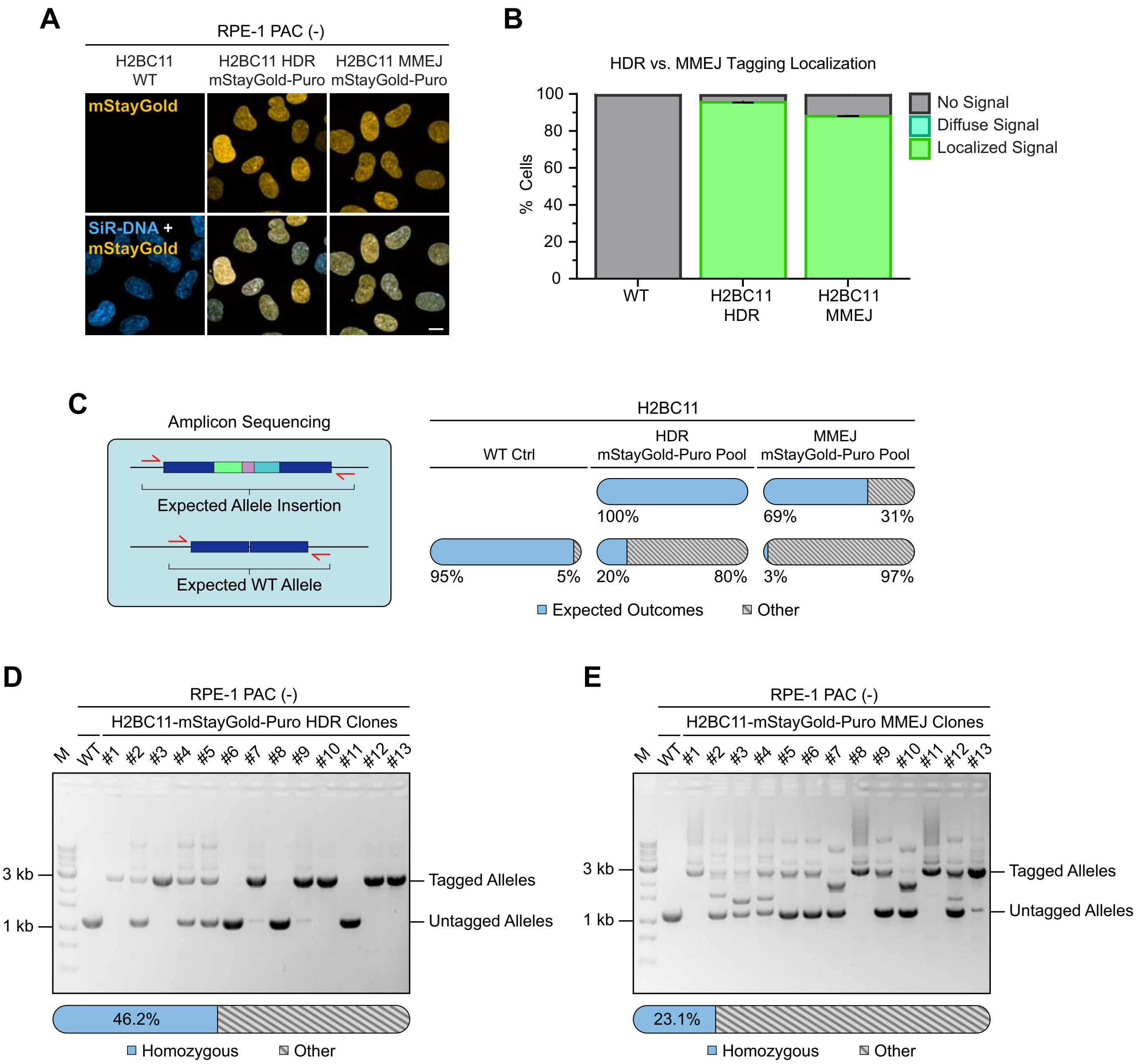

Figure EV3. qTAG localization efficiency and editing outcomes using cassettes targeting the HDR pathway vs MMEJ pathway.

(**A**) Representative images of WT, and fluorescently tagged-selected pools H2BC11-mStayGold edited with cassettes targeting the HDR pathway or MMEJ pathway. Scale bars: 10 µm. (**B**) Quantification of cells expressing no, diffuse, or localized endogenous fusion signal in antibiotic-selected pools of cells. >500 cells were quantified in each of the three biological replicates for each condition. Data were mean ± SEM. (**C**) Amplicon sequencing results from dominant amplicons in WT and selected edited cell pools. Expected edited outcomes are highlighted in blue, while gray represents the fraction of mutated sequences. (**D**, **E**) Genomic PCR targeting regions outside the homology arms to assess locus-specific integration of the qTAG cassette, serving as an indicator of clonal efficiency. Homozygous cells are highlighted in blue, while the proportion of other outcomes are shown in gray. Source data are available online for this figure.

 © The Author(s)

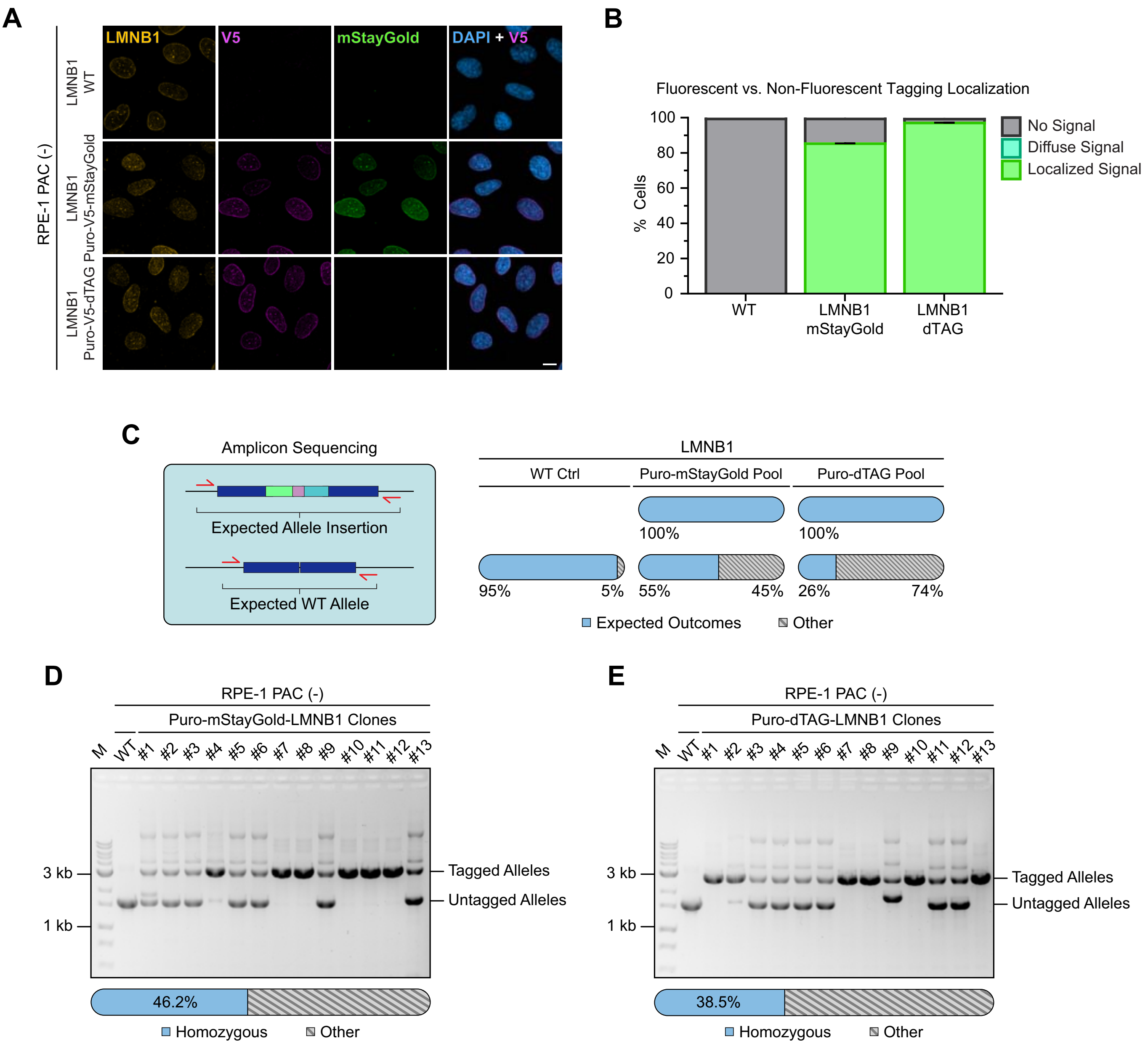

Figure EV4. qTAG localization efficiency and editing outcomes using fluorescent tagging cassettes vs. non-fluorescent tagging cassettes.

(**A**) Representative images of WT, fluorescently tagged-selected pools of mStayGold-LMNB1, and non-fluorescently tagged-selected pools of dTAG-LMNB1. Scale bar: 10 µm. (**B**) Quantification of cells expressing no, diffuse, or localized endogenous fusion signal in antibiotic-selected pools of cells. >500 cells were quantified in each of the three biological replicates for each condition. Data were mean ± SEM. (**C**) Amplicon sequencing results from dominant amplicons in WT and selected edited cell pools. Expected edited outcomes are highlighted in blue, while gray represents the fraction of mutated sequences. (**D**, **E**) Genomic PCR targeting regions outside the homology arms to assess locus-specific integration of the qTAG cassette, serving as an indicator of clonal efficiency. Homozygous cells are highlighted in blue, while the proportion of other outcomes are shown in gray. Source data are available online for this figure.

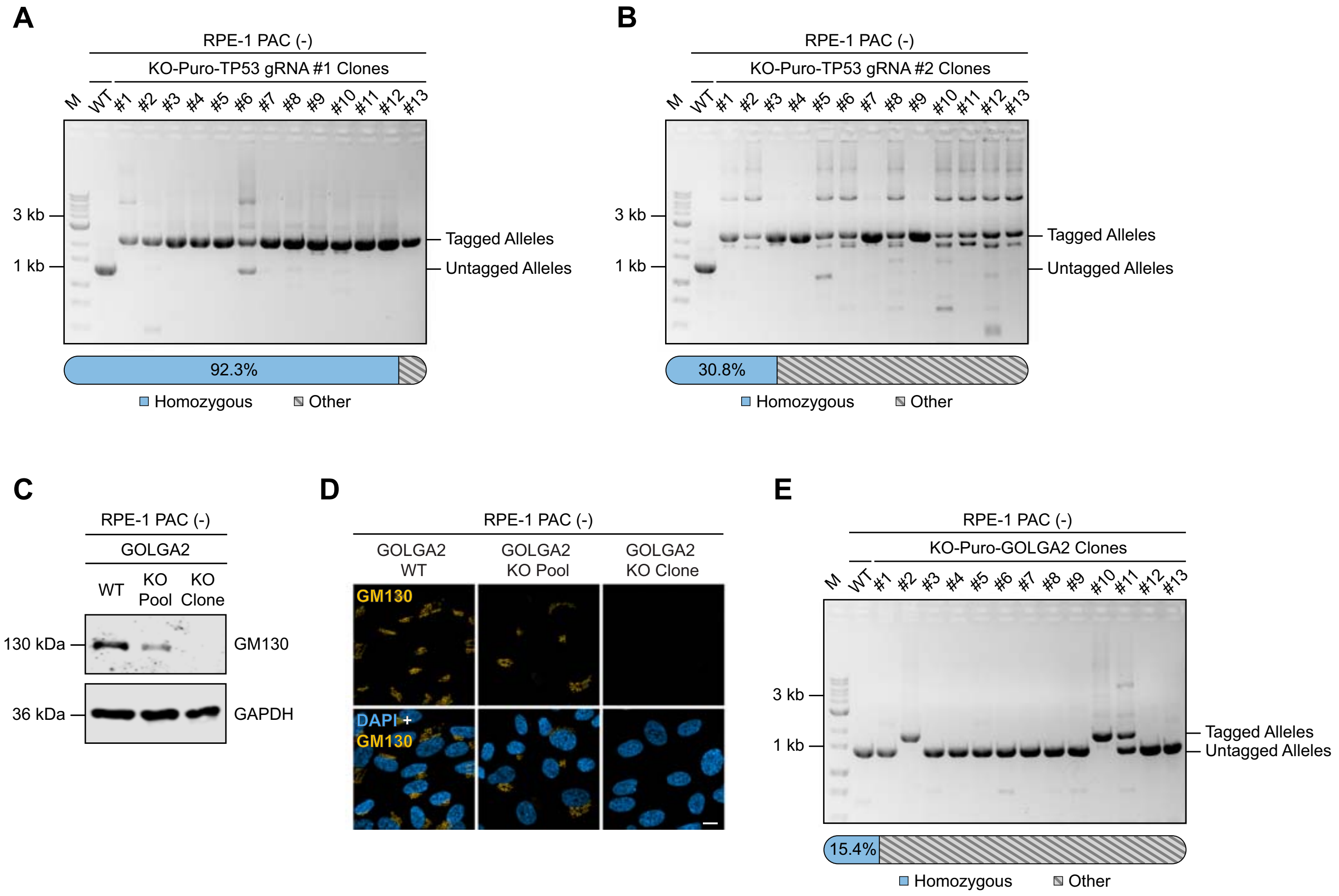

Figure EV5. Knockout tagging clonal efficiency.

(**A**, **B**) Genomic PCR targeting regions outside the homology arms of TP53 to assess locus-specific integration of the qTAG cassette, serving as an indicator of clonal efficiency. Homozygous cells are highlighted in blue, while the proportion of other outcomes are shown in gray. (**C**) A representative immunoblot of RPE-1 PAC (−) WT-GOLGA2 cells, a GOLGA2-KO-Puro selected pool of cells and a GOLGA2-KO-Puro selected clonal cell line probed with antibodies against GM130 and GAPDH. (**D**) Representative images of WT-GOLGA2 cells, a GOLGA2-KO-Puro selected pool of cells, and a GOLGA2-KO-Puro selected clonal cell line probed with an antibody against GM130 and co-stained with DAPI. Scale bar: 10 µm. (**E**) Genomic PCR targeting regions outside the homology arms of GOLGA2 to assess locus-specific integration of the qTAG cassette. Homozygous cells are highlighted in blue, while the proportion of other outcomes are shown in gray. Source data are available online for this figure.

© The Author(s)
